# Spatial, temporal, and demographic patterns in prevalence of smoking tobacco use and attributable disease burden in 204 countries and territories, 1990–2019: a systematic analysis from the Global Burden of Disease Study 2019

**DOI:** 10.1016/S0140-6736(21)01169-7

**Published:** 2021-06-19

**Authors:** GBD 2019 Tobacco Collaborators, Marissa B Reitsma, Parkes J Kendrick, Emad Ababneh, Cristiana Abbafati, Mohsen Abbasi-Kangevari, Amir Abdoli, Aidin Abedi, E S Abhilash, Derrick Bary Abila, Victor Aboyans, Niveen ME Abu-Rmeileh, Oladimeji M Adebayo, Shailesh M Advani, Mohammad Aghaali, Bright Opoku Ahinkorah, Sohail Ahmad, Keivan Ahmadi, Haroon Ahmed, Budi Aji, Chisom Joyqueenet Akunna, Ziyad Al-Aly, Turki M Alanzi, Khalid F Alhabib, Liaqat Ali, Sheikh Mohammad Alif, Vahid Alipour, Syed Mohamed Aljunid, François Alla, Peter Allebeck, Nelson Alvis-Guzman, Tarek Tawfik Amin, Saeed Amini, Hubert Amu, Gianna Gayle Herrera Amul, Robert Ancuceanu, Jason A Anderson, Alireza Ansari-Moghaddam, Carl Abelardo T Antonio, Benny Antony, Davood Anvari, Jalal Arabloo, Nicholas D Arian, Monika Arora, Malke Asaad, Marcel Ausloos, Asma Tahir Awan, Getinet Ayano, Getie Lake Aynalem, Samad Azari, Darshan B B, Ashish D Badiye, Atif Amin Baig, Mohammad Hossein Bakhshaei, Maciej Banach, Palash Chandra Banik, Suzanne Lyn Barker-Collo, Till Winfried Bärnighausen, Hiba Jawdat Barqawi, Sanjay Basu, Mohsen Bayati, Shahrzad Bazargan-Hejazi, Masoud Behzadifar, Tariku Tesfaye Bekuma, Derrick A Bennett, Isabela M Bensenor, Kathleen S Sachiko Berfield, Akshaya Srikanth Bhagavathula, Nikha Bhardwaj, Pankaj Bhardwaj, Krittika Bhattacharyya, Sadia Bibi, Ali Bijani, Bagas Suryo Bintoro, Antonio Biondi, Setognal Birara, Dejana Braithwaite, Hermann Brenner, Andre R Brunoni, Katrin Burkart, Zahid A Butt, Florentino Luciano Caetano dos Santos, Luis Alberto Cámera, Josip Car, Rosario Cárdenas, Giulia Carreras, Juan J Carrero, Joao Mauricio Castaldelli-Maia, Maria Sofia Sofia Cattaruzza, Jung-Chen Chang, Simiao Chen, Dinh-Toi Chu, Sheng-Chia Chung, Massimo Cirillo, Vera Marisa Costa, Rosa A S Couto, Omid Dadras, Xiaochen Dai, Albertino Antonio Moura Damasceno, Giovanni Damiani, Lalit Dandona, Rakhi Dandona, Parnaz Daneshpajouhnejad, Jiregna Darega Gela, Kairat Davletov, Meseret Derbew Molla, Getenet Ayalew Dessie, Abebaw Alemayehu Desta, Samath Dhamminda Dharmaratne, Mostafa Dianatinasab, Daniel Diaz, Hoa Thi Do, Abdel Douiri, Bruce B Duncan, Andre Rodrigues Duraes, Arielle Wilder Eagan, Mohammad Ebrahimi Kalan, Kristina Edvardsson, Iffat Elbarazi, Maha El Tantawi, Saman Esmaeilnejad, Ibtihal Fadhil, Emerito Jose A Faraon, Carla Sofia e Sá Farinha, Medhat Farwati, Farshad Farzadfar, Mehdi Fazlzadeh, Valery L Feigin, Rachel Feldman, Carlota Fernandez Prendes, Pietro Ferrara, Irina Filip, Filippos Filippidis, Florian Fischer, Luisa Sorio Flor, Nataliya A Foigt, Morenike Oluwatoyin Folayan, Masoud Foroutan, Mohamed M Gad, Abhay Motiramji Gaidhane, Silvano Gallus, Biniyam Sahiledengle Geberemariyam, Mansour Ghafourifard, Alireza Ghajar, Ahmad Ghashghaee, Simona Giampaoli, Paramjit Singh Gill, Franklin N Glozah, Elena V Gnedovskaya, Mahaveer Golechha, Sameer Vali Gopalani, Giuseppe Gorini, Houman Goudarzi, Alessandra C Goulart, Felix Greaves, Avirup Guha, Yuming Guo, Bhawna Gupta, Rajat Das Gupta, Rajeev Gupta, Tarun Gupta, Vin Gupta, Nima Hafezi-Nejad, Mohammad Rifat Haider, Randah R Hamadeh, Graeme J Hankey, Arief Hargono, Risky Kusuma Hartono, Hadi Hassankhani, Simon I Hay, Golnaz Heidari, Claudiu Herteliu, Kamal Hezam, Thomas R Hird, Michael K Hole, Ramesh Holla, Mehdi Hosseinzadeh, Sorin Hostiuc, Mowafa Househ, Thomas Hsiao, Junjie Huang, Vincent C Iannucci, Segun Emmanuel Ibitoye, Bulat Idrisov, Olayinka Stephen Ilesanmi, Irena M Ilic, Milena D Ilic, Leeberk Raja Inbaraj, Seyed Sina Naghibi Irvani, Jessica Y Islam, Rakibul M Islam, Sheikh Mohammed Shariful Islam, Farhad Islami, Hiroyasu Iso, Ramaiah Itumalla, Masao Iwagami, Jalil Jaafari, Vardhmaan Jain, Mihajlo Jakovljevic, Sung-In Jang, Hosna Janjani, Shubha Jayaram, Panniyammakal Jeemon, Ravi Prakash Jha, Jost B Jonas, Tamas Joo, Mikk Jürisson, Ali Kabir, Zubair Kabir, Leila R Kalankesh, Tanuj Kanchan, Himal Kandel, Neeti Kapoor, Salah Eddin Karimi, Srinivasa Vittal Katikireddi, Hafte Kahsay Kebede, Bayew Kelkay, Ryan David Kennedy, Abdullah T Khoja, Jagdish Khubchandani, Gyu Ri Kim, Young-Eun Kim, Ruth W Kimokoti, Mika Kivimäki, Soewarta Kosen, Sindhura Lakshmi Koulmane Laxminarayana, Ai Koyanagi, Kewal Krishan, Nuworza Kugbey, G Anil Kumar, Nithin Kumar, Om P Kurmi, Dian Kusuma, Ben Lacey, Jennifer O Lam, Iván Landires, Savita Lasrado, Paolo Lauriola, Doo Woong Lee, Yo Han Lee, Janni Leung, Shanshan Li, Hualiang Lin, Shai Linn, Wei Liu, Alan D Lopez, Platon D Lopukhov, Stefan Lorkowski, Alessandra Lugo, Azeem Majeed, Afshin Maleki, Reza Malekzadeh, Deborah Carvalho Malta, Abdullah A Mamun, Narayana Manjunatha, Borhan Mansouri, Mohammad Ali Mansournia, Jose Martinez-Raga, Santi Martini, Manu Raj Mathur, Carlo Eduardo Medina-Solís, Suresh Mehata, Walter Mendoza, Ritesh G Menezes, Atte Meretoja, Tuomo J Meretoja, Bartosz Miazgowski, Irmina Maria Michalek, Ted R Miller, Erkin M Mirrakhimov, Hamed Mirzaei, Mehdi Mirzaei-Alavijeh, Sanjeev Misra, Masoud Moghadaszadeh, Yousef Mohammad, Abdollah Mohammadian-Hafshejani, Shafiu Mohammed, Ali H Mokdad, Lorenzo Monasta, Mohammad Ali Moni, Ghobad Moradi, Maziar Moradi-Lakeh, Rahmatollah Moradzadeh, Shane Douglas Morrison, Tilahun Belete Mossie, Sumaira Mubarik, Erin C Mullany, Christopher J L Murray, Mohsen Naghavi, Behshad Naghshtabrizi, Sanjeev Nair, Mahdi Nalini, Vinay Nangia, Atta Abbas Naqvi, Sreenivas Narasimha Swamy, Muhammad Naveed, Smitha Nayak, Vinod C Nayak, Javad Nazari, Sabina O Nduaguba, Sandhya Neupane Kandel, Cuong Tat Nguyen, Huong Lan Thi Nguyen, Son Hoang Nguyen, Trang Huyen Nguyen, Molly R Nixon, Chukwudi A Nnaji, Bo Norrving, Jean Jacques Noubiap, Christoph Nowak, Felix Akpojene Ogbo, Ayodipupo Sikiru Oguntade, In-Hwan Oh, Andrew T Olagunju, Eyal Oren, Nikita Otstavnov, Stanislav S Otstavnov, Mayowa O Owolabi, Mahesh P A, Smita Pakhale, Keyvan Pakshir, Raffaele Palladino, Adrian Pana, Songhomitra Panda-Jonas, Ashok Pandey, Utsav Parekh, Eun-Cheol Park, Eun-Kee Park, Fatemeh Pashazadeh Kan, George C Patton, Shrikant Pawar, Richard G Pestell, Marina Pinheiro, Michael A Piradov, Saeed Pirouzpanah, Khem Narayan Pokhrel, Roman V Polibin, Akila Prashant, Dimas Ria Angga Pribadi, Amir Radfar, Vafa Rahimi-Movaghar, Azizur Rahman, Mohammad Hifz Ur Rahman, Muhammad Aziz Rahman, Amir Masoud Rahmani, Nazanin Rajai, Pradhum Ram, Chhabi Lal Ranabhat, Priya Rathi, Lal Rawal, Andre M N Renzaho, Luz Myriam Reynales-Shigematsu, Aziz Rezapour, Seyed Mohammad Riahi, Mavra A Riaz, Leonardo Roever, Luca Ronfani, Gholamreza Roshandel, Ambuj Roy, Bedanta Roy, Simona Sacco, Basema Saddik, Amirhossein Sahebkar, Sana Salehi, Hamideh Salimzadeh, Mehrnoosh Samaei, Abdallah M Samy, Itamar S Santos, Milena M Santric-Milicevic, Nizal Sarrafzadegan, Brijesh Sathian, Monika Sawhney, Mete Saylan, Michael P Schaub, Maria Inês Schmidt, Ione Jayce Ceola Schneider, Aletta Elisabeth Schutte, Falk Schwendicke, Abdul-Aziz Seidu, Nachimuthu Senthil Kumar, Sadaf G Sepanlou, Allen Seylani, Omid Shafaat, Syed Mahboob Shah, Masood Ali Shaikh, Ali S Shalash, Mohammed Shannawaz, Kiomars Sharafi, Aziz Sheikh, Sara Sheikhbahaei, Mika Shigematsu, Rahman Shiri, Kawkab Shishani, K M Shivakumar, Siddharudha Shivalli, Roman Shrestha, Soraya Siabani, Negussie Boti Sidemo, Inga Dora Sigfusdottir, Rannveig Sigurvinsdottir, Diego Augusto Santos Silva, João Pedro Silva, Ambrish Singh, Jasvinder A Singh, Virendra Singh, Dhirendra Narain Sinha, Freddy Sitas, Valentin Yurievich Skryabin, Anna Aleksandrovna Skryabina, Matiwos Soboka, Joan B Soriano, Ali Soroush, Sergey Soshnikov, Ireneous N Soyiri, Emma Elizabeth Spurlock, Chandrashekhar T Sreeramareddy, Dan J Stein, Paschalis Steiropoulos, Stefan Stortecky, Kurt Straif, Rizwan Suliankatchi Abdulkader, Gerhard Sulo, Johan Sundström, Takahiro Tabuchi, Santosh Kumar Tadakamadla, Biruk Wogayehu Taddele, Eyayou Girma Tadesse, Animut Tagele Tamiru, Minale Tareke, Md Ismail Tareque, Ingan Ukur Tarigan, Mohamad-Hani Temsah, Kavumpurathu Raman Thankappan, Rekha Thapar, Ales Tichopad, Musliu Adetola Tolani, Fotis Topouzis, Marcos Roberto Tovani-Palone, Bach Xuan Tran, Jaya Prasad Tripathy, Gebiyaw Wudie Tsegaye, Nikolaos Tsilimparis, Hayley D Tymeson, Anayat Ullah, Saif Ullah, Brigid Unim, Rachel L Updike, Marco Vacante, Pascual R Valdez, Constantine Vardavas, Patricia Varona Pérez, Tommi Juhani Vasankari, Narayanaswamy Venketasubramanian, Madhur Verma, Marina V Vetrova, Bay Vo, Giang Thu Vu, Yasir Waheed, Yanzhong Wang, Kevin Welding, Andrea Werdecker, Joanna L Whisnant, Nuwan Darshana Wickramasinghe, Kazumasa Yamagishi, Srikanth Yandrapalli, Hiroshi Yatsuya, Vahid Yazdi-Feyzabadi, Yigizie Yeshaw, Mohammed Zewdu Yimmer, Naohiro Yonemoto, Chuanhua Yu, Ismaeel Yunusa, Hasan Yusefzadeh, Telma Zahirian Moghadam, Muhammed Shahriar Zaman, Maryam Zamanian, Hamed Zandian, Heather J Zar, Mikhail Sergeevich Zastrozhin, Anasthasia Zastrozhina, Luis Zavala-Arciniega, Jianrong Zhang, Zhi-Jiang Zhang, Chenwen Zhong, Yves Miel H Zuniga, Emmanuela Gakidou

## Abstract

**Background:**

Ending the global tobacco epidemic is a defining challenge in global health. Timely and comprehensive estimates of the prevalence of smoking tobacco use and attributable disease burden are needed to guide tobacco control efforts nationally and globally.

**Methods:**

We estimated the prevalence of smoking tobacco use and attributable disease burden for 204 countries and territories, by age and sex, from 1990 to 2019 as part of the Global Burden of Diseases, Injuries, and Risk Factors Study. We modelled multiple smoking-related indicators from 3625 nationally representative surveys. We completed systematic reviews and did Bayesian meta-regressions for 36 causally linked health outcomes to estimate non-linear dose-response risk curves for current and former smokers. We used a direct estimation approach to estimate attributable burden, providing more comprehensive estimates of the health effects of smoking than previously available.

**Findings:**

Globally in 2019, 1·14 billion (95% uncertainty interval 1·13–1·16) individuals were current smokers, who consumed 7·41 trillion (7·11–7·74) cigarette-equivalents of tobacco in 2019. Although prevalence of smoking had decreased significantly since 1990 among both males (27·5% [26·5–28·5] reduction) and females (37·7% [35·4–39·9] reduction) aged 15 years and older, population growth has led to a significant increase in the total number of smokers from 0·99 billion (0·98–1·00) in 1990. Globally in 2019, smoking tobacco use accounted for 7·69 million (7·16–8·20) deaths and 200 million (185–214) disability-adjusted life-years, and was the leading risk factor for death among males (20·2% [19·3–21·1] of male deaths). 6·68 million [86·9%] of 7·69 million deaths attributable to smoking tobacco use were among current smokers.

**Interpretation:**

In the absence of intervention, the annual toll of 7·69 million deaths and 200 million disability-adjusted life-years attributable to smoking will increase over the coming decades. Substantial progress in reducing the prevalence of smoking tobacco use has been observed in countries from all regions and at all stages of development, but a large implementation gap remains for tobacco control. Countries have a clear and urgent opportunity to pass strong, evidence-based policies to accelerate reductions in the prevalence of smoking and reap massive health benefits for their citizens.

**Funding:**

Bloomberg Philanthropies and the Bill & Melinda Gates Foundation.

## Introduction

Over the past 30 years, more than 200 million deaths have been caused by smoking tobacco use, and annual economic costs due to smoking tobacco use exceed US$1 trillion.^1^, ^2^ With more than 1 billion current smokers globally in 2019, these numbers are likely to increase over the coming decades. The enormous health and economic consequences of the global tobacco epidemic make tobacco control a clear and urgent public health priority.^3^ Effective implementation and enforcement of tobacco control policies and interventions can both increase healthy life expectancy and decrease health-care costs.^4^, ^5^, ^6^, ^7^ Despite the clear benefits, progress in tobacco control has varied substantially across countries.

The first international public health treaty, the WHO Framework Convention on Tobacco Control (FCTC), entered into force and became an international binding law in 2005.^8^ Consensus on the importance of tobacco control led 182 countries to ratify the treaty, which outlines a suite of recommended demand-reduction tools. These tools include reducing affordability through taxation, passing smoke-free laws, mandating health warnings on packaging, and banning tobacco advertising, promotion, and sponsorship.^9^ 15 years after the FCTC entered into force, a large implementation gap remains. WHO has monitored the implementation of the FCTC articles using the MPOWER framework for more than a decade.^10^ Over this period, only two countries, Brazil and Turkey, have implemented all the demand-reduction policies included in MPOWER at their highest level.^10^ Nonetheless, progress has been made in expanding coverage of best-practice policies, with the number of countries implementing at least one best-practice policy increasing from 43 in 2007 to 136 in 2018.^10^

**Research in context**

**Evidence before this study**

The importance of smoking tobacco use as a risk factor has resulted in a long history of estimating its prevalence and health effects using a variety of epidemiological methods. WHO produces biennial updates to estimates of prevalence of smoking tobacco use and the status of tobacco control policies around the world. The most recent global analysis of the burden of disease attributable to smoking was published on the basis of results from The Global Burden of Diseases, Injuries, and Risk Factors Study (GBD) 2015.

**Added value of this study**

This study, which is based on results from GBD 2019, updates and improves on previous estimates of the prevalence of smoking tobacco use and attributable disease burden. Compared with the GBD 2015 smoking prevalence and disease burden study, we have included more than 800 additional data sources on prevalence of smoking. In terms of methods, we developed and implemented a new unified approach to estimating the disease burden attributable to smoking tobacco use that addresses limitations of previous methods with direct estimation for all 36 causally linked health outcomes and reflecting dose-response associations among both current and former smokers. We estimated new continuous exposure distributions, including distributions of age of initiation, cigarette-equivalents of tobacco smoked per day, pack-years, and years since cessation, and new cause-specific dose-response relative risk curves among both current and former smokers. These changes improve the reliability of estimates, particularly in low-income and middle-income countries, allow for disaggregation of disease burden by intensity of exposure, and also generate a multitude of new inputs that can be used by researchers and decision makers to inform and improve modelling studies.

**Implications of all the available evidence**

Smoking remains one of the most important risk factors for premature mortality and morbidity globally. Progress in reducing the prevalence of smoking tobacco use has varied widely, as has commitment to tobacco control across countries. Of concern, progress in many countries has slowed in the past 10 years and, with population growth, the total number of global smokers continues to increase. All countries must urgently adopt and enforce a comprehensive package of evidence-based policies to reduce the prevalence of current smoking and prevent initiation, particularly among adolescents and young adults.

The global importance of non-communicable diseases has led to their inclusion at the forefront of global progress targets, including a goal of 25% reduction in premature mortality from non-communicable diseases by 2025 outlined in the WHO global non-communicable disease monitoring framework and a third reduction by 2030 included in the UN Sustainable Development Goals (SDGs).^11^, ^12^ Tobacco control has been identified as a crucial and necessary part of reaching these goals, with one in six non-communicable disease-related deaths being attributable to smoking tobacco use.^13^, ^14^, ^15^, ^16^, ^17^ As countries work towards meeting global progress targets for reducing the prevalence of smoking tobacco use and premature mortality from non-communicable diseases, timely data on the prevalence of smoking tobacco use and attributable disease are necessary to guide effective policy and planning.^11^, ^17^

The public health significance of smoking tobacco use has resulted in a long tradition of estimating patterns of smoking tobacco use and its health effects.^17^, ^18^, ^19^, ^20^, ^21^ Estimates of the attributable burden of smoking tobacco use have been included in the Global Burden of Diseases, Injuries, and Risk Factors Study (GBD) since its initial publication in 1997.^22^ Previous studies estimating the attributable burden of tobacco smoking have combined indirect estimation using the Smoking Impact Ratio method for cancers and chronic obstructive pulmonary disease, which uses observed lung cancer mortality to indirectly estimate the disease burden attributable to tobacco smoking, with direct estimation using lagged prevalence of daily smoking tobacco use for cardiovascular and circulatory diseases and all other health outcomes.^20^, ^23^, ^24^ For health outcomes modelled using daily prevalence, risks among occasional smokers and former smokers were not included, and methods did not reflect well described dose-response associations between smoking intensity and risk of disease. For the health outcomes modelled using the Smoking Impact Ratio method, reliability was low in countries with either scare or poor quality data on lung cancer mortality and in countries with other important competing risks for lung cancer, such as air pollution.

The objective of this study, which is part of GBD 2019, was to update and improve previous estimates of global trends in the prevalence of tobacco smoking and tobacco smoking-attributable disease for 204 countries and territories, by age and sex, from 1990 to 2019. Using new methods and new data, we aimed to provide novel insights into patterns of smoking intensity and their association with health outcomes that are directly relevant to guiding tobacco control efforts nationally and globally. This manuscript was produced as part of the GBD Collaborator Network and in accordance with the GBD Protocol.

## Methods

### Overview

As part of GBD 2019, we estimated the burden of disease attributable to smoking of tobacco using the comparative risk assessment framework for 204 countries and territories, by age and sex, and from 1990 to 2019. We used direct estimation methods for 36 causally linked health outcomes that show dose-response associations among both current and former smokers. Here, we summarise the key analytical steps: estimating prevalence of current and former use of smoking tobacco; modelling distributions of cigarette-equivalents of tobacco smoked per day, pack-years, and years since cessation; estimating dose-response risk curves for the 36 health outcomes; and calculating population-attributable fractions (PAFs). Full details on each analytical step are provided in appendix 1 (pp 3–28). This study adheres to the Guidelines for Accurate and Transparent Health Estimates Reporting (GATHER).^25^

### Prevalence of smoking tobacco use

We systematically identified and extracted data from 3625 nationally representative surveys, including both multinational and country-specific surveys, covering 200 of the 204 countries and territories included in the analysis. 171 (86%) of 200 countries had at least five surveys for the period 1980–2019, and 141 (71%) countries had data available from 2015 or later. Data for 1980–89 were used to inform time trends, but are not reported in the results. For countries without data, estimates were entirely based on models. We extracted data for individuals aged 10 years and older, and used data on individuals aged 10–14 years to inform model estimates; however, we report prevalence among individuals aged 15 years and older. Additional information on identification of sources, inclusion criteria, and data extraction are in appendix 1 (pp 11–12). A complete list of data sources used in our analysis is available through the Global Health Data Exchange (GHDx).

To ensure that all data included in the model were comparable, and to prevent compositional bias from affecting our estimates, we used two key data processing steps. First, we used linear regression to adjust data from surveys that only reported non-reference case definitions. Our reference case definitions were current use of any smoked tobacco product on a daily or occasional basis, and former use of any smoked tobacco product. We included all smoked tobacco products—eg, cigarettes, pipes, cigars, shisha, bidis, kreteks, and other local smoked tobacco products. We did not include smokeless tobacco, electronic cigarettes (also known as e-cigarettes), vaping products, or heated tobacco products. Risks from chewing tobacco and second-hand smoke are included as other risk factors in GBD and are outside the scope of this study.^1^, ^26^

Second, we split data reported in aggregated age groups or as both sexes combined into our standard 5-year age-sex groups. To estimate an age-sex pattern that reflects observed spatial and temporal variation, we estimated a preliminary prevalence model using only data available in our standard age-sex groups. We then used the estimated age-sex pattern, which varied by location and year, to split aggregated data, a process that also allowed us to propagate uncertainty in the age-sex pattern. These methods have been previously published,^19^, ^20^, ^27^ and full details are in appendix 1 (pp 13, 20).

We used spatiotemporal Gaussian process regression (ST-GPR) to model prevalence of both current and former smoking tobacco use (appendix 1 p 21). This modelling approach has been used extensively in GBD to estimate time-varying risk factors.^27^, ^28^ Briefly, the model was estimated in three stages. First, the level and trend were set in countries using linear regression based on covariates. Next, the first-stage estimates were adjusted by adding residuals with decaying weights across time, age, and location. Finally, the second-stage estimates were used as the prior in Gaussian process regression, which further refined the model fit and incorporated both data and model uncertainty. 1000 draws from the posterior distribution were retained and used for analysis. We report results with the 95% uncertainty interval (UI) of estimates based on the 2·5th and 97·5th percentile of draws.

### Exposure distribution

Estimates of prevalence of current and former smoking tobacco use define the full population at risk, but risk of disease varies within these groups on the basis of intensity of smoking and length of time since cessation. To incorporate these differences in risk in our estimation framework, we modelled continuous exposure distributions among both current and former smokers. Among current smokers, we estimated two distributions: cross-sectional cigarette-equivalents of tobacco per smoker per day and cumulative pack-years across their lifetime. Among former smokers, we estimated the distribution of the number of years since cessation. To account for heterogeneity in smoked tobacco products, we use a standard unit of cigarette-equivalents of tobacco. We converted non-cigarette tobacco products to cigarette-equivalents on the basis of amount of tobacco (in g), assuming 1 g of tobacco per cigarette. Estimates of cigarette-equivalents consumed per smoker per day combine two sources of information: self-reported smoking patterns from household surveys and supply-side data on country-level consumption available from the Food and Agriculture Organization of the UN (1961–2013), the US Department of Agriculture (1960–2005), and Euromonitor (2002–17). Details on the modelling process for the supply-side data and approach to integrating the two sources of information are in appendix 1 (pp 22–23).

Using estimates of cigarette-equivalents per smoker per day by location, age, and sex from 1960 to 2019, along with estimates of the distribution of initiation age, we reconstructed individual smoking histories on the basis of birth cohort smoking patterns to estimate population-level distributions of pack-years consumed. This approach is crucial to estimation of the burden of health outcomes that are linked to long-term cumulative exposure, because assumptions of constant consumption on the basis of cross-sectional patterns result in underestimation of risk at older ages and overestimation of risk at younger ages.

Where available, we extracted age of cessation or years since cessation from surveys to estimate a distribution of years since cessation among former smokers. Distributions of years since cessation, cigarette-equivalents per smoker per day, and age of initiation were based on an ensemble of underlying distribution shapes, parameterised by means (estimated using ST-GPR) and SDs (predicted from means using linear regression). An expanded description of the ensemble distribution strategy is in appendix 1 (p 24).

### Dose-response risk curves

Previous estimates of smoking-attributable burden in GBD relied on dichotomous exposures, despite well documented dose-response associations. To address this limitation, we estimated dose-response risk curves for both current and former smokers for 36 health outcomes using meta-regression (appendix 1 p 26). This process involved an extensive systematic review, covering 71 996 total search string hits, from which 902 prospective cohort and case-control studies were found to be eligible and from which data were extracted (Preferred Reporting Items for Systematic reviews and Meta-Analyses [PRISMA] diagrams for each outcome are in appendix 1 [pp 31–66]). For cancers and chronic obstructive pulmonary disease, we used pack-years as the exposure, allowing risk to reflect both duration and dose (cigarette-equivalents per day) of exposure. For cardiovascular and circulatory diseases and all other health outcomes, we used cigarette-equivalents per smoker per day as the exposure among current smokers, because dose is generally thought to be more important than duration of exposure for these health outcomes. We used the Disease Modelling Ordinary Differential Equation (DisMod ODE) solver to fit non-linear Bayesian meta-regressions for each health outcome.^29^, ^30^ Due to a paucity of data on relative risks among individuals younger than 30 years, we attributed outcomes to individuals aged 30 years and older, an approach consistent with previous GBD studies.

Among former smokers, risk decreases with an increasing number of years since cessation, but the level of risk also depends on previous smoking history. To control for differences in smoking history across the populations included in the meta-regressions, we adjusted reported relative risk estimates to standardise the risk at the time of cessation (appendix 1 pp 26–28).^30^

### Population attributable fractions

Inputs to estimation of PAFs included prevalence of current and former smoking tobacco use, continuous exposure distributions, relative risks, and the theoretical minimum risk exposure level. For smoking tobacco use, the theoretical minimum risk exposure level is never smoking. The PAF equation is specified in appendix 1 (p 28). Because the risk reduction curves for former smokers must account for two dimensions—years since cessation and intensity of smoking before cessation—we scaled the risk reduction curves for former smokers to match their starting relative risk (when years since cessation equals zero) to the exposure-weighted relative risk among current smokers in that population. We combined global dose-response risk curves with country-year-age-sex-specific continuous exposure distributions so we could capture differences in risk across countries that result from heterogeneous smoking patterns. Exposures were lagged on the basis of the average length of follow-up across studies included in the meta-regressions (appendix 1 p 28). We calculated attributable burden by multiplying PAFs with cause-specific deaths by location, year, age, and sex, available from GBD 2019.^31^ Using the distributive property of PAFs, we also partitioned out smoking-attributable deaths by exposure categories.^32^

Using our PAF estimates, we calculated the number of smoking attributable deaths, disability-adjusted life-years (DALYs), years of life lost (YLLs), and years lived with disability (YLDs). We further calculated the ratio of YLLs to YLDs by country and examined associations of this ratio with Socio-demographic Index level. For analyses by country income level, we used World Bank income groups.

We did all analyses using R (versions 3.1–3.6) and Python (version 2.7).

### Role of the funding source

The funders of the study had no role in study design, data collection, data analysis, data interpretation, or writing of the report.

## Results

Globally, there were 1·14 billion (95% UI 1·13–1·16) current smokers in 2019. Age-standardised prevalence of current use of smoking tobacco among individuals aged 15 years and older was 32·7% (32·3–33·0) among males and 6·62% (6·43–6·83) among females. Age-standardised prevalence among males aged 15 years and older ranged from 7·33% (6·56–8·20) in Peru to 64·6% (62·7–66·6) in Timor-Leste, and among females from 0·696% (0·517–0·906) in Eritrea to 42·3% (36·5–48·4) in Greenland (table). Smoking prevalence exceeded 20% among males in 151 countries and among females in 42 countries (table). Among individuals aged 15 years and older, countries with the highest prevalence of smoking tobacco use among males were mostly in Asia and Oceania (appendix 2 p 83), whereas countries with the highest prevalence of smoking tobacco use among females were mostly in Europe and Oceania (appendix 2 p 84). Among the 159 countries with a population exceeding 1 million, the highest prevalence of smoking tobacco use in males aged 15 years and older was observed in Timor-Leste, Indonesia, Armenia, Jordan, and Georgia, and the highest prevalence of smoking tobacco use among females aged 15 years and older was observed in Serbia, Chile, Croatia, Bulgaria, and Greece (table). The ten countries with the largest number of tobacco smokers in 2019, together comprising nearly two-thirds of the global tobacco smoking population, were China, India, Indonesia, the USA, Russia, Bangladesh, Japan, Turkey, Vietnam, and the Philippines (appendix 2 pp 85–93); 341 million (30%) of 1·14 billion tobacco smokers globally lived in China in 2019.

**Table:** Age-standardised prevalence of current smoking tobacco use in 2019 and percentage change in age-standardised prevalence of current smoking tobacco use in 1990–2019 among individuals aged 15 years and older, by location and sex

		**Age-standardised prevalence, 2019**		**Percentage change 1990–2019**	
		Females	Males	Females	Males
**Global**		**6·62 (6·43 to 6·83)**	**32·7 (32·3 to 33·0)**	**−37·7 (−39·9 to −35·4)**	**−27·5 (−28·5 to −26·5)**
**Central Europe, eastern Europe, and central Asia**		**15·5 (14·9 to 16·2)**	**39·5 (38·9 to 40·0)**	**−4·37 (−10·2 to 2·00)**	**−21·6 (−23·2 to −20·0)**
Central Asia		3·79 (3·45 to 4·18)	34·3 (33·5 to 35·1)	−5·97 (−19·7 to 7·78)	−12·3 (−15·1 to −9·61)
	Armenia	3·10 (2·48 to 3·79)	55·3 (53·6 to 57·0)	−5·19 (−33·9 to 35·0)	−8·42 (−13·1 to −3·89)
	Azerbaijan	2·50 (1·86 to 3·30)	43·6 (41·9 to 45·2)	30·8 (−13·7 to 91·7)	−1·55 (−8·50 to 5·78)
	Georgia	7·03 (5·97 to 8·15)	51·8 (50·0 to 53·6)	11·3 (−16·7 to 45·2)	5·80 (−0·933 to 13·6)
	Kazakhstan	7·69 (6·46 to 9·16)	43·2 (41·3 to 45·0)	2·88 (−25·3 to 33·1)	−14·9 (−19·9 to −10·0)
	Kyrgyzstan	4·65 (3·77 to 5·63)	43·6 (41·7 to 45·5)	49·0 (7·52 to 105)	−5·39 (−10·5 to −0·251)
	Mongolia	8·26 (6·86 to 9·84)	51·7 (49·6 to 53·9)	44·4 (7·67 to 88·9)	11·0 (4·24 to 18·5)
	Tajikistan	1·29 (0·982 to 1·68)	16·9 (15·7 to 18·3)	−41·7 (−61·7 to −15·0)	−45·8 (−51·7 to −39·4)
	Turkmenistan	3·05 (2·34 to 3·91)	21·6 (19·8 to 23·4)	−37·4 (−58·3 to −9·91)	−46·5 (−52·2 to −40·4)
	Uzbekistan	1·94 (1·48 to 2·48)	24·8 (23·1 to 26·6)	47·9 (−0·335 to 117)	36·3 (19·6 to 53·5)
Central Europe		25·9 (25·0 to 26·7)	35·8 (35·1 to 36·4)	−8·47 (−13·0 to −3·96)	−24·7 (−26·5 to −22·8)
	Albania	11·9 (9·87 to 14·2)	51·5 (49·7 to 53·1)	85·0 (35·1 to 145)	25·4 (16·5 to 34·3)
	Bosnia and Herzegovina	30·5 (26·8 to 34·2)	45·1 (42·6 to 47·5)	41·1 (16·1 to 70·3)	17·5 (7·49 to 27·7)
	Bulgaria	32·5 (29·5 to 35·4)	42·5 (40·4 to 44·6)	−16·2 (−29·5 to −0·400)	−20·4 (−26·0 to −14·9)
	Croatia	32·6 (29·9 to 35·5)	39·1 (37·2 to 41·0)	−11·7 (−23·5 to 0·755)	−17·6 (−23·3 to −11·8)
	Czech Republic	23·2 (21·3 to 25·2)	34·1 (32·5 to 35·6)	−20·2 (−29·1 to −9·61)	−21·7 (−27·5 to −15·9)
	Hungary	26·5 (23·8 to 29·2)	34·2 (32·0 to 36·1)	−18·4 (−29·4 to −6·32)	−21·7 (−27·2 to −15·6)
	Montenegro	32·7 (30·3 to 35·2)	37·5 (35·7 to 39·3)	0·505 (−15·8 to 20·0)	−8·35 (−16·0 to −0·294)
	North Macedonia	31·0 (28·3 to 33·9)	47·1 (45·0 to 49·2)	4·38 (−14·7 to 25·1)	−2·24 (−9·31 to 5·48)
	Poland	24·4 (22·5 to 26·3)	31·8 (30·4 to 33·3)	−17·8 (−25·8 to −9·17)	−40·5 (−43·6 to −37·2)
	Romania	23·5 (21·7 to 25·5)	38·4 (36·8 to 40·0)	8·91 (−5·85 to 25·1)	−18·8 (−24·7 to −12·8)
	Serbia	37·8 (34·5 to 41·1)	38·8 (36·9 to 40·8)	20·3 (2·25 to 39·8)	−6·30 (−13·4 to 1·34)
	Slovakia	20·9 (18·7 to 23·3)	33·3 (31·2 to 35·3)	−14·9 (−31·4 to 4·94)	−23·3 (−30·0 to −16·2)
	Slovenia	24·9 (22·6 to 27·4)	29·8 (28·0 to 31·5)	9·05 (−11·3 to 30·7)	−11·0 (−19·1 to −1·45)
Eastern Europe		15·6 (14·4 to 17·0)	44·7 (43·7 to 45·8)	15·9 (2·24 to 31·3)	−19·4 (−22·0 to −16·8)
	Belarus	23·6 (20·7 to 26·6)	50·2 (47·9 to 52·5)	31·5 (0·570 to 69·1)	−5·15 (−11·8 to 1·42)
	Estonia	18·9 (17·2 to 20·7)	31·7 (30·2 to 33·1)	−12·9 (−24·8 to −1·42)	−28·3 (−32·5 to −23·9)
	Latvia	21·3 (19·3 to 23·4)	46·9 (45·2 to 48·7)	−3·33 (−18·8 to 15·5)	−12·9 (−17·4 to −7·98)
	Lithuania	20·3 (18·4 to 22·6)	37·9 (36·1 to 39·8)	28·9 (10·4 to 50·4)	−18·5 (−23·9 to −12·9)
	Moldova	7·20 (5·93 to 8·68)	40·6 (38·6 to 42·7)	11·7 (−17·2 to 47·7)	3·88 (−4·24 to 13·0)
	Russia	15·5 (13·9 to 17·3)	45·6 (44·1 to 47·0)	26·1 (5·67 to 49·7)	−17·6 (−21·1 to −13·9)
	Ukraine	14·4 (12·7 to 16·3)	42·0 (40·3 to 43·6)	−7·85 (−26·0 to 12·4)	−28·3 (−32·1 to −24·4)
**High income**		**17·6 (17·1 to 18·2)**	**26·9 (26·4 to 27·3)**	**−28·8 (−31·2 to −26·2)**	**−32·2 (−33·4 to −31·0)**
Australasia		14·5 (12·7 to 16·5)	16·7 (15·6 to 17·9)	−46·5 (−53·1 to −39·0)	−46·2 (−50·0 to −42·3)
	Australia	14·4 (12·2 to 16·7)	16·4 (15·1 to 17·8)	−46·8 (−54·9 to −38·0)	−48·1 (−52·4 to −43·7)
	New Zealand	15·2 (13·9 to 16·5)	18·4 (17·5 to 19·4)	−44·8 (−49·8 to −39·5)	−35·6 (−39·5 to −31·4)
High-income Asia Pacific		8·48 (7·48 to 9·64)	35·9 (34·4 to 37·3)	−25·6 (−35·7 to −14·7)	−36·9 (−39·7 to −34·0)
	Brunei	5·39 (4·28 to 6·78)	28·5 (26·1 to 31·2)	−31·3 (−50·9 to −7·75)	−33·3 (−40·4 to −25·8)
	Japan	10·2 (8·71 to 11·9)	33·4 (31·4 to 35·5)	−23·6 (−35·2 to −9·65)	−41·7 (−45·3 to −38·0)
	Singapore	6·82 (5·38 to 8·41)	20·6 (18·9 to 22·4)	−32·6 (−52·1 to −8·90)	−31·3 (−39·6 to −22·5)
	South Korea	5·21 (4·14 to 6·58)	42·5 (40·1 to 44·7)	−18·4 (−42·8 to 11·5)	−25·2 (−30·4 to −19·5)
High-income North America		15·3 (14·1 to 16·7)	19·7 (18·8 to 20·7)	−39·9 (−44·8 to −34·5)	−31·8 (−35·3 to −28·2)
	Canada	15·9 (13·4 to 18·6)	18·3 (16·8 to 19·8)	−48·2 (−56·5 to −38·9)	−46·8 (−51·7 to −42·0)
	Greenland	42·3 (36·5 to 48·4)	44·1 (40·9 to 47·3)	−5·32 (−21·0 to 13·6)	−10·2 (−18·0 to −2·05)
	USA	15·3 (13·9 to 16·7)	19·9 (18·8 to 21·0)	−38·7 (−44·2 to −32·7)	−29·8 (−33·7 to −25·7)
Southern Latin America		23·3 (21·2 to 25·3)	31·3 (29·9 to 32·7)	−20·0 (−31·2 to −8·02)	−17·2 (−22·8 to −11·3)
	Argentina	19·6 (17·3 to 22·2)	29·2 (27·4 to 31·0)	−22·6 (−37·9 to −4·07)	−17·5 (−25·7 to −7·94)
	Chile	32·7 (28·7 to 36·6)	36·2 (33·9 to 38·4)	−12·7 (−27·0 to 4·89)	−13·0 (−20·6 to −4·91)
	Uruguay	23·1 (19·4 to 26·7)	31·0 (28·8 to 33·3)	−18·1 (−33·7 to −0·553)	−23·0 (−30·0 to −14·8)
Western Europe		22·7 (22·0 to 23·4)	28·8 (28·2 to 29·3)	−24·2 (−26·7 to −21·8)	−28·8 (−30·2 to −27·4)
	Andorra	24·4 (19·6 to 29·5)	31·4 (28·6 to 34·2)	−19·8 (−37·9 to 1·56)	−25·2 (−32·8 to −17·2)
	Austria	26·1 (23·4 to 29·0)	36·1 (34·0 to 38·3)	−10·5 (−20·2 to 0·384)	−14·2 (−19·9 to −8·34)
	Belgium	21·4 (19·6 to 23·3)	24·9 (23·6 to 26·2)	−28·5 (−35·6 to −21·1)	−39·3 (−43·1 to −35·6)
	Cyprus	21·3 (18·7 to 24·3)	44·8 (42·6 to 47·1)	−11·6 (−30·8 to 9·57)	−5·43 (−12·5 to 2·85)
	Denmark	22·6 (20·6 to 24·8)	22·7 (21·3 to 24·1)	−49·5 (−54·0 to −44·4)	−49·2 (−52·3 to −45·7)
	Finland	18·1 (16·3 to 20·3)	24·8 (23·3 to 26·2)	−31·2 (−39·3 to −21·5)	−27·9 (−32·3 to −23·2)
	France	31·3 (28·8 to 33·9)	36·9 (35·1 to 38·6)	−1·18 (−9·35 to 7·54)	−17·2 (−21·4 to −12·9)
	Germany	23·0 (21·1 to 24·9)	29·9 (28·4 to 31·4)	−18·0 (−25·2 to −10·3)	−20·7 (−25·2 to −16·2)
	Greece	32·2 (29·5 to 35·1)	44·1 (42·3 to 46·1)	−0·620 (−9·99 to 8·67)	−18·4 (−22·2 to −14·5)
	Iceland	15·1 (13·1 to 17·5)	15·8 (14·7 to 17·1)	−47·7 (−56·6 to −37·8)	−51·4 (−55·8 to −46·3)
	Ireland	22·8 (20·4 to 25·3)	21·9 (20·2 to 23·6)	−27·8 (−35·7 to −19·6)	−38·7 (−43·8 to −33·7)
	Israel	14·9 (12·8 to 17·2)	26·4 (24·6 to 28·3)	−39·8 (−51·2 to −27·4)	−33·8 (−39·7 to −27·9)
	Italy	18·9 (17·3 to 20·6)	27·3 (25·8 to 28·8)	−28·1 (−34·5 to −21·2)	−30·5 (−34·6 to −26·3)
	Luxembourg	21·3 (19·3 to 23·3)	26·2 (24·5 to 28·0)	−27·9 (−36·3 to −19·1)	−31·6 (−36·8 to −25·4)
	Malta	22·0 (19·2 to 24·9)	26·9 (24·9 to 28·8)	−15·0 (−30·9 to 2·21)	−36·0 (−41·9 to −29·5)
	Monaco	23·6 (18·9 to 28·6)	29·0 (26·4 to 31·7)	−19·5 (−38·7 to 2·21)	−24·9 (−32·9 to −15·7)
	Netherlands	20·4 (18·6 to 22·2)	25·0 (23·6 to 26·4)	−46·8 (−51·7 to −41·5)	−42·4 (−46·0 to −38·9)
	Norway	16·5 (14·3 to 18·9)	20·2 (18·7 to 22·0)	−57·6 (−63·9 to −50·4)	−49·8 (−54·4 to −44·9)
	Portugal	22·4 (20·2 to 24·7)	33·4 (31·7 to 35·0)	30·6 (16·4 to 45·4)	−18·7 (−23·1 to −14·2)
	San Marino	18·1 (15·2 to 21·5)	21·6 (19·7 to 23·4)	−36·0 (−50·7 to −18·3)	−40·5 (−47·3 to −33·4)
	Spain	24·6 (22·6 to 26·8)	30·7 (29·1 to 32·5)	−23·6 (−30·2 to −16·3)	−39·6 (−42·9 to −36·0)
	Sweden	14·4 (12·6 to 16·5)	12·4 (11·3 to 13·5)	−47·1 (−54·5 to −38·7)	−44·6 (−50·0 to −38·8)
	Switzerland	23·4 (20·7 to 26·3)	29·3 (27·2 to 31·2)	−18·9 (−28·6 to −8·35)	−23·5 (−28·6 to −18·4)
	UK	18·1 (16·3 to 19·9)	21·7 (20·4 to 22·9)	−42·5 (−48·4 to −36·6)	−35·7 (−39·7 to −32·0)
**Latin America and Caribbean**		**7·64 (7·16 to 8·10)**	**17·1 (16·6 to 17·5)**	**−59·6 (−62·5 to −56·5)**	**−52·3 (−53·9 to −50·6)**
Andean Latin America		4·84 (4·25 to 5·48)	14·0 (13·3 to 14·6)	−17·4 (−33·4 to 0·994)	−16·9 (−23·1 to −10·5)
	Bolivia	7·31 (5·94 to 8·85)	18·2 (16·8 to 19·7)	−0·326 (−29·1 to 36·6)	−7·58 (−18·9 to 5·09)
	Ecuador	4·97 (4·14 to 5·98)	24·7 (23·5 to 25·9)	−18·1 (−38·7 to 7·54)	−8·37 (−17·2 to 0·777)
	Peru	4·02 (3·22 to 4·95)	7·33 (6·56 to 8·20)	−23·2 (−47·1 to 9·24)	−35·4 (−45·4 to −24·6)
Caribbean		8·65 (7·76 to 9·55)	19·6 (18·9 to 20·4)	−37·1 (−45·9 to −26·9)	−30·9 (−34·6 to −27·0)
	Antigua and Barbuda	5·56 (4·29 to 6·97)	12·5 (11·1 to 14·1)	31·5 (−6·42 to 81·9)	31·4 (11·8 to 52·7)
	The Bahamas	3·18 (2·51 to 4·04)	11·4 (10·4 to 12·6)	−11·4 (−38·4 to 27·1)	−0·304 (−13·6 to 15·5)
	Barbados	4·61 (3·60 to 5·88)	13·9 (12·6 to 15·5)	−3·25 (−34·2 to 38·0)	−15·4 (−26·6 to −2·65)
	Belize	4·13 (3·18 to 5·24)	22·5 (20·4 to 24·6)	9·24 (−25·9 to 50·3)	1·95 (−11·1 to 15·7)
	Bermuda	8·32 (6·80 to 10·2)	18·1 (16·5 to 19·9)	−2·45 (−28·8 to 33·4)	−0·776 (−13·6 to 14·7)
	Cuba	15·3 (12·7 to 17·9)	31·8 (29·4 to 34·4)	−34·2 (−49·2 to −15·1)	−25·9 (−32·8 to −18·2)
	Dominica	6·29 (4·82 to 8·04)	15·1 (13·4 to 16·8)	−6·88 (−35·3 to 27·5)	−2·73 (−17·0 to 13·5)
	Dominican Republic	9·27 (7·70 to 10·9)	13·7 (12·6 to 14·9)	−21·0 (−39·6 to 1·18)	−7·15 (−18·0 to 5·97)
	Grenada	5·39 (4·16 to 6·85)	17·7 (15·8 to 19·6)	2·67 (−30·5 to 44·3)	7·33 (−7·82 to 23·7)
	Guyana	4·20 (3·32 to 5·19)	24·9 (23·3 to 26·8)	−9·62 (−36·7 to 22·6)	6·45 (−6·41 to 20·9)
	Haiti	2·65 (2·05 to 3·36)	10·1 (9·22 to 11·0)	−48·6 (−65·2 to −27·5)	−45·8 (−52·6 to −38·4)
	Jamaica	6·82 (5·73 to 8·09)	20·1 (18·5 to 21·8)	−21·8 (−40·1 to 0·233)	−26·5 (−34·8 to −17·5)
	Puerto Rico	8·57 (7·42 to 9·87)	16·0 (14·9 to 17·2)	−14·2 (−31·8 to 6·44)	−18·3 (−26·4 to −8·88)
	Saint Kitts and Nevis	2·87 (2·16 to 3·75)	10·5 (9·30 to 11·8)	−0·0613 (−32·4 to 41·3)	1·56 (−15·0 to 20·1)
	Saint Lucia	5·27 (4·17 to 6·49)	19·2 (17·4 to 21·1)	−14·2 (−39·3 to 18·3)	−13·3 (−24·0 to −0·879)
	Saint Vincent and the Grenadines	4·24 (3·22 to 5·53)	20·3 (18·5 to 22·1)	2·04 (−28·9 to 41·4)	−4·93 (−15·8 to 7·45)
	Suriname	8·85 (7·07 to 11·0)	34·2 (31·7 to 36·6)	−25·5 (−46·9 to 0·950)	−17·7 (−25·5 to −8·96)
	Trinidad and Tobago	8·17 (6·44 to 10·1)	28·9 (26·6 to 31·2)	−7·88 (−34·0 to 26·3)	−12·7 (−22·4 to −2·34)
	Virgin Islands	4·98 (3·98 to 6·18)	8·28 (7·38 to 9·22)	−24·3 (−46·3 to 2·89)	−23·3 (−34·3 to −9·97)
Central Latin America		8·72 (8·04 to 9·48)	22·7 (22·0 to 23·4)	−42·2 (−49·0 to −34·4)	−40·1 (−42·6 to −37·5)
	Colombia	9·71 (8·42 to 11·2)	14·7 (13·6 to 15·9)	−33·3 (−49·3 to −13·6)	−53·6 (−58·7 to −48·1)
	Costa Rica	7·19 (5·93 to 8·67)	15·3 (14·1 to 16·6)	−48·2 (−60·7 to −32·1)	−45·9 (−52·2 to −38·8)
	El Salvador	3·70 (2·91 to 4·63)	17·3 (15·8 to 18·9)	36·9 (−5·05 to 87·8)	34·3 (15·4 to 55·2)
	Guatemala	4·34 (3·43 to 5·41)	20·5 (19·0 to 22·0)	−5·00 (−32·1 to 29·7)	−14·6 (−24·8 to −4·31)
	Honduras	5·25 (4·15 to 6·61)	24·0 (22·2 to 25·9)	−11·8 (−35·3 to 16·4)	−19·9 (−28·4 to −10·5)
	Mexico	9·01 (7·79 to 10·3)	27·0 (25·7 to 28·2)	−48·1 (−57·1 to −38·3)	−40·6 (−44·3 to −36·8)
	Nicaragua	5·46 (4·15 to 7·01)	21·5 (19·5 to 23·7)	−16·2 (−42·4 to 17·4)	−23·3 (−33·2 to −12·7)
	Panama	4·57 (3·58 to 5·71)	12·4 (11·3 to 13·7)	−35·9 (−54·5 to −10·6)	−39·1 (−46·3 to −31·1)
	Venezuela	11·7 (9·37 to 14·5)	22·8 (20·5 to 25·2)	−32·2 (−49·8 to −11·0)	−35·3 (−42·6 to −27·0)
Tropical Latin America		6·90 (6·06 to 7·74)	11·2 (10·6 to 11·9)	−74·3 (−77·6 to −70·8)	−71·5 (−73·7 to −69·2)
	Brazil	6·86 (6·02 to 7·73)	10·9 (10·1 to 11·6)	−74·7 (−78·0 to −71·2)	−72·5 (−74·7 to −70·1)
	Paraguay	8·33 (6·79 to 10·1)	24·6 (22·7 to 26·7)	−41·9 (−56·4 to −24·7)	−39·7 (−46·3 to −33·4)
**North Africa and Middle East**		**5·63 (5·31 to 5·99)**	**32·4 (31·9 to 32·9)**	**−2·88 (−12·7 to 7·23)**	**−11·2 (−13·6 to −8·75)**
Afghanistan		2·67 (2·10 to 3·39)	17·0 (15·7 to 18·4)	179 (93·4 to 285)	205 (158 to 254)
Algeria		1·74 (1·34 to 2·24)	32·7 (30·6 to 34·8)	−28·7 (−51·5 to −0·699)	−7·01 (−16·2 to 2·31)
Bahrain		5·10 (4·13 to 6·33)	23·4 (21·4 to 25·5)	−20·7 (−40·4 to 2·68)	−1·03 (−11·1 to 10·0)
Egypt		1·06 (0·803 to 1·37)	43·4 (42·2 to 44·7)	2·41 (−31·7 to 50·5)	13·1 (4·88 to 21·9)
Iran		4·73 (3·84 to 5·75)	24·9 (23·2 to 26·5)	8·28 (−26·7 to 47·9)	2·30 (−8·74 to 14·6)
Iraq		3·53 (2·81 to 4·38)	37·6 (35·4 to 39·6)	−22·1 (−45·0 to 6·51)	−18·8 (−25·2 to −11·5)
Jordan		11·8 (10·1 to 13·8)	53·0 (51·5 to 54·5)	10·6 (−15·8 to 44·6)	11·4 (4·64 to 18·3)
Kuwait		4·72 (3·78 to 5·76)	33·7 (31·5 to 35·8)	−8·57 (−36·3 to 24·3)	−14·2 (−21·6 to −6·18)
Lebanon		26·0 (22·8 to 29·0)	46·1 (44·0 to 48·2)	45·1 (15·8 to 80·2)	25·1 (14·4 to 36·5)
Libya		1·46 (1·10 to 1·94)	38·6 (36·3 to 41·0)	−2·10 (−34·2 to 38·4)	0·602 (−8·37 to 10·7)
Morocco		1·06 (0·788 to 1·42)	22·5 (20·9 to 24·1)	−31·3 (−54·3 to 1·02)	−33·2 (−39·3 to −26·1)
Oman		1·88 (1·45 to 2·43)	15·8 (14·5 to 17·3)	−11·0 (−37·4 to 23·1)	−24·6 (−32·4 to −16·5)
Palestine		3·89 (3·07 to 4·84)	40·8 (38·6 to 43·2)	−3·23 (−29·4 to 28·9)	−8·48 (−14·7 to −2·05)
Qatar		2·97 (2·24 to 3·94)	21·9 (20·3 to 23·6)	4·14 (−32·9 to 54·7)	−4·13 (−15·4 to 8·50)
Saudi Arabia		2·17 (1·69 to 2·71)	22·6 (21·1 to 24·0)	45·3 (0·241 to 99·6)	39·0 (26·2 to 53·6)
Sudan		1·95 (1·46 to 2·54)	19·5 (18·0 to 21·1)	−15·2 (−42·5 to 23·4)	−12·3 (−22·9 to −0·539)
Syria		6·20 (4·77 to 7·97)	41·9 (39·0 to 44·8)	−23·3 (−47·1 to 6·07)	−16·5 (−23·3 to −9·84)
Tunisia		2·70 (2·12 to 3·45)	45·4 (43·2 to 47·7)	−19·9 (−43·5 to 10·8)	−11·1 (−17·0 to −4·99)
Turkey		18·4 (16·6 to 20·3)	43·2 (41·6 to 44·9)	14·6 (−3·48 to 33·5)	−21·8 (−26·3 to −17·2)
United Arab Emirates		3·79 (2·96 to 4·81)	17·9 (16·5 to 19·3)	2·58 (−27·0 to 44·1)	−21·0 (−30·5 to −10·8)
Yemen		8·90 (7·46 to 10·7)	31·5 (29·5 to 33·6)	4·41 (−24·0 to 40·2)	−4·64 (−14·1 to 5·27)
**South Asia**		**3·26 (2·83 to 3·78)**	**25·2 (24·2 to 26·2)**	**−34·1 (−45·8 to −21·1)**	**−37·8 (−40·4 to −35·1)**
Bangladesh		2·13 (1·68 to 2·66)	44·9 (43·0 to 47·0)	−27·9 (−47·7 to −4·36)	−11·4 (−16·7 to −6·25)
Bhutan		4·80 (3·79 to 5·95)	14·8 (13·4 to 16·2)	−4·62 (−33·8 to 31·6)	−12·9 (−24·1 to −0·228)
India		3·10 (2·59 to 3·74)	23·0 (21·9 to 24·2)	−31·2 (−46·6 to −12·9)	−41·0 (−44·2 to −37·6)
Nepal		13·6 (11·9 to 15·6)	31·4 (29·8 to 33·0)	−54·0 (−61·9 to −44·6)	−37·0 (−41·4 to −32·6)
Pakistan		3·77 (3·05 to 4·65)	24·7 (23·3 to 26·3)	−40·7 (−58·0 to −18·0)	−39·2 (−44·3 to −33·6)
**Southeast Asia, east Asia, and Oceania**		**3·94 (3·51 to 4·39)**	**49·4 (48·4 to 50·4)**	**−22·1 (−33·2 to −11·0)**	**−16·6 (−18·6 to −14·5)**
East Asia		3·57 (2·97 to 4·18)	49·5 (48·0 to 50·8)	−20·7 (−36·4 to −3·84)	−18·1 (−20·8 to −15·4)
	China	3·54 (2·91 to 4·18)	49·7 (48·3 to 51·1)	−20·9 (−37·4 to −3·53)	−18·2 (−21·0 to −15·5)
	North Korea	4·48 (3·57 to 5·69)	43·6 (41·3 to 45·6)	11·6 (−20·7 to 51·2)	−8·47 (−15·3 to −0·989)
	Taiwan (Province of China)	4·98 (3·94 to 6·21)	39·6 (37·5 to 41·7)	−27·9 (−51·0 to −0·968)	−18·2 (−24·3 to −11·5)
Oceania		18·0 (16·0 to 20·2)	41·3 (39·4 to 43·2)	−17·7 (−32·1 to −1·23)	−14·9 (−19·7 to −9·54)
	American Samoa	22·4 (18·3 to 27·2)	42·1 (39·2 to 45·2)	−4·19 (−27·7 to 25·2)	−4·14 (−12·7 to 5·48)
	Cook Islands	24·2 (20·5 to 28·6)	36·7 (34·4 to 39·1)	−0·774 (−21·8 to 25·9)	−9·81 (−18·7 to −0·276)
	Federated States of Micronesia	36·4 (31·0 to 41·5)	62·2 (59·7 to 64·8)	12·4 (−9·52 to 35·5)	3·73 (−2·38 to 9·83)
	Fiji	14·5 (12·0 to 17·3)	42·5 (40·0 to 45·4)	−18·9 (−38·4 to 6·27)	−14·8 (−21·6 to −7·97)
	Guam	20·1 (17·8 to 22·3)	30·3 (28·6 to 32·1)	−8·28 (−26·4 to 12·2)	−14·6 (−22·4 to −6·08)
	Kiribati	35·1 (31·5 to 38·6)	63·8 (61·7 to 65·9)	0·681 (−16·7 to 21·3)	6·98 (0·976 to 13·2)
	Marshall Islands	9·75 (7·74 to 12·0)	35·0 (32·3 to 37·5)	27·1 (−9·19 to 75·7)	3·09 (−7·84 to 15·3)
	Nauru	40·3 (36·3 to 44·6)	43·3 (40·9 to 45·8)	−7·17 (−22·1 to 9·84)	−4·09 (−11·8 to 4·43)
	Niue	15·8 (13·1 to 18·8)	27·0 (25·0 to 29·1)	−1·31 (−26·2 to 32·3)	−7·53 (−17·6 to 3·99)
	Northern Mariana Islands	18·0 (14·3 to 22·1)	41·0 (38·0 to 43·9)	−17·7 (−37·6 to 8·84)	−13·1 (−21·1 to −4·45)
	Palau	12·6 (10·9 to 14·5)	32·4 (30·6 to 34·3)	−1·96 (−25·5 to 25·2)	−6·71 (−15·4 to 3·12)
	Papua New Guinea	18·4 (15·8 to 21·3)	40·2 (37·7 to 42·6)	−21·2 (−38·7 to 0·844)	−17·3 (−23·9 to −10·2)
	Samoa	14·5 (12·5 to 16·6)	39·4 (37·2 to 41·6)	−11·6 (−32·5 to 13·1)	−11·4 (−18·4 to −4·35)
	Solomon Islands	19·3 (17·0 to 21·8)	52·2 (49·9 to 54·2)	7·00 (−16·4 to 36·0)	3·90 (−2·92 to 11·7)
	Tokelau	16·6 (13·0 to 20·6)	39·6 (36·4 to 42·5)	0·116 (−26·1 to 31·5)	−5·30 (−14·4 to 5·05)
	Tonga	13·3 (11·3 to 15·4)	42·5 (40·2 to 44·8)	−1·21 (−20·6 to 22·1)	−22·3 (−27·0 to −17·2)
	Tuvalu	20·7 (17·3 to 24·4)	45·4 (42·6 to 48·0)	9·73 (−17·7 to 43·3)	3·49 (−4·63 to 13·0)
	Vanuatu	5·70 (4·54 to 6·95)	38·2 (36·0 to 40·3)	−17·8 (−43·4 to 14·2)	−11·8 (−19·4 to −3·90)
Southeast Asia		4·51 (4·18 to 4·87)	48·2 (47·5 to 48·9)	−29·9 (−37·0 to −22·0)	−12·6 (−14·4 to −10·7)
	Cambodia	5·09 (4·17 to 6·10)	40·6 (38·8 to 42·3)	−13·9 (−34·6 to 10·6)	−19·7 (−24·3 to −14·7)
	Indonesia	3·60 (3·00 to 4·31)	58·3 (57·0 to 59·6)	7·99 (−17·2 to 39·2)	6·94 (3·14 to 10·6)
	Laos	6·98 (5·73 to 8·29)	49·1 (46·8 to 51·2)	29·3 (−6·25 to 71·2)	9·06 (1·05 to 17·2)
	Malaysia	3·20 (2·57 to 3·92)	40·3 (38·1 to 42·3)	−26·0 (−47·5 to 1·04)	−22·3 (−28·1 to −16·0)
	Maldives	7·35 (5·88 to 9·03)	46·5 (43·8 to 48·9)	−6·48 (−32·8 to 23·8)	−6·02 (−13·3 to 0·792)
	Mauritius	5·40 (4·36 to 6·60)	41·2 (39·2 to 43·3)	−7·65 (−32·1 to 23·3)	−13·0 (−19·2 to −6·93)
	Myanmar	8·59 (7·24 to 10·0)	41·0 (39·2 to 42·8)	−48·7 (−59·6 to −35·3)	−32·2 (−36·6 to −27·8)
	Philippines	8·24 (7·24 to 9·31)	40·9 (39·3 to 42·5)	−37·7 (−50·7 to −22·6)	−28·9 (−32·9 to −24·6)
	Seychelles	7·01 (5·58 to 8·62)	39·7 (36·9 to 42·4)	−5·93 (−34·0 to 30·1)	−10·9 (−19·7 to −1·99)
	Sri Lanka	1·80 (1·39 to 2·26)	30·1 (28·5 to 31·8)	−40·7 (−58·4 to −17·4)	−30·8 (−36·0 to −24·9)
	Thailand	3·49 (2·89 to 4·19)	39·8 (38·5 to 41·3)	−46·5 (−59·9 to −31·5)	−25·5 (−29·4 to −21·5)
	Timor-Leste	5·15 (4·18 to 6·21)	64·6 (62·7 to 66·6)	−4·15 (−30·1 to 26·9)	1·49 (−3·55 to 6·71)
	Vietnam	2·68 (2·13 to 3·30)	47·1 (45·3 to 48·8)	−32·9 (−50·2 to −13·4)	−20·3 (−24·2 to −16·3)
**Sub-Saharan Africa**		**2·94 (2·78 to 3·10)**	**17·5 (17·2 to 17·9)**	**−34·1 (−39·1 to −28·9)**	**−22·8 (−24·7 to −20·6)**
Central sub-Saharan Africa		1·74 (1·46 to 2·06)	20·7 (19·6 to 22·0)	−4·72 (−26·1 to 18·9)	−6·47 (−14·6 to 1·85)
	Angola	2·74 (2·14 to 3·51)	18·7 (17·2 to 20·2)	−9·87 (−36·6 to 23·5)	−13·2 (−23·3 to −1·61)
	Central African Republic	1·60 (1·22 to 2·12)	15·0 (13·3 to 16·8)	−26·1 (−50·2 to 4·37)	−25·8 (−36·0 to −15·1)
	Congo (Brazzaville)	1·81 (1·33 to 2·42)	20·7 (19·0 to 22·6)	20·3 (−24·1 to 75·6)	28·8 (13·7 to 47·7)
	Democratic Republic of the Congo	1·36 (1·01 to 1·79)	21·6 (20·0 to 23·4)	−6·49 (−38·2 to 34·9)	−5·93 (−17·1 to 5·72)
	Equatorial Guinea	2·50 (1·81 to 3·47)	27·5 (25·0 to 29·8)	4·27 (−36·2 to 58·2)	1·93 (−10·7 to 15·7)
	Gabon	3·55 (2·71 to 4·54)	21·9 (20·3 to 23·7)	26·1 (−17·1 to 84·3)	17·3 (1·42 to 35·2)
Eastern sub-Saharan Africa		3·14 (2·93 to 3·37)	17·6 (17·1 to 18·0)	−18·6 (−26·5 to −9·69)	−23·7 (−26·4 to −20·4)
	Burundi	4·23 (3·38 to 5·28)	16·3 (15·1 to 17·5)	−31·9 (−52·9 to −4·28)	−36·3 (−43·5 to −28·5)
	Comoros	2·99 (2·31 to 3·76)	23·3 (21·5 to 25·1)	−6·26 (−35·1 to 35·3)	−15·6 (−25·0 to −5·67)
	Djibouti	5·52 (4·36 to 6·90)	42·2 (39·6 to 44·9)	14·4 (−18·8 to 61·1)	15·9 (5·62 to 27·6)
	Eritrea	0·696 (0·517 to 0·906)	12·5 (11·2 to 13·9)	−20·8 (−48·8 to 13·5)	−21·9 (−33·4 to −8·83)
	Ethiopia	1·31 (1·01 to 1·69)	10·5 (9·60 to 11·4)	−3·30 (−33·3 to 37·1)	−15·5 (−26·7 to −2·48)
	Kenya	2·52 (2·00 to 3·20)	19·2 (17·8 to 20·4)	−35·2 (−54·4 to −9·94)	−35·8 (−42·2 to −28·8)
	Madagascar	2·57 (2·00 to 3·30)	24·9 (22·9 to 26·8)	−40·1 (−57·7 to −16·5)	−37·7 (−44·0 to −30·9)
	Malawi	3·11 (2·44 to 3·93)	24·0 (22·6 to 25·4)	−15·5 (−41·7 to 17·6)	−0·233 (−9·99 to 10·1)
	Mozambique	5·94 (4·68 to 7·39)	23·6 (21·6 to 25·7)	−7·30 (−34·1 to 27·9)	−5·52 (−17·2 to 6·57)
	Rwanda	8·51 (7·00 to 10·1)	21·7 (20·4 to 23·2)	−1·82 (−26·1 to 29·3)	−1·51 (−12·1 to 10·9)
	Somalia	2·82 (2·17 to 3·66)	20·5 (18·3 to 22·7)	−17·9 (−43·1 to 12·0)	−15·7 (−26·9 to −4·18)
	South Sudan	2·71 (2·05 to 3·43)	20·6 (18·5 to 22·8)	−18·4 (−42·3 to 9·34)	−17·7 (−28·2 to −7·07)
	Uganda	4·18 (3·43 to 5·02)	14·6 (13·6 to 15·6)	−1·00 (−26·2 to 31·2)	−24·0 (−32·4 to −15·0)
	Tanzania	2·96 (2·35 to 3·68)	18·0 (16·8 to 19·2)	−27·7 (−49·0 to −1·96)	−38·9 (−45·2 to −31·8)
	Zambia	5·82 (4·75 to 6·99)	26·3 (24·9 to 27·7)	−10·0 (−32·5 to 18·6)	4·43 (−5·87 to 16·0)
Southern sub-Saharan Africa		8·70 (7·51 to 10·0)	34·6 (33·3 to 35·7)	−36·8 (−47·8 to −25·7)	−22·7 (−26·0 to −18·9)
	Botswana	8·39 (7·02 to 9·77)	36·3 (34·4 to 38·2)	−19·1 (−38·0 to 7·26)	−6·48 (−14·6 to 1·90)
	eSwatini	2·87 (2·28 to 3·67)	15·0 (13·6 to 16·5)	−26·9 (−47·4 to −0·171)	−30·9 (−38·9 to −21·5)
	Lesotho	1·85 (1·42 to 2·42)	41·5 (39·6 to 43·5)	4·41 (−27·9 to 50·9)	29·5 (17·1 to 42·3)
	Namibia	10·6 (8·83 to 12·7)	23·5 (21·9 to 25·1)	−25·7 (−43·6 to −5·01)	−21·6 (−29·4 to −13·3)
	South Africa	10·4 (8·75 to 12·2)	35·4 (33·8 to 36·9)	−38·0 (−50·2 to −24·8)	−27·5 (−31·3 to −23·3)
	Zimbabwe	3·47 (2·73 to 4·29)	34·4 (32·6 to 36·0)	−8·90 (−35·9 to 24·1)	3·59 (−5·33 to 13·2)
Western sub-Saharan Africa		1·81 (1·64 to 2·02)	12·7 (12·2 to 13·1)	−33·7 (−42·9 to −23·7)	−23·0 (−26·9 to −18·9)
	Benin	1·69 (1·28 to 2·15)	10·6 (9·77 to 11·5)	−45·1 (−63·0 to −23·6)	−42·3 (−49·6 to −34·4)
	Burkina Faso	1·47 (1·10 to 1·93)	17·0 (15·7 to 18·4)	−32·4 (−55·1 to −1·13)	−28·2 (−36·7 to −18·4)
	Cameroon	1·51 (1·14 to 1·94)	14·8 (13·5 to 16·2)	−24·1 (−47·2 to 6·20)	−33·9 (−42·5 to −24·7)
	Cape Verde	3·00 (2·36 to 3·83)	9·39 (8·41 to 10·5)	−35·2 (−54·4 to −9·63)	−45·5 (−53·3 to −36·9)
	Chad	2·83 (2·23 to 3·63)	16·0 (14·7 to 17·4)	−33·7 (−54·0 to −7·61)	−30·4 (−38·9 to −20·5)
	Côte d'Ivoire	3·79 (2·89 to 4·83)	21·8 (20·0 to 23·7)	0·960 (−28·8 to 37·6)	10·9 (−3·13 to 26·9)
	The Gambia	1·33 (1·02 to 1·72)	23·6 (21·9 to 25·3)	−35·0 (−55·2 to −9·14)	−32·4 (−39·2 to −25·1)
	Ghana	2·08 (1·58 to 2·72)	10·5 (9·42 to 11·6)	6·37 (−26·7 to 56·4)	−7·41 (−20·7 to 7·27)
	Guinea	2·03 (1·54 to 2·56)	29·2 (26·6 to 31·9)	−5·65 (−34·9 to 30·1)	−8·87 (−19·3 to 2·07)
	Guinea-Bissau	1·07 (0·811 to 1·39)	8·46 (7·42 to 9·54)	3·39 (−29·1 to 46·7)	16·3 (−2·20 to 38·5)
	Liberia	2·57 (2·03 to 3·24)	13·7 (12·5 to 15·0)	1·62 (−30·8 to 43·3)	−12·8 (−24·2 to 0·646)
	Mali	2·44 (1·83 to 3·22)	22·5 (20·8 to 24·1)	47·6 (−2·39 to 113)	27·0 (12·4 to 44·0)
	Mauritania	8·08 (6·28 to 10·1)	29·3 (26·8 to 31·8)	−12·3 (−39·3 to 20·6)	−21·6 (−29·5 to −11·8)
	Niger	1·63 (1·23 to 2·16)	14·1 (12·8 to 15·6)	18·3 (−21·9 to 68·9)	12·0 (−5·17 to 30·8)
	Nigeria	1·16 (0·862 to 1·54)	7·43 (6·69 to 8·21)	−53·3 (−67·9 to −33·2)	−38·5 (−46·7 to −30·1)
	São Tomé and Príncipe	1·70 (1·29 to 2·22)	7·80 (6·95 to 8·69)	15·1 (−23·8 to 70·5)	24·2 (5·43 to 43·3)
	Senegal	1·46 (1·11 to 1·90)	14·2 (13·2 to 15·3)	−41·2 (−60·4 to −17·5)	−52·3 (−57·5 to −46·0)
	Sierra Leone	7·84 (6·34 to 9·61)	30·5 (28·7 to 32·6)	−26·1 (−46·8 to 0·307)	−13·1 (−21·4 to −3·07)
	Togo	2·26 (1·73 to 2·92)	14·0 (12·8 to 15·2)	−37·3 (−57·5 to −11·9)	−42·8 (−49·8 to −35·5)

Data are given to three significant figures. Data in parentheses are 95% uncertainty intervals.

Since 1990, global age-standardised prevalence of smoking tobacco use among males aged 15 years and older decreased by 27·5% (95% UI 26·5–28·5) and among females decreased by 37·7% (35·4–39·9), with variable progress across countries (table). Prevalence of smoking tobacco use among males aged 15 years and older decreased significantly between 1990 and 2019 in 135 countries (66%), but decreased significantly among females in only 68 countries (33%). The largest decreases were observed in Brazil, where prevalence decreased by 72·5% (70·1–74·7) among males and by 74·7% (71·2–78·0) among females. Among individuals aged 15 years and older, prevalence of smoking tobacco use increased significantly over the past 30 years in 20 countries for males (Afghanistan, Saudi Arabia, Uzbekistan, El Salvador, Antigua and Barbuda, Lesotho, Congo [Brazzaville], Mali, Albania, Lebanon, São Tomé and Príncipe, Bosnia and Herzegovina, Gabon, Djibouti, Egypt, Jordan, Mongolia, Laos, Kiribati, and Indonesia) and in 12 countries for females (Afghanistan, Albania, Kyrgyzstan, Saudi Arabia, Lebanon, Mongolia, Bosnia and Herzegovina, Belarus, Portugal, Lithuania, Russia, and Serbia; table). Across both sexes combined, the largest decreases in age-standardised prevalence of smoking tobacco use were observed in Brazil (73·4% [71·4–75·2]), Norway (53·5% [49·1–57·6]), Senegal (50·9% [44·6–56·0]), Iceland (49·7% [44·5–54·1]), Denmark (49·3% [46·4–52·2]), Haiti (47·5% [40·5–54·4]), Australia (47·5% [43·1–51·8]), Costa Rica (47·4% [40·5–53·6]), Canada (47·4% [42·4–52·0]), and Colombia (47·1% [40·4–53·4]; appendix 2 pp 94–102).

**Figure 1:**
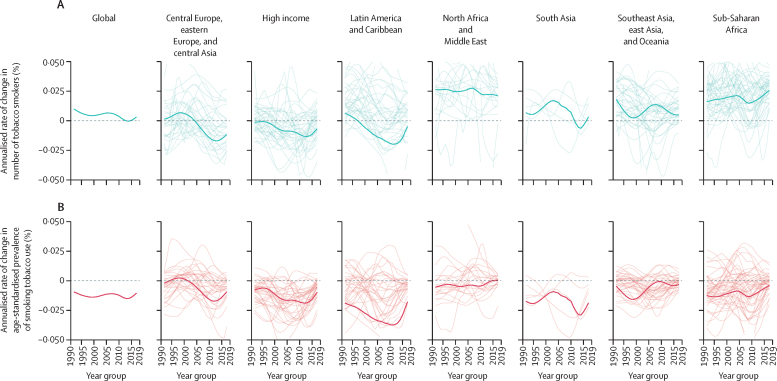
Annualised rate of change in number of tobacco smokers (A) and age-standardised prevalence of smoking tobacco use (B), by super-region Bold lines show regional averages, and thin lines show country-level changes.

Analysing the annualised rate of change per 5-year period in individuals aged 15 years and older between 1990 and 2019, we found the largest number of countries had their fastest decrease in age-standardised prevalence of smoking tobacco use between 2005 and 2009 for both males (68 countries) and females (56 countries; figure 1; country-level data are available online through the GHDx). Overall, 115 (56%) of 204 countries for males and 136 (67%) countries for females had their fastest decrease after the FCTC was ratified (ie, after 2005). Notably, decreases in prevalence were smaller in the period 2015–19 than in the period 2010–15 in 152 (75%) countries for males and 137 (67%) countries for females (figure 1; country-level data are available online through the GHDx).

In many countries, reductions in prevalence have not kept pace with population growth (figure 1; appendix 2 pp 94–102). As a result, the number of smokers globally has increased steadily each year since 1990, when there were 0·99 billion (95% UI 0·98–1·00) smokers globally, with the exception of the period between 2011 and 2017, during which no significant change in the number of smokers occurred (figure 1). Of 204 countries and territories included in our analysis, 113 (55%) had a significant increase in the number of current smokers between 1990 and 2019 and 111 (54%) had a significant increase between 2005 and 2019. Among both males and females, the super-regions with the largest relative increases in the number of smokers since 1990 were north Africa and the Middle East (104·1% [98·1–111] increase) and sub-Saharan Africa (74·6% [69·9–79·1] increase; appendix 2 pp 94–102). The largest relative decreases in the number of smokers were observed in the Latin America and the Caribbean (19·8% [16·9–22·5] decrease) and high-income (17·6% [16·2–18·9] decrease) super-regions.

7·41 trillion (95% UI 7·11–7·74) cigarette-equivalents of tobacco were consumed in 2019, amounting to 20·3 billion (19·5–21·2) cigarette-equivalents consumed each day worldwide. China accounted for more than a third of the world's tobacco consumption (2·72 trillion [2·47–3·01] cigarette-equivalents). Countries with the highest consumption per person in 2019 were mostly in Europe, with Montenegro, North Macedonia, Bulgaria, Slovenia, and Greece all having consumption exceeding 2350 cigarette-equivalents per person (appendix 2 pp 103–111). Countries with the lowest consumption per person were mostly in sub-Saharan Africa (appendix 2 pp 103–110).

**Figure 2:**
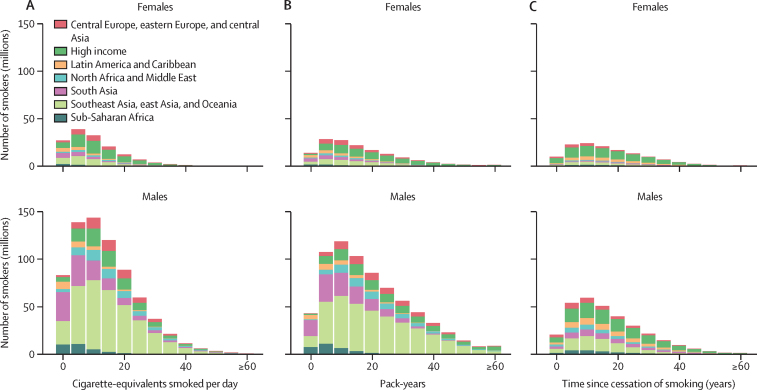
Distribution of number of tobacco smokers aged 30 years and older, by cigarette-equivalents smoked per day (A), and pack-years among current smokers (B), and years since quitting among former smokers (C), by sex and geographical region in 2019 Cigarette-equivalents smoked per day and pack-years are two different indicators of exposure among the current smoker population.

Cigarettes smoked per day is an important predictor of disease risk, although smokers who consume only a few cigarettes each day still have considerable excess risk compared with non-smokers. Among 719 million male current smokers aged 30 years and older in 2019, 83·2 million (11·6%) smoked 1–4 cigarette-equivalents per day, 139·2 million (19·4%) smoked 5–9 cigarette-equivalents per day, 144·0 million (20·0%) smoked 10–14 cigarette-equivalents per day, 120·5 million (16·8%) smoked 15–19 cigarette-equivalents per day, and 231·9 million (32·3%) smoked 20 or more cigarette-equivalents per day (figure 2). Among 146 million female current smokers aged 30 years and older in 2019, 27·0 million (18·5%) smoked 1–4 cigarette-equivalents per day, 39·0 million (26·7%) smoke 5–9 cigarette-equivalents per day, 32·4 million (22·1%) smoked 10–14 cigarette-equivalents per day, 20·8 million (14·2%) smoked 15–19 cigarette-equivalents per day, and 27·1 million (18·5%) smoked 20 or more cigarette-equivalents per day (figure 2). The distribution of cigarette-equivalents per smoker per day varies across countries. Most of the male current smoker population aged 30 years and older in 72 countries and most of the female current smoker population in 121 countries smoke fewer than 10 cigarette-equivalents per day on average (data not shown).

In 2019, 7·69 million (95% UI 7·16–8·20) deaths and 200 million (185–214) DALYs were attributable to smoking tobacco use, accounting for 13·6% (13·0–14·3) of all deaths and 7·89% (7·19–8·56) of all DALYs (appendix 2 pp 112–165). 6·18 million (80%) of these deaths were among males, and smoking accounted for the largest number and proportion (20·2% [19·3–21·1]) of deaths among males of the 87 risk factors included in GBD 2019.^1^ Among females, smoking accounted for 1·51 million (1·40–1·63) deaths and 5·84% (5·47–6·22) of all deaths. YLLs due to smoking tobacco use, which quantify the effects of premature mortality, exceeded YLDs due to smoking in 2019, which quantify the effects of non-fatal health loss, (168 million [156–180] YLLs *vs* 31·6 million [23·7–40·0] YLDs). The ratio of YLLs to YLDs attributable to smoking tobacco use varied across countries, from 1·59 (1·03–2·44) in Qatar to 16·1 (11·2–22·7) in the Solomon Islands (appendix 2 pp 166–174). Ratios of YLLs to YLDs decreased with increasing Socio-demographic Index level (appendix 2 p 175). Lower ratios of YLLs to YLDs indicate that a greater proportion of individuals are living with chronic health conditions due to smoking tobacco use in these countries than in countries with higher ratios of YLLs to YLDs.

Of the 36 health outcomes caused by smoking tobacco use (appendix 2 pp 5–82), the health outcomes with the largest number of deaths attributable to smoking tobacco use for both sexes combined in 2019 were ischaemic heart disease (1·68 million [95% UI 1·56–1·81]); chronic obstructive pulmonary disease (1·59 million [1·41–1·76]); tracheal, bronchus, and lung cancer (1·31 million [1·20–1·43]); and stroke (0·931 million [0·833–1·00]; appendix 2 pp 176–177), which together accounted for approximately 72% of all deaths attributable to smoking tobacco use that year. Top causes of death attributable to smoking varied by region, reflecting differences in both background cause-specific death rates and patterns of smoking. Ischaemic heart disease was the leading cause of deaths attributable to smoking tobacco use in all super-regions except the high-income super-region, for which the leading cause was lung cancer, and for countries in the southeast Asia, east Asia, and Oceania super-region for which the leading cause was chronic obstructive pulmonary disease (appendix 2 pp 176–177).

An estimated 5·96 million (77·5%) of 7·69 million deaths attributable to smoking tobacco use occurred in low-income and middle-income countries in 2019. Ukraine had the highest death rate from smoking among males (487 per 100 000 males [95% UI 396–590; appendix 2 pp 112–138). Countries with high rates of deaths attributable to smoking tobacco use among males were predominantly in the central Europe, eastern Europe, and east Asia regions. Among females, four locations had rates of deaths attributable to smoking tobacco use higher than 180 per 100 000 females (Denmark, Montenegro, Serbia, and Greenland; appendix 2 pp 112–138).

**Figure 3:**
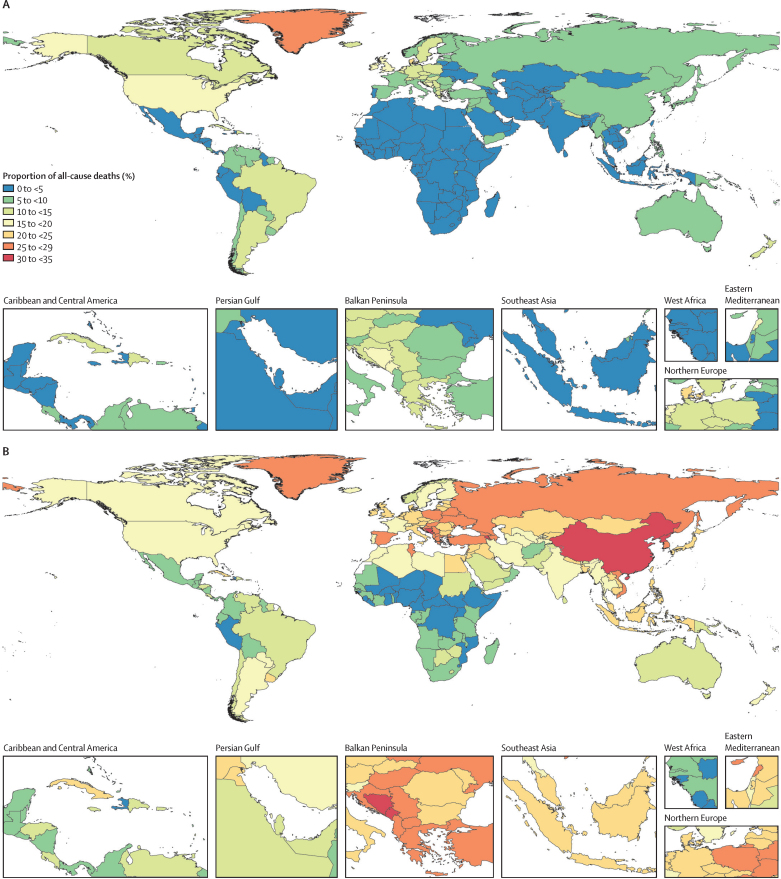
Proportion of all-cause deaths that were attributable to smoking tobacco use among females (A) and males (B) of all ages in 2019

Across all age groups, smoking tobacco use was the cause of more than 20% of all male deaths in 73 countries in 2019 (figure 3). 43 (59%) of 73 countries were designated as low-income or middle-income countries. Among all females, smoking accounted for more than 20% of deaths in only two locations (Denmark and Greenland), due to both lower smoking prevalence, shorter duration of smoking, and lower smoking intensity among females than among males (figure 3). The proportion of deaths attributable to smoking tobacco use increased with age, peaking among those aged 60–64 years (22·0% [95% UI 21·1–23·1] of deaths attributable to smoking), before decreasing in older age groups (appendix 2 p 178).

The share of all-cause deaths that were due to smoking tobacco use decreased significantly between 1990 and 2019 in 68 countries, increased significantly in 71 countries, and stayed constant in 65 countries (appendix 2 pp 179–187). 66 (93%) of 71 countries with significant increases in the proportion of all-cause deaths attributable to smoking tobacco use were low-income and middle-income countries. The largest decreases were observed in Australia, New Zealand, South Africa, Singapore, and Norway, whereas the largest increases were observed in Timor-Leste, Bhutan, Niger, São Tomé and Príncipe, and Malawi (appendix 2 pp 179–187). The largest absolute increases in the number of deaths attributable to tobacco smoking between 1990 and 2019 were observed in China (from 1·5 million deaths in 1990 to 2·4 million in 2019; a 57·9% [26·2–101] increase), India (from 0·6 million deaths in 1990 to 1·0 million in 2019; a 58·9% [30·7–90·8] increase), and Indonesia (from 112 800 deaths in 1990 to 246 400 deaths in 2019; a 118% [74·0–171] increase; appendix 2 pp 179–187).

**Figure 4:**
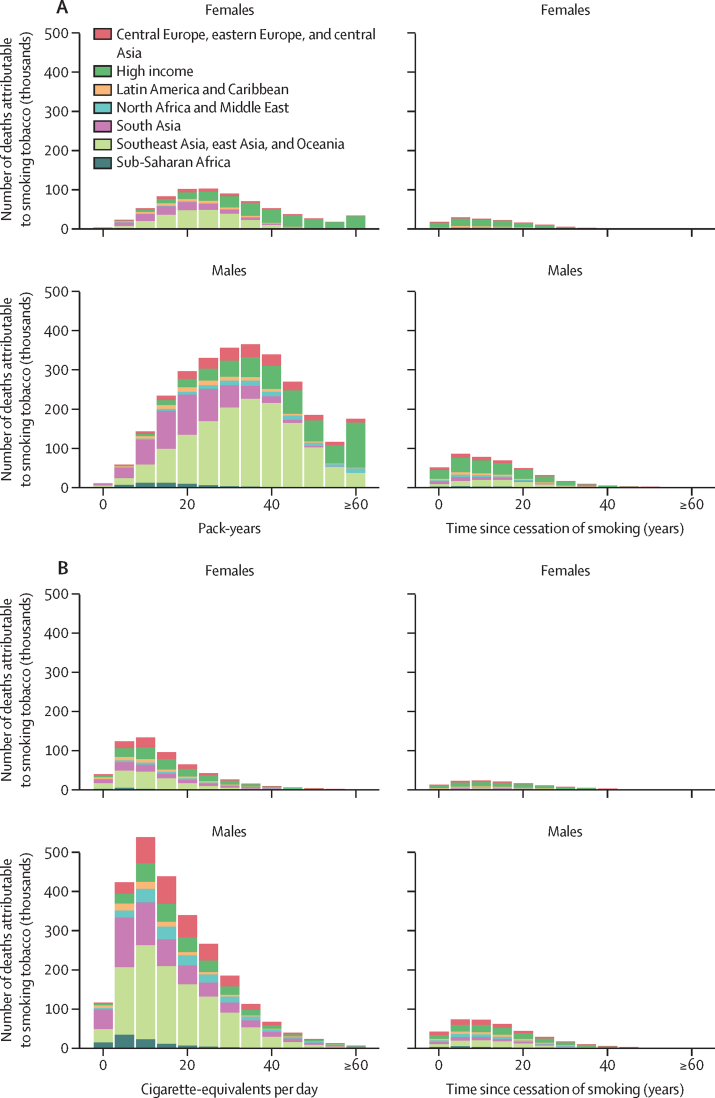
Number of deaths from cancers and chronic obstructive pulmonary disease (A) and deaths from cardiovascular and circulatory diseases and all other health outcomes (excluding cancers and chronic obstructive pulmonary disease; B) attributable to smoking tobacco use in individuals aged 30 years and older, by level of exposure, sex, and super-region

The dose-response association between risk exposure and disease results in an uneven distribution of burden among the current and former smoking populations aged 30 years and older (figure 4). Among ever smokers aged 30 years and older, 865 million (65·9%) of 1·31 billion are current smokers and 449 million (34·1%) are former smokers. A disproportionate share of all deaths attributable to smoking tobacco use occurred among current smokers (6·68 million [86·9%] of 7·69 million). Among former smokers, health risks decreased as a function of years since cessation (figure 4). Only 0·467 million (6·18%) global deaths attributable to smoking tobacco use occurred among individuals who had quit smoking at least 15 years ago, despite this group accounting for 257 million (19·6%) members of the global ever smoker population.

## Discussion

In this study, we present updated results on the prevalence of smoking tobacco use and the attributable disease burden from GBD 2019. We used new direct estimation methods, which allowed more comprehensive estimations, including reflecting dose-response associations between exposure and risk of disease and capture of health effects among daily and occasional smokers and former smokers. By using continuous exposure measures, we are better able to capture differences in risk across countries that result from heterogenous smoking patterns. Our findings are an urgent call to action for countries to implement and enforce stronger tobacco control policies than are currently in place, and serve as a blueprint for targeting interventions, monitoring progress, allocating resources, and planning for future health system strain.

In 2019, more than 1 billion people smoked tobacco regularly, and almost 8 million deaths were attributable to smoking. Smoking tobacco use accounted for 20·2% of all-cause deaths among males, and was the leading risk factor for both deaths and DALYs among males. Among females, smoking tobacco use accounted for approximately 5·8% of all deaths, due to lower prevalence, shorter duration, and lower intensity of smoking than in males. Tobacco control has contributed to reductions in global prevalence of smoking tobacco use of 27·5% (95% UI 26·5–28·5) for males and 37·7% (35·4–39·9) for females. However, these global aggregates do not illustrate important between-country heterogeneity. Between 1990 and 2019, significant decreases in prevalence of smoking tobacco use were observed in 135 countries for males and 68 countries for females, while significant increases were observed in 20 countries for males and 12 countries for females.

Although tobacco control efforts began as early as in the 1960s in some countries after the detrimental health effects of tobacco smoking were first documented, global progress in tobacco control was catalysed with the passing of the WHO FCTC in 2005.^9^, ^33^ The decade following the introduction of the WHO FCTC was the period of fastest decrease in the prevalence of smoking tobacco use across the largest number of countries.^34^ The effectiveness of the demand-reduction policies outlined in the FCTC articles has been documented, and the large reductions in prevalence of smoking tobacco use between 1990 and 2019 were observed in Brazil (73·4% decrease), Norway (53·5% decrease), and Senegal (50·9% decrease), along with Iceland, Denmark, Canada, Australia, Colombia, and Costa Rica, all with decreases in prevalence exceeding 45%, show the potential of these tools to operate in diverse contexts to greatly reduce the prevalence of smoking tobacco use and save millions of lives over the coming decades.^35^, ^36^, ^37^, ^38^

Despite these successes, we observed three concerning patterns. First, in several countries with large populations and high prevalence of smoking tobacco use, including China (2·4 million deaths in 2019, a 57·9% [95% UI 26·2–101] increase in attributable deaths since 1990) and Indonesia (246 400 deaths in 2019, a 118% [74·0–171] increase in attributable deaths since 1990), little to no progress has been made in reducing the prevalence of smoking. Second, most countries did not have sufficient decreases in the prevalence of smoking to offset the demographic force of population growth, resulting in constant or increasing numbers of smokers over time. And third, in many countries, including those that had large decreases in prevalence previously, the rate of progress has slowed, particularly in the past 5 years.

113 of 204 countries had a significant increase in the number of smokers since 1990, and 111 countries had a significant increase since 2005. To achieve the goals set forth in the SDGs and WHO global non-communicable disease monitoring framework, and in doing so reclaim the 200 million DALYs lost to smoking tobacco use each year, most countries will need stronger tobacco control policies than those already in place. As of 2018, only 62 countries had comprehensive smoke-free policies; 23 offered the full range of cessation support services recommended by WHO; 91 mandated best-practice pictorial health warnings; 48 were protected by complete advertising, promotion, and sponsorship bans; and 38 had the recommended level of tobacco taxation.^10^ Closing gaps in the adoption, implementation, and enforcement of evidence-based demand-reduction policies is vital to ending the global tobacco epidemic.

Taxation is one of the most effective tobacco control policies available to countries.^4^ Increasing taxes decreases demand by reducing the affordability of tobacco products. As income and purchasing power increase, particularly in rapidly developing countries, concordant increases in tobacco taxes to reduce affordability are necessary for this fiscal policy to remain potent. Yet, between 2008 and 2018, the affordability of cigarettes decreased in only 33% of low-income countries compared with in 38% of middle-income countries and 72% of high-income countries.^10^ Only one low-income country, Madagascar, taxes tobacco at the rate recommended by the WHO MPOWER framework. Low-income and middle-income countries face the additional challenge of population growth expanding their smoking population. Tobacco taxation is a highly cost-effective measure, and when combined with a progressive approach to redistributing revenue from taxation to tobacco control programmes, health care, and other social support services, can greatly reduce the prevalence of smoking and substantially improve population health.^39^

With 1·14 billion current smokers globally in 2019, increasing cessation rates among current smokers can yield massive health gains. We estimated that fewer than 15% of deaths attributable to smoking tobacco use in 2019 occurred among former smokers aged 30 years and older, despite former smokers comprising approximately a third of the ever smoker population aged 30 years and older. Consistent with other studies, we found in our meta-analyses of risk reduction among former smokers that cessation shifts smokers into a lower-risk category, with greater reduction in risk for longer durations since cessation.^40^, ^41^, ^42^ Relative risks of all-cause mortality from long-running cohort studies in the UK^41^, ^42^ and the USA^43^ indicate that up to two-thirds of long-term smokers will eventually die of a disease attributable to smoking. These data underscore the importance of adopting policies and interventions that increase rates of cessation.

The results of our study must be interpreted in the context of several limitations. First, data on smoking tobacco use are self-reported, which might lead to underestimates in demographic groups with low social acceptability of smoking, particularly among females in Asia and Africa.^44^ Second, the scope of our study focuses on smoked tobacco products, and does not include smokeless tobacco products, e-cigarettes, heated tobacco products, or other electronic nicotine delivery systems. Incorporating the health effects of the array of emerging electronic nicotine delivery systems, including both potential benefits and potential harms, is becoming increasingly important. Additionally, our analysis focuses on the health effects of primary smoking of tobacco and does not include additional harms due to second-hand smoke. Third, we converted non-cigarette smoked tobacco products to cigarette-equivalents on the basis of the weight of tobacco. Conversion on the basis of health effect equivalencies would be more accurate, but a paucity of evidence on the health effects of non-cigarette tobacco products presents challenges to this alternative approach. Fourth, the optimal lag-time between exposure and outcome exceeded 10 years for five outcomes (ischaemic heart disease, lower respiratory tract infections, aortic aneurysm, peripheral artery disease, and peptic ulcer disease). Due to the paucity of data on smoking patterns before 1980, we were restricted to using a maximum lag-time of 10 years. Fifth, the rate of risk reduction among former smokers probably varies by smoking intensity. Although we captured differences in the level of risk, we did not capture differences in the rate of risk reduction in our estimates. Despite these limitations, our results are broadly consistent with previous estimates that use different methods with a different set of limitations.^20^

Smoking remains a defining challenge in global health. Governments, and particularly ministers of health, face substantial obstacles ranging from population growth, to pressure from the tobacco industry, to competing health and political priorities. Nevertheless, it is increasingly important for all countries to adopt, implement, and enforce comprehensive packages of evidence-based tobacco control policies. The current level of tobacco control policy implementation is insufficient in many countries around the world. With more than 1 billion people smoking tobacco globally as of 2019, the annual death toll, economic costs, and burden to health systems caused by smoking will increase in the years to come unless countries take swift and strong action to substantially reduce their smoking rates.

**This online publication has been corrected. The corrected version first appeared at thelancet.com on June 2, 2021**

## Data sharing

To download the data used in these analyses, please visit the Global Health Data Exchange GBD 2019 website.

## Declaration of interests

ViA reports personal fees from Bayer Healthcare, Boehringer Ingelheim/Lilly alliance, Bristol Myers Squibb/Pfizer alliance, and Novo Nordisk outside of the submitted work. RA reports consultancy and speakers' fees from UCB, Sandoz, AbbVie, Zentiva, Teva, Laropharm, CEGEDIM, Angelini, Biessen Pharma, Hofigal, AstraZeneca, and Stada outside of the submitted work. BeA reports personal fees from Australian Institute of Sports; non-financial support from Zydus Cadila; and grants and non-financial support from Natural Remedies outside of the submitted work. FG was employed by Public Health England during the conduct of the study, which does not necessarily endorse this study. GJH reports personal fees from the American Heart Association outside of the submitted work. SMSI reports grants from National Heart Foundation of Australia and from the Australian National Health and Medical Research Council (NHMRC) outside of the submitted work. SVK reports grants from Chief Scientist Office and UK Medical Research Council during the conduct of the study. KK reports non-financial support from UGC Centre of Advanced Study (CAS II), Department of Anthropology, Panjab University, Chandigarh, India, outside of the submitted work. StL reports personal fees from Akcea Therapeutics, Amedes, AMGEN, Berlin-Chemie, Boehringer Ingelheim Pharma, Daiichi Sankyo, Lilly, MSD Sharp & Dohme, Novo Nordisk, Sanofi-Aventis, Synlab, Unilever, and Upfield, and non-financial support from Preventicus outside of the submitted work. WM is Program Analyst in Population and Development at the UN Population Fund-UNFPA Country Office in Peru, which does not necessarily endorse this study. TRM reports contracts from Gov't Plaintiff Lawyers, JUUL, outside of the submitted work. BoN reports personal fees from AstraZeneca and Bayer, outside of the submitted work. SimS reports grants, personal fees, and non-financial support from Abbott and Novartis; personal fees and non-financial support from Allergan-Abbvie, AstraZeneca, and Teva; and personal fees from Eli Lilly and Novo Nordisk outside of the submitted work. AES reports personal fees from Takeda, Novartis, Servier, and Omron Healthcare outside of the submitted work. JAS reports consultancy fees from Crealta/Horizon, Medisys, Fidia, Two Labs Inc, Adept Field Solutions, Clinical Care options, Clearview Healthcare Partners, Putnam Associates, Focus Forward, Navigant Consulting, Spherix, MedIQ, UBM, Trio Health, Medscape, WebMD, Practice Point communications, the National Institutes of Health, and the American College of Rheumatology; payment for lectures including service on Simply Speaking speaker's bureau; and stock ownership in TPT Global Tech, Vaxart pharmaceuticals, and Charlotte's Web Holdings. JAS previously owned stock options in Amarin, Viking, and Moderna pharmaceuticals; held placement on the steering committee of OMERACT, an international organisation that develops measures for clinical trials and receives arm's length funding from 12 pharmaceutical companies; serves on the US Food and Drug Administration Arthritis Advisory Committee; is a member of the Veterans Affairs Rheumatology Field Advisory Committee; and is the editor and the director of the UAB Cochrane Musculoskeletal Group Satellite Center on Network Meta-analysis. DJS reports personal fees from Lundbeck, Takeda, Johnson & Johnson, and Servier outside of the submitted work. StS reports grants from Edwards Lifesciences, Medtronic, Boston Scientific, and Abbott; and personal fees from Boston Scientific, Teleflex, and BTG outside of the submitted work. JS reports ownership in companies providing services to Itrim, Amgen, Janssen, Novo Nordisk, Eli Lilly, Boehringer, Bayer, Pfizer, and AstraZeneca outside of the submitted work. FT reports grants and personal fees from Novartis, Thea, Alcon, Pfizer, and Bayer; grants from Bausch & Lomb; and personal fees from Allergan, Omikron, and Santen outside of the submitted work. All other authors declare no competing interests.
