# Department of Error

**DOI:** 10.1016/S0140-6736(21)01282-4

**Published:** 2021-06-19

*GBD 2019 Tobacco Collaborators. Spatial, temporal, and demographic patterns in prevalence of smoking tobacco use and attributable disease burden in 204 countries and territories, 1990–2019: a systematic analysis from the Global Burden of Disease Study 2019.* Lancet *2021; **397:** 2337–60*—In this Article, the GBD 2019 Tobacco Collaborators list and accompanying affiliations have been updated. These corrections have been made to the online version as of June 2, 2021, and the printed version is correct.

