# Supplementary material for: Spatial, temporal, and demographic patterns in prevalence of smoking tobacco use and attributable disease burden in 204 countries and territories, 1990–2019: a systematic analysis from the Global Burden of Disease Study 2019
Source: Lancet. 2021 Jun 19;397(10292):2337–60. doi: 10.1016/S0140-6736(21)01169-7 (PMC8223261; doi:10.1016/S0140-6736(21)01169-7)
Supplement: Supplementary appendix 1 [file mmc1.pdf]

THE LANCET

Supplementary appendix

This appendix formed part of the original submission and has been peer reviewed. We post it as supplied by the authors.

Supplement to: GBD 2019 Tobacco Collaborators. Spatial, temporal, and demographic patterns in prevalence of smoking tobacco use and attributable disease burden in 204 countries and territories, 1990–2019: a systematic analysis from the Global Burden of Disease Study 2019. *Lancet* 2021; published online May 27. http://dx.doi.org/10.1016/S0140-6736(21)01169-7.

Supplementary Methods

Spatial, temporal, and demographic patterns in prevalence of smoking tobacco use and attributable disease burden in 204 countries and territories, 1990-2019: a systematic analysis from the Global Burden of Disease Study 2019

GBD 2019 Tobacco Collaborators

Table of Contents

Locations Estimated 3

Demographics 9

Model Flowchart 10

Exposure 11

Case Definitions 11
Inclusion Criteria 11
Data extraction 11
Adjustment for Non-Standard Case Definitions 13
Age and Sex Splitting 20
Smoking Prevalence Modelling 21
Dose-Response Exposure Among Current and Former Smokers 22
Supply-Side Consumption 22
Ensemble Distributions 24

Risk-Outcome Pairs 25

Dose-response risk curves 26

PAF Calculation 28

Analytic Code 28

GATHER Checklist 29

PRISMA Flowcharts 31

Author Contributions 67

Locations Estimated

For the Global Burden of Diseases, Injuries, and Risk Factors Study 2019 (GBD 2019), we produced estimates for 204 countries and territories (Table SM1). These countries and territories were nested in 21 aggregate regions and seven aggregate super-regions. Standard regions and super-regions in the GBD are defined based on a combination of epidemiologic patterns and spatial distance.

Table SM1. Location Hierarchy

Location	Super-Region	Region
**Armenia**	Central Europe, Eastern Europe, and Central Asia	Central Asia
**Azerbaijan**	Central Europe, Eastern Europe, and Central Asia	Central Asia
**Georgia**	Central Europe, Eastern Europe, and Central Asia	Central Asia
**Kazakhstan**	Central Europe, Eastern Europe, and Central Asia	Central Asia
**Kyrgyzstan**	Central Europe, Eastern Europe, and Central Asia	Central Asia
**Mongolia**	Central Europe, Eastern Europe, and Central Asia	Central Asia
**Tajikistan**	Central Europe, Eastern Europe, and Central Asia	Central Asia
**Turkmenistan**	Central Europe, Eastern Europe, and Central Asia	Central Asia
**Uzbekistan**	Central Europe, Eastern Europe, and Central Asia	Central Asia
**Albania**	Central Europe, Eastern Europe, and Central Asia	Central Europe
**Bosnia and Herzegovina**	Central Europe, Eastern Europe, and Central Asia	Central Europe
**Bulgaria**	Central Europe, Eastern Europe, and Central Asia	Central Europe
**Croatia**	Central Europe, Eastern Europe, and Central Asia	Central Europe
**Czechia**	Central Europe, Eastern Europe, and Central Asia	Central Europe
**Hungary**	Central Europe, Eastern Europe, and Central Asia	Central Europe
**Montenegro**	Central Europe, Eastern Europe, and Central Asia	Central Europe
**North Macedonia**	Central Europe, Eastern Europe, and Central Asia	Central Europe
**Poland**	Central Europe, Eastern Europe, and Central Asia	Central Europe
**Romania**	Central Europe, Eastern Europe, and Central Asia	Central Europe
**Serbia**	Central Europe, Eastern Europe, and Central Asia	Central Europe
**Slovakia**	Central Europe, Eastern Europe, and Central Asia	Central Europe
**Slovenia**	Central Europe, Eastern Europe, and Central Asia	Central Europe
**Belarus**	Central Europe, Eastern Europe, and Central Asia	Eastern Europe
**Estonia**	Central Europe, Eastern Europe, and Central Asia	Eastern Europe
**Latvia**	Central Europe, Eastern Europe, and Central Asia	Eastern Europe
**Lithuania**	Central Europe, Eastern Europe, and Central Asia	Eastern Europe
**Republic of Moldova**	Central Europe, Eastern Europe, and Central Asia	Eastern Europe

**Russia**	Central Europe, Eastern Europe, and Central Asia	Eastern Europe
**Ukraine**	Central Europe, Eastern Europe, and Central Asia	Eastern Europe
**Australia**	High-income	Australasia
**New Zealand**	High-income	Australasia
**Brunei Darussalam**	High-income	High-income Asia Pacific
**Japan**	High-income	High-income Asia Pacific
**Republic of Korea**	High-income	High-income Asia Pacific
**Singapore**	High-income	High-income Asia Pacific
**Canada**	High-income	High-income North America
**Greenland**	High-income	High-income North America
**United States of America**	High-income	High-income North America
**Argentina**	High-income	Southern Latin America
**Chile**	High-income	Southern Latin America
**Uruguay**	High-income	Southern Latin America
**Andorra**	High-income	Western Europe
**Austria**	High-income	Western Europe
**Belgium**	High-income	Western Europe
**Cyprus**	High-income	Western Europe
**Denmark**	High-income	Western Europe
**Finland**	High-income	Western Europe
**France**	High-income	Western Europe
**Germany**	High-income	Western Europe
**Greece**	High-income	Western Europe
**Iceland**	High-income	Western Europe
**Ireland**	High-income	Western Europe
**Israel**	High-income	Western Europe
**Italy**	High-income	Western Europe
**Luxembourg**	High-income	Western Europe
**Malta**	High-income	Western Europe
**Monaco**	High-income	Western Europe
**Netherlands**	High-income	Western Europe
**Norway**	High-income	Western Europe
**Portugal**	High-income	Western Europe
**San Marino**	High-income	Western Europe
**Spain**	High-income	Western Europe
**Sweden**	High-income	Western Europe
**Switzerland**	High-income	Western Europe
**United Kingdom**	High-income	Western Europe
**Bolivia (Plurinational State of)**	Latin America and Caribbean	Andean Latin America

**Ecuador**	Latin America and Caribbean	Andean Latin America
**Peru**	Latin America and Caribbean	Andean Latin America
**Antigua and Barbuda**	Latin America and Caribbean	Caribbean
**Bahamas**	Latin America and Caribbean	Caribbean
**Barbados**	Latin America and Caribbean	Caribbean
**Belize**	Latin America and Caribbean	Caribbean
**Bermuda**	Latin America and Caribbean	Caribbean
**Cuba**	Latin America and Caribbean	Caribbean
**Dominica**	Latin America and Caribbean	Caribbean
**Dominican Republic**	Latin America and Caribbean	Caribbean
**Grenada**	Latin America and Caribbean	Caribbean
**Guyana**	Latin America and Caribbean	Caribbean
**Haiti**	Latin America and Caribbean	Caribbean
**Jamaica**	Latin America and Caribbean	Caribbean
**Puerto Rico**	Latin America and Caribbean	Caribbean
**Saint Kitts and Nevis**	Latin America and Caribbean	Caribbean
**Saint Lucia**	Latin America and Caribbean	Caribbean
**Saint Vincent and the Grenadines**	Latin America and Caribbean	Caribbean
**Suriname**	Latin America and Caribbean	Caribbean
**Trinidad and Tobago**	Latin America and Caribbean	Caribbean
**United States Virgin Islands**	Latin America and Caribbean	Caribbean
**Colombia**	Latin America and Caribbean	Central Latin America
**Costa Rica**	Latin America and Caribbean	Central Latin America
**El Salvador**	Latin America and Caribbean	Central Latin America
**Guatemala**	Latin America and Caribbean	Central Latin America
**Honduras**	Latin America and Caribbean	Central Latin America
**Mexico**	Latin America and Caribbean	Central Latin America
**Nicaragua**	Latin America and Caribbean	Central Latin America
**Panama**	Latin America and Caribbean	Central Latin America
**Venezuela (Bolivarian Republic of)**	Latin America and Caribbean	Central Latin America
**Brazil**	Latin America and Caribbean	Tropical Latin America
**Paraguay**	Latin America and Caribbean	Tropical Latin America
**Afghanistan**	North Africa and Middle East	North Africa and Middle East
**Algeria**	North Africa and Middle East	North Africa and Middle East
**Bahrain**	North Africa and Middle East	North Africa and Middle East
**Egypt**	North Africa and Middle East	North Africa and Middle East
**Iran (Islamic Republic of)**	North Africa and Middle East	North Africa and Middle East
**Iraq**	North Africa and Middle East	North Africa and Middle East

**Jordan**	North Africa and Middle East	North Africa and Middle East
**Kuwait**	North Africa and Middle East	North Africa and Middle East
**Lebanon**	North Africa and Middle East	North Africa and Middle East
**Libya**	North Africa and Middle East	North Africa and Middle East
**Morocco**	North Africa and Middle East	North Africa and Middle East
**Oman**	North Africa and Middle East	North Africa and Middle East
**Palestine**	North Africa and Middle East	North Africa and Middle East
**Qatar**	North Africa and Middle East	North Africa and Middle East
**Saudi Arabia**	North Africa and Middle East	North Africa and Middle East
**Sudan**	North Africa and Middle East	North Africa and Middle East
**Syrian Arab Republic**	North Africa and Middle East	North Africa and Middle East
**Tunisia**	North Africa and Middle East	North Africa and Middle East
**Turkey**	North Africa and Middle East	North Africa and Middle East
**United Arab Emirates**	North Africa and Middle East	North Africa and Middle East
**Yemen**	North Africa and Middle East	North Africa and Middle East
**Bangladesh**	South Asia	South Asia
**Bhutan**	South Asia	South Asia
**India**	South Asia	South Asia
**Nepal**	South Asia	South Asia
**Pakistan**	South Asia	South Asia
**China**	Southeast Asia, East Asia, and Oceania	East Asia
**Democratic People's Republic of Korea**	Southeast Asia, East Asia, and Oceania	East Asia
**Taiwan (Province of China)**	Southeast Asia, East Asia, and Oceania	East Asia
**American Samoa**	Southeast Asia, East Asia, and Oceania	Oceania
**Cook Islands**	Southeast Asia, East Asia, and Oceania	Oceania
**Fiji**	Southeast Asia, East Asia, and Oceania	Oceania
**Guam**	Southeast Asia, East Asia, and Oceania	Oceania
**Kiribati**	Southeast Asia, East Asia, and Oceania	Oceania
**Marshall Islands**	Southeast Asia, East Asia, and Oceania	Oceania
**Micronesia (Federated States of)**	Southeast Asia, East Asia, and Oceania	Oceania
**Nauru**	Southeast Asia, East Asia, and Oceania	Oceania
**Niue**	Southeast Asia, East Asia, and Oceania	Oceania
**Northern Mariana Islands**	Southeast Asia, East Asia, and Oceania	Oceania
**Palau**	Southeast Asia, East Asia, and Oceania	Oceania
**Papua New Guinea**	Southeast Asia, East Asia, and Oceania	Oceania
**Samoa**	Southeast Asia, East Asia, and Oceania	Oceania
**Solomon Islands**	Southeast Asia, East Asia, and Oceania	Oceania
**Tokelau**	Southeast Asia, East Asia, and Oceania	Oceania

**Tonga**	Southeast Asia, East Asia, and Oceania	Oceania
**Tuvalu**	Southeast Asia, East Asia, and Oceania	Oceania
**Vanuatu**	Southeast Asia, East Asia, and Oceania	Oceania
**Cambodia**	Southeast Asia, East Asia, and Oceania	Southeast Asia
**Indonesia**	Southeast Asia, East Asia, and Oceania	Southeast Asia
**Lao People's Democratic Republic**	Southeast Asia, East Asia, and Oceania	Southeast Asia
**Malaysia**	Southeast Asia, East Asia, and Oceania	Southeast Asia
**Maldives**	Southeast Asia, East Asia, and Oceania	Southeast Asia
**Mauritius**	Southeast Asia, East Asia, and Oceania	Southeast Asia
**Myanmar**	Southeast Asia, East Asia, and Oceania	Southeast Asia
**Philippines**	Southeast Asia, East Asia, and Oceania	Southeast Asia
**Seychelles**	Southeast Asia, East Asia, and Oceania	Southeast Asia
**Sri Lanka**	Southeast Asia, East Asia, and Oceania	Southeast Asia
**Thailand**	Southeast Asia, East Asia, and Oceania	Southeast Asia
**Timor-Leste**	Southeast Asia, East Asia, and Oceania	Southeast Asia
**Viet Nam**	Southeast Asia, East Asia, and Oceania	Southeast Asia
**Angola**	Sub-Saharan Africa	Central Sub-Saharan Africa
**Central African Republic**	Sub-Saharan Africa	Central Sub-Saharan Africa
**Congo**	Sub-Saharan Africa	Central Sub-Saharan Africa
**Democratic Republic of the Congo**	Sub-Saharan Africa	Central Sub-Saharan Africa
**Equatorial Guinea**	Sub-Saharan Africa	Central Sub-Saharan Africa
**Gabon**	Sub-Saharan Africa	Central Sub-Saharan Africa
**Burundi**	Sub-Saharan Africa	Eastern Sub-Saharan Africa
**Comoros**	Sub-Saharan Africa	Eastern Sub-Saharan Africa
**Djibouti**	Sub-Saharan Africa	Eastern Sub-Saharan Africa
**Eritrea**	Sub-Saharan Africa	Eastern Sub-Saharan Africa
**Ethiopia**	Sub-Saharan Africa	Eastern Sub-Saharan Africa
**Kenya**	Sub-Saharan Africa	Eastern Sub-Saharan Africa
**Madagascar**	Sub-Saharan Africa	Eastern Sub-Saharan Africa
**Malawi**	Sub-Saharan Africa	Eastern Sub-Saharan Africa
**Mozambique**	Sub-Saharan Africa	Eastern Sub-Saharan Africa
**Rwanda**	Sub-Saharan Africa	Eastern Sub-Saharan Africa
**Somalia**	Sub-Saharan Africa	Eastern Sub-Saharan Africa
**South Sudan**	Sub-Saharan Africa	Eastern Sub-Saharan Africa
**Uganda**	Sub-Saharan Africa	Eastern Sub-Saharan Africa
**United Republic of Tanzania**	Sub-Saharan Africa	Eastern Sub-Saharan Africa
**Zambia**	Sub-Saharan Africa	Eastern Sub-Saharan Africa

**Botswana**	Sub-Saharan Africa	Southern Sub-Saharan Africa
**Eswatini**	Sub-Saharan Africa	Southern Sub-Saharan Africa
**Lesotho**	Sub-Saharan Africa	Southern Sub-Saharan Africa
**Namibia**	Sub-Saharan Africa	Southern Sub-Saharan Africa
**South Africa**	Sub-Saharan Africa	Southern Sub-Saharan Africa
**Zimbabwe**	Sub-Saharan Africa	Southern Sub-Saharan Africa
**Benin**	Sub-Saharan Africa	Western Sub-Saharan Africa
**Burkina Faso**	Sub-Saharan Africa	Western Sub-Saharan Africa
**Cabo Verde**	Sub-Saharan Africa	Western Sub-Saharan Africa
**Cameroon**	Sub-Saharan Africa	Western Sub-Saharan Africa
**Chad**	Sub-Saharan Africa	Western Sub-Saharan Africa
**Côte d'Ivoire**	Sub-Saharan Africa	Western Sub-Saharan Africa
**Gambia**	Sub-Saharan Africa	Western Sub-Saharan Africa
**Ghana**	Sub-Saharan Africa	Western Sub-Saharan Africa
**Guinea**	Sub-Saharan Africa	Western Sub-Saharan Africa
**Guinea-Bissau**	Sub-Saharan Africa	Western Sub-Saharan Africa
**Liberia**	Sub-Saharan Africa	Western Sub-Saharan Africa
**Mali**	Sub-Saharan Africa	Western Sub-Saharan Africa
**Mauritania**	Sub-Saharan Africa	Western Sub-Saharan Africa
**Niger**	Sub-Saharan Africa	Western Sub-Saharan Africa
**Nigeria**	Sub-Saharan Africa	Western Sub-Saharan Africa
**São Tomé and Príncipe**	Sub-Saharan Africa	Western Sub-Saharan Africa
**Senegal**	Sub-Saharan Africa	Western Sub-Saharan Africa
**Sierra Leone**	Sub-Saharan Africa	Western Sub-Saharan Africa
**Togo**	Sub-Saharan Africa	Western Sub-Saharan Africa

Demographics

For the 204 countries and territories included in analysis, we produced estimates by sex and five-year age group from 1990 to 2019. Additionally, we produced estimates aggregated by age:

- All-age smoking prevalence reflects population-weighted estimates for ages 15 and above.
- Age-standardised smoking prevalence reflects weighting according to the GBD population standard for ages 15 and above.
- Smoking attributable burden is calculated for ages 30 and above, as there is insufficient evidence to estimate the health effects of primary smoking among individuals under age 30.
    - Despite this age-restriction, all-age attributable burden reflects the share of burden across individuals of all ages (0+) in order to avoid overstatements of the population-wide health impacts of smoking.

Model Flowchart

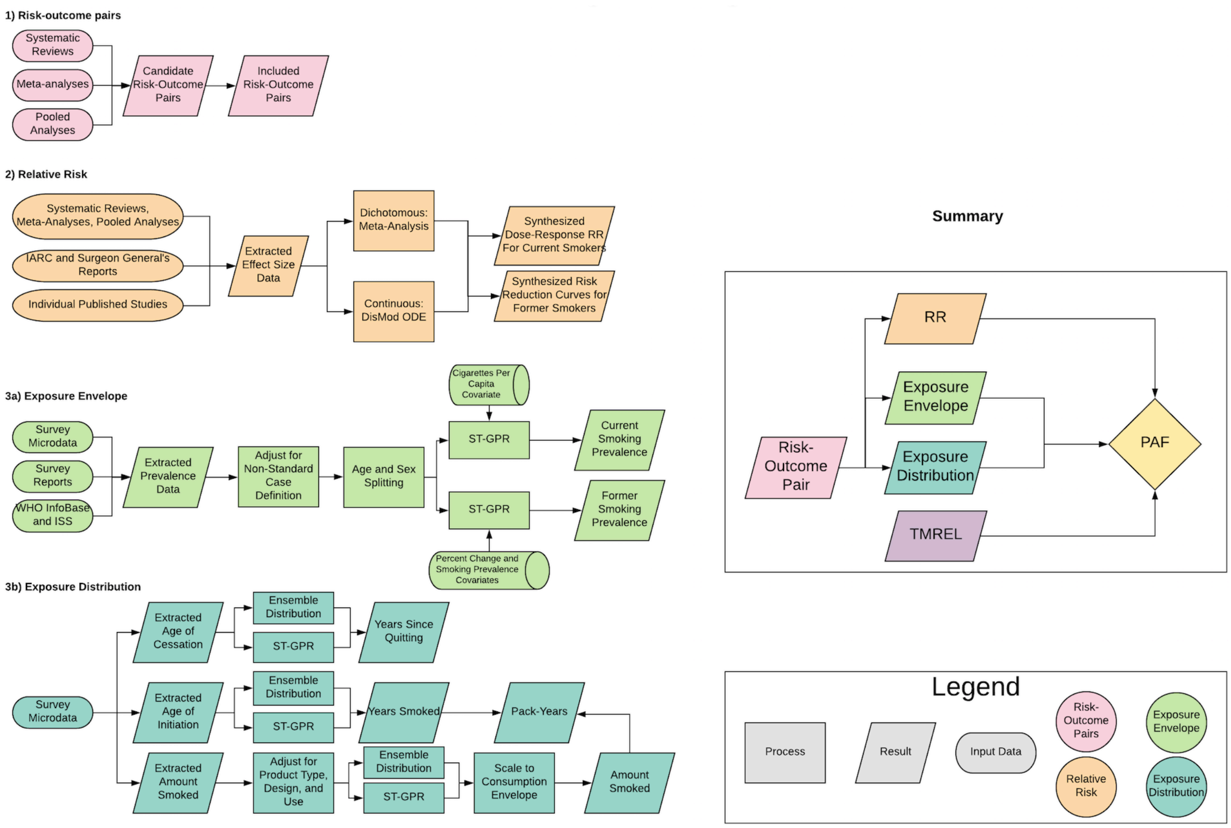

Exposure

Case Definitions

We defined current smokers as individuals who currently use any smoked tobacco product on a daily or occasional basis. This includes both manufactured and hand-rolled cigarettes, cigars, pipes, hookah, bidis, and all other forms of smoked tobacco.

We defined former smokers as individuals who quit using all smoked tobacco products for at least 6 months, where possible, or according to the definition used by the survey. When possible based on available survey questions, we restricted former smokers to only include individuals who had consumed at least 100 cigarette-equivalents in their lifetime.

Inclusion Criteria

We systematically reviewed surveys with information on tobacco use available in the Global Health Data Exchange (GHDx, http://ghdx.healthdata.org/). Surveys were extracted if they met our inclusion criteria, which were the following:

- Included indicators of tobacco use that met any of our standard case definitions, or alternatively, indicators that could be reliably adjusted to meet any of our standard case (see "adjusting for non-standard case-definitions" section)
- Representative of the general population of one of the 204 countries and territories included in our analysis
    - Excludes surveys conducted exclusively among sub-populations (including those with a specific disease, racial/ethnic minorities, pregnant women, etc.)
    - One exception is that school-based surveys were included for individuals in primary or secondary school. Most surveys covering tobacco use among youth rely on school-based data collection, resulting in a paucity of population-representative data. As a result, we chose to include school-based surveys in order to have broad data coverage for youth ages 10-17.
- Data collection occurred between January 1, 1980 and December 31, 2019
- Tobacco use was self-reported by the individual, not by a proxy respondent
- Respondents were ages 10 and above

Data extraction

We extracted primary data from individual-level microdata and survey report tabulations. We extracted data on current, former, and/or ever smoked tobacco use reported as any

combination of frequency of use (daily, occasional, and unspecified, which includes both daily and occasional smokers) and type of smoked tobacco used (all smoked tobacco, cigarettes, hookah, and other smoked tobacco products such as cigars or pipes), resulting in 36 possible combinations (3 time periods x 3 frequencies x 4 products). Other variants of tobacco products, for example hand-rolled cigarettes, were grouped into the four product categories listed above based on product similarities. Only smoked tobacco products are included - smoked drugs and smokeless tobacco are estimated separately as part of other risk factors. Due to the limited data available on both exposure to and the health effects from novel nicotine delivery systems, including e-cigarettes and heated tobacco products, these products were not included in GBD 2019.

For microdata, we extracted relevant demographic information, including age, sex, location, and year, as well as survey metadata, including survey weights, primary sampling units, and strata. This information allowed us to tabulate individual-level data in the standard GBD five-year age-sex groups and produce accurate estimates of uncertainty. When survey design indicators are not reported in the microdata, we assume a conservative design effect of 2.25. For survey report tabulations, we extracted data at the most granular age-sex group provided.

We estimated the prevalence of current smoking and the prevalence of former smoking using data from 3,625 cross-sectional nationally representative household surveys. These included both multinational and country-specific surveys. Figure SM1 depicts the number of data sources used for each country. Out of 204 countries and territories, 200 (98%) had at least one data source and 171 (84%) had at least five data sources. Ninety (44%) of countries had their most recent data source from either 2017 or 2018.

A complete list of data sources is available from the GBD 2019 Data Input Sources Tool: http://ghdx.healthdata.org/gbd-2019/data-input-sources.

Figure SM1. Number of data sources used to estimate current smoking prevalence, 1980-2019

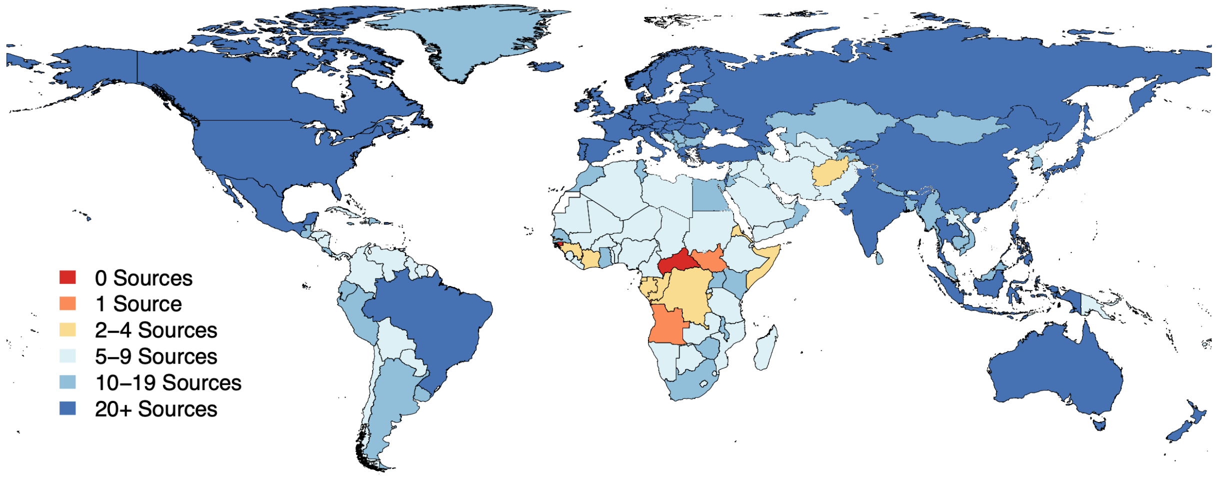

Adjustment for Non-Standard Case Definitions

Our GBD smoking case definitions were current use of any smoked tobacco product and former use of any smoked tobacco product. All other data points were adjusted to be consistent with these definitions. Some sources contained information on more than one case definition and these sources were used to develop the adjustment coefficients to transform alternative case definitions to the GBD standard case definitions. The adjustment coefficient was the beta value derived from the following linear regression model:

$$p_{gs,k} = \beta p_{alt,k} + \epsilon_k$$

where $p_{gs,k}$ is the prevalence based on the standard case definition in survey k and $p_{alt,k}$ is the prevalence based on an alternative case definition that is also reported in survey k.
Since smoking patterns vary by age, we fit separate models for adults over age 20, teens ages 15-19, and youth ages 10-14. Models for all combinations of case definitions were fit. Models with adjusted R-squared values > 0.8 were then ranked in order of their R-squared value. For surveys with multiple non-standard case definitions, the single coefficient from the most highly ranked model was used for adjustment.

In order to capture differences in smoking pattern by location, weights were assigned to data based on a hierarchy of geographic regions. When considering an adjustment for a country from region r, surveys used to fit the model from the same geographic region were given two times the weight of surveys outside that region, and a maximum of 200 of the geographically closest points were included in each model. We tested applying a similar weight based on time period, but found that it did not significantly change estimated coefficients. At minimum, 20

sources were used to fit crosswalk coefficients. Due to data limitations, we could only fit global models for youth adjustments.

We propagated uncertainty at the survey (k) level from the crosswalk using the following equation:

$$PE_k = \sigma_\epsilon^2 + X_k^2 \mathrm{var}(\hat{\beta})$$

where PE_k is the crosswalk prediction error that is added to the sampling variance of the data point, σ_ϵ^2 is the variance of the error, X_k^2 is the squared value of the data being adjusted, and $\mathrm{var}(\hat{\beta})$ is the variance of the adjustment coefficient.

Adjustment coefficients are reported for adults in Table SM2a, for teenagers in Table SM2b, and for youth in Table SM2c.

Table SM2a. Adult (Ages 20+) Adjustment Coefficients

**Super-Region**	**Region**	**Alternative Definition***	**R^2**	**β**	**$se(\hat{\beta})$**	**σ_ϵ^2**
Global	Global	cig_current_any	0.991	1.023	0.001	0.001
Global	Global	smoked_current_daily	0.912	1.099	0.002	0.009
Global	Global	cig_current_daily	0.911	1.135	0.002	0.008
Global	Global	cig_ever_any	0.843	0.578	0.003	0.015
Global	Global	cig_ever_daily	0.830	0.596	0.005	0.015
Central Europe, Eastern Europe, and Central Asia	Central Asia	cig_current_any	0.999	1.003	0.001	0.000
Central Europe, Eastern Europe, and Central Asia	Central Asia	smoked_current_daily	0.975	1.151	0.016	0.004
Central Europe, Eastern Europe, and Central Asia	Central Asia	cig_current_daily	0.974	1.154	0.016	0.004
Central Europe, Eastern Europe, and Central Asia	Central Asia	cig_ever_any	0.894	0.631	0.007	0.012
Central Europe, Eastern Europe, and Central Asia	Central Asia	cig_ever_daily	0.890	0.691	0.008	0.012
Central Europe, Eastern Europe, and Central Asia	Central Asia	smoked_ever_any	0.870	0.617	0.004	0.008
Central Europe, Eastern Europe, and Central Asia	Central Asia	smoked_ever_daily	0.859	0.697	0.009	0.017
Central Europe, Eastern Europe, and Central Asia	Central Europe	cig_current_any	0.998	1.011	0.001	0.000
Central Europe, Eastern Europe, and Central Asia	Central Europe	smoked_current_daily	0.918	1.132	0.010	0.011
Central Europe, Eastern Europe, and Central Asia	Central Europe	cig_current_daily	0.898	1.115	0.013	0.014
Central Europe, Eastern Europe, and Central Asia	Central Europe	cig_ever_any	0.891	0.641	0.009	0.012

Central Europe, Eastern Europe, and Central Asia	Central Europe	smoked_ever_daily	0.889	0.688	0.010	0.012
Central Europe, Eastern Europe, and Central Asia	Central Europe	cig_ever_daily	0.888	0.692	0.010	0.012
Central Europe, Eastern Europe, and Central Asia	Central Europe	smoked_ever_any	0.880	0.629	0.007	0.015
Central Europe, Eastern Europe, and Central Asia	Eastern Europe	cig_current_any	0.999	1.002	0.000	0.000
Central Europe, Eastern Europe, and Central Asia	Eastern Europe	smoked_current_daily	0.992	1.010	0.001	0.002
Central Europe, Eastern Europe, and Central Asia	Eastern Europe	cig_ever_any	0.901	0.608	0.014	0.012
Central Europe, Eastern Europe, and Central Asia	Eastern Europe	cig_ever_daily	0.894	0.689	0.016	0.013
Central Europe, Eastern Europe, and Central Asia	Eastern Europe	cig_current_daily	0.878	1.131	0.018	0.020
Central Europe, Eastern Europe, and Central Asia	Eastern Europe	smoked_ever_any	0.862	0.605	0.004	0.006
Central Europe, Eastern Europe, and Central Asia	Eastern Europe	smoked_ever_daily	0.854	0.761	0.017	0.019
High-income	Australasia	cig_current_daily	0.996	1.096	0.004	0.000
High-income	Australasia	smoked_current_daily	0.988	1.116	0.005	0.001
High-income	Australasia	cig_current_any	0.983	1.044	0.001	0.002
High-income	Australasia	cig_ever_any	0.837	0.561	0.003	0.016
High-income	Australasia	smoked_ever_daily	0.812	0.553	0.007	0.015
High-income	Australasia	cig_ever_daily	0.810	0.532	0.006	0.016
High-income	High-income Asia Pacific	cig_current_any	0.983	1.044	0.001	0.002
High-income	High-income Asia Pacific	smoked_current_daily	0.977	1.160	0.002	0.002
High-income	High-income Asia Pacific	cig_current_daily	0.961	1.146	0.002	0.004
High-income	High-income Asia Pacific	cig_ever_any	0.837	0.561	0.003	0.016
High-income	High-income Asia Pacific	smoked_ever_any	0.835	0.501	0.002	0.017
High-income	High-income Asia Pacific	smoked_ever_daily	0.812	0.553	0.007	0.015
High-income	High-income Asia Pacific	cig_ever_daily	0.810	0.532	0.006	0.016
High-income	High-income North America	smoked_current_daily	0.987	1.177	0.002	0.001
High-income	High-income North America	cig_current_any	0.969	1.071	0.003	0.003
High-income	High-income North America	cig_current_daily	0.954	1.255	0.004	0.005
High-income	High-income North America	smoked_ever_any	0.894	0.581	0.003	0.011
High-income	High-income North America	cig_ever_any	0.889	0.571	0.003	0.011
High-income	High-income North America	smoked_current_ltd	0.825	2.376	0.015	0.018
High-income	High-income North America	smoked_ever_daily	0.812	0.553	0.007	0.015
High-income	High-income North America	cig_ever_daily	0.810	0.532	0.006	0.016
High-income	Southern Latin America	cig_current_any	0.983	1.044	0.001	0.002
High-income	Southern Latin America	smoked_current_daily	0.977	1.160	0.002	0.002
High-income	Southern Latin America	cig_current_daily	0.961	1.146	0.002	0.004
High-income	Southern Latin America	cig_ever_any	0.837	0.561	0.003	0.016

High-income	Southern Latin America	smoked_ever_any	0.835	0.501	0.002	0.017
High-income	Southern Latin America	smoked_ever_daily	0.812	0.553	0.007	0.015
High-income	Southern Latin America	cig_ever_daily	0.810	0.532	0.006	0.016
High-income	Western Europe	cig_current_any	0.991	1.032	0.001	0.001
High-income	Western Europe	cig_current_daily	0.978	1.070	0.002	0.002
High-income	Western Europe	smoked_current_daily	0.960	1.140	0.004	0.004
High-income	Western Europe	smoked_ever_any	0.824	0.480	0.002	0.019
High-income	Western Europe	smoked_ever_daily	0.811	0.553	0.007	0.015
High-income	Western Europe	cig_ever_daily	0.810	0.532	0.006	0.016
Latin America and Caribbean	Andean Latin America	cig_current_any	0.992	1.032	0.002	0.000
Latin America and Caribbean	Andean Latin America	cig_current_daily	0.946	1.148	0.004	0.001
Latin America and Caribbean	Andean Latin America	smoked_current_daily	0.901	1.210	0.004	0.002
Latin America and Caribbean	Andean Latin America	cig_ever_any	0.848	0.501	0.021	0.009
Latin America and Caribbean	Andean Latin America	cig_ever_daily	0.843	0.739	0.032	0.010
Latin America and Caribbean	Caribbean	cig_current_any	0.980	1.076	0.012	0.001
Latin America and Caribbean	Caribbean	smoked_current_daily	0.901	1.210	0.004	0.002
Latin America and Caribbean	Caribbean	cig_current_daily	0.897	1.399	0.047	0.004
Latin America and Caribbean	Caribbean	cig_ever_any	0.848	0.501	0.021	0.009
Latin America and Caribbean	Caribbean	cig_ever_daily	0.843	0.739	0.032	0.010
Latin America and Caribbean	Central Latin America	cig_current_any	0.999	1.023	0.004	0.000
Latin America and Caribbean	Central Latin America	cig_current_daily	0.944	1.157	0.008	0.003
Latin America and Caribbean	Central Latin America	smoked_ever_any	0.863	0.503	0.004	0.008
Latin America and Caribbean	Central Latin America	smoked_current_daily	0.850	1.505	0.016	0.008
Latin America and Caribbean	Central Latin America	cig_ever_any	0.848	0.501	0.021	0.009
Latin America and Caribbean	Central Latin America	cig_ever_daily	0.843	0.739	0.032	0.010
Latin America and Caribbean	Tropical Latin America	cig_current_any	0.992	1.029	0.002	0.000
Latin America and Caribbean	Tropical Latin America	smoked_current_daily	0.967	1.116	0.002	0.001
Latin America and Caribbean	Tropical Latin America	cig_current_daily	0.955	1.125	0.004	0.001
Latin America and Caribbean	Tropical Latin America	cig_ever_any	0.848	0.501	0.021	0.009
Latin America and Caribbean	Tropical Latin America	cig_ever_daily	0.843	0.739	0.032	0.010
North Africa and Middle East	North Africa and Middle East	cig_current_any	0.989	1.030	0.006	0.002

North Africa and Middle East	North Africa and Middle East	smoked_ever_any	0.954	0.880	0.016	0.012
North Africa and Middle East	North Africa and Middle East	smoked_ever_ltd	0.952	1.029	0.035	0.027
North Africa and Middle East	North Africa and Middle East	cig_ever_daily	0.889	0.667	0.051	0.016
North Africa and Middle East	North Africa and Middle East	cig_ever_any	0.886	0.652	0.037	0.010
South Asia	South Asia	cig_current_any	0.994	1.019	0.002	0.000
South Asia	South Asia	smoked_ever_any	0.934	0.772	0.005	0.004
South Asia	South Asia	smoked_current_daily	0.929	1.182	0.008	0.006
South Asia	South Asia	cig_current_daily	0.890	1.225	0.013	0.010
Southeast Asia, East Asia, and Oceania	East Asia	smoked_ever_ltd	0.997	0.992	0.007	0.003
Southeast Asia, East Asia, and Oceania	East Asia	cig_current_any	0.996	1.009	0.002	0.001
Southeast Asia, East Asia, and Oceania	East Asia	smoked_ever_any	0.984	0.920	0.003	0.003
Southeast Asia, East Asia, and Oceania	East Asia	smoked_current_daily	0.970	1.094	0.004	0.005
Southeast Asia, East Asia, and Oceania	East Asia	cig_current_daily	0.963	1.014	0.005	0.007
Southeast Asia, East Asia, and Oceania	East Asia	cig_current_ltd	0.908	1.038	0.044	0.046
Southeast Asia, East Asia, and Oceania	Oceania	smoked_ever_ltd	0.997	0.992	0.007	0.003
Southeast Asia, East Asia, and Oceania	Oceania	cig_current_any	0.996	1.009	0.002	0.001
Southeast Asia, East Asia, and Oceania	Oceania	smoked_ever_any	0.971	0.909	0.004	0.006
Southeast Asia, East Asia, and Oceania	Oceania	smoked_current_daily	0.935	1.211	0.024	0.011
Southeast Asia, East Asia, and Oceania	Oceania	cig_current_ltd	0.908	1.038	0.044	0.046
Southeast Asia, East Asia, and Oceania	Oceania	cig_current_daily	0.866	1.067	0.009	0.027
Southeast Asia, East Asia, and Oceania	Southeast Asia	smoked_ever_ltd	0.997	0.992	0.007	0.003
Southeast Asia, East Asia, and Oceania	Southeast Asia	cig_current_any	0.996	1.008	0.002	0.001
Southeast Asia, East Asia, and Oceania	Southeast Asia	smoked_ever_any	0.971	0.909	0.004	0.006
Southeast Asia, East Asia, and Oceania	Southeast Asia	cig_current_ltd	0.908	1.038	0.044	0.046
Sub-Saharan Africa	Central Sub-Saharan Africa	cig_ever_any	0.991	0.829	0.016	0.001
Sub-Saharan Africa	Central Sub-Saharan Africa	cig_current_any	0.987	1.013	0.003	0.001
Sub-Saharan Africa	Central Sub-Saharan Africa	smoked_ever_ltd	0.951	1.024	0.029	0.007
Sub-Saharan Africa	Central Sub-Saharan Africa	smoked_ever_any	0.933	0.860	0.021	0.008
Sub-Saharan Africa	Central Sub-Saharan Africa	smoked_current_daily	0.903	1.066	0.011	0.010
Sub-Saharan Africa	Central Sub-Saharan Africa	cig_current_ltd	0.894	1.042	0.032	0.014
Sub-Saharan Africa	Central Sub-Saharan Africa	cig_current_daily	0.863	1.076	0.013	0.015

Sub-Saharan Africa	Central Sub-Saharan Africa	smoked_ever_daily	0.814	0.759	0.022	0.009
Sub-Saharan Africa	Eastern Sub-Saharan Africa	smoked_current_daily	0.991	1.023	0.005	0.002
Sub-Saharan Africa	Eastern Sub-Saharan Africa	cig_ever_any	0.991	0.829	0.016	0.001
Sub-Saharan Africa	Eastern Sub-Saharan Africa	cig_current_any	0.983	1.012	0.005	0.002
Sub-Saharan Africa	Eastern Sub-Saharan Africa	cig_current_daily	0.961	1.027	0.012	0.008
Sub-Saharan Africa	Eastern Sub-Saharan Africa	smoked_ever_ltd	0.951	1.024	0.029	0.007
Sub-Saharan Africa	Eastern Sub-Saharan Africa	smoked_ever_any	0.933	0.860	0.021	0.008
Sub-Saharan Africa	Eastern Sub-Saharan Africa	cig_current_ltd	0.894	1.042	0.032	0.014
Sub-Saharan Africa	Eastern Sub-Saharan Africa	smoked_ever_daily	0.814	0.759	0.022	0.009
Sub-Saharan Africa	Southern Sub-Saharan Africa	cig_current_any	0.996	1.029	0.005	0.000
Sub-Saharan Africa	Southern Sub-Saharan Africa	cig_ever_any	0.991	0.829	0.016	0.001
Sub-Saharan Africa	Southern Sub-Saharan Africa	smoked_current_daily	0.976	1.206	0.013	0.001
Sub-Saharan Africa	Southern Sub-Saharan Africa	smoked_ever_ltd	0.951	1.024	0.029	0.007
Sub-Saharan Africa	Southern Sub-Saharan Africa	cig_current_daily	0.933	1.111	0.014	0.005
Sub-Saharan Africa	Southern Sub-Saharan Africa	smoked_ever_any	0.933	0.860	0.021	0.008
Sub-Saharan Africa	Southern Sub-Saharan Africa	cig_current_ltd	0.894	1.042	0.032	0.014
Sub-Saharan Africa	Southern Sub-Saharan Africa	smoked_ever_daily	0.814	0.759	0.022	0.009
Sub-Saharan Africa	Western Sub-Saharan Africa	cig_current_any	0.997	1.007	0.003	0.000
Sub-Saharan Africa	Western Sub-Saharan Africa	cig_ever_any	0.991	0.829	0.016	0.001
Sub-Saharan Africa	Western Sub-Saharan Africa	smoked_ever_ltd	0.951	1.024	0.029	0.007
Sub-Saharan Africa	Western Sub-Saharan Africa	smoked_ever_any	0.933	0.860	0.021	0.008
Sub-Saharan Africa	Western Sub-Saharan Africa	cig_current_ltd	0.894	1.042	0.032	0.014

Table SM2b. Teenager (Ages 15-19) Adjustment Coefficients

Super-Region	Region	Alternative Definition	R^2	β	$se(\hat{\beta})$	σ_ϵ^2
Global	Global	cig_current_any_var	0.992	1.024	0.002	0.000
Global	Global	cig_ever_any_var	0.932	0.759	0.008	0.005
Global	Global	cig_ever_daily_var	0.927	0.918	0.017	0.005
Global	Global	smoked_ever_any_var	0.870	0.600	0.004	0.008
Global	Global	cig_current_daily_var	0.817	1.237	0.013	0.011

Table SM2c. Youth (Ages 10-14) Adjustment Coefficients

Super-Region	Region	Alternative Definition	R^2	β	$se(\hat{\beta})$	σ_ϵ^2
Global	Global	hookah_ever_any_var	0.977	0.835	0.026	0.001
Global	Global	cig_current_any_var	0.959	1.139	0.011	0.002
Global	Global	hookah_current_ltd_var	0.932	1.695	0.076	0.003
Global	Global	hookah_current_any_var	0.928	1.601	0.071	0.002
Global	Global	hookah_former_any_var	0.892	1.255	0.089	0.004
Global	Global	othsmk_current_ltd_var	0.877	2.663	0.131	0.005

Global	Global	cig_ever_any_var	0.863	0.472	0.015	0.002
Global	Global	smoked_ever_any_var	0.859	0.393	0.005	0.002
Global	Global	smoked_ever_ltd_var	0.842	1.030	0.095	0.004
Global	Global	chew_current_ltd_var	0.812	0.629	0.042	0.004

* The alternative definition is based on a combination of four smoked tobacco product types, three frequencies of use, and three time periods of use. The four product types are: cigarettes (cig), all smoked tobacco (smoked), hookah/shisha (hookah), and pipes, cigars, and other local smoked tobacco products (othsmk). The three frequencies of use are daily, occasionally (ltd), and any. The three time periods of use are current, former, and ever.

Age and Sex Splitting

We split data reported in broader age groups than the GBD 5-year age groups or as both sexes combined by adapting the method reported in Ng et al. (http://jamanetwork.com/journals/jama/fullarticle/1812960) to split using a location- time-specific reference age pattern. We separated the data into two sets: a training dataset, with data already falling into GBD sex-specific 5-year age groups, and a split dataset, which reported data in aggregated age or sex groups. We then used spatiotemporal Gaussian Process Regression (ST-GPR) to estimate sex-location-time specific age-sex patterns using data in the training dataset. The estimated age-sex patterns were used to split each source in the split dataset using the following equation:

$$\tilde{p}_{a,c,s} = \frac{N^{a+x}_{a,c,s,k}}{Pop_{a,c,s}} \left(\frac{R_{a,s}}{R^{a+x}_{a,s}} \right)$$

$N^{a+x}_{a,s,c,}$: number of smokers reported in survey k, for country c, sex s, and an age group spanning a to $a + x$.

$R_{a,s}$: total number of smokers estimated for the target five-year age group a.

$R^{a+x}_{a,s}$: total number of smokers estimated for the age groups spanning a to $a + x$.

$Pop_{a,c,s}$: the population of the target five-year age group a, for country c, and sex s.

The ST-GPR model (model described in the "Smoking Prevalence Modeling" section below) used to estimate the age patterns for age-sex splitting ($\frac{R_{a,s}}{R^{a+x}_{a,s}}$) used an age weight parameter value that minimises the effect of any age smoothing. This parameter choice allows the estimated age pattern to be driven by data, rather than being enforced by any smoothing parameters of the model. Because these age-sex split data points will be incorporated in the final ST-GPR exposure model, we do not want to doubly enforce a modelled age pattern for a given sex-location-year on a given aggregate data point.

Two main advantages of the current approach, compared to that published in Ng. et al., are that the age-sex pattern is allowed to vary by country and year, and also that uncertainty from the age-sex pattern used to split data is captured and propagated. By adding additional uncertainty to the data (based on 100 draws of the estimated age-sex pattern), data points that require age-sex splitting have less influence on the final modeled estimates compared to data points that do not require splitting.

Smoking Prevalence Modelling

We used spatiotemporal Gaussian process regression (ST-GPR) to model current and former smoking prevalence. Full details on the ST-GPR method are reported elsewhere. Briefly, the mean function input to GPR is a complete time series of estimates generated from a mixed effects hierarchical linear model plus weighted residuals smoothed across time, space and age. The linear model formula for current smoking, fit separately by sex using restricted maximum likelihood in R, is:

$$logit(p_{l,a,t}) = \beta_0 + \beta_1 CPC_{l,t} + \sum_{k=2}^{19} \beta_k I_{A[a]} + \alpha_s + \alpha_r + \alpha_l + \epsilon_{l,a,t}$$

Where $CPC_{g,t}$ is the tobacco consumption covariate by location l and time t, described above, $I_{A[a]}$ is a dummy variable indicating specific age group A that the prevalence point $p_{g,a,t}$ captures, and α_s, α_r, and α_l are super region, region, and location random intercepts, respectively. Random effects were used in model fitting but not in prediction.

The linear model formula for former smoking is:

$$logit(p_{l,a,t}) = \beta_0 + \beta_1 PctChange_{A[a],l,t} + \beta_3 CSP_{A[a],l,t} + \sum_{k=3}^{20} \beta_k I_{A[a]} + \alpha_s + \alpha_r + \alpha_l + \epsilon_{l,a,t}$$

Where $PctChange_{A[a],l,t}$ is the percent change in current smoking prevalence from the previous year, and $CSP_{A[a],l,t}$ is the current smoking prevalence by specific age group A, location l, and time t that point $p_{l,a,t}$ captures, both derived from the current smoking ST-GPR model defined above.

The model hyperparameters were used as follows:

Data Category	Lambda	Omega	Zeta	Scale
**<5 Sources**	0.04	0.87	0.01	20
**5-9 Sources**	0.04	1.48	0.01	20
**10-19 Sources**	0.04	1.48	0.005	20
**20+ Sources**	0.04	1.84	0.005	20

These were based on a previous 10-fold out-of-sample cross-validation of the smoking prevalence model, where data sources were randomly held out and the model parameters that

minimized root mean squared error by data density category were selected. Due to the computationally intensive nature of this model parameter selection process, data density categories were used to apply appropriate parameters to all models utilized for final attributable burden estimation.

Dose-Response Exposure Among Current and Former Smokers

We estimated exposure among current smokers for two continuous indicators: cigarette-equivalents per smoker per day and pack-years. Pack-years incorporates aspects of both duration and amount. One pack-year represents the equivalent of smoking one pack of cigarettes (assuming a 20-cigarette pack) per day for one year. Since the pack-years indicator collapses duration and intensity into a single dimension, one pack-year of exposure can reflect smoking 40 cigarettes per day for six months or smoking 10 cigarettes per day for two years.

To produce these indicators, we simulated individual smoking histories based on distributions of age of initiation and amount smoked. We informed the simulation with cross-sectional survey data capturing these indicators, modelled at the mean level for all locations, years, ages, and sexes using spatiotemporal Gaussian process regression. We chose not to include a time-varying covariate in the linear model for these indicators, instead allowing the spatiotemporal smoothing step to capture observed trends in the data. We predicted distribution standard deviation based on the predicted mean estimates and a linear regression model with effects on age, sex, year, and location. This model was selected based on out-of-sample RMSE.

Supply-Side Consumption

In producing estimates of mean cigarette-equivalents per smoker per day, we split estimated supply-side consumption using age-sex specific estimates of consumption from survey data. The supply-side data provide a more complete and objective time-series compared to survey data, and therefore is preferable to include when setting the level of consumption in a country. We included data from UN FAO (1961-2013, domestic supply), United States Department of Agriculture (1960-2005, domestic supply), and Euromonitor (2002-2017, retail supply) to estimate the supply-side envelope, as measured in cigarette-equivalents per capita (ages 15+). Domestic supply is calculated as:

$$supply - side\ consumption = production + imports - exports$$

The supply-side data go through three rounds of outliering. First, they are age-sex split using daily smoking prevalence to generate number of cigarette-equivalents per smoker per day for a given location-age-sex-year. If more than 12 points for a particular source-location-year (equal

to over 1/3 of the split points) are above the given thresholds, that source-location-year is outliered. A point would not be outliered if it was (in cigarette-equivalents per smoker): under 5 (10-14 year olds); under 20 (males, 15-19 year olds); under 18 (females, 15-19 year olds); under 38/35 and over 3 (males/females, 20+ year olds). These thresholds were chosen by visualizing histograms of the data for each age-sex, as well as with expert knowledge about feasible consumption levels. In the second round of outliering, the mean tobacco per capita value over a 10-year window is calculated. If a point is over 70% of that mean value away from the mean value, it is outliered. The 70% limit was chosen using histograms of these distances. Additionally, some manual outliering is performed to account for edge cases. Finally, data smoothing is performed by taking a 3-year rolling mean over each location-year.

Next, an imputation to fill in missing years is performed for all series to remove compositional bias from our final estimates. Since the data from our main sources cover different time periods, by imputing a complete time series for each data series, we reduce the probability that compositional bias of the sources is leading to biased final estimates. To impute the missing years for each series, we model the log ratio of each pair of sources as a function of an intercept and nested random effects on super region, region, and location. The appropriate predicted ratio is multiplied by each source that we do have, and then the predictions are averaged to get the final imputed value. For example, if source A is missing for a particular location-year, but sources B and C are present, then we predicted A twice: once from the modelled ratio of A to B, and again from the modelled ratio of A to C. These two predictions are then averaged. For some locations where there was limited overlap between series, the predicted ratio did not make sense, and a regional ratio was used.

Finally, variance was calculated both across series (within a location-year) as well as across years (within a location-source). Additionally, if a location-year had one imputed point was, the variance was multiplied by 2. If a location-year had two imputed points, the variance was multiplied by 4. The average estimates in each location-year were the input to an ST-GPR model. This uses a simple mixed effects model modelled in log space with nested location random effects.

We then use the estimated age-sex pattern of cigarette-equivalent consumption to split the supply-side consumption estimates available by country and year. This rescaling is performed using the equations below:

$$Proportion\ of\ total\ cigarette\ consumption\ l, y, s, a = \frac{Cigarette\ consumption\ l, y, s, a * population\ l, y, s, a * \%\ current\ smokers\ l, y, s, a}{\Sigma s, a(Cigarette\ consumption\ l, y, s, a * population\ l, y, s, a * \%\ current\ smokers\ l, y, s, a)}$$

$$Cigarette\ Consumption l,y,s,a = \frac{Supply - Side\ Consumption\ l,y * population\ l,y,s,a * proportion\ of\ total\ consumption\ l,y,s,a}{\%\ current\ smokers l,y,s,a * population\ l,y,s,a}$$

Final estimates of cigarette-equivalents per smoker per day used in estimating smoking attributable burden are an average of estimates from household surveys and estimates from supply-side data.

We estimated pack-years of exposure by summing samples from age- and time-specific distributions of cigarette-equivalents per smoker for a birth cohort in order to capture both age trends and time trends and avoid the common assumption that the amount someone currently smokes is the amount they have smoked since they began smoking. A sample of individuals started smoking at a given age based on an estimated distribution of age of initiating regular smoking. By five-year increment samples were drawn from estimates of cigarette-equivalents per smoker following a birth cohort to re-construct a smoking history. Two important limitations of this approach are: 1) individuals are assumed to not have quit for any period since they initiated smoking, and 2) the samples of cigarette-equivalents/day were independently drawn without introducing any correlation.

Ensemble Distributions

Distributions were age-, sex-, and region- specific ensemble distributions, which were found to outperform any single distribution.

After modeling mean age of initiation and mean cigarette-equivalents per smoker per day, we proceeded to estimate the full distributions of each indicator for every country, year, age group, and sex. We utilized paired data on mean and standard deviation from surveys to fit a linear regression that predicts standard deviation from mean:

$$\log(SD) = \beta_0 + \beta_1 \log(MEAN) + \beta_2 sex + \sum \beta_i age_i + \alpha + \varepsilon$$

Where the logarithm of mean, sex, and age group dummy variables are predictors, along with a nested random intercept (α) at three levels of geographic granularity (super-region, region, and country).

We predicted an estimate of the standard deviation for each of the 1,000 draws of the mean, by location, year, age, and sex. Together, these two parameters characterized the distributions. We used an ensemble approach to construct a probability density function for each indicator. Instead of using a single distribution, we used a linear combination eleven candidate

distributions (beta, exponential, gamma, inverse gamma, mirror gamma, gumbel, mirror gumbel, log-logistic, lognormal, normal, and Weibull), with weights optimized to minimize the Kolmogorov-Smirnov test statistic. Weights were optimized using individual-level data by region, age group, and sex. This prevented overfitting to a single dataset, while allowing for the shape of the distribution to vary by demographic group. Using the estimated mean, standard deviation, and ensemble weights, we were able to construct probability densities and cumulative distribution functions of each indicator for every location, year, age, and sex.

Figure SM2 shows an example ensemble distribution.

We estimated exposure among former smokers using years since cessation. We utilized spatiotemporal Gaussian process regression to model mean age of cessation using cross-sectional survey data capturing age of cessation. Using these estimates, we generated ensemble distributions of years since cessation for every location, year, age group, and sex.

Figure SM2. Example Ensemble Distribution

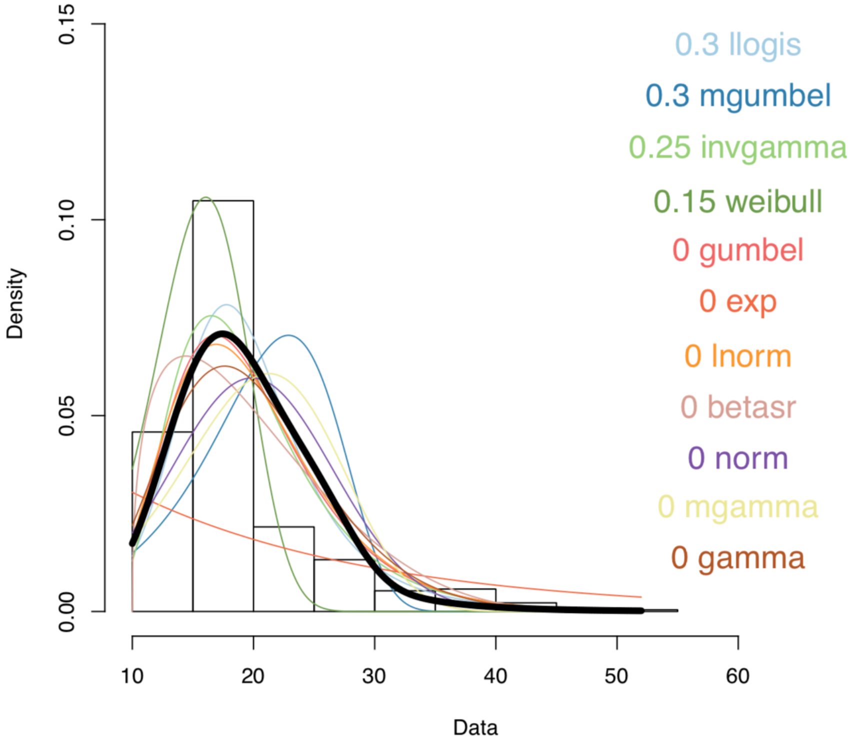

Risk-Outcome Pairs

We included the following risk-outcome pairs based on evidence supporting a causal relationship: tuberculosis, lower respiratory tract infections, esophageal cancer, stomach cancer, bladder cancer, liver cancer, laryngeal cancer, lung cancer, breast cancer, cervical

cancer, colorectal cancer, lip and oral cancer, nasopharyngeal cancer, other pharyngeal cancer, pancreatic cancer, kidney cancer, leukemia, ischemic heart disease, stroke, atrial fibrillation and flutter, aortic aneurysm, peripheral arterial disease, chronic obstructive pulmonary disease, other chronic respiratory diseases, asthma, peptic ulcer disease, gallbladder and biliary tract diseases, Alzheimer disease and other dementias, Parkinson disease (protective), multiple sclerosis, type-II diabetes, rheumatoid arthritis, low back pain, cataracts, macular degeneration, and fracture.

Dose-response risk curves

We conducted systematic literature reviews for all risk-outcome pairs identified as being caused by smoking. PRISMA charts depict the review process for each risk-outcome pair (supplemental folder). We extracted effect sizes (hazard ratios, relative risks, or odds ratios) by cigarette-equivalents per smoker per day, pack-years, and years since quitting from cohort and case-control studies. We synthesized these data to produce non-linear dose response curves using DisMod ODE as a Bayesian meta-regression model. For outcomes with significant differences in effect size by sex or age, we produced sex- or age-specific risk curves.

We estimate risk curves of former smokers compared to never smokers taking into account the rate of risk reduction among former smokers seen in the cohort and case-control studies, and the cumulative exposure among former smokers within each age, sex, location and year group. Risk reduction data were rescaled to start from a relative risk value for former smokers compared to never smokers of 10. Figure SM3 shows an example rescaling.

Figure SM3. Rescaled estimates for lung cancer risk reduction from Thun 2013 CPS-I data

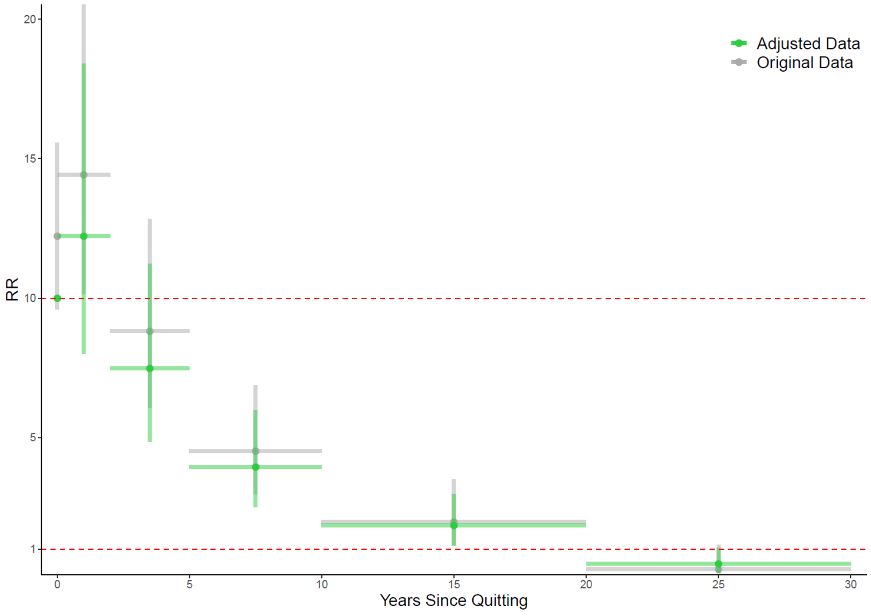

In Figure SM3, the crosses represent data points, where the vertical bars cover the 95% reported confidence interval, the horizontal bars cover the years-since-quitting range, and the midpoint represents the mean RR and years-since-quitting midpoint. One can see that the starting RR in the original data is now fixed at 10 after the transformation, while the RRs for various cessation categories have been adjusted to preserve the rate of risk reduction. This adjustment is necessary to avoid compositional bias due to the variety of smoking histories from different populations included in the meta-regression. One important limitation of this approach is the assumption of a constant rate of risk reduction regardless of starting relative risk. Future work should explore using the relative risk at years since quitting = 0 as a predictor of the rate of risk reduction.

In applying the risk-reduction curves, we again adjusted the originally fit risk-reduction model, which had been rescaled to start at $RR(time_0) = 10$, to have:

$$RR(time_0) = \int \exp(x) * rr(x)$$

Where (x) is the exposure among current smokers in a given location, year, age, and sex. In other words, the RR at the moment of quitting is equivalent to the average relative risk among current smokers in that population. This approach is necessary to not overestimate the relative risk among former smokers in populations that have less intense smoking histories, particularly critical due to the fact that most cohort and case-control studies on the health effects of

smoking have been conducted in high-income countries or populations with intense historical smoking epidemics.

PAF Calculation

We estimated population attributable fractions based on the following equation:

$$PAF = \frac{p(n) + p(f)\int \exp(x) * rr(x) + p(c)\int \exp(y) * rr(y) - 1}{p(n) + p(f)\int \exp(x) * rr(x) + p(c)\int \exp(y) * rr(y)}$$

where $p(n)$ is the prevalence of never smokers, $p(f)$ is the prevalence of former smokers, $p(c)$ is the prevalence of current smokers, $\exp(x)$ is a distribution of years since quitting among former smokers, $rr(x)$ is the relative risk for years since quitting, $\exp(y)$ is a distribution of cigarette-equivalents per smoker per day or pack-years, and $rr(y)$ is the relative risk for cigarette-equivalents per smoker per day or pack-years.

We used pack-years as the exposure definition for cancers and chronic respiratory diseases, and cigarette-equivalents per smoker per day for cardiovascular diseases and all other health outcomes. There is wide variation in smoking patterns across countries, demographic groups, and time periods. For cancers and chronic respiratory diseases, which are predominantly due to long-term smoking, using pack-years allows estimates to reflect both the duration of exposure and dose of exposure (cigarette-equivalents per day). For cardiovascular disease and all other health outcomes, cigarette-equivalents per smoker per day allows our methods to reflect dose of exposure, which is generally thought to be more important than duration of exposure for these health outcomes.

Exposures were lagged based on the weighted-average length of follow-up of studies included in meta-regressions, rounded to the nearest five-year increment.

Analytic Code

Analytic code are available at: https://github.com/ihmeuw/ihme-modeling/tree/master/gbd_2019/risk_factors_code/smoking_direct

GATHER Checklist

Item #	Checklist item	Reported on page #
**Objectives and funding**		
**1**	Define the indicator(s), populations (including age, sex, and geographic entities), and time period(s) for which estimates were made.	Main Pg. 5, Appendix Pg. 9
**2**	List the funding sources for the work.	Main Pg. 1
**Data Inputs**		
For all data inputs from multiple sources that are synthesized as part of the study:		
**3**	Describe how the data were identified and how the data were accessed.	Appendix pg. 11
**4**	Specify the inclusion and exclusion criteria. Identify all ad-hoc exclusions.	Appendix pg. 11
**5**	Provide information on all included data sources and their main characteristics. For each data source used, report reference information or contact name/institution, population represented, data collection method, year(s) of data collection, sex and age range, diagnostic criteria or measurement method, and sample size, as relevant.	http://ghdx.healthdata.org/gbd-2019/data-input-sources
**6**	Identify and describe any categories of input data that have potentially important biases (e.g., based on characteristics listed in item 5).	http://ghdx.healthdata.org/gbd-2019/data-input-sources
For data inputs that contribute to the analysis but were not synthesized as part of the study:		
**7**	Describe and give sources for any other data inputs.	N/A
For all data inputs:		
**8**	Provide all data inputs in a file format from which data can be efficiently extracted (e.g., a spreadsheet rather than a PDF), including all relevant meta-data listed in item 5. For any data inputs that cannot be shared because of ethical or legal reasons, such as third-party ownership, provide a contact name or the name of the institution that retains the right to the data.	http://ghdx.healthdata.org/gbd-2019/data-input-sources
**Data analysis**		
**9**	Provide a conceptual overview of the data analysis method. A diagram may be helpful.	Appendix Pg. 10
**10**	Provide a detailed description of all steps of the analysis, including mathematical formulae. This description should cover, as relevant, data cleaning, data pre-processing,	Appendix Pg. 11-28

	data adjustments and weighting of data sources, and mathematical or statistical model(s).	
**11**	Describe how candidate models were evaluated and how the final model(s) were selected.	Appendix Pg. 11-28
**12**	Provide the results of an evaluation of model performance, if done, as well as the results of any relevant sensitivity analysis.	N/A
**13**	Describe methods for calculating uncertainty of the estimates. State which sources of uncertainty were, and were not, accounted for in the uncertainty analysis.	Main Pg. 3
**14**	State how analytic or statistical source code used to generate estimates can be accessed.	Appendix Pg. 28 (https://github.com/ihmeuw/ihme-modeling/tree/master/gbd_2019/risk_factors_code/smoking_direct)
**Results and Discussion**		
**15**	Provide published estimates in a file format from which data can be efficiently extracted.	http://ghdx.healthdata.org/gbd-results-tool
**16**	Report a quantitative measure of the uncertainty of the estimates (e.g. uncertainty intervals).	Main Pg. 4-14
**17**	Interpret results in light of existing evidence. If updating a previous set of estimates, describe the reasons for changes in estimates.	Main Pg. 2
**18**	Discuss limitations of the estimates. Include a discussion of any modelling assumptions or data limitations that affect interpretation of the estimates.	Main Pg. 15-16

PRISMA Flowcharts

Atrial Fibrillation and Flutter

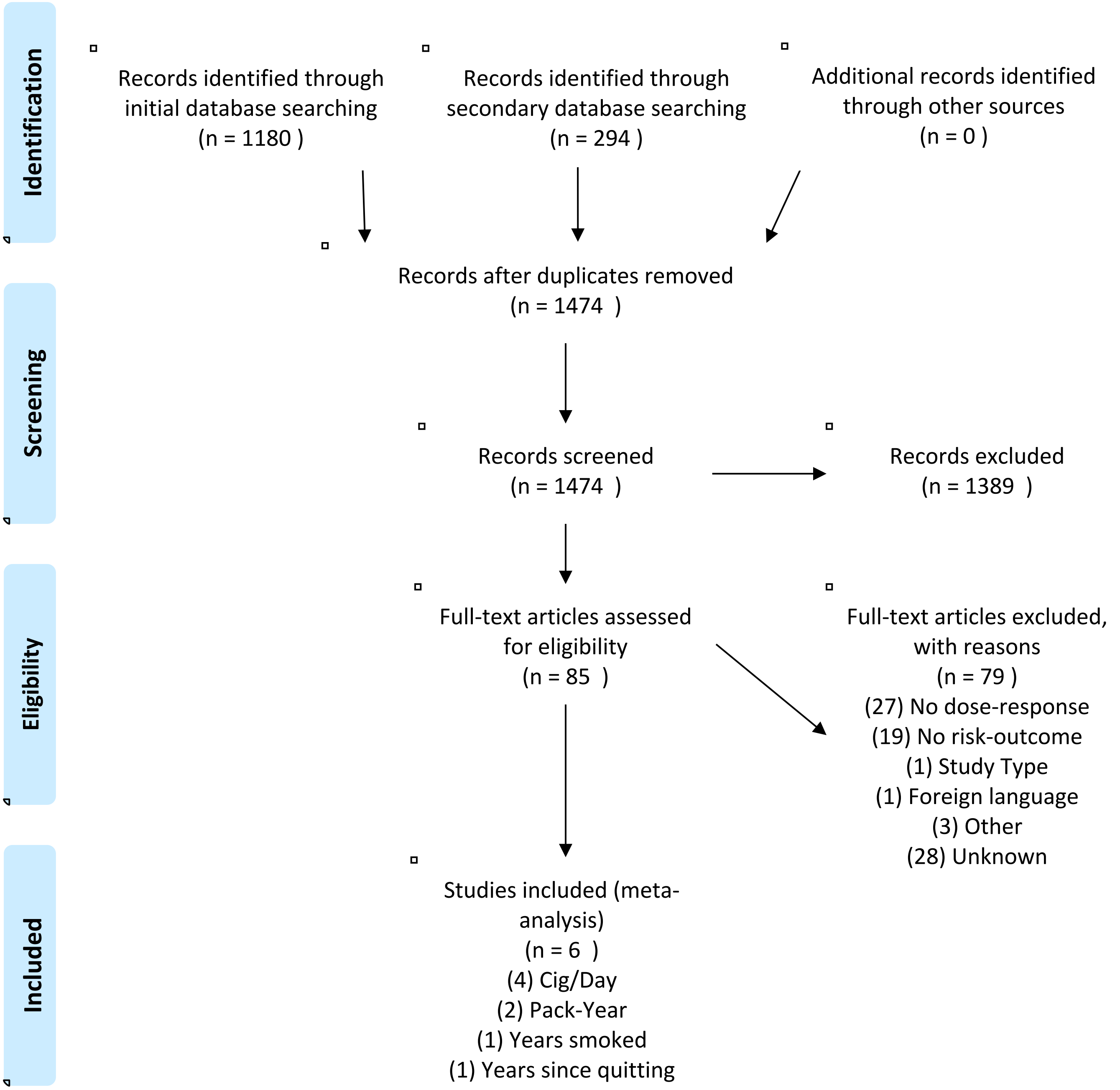

From: Moher D, Liberati A, Tetzlaff J, Altman DG, The PRISMA Group (2009). *P*referred *R*eporting *I*tems for *S*ystematic Reviews and *M*eta-*A*nalyses: The PRISMA Statement. PLoS Med 6(7): e1000097. doi:10.1371/journal.pmed1000097

For more information, visit www.prisma-statement.org.

Alzheimer’s Disease and Other Dementias

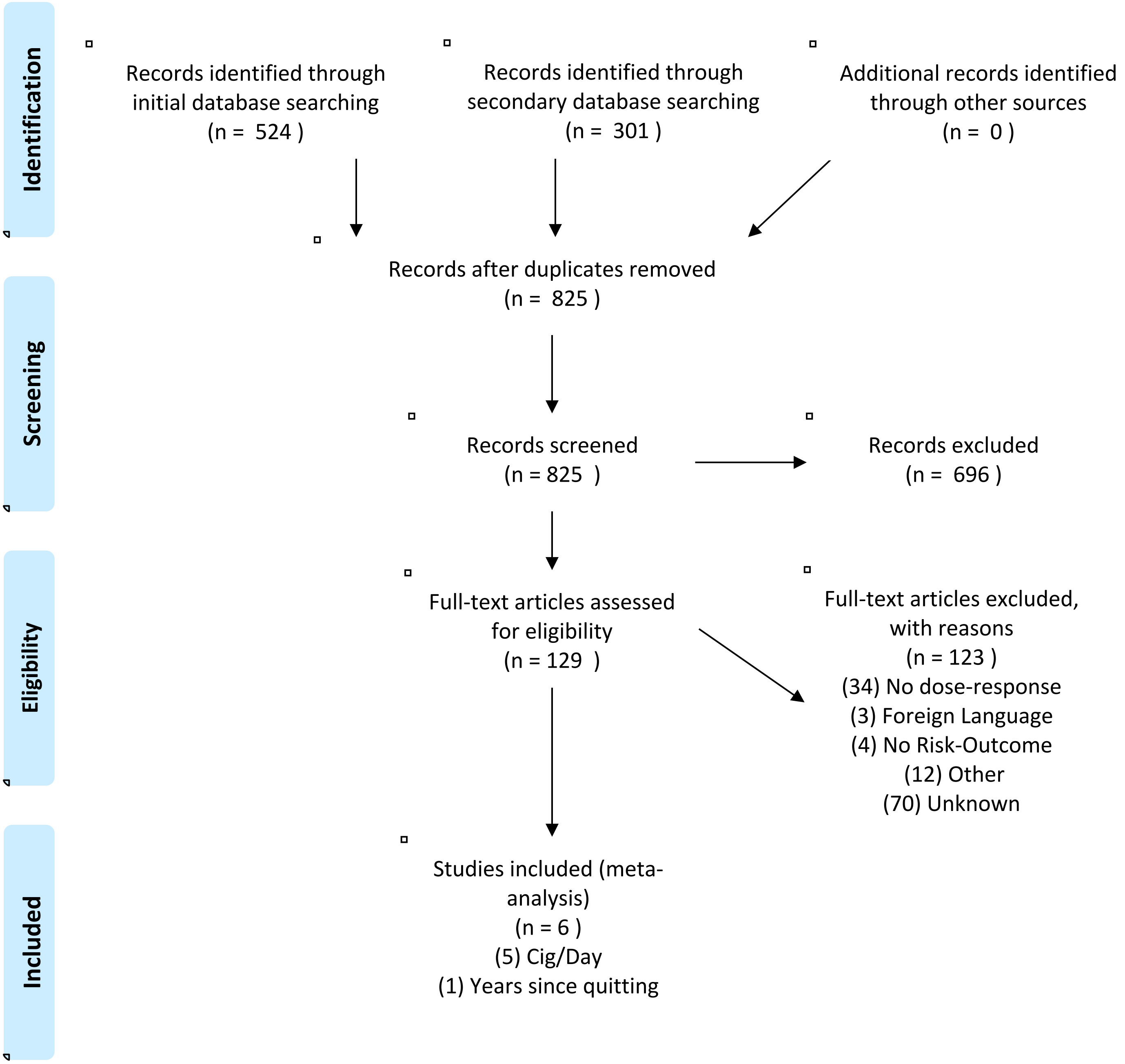

From: Moher D, Liberati A, Tetzlaff J, Altman DG, The PRISMA Group (2009). *P*referred *R*eporting *I*tems for *S*ystematic Reviews and *M*eta-*A*nalyses: The PRISMA Statement. PLoS Med 6(7): e1000097. doi:10.1371/journal.pmed1000097

For more information, visit www.prisma-statement.org.

Aortic Aneurysm

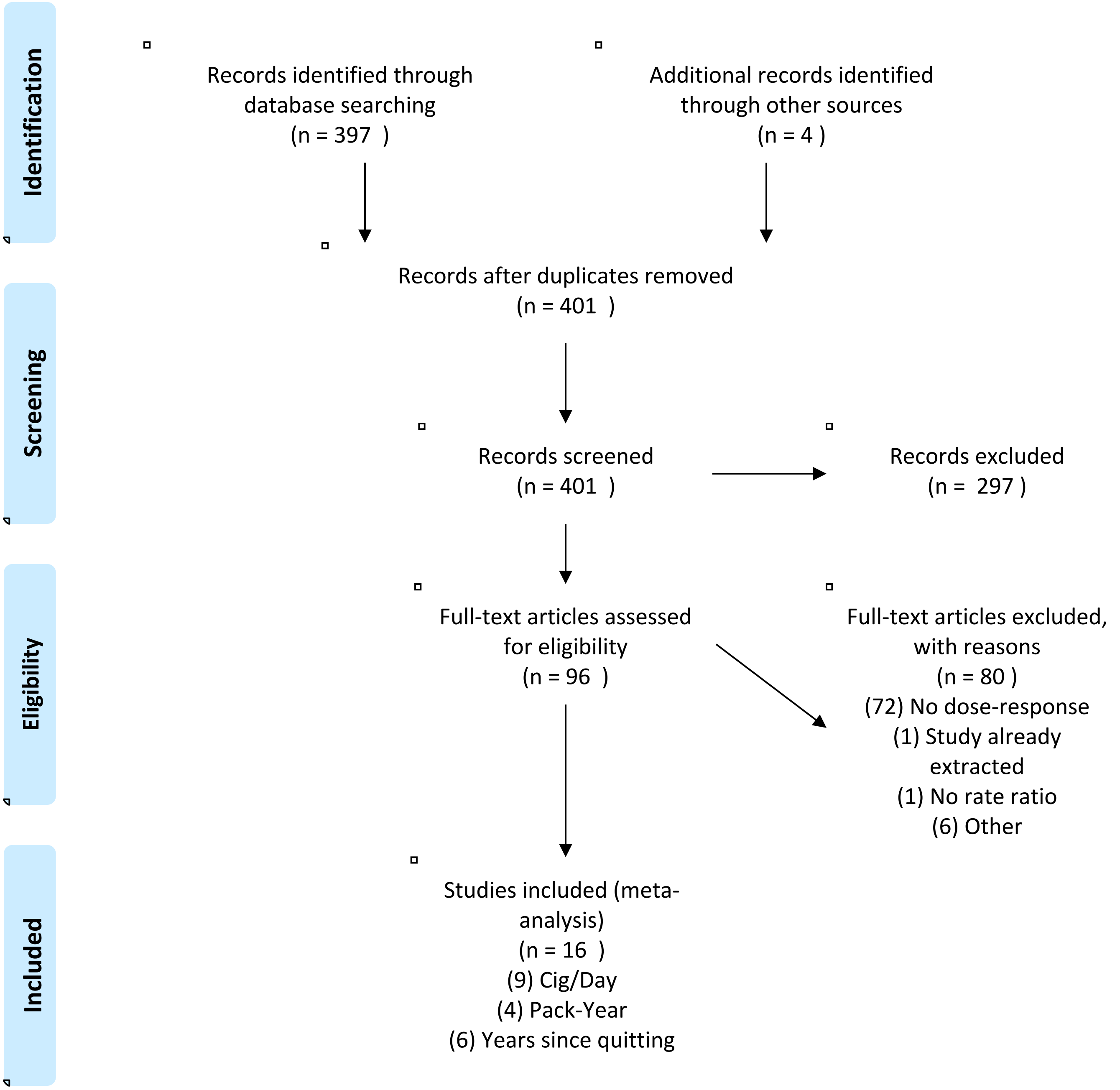

From: Moher D, Liberati A, Tetzlaff J, Altman DG, The PRISMA Group (2009). *P*referred *R*eporting *I*tems for *S*ystematic Reviews and *M*eta-*A*nalyses: The PRISMA Statement. PLoS Med 6(7): e1000097. doi:10.1371/journal.pmed1000097

For more information, visit www.prisma-statement.org.

Asthma

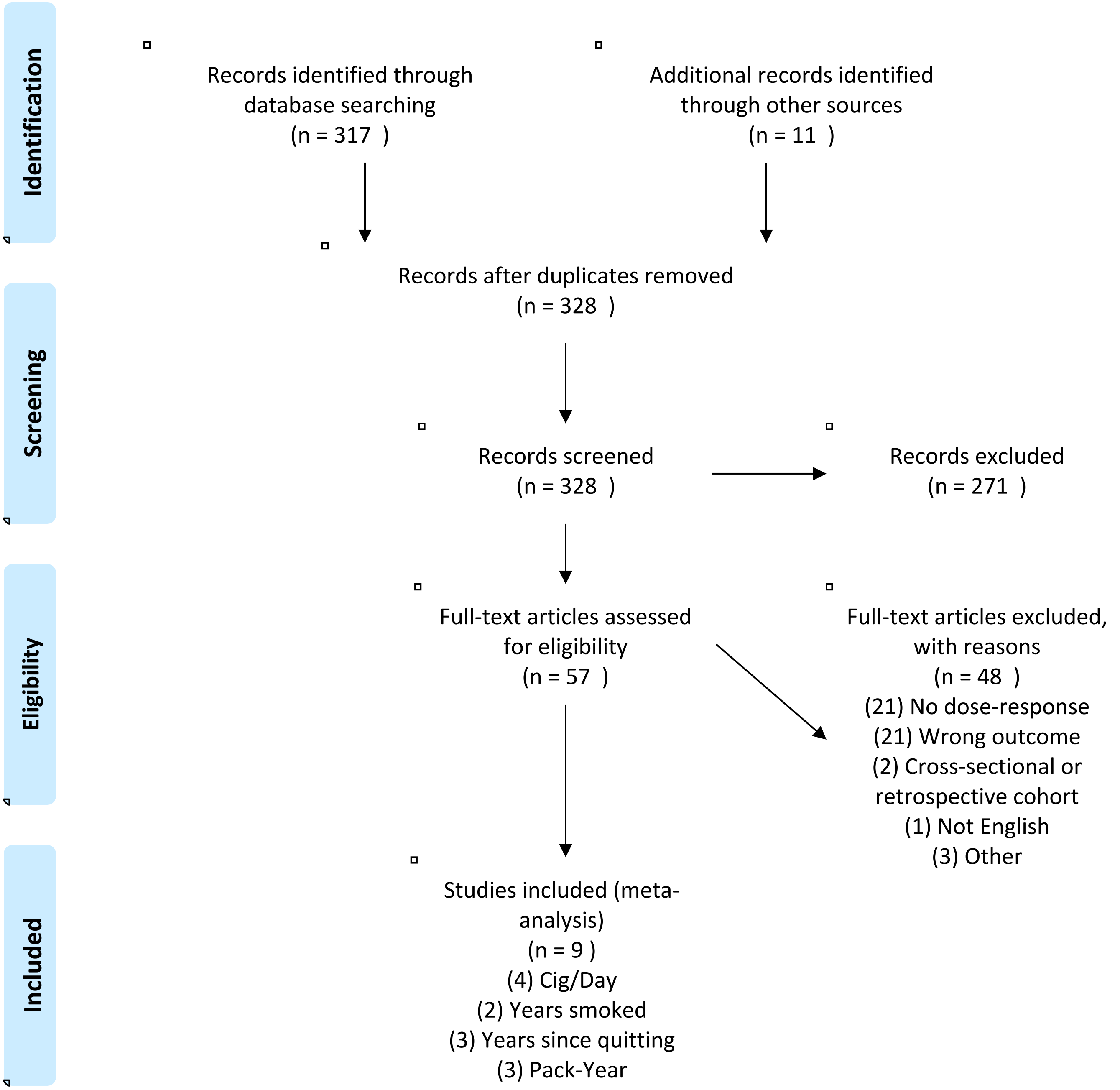

From: Moher D, Liberati A, Tetzlaff J, Altman DG, The PRISMA Group (2009). *P*referred *R*eporting *I*tems for *S*ystematic Reviews and *M*eta-*A*nalyses: The PRISMA Statement. PLoS Med 6(7): e1000097. doi:10.1371/journal.pmed1000097

For more information, visit www.prisma-statement.org.

Bladder Cancer

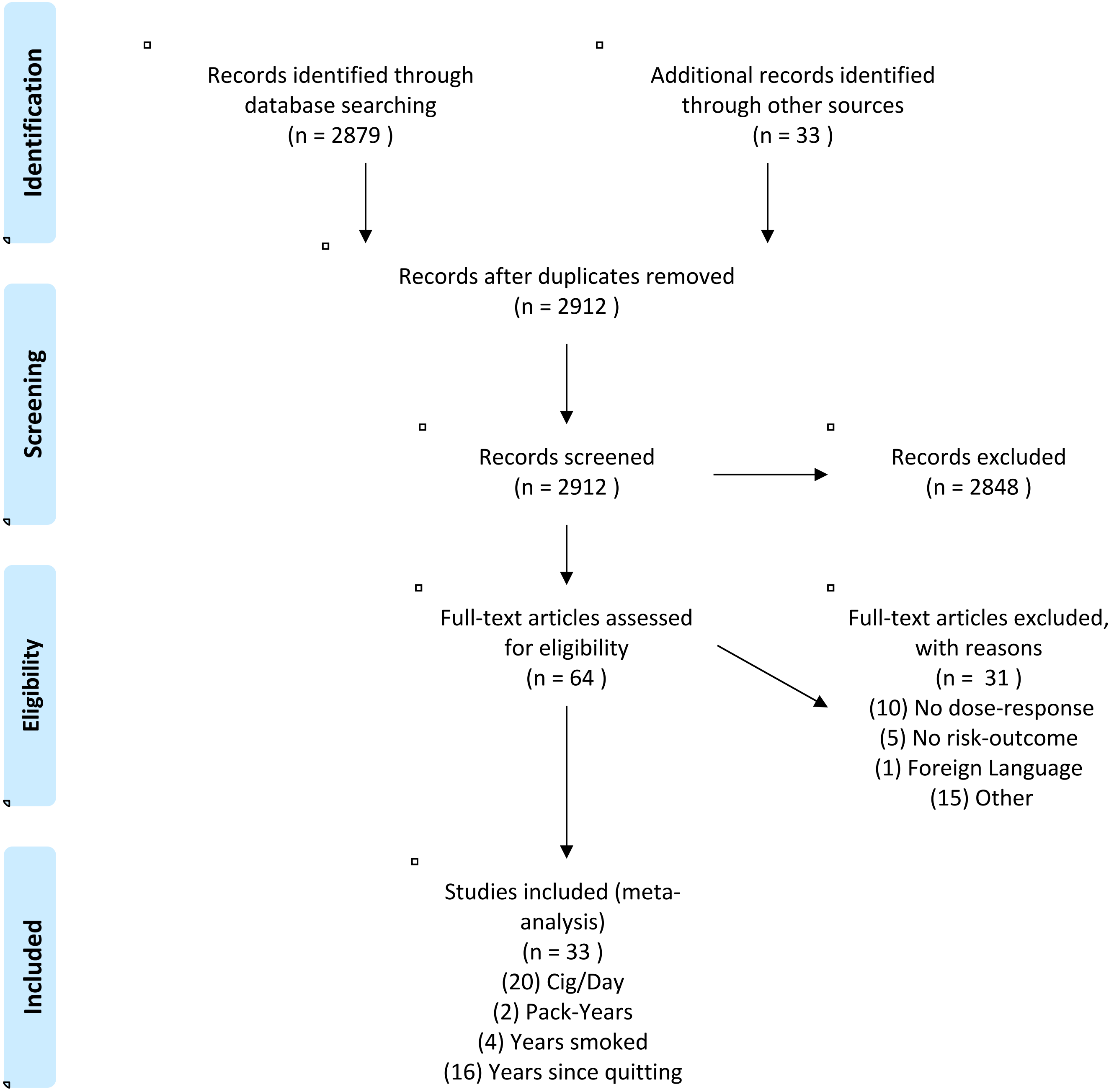

From: Moher D, Liberati A, Tetzlaff J, Altman DG, The PRISMA Group (2009). *P*referred *R*eporting *I*tems for *S*ystematic Reviews and *M*eta-*A*nalyses: The PRISMA Statement. PLoS Med 6(7): e1000097. doi:10.1371/journal.pmed1000097

For more information, visit www.prisma-statement.org.

Breast Cancer

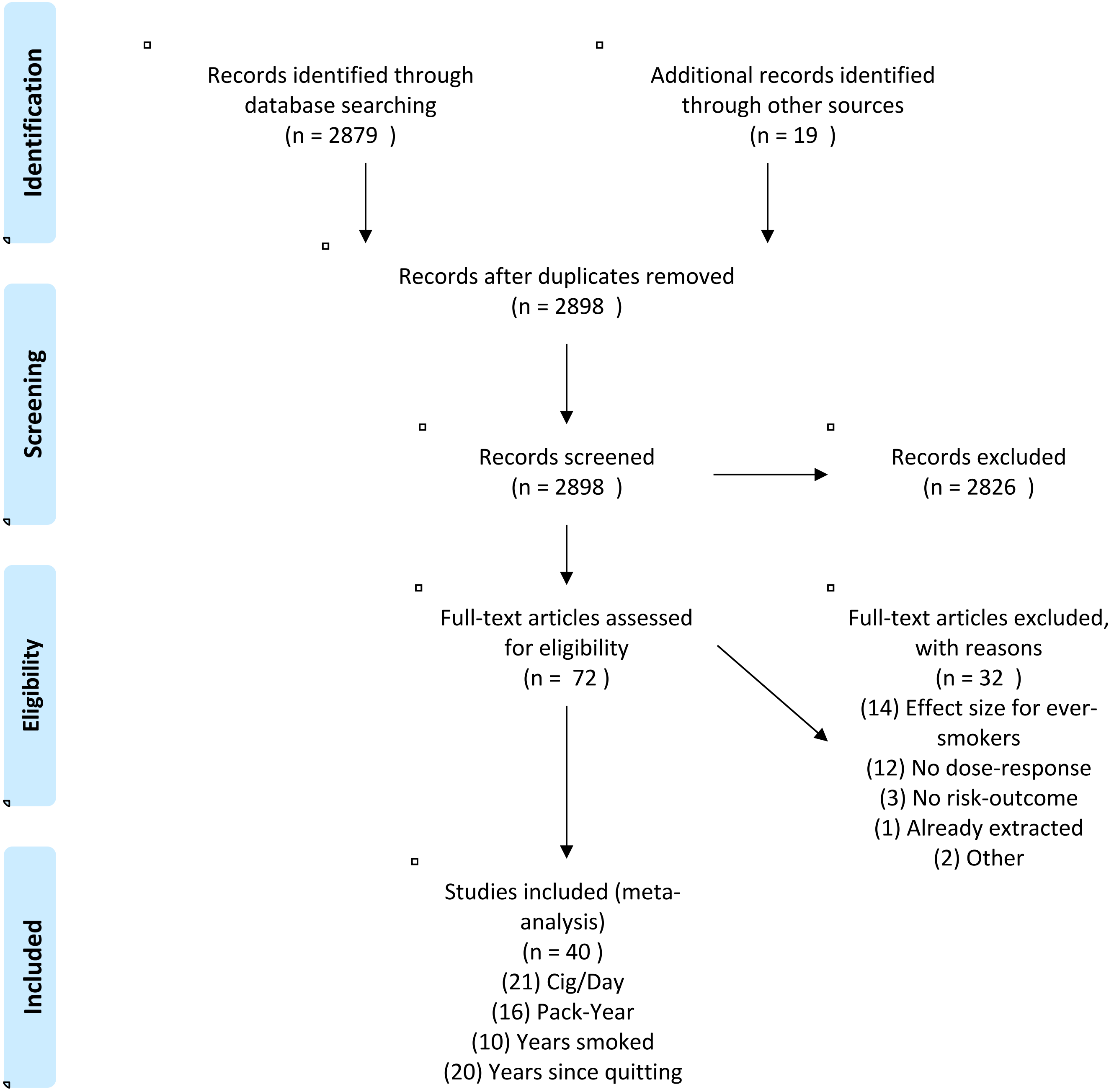

From: Moher D, Liberati A, Tetzlaff J, Altman DG, The PRISMA Group (2009). *P*referred *R*eporting *I*tems for *S*ystematic Reviews and *M*eta-*A*nalyses: The PRISMA Statement. PLoS Med 6(7): e1000097. doi:10.1371/journal.pmed1000097

For more information, visit www.prisma-statement.org.

Cataracts

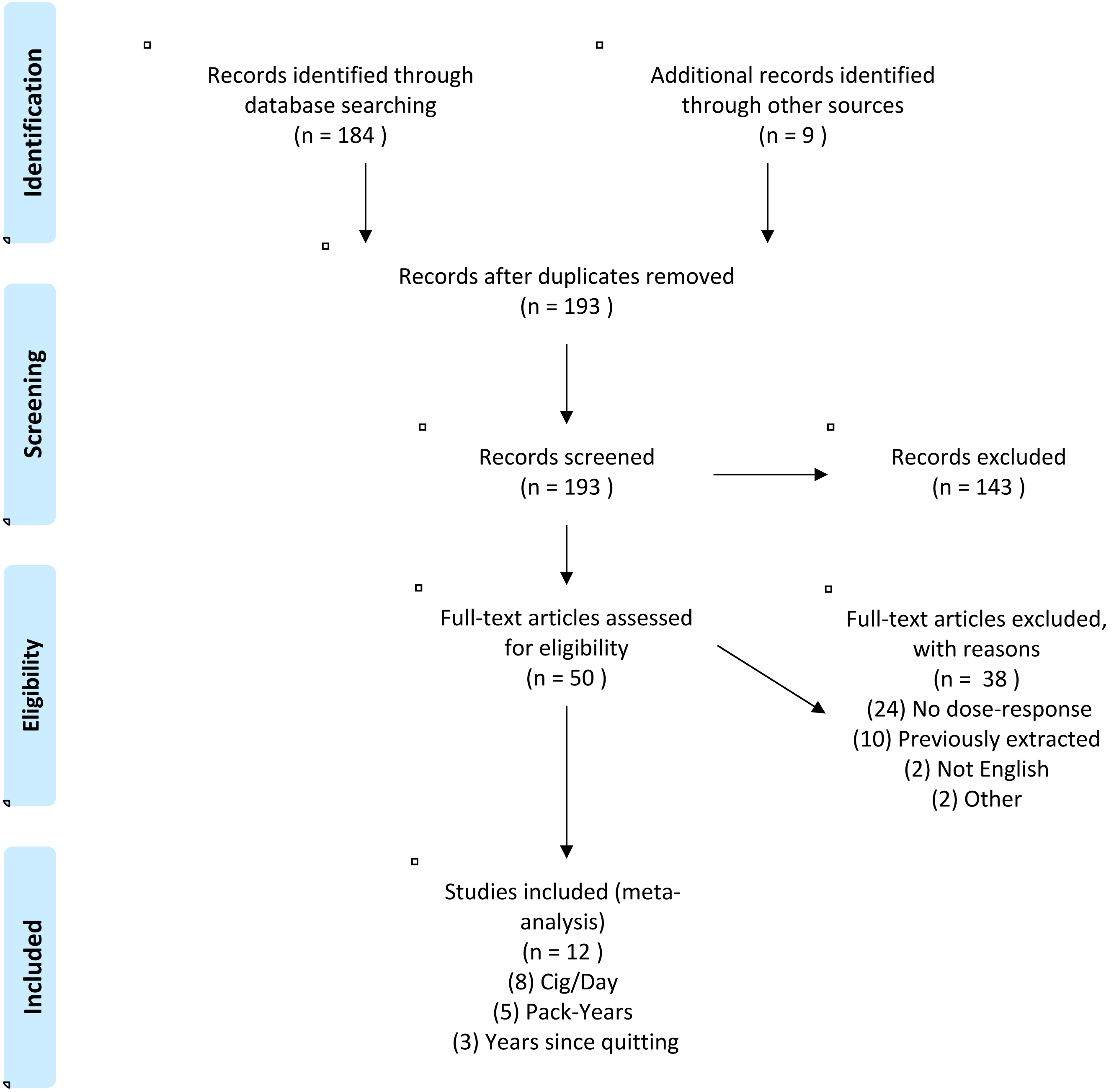

From: Moher D, Liberati A, Tetzlaff J, Altman DG, The PRISMA Group (2009). *P*referred *R*eporting *I*tems for *S*ystematic Reviews and *M*eta-*A*nalyses: The PRISMA Statement. PLoS Med 6(7): e1000097. doi:10.1371/journal.pmed1000097

For more information, visit www.prisma-statement.org.

Cervical Cancer

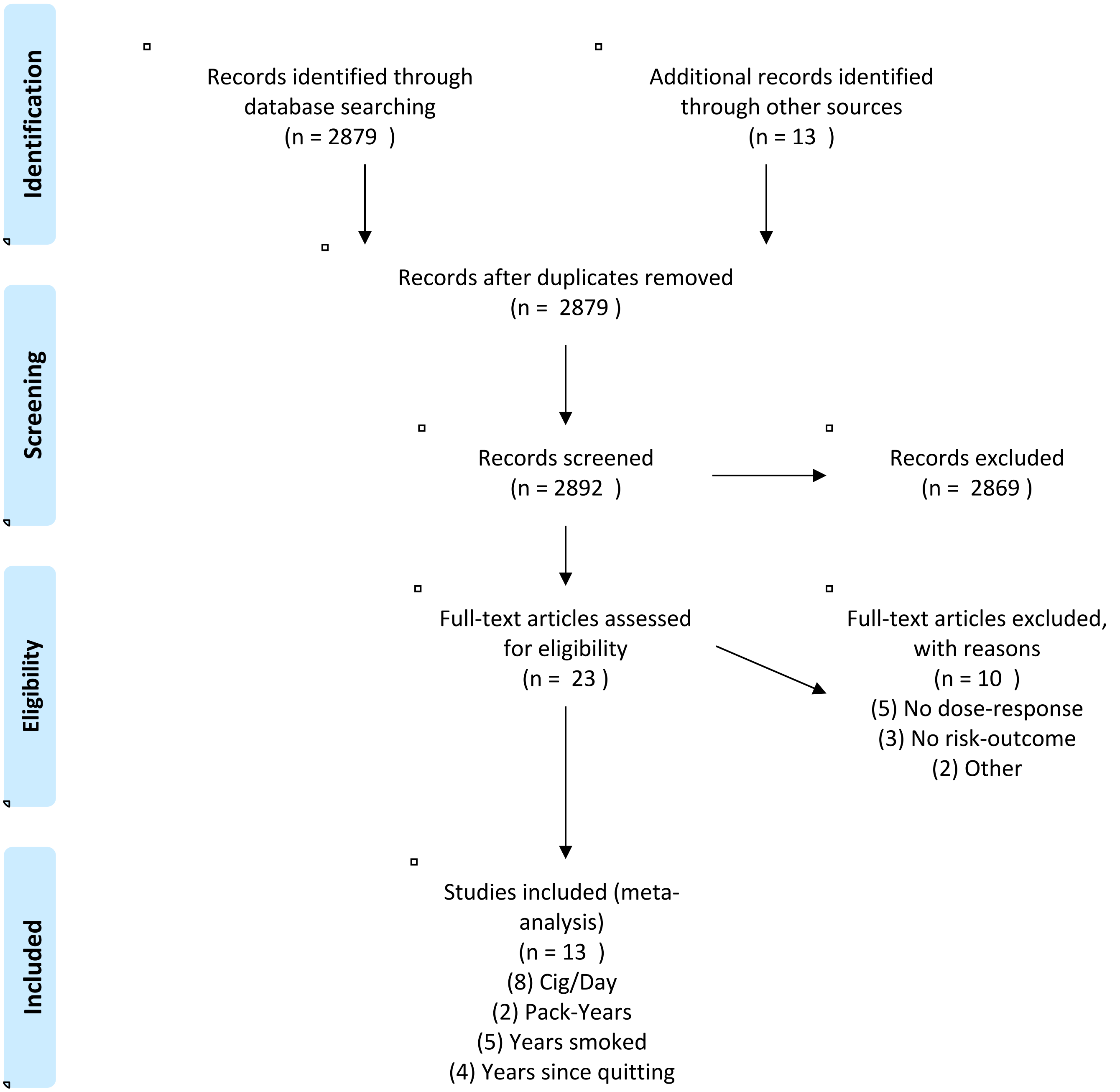

From: Moher D, Liberati A, Tetzlaff J, Altman DG, The PRISMA Group (2009). *P*referred *R*eporting *I*tems for *S*ystematic Reviews and *M*eta-*A*nalyses: The PRISMA Statement. PLoS Med 6(7): e1000097. doi:10.1371/journal.pmed1000097

For more information, visit www.prisma-statement.org.

Colon and Rectum Cancer

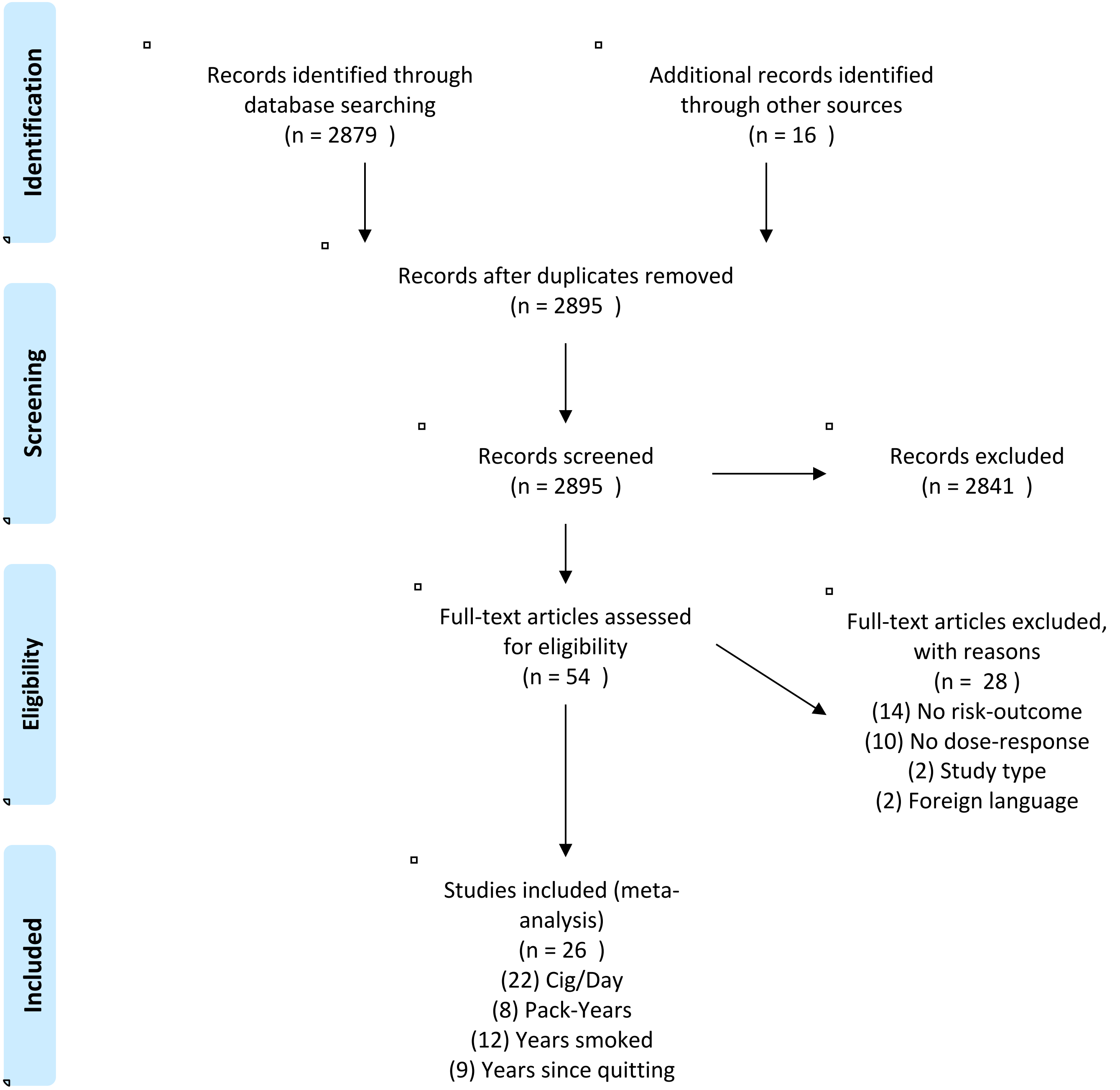

From: Moher D, Liberati A, Tetzlaff J, Altman DG, The PRISMA Group (2009). *P*referred *R*eporting *I*tems for *S*ystematic Reviews and *M*eta-*A*nalyses: The PRISMA Statement. PLoS Med 6(7): e1000097. doi:10.1371/journal.pmed1000097

For more information, visit www.prisma-statement.org.

Chronic Obstructive Pulmonary Disease

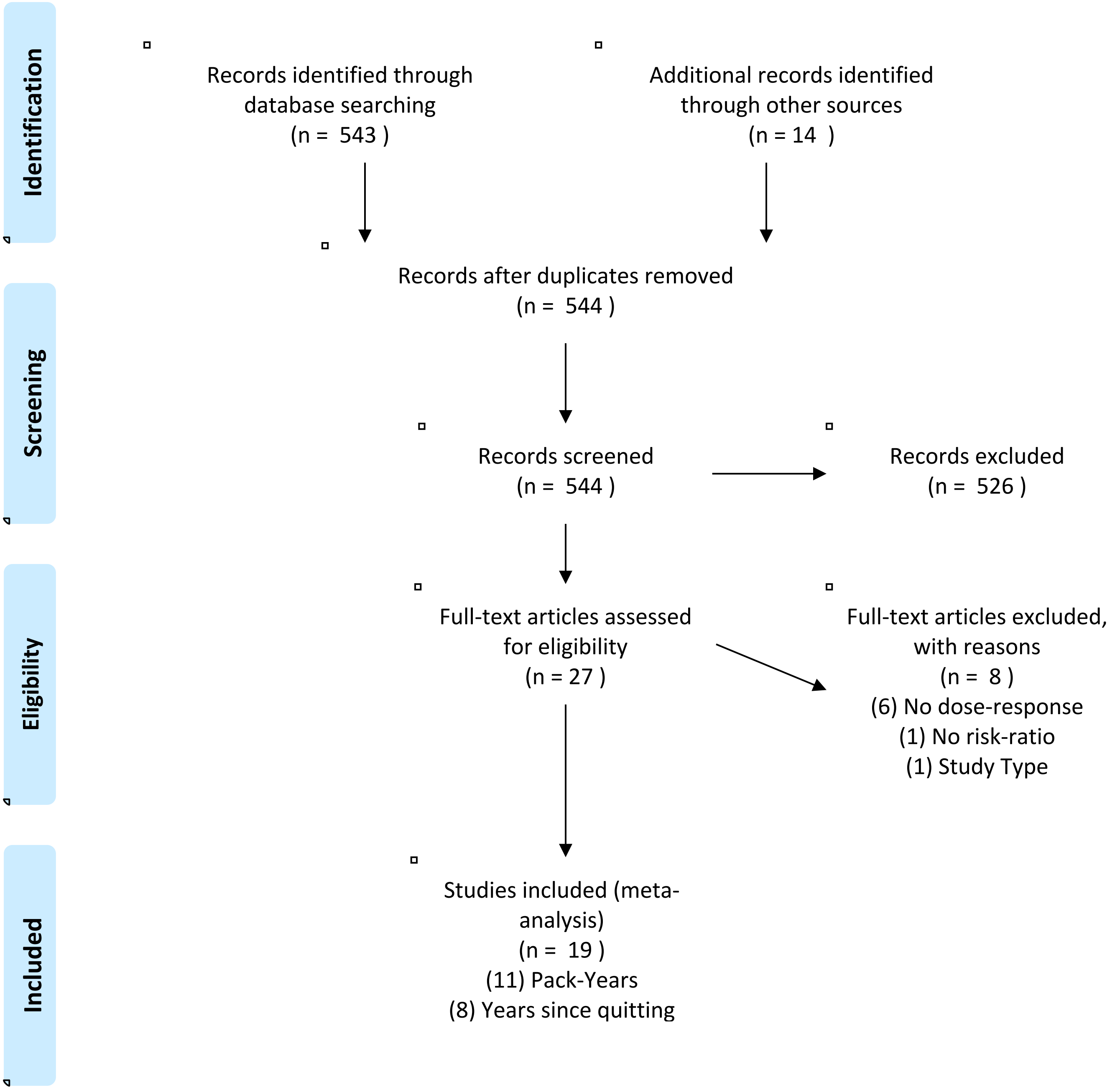

From: Moher D, Liberati A, Tetzlaff J, Altman DG, The PRISMA Group (2009). *P*referred *R*eporting *I*tems for *S*ystematic Reviews and *M*eta-*A*nalyses: The PRISMA Statement. PLoS Med 6(7): e1000097. doi:10.1371/journal.pmed1000097

For more information, visit www.prisma-statement.org.

Diabetes

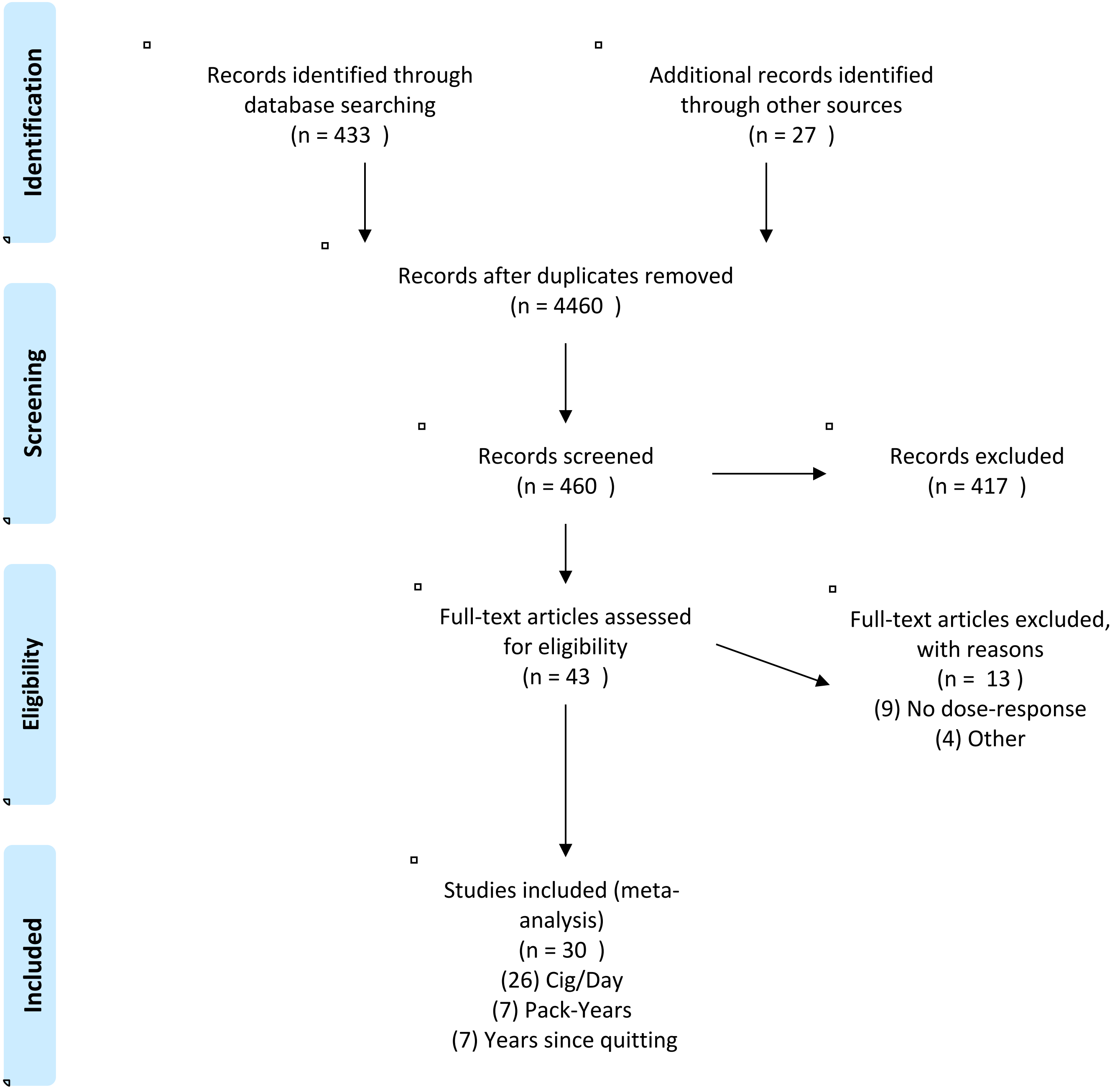

From: Moher D, Liberati A, Tetzlaff J, Altman DG, The PRISMA Group (2009). *P*referred *R*eporting *I*tems for *S*ystematic Reviews and *M*eta-*A*nalyses: The PRISMA Statement. PLoS Med 6(7): e1000097. doi:10.1371/journal.pmed1000097

For more information, visit www.prisma-statement.org.

Esophageal Cancer

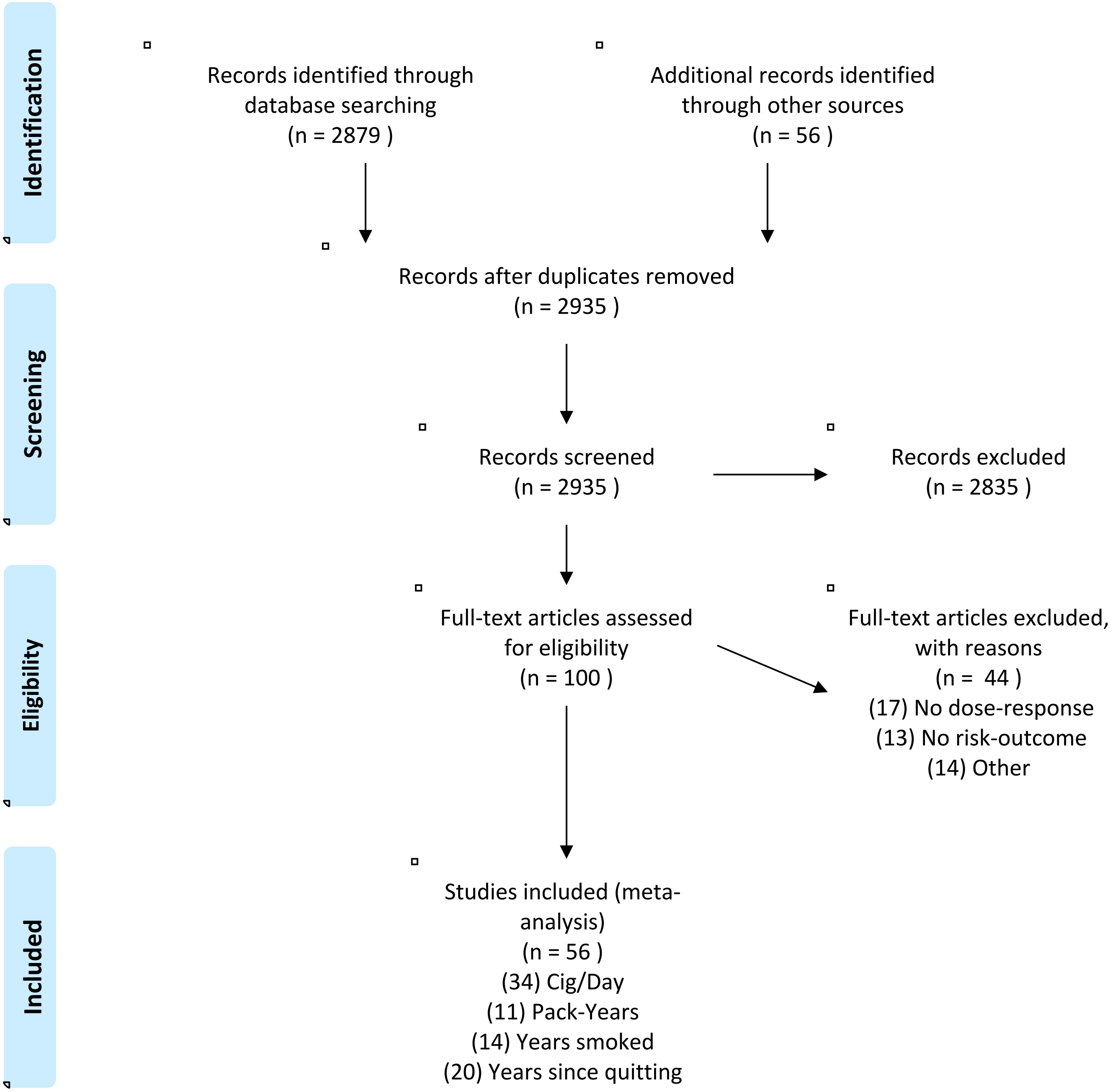

From: Moher D, Liberati A, Tetzlaff J, Altman DG, The PRISMA Group (2009). *P*referred *R*eporting *I*tems for *S*ystematic Reviews and *M*eta-*A*nalyses: The PRISMA Statement. PLoS Med 6(7): e1000097. doi:10.1371/journal.pmed1000097

For more information, visit www.prisma-statement.org.

Gallbladder and Biliary Tract Diseases

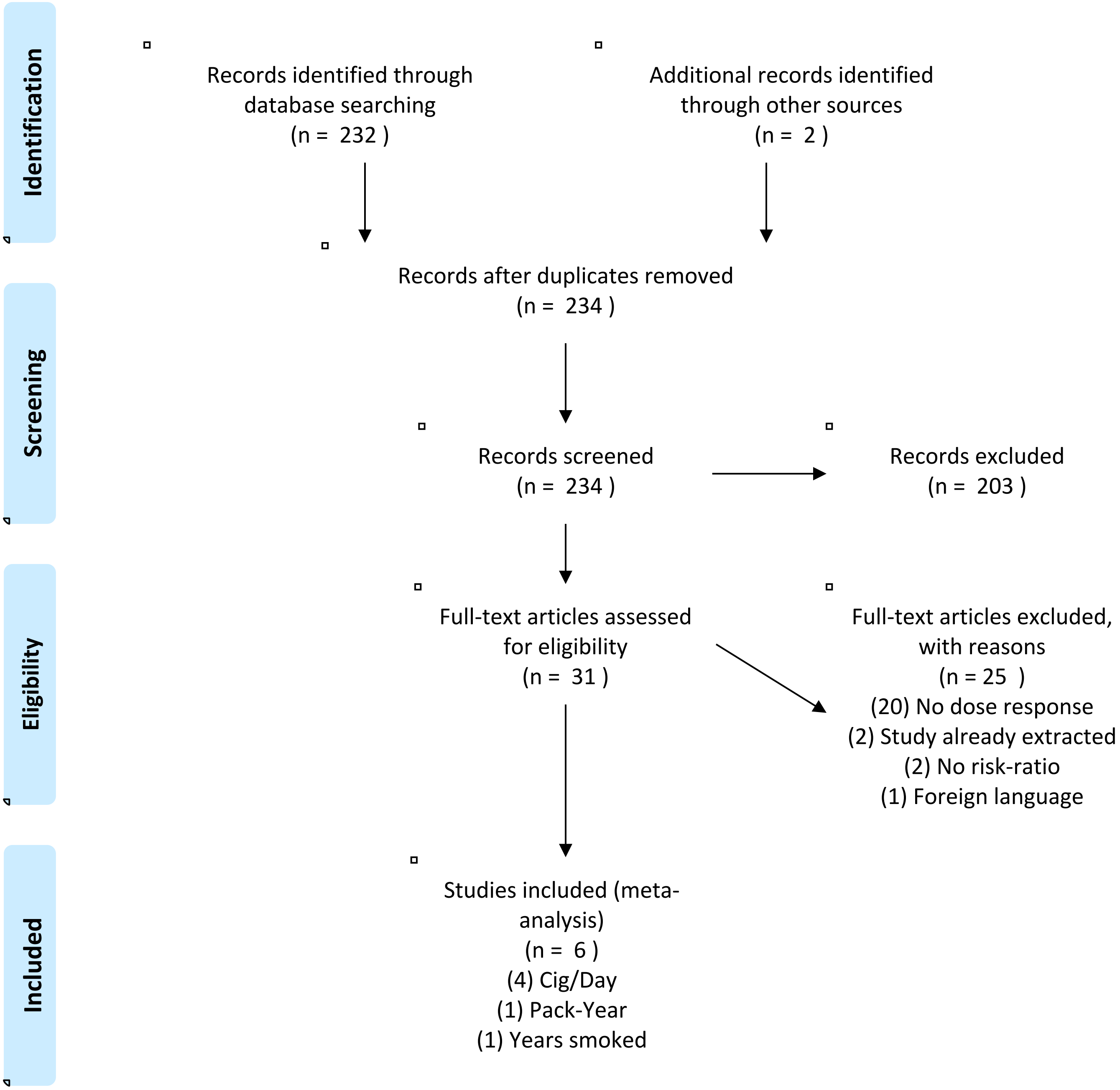

From: Moher D, Liberati A, Tetzlaff J, Altman DG, The PRISMA Group (2009). *P*referred *R*eporting *I*tems for *S*ystematic Reviews and *M*eta-*A*nalyses: The PRISMA Statement. PLoS Med 6(7): e1000097. doi:10.1371/journal.pmed1000097

For more information, visit www.prisma-statement.org.

Ischaemic Heart Disease

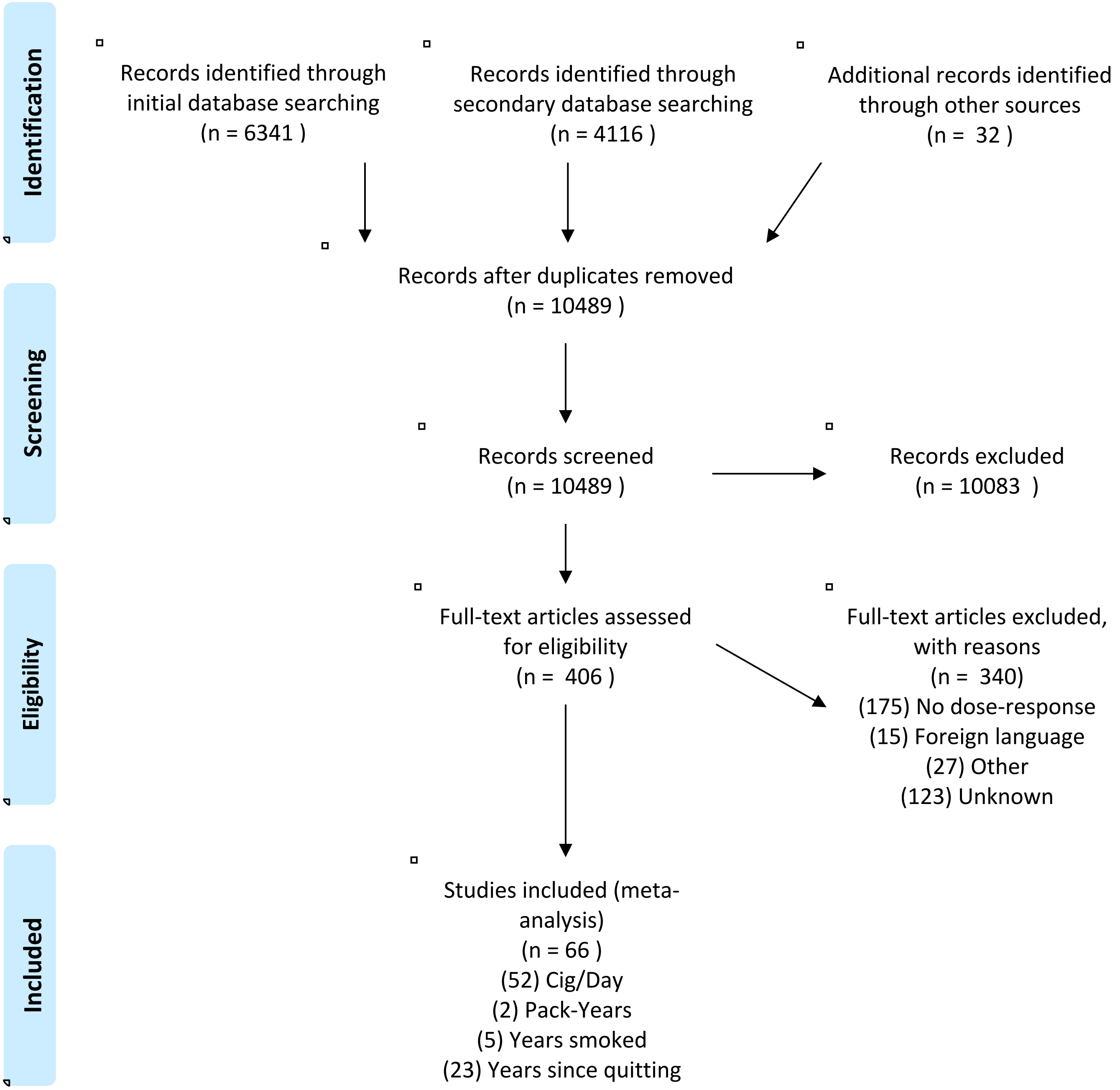

From: Moher D, Liberati A, Tetzlaff J, Altman DG, The PRISMA Group (2009). *P*referred *R*eporting *I*tems for *S*ystematic Reviews and *M*eta-*A*nalyses: The PRISMA Statement. PLoS Med 6(7): e1000097. doi:10.1371/journal.pmed1000097

For more information, visit www.prisma-statement.org.

Kidney Cancer

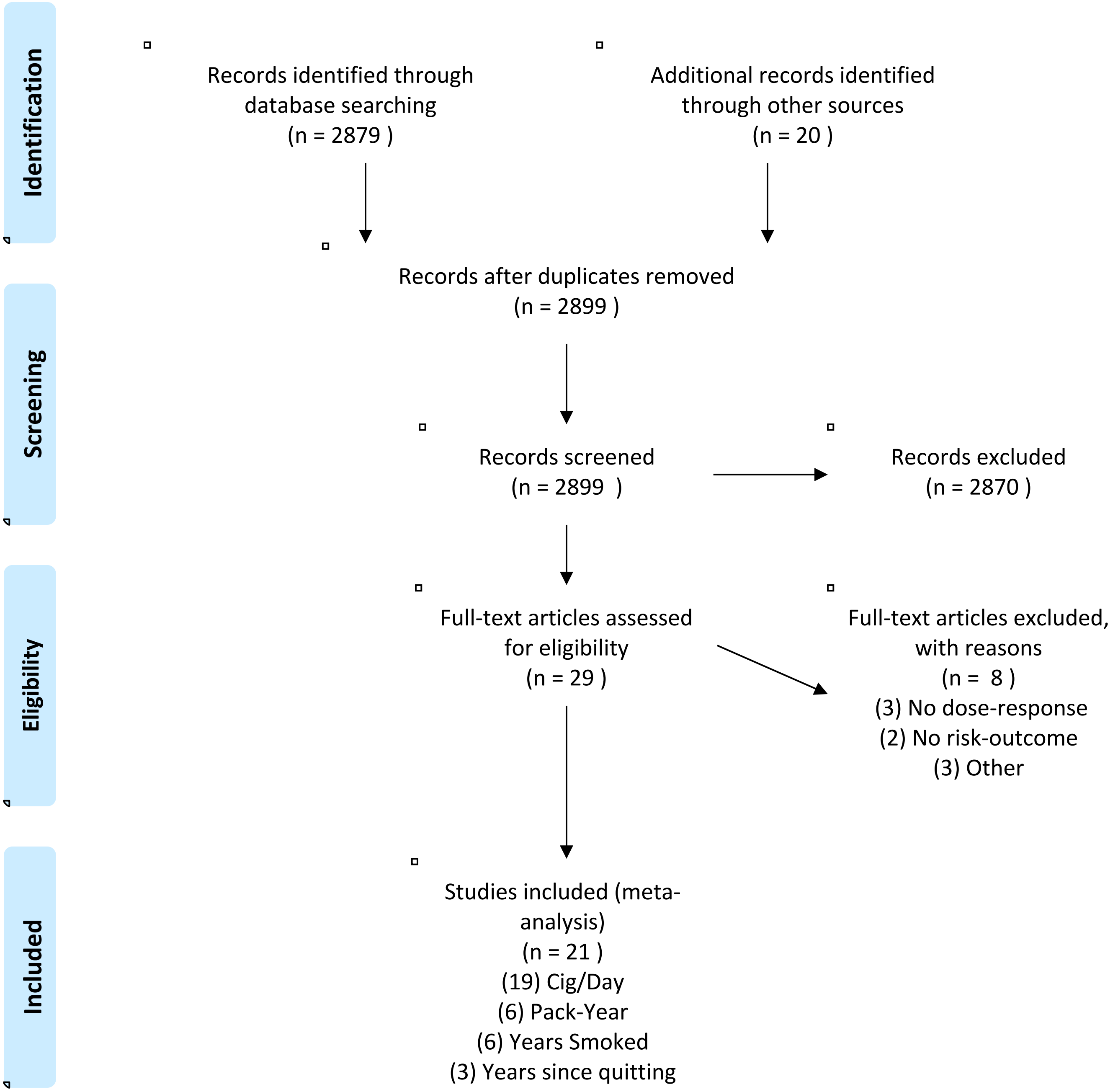

From: Moher D, Liberati A, Tetzlaff J, Altman DG, The PRISMA Group (2009). *P*referred *R*eporting *I*tems for *S*ystematic Reviews and *M*eta-*A*nalyses: The PRISMA Statement. PLoS Med 6(7): e1000097. doi:10.1371/journal.pmed1000097

For more information, visit www.prisma-statement.org.

Laryngeal Cancer

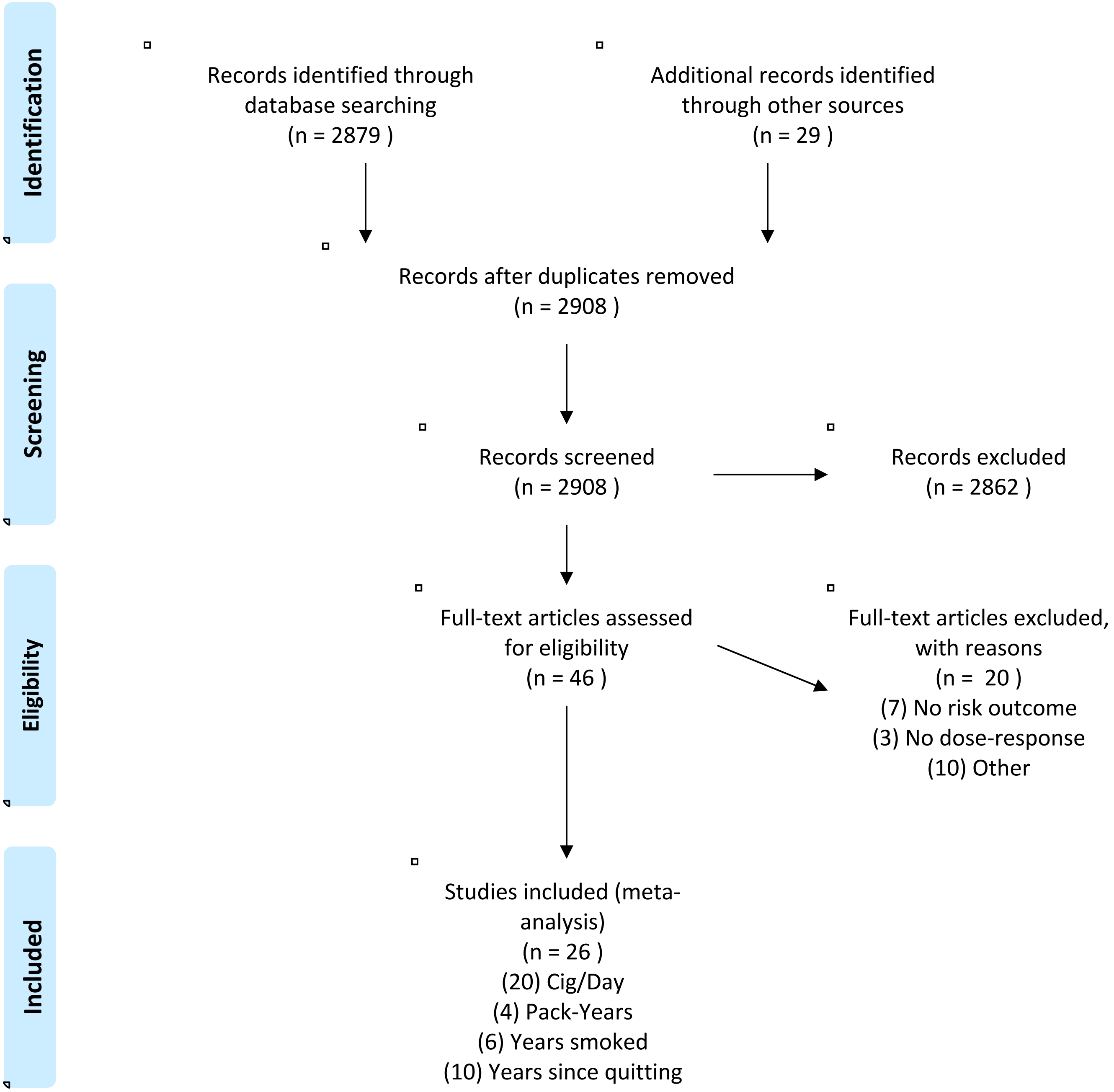

From: Moher D, Liberati A, Tetzlaff J, Altman DG, The PRISMA Group (2009). *P*referred *R*eporting *I*tems for *S*ystematic Reviews and *M*eta-*A*nalyses: The PRISMA Statement. PLoS Med 6(7): e1000097. doi:10.1371/journal.pmed1000097

For more information, visit www.prisma-statement.org.

Leukemia

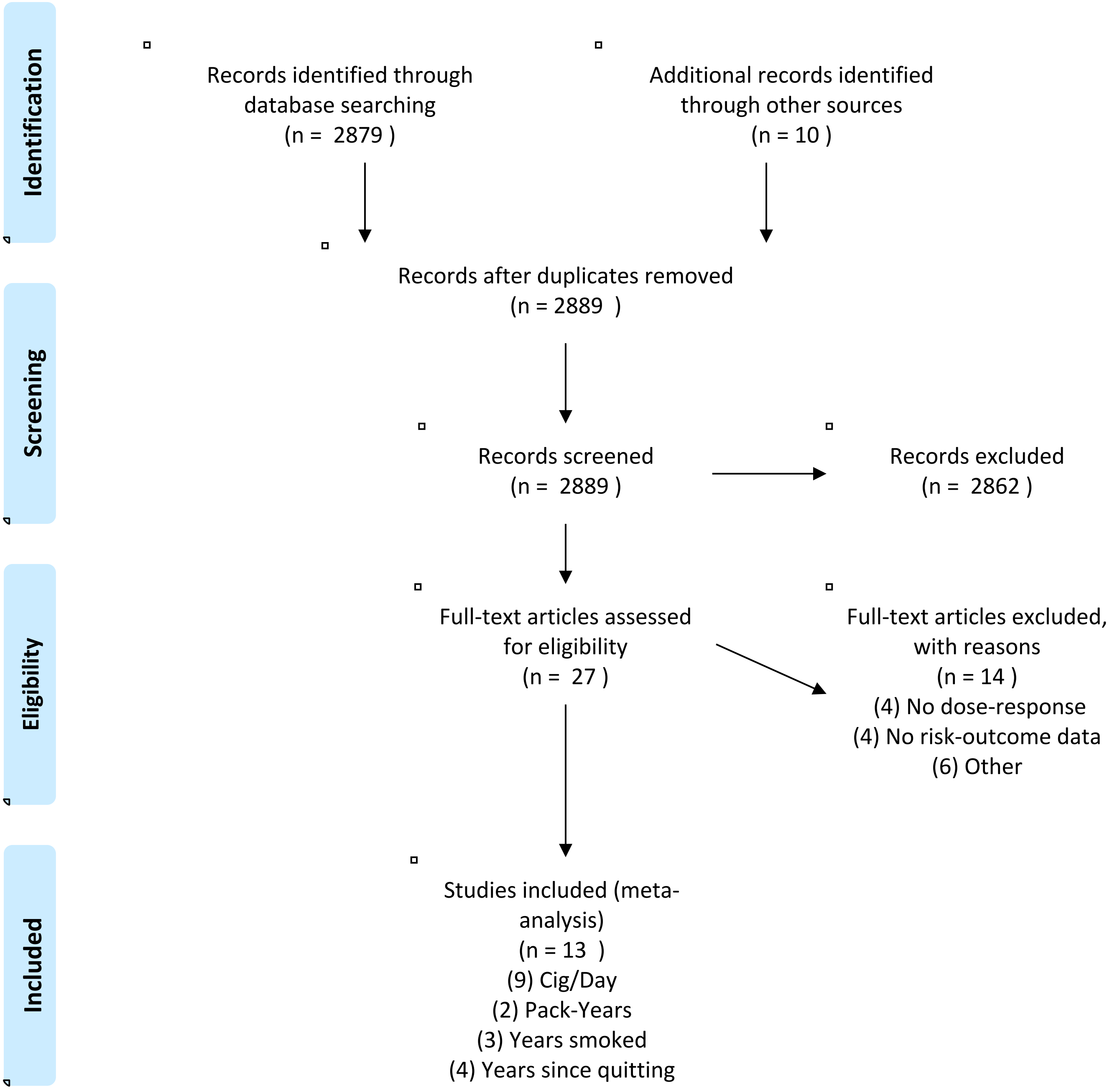

From: Moher D, Liberati A, Tetzlaff J, Altman DG, The PRISMA Group (2009). *P*referred *R*eporting *I*tems for *S*ystematic Reviews and *M*eta-*A*nalyses: The PRISMA Statement. PLoS Med 6(7): e1000097. doi:10.1371/journal.pmed1000097

For more information, visit www.prisma-statement.org.

Lip and Oral Cavity Cancer

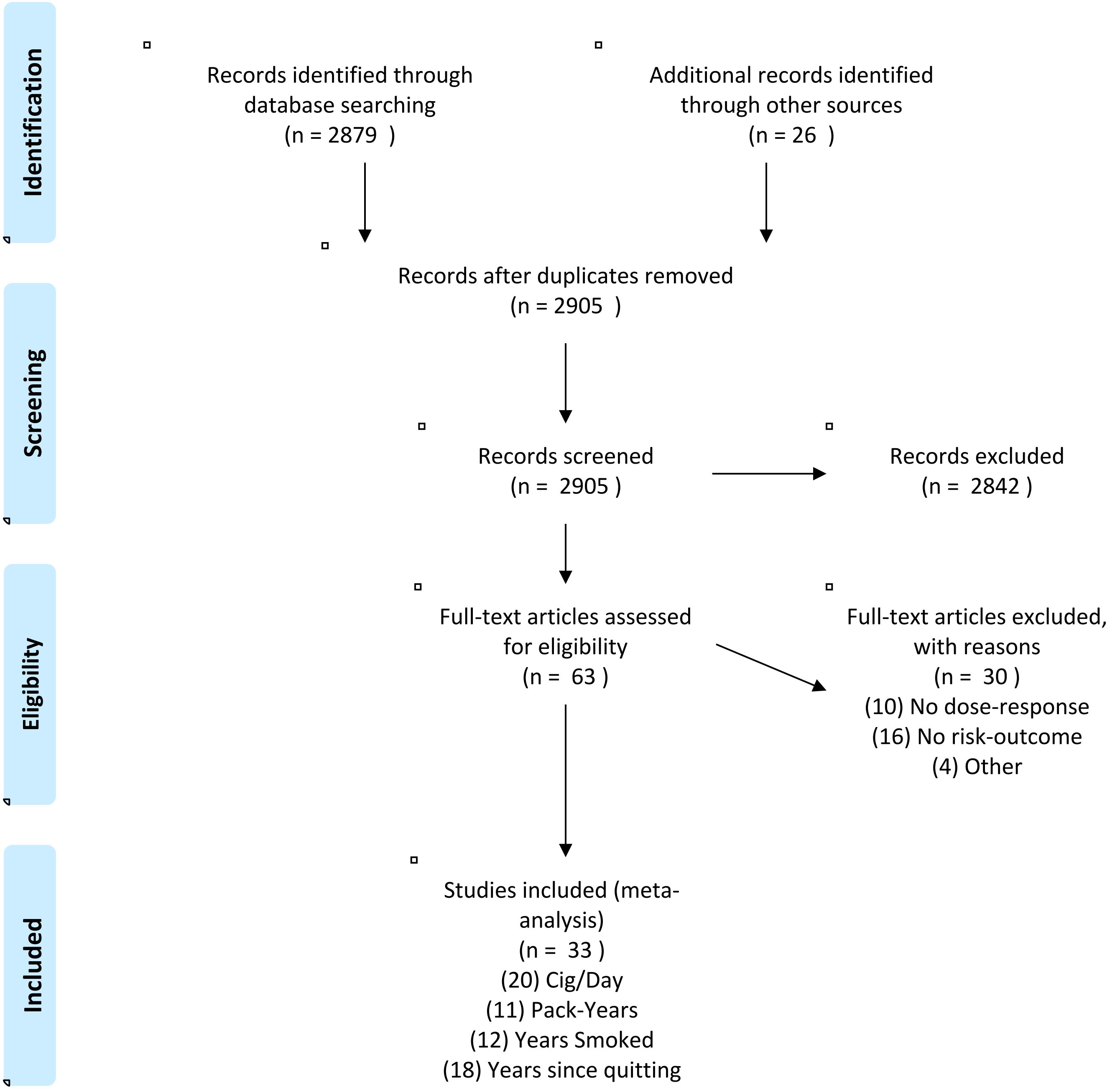

From: Moher D, Liberati A, Tetzlaff J, Altman DG, The PRISMA Group (2009). *P*referred *R*eporting *I*tems for *S*ystematic Reviews and *M*eta-*A*nalyses: The PRISMA Statement. PLoS Med 6(7): e1000097. doi:10.1371/journal.pmed1000097

For more information, visit www.prisma-statement.org.

Liver Cancer

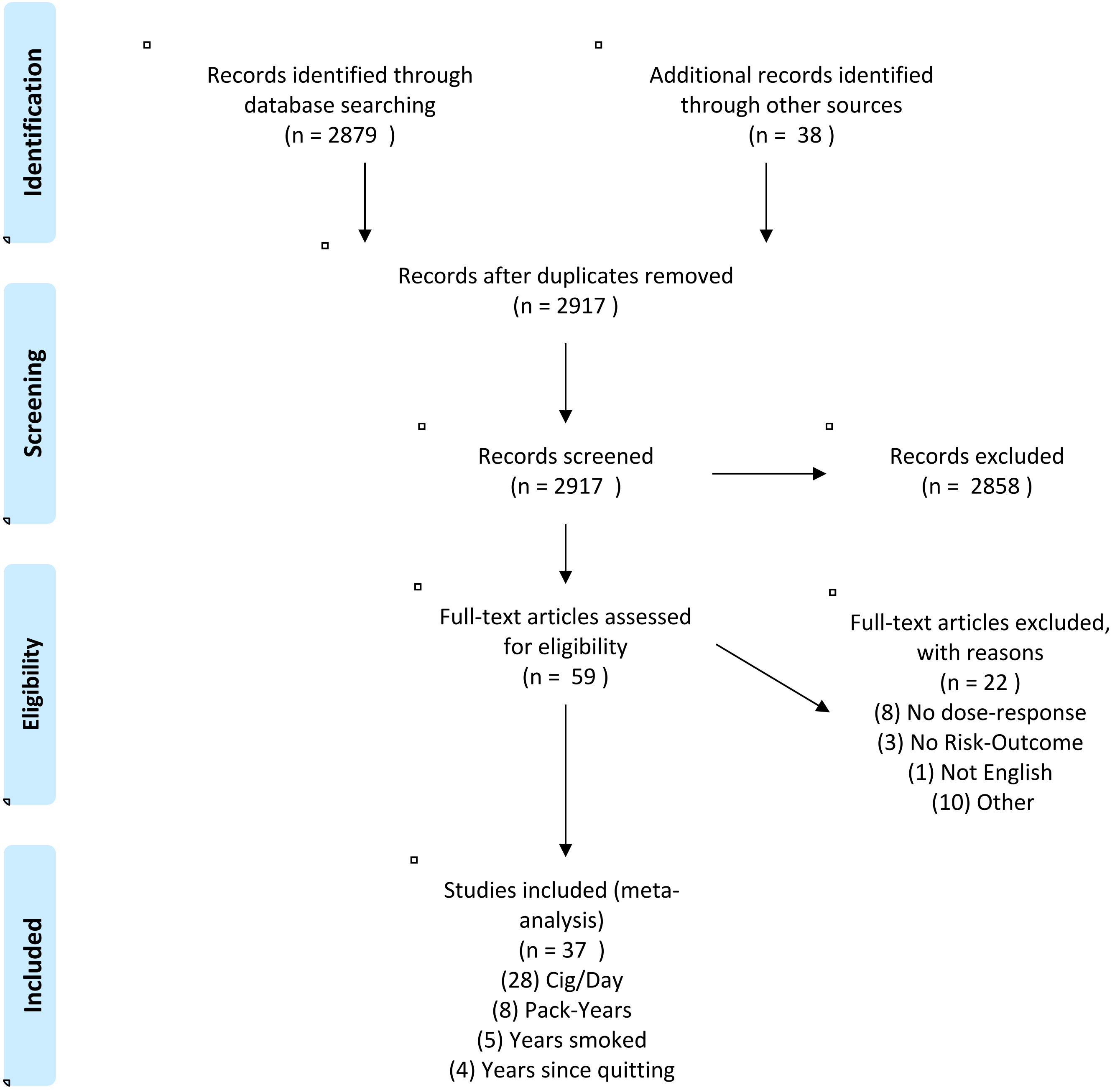

From: Moher D, Liberati A, Tetzlaff J, Altman DG, The PRISMA Group (2009). *P*referred *R*eporting *I*tems for *S*ystematic Reviews and *M*eta-*A*nalyses: The PRISMA Statement. PLoS Med 6(7): e1000097. doi:10.1371/journal.pmed1000097

For more information, visit www.prisma-statement.org.

Low Back Pain

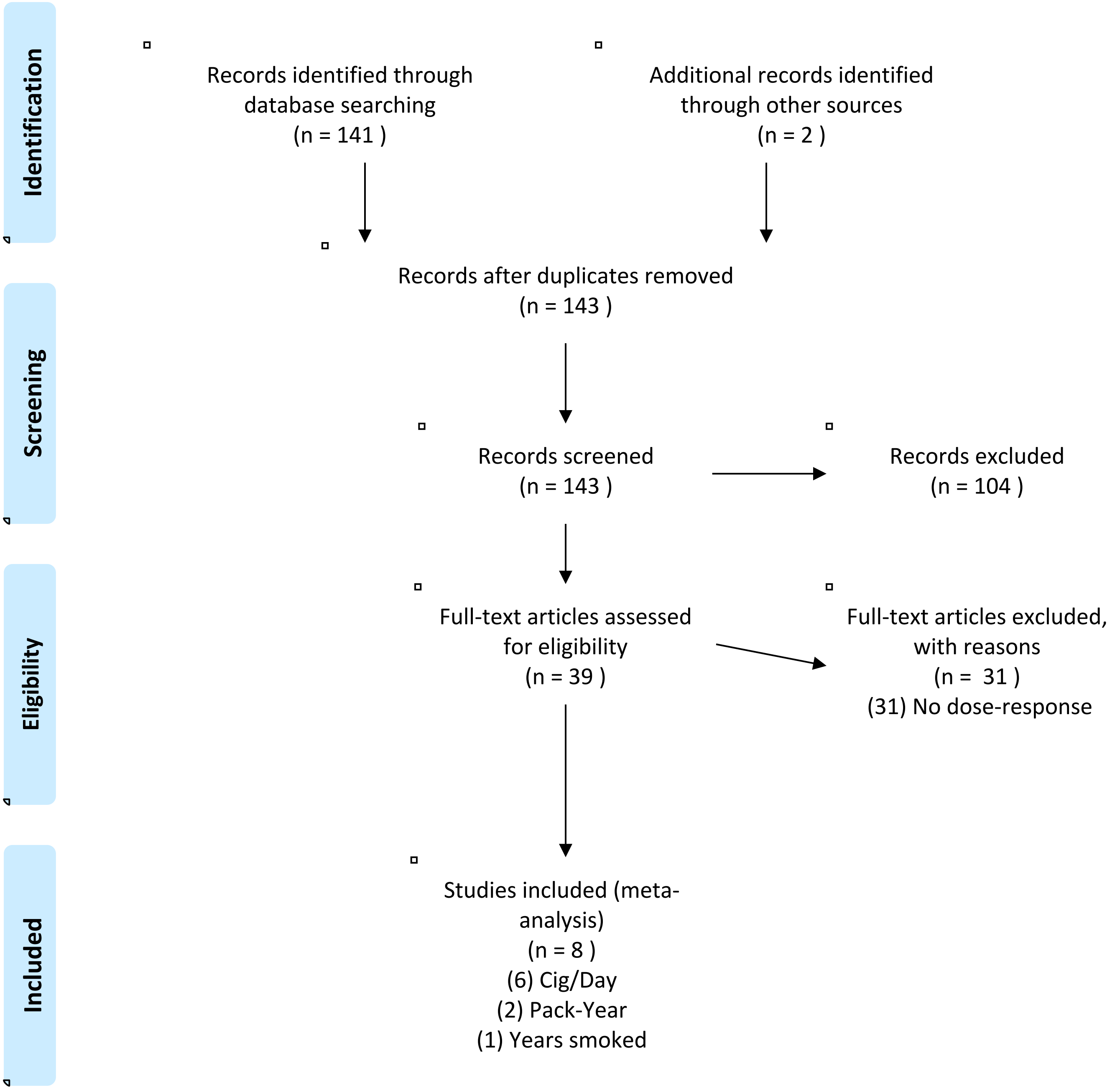

From: Moher D, Liberati A, Tetzlaff J, Altman DG, The PRISMA Group (2009). *P*referred *R*eporting *I*tems for *S*ystematic Reviews and *M*eta-*A*nalyses: The PRISMA Statement. PLoS Med 6(7): e1000097. doi:10.1371/journal.pmed1000097

For more information, visit www.prisma-statement.org.

Lower Respiratory Tract Infections

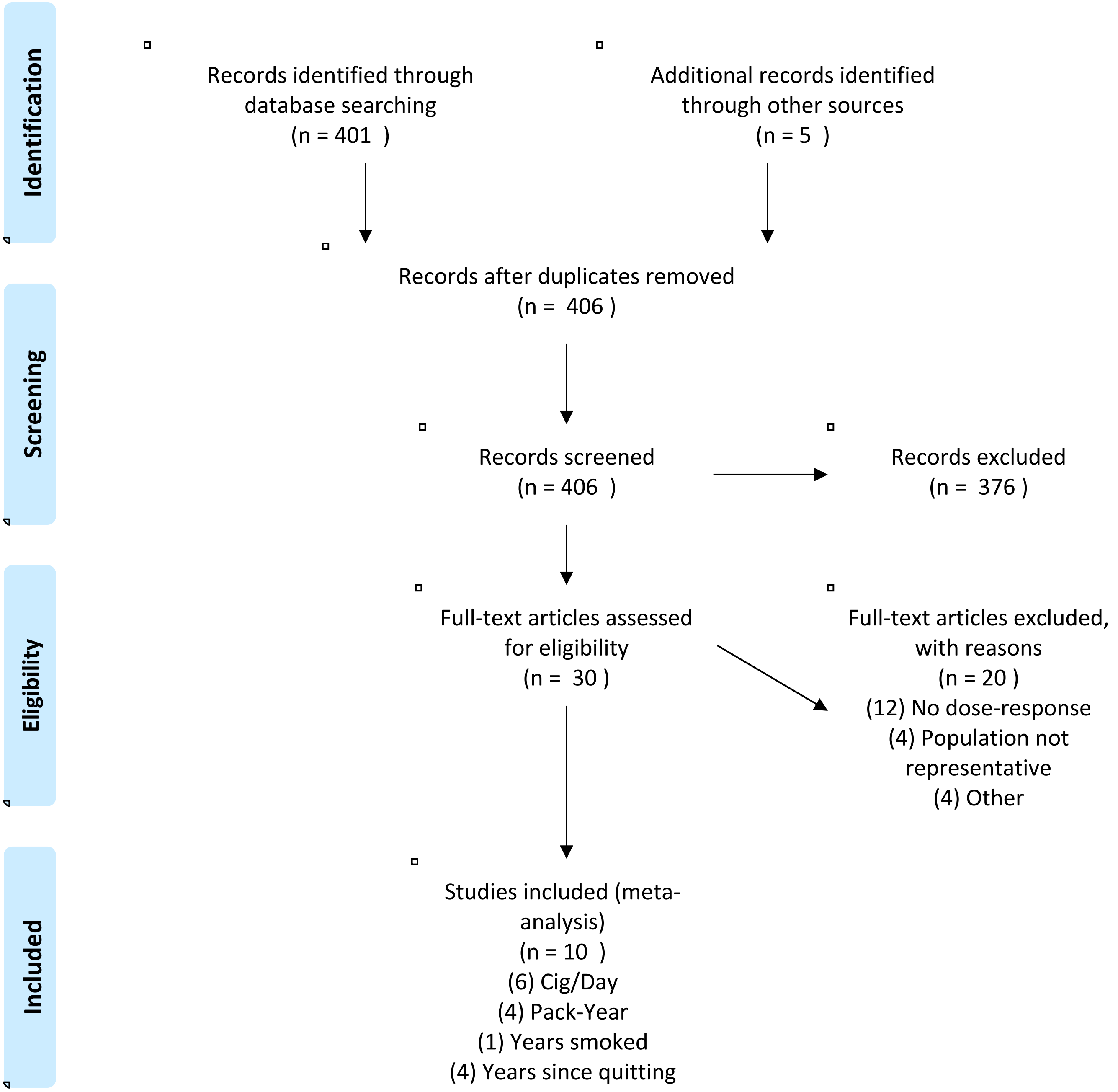

From: Moher D, Liberati A, Tetzlaff J, Altman DG, The PRISMA Group (2009). *P*referred *R*eporting *I*tems for *S*ystematic Reviews and *M*eta-*A*nalyses: The PRISMA Statement. PLoS Med 6(7): e1000097. doi:10.1371/journal.pmed1000097

For more information, visit www.prisma-statement.org.

Lung Cancer

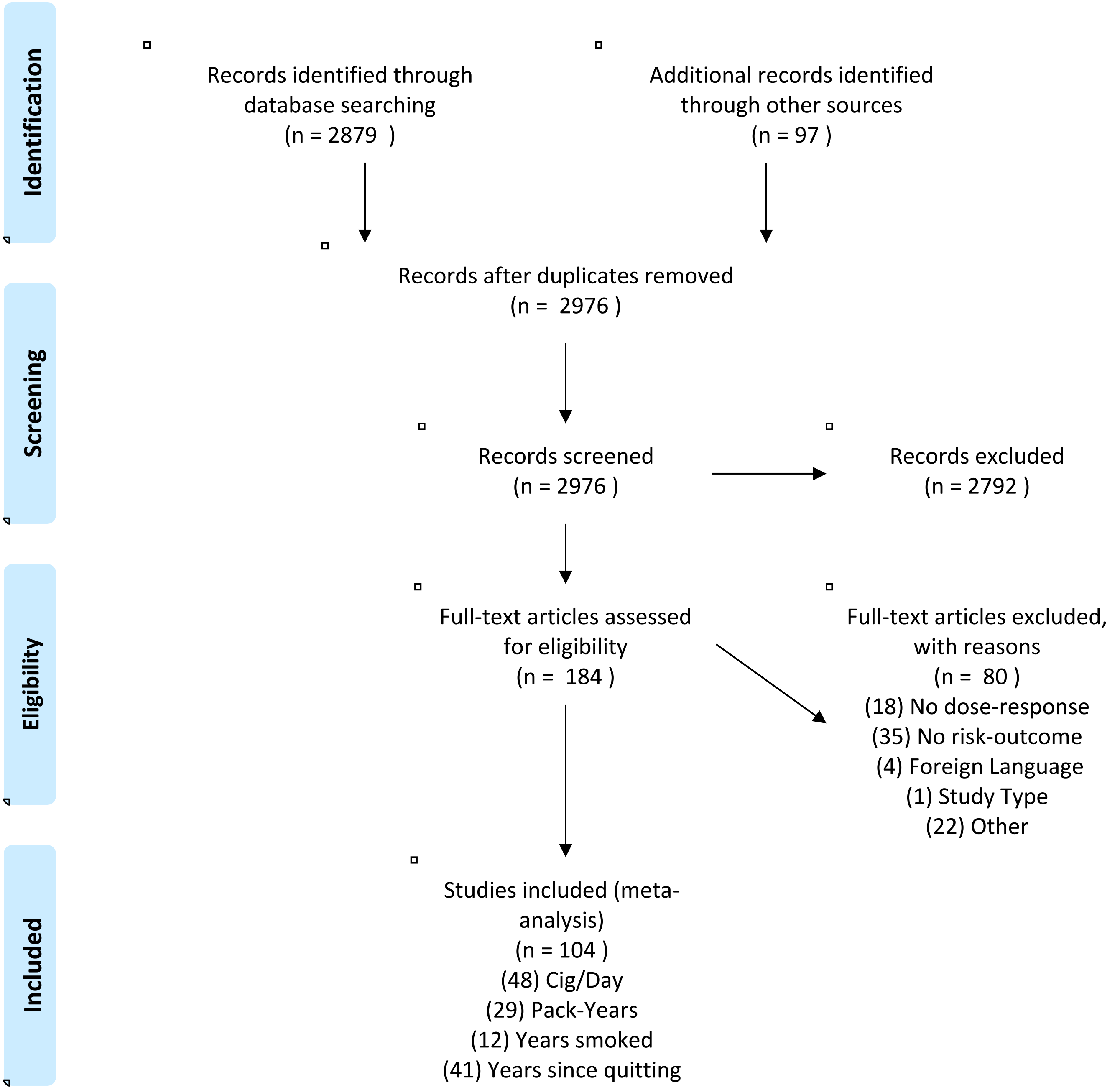

From: Moher D, Liberati A, Tetzlaff J, Altman DG, The PRISMA Group (2009). *P*referred *R*eporting *I*tems for *S*ystematic Reviews and *M*eta-*A*nalyses: The PRISMA Statement. PLoS Med 6(7): e1000097. doi:10.1371/journal.pmed1000097

For more information, visit www.prisma-statement.org.

Macular Degeneration

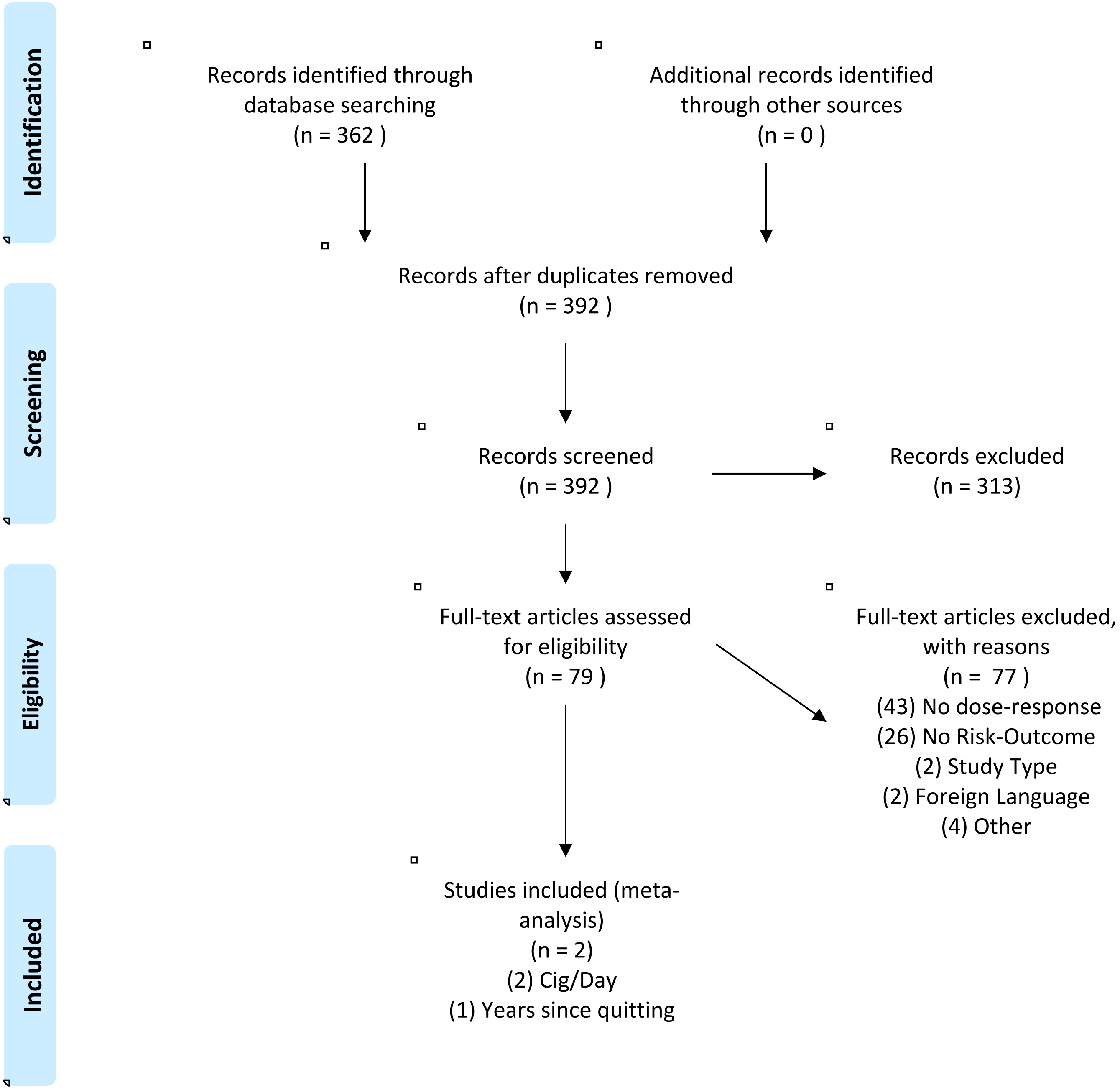

From: Moher D, Liberati A, Tetzlaff J, Altman DG, The PRISMA Group (2009). *P*referred *R*eporting *I*tems for *S*ystematic Reviews and *M*eta-*A*nalyses: The PRISMA Statement. PLoS Med 6(7): e1000097. doi:10.1371/journal.pmed1000097

For more information, visit www.prisma-statement.org.

Multiple Sclerosis

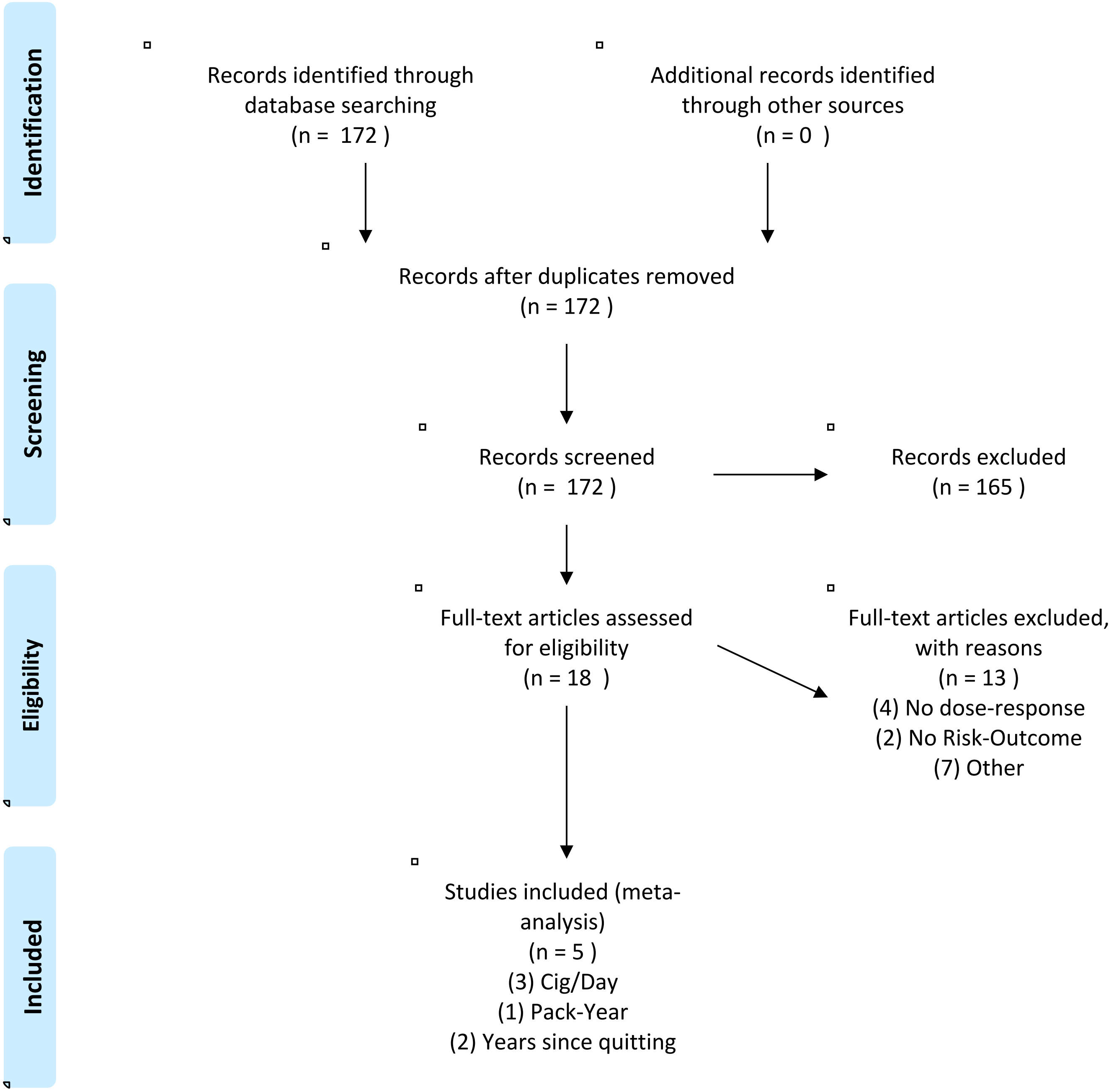

From: Moher D, Liberati A, Tetzlaff J, Altman DG, The PRISMA Group (2009). *P*referred *R*eporting *I*tems for *S*ystematic Reviews and *M*eta-*A*nalyses: The PRISMA Statement. PLoS Med 6(7): e1000097. doi:10.1371/journal.pmed1000097

For more information, visit www.prisma-statement.org.

Nasopharyngeal Cancer

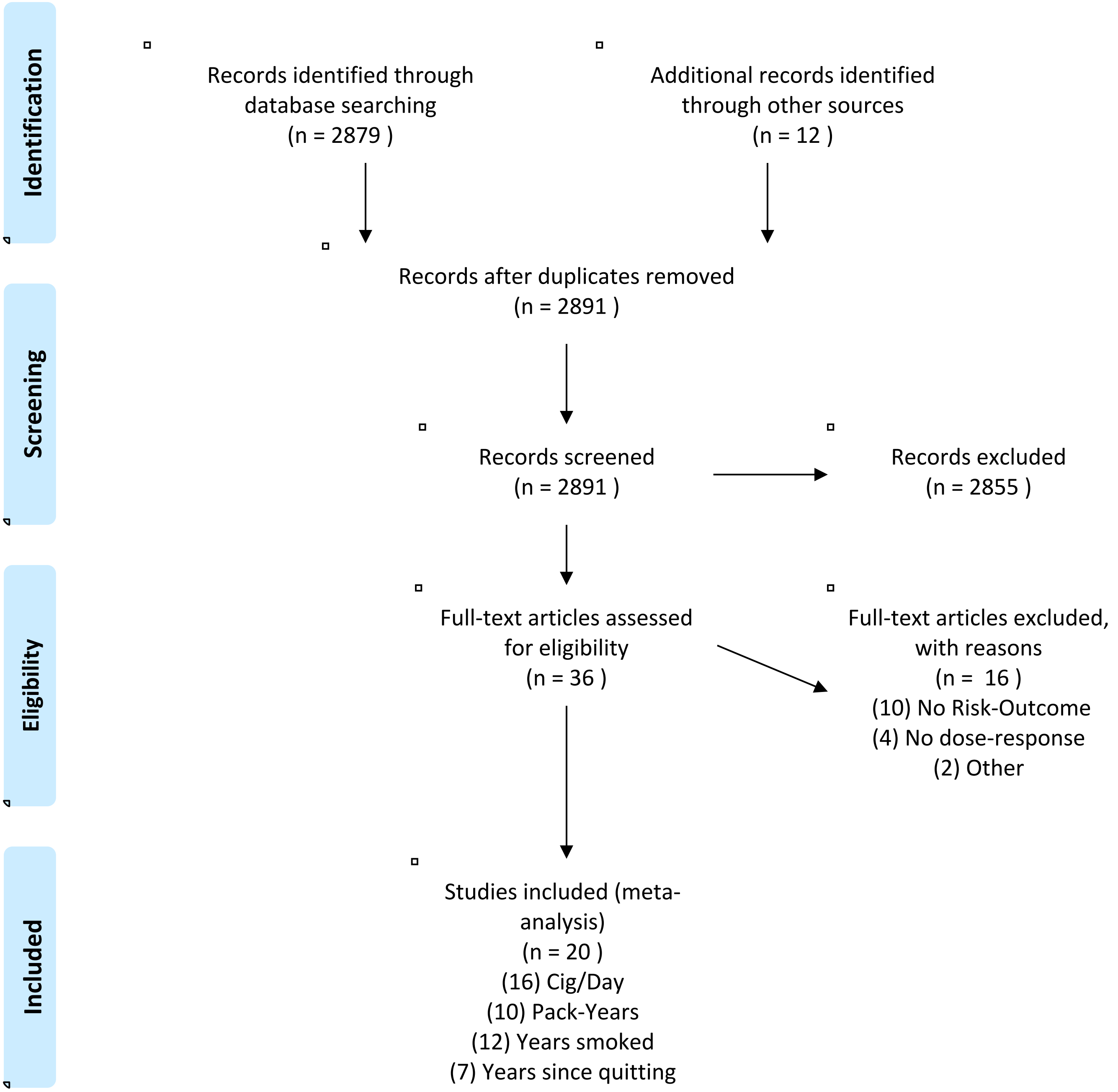

From: Moher D, Liberati A, Tetzlaff J, Altman DG, The PRISMA Group (2009). *P*referred *R*eporting *I*tems for *S*ystematic Reviews and *M*eta-*A*nalyses: The PRISMA Statement. PLoS Med 6(7): e1000097. doi:10.1371/journal.pmed1000097

For more information, visit www.prisma-statement.org.

Other Pharyngeal Cancer

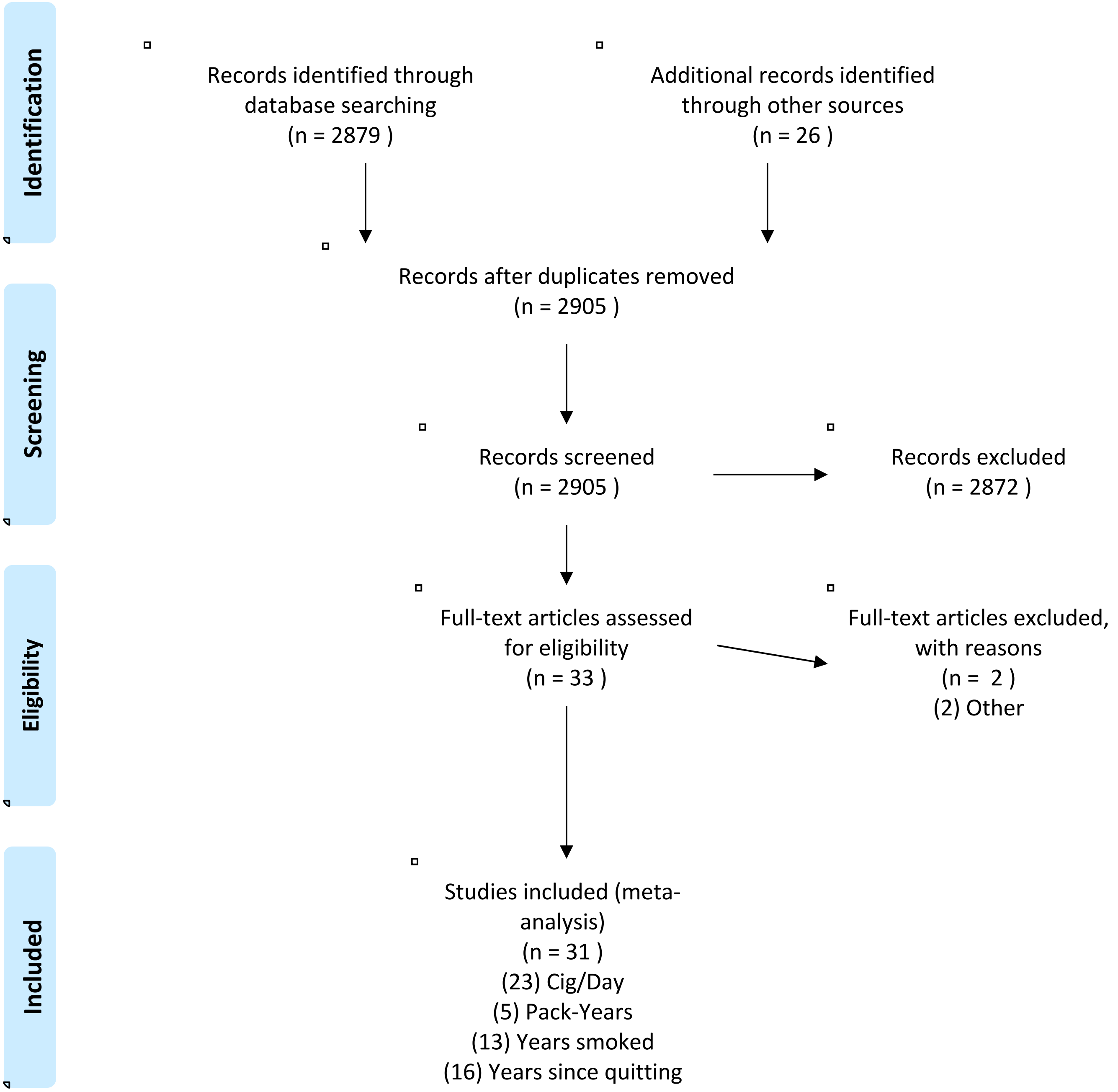

From: Moher D, Liberati A, Tetzlaff J, Altman DG, The PRISMA Group (2009). *P*referred *R*eporting *I*tems for *S*ystematic Reviews and *M*eta-*A*nalyses: The PRISMA Statement. PLoS Med 6(7): e1000097. doi:10.1371/journal.pmed1000097

For more information, visit www.prisma-statement.org.

Pancreatic Cancer

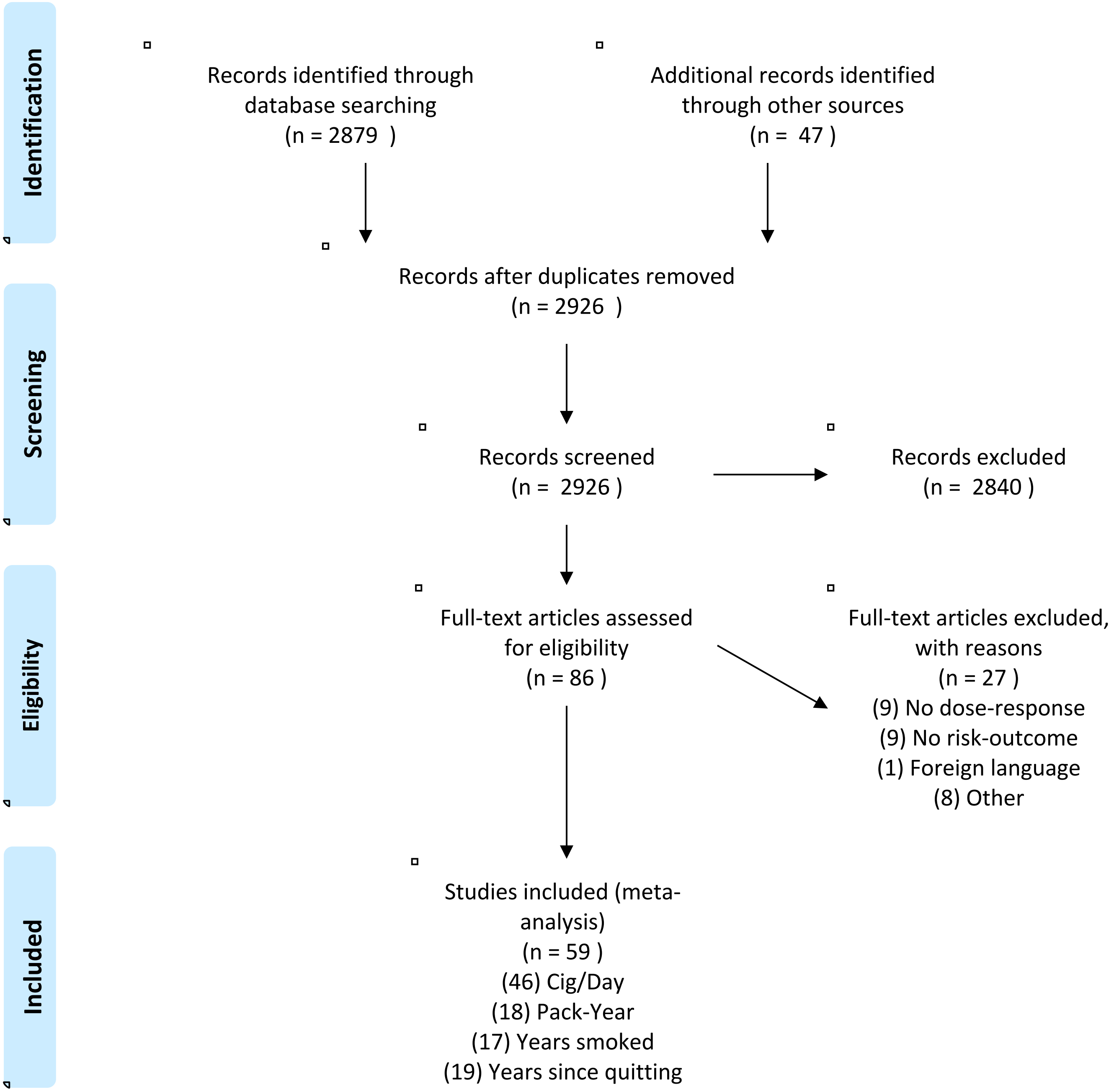

From: Moher D, Liberati A, Tetzlaff J, Altman DG, The PRISMA Group (2009). *P*referred *R*eporting *I*tems for *S*ystematic Reviews and *M*eta-*A*nalyses: The PRISMA Statement. PLoS Med 6(7): e1000097. doi:10.1371/journal.pmed1000097

For more information, visit www.prisma-statement.org.

Parkinson's Disease

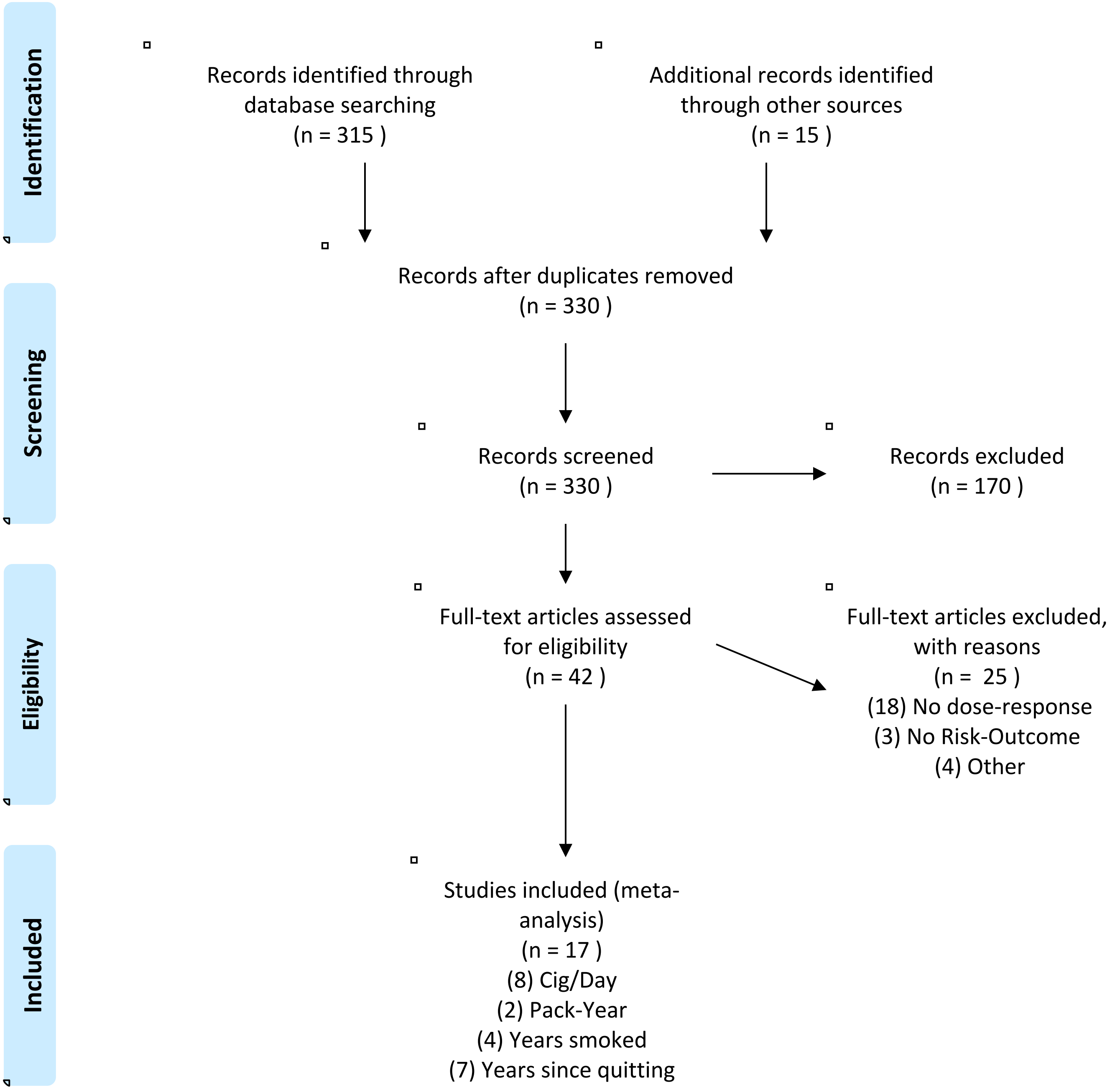

From: Moher D, Liberati A, Tetzlaff J, Altman DG, The PRISMA Group (2009). *P*referred *R*eporting *I*tems for *S*ystematic Reviews and *M*eta-*A*nalyses: The PRISMA Statement. PLoS Med 6(7): e1000097. doi:10.1371/journal.pmed1000097

For more information, visit www.prisma-statement.org.

Peptic Ulcer Disease

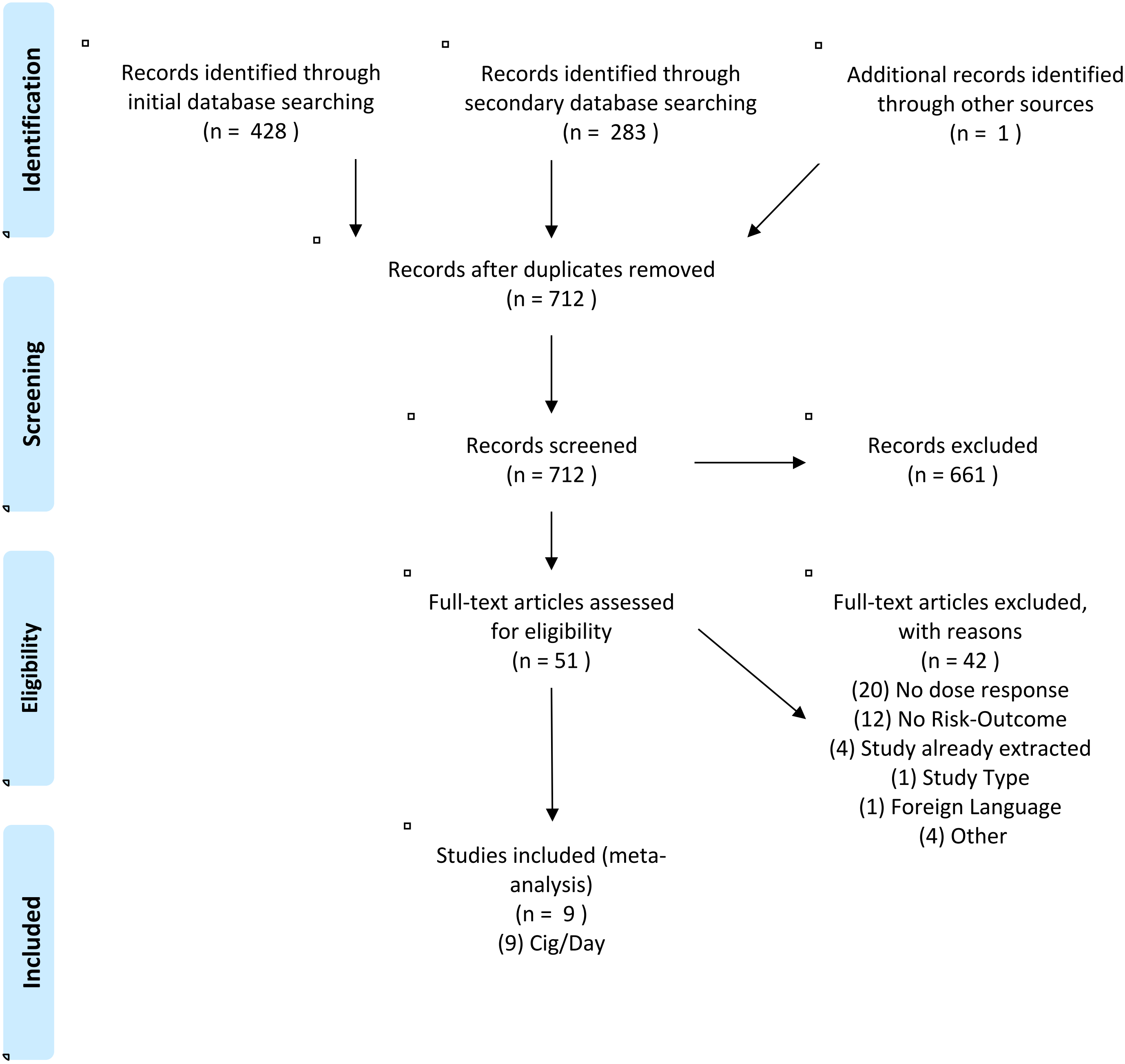

From: Moher D, Liberati A, Tetzlaff J, Altman DG, The PRISMA Group (2009). *P*referred *R*eporting *I*tems for *S*ystematic Reviews and *M*eta-*A*nalyses: The PRISMA Statement. PLoS Med 6(7): e1000097. doi:10.1371/journal.pmed1000097

For more information, visit www.prisma-statement.org.

Peripheral Artery Disease

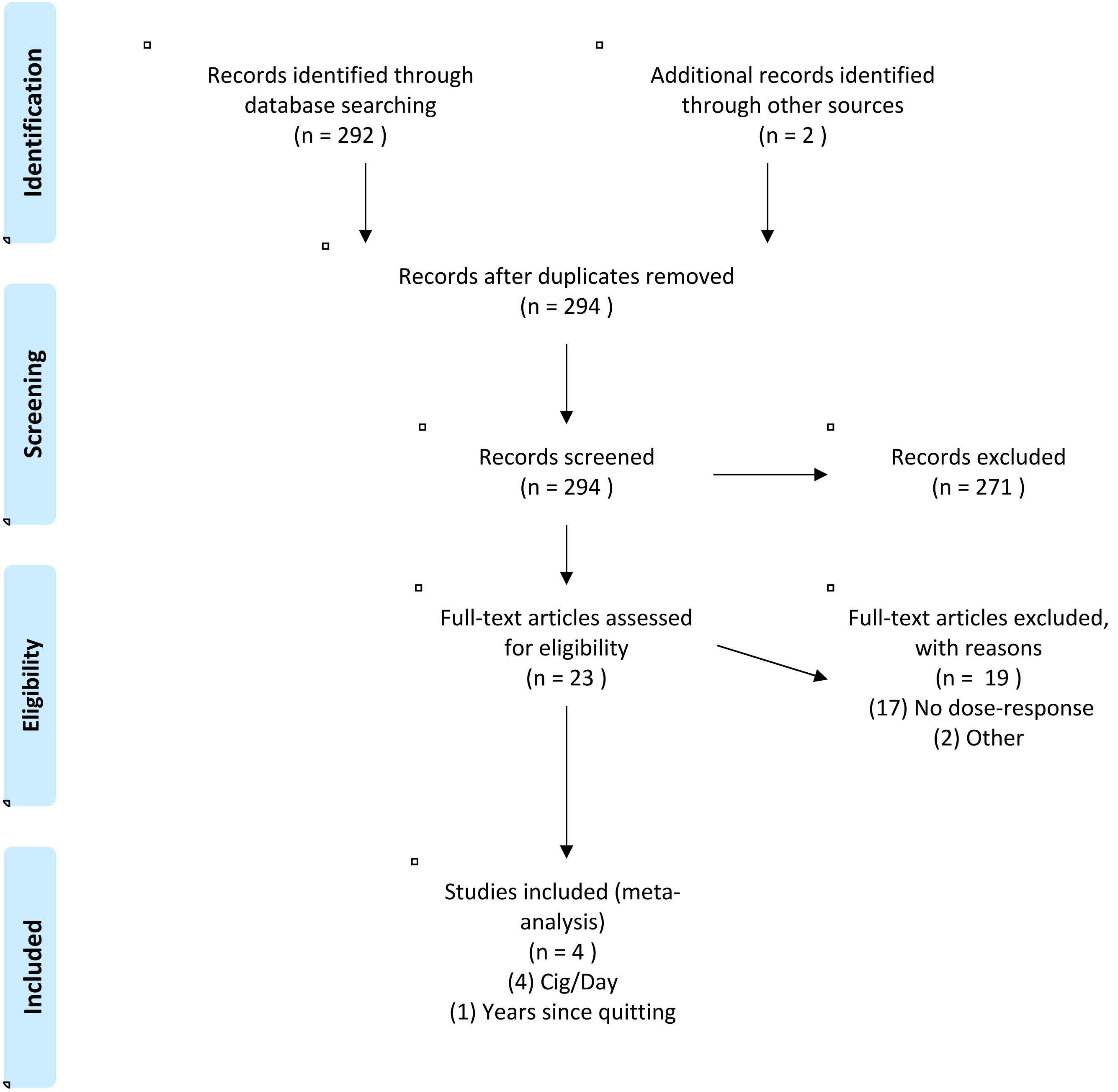

From: Moher D, Liberati A, Tetzlaff J, Altman DG, The PRISMA Group (2009). *P*referred *R*eporting *I*tems for *S*ystematic Reviews and *M*eta-*A*nalyses: The PRISMA Statement. PLoS Med 6(7): e1000097. doi:10.1371/journal.pmed1000097

For more information, visit www.prisma-statement.org.

Prostate Cancer

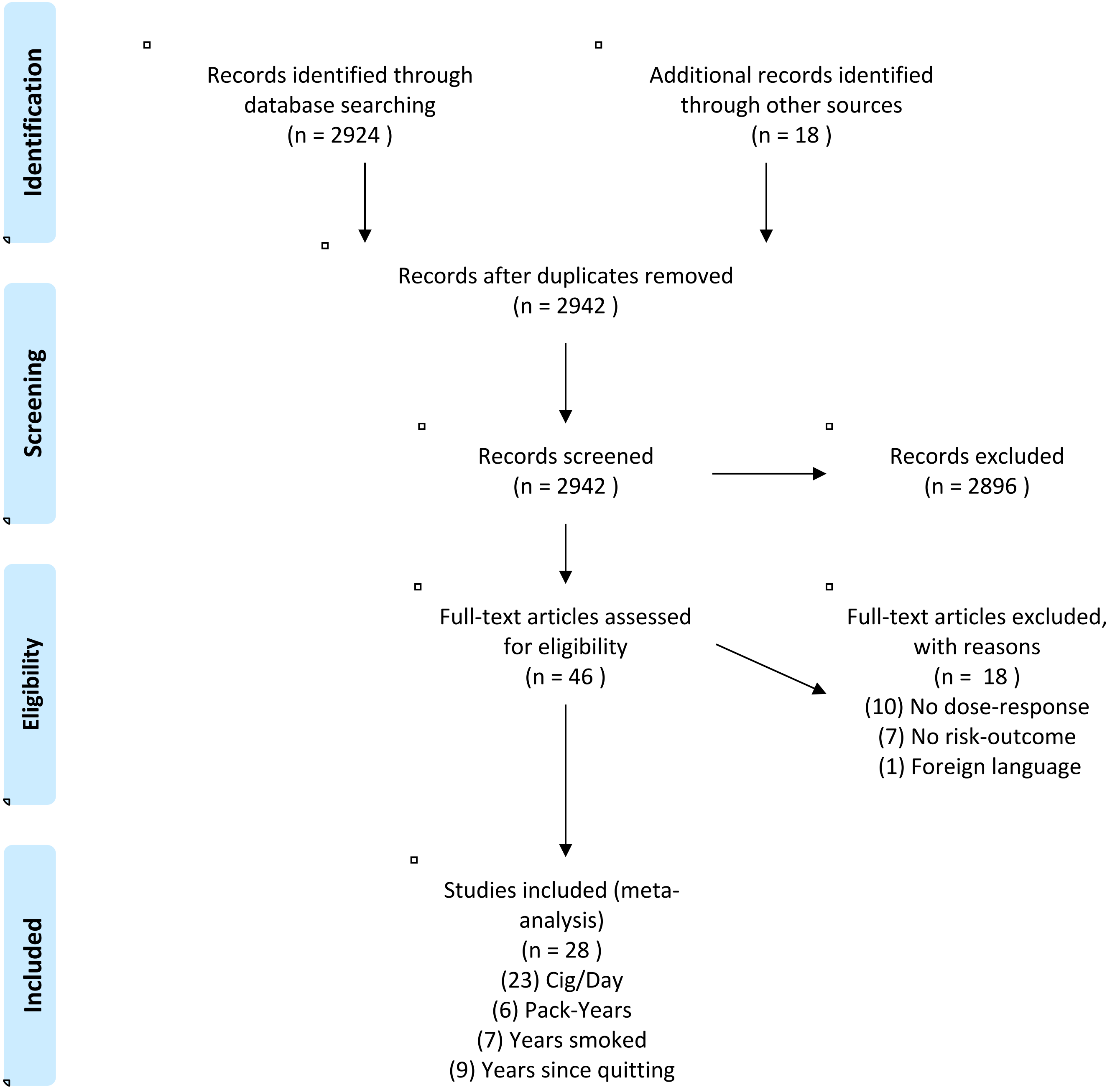

From: Moher D, Liberati A, Tetzlaff J, Altman DG, The PRISMA Group (2009). *P*referred *R*eporting *I*tems for *S*ystematic Reviews and *M*eta-*A*nalyses: The PRISMA Statement. PLoS Med 6(7): e1000097. doi:10.1371/journal.pmed1000097

For more information, visit www.prisma-statement.org.

Rheumatoid Arthritis

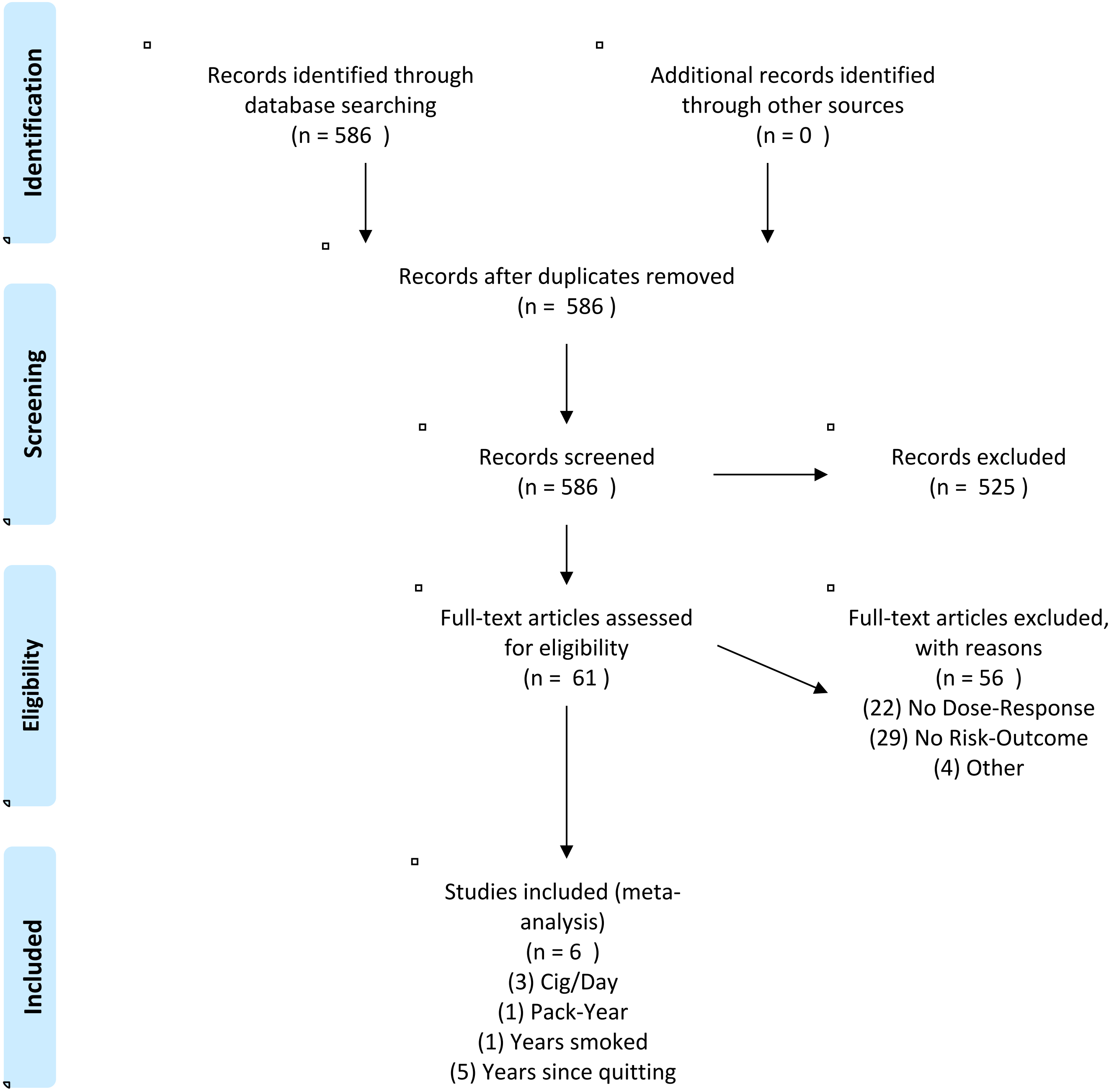

From: Moher D, Liberati A, Tetzlaff J, Altman DG, The PRISMA Group (2009). *P*referred *R*eporting *I*tems for *S*ystematic Reviews and *M*eta-*A*nalyses: The PRISMA Statement. PLoS Med 6(7): e1000097. doi:10.1371/journal.pmed1000097

For more information, visit www.prisma-statement.org.

Stomach Cancer

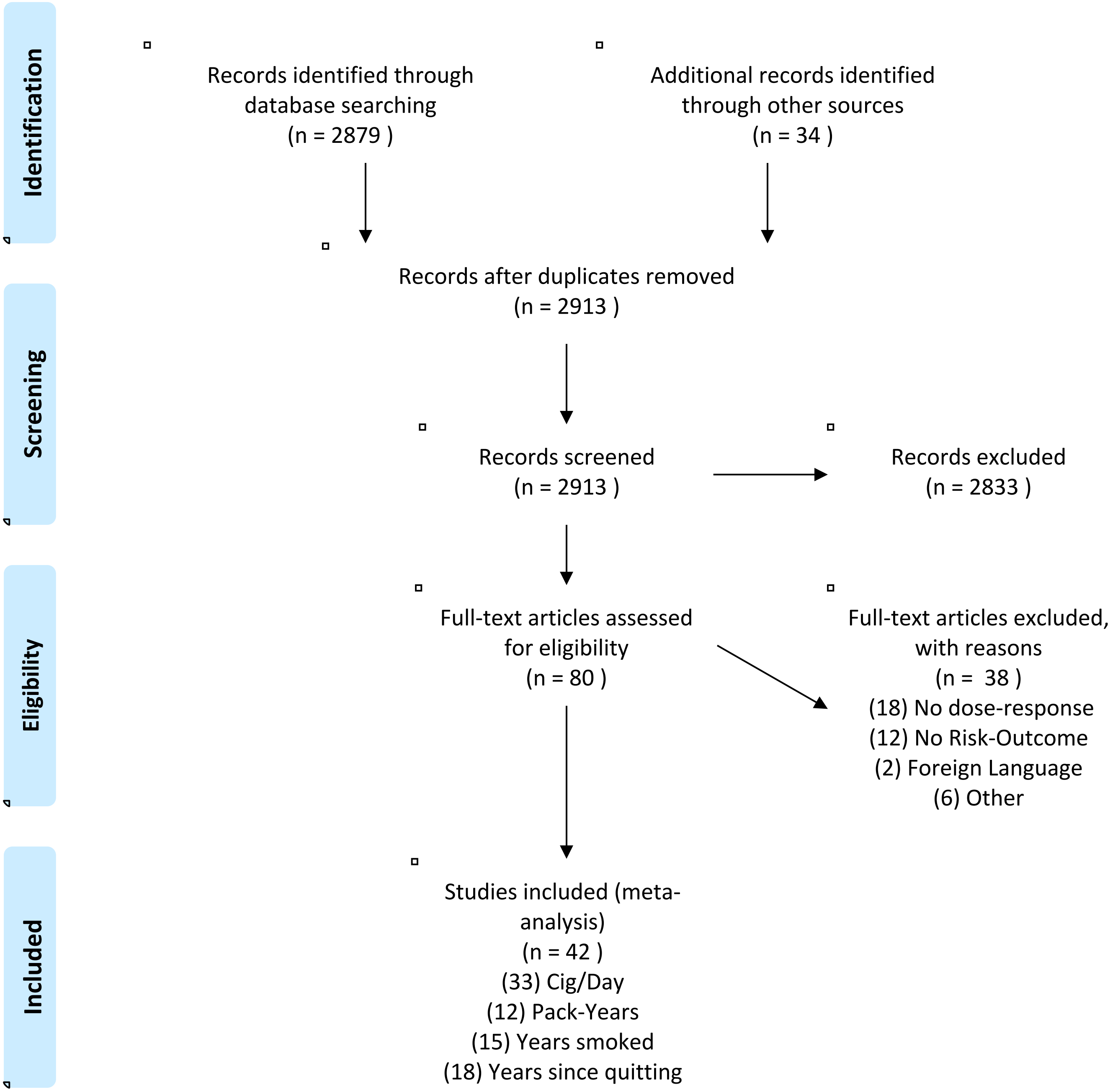

From: Moher D, Liberati A, Tetzlaff J, Altman DG, The PRISMA Group (2009). *P*referred *R*eporting *I*tems for *S*ystematic Reviews and *M*eta-*A*nalyses: The PRISMA Statement. PLoS Med 6(7): e1000097. doi:10.1371/journal.pmed1000097

For more information, visit www.prisma-statement.org.

Stroke

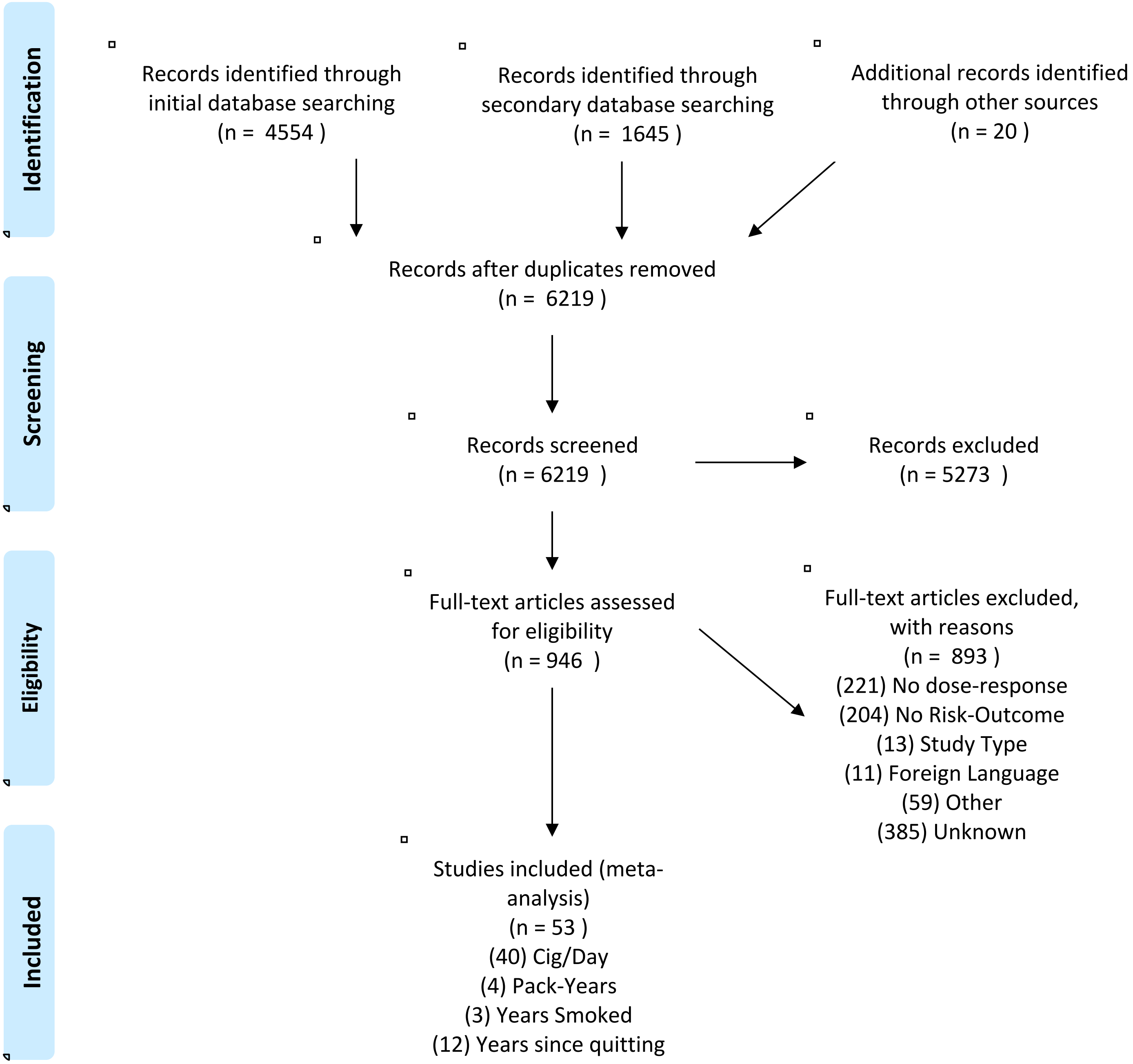

From: Moher D, Liberati A, Tetzlaff J, Altman DG, The PRISMA Group (2009). *P*referred *R*eporting *I*tems for *S*ystematic Reviews and *M*eta-*A*nalyses: The PRISMA Statement. PLoS Med 6(7): e1000097. doi:10.1371/journal.pmed1000097

For more information, visit www.prisma-statement.org.

Tuberculosis

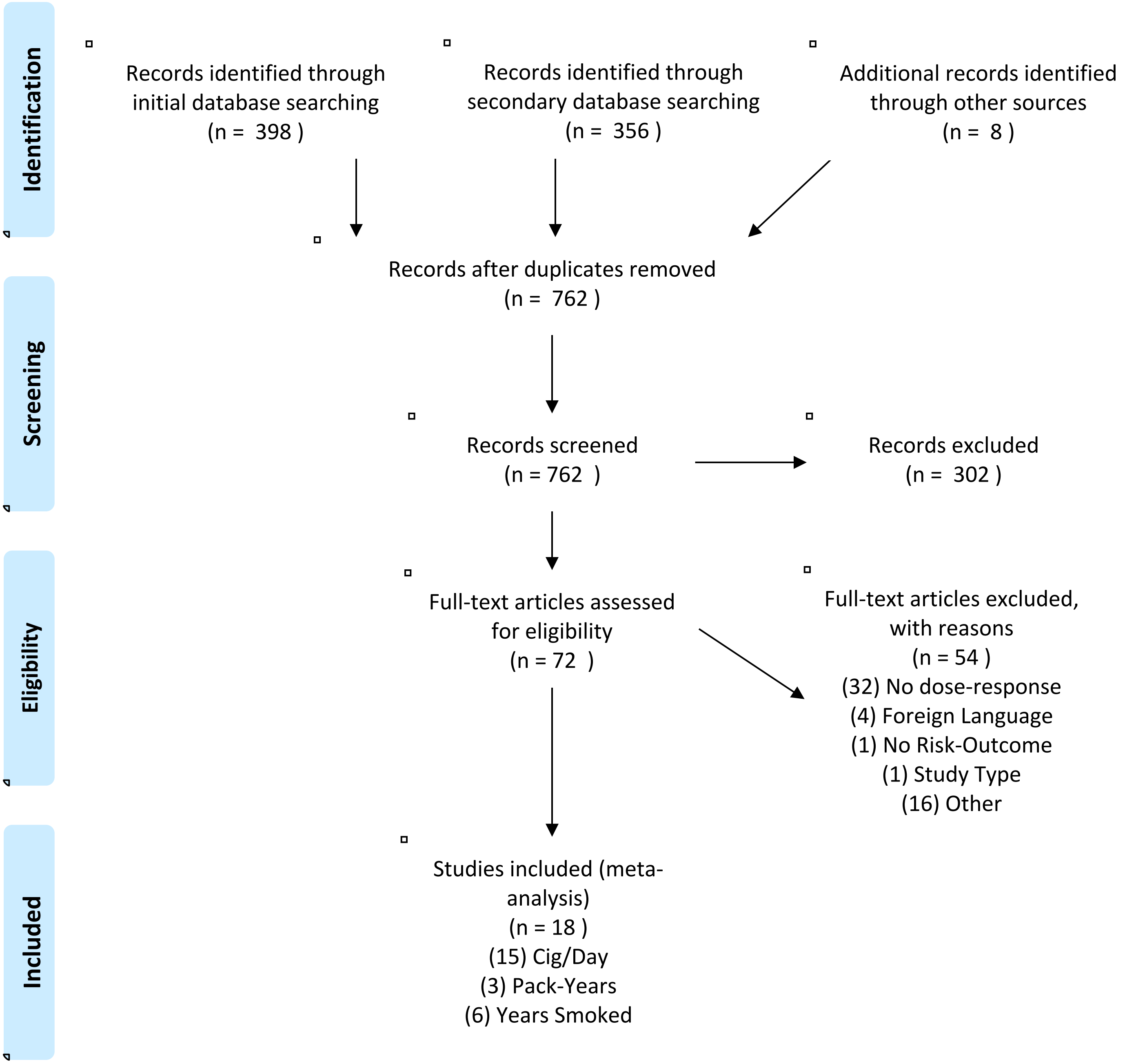

From: Moher D, Liberati A, Tetzlaff J, Altman DG, The PRISMA Group (2009). *P*referred *R*eporting *I*tems for *S*ystematic Reviews and *M*eta-*A*nalyses: The PRISMA Statement. PLoS Med 6(7): e1000097. doi:10.1371/journal.pmed1000097

For more information, visit www.prisma-statement.org.

Author Contributions

Managing the estimation or publications process
Erin C Mullany, Christopher J L Murray, and Emmanuela Gakidou.

Writing the first draft of the manuscript

Marissa B Reitsma, Simon I Hay, and Emmanuela Gakidou.

Primary responsibility for applying analytical methods to produce estimates

Marissa B Reitsma, Parkes J Kendrick, Xiaochen Dai, Luisa Sorio Flor, Thomas Hsiao, Hayley D Tymeson, and Rachel L Updike.

Primary responsibility for seeking, cataloguing, extracting, or cleaning data; designing or coding figures and tables

Marissa B Reitsma, Parkes J Kendrick, Jason A Anderson, Nicholas D Arian, Xiaochen Dai, Luisa Sorio Flor, Thomas Hsiao, Rachel Feldman, Hayley D Tymeson, Rachel L Updike, and Joanna L Whisnant.

Providing data or critical feedback on data sources

Marissa B Reitsma, Parkes J Kendrick, Cristiana Abbafati, Amir Abdoli, E S Abhilash, Derrick Bary Abila, Niveen ME Abu-Rmeileh, Oladimeji M Adebayo, Mohammad Aghaali, Bright Opoku Ahinkorah, Haroon Ahmed, Budi Aji, Chisom Joyqueenet Akunna, Turki M Alanzi, Khalid F Alhabib, Liaqat Ali, Sheikh Mohammad Alif, François Alla, Peter Allebeck, Nelson Alvis-Guzman, Saeed Amini, Hubert Amu, Carl Abelardo T Antonio, Davood Anvari, Jalal Arabloo, Marcel Ausloos, Asma Tahir Awan, Getie Lake Aynalem, Samad Azari, Maciej Banach, Palash Chandra Banik, Till Winfried Bärnighausen, Hiba Jawdat Barqawi, Sanjay Basu, Derrick A Bennett, Akshaya Srikanth Bhagavathula, Sadia Bibi, Bagas Suryo Bintoro, Dejana Braithwaite, Katrin Burkart, Florentino Luciano Caetano dos Santos, Luis Alberto Cámera, Joao Mauricio Castaldelli-Maia, Dinh-Toi Chu, Massimo Cirillo, Vera Marisa Costa, Albertino Antonio Moura Damasceno, Giovanni Damiani, Lalit Dandona, Rakhi Dandona, Kairat Davletov, Meseret Derbew Molla, Getenet Ayalew Dessie, Samath Dhamminda Dharmaratne, Mostafa Dianatinasab, Hoa Thi Do, Abdel Douiri, Bruce B Duncan, Andre Rodrigues Duraes, Ibtihal Fadhil, Emerito Jose A Faraon, Carla Sofia e Sá Farinha, Farshad Farzadfar, Carlota Fernandez Prendes, Irina Filip, Mohamed M Gad, Silvano Gallus, Alireza Ghajar, Ahmad Ghashghaee, Simona Giampaoli, Mahaveer Golechha, Sameer Vali Gopalani, Houman Goudarzi, Felix Greaves, Avirup Guha, Bhawna Gupta, Tarun Gupta, Nima Hafezi-Nejad, Hadi Hassankhani, Claudiu Herteliu, Kamal Hezam, Thomas R Hird, Mehdi Hosseinzadeh, Mowafa Househ, Segun Emmanuel Ibitoye, Bulat Idrisov, Seyed Sina Naghibi Irvani, Rakibul M Islam, Sheikh Mohammed Shariful Islam, Hiroyasu Iso, Ramaiah Itumalla, Jalil Jaafari, Mihajlo Jakovljevic, Sung-In Jang, Shubha Jayaram, Panniyammakal Jeemon, Jost B Jonas, Tamas Joo, Mikk Jürisson, Zubair Kabir, Leila R Kalankesh, Srinivasa Vittal Katikireddi, Bayew Kelkay, Abdullah T Khoja, Gyu Ri Kim, Young-Eun Kim, Mika Kivimäki, Soewarta Kosen, Kewal Krishan, Nuworza Kugbey, G Anil Kumar, Om P Kurmi, Dian Kusuma, Savita Lasrado, Doo Woong Lee, Yo Han Lee, Janni Leung, Wei Liu, Alan D Lopez, Stefan Lorkowski, Reza Malekzadeh, Deborah Carvalho Malta, Borhan Mansouri, Mohammad Ali Mansournia, Manu Raj Mathur, Carlo Eduardo Medina-Solís, Walter Mendoza, Ritesh G Menezes, Atte Meretoja, Irmina Maria Michalek, Erkin M Mirrakhimov, Masoud Moghadaszadeh, Abdollah Mohammadian-Hafshejani, Shafiu Mohammed, Ali H Mokdad, Lorenzo Monasta, Ghobad Moradi, Maziar Moradi-Lakeh, Tilahun Belete

Mossie, Sumaira Mubarik, Christopher J L Murray, Mohsen Naghavi, Sanjeev Nair, Mahdi Nalini, Sreenivas Narasimha Swamy, Javad Nazari, Cuong Tat Nguyen, Huong Lan Thi Nguyen, Son Hoang Nguyen, Trang Huyen Nguyen, Chukwudi A Nnaji, Bo Norrving, Jean Jacques Noubiap, Christoph Nowak, Felix Akpojene Ogbo, Ayodipupo Sikiru Oguntade, Andrew T Olagunju, Mayowa O Owolabi, Mahesh P A, Keyvan Pakshir, Raffaele Palladino, Adrian Pana, Songhomitra Panda-Jonas, Eun-Cheol Park, Fatemeh Pashazadeh Kan, Shrikant Pawar, Saeed Pirouzpanah, Khem Narayan Pokhrel, Dimas Ria Angga Pribadi, Amir Radfar, Vafa Rahimi-Movaghar, Azizur Rahman, Mohammad Hifz Ur Rahman, Amir Masoud Rahmani, Pradhum Ram, Chhabi Lal Ranabhat, Lal Rawal, Andre M N Renzaho, Luz Myriam Reynales-Shigematsu, Seyed Mohammad Riahi, Leonardo Roever, Luca Ronfani, Gholamreza Roshandel, Ambuj Roy, Basema Saddik, Mehrnoosh Samaei, Abdallah M Samy, Milena M Santric-Milicevic, Brijesh Sathian, Monika Sawhney, Mete Saylan, Maria Inês Schmidt, Ione Jayce Ceola Schneider, Abdul-Aziz Seidu, Sadaf G Sepanlou, Allen Seylani, Syed Mahboob Shah, Masood Ali Shaikh, Ali S Shalash, Mohammed Shannawaz, Sara Sheikhbahaei, K M Shivakumar, Roman Shrestha, Soraya Siabani, Negussie Boti Sidemo, Ambrish Singh, Jasvinder A Singh, Virendra Singh, Valentin Yurievich Skryabin, Anna Aleksandrovna Skryabina, Matiwos Soboka, Chandrashekhar T Sreeramareddy, Kurt Straif, Rizwan Suliankatchi Abdulkader, Takahiro Tabuchi, Biruk Wogayehu Taddele, Animut Tagele Tamiru, Mohamad-Hani Temsah, Musliu Adetola Tolani, Marcos Roberto Tovani-Palone, Bach Xuan Tran, Gebiyaw Wudie Tsegaye, Anayat Ullah, Saif Ullah, Pascual R Valdez, Constantine Vardavas, Tommi Juhani Vasankari, Narayanaswamy Venketasubramanian, Bay Vo, Giang Thu Vu, Yasir Waheed, Vahid Yazdi-Feyzabadi, Naohiro Yonemoto, Chuanhua Yu, Ismaeel Yunusa, Luis Zavala-Arciniega, and Yves Miel H Zuniga.

Development of methods or computational machinery

Marissa B Reitsma, Parkes J Kendrick, Liaqat Ali, Hubert Amu, Davood Anvari, Xiaochen Dai, Mostafa Dianatinasab, Mahaveer Golechha, Yuming Guo, Bhawna Gupta, Rajat Das Gupta, Mowafa Househ, Gyu Ri Kim, Young-Eun Kim, Shanshan Li, Alan D Lopez, Borhan Mansouri, Masoud Moghadaszadeh, Ali H Mokdad, Mohammad Ali Moni, Christopher J L Murray, Mohsen Naghavi, Eun-Cheol Park, Saeed Pirouzpanah, Azizur Rahman, Chhabi Lal Ranabhat, Seyed Mohammad Riahi, Abdallah M Samy, Allen Seylani, Negussie Boti Sidemo, Mikhail Sergeevich Zastrozhin, and Anasthasia Zastrozhina.

Providing critical feedback on methods or results

Marissa B Reitsma, Parkes J Kendrick, Cristiana Abbafati, Aidin Abedi, E S Abhilash, Derrick Bary Abila, Victor Aboyans, Oladimeji M Adebayo, Shailesh M Advani, Mohammad Aghaali, Bright Opoku Ahinkorah, Sohail Ahmad, Keivan Ahmadi, Haroon Ahmed, Budi Aji, Chisom Joyqueenet Akunna, Ziyad Al-Aly, Turki M Alanzi, Khalid F Alhabib, Liaqat Ali, Sheikh Mohammad Alif, Vahid Alipour, Syed Mohamed Aljunid, Peter Allebeck, Nelson Alvis-Guzman, Tarek Tawfik Amin, Saeed Amini, Hubert Amu, Gianna Gayle Herrera Amul, Robert Ancuceanu, Alireza Ansari-Moghaddam, Benny Antony, Davood Anvari, Jalal Arabloo, Marcel Ausloos, Asma Tahir Awan, Getinet Ayano, Getie Lake Aynalem, Samad Azari, Darshan B B, Ashish D Badiye, Atif Amin Baig, Maciej Banach, Palash Chandra Banik, Till Winfried Bärnighausen, Hiba Jawdat Barqawi, Sanjay Basu, Mohsen Bayati, Shahrzad Bazargan-Hejazi, Masoud Behzadifar, Tariku Tesfaye Bekuma, Derrick A Bennett, Isabela M Bensenor, Akshaya Srikanth Bhagavathula, Nikha Bhardwaj, Pankaj Bhardwaj, Krittika Bhattacharyya, Sadia Bibi, Ali Bijani, Bagas Suryo Bintoro, Antonio

Biondi, Setognal Birara, Dejana Braithwaite, Andre R Brunoni, Katrin Burkart, Zahid A Butt, Florentino Luciano Caetano dos Santos, Luis Alberto Cámera, Josip Car, Rosario Cárdenas, Giulia Carreras, Juan J Carrero, Joao Mauricio Castaldelli-Maia, Jung-Chen Chang, Dinh-Toi Chu, Sheng-Chia Chung, Vera Marisa Costa, Omid Dadras, Xiaochen Dai, Giovanni Damiani, Lalit Dandona, Rakhi Dandona, Parnaz Daneshpajouhnejad, Jiregna Darega Gela, Kairat Davletov, Meseret Derbew Molla, Getenet Ayalew Dessie, Abebaw Alemayehu Desta, Samath Dhamminda Dharmaratne, Mostafa Dianatinasab, Daniel Diaz, Hoa Thi Do, Abdel Douiri, Andre Rodrigues Duraes, Arielle Wilder Eagan, Mohammad Ebrahimi Kalan, Kristina Edvardsson, Saman Esmaeilnejad, Emerito Jose A Faraon, Carla Sofia e Sá Farinha, Medhat Farwati, Farshad Farzadfar, Mehdi Fazlzadeh, Valery L Feigin, Carlota Fernandez Prendes, Pietro Ferrara, Irina Filip, Filippos Filippidis, Florian Fischer, Nataliya A Foigt, Morenike Oluwatoyin Folayan, Masoud Foroutan, Mohamed M Gad, Abhay Motiramji Gaidhane, Biniyam Sahiledengle Geberemariyam, Mansour Ghafourifard, Alireza Ghajar, Ahmad Ghashghaee, Paramjit Singh Gill, Franklin N Glozah, Elena V Gnedovskaya, Mahaveer Golechha, Sameer Vali Gopalani, Houman Goudarzi, Alessandra C Goulart, Felix Greaves, Avirup Guha, Yuming Guo, Bhawna Gupta, Rajat Das Gupta, Rajeev Gupta, Tarun Gupta, Nima Hafezi-Nejad, Mohammad Rifat Haider, Randah R Hamadeh, Arief Hargono, Risky Kusuma Hartono, Hadi Hassankhani, Golnaz Heidari, Claudiu Herteliu, Thomas R Hird, Ramesh Holla, Mehdi Hosseinzadeh, Sorin Hostiuc, Mowafa Househ, Segun Emmanuel Ibitoye, Bulat Idrisov, Olayinka Stephen Ilesanmi, Irena M Ilic, Milena D Ilic, Leeberk Raja Inbaraj, Seyed Sina Naghibi Irvani, Jessica Y Islam, Rakibul M Islam, Sheikh Mohammed Shariful Islam, Farhad Islami, Ramaiah Itumalla, Masao Iwagami, Jalil Jaafari, Vardhmaan Jain, Mihajlo Jakovljevic, Sung-In Jang, Hosna Janjani, Shubha Jayaram, Panniyammakal Jeemon, Ravi Prakash Jha, Jost B Jonas, Tamas Joo, Mikk Jürisson, Ali Kabir, Zubair Kabir, Leila R Kalankesh, Tanuj Kanchan, Himal Kandel, Neeti Kapoor, Salah Eddin Karimi, Srinivasa Vittal Katikireddi, Hafte Kahsay Kebede, Bayew Kelkay, Abdullah T Khoja, Jagdish Khubchandani, Gyu Ri Kim, Young-Eun Kim, Ruth W Kimokoti, Mika Kivimäki, Sindhura Lakshmi Koulmane Laxminarayana, Ai Koyanagi, Kewal Krishan, Nuworza Kugbey, G Anil Kumar, Nithin Kumar, Om P Kurmi, Dian Kusuma, Jennifer O Lam, Savita Lasrado, Paolo Lauriola, Doo Woong Lee, Yo Han Lee, Shanshan Li, Hualiang Lin, Shai Linn, Wei Liu, Stefan Lorkowski, Azeem Majeed, Reza Malekzadeh, Deborah Carvalho Malta, Abdullah A Mamun, Borhan Mansouri, Mohammad Ali Mansournia, Jose Martinez-Raga, Santi Martini, Manu Raj Mathur, Carlo Eduardo Medina-Solís, Suresh Mehata, Walter Mendoza, Ritesh G Menezes, Atte Meretoja, Tuomo J Meretoja, Bartosz Miazgowski, Irmina Maria Michalek, Ted R Miller, Erkin M Mirrakhimov, Hamed Mirzaei, Mehdi Mirzaei-Alavijeh, Sanjeev Misra, Masoud Moghadaszadeh, Yousef Mohammad, Abdollah Mohammadian-Hafshejani, Shafiu Mohammed, Ali H Mokdad, Mohammad Ali Moni, Ghobad Moradi, Maziar Moradi-Lakeh, Tilahun Belete Mossie, Sumaira Mubarik, Christopher J L Murray, Mohsen Naghavi, Sanjeev Nair, Mahdi Nalini, Vinay Nangia, Atta Abbas Naqvi, Sreenivas Narasimha Swamy, Muhammad Naveed, Javad Nazari, Sandhya Neupane Kandel, Cuong Tat Nguyen, Huong Lan Thi Nguyen, Son Hoang Nguyen, Trang Huyen Nguyen, Chukwudi A Nnaji, Bo Norrving, Jean Jacques Noubiap, Christoph Nowak, Felix Akpojene Ogbo, Ayodipupo Sikiru Oguntade, Andrew T Olagunju, Eyal Oren, Nikita Otstavnov, Stanislav S Otstavnov, Mayowa O Owolabi, Mahesh P A, Smita Pakhale, Raffaele Palladino, Adrian Pana, Songhomitra Panda-Jonas, Ashok Pandey, Utsav Parekh, Eun-Cheol Park, Eun-Kee Park, Fatemeh Pashazadeh Kan, Shrikant Pawar, Richard G Pestell, Michael A Piradov, Saeed Pirouzpanah, Khem Narayan Pokhrel, Akila Prashant, Amir Radfar, Vafa Rahimi-Movaghar, Azizur Rahman, Mohammad Hifz Ur Rahman, Amir Masoud Rahmani, Nazanin Rajai, Pradhum Ram, Chhabi Lal Ranabhat, Priya Rathi, Lal Rawal, Andre M N Renzaho, Aziz Rezapour, Seyed Mohammad Riahi, Mavra A Riaz, Leonardo Roever, Gholamreza Roshandel, Bedanta Roy, Simona Sacco,

Basema Saddik, Sana Salehi, Mehrnoosh Samaei, Abdallah M Samy, Itamar S Santos, Milena M Santric-Milicevic, Nizal Sarrafzadegan, Brijesh Sathian, Monika Sawhney, Mete Saylan, Michael P Schaub, Ione Jayce Ceola Schneider, Aletta Elisabeth Schutte, Falk Schwendicke, Nachimuthu Senthil Kumar, Sadaf G Sepanlou, Allen Seylani, Omid Shafaat, Syed Mahboob Shah, Masood Ali Shaikh, Ali S Shalash, Mohammed Shannawaz, Kiomars Sharafi, Aziz Sheikh, Sara Sheikhbahaei, Mika Shigematsu, Rahman Shiri, Kawkab Shishani, K M Shivakumar, Siddharudha Shivalli, Roman Shrestha, Soraya Siabani, Negussie Boti Sidemo, Diego Augusto Santos Silva, João Pedro Silva, Ambrish Singh, Jasvinder A Singh, Dhirendra Narain Sinha, Freddy Sitas, Valentin Yurievich Skryabin, Anna Aleksandrovna Skryabina, Matiwos Soboka, Joan B Soriano, Ali Soroush, Sergey Soshnikov, Ireneous N Soyiri, Chandrashekhar T Sreeramareddy, Paschalis Steiropoulos, Stefan Stortecky, Kurt Straif, Rizwan Suliankatchi Abdulkader, Gerhard Sulo, Santosh Kumar Tadakamadla, Eyayou Girma Tadesse, Animut Tagele Tamiru, Minale Tareke, Md Ismail Tareque, Ingan Ukur Tarigan, Mohamad-Hani Temsah, Rekha Thapar, Ales Tichopad, Musliu Adetola Tolani, Fotis Topouzis, Marcos Roberto Tovani-Palone, Bach Xuan Tran, Jaya Prasad Tripathy, Gebiyaw Wudie Tsegaye, Nikolaos Tsilimparis, Anayat Ullah, Saif Ullah, Marco Vacante, Pascual R Valdez, Constantine Vardavas, Narayanaswamy Venketasubramanian, Madhur Verma, Marina V Vetrova, Bay Vo, Giang Thu Vu, Yasir Waheed, Yanzhong Wang, Andrea Werdecker, Joanna L Whisnant, Nuwan Darshana Wickramasinghe, Kazumasa Yamagishi, Srikanth Yandrapalli, Vahid Yazdi-Feyzabadi, Yigizie Yeshaw, Naohiro Yonemoto, Chuanhua Yu, Ismaeel Yunusa, Hasan Yusefzadeh, Telma Zahirian Moghadam, Muhammed Shahriar Zaman, Maryam Zamanian, Hamed Zandian, Heather J Zar, Mikhail Sergeevich Zastrozhin, Anasthasia Zastrozhina, Jianrong Zhang, and Yves Miel H Zuniga.

Drafting the manuscript or revising it critically for important intellectual content

Marissa B Reitsma, Parkes J Kendrick, Emad Ababneh, Mohsen Abbasi-Kangevari, Aidin Abedi, Derrick Bary Abila, Niveen ME Abu-Rmeileh, Oladimeji M Adebayo, Shailesh M Advani, Mohammad Aghaali, Bright Opoku Ahinkorah, Sohail Ahmad, Budi Aji, Liaqat Ali, Peter Allebeck, Nelson Alvis-Guzman, Tarek Tawfik Amin, Saeed Amini, Hubert Amu, Robert Ancuceanu, Carl Abelardo T Antonio, Benny Antony, Jalal Arabloo, Monika Arora, Malke Asaad, Marcel Ausloos, Getie Lake Aynalem, Darshan B B, Ashish D Badiye, Atif Amin Baig, Mohammad Hossein Bakhshaei, Maciej Banach, Palash Chandra Banik, Suzanne Lyn Barker-Collo, Till Winfried Bärnighausen, Hiba Jawdat Barqawi, Sanjay Basu, Masoud Behzadifar, Isabela M Bensenor, Kathleen S Sachiko Berfield, Akshaya Srikanth Bhagavathula, Krittika Bhattacharyya, Sadia Bibi, Antonio Biondi, Dejana Braithwaite, Hermann Brenner, Florentino Luciano Caetano dos Santos, Josip Car, Juan J Carrero, Joao Mauricio Castaldelli-Maia, Maria Sofia Sofia Cattaruzza, Simiao Chen, Dinh-Toi Chu, Vera Marisa Costa, Rosa A S Couto, Xiaochen Dai, Albertino Antonio Moura Damasceno, Giovanni Damiani, Meseret Derbew Molla, Samath Dhamminda Dharmaratne, Mostafa Dianatinasab, Daniel Diaz, Hoa Thi Do, Abdel Douiri, Bruce B Duncan, Arielle Wilder Eagan, Mohammad Ebrahimi Kalan, Kristina Edvardsson, Iffat Elbarazi, Maha El Tantawi, Saman Esmaeilnejad, Emerito Jose A Faraon, Medhat Farwati, Farshad Farzadfar, Carlota Fernandez Prendes, Pietro Ferrara, Irina Filip, Florian Fischer, Nataliya A Foigt, Morenike Oluwatoyin Folayan, Masoud Foroutan, Mohamed M Gad, Silvano Gallus, Biniyam Sahiledengle Geberemariyam, Mansour Ghafourifard, Alireza Ghajar, Ahmad Ghashghaee, Elena V Gnedovskaya, Sameer Vali Gopalani, Giuseppe Gorini, Alessandra C Goulart, Felix Greaves, Yuming Guo, Bhawna Gupta, Rajat Das Gupta, Rajeev Gupta, Vin Gupta, Nima Hafezi-Nejad,

Randah R Hamadeh, Graeme J Hankey, Simon I Hay, Claudiu Herteliu, Kamal Hezam, Thomas R Hird, Michael K Hole, Ramesh Holla, Mowafa Househ, Junjie Huang, Segun Emmanuel Ibitoye, Bulat Idrisov, Irena M Ilic, Milena D Ilic, Seyed Sina Naghibi Irvani, Jessica Y Islam, Rakibul M Islam, Sheikh Mohammed Shariful Islam, Farhad Islami, Vardhmaan Jain, Mihajlo Jakovljevic, Sung-In Jang, Shubha Jayaram, Ravi Prakash Jha, Jost B Jonas, Himal Kandel, Neeti Kapoor, Srinivasa Vittal Katikireddi, Bayew Kelkay, Jagdish Khubchandani, Gyu Ri Kim, Young-Eun Kim, Mika Kivimäki, Sindhura Lakshmi Koulmane Laxminarayana, Ai Koyanagi, Kewal Krishan, Nuworza Kugbey, Om P Kurmi, Dian Kusuma, Ben Lacey, Jennifer O Lam, Iván Landires, Savita Lasrado, Doo Woong Lee, Janni Leung, Shanshan Li, Hualiang Lin, Wei Liu, Platon D Lopukhov, Stefan Lorkowski, Alessandra Lugo, Azeem Majeed, Afshin Maleki, Reza Malekzadeh, Deborah Carvalho Malta, Abdullah A Mamun, Narayana Manjunatha, Borhan Mansouri, Jose Martinez-Raga, Manu Raj Mathur, Carlo Eduardo Medina-Solís, Walter Mendoza, Ritesh G Menezes, Atte Meretoja, Tuomo J Meretoja, Irmina Maria Michalek, Ted R Miller, Masoud Moghadaszadeh, Yousef Mohammad, Abdollah Mohammadian-Hafshejani, Shafiu Mohammed, Ali H Mokdad, Lorenzo Monasta, Mohammad Ali Moni, Maziar Moradi-Lakeh, Rahmatollah Moradzadeh, Shane Douglas Morrison, Tilahun Belete Mossie, Sumaira Mubarik, Christopher J L Murray, Behshad Naghshtabrizi, Sanjeev Nair, Mahdi Nalini, Vinay Nangia, Sreenivas Narasimha Swamy, Smitha Nayak, Vinod C Nayak, Javad Nazari, Sabina O Nduaguba, Sandhya Neupane Kandel, Cuong Tat Nguyen, Huong Lan Thi Nguyen, Son Hoang Nguyen, Trang Huyen Nguyen, Molly R Nixon, Bo Norrving, Jean Jacques Noubiap, Christoph Nowak, Felix Akpojene Ogbo, In-Hwan Oh, Andrew T Olagunju, Eyal Oren, Nikita Otstavnov, Stanislav S Otstavnov, Mayowa O Owolabi, Mahesh P A, Smita Pakhale, Adrian Pana, Songhomitra Panda-Jonas, Eun-Cheol Park, Eun-Kee Park, Fatemeh Pashazadeh Kan, George C Patton, Shrikant Pawar, Richard G Pestell, Marina Pinheiro, Michael A Piradov, Saeed Pirouzpanah, Roman V Polibin, Amir Radfar, Vafa Rahimi-Movaghar, Azizur Rahman, Mohammad Hifz Ur Rahman, Muhammad Aziz Rahman, Pradhum Ram, Chhabi Lal Ranabhat, Lal Rawal, Andre M N Renzaho, Seyed Mohammad Riahi, Mavra A Riaz, Leonardo Roever, Luca Ronfani, Bedanta Roy, Basema Saddik, Amirhossein Sahebkar, Hamideh Salimzadeh, Mehrnoosh Samaei, Abdallah M Samy, Itamar S Santos, Milena M Santric-Milicevic, Mete Saylan, Maria Inês Schmidt, Ione Jayce Ceola Schneider, Aletta Elisabeth Schutte, Falk Schwendicke, Nachimuthu Senthil Kumar, Sadaf G Sepanlou, Allen Seylani, Syed Mahboob Shah, Mohammed Shannawaz, Sara Sheikhbahaei, Mika Shigematsu, K M Shivakumar, Siddharudha Shivalli, Negussie Boti Sidemo, Inga Dora Sigfusdottir, Rannveig Sigurvinsdottir, Diego Augusto Santos Silva, João Pedro Silva, Jasvinder A Singh, Dhirendra Narain Sinha, Joan B Soriano, Ali Soroush, Ireneous N Soyiri, Chandrashekhar T Sreeramareddy, Dan J Stein, Paschalis Steiropoulos, Stefan Stortecky, Gerhard Sulo, Johan Sundström, Takahiro Tabuchi, Santosh Kumar Tadakamadla, Eyayou Girma Tadesse, Animut Tagele Tamiru, Mohamad-Hani Temsah, Kavumpurathu Raman Thankappan, Musliu Adetola Tolani, Marcos Roberto Tovani-Palone, Bach Xuan Tran, Jaya Prasad Tripathy, Nikolaos Tsilimparis, Anayat Ullah, Saif Ullah, Brigid Unim, Marco Vacante, Constantine Vardavas, Tommi Juhani Vasankari, Narayanaswamy Venketasubramanian, Giang Thu Vu, Yasir Waheed, Yanzhong Wang, Andrea Werdecker, Nuwan Darshana Wickramasinghe, Kazumasa Yamagishi, Hiroshi Yatsuya, Vahid Yazdi-Feyzabadi, Yigizie Yeshaw, Mohammed Zewdu Yimmer, Naohiro Yonemoto, Maryam Zamanian, Heather J Zar, Anasthasia Zastrozhina, Jianrong Zhang, Zhi-Jiang Zhang, and Chenwen Zhong.

Extracting, cleaning, or cataloging data; designing or coding figures and tables

Marissa B Reitsma, Parkes J Kendrick, Haroon Ahmed, Liaqat Ali, Hubert Amu, Jason A Anderson, Xiaochen Dai, Rachel Feldman, Vincent C Iannucci, Tamas Joo, Young-Eun Kim, Borhan Mansouri, Ted R Miller, Masoud Moghadaszadeh, Abdollah Mohammadian-Hafshejani, Ali H Mokdad, Christopher J L Murray, Mohsen Naghavi, Utsav Parekh, Shrikant Pawar, Leonardo Roever, Abdallah M Samy, Nachimuthu Senthil Kumar, Negussie Boti Sidemo, Emma Elizabeth Spurlock, and Joanna L Whisnant.

Management of the overall research enterprise

Lalit Dandona, Simon I Hay, Alan D Lopez, Ali H Mokdad, Erin C Mullany, Christopher J L Murray, Mohsen Naghavi, Molly R Nixon, and George C Patton.
