# Supplementary material for: Spatial, temporal, and demographic patterns in prevalence of smoking tobacco use and attributable disease burden in 204 countries and territories, 1990–2019: a systematic analysis from the Global Burden of Disease Study 2019
Source: Lancet. 2021 Jun 19;397(10292):2337–60. doi: 10.1016/S0140-6736(21)01169-7 (PMC8223261; doi:10.1016/S0140-6736(21)01169-7)
Supplement: Supplementary appendix 2 [file mmc2.pdf]

THE LANCET

Supplementary appendix

This appendix formed part of the original submission and has been peer reviewed. We post it as supplied by the authors.

Supplement to: GBD 2019 Tobacco Collaborators. Spatial, temporal, and demographic patterns in prevalence of smoking tobacco use and attributable disease burden in 204 countries and territories, 1990–2019: a systematic analysis from the Global Burden of Disease Study 2019. *Lancet* 2021; published online May 27. http://dx.doi.org/10.1016/S0140-6736(21)01169-7.

Supplementary Results

Spatial, temporal, and demographic patterns in prevalence of smoking tobacco use and attributable disease burden in 204 countries and territories, 1990-2019: a systematic analysis from the Global Burden of Disease Study 2019

GBD 2019 Tobacco Collaborators

Table of Contents

Tables and Figures *3*

Supplemental Table S1a. Results from dose-response meta-regressions for 36 health outcomes caused by smoking, at doses used as knots in estimation. 5

Supplemental Table S1b. Results from dose-response meta-regressions for 36 health outcomes caused by smoking, at standardized doses. 43

Supplemental Figure S1a. Age-standardized smoking prevalence among males ages 15 and above, 2019. 83

Supplemental Figure S1b. Age-standardized smoking prevalence among females ages 15 and above, 2019. 84

Supplemental Table S2. Number of current smokers (millions), by sex, in 2019, for 204 countries and territories. 85

Supplemental Table S3. Relative percent change in number of smokers and age-standardized smoking prevalence, between 1990 and 2019, for 204 countries and territories. 94

Supplemental Table S4. Cigarette-equivalents per person aged 15 years and older in 1990 and 2019, for 204 countries and territories. 103

Supplemental Table S5. All-cause and all-age smoking attributable deaths by location and sex, 2019. Estimates are reported as total number of attributable deaths, percent of deaths attributable to smoking, and smoking attributable death rate (per 100,000). 112

Supplemental Table S6. All-cause and all-age smoking attributable disability-adjusted life-years (DALYs) by location and sex, 2019. Estimates are reported as total number of attributable DALYs, percent of DALYs attributable to smoking, and smoking attributable DALY rate (per 100,000). 139

Supplemental Table S7. Ratio of smoking attributable years of life lost (YLLs) to years lived with disability (YLDs), by location for 2019. 166

Supplemental Figure S2. Ratio of smoking attributable years of life lost (YLLs) to years lived with disability (YLDs), by location and Socio-demographic Index level, in 1990 and 2019. Size of the point corresponds to the smoking-attributable disability-adjusted life-year rate. Colors correspond to the geographic region of the location. 175

Supplemental Table S8. Number of smoking attributable deaths for all causes and the top four causes of death (chronic obstructive pulmonary disease, ischaemic heart disease, stroke, and lung cancer), by region, in 2019. 176

Supplemental Figure S3. Global smoking attributable deaths (number, percent, rate per 100,000) by five-year age group for both sexes combined in 2019. 178

Supplemental Table S9. Percent change in number and share of all-age all-cause deaths attributable to smoking tobacco use, 1990-2019, by location. 179

Tables and Figures

Supplemental Table S1a. Results from dose-response meta-regressions for 36 health outcomes caused by smoking, at doses used as knots in estimation.

Supplemental Table S1b. Results from dose-response meta-regressions for 36 health outcomes caused by smoking, at standardized doses.

Supplemental Figure S1a. Age-standardized smoking prevalence among males ages 15 and above, 2019.

Supplemental Figure S1b. Age-standardized smoking prevalence among females ages 15 and above, 2019.

Supplemental Table S2. Number of current smokers (millions), by sex, in 2019, for 204 countries and territories.

Supplemental Table S3. Relative percent change in number of smokers and age-standardized smoking prevalence, between 1990 and 2019, for 204 countries and territories.

Supplemental Table S4. Cigarette-equivalents per person in 1990 and 2019, for 204 countries and territories.

Supplemental Table S5. All-cause and all-age smoking attributable deaths by location and sex, 2019. Estimates are reported as total number of attributable deaths, percent of deaths attributable to smoking, and smoking attributable death rate (per 100,000).

Supplemental Table S6. All-cause and all-age smoking attributable disability-adjusted life-years (DALYs) by location and sex, 2019. Estimates are reported as total number of attributable DALYs, percent of DALYs attributable to smoking, and smoking attributable DALY rate (per 100,000).

Supplemental Table S7. Ratio of smoking attributable years of life lost (YLLs) to years lived with disability (YLDs), by location for 2019.

Supplemental Figure S2. Ratio of smoking attributable years of life lost (YLLs) to years lived with disability (YLDs), by location and Socio-demographic Index level, in 1990 and 2019. Size of the point corresponds to the smoking-attributable disability-adjusted life-year rate. Colors correspond to the geographic region of the location.

Supplemental Table S8. Number of smoking attributable deaths for all causes and the top four causes of death (chronic obstructive pulmonary disease, ischaemic heart disease, stroke, and lung cancer), by region, in 2019.

Supplemental Figure S3. Global smoking attributable deaths (number, percent, rate per 100,000) by five-year age group for both sexes combined in 2019.

Supplemental Table S9. Percent change in number and share of all-age all-cause deaths attributable to smoking tobacco use, 1990-2019, by location.

Supplemental Table S1a. Results from dose-response meta-regressions for 36 health outcomes caused by smoking, at doses used as knots in estimation.

Health Outcome	Age Group	Sex	Dose	Relative Risk
Tuberculosis	All Ages	Both	0 Cigarette-Equivalents	1·00 (1·00–1·00)
Tuberculosis	All Ages	Both	9 Cigarette-Equivalents	2·07 (1·60–2·55)
Tuberculosis	All Ages	Both	18 Cigarette-Equivalents	2·31 (1·81–2·92)
Tuberculosis	All Ages	Both	27 Cigarette-Equivalents	3·03 (2·31–3·97)
Tuberculosis	All Ages	Both	36 Cigarette-Equivalents	4·01 (2·49–5·99)
Lower respiratory infections	All Ages	Both	0 Cigarette-Equivalents	1·00 (1·00–1·00)
Lower respiratory infections	All Ages	Both	1.5 Cigarette-Equivalents	1·32 (1·00–1·79)
Lower respiratory infections	All Ages	Both	10 Cigarette-Equivalents	1·96 (1·36–2·73)
Lower respiratory infections	All Ages	Both	19.5 Cigarette-Equivalents	2·43 (1·74–3·31)
Lower respiratory infections	All Ages	Both	31.2 Cigarette-Equivalents	3·16 (2·29–4·20)
Esophageal cancer	All Ages	Both	0 Pack-Years	1·00 (1·00–1·00)
Esophageal cancer	All Ages	Both	15 Pack-Years	3·04 (2·32–3·82)
Esophageal cancer	All Ages	Both	30 Pack-Years	2·78 (1·88–3·80)
Esophageal cancer	All Ages	Both	45 Pack-Years	4·47 (3·10–5·83)
Esophageal cancer	All Ages	Both	60 Pack-Years	5·33 (2·86–8·23)
Esophageal cancer	All Ages	Both	75 Pack-Years	7·33 (3·28–12·5)
Esophageal cancer	All Ages	Both	90 Pack-Years	9·59 (4·87–15·6)
Stomach cancer	All Ages	Both	0 Pack-Years	1·00 (1·00–1·00)
Stomach cancer	All Ages	Both	12.5 Pack-Years	1·43 (1·18–1·71)
Stomach cancer	All Ages	Both	25 Pack-Years	1·61 (1·29–1·99)
Stomach cancer	All Ages	Both	37.5 Pack-Years	1·91 (1·47–2·41)
Stomach cancer	All Ages	Both	50 Pack-Years	2·05 (1·56–2·54)
Stomach cancer	All Ages	Both	75 Pack-Years	2·12 (1·55–2·93)
Liver cancer	All Ages	Both	0 Pack-Years	1·00 (1·00–1·00)

Liver cancer	All Ages	Both	14.6 Pack-Years	1·51 (1·04–2·04)
Liver cancer	All Ages	Both	29.2 Pack-Years	1·64 (1·10–2·26)
Liver cancer	All Ages	Both	43.8 Pack-Years	1·83 (1·22–2·61)
Liver cancer	All Ages	Both	58.3 Pack-Years	1·79 (1·14–2·72)
Liver cancer	All Ages	Both	72.9 Pack-Years	1·94 (1·15–3·01)
Larynx cancer	All Ages	Both	0 Pack-Years	1·00 (1·00–1·00)
Larynx cancer	All Ages	Both	15 Pack-Years	4·59 (2·79–6·82)
Larynx cancer	All Ages	Both	30 Pack-Years	8·45 (5·06–12·9)
Larynx cancer	All Ages	Both	45 Pack-Years	16·2 (9·23–25·7)
Larynx cancer	All Ages	Both	60 Pack-Years	21·9 (10·6–36·7)
Larynx cancer	All Ages	Both	75 Pack-Years	26·1 (9·62–56·9)
Tracheal, bronchus, and lung cancer	All Ages	Both	0 Pack-Years	1·00 (1·00–1·00)
Tracheal, bronchus, and lung cancer	All Ages	Both	5 Pack-Years	1·76 (1·21–2·45)
Tracheal, bronchus, and lung cancer	All Ages	Both	14.3 Pack-Years	4·86 (3·92–5·87)
Tracheal, bronchus, and lung cancer	All Ages	Both	28.6 Pack-Years	8·90 (7·38–10·6)
Tracheal, bronchus, and lung cancer	All Ages	Both	42.9 Pack-Years	13·5 (10·5–16·8)
Tracheal, bronchus, and lung cancer	All Ages	Both	57.1 Pack-Years	14·8 (10·6–19·4)
Tracheal, bronchus, and lung cancer	All Ages	Both	71.4 Pack-Years	18·6 (12·8–25·6)
Tracheal, bronchus, and lung cancer	All Ages	Both	85.7 Pack-Years	21·5 (13·9–30·4)
Breast cancer	All Ages	Male	0 Pack-Years	1·00 (1·00–1·00)
Breast cancer	All Ages	Male	12.8 Pack-Years	1·00 (1·00–1·00)
Breast cancer	All Ages	Male	25.5 Pack-Years	1·00 (1·00–1·00)
Breast cancer	All Ages	Male	38.2 Pack-Years	1·00 (1·00–1·00)
Breast cancer	All Ages	Male	63.8 Pack-Years	1·00 (1·00–1·00)
Breast cancer	All Ages	Female	0 Pack-Years	1·00 (1·00–1·00)
Breast cancer	All Ages	Female	12.8 Pack-Years	1·21 (1·09–1·32)
Breast cancer	All Ages	Female	25.5 Pack-Years	1·31 (1·20–1·43)

Breast cancer	All Ages	Female	38.2 Pack-Years	1·24 (1·09–1·38)
Breast cancer	All Ages	Female	63.8 Pack-Years	1·27 (1·08–1·49)
Cervical cancer	All Ages	Both	0 Pack-Years	1·00 (1·00–1·00)
Cervical cancer	All Ages	Both	5 Pack-Years	1·79 (1·10–2·75)
Cervical cancer	All Ages	Both	7.4 Pack-Years	1·94 (1·17–2·98)
Cervical cancer	All Ages	Both	14.8 Pack-Years	2·25 (1·26–4·10)
Cervical cancer	All Ages	Both	22.1 Pack-Years	3·35 (1·72–6·12)
Cervical cancer	All Ages	Both	29.5 Pack-Years	4·21 (1·74–8·53)
Prostate cancer	All Ages	Male	0 Cigarette-Equivalents	1·00 (1·00–1·00)
Prostate cancer	All Ages	Male	7.5 Cigarette-Equivalents	1·19 (1·01–1·38)
Prostate cancer	All Ages	Male	15 Cigarette-Equivalents	1·17 (1·00–1·37)
Prostate cancer	All Ages	Male	22.5 Cigarette-Equivalents	1·17 (1·00–1·36)
Prostate cancer	All Ages	Male	30 Cigarette-Equivalents	1·24 (1·01–1·51)
Prostate cancer	All Ages	Male	37.5 Cigarette-Equivalents	1·33 (1·00–1·78)
Prostate cancer	All Ages	Female	0 Cigarette-Equivalents	1·00 (1·00–1·00)
Prostate cancer	All Ages	Female	7.5 Cigarette-Equivalents	1·00 (1·00–1·00)
Prostate cancer	All Ages	Female	15 Cigarette-Equivalents	1·00 (1·00–1·00)
Prostate cancer	All Ages	Female	22.5 Cigarette-Equivalents	1·00 (1·00–1·00)
Prostate cancer	All Ages	Female	30 Cigarette-Equivalents	1·00 (1·00–1·00)
Prostate cancer	All Ages	Female	37.5 Cigarette-Equivalents	1·00 (1·00–1·00)
Colon and rectum cancer	All Ages	Both	0 Pack-Years	1·00 (1·00–1·00)
Colon and rectum cancer	All Ages	Both	18.8 Pack-Years	1·50 (1·14–1·85)
Colon and rectum cancer	All Ages	Both	37.5 Pack-Years	1·61 (1·23–2·06)
Colon and rectum cancer	All Ages	Both	56.2 Pack-Years	1·58 (1·15–2·10)
Lip and oral cavity cancer	All Ages	Both	0 Pack-Years	1·00 (1·00–1·00)
Lip and oral cavity cancer	All Ages	Both	17.7 Pack-Years	3·19 (2·16–4·36)
Lip and oral cavity cancer	All Ages	Both	35.4 Pack-Years	3·75 (2·33–5·44)

Lip and oral cavity cancer	All Ages	Both	53.1 Pack-Years	4·02 (2·38–6·00)
Lip and oral cavity cancer	All Ages	Both	70.8 Pack-Years	5·52 (2·57–9·80)
Lip and oral cavity cancer	All Ages	Both	88.5 Pack-Years	7·27 (2·60–14·7)
Nasopharynx cancer	All Ages	Both	0 Pack-Years	1·00 (1·00–1·00)
Nasopharynx cancer	All Ages	Both	10.7 Pack-Years	1·88 (1·34–2·52)
Nasopharynx cancer	All Ages	Both	21.4 Pack-Years	1·99 (1·32–2·87)
Nasopharynx cancer	All Ages	Both	32.1 Pack-Years	2·30 (1·45–3·32)
Nasopharynx cancer	All Ages	Both	42.9 Pack-Years	2·66 (1·65–4·01)
Nasopharynx cancer	All Ages	Both	53.6 Pack-Years	3·38 (2·01–5·04)
Nasopharynx cancer	All Ages	Both	64.3 Pack-Years	4·47 (2·64–6·83)
Other pharynx cancer	All Ages	Both	0 Pack-Years	1·00 (1·00–1·00)
Other pharynx cancer	All Ages	Both	17.7 Pack-Years	4·79 (3·27–6·50)
Other pharynx cancer	All Ages	Both	35.4 Pack-Years	5·26 (3·54–7·38)
Other pharynx cancer	All Ages	Both	53.1 Pack-Years	6·59 (4·41–8·89)
Other pharynx cancer	All Ages	Both	70.8 Pack-Years	7·21 (3·97–11·6)
Other pharynx cancer	All Ages	Both	88.5 Pack-Years	11·1 (5·54–18·6)
Pancreatic cancer	All Ages	Male	0 Pack-Years	1·00 (1·00–1·00)
Pancreatic cancer	All Ages	Male	15.8 Pack-Years	1·65 (1·33–1·98)
Pancreatic cancer	All Ages	Male	31.5 Pack-Years	1·80 (1·45–2·18)
Pancreatic cancer	All Ages	Male	47.3 Pack-Years	2·17 (1·75–2·61)
Pancreatic cancer	All Ages	Male	63 Pack-Years	2·43 (1·79–3·21)
Pancreatic cancer	All Ages	Female	0 Pack-Years	1·00 (1·00–1·00)
Pancreatic cancer	All Ages	Female	15.8 Pack-Years	2·36 (1·90–2·85)
Pancreatic cancer	All Ages	Female	31.5 Pack-Years	2·75 (2·10–3·46)
Pancreatic cancer	All Ages	Female	47.3 Pack-Years	3·28 (2·36–4·34)
Pancreatic cancer	All Ages	Female	63 Pack-Years	3·84 (2·06–6·06)
Kidney cancer	All Ages	Both	0 Pack-Years	1·00 (1·00–1·00)

Kidney cancer	All Ages	Both	12.3 Pack-Years	1·37 (1·05–1·75)
Kidney cancer	All Ages	Both	24.6 Pack-Years	1·73 (1·34–2·16)
Kidney cancer	All Ages	Both	36.9 Pack-Years	1·76 (1·30–2·31)
Kidney cancer	All Ages	Both	49.2 Pack-Years	1·89 (1·43–2·44)
Kidney cancer	All Ages	Both	61.5 Pack-Years	2·01 (1·25–3·03)
Bladder cancer	All Ages	Both	0 Pack-Years	1·00 (1·00–1·00)
Bladder cancer	All Ages	Both	15 Pack-Years	2·80 (1·69–4·16)
Bladder cancer	All Ages	Both	30 Pack-Years	3·33 (2·09–5·00)
Bladder cancer	All Ages	Both	45 Pack-Years	4·24 (2·45–6·53)
Bladder cancer	All Ages	Both	60 Pack-Years	4·55 (2·54–7·64)
Leukemia	All Ages	Both	0 Pack-Years	1·00 (1·00–1·00)
Leukemia	All Ages	Both	12.4 Pack-Years	2·01 (1·00–3·61)
Leukemia	All Ages	Both	24.8 Pack-Years	2·25 (1·07–3·96)
Leukemia	All Ages	Both	37.1 Pack-Years	2·64 (1·48–4·39)
Ischemic heart disease	30 to 34	Male	0 Cigarette-Equivalents	1·00 (1·00–1·00)
Ischemic heart disease	30 to 34	Male	11.2 Cigarette-Equivalents	2·97 (2·57–3·37)
Ischemic heart disease	30 to 34	Male	22.5 Cigarette-Equivalents	3·29 (2·80–3·85)
Ischemic heart disease	30 to 34	Male	33.8 Cigarette-Equivalents	4·28 (3·41–5·10)
Ischemic heart disease	30 to 34	Male	45 Cigarette-Equivalents	5·45 (3·72–7·54)
Ischemic heart disease	30 to 34	Male	56.2 Cigarette-Equivalents	5·57 (3·77–7·90)
Ischemic heart disease	30 to 34	Female	0 Cigarette-Equivalents	1·00 (1·00–1·00)
Ischemic heart disease	30 to 34	Female	11.2 Cigarette-Equivalents	3·78 (2·96–4·81)
Ischemic heart disease	30 to 34	Female	22.5 Cigarette-Equivalents	6·68 (5·11–8·41)
Ischemic heart disease	30 to 34	Female	33.8 Cigarette-Equivalents	9·74 (7·03–12·9)
Ischemic heart disease	30 to 34	Female	45 Cigarette-Equivalents	11·8 (7·22–17·6)
Ischemic heart disease	30 to 34	Female	56.2 Cigarette-Equivalents	13·6 (7·46–22·8)
Ischemic heart disease	35 to 39	Male	0 Cigarette-Equivalents	1·00 (1·00–1·00)

Ischemic heart disease	35 to 39	Male	11.2 Cigarette-Equivalents	2·97 (2·57–3·37)
Ischemic heart disease	35 to 39	Male	22.5 Cigarette-Equivalents	3·29 (2·80–3·85)
Ischemic heart disease	35 to 39	Male	33.8 Cigarette-Equivalents	4·28 (3·41–5·10)
Ischemic heart disease	35 to 39	Male	45 Cigarette-Equivalents	5·45 (3·72–7·54)
Ischemic heart disease	35 to 39	Male	56.2 Cigarette-Equivalents	5·57 (3·77–7·90)
Ischemic heart disease	35 to 39	Female	0 Cigarette-Equivalents	1·00 (1·00–1·00)
Ischemic heart disease	35 to 39	Female	11.2 Cigarette-Equivalents	3·78 (2·96–4·81)
Ischemic heart disease	35 to 39	Female	22.5 Cigarette-Equivalents	6·68 (5·11–8·41)
Ischemic heart disease	35 to 39	Female	33.8 Cigarette-Equivalents	9·74 (7·03–12·9)
Ischemic heart disease	35 to 39	Female	45 Cigarette-Equivalents	11·8 (7·22–17·6)
Ischemic heart disease	35 to 39	Female	56.2 Cigarette-Equivalents	13·6 (7·46–22·8)
Ischemic heart disease	40 to 44	Male	0 Cigarette-Equivalents	1·00 (1·00–1·00)
Ischemic heart disease	40 to 44	Male	11.2 Cigarette-Equivalents	2·97 (2·57–3·37)
Ischemic heart disease	40 to 44	Male	22.5 Cigarette-Equivalents	3·29 (2·80–3·85)
Ischemic heart disease	40 to 44	Male	33.8 Cigarette-Equivalents	4·28 (3·41–5·10)
Ischemic heart disease	40 to 44	Male	45 Cigarette-Equivalents	5·45 (3·72–7·54)
Ischemic heart disease	40 to 44	Male	56.2 Cigarette-Equivalents	5·57 (3·77–7·90)
Ischemic heart disease	40 to 44	Female	0 Cigarette-Equivalents	1·00 (1·00–1·00)
Ischemic heart disease	40 to 44	Female	11.2 Cigarette-Equivalents	3·78 (2·96–4·81)
Ischemic heart disease	40 to 44	Female	22.5 Cigarette-Equivalents	6·68 (5·11–8·41)
Ischemic heart disease	40 to 44	Female	33.8 Cigarette-Equivalents	9·74 (7·03–12·9)
Ischemic heart disease	40 to 44	Female	45 Cigarette-Equivalents	11·8 (7·22–17·6)
Ischemic heart disease	40 to 44	Female	56.2 Cigarette-Equivalents	13·6 (7·46–22·8)
Ischemic heart disease	45 to 49	Male	0 Cigarette-Equivalents	1·00 (1·00–1·00)
Ischemic heart disease	45 to 49	Male	11.2 Cigarette-Equivalents	2·53 (2·23–2·81)
Ischemic heart disease	45 to 49	Male	22.5 Cigarette-Equivalents	2·67 (2·33–3·06)
Ischemic heart disease	45 to 49	Male	33.8 Cigarette-Equivalents	3·39 (2·79–4·01)

Ischemic heart disease	45 to 49	Male	45 Cigarette-Equivalents	4·14 (2·95–5·55)
Ischemic heart disease	45 to 49	Male	56.2 Cigarette-Equivalents	4·18 (2·88–5·61)
Ischemic heart disease	45 to 49	Female	0 Cigarette-Equivalents	1·00 (1·00–1·00)
Ischemic heart disease	45 to 49	Female	11.2 Cigarette-Equivalents	3·18 (2·59–3·82)
Ischemic heart disease	45 to 49	Female	22.5 Cigarette-Equivalents	4·92 (3·92–5·93)
Ischemic heart disease	45 to 49	Female	33.8 Cigarette-Equivalents	6·80 (5·18–8·76)
Ischemic heart disease	45 to 49	Female	45 Cigarette-Equivalents	7·73 (4·89–11·4)
Ischemic heart disease	45 to 49	Female	56.2 Cigarette-Equivalents	8·81 (4·96–14·0)
Ischemic heart disease	50 to 54	Male	0 Cigarette-Equivalents	1·00 (1·00–1·00)
Ischemic heart disease	50 to 54	Male	11.2 Cigarette-Equivalents	2·53 (2·23–2·81)
Ischemic heart disease	50 to 54	Male	22.5 Cigarette-Equivalents	2·67 (2·33–3·06)
Ischemic heart disease	50 to 54	Male	33.8 Cigarette-Equivalents	3·39 (2·79–4·01)
Ischemic heart disease	50 to 54	Male	45 Cigarette-Equivalents	4·14 (2·95–5·55)
Ischemic heart disease	50 to 54	Male	56.2 Cigarette-Equivalents	4·18 (2·88–5·61)
Ischemic heart disease	50 to 54	Female	0 Cigarette-Equivalents	1·00 (1·00–1·00)
Ischemic heart disease	50 to 54	Female	11.2 Cigarette-Equivalents	3·18 (2·59–3·82)
Ischemic heart disease	50 to 54	Female	22.5 Cigarette-Equivalents	4·92 (3·92–5·93)
Ischemic heart disease	50 to 54	Female	33.8 Cigarette-Equivalents	6·80 (5·18–8·76)
Ischemic heart disease	50 to 54	Female	45 Cigarette-Equivalents	7·73 (4·89–11·4)
Ischemic heart disease	50 to 54	Female	56.2 Cigarette-Equivalents	8·81 (4·96–14·0)
Ischemic heart disease	55 to 59	Male	0 Cigarette-Equivalents	1·00 (1·00–1·00)
Ischemic heart disease	55 to 59	Male	11.2 Cigarette-Equivalents	2·13 (1·91–2·35)
Ischemic heart disease	55 to 59	Male	22.5 Cigarette-Equivalents	2·19 (1·94–2·43)
Ischemic heart disease	55 to 59	Male	33.8 Cigarette-Equivalents	2·63 (2·26–3·04)
Ischemic heart disease	55 to 59	Male	45 Cigarette-Equivalents	3·08 (2·30–3·93)
Ischemic heart disease	55 to 59	Male	56.2 Cigarette-Equivalents	3·02 (2·22–3·96)
Ischemic heart disease	55 to 59	Female	0 Cigarette-Equivalents	1·00 (1·00–1·00)

Ischemic heart disease	55 to 59	Female	11.2 Cigarette-Equivalents	2·61 (2·20–3·03)
Ischemic heart disease	55 to 59	Female	22.5 Cigarette-Equivalents	3·57 (3·01–4·16)
Ischemic heart disease	55 to 59	Female	33.8 Cigarette-Equivalents	4·68 (3·71–5·73)
Ischemic heart disease	55 to 59	Female	45 Cigarette-Equivalents	5·10 (3·52–7·02)
Ischemic heart disease	55 to 59	Female	56.2 Cigarette-Equivalents	5·61 (3·44–8·34)
Ischemic heart disease	60 to 64	Male	0 Cigarette-Equivalents	1·00 (1·00–1·00)
Ischemic heart disease	60 to 64	Male	11.2 Cigarette-Equivalents	2·13 (1·91–2·35)
Ischemic heart disease	60 to 64	Male	22.5 Cigarette-Equivalents	2·19 (1·94–2·43)
Ischemic heart disease	60 to 64	Male	33.8 Cigarette-Equivalents	2·63 (2·26–3·04)
Ischemic heart disease	60 to 64	Male	45 Cigarette-Equivalents	3·08 (2·30–3·93)
Ischemic heart disease	60 to 64	Male	56.2 Cigarette-Equivalents	3·02 (2·22–3·96)
Ischemic heart disease	60 to 64	Female	0 Cigarette-Equivalents	1·00 (1·00–1·00)
Ischemic heart disease	60 to 64	Female	11.2 Cigarette-Equivalents	2·61 (2·20–3·03)
Ischemic heart disease	60 to 64	Female	22.5 Cigarette-Equivalents	3·57 (3·01–4·16)
Ischemic heart disease	60 to 64	Female	33.8 Cigarette-Equivalents	4·68 (3·71–5·73)
Ischemic heart disease	60 to 64	Female	45 Cigarette-Equivalents	5·10 (3·52–7·02)
Ischemic heart disease	60 to 64	Female	56.2 Cigarette-Equivalents	5·61 (3·44–8·34)
Ischemic heart disease	65 to 69	Male	0 Cigarette-Equivalents	1·00 (1·00–1·00)
Ischemic heart disease	65 to 69	Male	11.2 Cigarette-Equivalents	1·77 (1·64–1·91)
Ischemic heart disease	65 to 69	Male	22.5 Cigarette-Equivalents	1·78 (1·63–1·96)
Ischemic heart disease	65 to 69	Male	33.8 Cigarette-Equivalents	2·05 (1·82–2·30)
Ischemic heart disease	65 to 69	Male	45 Cigarette-Equivalents	2·33 (1·75–2·93)
Ischemic heart disease	65 to 69	Male	56.2 Cigarette-Equivalents	2·23 (1·65–2·85)
Ischemic heart disease	65 to 69	Female	0 Cigarette-Equivalents	1·00 (1·00–1·00)
Ischemic heart disease	65 to 69	Female	11.2 Cigarette-Equivalents	2·13 (1·91–2·37)
Ischemic heart disease	65 to 69	Female	22.5 Cigarette-Equivalents	2·57 (2·26–2·88)
Ischemic heart disease	65 to 69	Female	33.8 Cigarette-Equivalents	3·25 (2·74–3·79)

Ischemic heart disease	65 to 69	Female	45 Cigarette-Equivalents	3·41 (2·46–4·47)
Ischemic heart disease	65 to 69	Female	56.2 Cigarette-Equivalents	3·69 (2·60–5·04)
Ischemic heart disease	70 to 74	Male	0 Cigarette-Equivalents	1·00 (1·00–1·00)
Ischemic heart disease	70 to 74	Male	11.2 Cigarette-Equivalents	1·77 (1·64–1·91)
Ischemic heart disease	70 to 74	Male	22.5 Cigarette-Equivalents	1·78 (1·63–1·96)
Ischemic heart disease	70 to 74	Male	33.8 Cigarette-Equivalents	2·05 (1·82–2·30)
Ischemic heart disease	70 to 74	Male	45 Cigarette-Equivalents	2·33 (1·75–2·93)
Ischemic heart disease	70 to 74	Male	56.2 Cigarette-Equivalents	2·23 (1·65–2·85)
Ischemic heart disease	70 to 74	Female	0 Cigarette-Equivalents	1·00 (1·00–1·00)
Ischemic heart disease	70 to 74	Female	11.2 Cigarette-Equivalents	2·13 (1·91–2·37)
Ischemic heart disease	70 to 74	Female	22.5 Cigarette-Equivalents	2·57 (2·26–2·88)
Ischemic heart disease	70 to 74	Female	33.8 Cigarette-Equivalents	3·25 (2·74–3·79)
Ischemic heart disease	70 to 74	Female	45 Cigarette-Equivalents	3·41 (2·46–4·47)
Ischemic heart disease	70 to 74	Female	56.2 Cigarette-Equivalents	3·69 (2·60–5·04)
Ischemic heart disease	75 to 79	Male	0 Cigarette-Equivalents	1·00 (1·00–1·00)
Ischemic heart disease	75 to 79	Male	11.2 Cigarette-Equivalents	1·46 (1·37–1·56)
Ischemic heart disease	75 to 79	Male	22.5 Cigarette-Equivalents	1·46 (1·37–1·56)
Ischemic heart disease	75 to 79	Male	33.8 Cigarette-Equivalents	1·60 (1·46–1·73)
Ischemic heart disease	75 to 79	Male	45 Cigarette-Equivalents	1·74 (1·39–2·10)
Ischemic heart disease	75 to 79	Male	56.2 Cigarette-Equivalents	1·65 (1·29–2·02)
Ischemic heart disease	75 to 79	Female	0 Cigarette-Equivalents	1·00 (1·00–1·00)
Ischemic heart disease	75 to 79	Female	11.2 Cigarette-Equivalents	1·68 (1·56–1·79)
Ischemic heart disease	75 to 79	Female	22.5 Cigarette-Equivalents	1·85 (1·71–2·00)
Ischemic heart disease	75 to 79	Female	33.8 Cigarette-Equivalents	2·18 (1·94–2·43)
Ischemic heart disease	75 to 79	Female	45 Cigarette-Equivalents	2·24 (1·75–2·79)
Ischemic heart disease	75 to 79	Female	56.2 Cigarette-Equivalents	2·41 (1·79–3·04)
Ischemic heart disease	80 to 84	Male	0 Cigarette-Equivalents	1·00 (1·00–1·00)

Ischemic heart disease	80 to 84	Male	11.2 Cigarette-Equivalents	1·46 (1·37–1·56)
Ischemic heart disease	80 to 84	Male	22.5 Cigarette-Equivalents	1·46 (1·37–1·56)
Ischemic heart disease	80 to 84	Male	33.8 Cigarette-Equivalents	1·60 (1·46–1·73)
Ischemic heart disease	80 to 84	Male	45 Cigarette-Equivalents	1·74 (1·39–2·10)
Ischemic heart disease	80 to 84	Male	56.2 Cigarette-Equivalents	1·65 (1·29–2·02)
Ischemic heart disease	80 to 84	Female	0 Cigarette-Equivalents	1·00 (1·00–1·00)
Ischemic heart disease	80 to 84	Female	11.2 Cigarette-Equivalents	1·68 (1·56–1·79)
Ischemic heart disease	80 to 84	Female	22.5 Cigarette-Equivalents	1·85 (1·71–2·00)
Ischemic heart disease	80 to 84	Female	33.8 Cigarette-Equivalents	2·18 (1·94–2·43)
Ischemic heart disease	80 to 84	Female	45 Cigarette-Equivalents	2·24 (1·75–2·79)
Ischemic heart disease	80 to 84	Female	56.2 Cigarette-Equivalents	2·41 (1·79–3·04)
Ischemic heart disease	85 to 89	Male	0 Cigarette-Equivalents	1·00 (1·00–1·00)
Ischemic heart disease	85 to 89	Male	11.2 Cigarette-Equivalents	1·19 (1·13–1·25)
Ischemic heart disease	85 to 89	Male	22.5 Cigarette-Equivalents	1·19 (1·15–1·23)
Ischemic heart disease	85 to 89	Male	33.8 Cigarette-Equivalents	1·24 (1·17–1·31)
Ischemic heart disease	85 to 89	Male	45 Cigarette-Equivalents	1·30 (1·13–1·48)
Ischemic heart disease	85 to 89	Male	56.2 Cigarette-Equivalents	1·23 (1·02–1·44)
Ischemic heart disease	85 to 89	Female	0 Cigarette-Equivalents	1·00 (1·00–1·00)
Ischemic heart disease	85 to 89	Female	11.2 Cigarette-Equivalents	1·28 (1·22–1·33)
Ischemic heart disease	85 to 89	Female	22.5 Cigarette-Equivalents	1·33 (1·28–1·39)
Ischemic heart disease	85 to 89	Female	33.8 Cigarette-Equivalents	1·45 (1·37–1·54)
Ischemic heart disease	85 to 89	Female	45 Cigarette-Equivalents	1·44 (1·24–1·65)
Ischemic heart disease	85 to 89	Female	56.2 Cigarette-Equivalents	1·58 (1·33–1·85)
Ischemic heart disease	90 to 94	Male	0 Cigarette-Equivalents	1·00 (1·00–1·00)
Ischemic heart disease	90 to 94	Male	11.2 Cigarette-Equivalents	1·19 (1·13–1·25)
Ischemic heart disease	90 to 94	Male	22.5 Cigarette-Equivalents	1·19 (1·15–1·23)
Ischemic heart disease	90 to 94	Male	33.8 Cigarette-Equivalents	1·24 (1·17–1·31)

Ischemic heart disease	90 to 94	Male	45 Cigarette-Equivalents	1·30 (1·13–1·48)
Ischemic heart disease	90 to 94	Male	56.2 Cigarette-Equivalents	1·23 (1·02–1·44)
Ischemic heart disease	90 to 94	Female	0 Cigarette-Equivalents	1·00 (1·00–1·00)
Ischemic heart disease	90 to 94	Female	11.2 Cigarette-Equivalents	1·28 (1·22–1·33)
Ischemic heart disease	90 to 94	Female	22.5 Cigarette-Equivalents	1·33 (1·28–1·39)
Ischemic heart disease	90 to 94	Female	33.8 Cigarette-Equivalents	1·45 (1·37–1·54)
Ischemic heart disease	90 to 94	Female	45 Cigarette-Equivalents	1·44 (1·24–1·65)
Ischemic heart disease	90 to 94	Female	56.2 Cigarette-Equivalents	1·58 (1·33–1·85)
Ischemic heart disease	95 plus	Male	0 Cigarette-Equivalents	1·00 (1·00–1·00)
Ischemic heart disease	95 plus	Male	11.2 Cigarette-Equivalents	1·00 (1·00–1·01)
Ischemic heart disease	95 plus	Male	22.5 Cigarette-Equivalents	1·00 (1·00–1·01)
Ischemic heart disease	95 plus	Male	33.8 Cigarette-Equivalents	1·00 (1·00–1·01)
Ischemic heart disease	95 plus	Male	45 Cigarette-Equivalents	1·01 (1·00–1·04)
Ischemic heart disease	95 plus	Male	56.2 Cigarette-Equivalents	1·01 (1·00–1·05)
Ischemic heart disease	95 plus	Female	0 Cigarette-Equivalents	1·00 (1·00–1·00)
Ischemic heart disease	95 plus	Female	11.2 Cigarette-Equivalents	1·00 (1·00–1·01)
Ischemic heart disease	95 plus	Female	22.5 Cigarette-Equivalents	1·00 (1·00–1·01)
Ischemic heart disease	95 plus	Female	33.8 Cigarette-Equivalents	1·00 (1·00–1·01)
Ischemic heart disease	95 plus	Female	45 Cigarette-Equivalents	1·01 (1·00–1·04)
Ischemic heart disease	95 plus	Female	56.2 Cigarette-Equivalents	1·02 (1·00–1·08)
Stroke	30 to 34	Male	0 Cigarette-Equivalents	1·00 (1·00–1·00)
Stroke	30 to 34	Male	10 Cigarette-Equivalents	2·36 (1·98–2·74)
Stroke	30 to 34	Male	20 Cigarette-Equivalents	2·32 (1·92–2·75)
Stroke	30 to 34	Male	30 Cigarette-Equivalents	3·18 (2·65–3·77)
Stroke	30 to 34	Male	40 Cigarette-Equivalents	3·43 (2·45–4·61)
Stroke	30 to 34	Male	50 Cigarette-Equivalents	4·46 (2·94–6·30)
Stroke	30 to 34	Male	60 Cigarette-Equivalents	5·32 (2·79–8·67)

Stroke	30 to 34	Female	0 Cigarette-Equivalents	1·00 (1·00–1·00)
Stroke	30 to 34	Female	10 Cigarette-Equivalents	2·33 (1·69–3·17)
Stroke	30 to 34	Female	20 Cigarette-Equivalents	3·74 (2·69–4·98)
Stroke	30 to 34	Female	30 Cigarette-Equivalents	5·49 (3·81–7·70)
Stroke	30 to 34	Female	40 Cigarette-Equivalents	7·13 (4·10–11·5)
Stroke	30 to 34	Female	50 Cigarette-Equivalents	9·85 (5·01–17·9)
Stroke	30 to 34	Female	60 Cigarette-Equivalents	11·7 (4·84–23·9)
Stroke	35 to 39	Male	0 Cigarette-Equivalents	1·00 (1·00–1·00)
Stroke	35 to 39	Male	10 Cigarette-Equivalents	2·36 (1·98–2·74)
Stroke	35 to 39	Male	20 Cigarette-Equivalents	2·32 (1·92–2·75)
Stroke	35 to 39	Male	30 Cigarette-Equivalents	3·18 (2·65–3·77)
Stroke	35 to 39	Male	40 Cigarette-Equivalents	3·43 (2·45–4·61)
Stroke	35 to 39	Male	50 Cigarette-Equivalents	4·46 (2·94–6·30)
Stroke	35 to 39	Male	60 Cigarette-Equivalents	5·32 (2·79–8·67)
Stroke	35 to 39	Female	0 Cigarette-Equivalents	1·00 (1·00–1·00)
Stroke	35 to 39	Female	10 Cigarette-Equivalents	2·33 (1·69–3·17)
Stroke	35 to 39	Female	20 Cigarette-Equivalents	3·74 (2·69–4·98)
Stroke	35 to 39	Female	30 Cigarette-Equivalents	5·49 (3·81–7·70)
Stroke	35 to 39	Female	40 Cigarette-Equivalents	7·13 (4·10–11·5)
Stroke	35 to 39	Female	50 Cigarette-Equivalents	9·85 (5·01–17·9)
Stroke	35 to 39	Female	60 Cigarette-Equivalents	11·7 (4·84–23·9)
Stroke	40 to 44	Male	0 Cigarette-Equivalents	1·00 (1·00–1·00)
Stroke	40 to 44	Male	10 Cigarette-Equivalents	2·36 (1·98–2·74)
Stroke	40 to 44	Male	20 Cigarette-Equivalents	2·32 (1·92–2·75)
Stroke	40 to 44	Male	30 Cigarette-Equivalents	3·18 (2·65–3·77)
Stroke	40 to 44	Male	40 Cigarette-Equivalents	3·43 (2·45–4·61)
Stroke	40 to 44	Male	50 Cigarette-Equivalents	4·46 (2·94–6·30)

Stroke	40 to 44	Male	60 Cigarette-Equivalents	5·32 (2·79–8·67)
Stroke	40 to 44	Female	0 Cigarette-Equivalents	1·00 (1·00–1·00)
Stroke	40 to 44	Female	10 Cigarette-Equivalents	2·33 (1·69–3·17)
Stroke	40 to 44	Female	20 Cigarette-Equivalents	3·74 (2·69–4·98)
Stroke	40 to 44	Female	30 Cigarette-Equivalents	5·49 (3·81–7·70)
Stroke	40 to 44	Female	40 Cigarette-Equivalents	7·13 (4·10–11·5)
Stroke	40 to 44	Female	50 Cigarette-Equivalents	9·85 (5·01–17·9)
Stroke	40 to 44	Female	60 Cigarette-Equivalents	11·7 (4·84–23·9)
Stroke	45 to 49	Male	0 Cigarette-Equivalents	1·00 (1·00–1·00)
Stroke	45 to 49	Male	10 Cigarette-Equivalents	2·11 (1·84–2·40)
Stroke	45 to 49	Male	20 Cigarette-Equivalents	1·97 (1·70–2·28)
Stroke	45 to 49	Male	30 Cigarette-Equivalents	2·67 (2·26–3·07)
Stroke	45 to 49	Male	40 Cigarette-Equivalents	2·73 (2·01–3·60)
Stroke	45 to 49	Male	50 Cigarette-Equivalents	3·49 (2·40–4·74)
Stroke	45 to 49	Male	60 Cigarette-Equivalents	4·16 (2·30–6·58)
Stroke	45 to 49	Female	0 Cigarette-Equivalents	1·00 (1·00–1·00)
Stroke	45 to 49	Female	10 Cigarette-Equivalents	2·10 (1·60–2·72)
Stroke	45 to 49	Female	20 Cigarette-Equivalents	3·04 (2·30–3·94)
Stroke	45 to 49	Female	30 Cigarette-Equivalents	4·21 (2·99–5·64)
Stroke	45 to 49	Female	40 Cigarette-Equivalents	5·10 (3·04–7·72)
Stroke	45 to 49	Female	50 Cigarette-Equivalents	6·92 (3·96–11·4)
Stroke	45 to 49	Female	60 Cigarette-Equivalents	8·28 (3·61–16·4)
Stroke	50 to 54	Male	0 Cigarette-Equivalents	1·00 (1·00–1·00)
Stroke	50 to 54	Male	10 Cigarette-Equivalents	2·11 (1·84–2·40)
Stroke	50 to 54	Male	20 Cigarette-Equivalents	1·97 (1·70–2·28)
Stroke	50 to 54	Male	30 Cigarette-Equivalents	2·67 (2·26–3·07)
Stroke	50 to 54	Male	40 Cigarette-Equivalents	2·73 (2·01–3·60)

Stroke	50 to 54	Male	50 Cigarette-Equivalents	3·49 (2·40–4·74)
Stroke	50 to 54	Male	60 Cigarette-Equivalents	4·16 (2·30–6·58)
Stroke	50 to 54	Female	0 Cigarette-Equivalents	1·00 (1·00–1·00)
Stroke	50 to 54	Female	10 Cigarette-Equivalents	2·10 (1·60–2·72)
Stroke	50 to 54	Female	20 Cigarette-Equivalents	3·04 (2·30–3·94)
Stroke	50 to 54	Female	30 Cigarette-Equivalents	4·21 (2·99–5·64)
Stroke	50 to 54	Female	40 Cigarette-Equivalents	5·10 (3·04–7·72)
Stroke	50 to 54	Female	50 Cigarette-Equivalents	6·92 (3·96–11·4)
Stroke	50 to 54	Female	60 Cigarette-Equivalents	8·28 (3·61–16·4)
Stroke	55 to 59	Male	0 Cigarette-Equivalents	1·00 (1·00–1·00)
Stroke	55 to 59	Male	10 Cigarette-Equivalents	1·84 (1·64–2·06)
Stroke	55 to 59	Male	20 Cigarette-Equivalents	1·66 (1·45–1·88)
Stroke	55 to 59	Male	30 Cigarette-Equivalents	2·22 (1·94–2·52)
Stroke	55 to 59	Male	40 Cigarette-Equivalents	2·12 (1·62–2·74)
Stroke	55 to 59	Male	50 Cigarette-Equivalents	2·66 (1·92–3·56)
Stroke	55 to 59	Male	60 Cigarette-Equivalents	3·17 (1·80–4·85)
Stroke	55 to 59	Female	0 Cigarette-Equivalents	1·00 (1·00–1·00)
Stroke	55 to 59	Female	10 Cigarette-Equivalents	1·92 (1·51–2·33)
Stroke	55 to 59	Female	20 Cigarette-Equivalents	2·44 (1·88–3·06)
Stroke	55 to 59	Female	30 Cigarette-Equivalents	3·14 (2·33–4·02)
Stroke	55 to 59	Female	40 Cigarette-Equivalents	3·64 (2·30–5·22)
Stroke	55 to 59	Female	50 Cigarette-Equivalents	5·00 (3·06–7·75)
Stroke	55 to 59	Female	60 Cigarette-Equivalents	5·94 (3·01–11·1)
Stroke	60 to 64	Male	0 Cigarette-Equivalents	1·00 (1·00–1·00)
Stroke	60 to 64	Male	10 Cigarette-Equivalents	1·84 (1·64–2·06)
Stroke	60 to 64	Male	20 Cigarette-Equivalents	1·66 (1·45–1·88)
Stroke	60 to 64	Male	30 Cigarette-Equivalents	2·22 (1·94–2·52)

Stroke	60 to 64	Male	40 Cigarette-Equivalents	2·12 (1·62–2·74)
Stroke	60 to 64	Male	50 Cigarette-Equivalents	2·66 (1·92–3·56)
Stroke	60 to 64	Male	60 Cigarette-Equivalents	3·17 (1·80–4·85)
Stroke	60 to 64	Female	0 Cigarette-Equivalents	1·00 (1·00–1·00)
Stroke	60 to 64	Female	10 Cigarette-Equivalents	1·92 (1·51–2·33)
Stroke	60 to 64	Female	20 Cigarette-Equivalents	2·44 (1·88–3·06)
Stroke	60 to 64	Female	30 Cigarette-Equivalents	3·14 (2·33–4·02)
Stroke	60 to 64	Female	40 Cigarette-Equivalents	3·64 (2·30–5·22)
Stroke	60 to 64	Female	50 Cigarette-Equivalents	5·00 (3·06–7·75)
Stroke	60 to 64	Female	60 Cigarette-Equivalents	5·94 (3·01–11·1)
Stroke	65 to 69	Male	0 Cigarette-Equivalents	1·00 (1·00–1·00)
Stroke	65 to 69	Male	10 Cigarette-Equivalents	1·61 (1·46–1·76)
Stroke	65 to 69	Male	20 Cigarette-Equivalents	1·42 (1·28–1·56)
Stroke	65 to 69	Male	30 Cigarette-Equivalents	1·85 (1·66–2·04)
Stroke	65 to 69	Male	40 Cigarette-Equivalents	1·66 (1·31–2·06)
Stroke	65 to 69	Male	50 Cigarette-Equivalents	2·05 (1·53–2·61)
Stroke	65 to 69	Male	60 Cigarette-Equivalents	2·40 (1·46–3·51)
Stroke	65 to 69	Female	0 Cigarette-Equivalents	1·00 (1·00–1·00)
Stroke	65 to 69	Female	10 Cigarette-Equivalents	1·72 (1·44–2·01)
Stroke	65 to 69	Female	20 Cigarette-Equivalents	1·94 (1·60–2·28)
Stroke	65 to 69	Female	30 Cigarette-Equivalents	2·30 (1·87–2·82)
Stroke	65 to 69	Female	40 Cigarette-Equivalents	2·59 (1·81–3·59)
Stroke	65 to 69	Female	50 Cigarette-Equivalents	3·57 (2·40–4·97)
Stroke	65 to 69	Female	60 Cigarette-Equivalents	4·29 (2·32–7·08)
Stroke	70 to 74	Male	0 Cigarette-Equivalents	1·00 (1·00–1·00)
Stroke	70 to 74	Male	10 Cigarette-Equivalents	1·61 (1·46–1·76)
Stroke	70 to 74	Male	20 Cigarette-Equivalents	1·42 (1·28–1·56)

Stroke	70 to 74	Male	30 Cigarette-Equivalents	1·85 (1·66–2·04)
Stroke	70 to 74	Male	40 Cigarette-Equivalents	1·66 (1·31–2·06)
Stroke	70 to 74	Male	50 Cigarette-Equivalents	2·05 (1·53–2·61)
Stroke	70 to 74	Male	60 Cigarette-Equivalents	2·40 (1·46–3·51)
Stroke	70 to 74	Female	0 Cigarette-Equivalents	1·00 (1·00–1·00)
Stroke	70 to 74	Female	10 Cigarette-Equivalents	1·72 (1·44–2·01)
Stroke	70 to 74	Female	20 Cigarette-Equivalents	1·94 (1·60–2·28)
Stroke	70 to 74	Female	30 Cigarette-Equivalents	2·30 (1·87–2·82)
Stroke	70 to 74	Female	40 Cigarette-Equivalents	2·59 (1·81–3·59)
Stroke	70 to 74	Female	50 Cigarette-Equivalents	3·57 (2·40–4·97)
Stroke	70 to 74	Female	60 Cigarette-Equivalents	4·29 (2·32–7·08)
Stroke	75 to 79	Male	0 Cigarette-Equivalents	1·00 (1·00–1·00)
Stroke	75 to 79	Male	10 Cigarette-Equivalents	1·39 (1·29–1·48)
Stroke	75 to 79	Male	20 Cigarette-Equivalents	1·22 (1·14–1·31)
Stroke	75 to 79	Male	30 Cigarette-Equivalents	1·52 (1·40–1·64)
Stroke	75 to 79	Male	40 Cigarette-Equivalents	1·34 (1·12–1·59)
Stroke	75 to 79	Male	50 Cigarette-Equivalents	1·58 (1·20–1·98)
Stroke	75 to 79	Male	60 Cigarette-Equivalents	1·84 (1·18–2·68)
Stroke	75 to 79	Female	0 Cigarette-Equivalents	1·00 (1·00–1·00)
Stroke	75 to 79	Female	10 Cigarette-Equivalents	1·48 (1·32–1·64)
Stroke	75 to 79	Female	20 Cigarette-Equivalents	1·53 (1·33–1·72)
Stroke	75 to 79	Female	30 Cigarette-Equivalents	1·72 (1·47–2·01)
Stroke	75 to 79	Female	40 Cigarette-Equivalents	1·78 (1·32–2·38)
Stroke	75 to 79	Female	50 Cigarette-Equivalents	2·35 (1·70–3·09)
Stroke	75 to 79	Female	60 Cigarette-Equivalents	2·78 (1·64–4·28)
Stroke	80 to 84	Male	0 Cigarette-Equivalents	1·00 (1·00–1·00)
Stroke	80 to 84	Male	10 Cigarette-Equivalents	1·39 (1·29–1·48)

Stroke	80 to 84	Male	20 Cigarette-Equivalents	1·22 (1·14–1·31)
Stroke	80 to 84	Male	30 Cigarette-Equivalents	1·52 (1·40–1·64)
Stroke	80 to 84	Male	40 Cigarette-Equivalents	1·34 (1·12–1·59)
Stroke	80 to 84	Male	50 Cigarette-Equivalents	1·58 (1·20–1·98)
Stroke	80 to 84	Male	60 Cigarette-Equivalents	1·84 (1·18–2·68)
Stroke	80 to 84	Female	0 Cigarette-Equivalents	1·00 (1·00–1·00)
Stroke	80 to 84	Female	10 Cigarette-Equivalents	1·48 (1·32–1·64)
Stroke	80 to 84	Female	20 Cigarette-Equivalents	1·53 (1·33–1·72)
Stroke	80 to 84	Female	30 Cigarette-Equivalents	1·72 (1·47–2·01)
Stroke	80 to 84	Female	40 Cigarette-Equivalents	1·78 (1·32–2·38)
Stroke	80 to 84	Female	50 Cigarette-Equivalents	2·35 (1·70–3·09)
Stroke	80 to 84	Female	60 Cigarette-Equivalents	2·78 (1·64–4·28)
Stroke	85 to 89	Male	0 Cigarette-Equivalents	1·00 (1·00–1·00)
Stroke	85 to 89	Male	10 Cigarette-Equivalents	1·15 (1·10–1·20)
Stroke	85 to 89	Male	20 Cigarette-Equivalents	1·08 (1·04–1·12)
Stroke	85 to 89	Male	30 Cigarette-Equivalents	1·21 (1·15–1·26)
Stroke	85 to 89	Male	40 Cigarette-Equivalents	1·12 (1·02–1·22)
Stroke	85 to 89	Male	50 Cigarette-Equivalents	1·21 (1·00–1·46)
Stroke	85 to 89	Male	60 Cigarette-Equivalents	1·39 (1·00–1·89)
Stroke	85 to 89	Female	0 Cigarette-Equivalents	1·00 (1·00–1·00)
Stroke	85 to 89	Female	10 Cigarette-Equivalents	1·20 (1·12–1·30)
Stroke	85 to 89	Female	20 Cigarette-Equivalents	1·19 (1·13–1·27)
Stroke	85 to 89	Female	30 Cigarette-Equivalents	1·29 (1·17–1·41)
Stroke	85 to 89	Female	40 Cigarette-Equivalents	1·21 (1·00–1·43)
Stroke	85 to 89	Female	50 Cigarette-Equivalents	1·45 (1·14–1·81)
Stroke	85 to 89	Female	60 Cigarette-Equivalents	1·69 (1·09–2·38)
Stroke	90 to 94	Male	0 Cigarette-Equivalents	1·00 (1·00–1·00)

Stroke	90 to 94	Male	10 Cigarette-Equivalents	1·15 (1·10–1·20)
Stroke	90 to 94	Male	20 Cigarette-Equivalents	1·08 (1·04–1·12)
Stroke	90 to 94	Male	30 Cigarette-Equivalents	1·21 (1·15–1·26)
Stroke	90 to 94	Male	40 Cigarette-Equivalents	1·12 (1·02–1·22)
Stroke	90 to 94	Male	50 Cigarette-Equivalents	1·21 (1·00–1·46)
Stroke	90 to 94	Male	60 Cigarette-Equivalents	1·39 (1·00–1·89)
Stroke	90 to 94	Female	0 Cigarette-Equivalents	1·00 (1·00–1·00)
Stroke	90 to 94	Female	10 Cigarette-Equivalents	1·20 (1·12–1·30)
Stroke	90 to 94	Female	20 Cigarette-Equivalents	1·19 (1·13–1·27)
Stroke	90 to 94	Female	30 Cigarette-Equivalents	1·29 (1·17–1·41)
Stroke	90 to 94	Female	40 Cigarette-Equivalents	1·21 (1·00–1·43)
Stroke	90 to 94	Female	50 Cigarette-Equivalents	1·45 (1·14–1·81)
Stroke	90 to 94	Female	60 Cigarette-Equivalents	1·69 (1·09–2·38)
Stroke	95 plus	Male	0 Cigarette-Equivalents	1·00 (1·00–1·00)
Stroke	95 plus	Male	10 Cigarette-Equivalents	1·01 (1·00–1·04)
Stroke	95 plus	Male	20 Cigarette-Equivalents	1·00 (1·00–1·02)
Stroke	95 plus	Male	30 Cigarette-Equivalents	1·01 (1·00–1·03)
Stroke	95 plus	Male	40 Cigarette-Equivalents	1·01 (1·00–1·04)
Stroke	95 plus	Male	50 Cigarette-Equivalents	1·04 (1·00–1·14)
Stroke	95 plus	Male	60 Cigarette-Equivalents	1·10 (1·00–1·37)
Stroke	95 plus	Female	0 Cigarette-Equivalents	1·00 (1·00–1·00)
Stroke	95 plus	Female	10 Cigarette-Equivalents	1·01 (1·00–1·03)
Stroke	95 plus	Female	20 Cigarette-Equivalents	1·00 (1·00–1·01)
Stroke	95 plus	Female	30 Cigarette-Equivalents	1·01 (1·00–1·02)
Stroke	95 plus	Female	40 Cigarette-Equivalents	1·01 (1·00–1·04)
Stroke	95 plus	Female	50 Cigarette-Equivalents	1·03 (1·00–1·11)
Stroke	95 plus	Female	60 Cigarette-Equivalents	1·04 (1·00–1·23)

Atrial fibrillation and flutter	30 to 34	Female	0 Cigarette-Equivalents	1·00 (1·00–1·00)
Atrial fibrillation and flutter	30 to 34	Female	6.3 Cigarette-Equivalents	1·53 (1·04–2·15)
Atrial fibrillation and flutter	30 to 34	Female	12.6 Cigarette-Equivalents	1·90 (1·24–2·79)
Atrial fibrillation and flutter	30 to 34	Female	18.9 Cigarette-Equivalents	2·22 (1·43–3·33)
Atrial fibrillation and flutter	30 to 34	Female	25.2 Cigarette-Equivalents	2·59 (1·54–4·08)
Atrial fibrillation and flutter	30 to 34	Male	0 Cigarette-Equivalents	1·00 (1·00–1·00)
Atrial fibrillation and flutter	30 to 34	Male	6.3 Cigarette-Equivalents	1·53 (1·04–2·15)
Atrial fibrillation and flutter	30 to 34	Male	12.6 Cigarette-Equivalents	1·90 (1·24–2·79)
Atrial fibrillation and flutter	30 to 34	Male	18.9 Cigarette-Equivalents	2·22 (1·43–3·33)
Atrial fibrillation and flutter	30 to 34	Male	25.2 Cigarette-Equivalents	2·59 (1·54–4·08)
Atrial fibrillation and flutter	35 to 39	Male	0 Cigarette-Equivalents	1·00 (1·00–1·00)
Atrial fibrillation and flutter	35 to 39	Male	6.3 Cigarette-Equivalents	1·53 (1·04–2·15)
Atrial fibrillation and flutter	35 to 39	Male	12.6 Cigarette-Equivalents	1·90 (1·24–2·79)
Atrial fibrillation and flutter	35 to 39	Male	18.9 Cigarette-Equivalents	2·22 (1·43–3·33)
Atrial fibrillation and flutter	35 to 39	Male	25.2 Cigarette-Equivalents	2·59 (1·54–4·08)
Atrial fibrillation and flutter	35 to 39	Female	0 Cigarette-Equivalents	1·00 (1·00–1·00)
Atrial fibrillation and flutter	35 to 39	Female	6.3 Cigarette-Equivalents	1·53 (1·04–2·15)
Atrial fibrillation and flutter	35 to 39	Female	12.6 Cigarette-Equivalents	1·90 (1·24–2·79)
Atrial fibrillation and flutter	35 to 39	Female	18.9 Cigarette-Equivalents	2·22 (1·43–3·33)
Atrial fibrillation and flutter	35 to 39	Female	25.2 Cigarette-Equivalents	2·59 (1·54–4·08)
Atrial fibrillation and flutter	40 to 44	Female	0 Cigarette-Equivalents	1·00 (1·00–1·00)
Atrial fibrillation and flutter	40 to 44	Female	6.3 Cigarette-Equivalents	1·53 (1·04–2·15)
Atrial fibrillation and flutter	40 to 44	Female	12.6 Cigarette-Equivalents	1·90 (1·24–2·79)
Atrial fibrillation and flutter	40 to 44	Female	18.9 Cigarette-Equivalents	2·22 (1·43–3·33)
Atrial fibrillation and flutter	40 to 44	Female	25.2 Cigarette-Equivalents	2·59 (1·54–4·08)
Atrial fibrillation and flutter	40 to 44	Male	0 Cigarette-Equivalents	1·00 (1·00–1·00)
Atrial fibrillation and flutter	40 to 44	Male	6.3 Cigarette-Equivalents	1·53 (1·04–2·15)

Atrial fibrillation and flutter	40 to 44	Male	12.6 Cigarette-Equivalents	1·90 (1·24–2·79)
Atrial fibrillation and flutter	40 to 44	Male	18.9 Cigarette-Equivalents	2·22 (1·43–3·33)
Atrial fibrillation and flutter	40 to 44	Male	25.2 Cigarette-Equivalents	2·59 (1·54–4·08)
Atrial fibrillation and flutter	45 to 49	Male	0 Cigarette-Equivalents	1·00 (1·00–1·00)
Atrial fibrillation and flutter	45 to 49	Male	6.3 Cigarette-Equivalents	1·51 (1·05–2·05)
Atrial fibrillation and flutter	45 to 49	Male	12.6 Cigarette-Equivalents	1·77 (1·21–2·55)
Atrial fibrillation and flutter	45 to 49	Male	18.9 Cigarette-Equivalents	2·00 (1·35–2·91)
Atrial fibrillation and flutter	45 to 49	Male	25.2 Cigarette-Equivalents	2·26 (1·45–3·40)
Atrial fibrillation and flutter	45 to 49	Female	0 Cigarette-Equivalents	1·00 (1·00–1·00)
Atrial fibrillation and flutter	45 to 49	Female	6.3 Cigarette-Equivalents	1·51 (1·05–2·05)
Atrial fibrillation and flutter	45 to 49	Female	12.6 Cigarette-Equivalents	1·77 (1·21–2·55)
Atrial fibrillation and flutter	45 to 49	Female	18.9 Cigarette-Equivalents	2·00 (1·35–2·91)
Atrial fibrillation and flutter	45 to 49	Female	25.2 Cigarette-Equivalents	2·26 (1·45–3·40)
Atrial fibrillation and flutter	50 to 54	Female	0 Cigarette-Equivalents	1·00 (1·00–1·00)
Atrial fibrillation and flutter	50 to 54	Female	6.3 Cigarette-Equivalents	1·51 (1·05–2·05)
Atrial fibrillation and flutter	50 to 54	Female	12.6 Cigarette-Equivalents	1·77 (1·21–2·55)
Atrial fibrillation and flutter	50 to 54	Female	18.9 Cigarette-Equivalents	2·00 (1·35–2·91)
Atrial fibrillation and flutter	50 to 54	Female	25.2 Cigarette-Equivalents	2·26 (1·45–3·40)
Atrial fibrillation and flutter	50 to 54	Male	0 Cigarette-Equivalents	1·00 (1·00–1·00)
Atrial fibrillation and flutter	50 to 54	Male	6.3 Cigarette-Equivalents	1·51 (1·05–2·05)
Atrial fibrillation and flutter	50 to 54	Male	12.6 Cigarette-Equivalents	1·77 (1·21–2·55)
Atrial fibrillation and flutter	50 to 54	Male	18.9 Cigarette-Equivalents	2·00 (1·35–2·91)
Atrial fibrillation and flutter	50 to 54	Male	25.2 Cigarette-Equivalents	2·26 (1·45–3·40)
Atrial fibrillation and flutter	55 to 59	Male	0 Cigarette-Equivalents	1·00 (1·00–1·00)
Atrial fibrillation and flutter	55 to 59	Male	6.3 Cigarette-Equivalents	1·47 (1·03–1·94)
Atrial fibrillation and flutter	55 to 59	Male	12.6 Cigarette-Equivalents	1·63 (1·15–2·24)
Atrial fibrillation and flutter	55 to 59	Male	18.9 Cigarette-Equivalents	1·77 (1·25–2·48)

Atrial fibrillation and flutter	55 to 59	Male	25.2 Cigarette-Equivalents	1·95 (1·35–2·87)
Atrial fibrillation and flutter	55 to 59	Female	0 Cigarette-Equivalents	1·00 (1·00–1·00)
Atrial fibrillation and flutter	55 to 59	Female	6.3 Cigarette-Equivalents	1·47 (1·03–1·94)
Atrial fibrillation and flutter	55 to 59	Female	12.6 Cigarette-Equivalents	1·63 (1·15–2·24)
Atrial fibrillation and flutter	55 to 59	Female	18.9 Cigarette-Equivalents	1·77 (1·25–2·48)
Atrial fibrillation and flutter	55 to 59	Female	25.2 Cigarette-Equivalents	1·95 (1·35–2·87)
Atrial fibrillation and flutter	60 to 64	Female	0 Cigarette-Equivalents	1·00 (1·00–1·00)
Atrial fibrillation and flutter	60 to 64	Female	6.3 Cigarette-Equivalents	1·47 (1·03–1·94)
Atrial fibrillation and flutter	60 to 64	Female	12.6 Cigarette-Equivalents	1·63 (1·15–2·24)
Atrial fibrillation and flutter	60 to 64	Female	18.9 Cigarette-Equivalents	1·77 (1·25–2·48)
Atrial fibrillation and flutter	60 to 64	Female	25.2 Cigarette-Equivalents	1·95 (1·35–2·87)
Atrial fibrillation and flutter	60 to 64	Male	0 Cigarette-Equivalents	1·00 (1·00–1·00)
Atrial fibrillation and flutter	60 to 64	Male	6.3 Cigarette-Equivalents	1·47 (1·03–1·94)
Atrial fibrillation and flutter	60 to 64	Male	12.6 Cigarette-Equivalents	1·63 (1·15–2·24)
Atrial fibrillation and flutter	60 to 64	Male	18.9 Cigarette-Equivalents	1·77 (1·25–2·48)
Atrial fibrillation and flutter	60 to 64	Male	25.2 Cigarette-Equivalents	1·95 (1·35–2·87)
Atrial fibrillation and flutter	65 to 69	Male	0 Cigarette-Equivalents	1·00 (1·00–1·00)
Atrial fibrillation and flutter	65 to 69	Male	6.3 Cigarette-Equivalents	1·43 (1·04–1·79)
Atrial fibrillation and flutter	65 to 69	Male	12.6 Cigarette-Equivalents	1·46 (1·10–1·90)
Atrial fibrillation and flutter	65 to 69	Male	18.9 Cigarette-Equivalents	1·52 (1·13–2·05)
Atrial fibrillation and flutter	65 to 69	Male	25.2 Cigarette-Equivalents	1·63 (1·23–2·19)
Atrial fibrillation and flutter	65 to 69	Female	0 Cigarette-Equivalents	1·00 (1·00–1·00)
Atrial fibrillation and flutter	65 to 69	Female	6.3 Cigarette-Equivalents	1·43 (1·04–1·79)
Atrial fibrillation and flutter	65 to 69	Female	12.6 Cigarette-Equivalents	1·46 (1·10–1·90)
Atrial fibrillation and flutter	65 to 69	Female	18.9 Cigarette-Equivalents	1·52 (1·13–2·05)
Atrial fibrillation and flutter	65 to 69	Female	25.2 Cigarette-Equivalents	1·63 (1·23–2·19)
Atrial fibrillation and flutter	70 to 74	Female	0 Cigarette-Equivalents	1·00 (1·00–1·00)

Atrial fibrillation and flutter	70 to 74	Female	6.3 Cigarette-Equivalents	1·43 (1·04–1·79)
Atrial fibrillation and flutter	70 to 74	Female	12.6 Cigarette-Equivalents	1·46 (1·10–1·90)
Atrial fibrillation and flutter	70 to 74	Female	18.9 Cigarette-Equivalents	1·52 (1·13–2·05)
Atrial fibrillation and flutter	70 to 74	Female	25.2 Cigarette-Equivalents	1·63 (1·23–2·19)
Atrial fibrillation and flutter	70 to 74	Male	0 Cigarette-Equivalents	1·00 (1·00–1·00)
Atrial fibrillation and flutter	70 to 74	Male	6.3 Cigarette-Equivalents	1·43 (1·04–1·79)
Atrial fibrillation and flutter	70 to 74	Male	12.6 Cigarette-Equivalents	1·46 (1·10–1·90)
Atrial fibrillation and flutter	70 to 74	Male	18.9 Cigarette-Equivalents	1·52 (1·13–2·05)
Atrial fibrillation and flutter	70 to 74	Male	25.2 Cigarette-Equivalents	1·63 (1·23–2·19)
Atrial fibrillation and flutter	75 to 79	Male	0 Cigarette-Equivalents	1·00 (1·00–1·00)
Atrial fibrillation and flutter	75 to 79	Male	6.3 Cigarette-Equivalents	1·32 (1·05–1·59)
Atrial fibrillation and flutter	75 to 79	Male	12.6 Cigarette-Equivalents	1·28 (1·02–1·61)
Atrial fibrillation and flutter	75 to 79	Male	18.9 Cigarette-Equivalents	1·30 (1·00–1·67)
Atrial fibrillation and flutter	75 to 79	Male	25.2 Cigarette-Equivalents	1·37 (1·10–1·74)
Atrial fibrillation and flutter	75 to 79	Female	0 Cigarette-Equivalents	1·00 (1·00–1·00)
Atrial fibrillation and flutter	75 to 79	Female	6.3 Cigarette-Equivalents	1·32 (1·05–1·59)
Atrial fibrillation and flutter	75 to 79	Female	12.6 Cigarette-Equivalents	1·28 (1·02–1·61)
Atrial fibrillation and flutter	75 to 79	Female	18.9 Cigarette-Equivalents	1·30 (1·00–1·67)
Atrial fibrillation and flutter	75 to 79	Female	25.2 Cigarette-Equivalents	1·37 (1·10–1·74)
Atrial fibrillation and flutter	80 to 84	Female	0 Cigarette-Equivalents	1·00 (1·00–1·00)
Atrial fibrillation and flutter	80 to 84	Female	6.3 Cigarette-Equivalents	1·32 (1·05–1·59)
Atrial fibrillation and flutter	80 to 84	Female	12.6 Cigarette-Equivalents	1·28 (1·02–1·61)
Atrial fibrillation and flutter	80 to 84	Female	18.9 Cigarette-Equivalents	1·30 (1·00–1·67)
Atrial fibrillation and flutter	80 to 84	Female	25.2 Cigarette-Equivalents	1·37 (1·10–1·74)
Atrial fibrillation and flutter	80 to 84	Male	0 Cigarette-Equivalents	1·00 (1·00–1·00)
Atrial fibrillation and flutter	80 to 84	Male	6.3 Cigarette-Equivalents	1·32 (1·05–1·59)
Atrial fibrillation and flutter	80 to 84	Male	12.6 Cigarette-Equivalents	1·28 (1·02–1·61)

Atrial fibrillation and flutter	80 to 84	Male	18.9 Cigarette-Equivalents	1·30 (1·00–1·67)
Atrial fibrillation and flutter	80 to 84	Male	25.2 Cigarette-Equivalents	1·37 (1·10–1·74)
Atrial fibrillation and flutter	85 to 89	Male	0 Cigarette-Equivalents	1·00 (1·00–1·00)
Atrial fibrillation and flutter	85 to 89	Male	6.3 Cigarette-Equivalents	1·18 (1·00–1·36)
Atrial fibrillation and flutter	85 to 89	Male	12.6 Cigarette-Equivalents	1·13 (1·00–1·32)
Atrial fibrillation and flutter	85 to 89	Male	18.9 Cigarette-Equivalents	1·12 (1·00–1·38)
Atrial fibrillation and flutter	85 to 89	Male	25.2 Cigarette-Equivalents	1·16 (1·00–1·37)
Atrial fibrillation and flutter	85 to 89	Female	0 Cigarette-Equivalents	1·00 (1·00–1·00)
Atrial fibrillation and flutter	85 to 89	Female	6.3 Cigarette-Equivalents	1·18 (1·00–1·36)
Atrial fibrillation and flutter	85 to 89	Female	12.6 Cigarette-Equivalents	1·13 (1·00–1·32)
Atrial fibrillation and flutter	85 to 89	Female	18.9 Cigarette-Equivalents	1·12 (1·00–1·38)
Atrial fibrillation and flutter	85 to 89	Female	25.2 Cigarette-Equivalents	1·16 (1·00–1·37)
Atrial fibrillation and flutter	90 to 94	Female	0 Cigarette-Equivalents	1·00 (1·00–1·00)
Atrial fibrillation and flutter	90 to 94	Female	6.3 Cigarette-Equivalents	1·18 (1·00–1·36)
Atrial fibrillation and flutter	90 to 94	Female	12.6 Cigarette-Equivalents	1·13 (1·00–1·32)
Atrial fibrillation and flutter	90 to 94	Female	18.9 Cigarette-Equivalents	1·12 (1·00–1·38)
Atrial fibrillation and flutter	90 to 94	Female	25.2 Cigarette-Equivalents	1·16 (1·00–1·37)
Atrial fibrillation and flutter	90 to 94	Male	0 Cigarette-Equivalents	1·00 (1·00–1·00)
Atrial fibrillation and flutter	90 to 94	Male	6.3 Cigarette-Equivalents	1·18 (1·00–1·36)
Atrial fibrillation and flutter	90 to 94	Male	12.6 Cigarette-Equivalents	1·13 (1·00–1·32)
Atrial fibrillation and flutter	90 to 94	Male	18.9 Cigarette-Equivalents	1·12 (1·00–1·38)
Atrial fibrillation and flutter	90 to 94	Male	25.2 Cigarette-Equivalents	1·16 (1·00–1·37)
Atrial fibrillation and flutter	95 plus	Male	0 Cigarette-Equivalents	1·00 (1·00–1·00)
Atrial fibrillation and flutter	95 plus	Male	6.3 Cigarette-Equivalents	1·01 (1·00–1·05)
Atrial fibrillation and flutter	95 plus	Male	12.6 Cigarette-Equivalents	1·02 (1·00–1·11)
Atrial fibrillation and flutter	95 plus	Male	18.9 Cigarette-Equivalents	1·03 (1·00–1·16)
Atrial fibrillation and flutter	95 plus	Male	25.2 Cigarette-Equivalents	1·01 (1·00–1·09)

Atrial fibrillation and flutter	95 plus	Female	0 Cigarette-Equivalents	1·00 (1·00–1·00)
Atrial fibrillation and flutter	95 plus	Female	6.3 Cigarette-Equivalents	1·01 (1·00–1·05)
Atrial fibrillation and flutter	95 plus	Female	12.6 Cigarette-Equivalents	1·02 (1·00–1·11)
Atrial fibrillation and flutter	95 plus	Female	18.9 Cigarette-Equivalents	1·03 (1·00–1·16)
Atrial fibrillation and flutter	95 plus	Female	25.2 Cigarette-Equivalents	1·01 (1·00–1·09)
Aortic aneurysm	30 to 34	Female	0 Cigarette-Equivalents	1·00 (1·00–1·00)
Aortic aneurysm	30 to 34	Female	12 Cigarette-Equivalents	5·05 (3·68–6·52)
Aortic aneurysm	30 to 34	Female	24 Cigarette-Equivalents	9·53 (6·98–12·5)
Aortic aneurysm	30 to 34	Female	36 Cigarette-Equivalents	12·4 (7·77–18·2)
Aortic aneurysm	30 to 34	Female	48 Cigarette-Equivalents	14·5 (7·45–24·3)
Aortic aneurysm	30 to 34	Male	0 Cigarette-Equivalents	1·00 (1·00–1·00)
Aortic aneurysm	30 to 34	Male	12 Cigarette-Equivalents	5·05 (3·68–6·52)
Aortic aneurysm	30 to 34	Male	24 Cigarette-Equivalents	9·53 (6·98–12·5)
Aortic aneurysm	30 to 34	Male	36 Cigarette-Equivalents	12·4 (7·77–18·2)
Aortic aneurysm	30 to 34	Male	48 Cigarette-Equivalents	14·5 (7·45–24·3)
Aortic aneurysm	35 to 39	Male	0 Cigarette-Equivalents	1·00 (1·00–1·00)
Aortic aneurysm	35 to 39	Male	12 Cigarette-Equivalents	5·05 (3·68–6·52)
Aortic aneurysm	35 to 39	Male	24 Cigarette-Equivalents	9·53 (6·98–12·5)
Aortic aneurysm	35 to 39	Male	36 Cigarette-Equivalents	12·4 (7·77–18·2)
Aortic aneurysm	35 to 39	Male	48 Cigarette-Equivalents	14·5 (7·45–24·3)
Aortic aneurysm	35 to 39	Female	0 Cigarette-Equivalents	1·00 (1·00–1·00)
Aortic aneurysm	35 to 39	Female	12 Cigarette-Equivalents	5·05 (3·68–6·52)
Aortic aneurysm	35 to 39	Female	24 Cigarette-Equivalents	9·53 (6·98–12·5)
Aortic aneurysm	35 to 39	Female	36 Cigarette-Equivalents	12·4 (7·77–18·2)
Aortic aneurysm	35 to 39	Female	48 Cigarette-Equivalents	14·5 (7·45–24·3)
Aortic aneurysm	40 to 44	Female	0 Cigarette-Equivalents	1·00 (1·00–1·00)
Aortic aneurysm	40 to 44	Female	12 Cigarette-Equivalents	5·05 (3·68–6·52)

Aortic aneurysm	40 to 44	Female	24 Cigarette-Equivalents	9·53 (6·98–12·5)
Aortic aneurysm	40 to 44	Female	36 Cigarette-Equivalents	12·4 (7·77–18·2)
Aortic aneurysm	40 to 44	Female	48 Cigarette-Equivalents	14·5 (7·45–24·3)
Aortic aneurysm	40 to 44	Male	0 Cigarette-Equivalents	1·00 (1·00–1·00)
Aortic aneurysm	40 to 44	Male	12 Cigarette-Equivalents	5·05 (3·68–6·52)
Aortic aneurysm	40 to 44	Male	24 Cigarette-Equivalents	9·53 (6·98–12·5)
Aortic aneurysm	40 to 44	Male	36 Cigarette-Equivalents	12·4 (7·77–18·2)
Aortic aneurysm	40 to 44	Male	48 Cigarette-Equivalents	14·5 (7·45–24·3)
Aortic aneurysm	45 to 49	Male	0 Cigarette-Equivalents	1·00 (1·00–1·00)
Aortic aneurysm	45 to 49	Male	12 Cigarette-Equivalents	4·20 (3·21–5·30)
Aortic aneurysm	45 to 49	Male	24 Cigarette-Equivalents	6·72 (5·10–8·60)
Aortic aneurysm	45 to 49	Male	36 Cigarette-Equivalents	8·51 (5·66–11·9)
Aortic aneurysm	45 to 49	Male	48 Cigarette-Equivalents	9·77 (5·93–15·1)
Aortic aneurysm	45 to 49	Female	0 Cigarette-Equivalents	1·00 (1·00–1·00)
Aortic aneurysm	45 to 49	Female	12 Cigarette-Equivalents	4·20 (3·21–5·30)
Aortic aneurysm	45 to 49	Female	24 Cigarette-Equivalents	6·72 (5·10–8·60)
Aortic aneurysm	45 to 49	Female	36 Cigarette-Equivalents	8·51 (5·66–11·9)
Aortic aneurysm	45 to 49	Female	48 Cigarette-Equivalents	9·77 (5·93–15·1)
Aortic aneurysm	50 to 54	Female	0 Cigarette-Equivalents	1·00 (1·00–1·00)
Aortic aneurysm	50 to 54	Female	12 Cigarette-Equivalents	4·20 (3·21–5·30)
Aortic aneurysm	50 to 54	Female	24 Cigarette-Equivalents	6·72 (5·10–8·60)
Aortic aneurysm	50 to 54	Female	36 Cigarette-Equivalents	8·51 (5·66–11·9)
Aortic aneurysm	50 to 54	Female	48 Cigarette-Equivalents	9·77 (5·93–15·1)
Aortic aneurysm	50 to 54	Male	0 Cigarette-Equivalents	1·00 (1·00–1·00)
Aortic aneurysm	50 to 54	Male	12 Cigarette-Equivalents	4·20 (3·21–5·30)
Aortic aneurysm	50 to 54	Male	24 Cigarette-Equivalents	6·72 (5·10–8·60)
Aortic aneurysm	50 to 54	Male	36 Cigarette-Equivalents	8·51 (5·66–11·9)

Aortic aneurysm	50 to 54	Male	48 Cigarette-Equivalents	9·77 (5·93–15·1)
Aortic aneurysm	55 to 59	Male	0 Cigarette-Equivalents	1·00 (1·00–1·00)
Aortic aneurysm	55 to 59	Male	12 Cigarette-Equivalents	3·40 (2·71–4·16)
Aortic aneurysm	55 to 59	Male	24 Cigarette-Equivalents	4·62 (3·71–5·76)
Aortic aneurysm	55 to 59	Male	36 Cigarette-Equivalents	5·63 (3·93–7·70)
Aortic aneurysm	55 to 59	Male	48 Cigarette-Equivalents	6·26 (4·09–9·24)
Aortic aneurysm	55 to 59	Female	0 Cigarette-Equivalents	1·00 (1·00–1·00)
Aortic aneurysm	55 to 59	Female	12 Cigarette-Equivalents	3·40 (2·71–4·16)
Aortic aneurysm	55 to 59	Female	24 Cigarette-Equivalents	4·62 (3·71–5·76)
Aortic aneurysm	55 to 59	Female	36 Cigarette-Equivalents	5·63 (3·93–7·70)
Aortic aneurysm	55 to 59	Female	48 Cigarette-Equivalents	6·26 (4·09–9·24)
Aortic aneurysm	60 to 64	Female	0 Cigarette-Equivalents	1·00 (1·00–1·00)
Aortic aneurysm	60 to 64	Female	12 Cigarette-Equivalents	3·40 (2·71–4·16)
Aortic aneurysm	60 to 64	Female	24 Cigarette-Equivalents	4·62 (3·71–5·76)
Aortic aneurysm	60 to 64	Female	36 Cigarette-Equivalents	5·63 (3·93–7·70)
Aortic aneurysm	60 to 64	Female	48 Cigarette-Equivalents	6·26 (4·09–9·24)
Aortic aneurysm	60 to 64	Male	0 Cigarette-Equivalents	1·00 (1·00–1·00)
Aortic aneurysm	60 to 64	Male	12 Cigarette-Equivalents	3·40 (2·71–4·16)
Aortic aneurysm	60 to 64	Male	24 Cigarette-Equivalents	4·62 (3·71–5·76)
Aortic aneurysm	60 to 64	Male	36 Cigarette-Equivalents	5·63 (3·93–7·70)
Aortic aneurysm	60 to 64	Male	48 Cigarette-Equivalents	6·26 (4·09–9·24)
Aortic aneurysm	65 to 69	Male	0 Cigarette-Equivalents	1·00 (1·00–1·00)
Aortic aneurysm	65 to 69	Male	12 Cigarette-Equivalents	2·67 (2·26–3·04)
Aortic aneurysm	65 to 69	Male	24 Cigarette-Equivalents	3·12 (2·58–3·72)
Aortic aneurysm	65 to 69	Male	36 Cigarette-Equivalents	3·70 (2·78–4·80)
Aortic aneurysm	65 to 69	Male	48 Cigarette-Equivalents	3·95 (2·76–5·44)
Aortic aneurysm	65 to 69	Female	0 Cigarette-Equivalents	1·00 (1·00–1·00)

Aortic aneurysm	65 to 69	Female	12 Cigarette-Equivalents	2·67 (2·26–3·04)
Aortic aneurysm	65 to 69	Female	24 Cigarette-Equivalents	3·12 (2·58–3·72)
Aortic aneurysm	65 to 69	Female	36 Cigarette-Equivalents	3·70 (2·78–4·80)
Aortic aneurysm	65 to 69	Female	48 Cigarette-Equivalents	3·95 (2·76–5·44)
Aortic aneurysm	70 to 74	Female	0 Cigarette-Equivalents	1·00 (1·00–1·00)
Aortic aneurysm	70 to 74	Female	12 Cigarette-Equivalents	2·67 (2·26–3·04)
Aortic aneurysm	70 to 74	Female	24 Cigarette-Equivalents	3·12 (2·58–3·72)
Aortic aneurysm	70 to 74	Female	36 Cigarette-Equivalents	3·70 (2·78–4·80)
Aortic aneurysm	70 to 74	Female	48 Cigarette-Equivalents	3·95 (2·76–5·44)
Aortic aneurysm	70 to 74	Male	0 Cigarette-Equivalents	1·00 (1·00–1·00)
Aortic aneurysm	70 to 74	Male	12 Cigarette-Equivalents	2·67 (2·26–3·04)
Aortic aneurysm	70 to 74	Male	24 Cigarette-Equivalents	3·12 (2·58–3·72)
Aortic aneurysm	70 to 74	Male	36 Cigarette-Equivalents	3·70 (2·78–4·80)
Aortic aneurysm	70 to 74	Male	48 Cigarette-Equivalents	3·95 (2·76–5·44)
Aortic aneurysm	75 to 79	Male	0 Cigarette-Equivalents	1·00 (1·00–1·00)
Aortic aneurysm	75 to 79	Male	12 Cigarette-Equivalents	2·01 (1·79–2·24)
Aortic aneurysm	75 to 79	Male	24 Cigarette-Equivalents	2·08 (1·80–2·40)
Aortic aneurysm	75 to 79	Male	36 Cigarette-Equivalents	2·46 (1·90–3·04)
Aortic aneurysm	75 to 79	Male	48 Cigarette-Equivalents	2·50 (1·88–3·30)
Aortic aneurysm	75 to 79	Female	0 Cigarette-Equivalents	1·00 (1·00–1·00)
Aortic aneurysm	75 to 79	Female	12 Cigarette-Equivalents	2·01 (1·79–2·24)
Aortic aneurysm	75 to 79	Female	24 Cigarette-Equivalents	2·08 (1·80–2·40)
Aortic aneurysm	75 to 79	Female	36 Cigarette-Equivalents	2·46 (1·90–3·04)
Aortic aneurysm	75 to 79	Female	48 Cigarette-Equivalents	2·50 (1·88–3·30)
Aortic aneurysm	80 to 84	Female	0 Cigarette-Equivalents	1·00 (1·00–1·00)
Aortic aneurysm	80 to 84	Female	12 Cigarette-Equivalents	2·01 (1·79–2·24)
Aortic aneurysm	80 to 84	Female	24 Cigarette-Equivalents	2·08 (1·80–2·40)

Aortic aneurysm	80 to 84	Female	36 Cigarette-Equivalents	2·46 (1·90–3·04)
Aortic aneurysm	80 to 84	Female	48 Cigarette-Equivalents	2·50 (1·88–3·30)
Aortic aneurysm	80 to 84	Male	0 Cigarette-Equivalents	1·00 (1·00–1·00)
Aortic aneurysm	80 to 84	Male	12 Cigarette-Equivalents	2·01 (1·79–2·24)
Aortic aneurysm	80 to 84	Male	24 Cigarette-Equivalents	2·08 (1·80–2·40)
Aortic aneurysm	80 to 84	Male	36 Cigarette-Equivalents	2·46 (1·90–3·04)
Aortic aneurysm	80 to 84	Male	48 Cigarette-Equivalents	2·50 (1·88–3·30)
Aortic aneurysm	85 to 89	Male	0 Cigarette-Equivalents	1·00 (1·00–1·00)
Aortic aneurysm	85 to 89	Male	12 Cigarette-Equivalents	1·44 (1·33–1·56)
Aortic aneurysm	85 to 89	Male	24 Cigarette-Equivalents	1·39 (1·24–1·55)
Aortic aneurysm	85 to 89	Male	36 Cigarette-Equivalents	1·59 (1·31–1·88)
Aortic aneurysm	85 to 89	Male	48 Cigarette-Equivalents	1·56 (1·22–1·95)
Aortic aneurysm	85 to 89	Female	0 Cigarette-Equivalents	1·00 (1·00–1·00)
Aortic aneurysm	85 to 89	Female	12 Cigarette-Equivalents	1·44 (1·33–1·56)
Aortic aneurysm	85 to 89	Female	24 Cigarette-Equivalents	1·39 (1·24–1·55)
Aortic aneurysm	85 to 89	Female	36 Cigarette-Equivalents	1·59 (1·31–1·88)
Aortic aneurysm	85 to 89	Female	48 Cigarette-Equivalents	1·56 (1·22–1·95)
Aortic aneurysm	90 to 94	Female	0 Cigarette-Equivalents	1·00 (1·00–1·00)
Aortic aneurysm	90 to 94	Female	12 Cigarette-Equivalents	1·44 (1·33–1·56)
Aortic aneurysm	90 to 94	Female	24 Cigarette-Equivalents	1·39 (1·24–1·55)
Aortic aneurysm	90 to 94	Female	36 Cigarette-Equivalents	1·59 (1·31–1·88)
Aortic aneurysm	90 to 94	Female	48 Cigarette-Equivalents	1·56 (1·22–1·95)
Aortic aneurysm	90 to 94	Male	0 Cigarette-Equivalents	1·00 (1·00–1·00)
Aortic aneurysm	90 to 94	Male	12 Cigarette-Equivalents	1·44 (1·33–1·56)
Aortic aneurysm	90 to 94	Male	24 Cigarette-Equivalents	1·39 (1·24–1·55)
Aortic aneurysm	90 to 94	Male	36 Cigarette-Equivalents	1·59 (1·31–1·88)
Aortic aneurysm	90 to 94	Male	48 Cigarette-Equivalents	1·56 (1·22–1·95)

Aortic aneurysm	95 plus	Male	0 Cigarette-Equivalents	1·00 (1·00–1·00)
Aortic aneurysm	95 plus	Male	12 Cigarette-Equivalents	1·01 (1·00–1·03)
Aortic aneurysm	95 plus	Male	24 Cigarette-Equivalents	1·00 (1·00–1·02)
Aortic aneurysm	95 plus	Male	36 Cigarette-Equivalents	1·01 (1·00–1·03)
Aortic aneurysm	95 plus	Male	48 Cigarette-Equivalents	1·03 (1·00–1·16)
Aortic aneurysm	95 plus	Female	0 Cigarette-Equivalents	1·00 (1·00–1·00)
Aortic aneurysm	95 plus	Female	12 Cigarette-Equivalents	1·01 (1·00–1·03)
Aortic aneurysm	95 plus	Female	24 Cigarette-Equivalents	1·00 (1·00–1·02)
Aortic aneurysm	95 plus	Female	36 Cigarette-Equivalents	1·01 (1·00–1·03)
Aortic aneurysm	95 plus	Female	48 Cigarette-Equivalents	1·03 (1·00–1·16)
Lower extremity peripheral arterial disease	30 to 34	Female	0 Cigarette-Equivalents	1·00 (1·00–1·00)
Lower extremity peripheral arterial disease	30 to 34	Female	9.3 Cigarette-Equivalents	3·03 (1·98–4·60)
Lower extremity peripheral arterial disease	30 to 34	Female	18.6 Cigarette-Equivalents	6·37 (3·69–10·3)
Lower extremity peripheral arterial disease	30 to 34	Female	27.9 Cigarette-Equivalents	9·00 (4·62–15·9)
Lower extremity peripheral arterial disease	30 to 34	Female	37.2 Cigarette-Equivalents	10·8 (4·70–21·0)
Lower extremity peripheral arterial disease	30 to 34	Female	46.5 Cigarette-Equivalents	12·0 (4·66–25·3)
Lower extremity peripheral arterial disease	30 to 34	Male	0 Cigarette-Equivalents	1·00 (1·00–1·00)
Lower extremity peripheral arterial disease	30 to 34	Male	9.3 Cigarette-Equivalents	3·03 (1·98–4·60)
Lower extremity peripheral arterial disease	30 to 34	Male	18.6 Cigarette-Equivalents	6·37 (3·69–10·3)
Lower extremity peripheral arterial disease	30 to 34	Male	27.9 Cigarette-Equivalents	9·00 (4·62–15·9)
Lower extremity peripheral arterial disease	30 to 34	Male	37.2 Cigarette-Equivalents	10·8 (4·70–21·0)
Lower extremity peripheral arterial disease	30 to 34	Male	46.5 Cigarette-Equivalents	12·0 (4·66–25·3)
Lower extremity peripheral arterial disease	35 to 39	Male	0 Cigarette-Equivalents	1·00 (1·00–1·00)
Lower extremity peripheral arterial disease	35 to 39	Male	9.3 Cigarette-Equivalents	3·03 (1·98–4·60)
Lower extremity peripheral arterial disease	35 to 39	Male	18.6 Cigarette-Equivalents	6·37 (3·69–10·3)
Lower extremity peripheral arterial disease	35 to 39	Male	27.9 Cigarette-Equivalents	9·00 (4·62–15·9)
Lower extremity peripheral arterial disease	35 to 39	Male	37.2 Cigarette-Equivalents	10·8 (4·70–21·0)

Lower extremity peripheral arterial disease	35 to 39	Male	46.5 Cigarette-Equivalents	12·0 (4·66–25·3)
Lower extremity peripheral arterial disease	35 to 39	Female	0 Cigarette-Equivalents	1·00 (1·00–1·00)
Lower extremity peripheral arterial disease	35 to 39	Female	9.3 Cigarette-Equivalents	3·03 (1·98–4·60)
Lower extremity peripheral arterial disease	35 to 39	Female	18.6 Cigarette-Equivalents	6·37 (3·69–10·3)
Lower extremity peripheral arterial disease	35 to 39	Female	27.9 Cigarette-Equivalents	9·00 (4·62–15·9)
Lower extremity peripheral arterial disease	35 to 39	Female	37.2 Cigarette-Equivalents	10·8 (4·70–21·0)
Lower extremity peripheral arterial disease	35 to 39	Female	46.5 Cigarette-Equivalents	12·0 (4·66–25·3)
Lower extremity peripheral arterial disease	40 to 44	Female	0 Cigarette-Equivalents	1·00 (1·00–1·00)
Lower extremity peripheral arterial disease	40 to 44	Female	9.3 Cigarette-Equivalents	3·03 (1·98–4·60)
Lower extremity peripheral arterial disease	40 to 44	Female	18.6 Cigarette-Equivalents	6·37 (3·69–10·3)
Lower extremity peripheral arterial disease	40 to 44	Female	27.9 Cigarette-Equivalents	9·00 (4·62–15·9)
Lower extremity peripheral arterial disease	40 to 44	Female	37.2 Cigarette-Equivalents	10·8 (4·70–21·0)
Lower extremity peripheral arterial disease	40 to 44	Female	46.5 Cigarette-Equivalents	12·0 (4·66–25·3)
Lower extremity peripheral arterial disease	40 to 44	Male	0 Cigarette-Equivalents	1·00 (1·00–1·00)
Lower extremity peripheral arterial disease	40 to 44	Male	9.3 Cigarette-Equivalents	3·03 (1·98–4·60)
Lower extremity peripheral arterial disease	40 to 44	Male	18.6 Cigarette-Equivalents	6·37 (3·69–10·3)
Lower extremity peripheral arterial disease	40 to 44	Male	27.9 Cigarette-Equivalents	9·00 (4·62–15·9)
Lower extremity peripheral arterial disease	40 to 44	Male	37.2 Cigarette-Equivalents	10·8 (4·70–21·0)
Lower extremity peripheral arterial disease	40 to 44	Male	46.5 Cigarette-Equivalents	12·0 (4·66–25·3)
Lower extremity peripheral arterial disease	45 to 49	Male	0 Cigarette-Equivalents	1·00 (1·00–1·00)
Lower extremity peripheral arterial disease	45 to 49	Male	9.3 Cigarette-Equivalents	2·78 (1·76–4·24)
Lower extremity peripheral arterial disease	45 to 49	Male	18.6 Cigarette-Equivalents	5·44 (3·03–8·94)
Lower extremity peripheral arterial disease	45 to 49	Male	27.9 Cigarette-Equivalents	7·43 (3·68–13·2)
Lower extremity peripheral arterial disease	45 to 49	Male	37.2 Cigarette-Equivalents	8·84 (3·84–18·1)
Lower extremity peripheral arterial disease	45 to 49	Male	46.5 Cigarette-Equivalents	9·78 (3·82–21·0)
Lower extremity peripheral arterial disease	45 to 49	Female	0 Cigarette-Equivalents	1·00 (1·00–1·00)
Lower extremity peripheral arterial disease	45 to 49	Female	9.3 Cigarette-Equivalents	2·78 (1·76–4·24)

Lower extremity peripheral arterial disease	45 to 49	Female	18.6 Cigarette-Equivalents	5·44 (3·03–8·94)
Lower extremity peripheral arterial disease	45 to 49	Female	27.9 Cigarette-Equivalents	7·43 (3·68–13·2)
Lower extremity peripheral arterial disease	45 to 49	Female	37.2 Cigarette-Equivalents	8·84 (3·84–18·1)
Lower extremity peripheral arterial disease	45 to 49	Female	46.5 Cigarette-Equivalents	9·78 (3·82–21·0)
Lower extremity peripheral arterial disease	50 to 54	Female	0 Cigarette-Equivalents	1·00 (1·00–1·00)
Lower extremity peripheral arterial disease	50 to 54	Female	9.3 Cigarette-Equivalents	2·78 (1·76–4·24)
Lower extremity peripheral arterial disease	50 to 54	Female	18.6 Cigarette-Equivalents	5·44 (3·03–8·94)
Lower extremity peripheral arterial disease	50 to 54	Female	27.9 Cigarette-Equivalents	7·43 (3·68–13·2)
Lower extremity peripheral arterial disease	50 to 54	Female	37.2 Cigarette-Equivalents	8·84 (3·84–18·1)
Lower extremity peripheral arterial disease	50 to 54	Female	46.5 Cigarette-Equivalents	9·78 (3·82–21·0)
Lower extremity peripheral arterial disease	50 to 54	Male	0 Cigarette-Equivalents	1·00 (1·00–1·00)
Lower extremity peripheral arterial disease	50 to 54	Male	9.3 Cigarette-Equivalents	2·78 (1·76–4·24)
Lower extremity peripheral arterial disease	50 to 54	Male	18.6 Cigarette-Equivalents	5·44 (3·03–8·94)
Lower extremity peripheral arterial disease	50 to 54	Male	27.9 Cigarette-Equivalents	7·43 (3·68–13·2)
Lower extremity peripheral arterial disease	50 to 54	Male	37.2 Cigarette-Equivalents	8·84 (3·84–18·1)
Lower extremity peripheral arterial disease	50 to 54	Male	46.5 Cigarette-Equivalents	9·78 (3·82–21·0)
Lower extremity peripheral arterial disease	55 to 59	Male	0 Cigarette-Equivalents	1·00 (1·00–1·00)
Lower extremity peripheral arterial disease	55 to 59	Male	9.3 Cigarette-Equivalents	3·08 (1·72–4·74)
Lower extremity peripheral arterial disease	55 to 59	Male	18.6 Cigarette-Equivalents	5·69 (2·72–8·82)
Lower extremity peripheral arterial disease	55 to 59	Male	27.9 Cigarette-Equivalents	7·07 (3·10–11·2)
Lower extremity peripheral arterial disease	55 to 59	Male	37.2 Cigarette-Equivalents	8·20 (3·61–14·7)
Lower extremity peripheral arterial disease	55 to 59	Male	46.5 Cigarette-Equivalents	9·03 (3·61–19·0)
Lower extremity peripheral arterial disease	55 to 59	Female	0 Cigarette-Equivalents	1·00 (1·00–1·00)
Lower extremity peripheral arterial disease	55 to 59	Female	9.3 Cigarette-Equivalents	3·08 (1·72–4·74)
Lower extremity peripheral arterial disease	55 to 59	Female	18.6 Cigarette-Equivalents	5·69 (2·72–8·82)
Lower extremity peripheral arterial disease	55 to 59	Female	27.9 Cigarette-Equivalents	7·07 (3·10–11·2)
Lower extremity peripheral arterial disease	55 to 59	Female	37.2 Cigarette-Equivalents	8·20 (3·61–14·7)

Lower extremity peripheral arterial disease	55 to 59	Female	46.5 Cigarette-Equivalents	9·03 (3·61–19·0)
Lower extremity peripheral arterial disease	60 to 64	Female	0 Cigarette-Equivalents	1·00 (1·00–1·00)
Lower extremity peripheral arterial disease	60 to 64	Female	9.3 Cigarette-Equivalents	3·08 (1·72–4·74)
Lower extremity peripheral arterial disease	60 to 64	Female	18.6 Cigarette-Equivalents	5·69 (2·72–8·82)
Lower extremity peripheral arterial disease	60 to 64	Female	27.9 Cigarette-Equivalents	7·07 (3·10–11·2)
Lower extremity peripheral arterial disease	60 to 64	Female	37.2 Cigarette-Equivalents	8·20 (3·61–14·7)
Lower extremity peripheral arterial disease	60 to 64	Female	46.5 Cigarette-Equivalents	9·03 (3·61–19·0)
Lower extremity peripheral arterial disease	60 to 64	Male	0 Cigarette-Equivalents	1·00 (1·00–1·00)
Lower extremity peripheral arterial disease	60 to 64	Male	9.3 Cigarette-Equivalents	3·08 (1·72–4·74)
Lower extremity peripheral arterial disease	60 to 64	Male	18.6 Cigarette-Equivalents	5·69 (2·72–8·82)
Lower extremity peripheral arterial disease	60 to 64	Male	27.9 Cigarette-Equivalents	7·07 (3·10–11·2)
Lower extremity peripheral arterial disease	60 to 64	Male	37.2 Cigarette-Equivalents	8·20 (3·61–14·7)
Lower extremity peripheral arterial disease	60 to 64	Male	46.5 Cigarette-Equivalents	9·03 (3·61–19·0)
Lower extremity peripheral arterial disease	65 to 69	Male	0 Cigarette-Equivalents	1·00 (1·00–1·00)
Lower extremity peripheral arterial disease	65 to 69	Male	9.3 Cigarette-Equivalents	2·98 (2·18–3·75)
Lower extremity peripheral arterial disease	65 to 69	Male	18.6 Cigarette-Equivalents	4·87 (3·56–6·08)
Lower extremity peripheral arterial disease	65 to 69	Male	27.9 Cigarette-Equivalents	5·12 (3·76–6·66)
Lower extremity peripheral arterial disease	65 to 69	Male	37.2 Cigarette-Equivalents	5·97 (3·76–9·09)
Lower extremity peripheral arterial disease	65 to 69	Male	46.5 Cigarette-Equivalents	6·53 (3·54–11·1)
Lower extremity peripheral arterial disease	65 to 69	Female	0 Cigarette-Equivalents	1·00 (1·00–1·00)
Lower extremity peripheral arterial disease	65 to 69	Female	9.3 Cigarette-Equivalents	2·98 (2·18–3·75)
Lower extremity peripheral arterial disease	65 to 69	Female	18.6 Cigarette-Equivalents	4·87 (3·56–6·08)
Lower extremity peripheral arterial disease	65 to 69	Female	27.9 Cigarette-Equivalents	5·12 (3·76–6·66)
Lower extremity peripheral arterial disease	65 to 69	Female	37.2 Cigarette-Equivalents	5·97 (3·76–9·09)
Lower extremity peripheral arterial disease	65 to 69	Female	46.5 Cigarette-Equivalents	6·53 (3·54–11·1)
Lower extremity peripheral arterial disease	70 to 74	Female	0 Cigarette-Equivalents	1·00 (1·00–1·00)
Lower extremity peripheral arterial disease	70 to 74	Female	9.3 Cigarette-Equivalents	2·98 (2·18–3·75)

Lower extremity peripheral arterial disease	70 to 74	Female	18.6 Cigarette-Equivalents	4·87 (3·56–6·08)
Lower extremity peripheral arterial disease	70 to 74	Female	27.9 Cigarette-Equivalents	5·12 (3·76–6·66)
Lower extremity peripheral arterial disease	70 to 74	Female	37.2 Cigarette-Equivalents	5·97 (3·76–9·09)
Lower extremity peripheral arterial disease	70 to 74	Female	46.5 Cigarette-Equivalents	6·53 (3·54–11·1)
Lower extremity peripheral arterial disease	70 to 74	Male	0 Cigarette-Equivalents	1·00 (1·00–1·00)
Lower extremity peripheral arterial disease	70 to 74	Male	9.3 Cigarette-Equivalents	2·98 (2·18–3·75)
Lower extremity peripheral arterial disease	70 to 74	Male	18.6 Cigarette-Equivalents	4·87 (3·56–6·08)
Lower extremity peripheral arterial disease	70 to 74	Male	27.9 Cigarette-Equivalents	5·12 (3·76–6·66)
Lower extremity peripheral arterial disease	70 to 74	Male	37.2 Cigarette-Equivalents	5·97 (3·76–9·09)
Lower extremity peripheral arterial disease	70 to 74	Male	46.5 Cigarette-Equivalents	6·53 (3·54–11·1)
Lower extremity peripheral arterial disease	75 to 79	Male	0 Cigarette-Equivalents	1·00 (1·00–1·00)
Lower extremity peripheral arterial disease	75 to 79	Male	9.3 Cigarette-Equivalents	2·19 (1·72–2·64)
Lower extremity peripheral arterial disease	75 to 79	Male	18.6 Cigarette-Equivalents	3·01 (2·44–3·56)
Lower extremity peripheral arterial disease	75 to 79	Male	27.9 Cigarette-Equivalents	2·89 (2·24–3·71)
Lower extremity peripheral arterial disease	75 to 79	Male	37.2 Cigarette-Equivalents	3·34 (2·18–4·95)
Lower extremity peripheral arterial disease	75 to 79	Male	46.5 Cigarette-Equivalents	3·63 (1·99–6·14)
Lower extremity peripheral arterial disease	75 to 79	Female	0 Cigarette-Equivalents	1·00 (1·00–1·00)
Lower extremity peripheral arterial disease	75 to 79	Female	9.3 Cigarette-Equivalents	2·19 (1·72–2·64)
Lower extremity peripheral arterial disease	75 to 79	Female	18.6 Cigarette-Equivalents	3·01 (2·44–3·56)
Lower extremity peripheral arterial disease	75 to 79	Female	27.9 Cigarette-Equivalents	2·89 (2·24–3·71)
Lower extremity peripheral arterial disease	75 to 79	Female	37.2 Cigarette-Equivalents	3·34 (2·18–4·95)
Lower extremity peripheral arterial disease	75 to 79	Female	46.5 Cigarette-Equivalents	3·63 (1·99–6·14)
Lower extremity peripheral arterial disease	80 to 84	Female	0 Cigarette-Equivalents	1·00 (1·00–1·00)
Lower extremity peripheral arterial disease	80 to 84	Female	9.3 Cigarette-Equivalents	2·19 (1·72–2·64)
Lower extremity peripheral arterial disease	80 to 84	Female	18.6 Cigarette-Equivalents	3·01 (2·44–3·56)
Lower extremity peripheral arterial disease	80 to 84	Female	27.9 Cigarette-Equivalents	2·89 (2·24–3·71)
Lower extremity peripheral arterial disease	80 to 84	Female	37.2 Cigarette-Equivalents	3·34 (2·18–4·95)

Lower extremity peripheral arterial disease	80 to 84	Female	46.5 Cigarette-Equivalents	3·63 (1·99–6·14)
Lower extremity peripheral arterial disease	80 to 84	Male	0 Cigarette-Equivalents	1·00 (1·00–1·00)
Lower extremity peripheral arterial disease	80 to 84	Male	9.3 Cigarette-Equivalents	2·19 (1·72–2·64)
Lower extremity peripheral arterial disease	80 to 84	Male	18.6 Cigarette-Equivalents	3·01 (2·44–3·56)
Lower extremity peripheral arterial disease	80 to 84	Male	27.9 Cigarette-Equivalents	2·89 (2·24–3·71)
Lower extremity peripheral arterial disease	80 to 84	Male	37.2 Cigarette-Equivalents	3·34 (2·18–4·95)
Lower extremity peripheral arterial disease	80 to 84	Male	46.5 Cigarette-Equivalents	3·63 (1·99–6·14)
Lower extremity peripheral arterial disease	85 to 89	Male	0 Cigarette-Equivalents	1·00 (1·00–1·00)
Lower extremity peripheral arterial disease	85 to 89	Male	9.3 Cigarette-Equivalents	1·54 (1·24–1·83)
Lower extremity peripheral arterial disease	85 to 89	Male	18.6 Cigarette-Equivalents	1·75 (1·45–2·08)
Lower extremity peripheral arterial disease	85 to 89	Male	27.9 Cigarette-Equivalents	1·65 (1·30–2·06)
Lower extremity peripheral arterial disease	85 to 89	Male	37.2 Cigarette-Equivalents	1·89 (1·25–2·83)
Lower extremity peripheral arterial disease	85 to 89	Male	46.5 Cigarette-Equivalents	2·06 (1·12–3·54)
Lower extremity peripheral arterial disease	85 to 89	Female	0 Cigarette-Equivalents	1·00 (1·00–1·00)
Lower extremity peripheral arterial disease	85 to 89	Female	9.3 Cigarette-Equivalents	1·54 (1·24–1·83)
Lower extremity peripheral arterial disease	85 to 89	Female	18.6 Cigarette-Equivalents	1·75 (1·45–2·08)
Lower extremity peripheral arterial disease	85 to 89	Female	27.9 Cigarette-Equivalents	1·65 (1·30–2·06)
Lower extremity peripheral arterial disease	85 to 89	Female	37.2 Cigarette-Equivalents	1·89 (1·25–2·83)
Lower extremity peripheral arterial disease	85 to 89	Female	46.5 Cigarette-Equivalents	2·06 (1·12–3·54)
Lower extremity peripheral arterial disease	90 to 94	Female	0 Cigarette-Equivalents	1·00 (1·00–1·00)
Lower extremity peripheral arterial disease	90 to 94	Female	9.3 Cigarette-Equivalents	1·54 (1·24–1·83)
Lower extremity peripheral arterial disease	90 to 94	Female	18.6 Cigarette-Equivalents	1·75 (1·45–2·08)
Lower extremity peripheral arterial disease	90 to 94	Female	27.9 Cigarette-Equivalents	1·65 (1·30–2·06)
Lower extremity peripheral arterial disease	90 to 94	Female	37.2 Cigarette-Equivalents	1·89 (1·25–2·83)
Lower extremity peripheral arterial disease	90 to 94	Female	46.5 Cigarette-Equivalents	2·06 (1·12–3·54)
Lower extremity peripheral arterial disease	90 to 94	Male	0 Cigarette-Equivalents	1·00 (1·00–1·00)
Lower extremity peripheral arterial disease	90 to 94	Male	9.3 Cigarette-Equivalents	1·54 (1·24–1·83)

Lower extremity peripheral arterial disease	90 to 94	Male	18.6 Cigarette-Equivalents	1·75 (1·45–2·08)
Lower extremity peripheral arterial disease	90 to 94	Male	27.9 Cigarette-Equivalents	1·65 (1·30–2·06)
Lower extremity peripheral arterial disease	90 to 94	Male	37.2 Cigarette-Equivalents	1·89 (1·25–2·83)
Lower extremity peripheral arterial disease	90 to 94	Male	46.5 Cigarette-Equivalents	2·06 (1·12–3·54)
Lower extremity peripheral arterial disease	95 plus	Male	0 Cigarette-Equivalents	1·00 (1·00–1·00)
Lower extremity peripheral arterial disease	95 plus	Male	9.3 Cigarette-Equivalents	1·04 (1·00–1·18)
Lower extremity peripheral arterial disease	95 plus	Male	18.6 Cigarette-Equivalents	1·03 (1·00–1·17)
Lower extremity peripheral arterial disease	95 plus	Male	27.9 Cigarette-Equivalents	1·05 (1·00–1·22)
Lower extremity peripheral arterial disease	95 plus	Male	37.2 Cigarette-Equivalents	1·16 (1·00–1·68)
Lower extremity peripheral arterial disease	95 plus	Male	46.5 Cigarette-Equivalents	1·25 (1·00–2·04)
Lower extremity peripheral arterial disease	95 plus	Female	0 Cigarette-Equivalents	1·00 (1·00–1·00)
Lower extremity peripheral arterial disease	95 plus	Female	9.3 Cigarette-Equivalents	1·04 (1·00–1·18)
Lower extremity peripheral arterial disease	95 plus	Female	18.6 Cigarette-Equivalents	1·03 (1·00–1·17)
Lower extremity peripheral arterial disease	95 plus	Female	27.9 Cigarette-Equivalents	1·05 (1·00–1·22)
Lower extremity peripheral arterial disease	95 plus	Female	37.2 Cigarette-Equivalents	1·16 (1·00–1·68)
Lower extremity peripheral arterial disease	95 plus	Female	46.5 Cigarette-Equivalents	1·25 (1·00–2·04)
Chronic obstructive pulmonary disease	All Ages	Both	0 Pack-Years	1·00 (1·00–1·00)
Chronic obstructive pulmonary disease	All Ages	Both	10 Pack-Years	3·56 (2·63–4·56)
Chronic obstructive pulmonary disease	All Ages	Both	20 Pack-Years	5·79 (4·36–7·36)
Chronic obstructive pulmonary disease	All Ages	Both	45 Pack-Years	7·86 (6·28–9·84)
Chronic obstructive pulmonary disease	All Ages	Both	60 Pack-Years	8·01 (4·24–12·7)
Chronic obstructive pulmonary disease	All Ages	Both	75 Pack-Years	10·1 (6·09–15·3)
Chronic obstructive pulmonary disease	All Ages	Both	90 Pack-Years	13·2 (7·98–20·7)
Asthma	All Ages	Both	0 Cigarette-Equivalents	1·00 (1·00–1·00)
Asthma	All Ages	Both	2.5 Cigarette-Equivalents	1·24 (1·00–1·52)
Asthma	All Ages	Both	7.5 Cigarette-Equivalents	1·66 (1·19–2·18)
Asthma	All Ages	Both	15 Cigarette-Equivalents	1·86 (1·32–2·40)

Asthma	All Ages	Both	22.5 Cigarette-Equivalents	1·99 (1·37–2·87)
Asthma	All Ages	Both	30 Cigarette-Equivalents	2·37 (1·45–3·85)
Peptic ulcer disease	All Ages	Both	0 Cigarette-Equivalents	1·00 (1·00–1·00)
Peptic ulcer disease	All Ages	Both	6.5 Cigarette-Equivalents	1·82 (1·46–2·20)
Peptic ulcer disease	All Ages	Both	13 Cigarette-Equivalents	2·16 (1·76–2·57)
Peptic ulcer disease	All Ages	Both	19.5 Cigarette-Equivalents	2·52 (2·07–2·99)
Peptic ulcer disease	All Ages	Both	26 Cigarette-Equivalents	2·55 (1·95–3·26)
Peptic ulcer disease	All Ages	Both	32.5 Cigarette-Equivalents	2·89 (2·15–3·74)
Gallbladder and biliary diseases	All Ages	Both	0 Cigarette-Equivalents	1·00 (1·00–1·00)
Gallbladder and biliary diseases	All Ages	Both	8.8 Cigarette-Equivalents	1·36 (1·20–1·53)
Gallbladder and biliary diseases	All Ages	Both	17.5 Cigarette-Equivalents	1·30 (1·18–1·43)
Gallbladder and biliary diseases	All Ages	Both	26.2 Cigarette-Equivalents	1·40 (1·24–1·56)
Gallbladder and biliary diseases	All Ages	Both	35 Cigarette-Equivalents	1·61 (1·30–1·96)
Gallbladder and biliary diseases	All Ages	Both	43.8 Cigarette-Equivalents	1·81 (1·45–2·16)
Alzheimer's disease and other dementias	All Ages	Both	0 Cigarette-Equivalents	1·00 (1·00–1·00)
Alzheimer's disease and other dementias	All Ages	Both	12 Cigarette-Equivalents	2·08 (1·40–2·84)
Alzheimer's disease and other dementias	All Ages	Both	24 Cigarette-Equivalents	2·94 (1·78–4·47)
Alzheimer's disease and other dementias	All Ages	Both	36 Cigarette-Equivalents	3·74 (1·97–6·02)
Alzheimer's disease and other dementias	All Ages	Both	48 Cigarette-Equivalents	4·10 (2·07–7·23)
Parkinson's disease	All Ages	Both	0 Cigarette-Equivalents	1·00 (1·00–1·00)
Parkinson's disease	All Ages	Both	7.5 Cigarette-Equivalents	0·835 (0·644–0·989)
Parkinson's disease	All Ages	Both	15 Cigarette-Equivalents	0·689 (0·531–0·869)
Parkinson's disease	All Ages	Both	22.5 Cigarette-Equivalents	0·604 (0·440–0·789)
Parkinson's disease	All Ages	Both	30 Cigarette-Equivalents	0·520 (0·346–0·710)
Parkinson's disease	All Ages	Both	37.5 Cigarette-Equivalents	0·432 (0·238–0·644)
Parkinson's disease	All Ages	Both	45 Cigarette-Equivalents	0·323 (0·126–0·561)
Multiple sclerosis	All Ages	Both	0 Cigarette-Equivalents	1·00 (1·00–1·00)

Multiple sclerosis	All Ages	Both	5.6 Cigarette-Equivalents	1·38 (1·15–1·62)
Multiple sclerosis	All Ages	Both	11.2 Cigarette-Equivalents	1·76 (1·49–2·05)
Multiple sclerosis	All Ages	Both	16.9 Cigarette-Equivalents	1·96 (1·65–2·29)
Multiple sclerosis	All Ages	Both	22.5 Cigarette-Equivalents	2·04 (1·58–2·55)
Diabetes mellitus	All Ages	Both	0 Cigarette-Equivalents	1·00 (1·00–1·00)
Diabetes mellitus	All Ages	Both	6.5 Cigarette-Equivalents	1·44 (1·21–1·65)
Diabetes mellitus	All Ages	Both	12.9 Cigarette-Equivalents	1·43 (1·13–1·76)
Diabetes mellitus	All Ages	Both	19.4 Cigarette-Equivalents	1·64 (1·25–2·03)
Diabetes mellitus	All Ages	Both	25.8 Cigarette-Equivalents	1·66 (1·35–1·99)
Diabetes mellitus	All Ages	Both	32.3 Cigarette-Equivalents	1·84 (1·22–2·46)
Diabetes mellitus	All Ages	Both	38.8 Cigarette-Equivalents	2·16 (1·16–3·35)
Rheumatoid arthritis	All Ages	Both	0 Cigarette-Equivalents	1·00 (1·00–1·00)
Rheumatoid arthritis	All Ages	Both	7.5 Cigarette-Equivalents	1·41 (1·00–1·96)
Rheumatoid arthritis	All Ages	Both	15 Cigarette-Equivalents	1·74 (1·10–2·63)
Rheumatoid arthritis	All Ages	Both	22.5 Cigarette-Equivalents	2·00 (1·17–3·19)
Rheumatoid arthritis	All Ages	Both	30 Cigarette-Equivalents	2·26 (1·15–3·91)
Rheumatoid arthritis	All Ages	Both	37.5 Cigarette-Equivalents	2·44 (1·15–4·71)
Low back pain	All Ages	Both	0 Cigarette-Equivalents	1·00 (1·00–1·00)
Low back pain	All Ages	Both	2.5 Cigarette-Equivalents	1·32 (1·10–1·56)
Low back pain	All Ages	Both	5 Cigarette-Equivalents	1·67 (1·39–1·98)
Low back pain	All Ages	Both	10 Cigarette-Equivalents	2·16 (1·75–2·64)
Low back pain	All Ages	Both	15 Cigarette-Equivalents	2·24 (1·80–2·81)
Low back pain	All Ages	Both	20 Cigarette-Equivalents	2·35 (1·61–3·31)
Cataract	All Ages	Both	0 Cigarette-Equivalents	1·00 (1·00–1·00)
Cataract	All Ages	Both	9 Cigarette-Equivalents	1·44 (1·29–1·61)
Cataract	All Ages	Both	18 Cigarette-Equivalents	1·48 (1·29–1·69)
Cataract	All Ages	Both	27 Cigarette-Equivalents	1·79 (1·36–2·27)

Cataract	All Ages	Both	36 Cigarette-Equivalents	1·88 (1·21–2·73)
Age-related macular degeneration	All Ages	Both	0 Cigarette-Equivalents	1·00 (1·00–1·00)
Age-related macular degeneration	All Ages	Both	7.5 Cigarette-Equivalents	1·35 (1·00–1·77)
Age-related macular degeneration	All Ages	Both	15 Cigarette-Equivalents	1·67 (1·15–2·35)
Age-related macular degeneration	All Ages	Both	22.5 Cigarette-Equivalents	2·02 (1·37–2·78)
Age-related macular degeneration	All Ages	Both	30 Cigarette-Equivalents	2·42 (1·62–3·35)
Fracture	All Ages	Both	Prevalence	1·85 (1·52–2·25)

Supplemental Table S1b. Results from dose-response meta-regressions for 36 health outcomes caused by smoking, at standardized doses.

Health Outcome	Age Group	Sex	Dose	Relative Risk
Tuberculosis	All Ages	Both	0 Cigarette-Equivalents	1·00 (1·00–1·00)
Tuberculosis	All Ages	Both	1 Cigarette-Equivalents	1·12 (1·07–1·17)
Tuberculosis	All Ages	Both	5 Cigarette-Equivalents	1·60 (1·34–1·86)
Tuberculosis	All Ages	Both	10 Cigarette-Equivalents	2·10 (1·68–2·52)
Tuberculosis	All Ages	Both	20 Cigarette-Equivalents	2·47 (2·03–2·97)
Tuberculosis	All Ages	Both	30 Cigarette-Equivalents	3·36 (2·61–4·34)
Lower respiratory infections	All Ages	Both	0 Cigarette-Equivalents	1·00 (1·00–1·00)
Lower respiratory infections	All Ages	Both	1 Cigarette-Equivalents	1·21 (1·00–1·52)
Lower respiratory infections	All Ages	Both	5 Cigarette-Equivalents	1·58 (1·26–1·95)
Lower respiratory infections	All Ages	Both	10 Cigarette-Equivalents	1·96 (1·36–2·73)
Lower respiratory infections	All Ages	Both	20 Cigarette-Equivalents	2·47 (1·80–3·31)
Lower respiratory infections	All Ages	Both	30 Cigarette-Equivalents	3·08 (2·32–3·99)
Esophageal cancer	All Ages	Both	0 Pack-Years	1·00 (1·00–1·00)
Esophageal cancer	All Ages	Both	1 Pack-Years	1·14 (1·09–1·19)
Esophageal cancer	All Ages	Both	5 Pack-Years	1·68 (1·44–1·94)
Esophageal cancer	All Ages	Both	10 Pack-Years	2·36 (1·88–2·88)
Esophageal cancer	All Ages	Both	20 Pack-Years	2·95 (2·45–3·44)
Esophageal cancer	All Ages	Both	40 Pack-Years	3·91 (3·09–4·79)
Esophageal cancer	All Ages	Both	60 Pack-Years	5·33 (2·86–8·23)
Stomach cancer	All Ages	Both	0 Pack-Years	1·00 (1·00–1·00)
Stomach cancer	All Ages	Both	1 Pack-Years	1·03 (1·01–1·06)
Stomach cancer	All Ages	Both	5 Pack-Years	1·17 (1·07–1·29)
Stomach cancer	All Ages	Both	10 Pack-Years	1·34 (1·15–1·57)
Stomach cancer	All Ages	Both	20 Pack-Years	1·53 (1·32–1·78)

Stomach cancer	All Ages	Both	40 Pack-Years	1·94 (1·58–2·33)
Stomach cancer	All Ages	Both	60 Pack-Years	2·08 (1·74–2·44)
Liver cancer	All Ages	Both	0 Pack-Years	1·00 (1·00–1·00)
Liver cancer	All Ages	Both	1 Pack-Years	1·04 (1·00–1·07)
Liver cancer	All Ages	Both	5 Pack-Years	1·18 (1·01–1·36)
Liver cancer	All Ages	Both	10 Pack-Years	1·35 (1·03–1·71)
Liver cancer	All Ages	Both	20 Pack-Years	1·56 (1·20–1·97)
Liver cancer	All Ages	Both	40 Pack-Years	1·78 (1·27–2·38)
Liver cancer	All Ages	Both	60 Pack-Years	1·80 (1·19–2·67)
Larynx cancer	All Ages	Both	0 Pack-Years	1·00 (1·00–1·00)
Larynx cancer	All Ages	Both	1 Pack-Years	1·24 (1·12–1·39)
Larynx cancer	All Ages	Both	5 Pack-Years	2·20 (1·60–2·94)
Larynx cancer	All Ages	Both	10 Pack-Years	3·40 (2·19–4·88)
Larynx cancer	All Ages	Both	20 Pack-Years	5·88 (4·03–8·04)
Larynx cancer	All Ages	Both	40 Pack-Years	13·6 (8·67–20·4)
Larynx cancer	All Ages	Both	60 Pack-Years	21·9 (10·6–36·7)
Tracheal, bronchus, and lung cancer	All Ages	Both	0 Pack-Years	1·00 (1·00–1·00)
Tracheal, bronchus, and lung cancer	All Ages	Both	1 Pack-Years	1·15 (1·04–1·29)
Tracheal, bronchus, and lung cancer	All Ages	Both	5 Pack-Years	1·76 (1·21–2·45)
Tracheal, bronchus, and lung cancer	All Ages	Both	10 Pack-Years	3·43 (2·92–4·00)
Tracheal, bronchus, and lung cancer	All Ages	Both	20 Pack-Years	6·47 (5·78–7·26)
Tracheal, bronchus, and lung cancer	All Ages	Both	40 Pack-Years	12·6 (10·3–15·1)
Tracheal, bronchus, and lung cancer	All Ages	Both	60 Pack-Years	15·6 (12·4–19·2)
Breast cancer	All Ages	Male	0 Pack-Years	1·00 (1·00–1·00)
Breast cancer	All Ages	Male	1 Pack-Years	1·00 (1·00–1·00)
Breast cancer	All Ages	Male	5 Pack-Years	1·00 (1·00–1·00)
Breast cancer	All Ages	Male	10 Pack-Years	1·00 (1·00–1·00)

Breast cancer	All Ages	Male	20 Pack-Years	1·00 (1·00–1·00)
Breast cancer	All Ages	Male	40 Pack-Years	1·00 (1·00–1·00)
Breast cancer	All Ages	Male	60 Pack-Years	1·00 (1·00–1·00)
Breast cancer	All Ages	Female	0 Pack-Years	1·00 (1·00–1·00)
Breast cancer	All Ages	Female	1 Pack-Years	1·02 (1·01–1·03)
Breast cancer	All Ages	Female	5 Pack-Years	1·08 (1·04–1·13)
Breast cancer	All Ages	Female	10 Pack-Years	1·16 (1·07–1·25)
Breast cancer	All Ages	Female	20 Pack-Years	1·27 (1·19–1·35)
Breast cancer	All Ages	Female	40 Pack-Years	1·24 (1·11–1·38)
Breast cancer	All Ages	Female	60 Pack-Years	1·27 (1·11–1·45)
Cervical cancer	All Ages	Both	0 Pack-Years	1·00 (1·00–1·00)
Cervical cancer	All Ages	Both	1 Pack-Years	1·16 (1·02–1·35)
Cervical cancer	All Ages	Both	5 Pack-Years	1·79 (1·10–2·75)
Cervical cancer	All Ages	Both	10 Pack-Years	2·05 (1·31–3·13)
Cervical cancer	All Ages	Both	20 Pack-Years	3·04 (1·74–5·28)
Cervical cancer	All Ages	Both	40 Pack-Years	4·21 (1·74–8·53)
Cervical cancer	All Ages	Both	60 Pack-Years	4·21 (1·74–8·53)
Prostate cancer	All Ages	Male	0 Cigarette-Equivalents	1·00 (1·00–1·00)
Prostate cancer	All Ages	Male	1 Cigarette-Equivalents	1·03 (1·00–1·05)
Prostate cancer	All Ages	Male	5 Cigarette-Equivalents	1·13 (1·01–1·25)
Prostate cancer	All Ages	Male	10 Cigarette-Equivalents	1·19 (1·05–1·33)
Prostate cancer	All Ages	Male	20 Cigarette-Equivalents	1·17 (1·05–1·31)
Prostate cancer	All Ages	Male	30 Cigarette-Equivalents	1·24 (1·01–1·51)
Prostate cancer	All Ages	Female	0 Cigarette-Equivalents	1·00 (1·00–1·00)
Prostate cancer	All Ages	Female	1 Cigarette-Equivalents	1·00 (1·00–1·00)
Prostate cancer	All Ages	Female	5 Cigarette-Equivalents	1·00 (1·00–1·00)
Prostate cancer	All Ages	Female	10 Cigarette-Equivalents	1·00 (1·00–1·00)

Prostate cancer	All Ages	Female	20 Cigarette-Equivalents	1·00 (1·00–1·00)
Prostate cancer	All Ages	Female	30 Cigarette-Equivalents	1·00 (1·00–1·00)
Colon and rectum cancer	All Ages	Both	0 Pack-Years	1·00 (1·00–1·00)
Colon and rectum cancer	All Ages	Both	1 Pack-Years	1·03 (1·01–1·05)
Colon and rectum cancer	All Ages	Both	5 Pack-Years	1·13 (1·04–1·23)
Colon and rectum cancer	All Ages	Both	10 Pack-Years	1·27 (1·08–1·45)
Colon and rectum cancer	All Ages	Both	20 Pack-Years	1·51 (1·18–1·83)
Colon and rectum cancer	All Ages	Both	40 Pack-Years	1·60 (1·27–2·00)
Colon and rectum cancer	All Ages	Both	60 Pack-Years	1·58 (1·15–2·10)
Lip and oral cavity cancer	All Ages	Both	0 Pack-Years	1·00 (1·00–1·00)
Lip and oral cavity cancer	All Ages	Both	1 Pack-Years	1·12 (1·07–1·19)
Lip and oral cavity cancer	All Ages	Both	5 Pack-Years	1·62 (1·33–1·95)
Lip and oral cavity cancer	All Ages	Both	10 Pack-Years	2·24 (1·66–2·90)
Lip and oral cavity cancer	All Ages	Both	20 Pack-Years	3·26 (2·42–4·22)
Lip and oral cavity cancer	All Ages	Both	40 Pack-Years	3·82 (2·80–4·98)
Lip and oral cavity cancer	All Ages	Both	60 Pack-Years	4·60 (3·09–6·50)
Nasopharynx cancer	All Ages	Both	0 Pack-Years	1·00 (1·00–1·00)
Nasopharynx cancer	All Ages	Both	1 Pack-Years	1·08 (1·03–1·14)
Nasopharynx cancer	All Ages	Both	5 Pack-Years	1·41 (1·16–1·71)
Nasopharynx cancer	All Ages	Both	10 Pack-Years	1·82 (1·32–2·42)
Nasopharynx cancer	All Ages	Both	20 Pack-Years	1·98 (1·39–2·72)
Nasopharynx cancer	All Ages	Both	40 Pack-Years	2·56 (1·73–3·59)
Nasopharynx cancer	All Ages	Both	60 Pack-Years	4·03 (2·72–5·75)
Other pharynx cancer	All Ages	Both	0 Pack-Years	1·00 (1·00–1·00)
Other pharynx cancer	All Ages	Both	1 Pack-Years	1·21 (1·13–1·31)
Other pharynx cancer	All Ages	Both	5 Pack-Years	2·07 (1·64–2·55)
Other pharynx cancer	All Ages	Both	10 Pack-Years	3·14 (2·28–4·11)

Other pharynx cancer	All Ages	Both	20 Pack-Years	4·85 (3·59–6·36)
Other pharynx cancer	All Ages	Both	40 Pack-Years	5·60 (4·34–7·12)
Other pharynx cancer	All Ages	Both	60 Pack-Years	6·83 (5·28–8·92)
Pancreatic cancer	All Ages	Male	0 Pack-Years	1·00 (1·00–1·00)
Pancreatic cancer	All Ages	Male	1 Pack-Years	1·04 (1·02–1·06)
Pancreatic cancer	All Ages	Male	5 Pack-Years	1·21 (1·11–1·31)
Pancreatic cancer	All Ages	Male	10 Pack-Years	1·41 (1·21–1·62)
Pancreatic cancer	All Ages	Male	20 Pack-Years	1·69 (1·47–1·92)
Pancreatic cancer	All Ages	Male	40 Pack-Years	2·00 (1·75–2·26)
Pancreatic cancer	All Ages	Male	60 Pack-Years	2·38 (1·86–3·00)
Pancreatic cancer	All Ages	Female	0 Pack-Years	1·00 (1·00–1·00)
Pancreatic cancer	All Ages	Female	1 Pack-Years	1·09 (1·06–1·12)
Pancreatic cancer	All Ages	Female	5 Pack-Years	1·43 (1·29–1·58)
Pancreatic cancer	All Ages	Female	10 Pack-Years	1·86 (1·57–2·17)
Pancreatic cancer	All Ages	Female	20 Pack-Years	2·46 (2·09–2·87)
Pancreatic cancer	All Ages	Female	40 Pack-Years	3·03 (2·41–3·67)
Pancreatic cancer	All Ages	Female	60 Pack-Years	3·74 (2·26–5·61)
Kidney cancer	All Ages	Both	0 Pack-Years	1·00 (1·00–1·00)
Kidney cancer	All Ages	Both	1 Pack-Years	1·03 (1·00–1·06)
Kidney cancer	All Ages	Both	5 Pack-Years	1·15 (1·02–1·30)
Kidney cancer	All Ages	Both	10 Pack-Years	1·30 (1·04–1·61)
Kidney cancer	All Ages	Both	20 Pack-Years	1·59 (1·29–1·90)
Kidney cancer	All Ages	Both	40 Pack-Years	1·79 (1·39–2·27)
Kidney cancer	All Ages	Both	60 Pack-Years	1·99 (1·31–2·91)
Bladder cancer	All Ages	Both	0 Pack-Years	1·00 (1·00–1·00)
Bladder cancer	All Ages	Both	1 Pack-Years	1·12 (1·05–1·21)
Bladder cancer	All Ages	Both	5 Pack-Years	1·60 (1·23–2·05)

Bladder cancer	All Ages	Both	10 Pack-Years	2·20 (1·46–3·11)
Bladder cancer	All Ages	Both	20 Pack-Years	2·98 (1·96–3·98)
Bladder cancer	All Ages	Both	40 Pack-Years	3·94 (2·67–5·63)
Bladder cancer	All Ages	Both	60 Pack-Years	4·55 (2·54–7·64)
Leukemia	All Ages	Both	0 Pack-Years	1·00 (1·00–1·00)
Leukemia	All Ages	Both	1 Pack-Years	1·08 (1·00–1·21)
Leukemia	All Ages	Both	5 Pack-Years	1·41 (1·00–2·05)
Leukemia	All Ages	Both	10 Pack-Years	1·81 (1·00–3·10)
Leukemia	All Ages	Both	20 Pack-Years	2·16 (1·33–3·33)
Leukemia	All Ages	Both	40 Pack-Years	2·64 (1·48–4·39)
Leukemia	All Ages	Both	60 Pack-Years	2·64 (1·48–4·39)
Ischemic heart disease	30 to 34	Male	0 Cigarette-Equivalents	1·00 (1·00–1·00)
Ischemic heart disease	30 to 34	Male	1 Cigarette-Equivalents	1·18 (1·14–1·21)
Ischemic heart disease	30 to 34	Male	5 Cigarette-Equivalents	1·88 (1·70–2·06)
Ischemic heart disease	30 to 34	Male	10 Cigarette-Equivalents	2·75 (2·40–3·12)
Ischemic heart disease	30 to 34	Male	20 Cigarette-Equivalents	3·22 (2·84–3·61)
Ischemic heart disease	30 to 34	Male	30 Cigarette-Equivalents	3·94 (3·41–4·48)
Ischemic heart disease	30 to 34	Female	0 Cigarette-Equivalents	1·00 (1·00–1·00)
Ischemic heart disease	30 to 34	Female	1 Cigarette-Equivalents	1·25 (1·17–1·34)
Ischemic heart disease	30 to 34	Female	5 Cigarette-Equivalents	2·24 (1·87–2·70)
Ischemic heart disease	30 to 34	Female	10 Cigarette-Equivalents	3·48 (2·75–4·40)
Ischemic heart disease	30 to 34	Female	20 Cigarette-Equivalents	6·04 (4·84–7·37)
Ischemic heart disease	30 to 34	Female	30 Cigarette-Equivalents	8·72 (6·88–10·8)
Ischemic heart disease	35 to 39	Male	0 Cigarette-Equivalents	1·00 (1·00–1·00)
Ischemic heart disease	35 to 39	Male	1 Cigarette-Equivalents	1·18 (1·14–1·21)
Ischemic heart disease	35 to 39	Male	5 Cigarette-Equivalents	1·88 (1·70–2·06)
Ischemic heart disease	35 to 39	Male	10 Cigarette-Equivalents	2·75 (2·40–3·12)

Ischemic heart disease	35 to 39	Male	20 Cigarette-Equivalents	3·22 (2·84–3·61)
Ischemic heart disease	35 to 39	Male	30 Cigarette-Equivalents	3·94 (3·41–4·48)
Ischemic heart disease	35 to 39	Female	0 Cigarette-Equivalents	1·00 (1·00–1·00)
Ischemic heart disease	35 to 39	Female	1 Cigarette-Equivalents	1·25 (1·17–1·34)
Ischemic heart disease	35 to 39	Female	5 Cigarette-Equivalents	2·24 (1·87–2·70)
Ischemic heart disease	35 to 39	Female	10 Cigarette-Equivalents	3·48 (2·75–4·40)
Ischemic heart disease	35 to 39	Female	20 Cigarette-Equivalents	6·04 (4·84–7·37)
Ischemic heart disease	35 to 39	Female	30 Cigarette-Equivalents	8·72 (6·88–10·8)
Ischemic heart disease	40 to 44	Male	0 Cigarette-Equivalents	1·00 (1·00–1·00)
Ischemic heart disease	40 to 44	Male	1 Cigarette-Equivalents	1·18 (1·14–1·21)
Ischemic heart disease	40 to 44	Male	5 Cigarette-Equivalents	1·88 (1·70–2·06)
Ischemic heart disease	40 to 44	Male	10 Cigarette-Equivalents	2·75 (2·40–3·12)
Ischemic heart disease	40 to 44	Male	20 Cigarette-Equivalents	3·22 (2·84–3·61)
Ischemic heart disease	40 to 44	Male	30 Cigarette-Equivalents	3·94 (3·41–4·48)
Ischemic heart disease	40 to 44	Female	0 Cigarette-Equivalents	1·00 (1·00–1·00)
Ischemic heart disease	40 to 44	Female	1 Cigarette-Equivalents	1·25 (1·17–1·34)
Ischemic heart disease	40 to 44	Female	5 Cigarette-Equivalents	2·24 (1·87–2·70)
Ischemic heart disease	40 to 44	Female	10 Cigarette-Equivalents	3·48 (2·75–4·40)
Ischemic heart disease	40 to 44	Female	20 Cigarette-Equivalents	6·04 (4·84–7·37)
Ischemic heart disease	40 to 44	Female	30 Cigarette-Equivalents	8·72 (6·88–10·8)
Ischemic heart disease	45 to 49	Male	0 Cigarette-Equivalents	1·00 (1·00–1·00)
Ischemic heart disease	45 to 49	Male	1 Cigarette-Equivalents	1·14 (1·11–1·16)
Ischemic heart disease	45 to 49	Male	5 Cigarette-Equivalents	1·68 (1·55–1·81)
Ischemic heart disease	45 to 49	Male	10 Cigarette-Equivalents	2·36 (2·10–2·62)
Ischemic heart disease	45 to 49	Male	20 Cigarette-Equivalents	2·64 (2·38–2·94)
Ischemic heart disease	45 to 49	Male	30 Cigarette-Equivalents	3·15 (2·79–3·54)
Ischemic heart disease	45 to 49	Female	0 Cigarette-Equivalents	1·00 (1·00–1·00)

Ischemic heart disease	45 to 49	Female	1 Cigarette-Equivalents	1·19 (1·14–1·25)
Ischemic heart disease	45 to 49	Female	5 Cigarette-Equivalents	1·97 (1·71–2·26)
Ischemic heart disease	45 to 49	Female	10 Cigarette-Equivalents	2·95 (2·42–3·52)
Ischemic heart disease	45 to 49	Female	20 Cigarette-Equivalents	4·53 (3·78–5·30)
Ischemic heart disease	45 to 49	Female	30 Cigarette-Equivalents	6·17 (5·07–7·40)
Ischemic heart disease	50 to 54	Male	0 Cigarette-Equivalents	1·00 (1·00–1·00)
Ischemic heart disease	50 to 54	Male	1 Cigarette-Equivalents	1·14 (1·11–1·16)
Ischemic heart disease	50 to 54	Male	5 Cigarette-Equivalents	1·68 (1·55–1·81)
Ischemic heart disease	50 to 54	Male	10 Cigarette-Equivalents	2·36 (2·10–2·62)
Ischemic heart disease	50 to 54	Male	20 Cigarette-Equivalents	2·64 (2·38–2·94)
Ischemic heart disease	50 to 54	Male	30 Cigarette-Equivalents	3·15 (2·79–3·54)
Ischemic heart disease	50 to 54	Female	0 Cigarette-Equivalents	1·00 (1·00–1·00)
Ischemic heart disease	50 to 54	Female	1 Cigarette-Equivalents	1·19 (1·14–1·25)
Ischemic heart disease	50 to 54	Female	5 Cigarette-Equivalents	1·97 (1·71–2·26)
Ischemic heart disease	50 to 54	Female	10 Cigarette-Equivalents	2·95 (2·42–3·52)
Ischemic heart disease	50 to 54	Female	20 Cigarette-Equivalents	4·53 (3·78–5·30)
Ischemic heart disease	50 to 54	Female	30 Cigarette-Equivalents	6·17 (5·07–7·40)
Ischemic heart disease	55 to 59	Male	0 Cigarette-Equivalents	1·00 (1·00–1·00)
Ischemic heart disease	55 to 59	Male	1 Cigarette-Equivalents	1·10 (1·08–1·12)
Ischemic heart disease	55 to 59	Male	5 Cigarette-Equivalents	1·50 (1·40–1·60)
Ischemic heart disease	55 to 59	Male	10 Cigarette-Equivalents	2·00 (1·81–2·20)
Ischemic heart disease	55 to 59	Male	20 Cigarette-Equivalents	2·17 (1·99–2·36)
Ischemic heart disease	55 to 59	Male	30 Cigarette-Equivalents	2·48 (2·26–2·74)
Ischemic heart disease	55 to 59	Female	0 Cigarette-Equivalents	1·00 (1·00–1·00)
Ischemic heart disease	55 to 59	Female	1 Cigarette-Equivalents	1·14 (1·11–1·18)
Ischemic heart disease	55 to 59	Female	5 Cigarette-Equivalents	1·72 (1·54–1·90)
Ischemic heart disease	55 to 59	Female	10 Cigarette-Equivalents	2·44 (2·07–2·81)

Ischemic heart disease	55 to 59	Female	20 Cigarette-Equivalents	3·36 (2·95–3·80)
Ischemic heart disease	55 to 59	Female	30 Cigarette-Equivalents	4·31 (3·69–4·96)
Ischemic heart disease	60 to 64	Male	0 Cigarette-Equivalents	1·00 (1·00–1·00)
Ischemic heart disease	60 to 64	Male	1 Cigarette-Equivalents	1·10 (1·08–1·12)
Ischemic heart disease	60 to 64	Male	5 Cigarette-Equivalents	1·50 (1·40–1·60)
Ischemic heart disease	60 to 64	Male	10 Cigarette-Equivalents	2·00 (1·81–2·20)
Ischemic heart disease	60 to 64	Male	20 Cigarette-Equivalents	2·17 (1·99–2·36)
Ischemic heart disease	60 to 64	Male	30 Cigarette-Equivalents	2·48 (2·26–2·74)
Ischemic heart disease	60 to 64	Female	0 Cigarette-Equivalents	1·00 (1·00–1·00)
Ischemic heart disease	60 to 64	Female	1 Cigarette-Equivalents	1·14 (1·11–1·18)
Ischemic heart disease	60 to 64	Female	5 Cigarette-Equivalents	1·72 (1·54–1·90)
Ischemic heart disease	60 to 64	Female	10 Cigarette-Equivalents	2·44 (2·07–2·81)
Ischemic heart disease	60 to 64	Female	20 Cigarette-Equivalents	3·36 (2·95–3·80)
Ischemic heart disease	60 to 64	Female	30 Cigarette-Equivalents	4·31 (3·69–4·96)
Ischemic heart disease	65 to 69	Male	0 Cigarette-Equivalents	1·00 (1·00–1·00)
Ischemic heart disease	65 to 69	Male	1 Cigarette-Equivalents	1·07 (1·06–1·08)
Ischemic heart disease	65 to 69	Male	5 Cigarette-Equivalents	1·35 (1·29–1·41)
Ischemic heart disease	65 to 69	Male	10 Cigarette-Equivalents	1·69 (1·57–1·81)
Ischemic heart disease	65 to 69	Male	20 Cigarette-Equivalents	1·78 (1·66–1·91)
Ischemic heart disease	65 to 69	Male	30 Cigarette-Equivalents	1·96 (1·82–2·11)
Ischemic heart disease	65 to 69	Female	0 Cigarette-Equivalents	1·00 (1·00–1·00)
Ischemic heart disease	65 to 69	Female	1 Cigarette-Equivalents	1·10 (1·08–1·12)
Ischemic heart disease	65 to 69	Female	5 Cigarette-Equivalents	1·50 (1·40–1·61)
Ischemic heart disease	65 to 69	Female	10 Cigarette-Equivalents	2·01 (1·81–2·22)
Ischemic heart disease	65 to 69	Female	20 Cigarette-Equivalents	2·47 (2·24–2·71)
Ischemic heart disease	65 to 69	Female	30 Cigarette-Equivalents	3·02 (2·70–3·35)
Ischemic heart disease	70 to 74	Male	0 Cigarette-Equivalents	1·00 (1·00–1·00)

Ischemic heart disease	70 to 74	Male	1 Cigarette-Equivalents	1·07 (1·06–1·08)
Ischemic heart disease	70 to 74	Male	5 Cigarette-Equivalents	1·35 (1·29–1·41)
Ischemic heart disease	70 to 74	Male	10 Cigarette-Equivalents	1·69 (1·57–1·81)
Ischemic heart disease	70 to 74	Male	20 Cigarette-Equivalents	1·78 (1·66–1·91)
Ischemic heart disease	70 to 74	Male	30 Cigarette-Equivalents	1·96 (1·82–2·11)
Ischemic heart disease	70 to 74	Female	0 Cigarette-Equivalents	1·00 (1·00–1·00)
Ischemic heart disease	70 to 74	Female	1 Cigarette-Equivalents	1·10 (1·08–1·12)
Ischemic heart disease	70 to 74	Female	5 Cigarette-Equivalents	1·50 (1·40–1·61)
Ischemic heart disease	70 to 74	Female	10 Cigarette-Equivalents	2·01 (1·81–2·22)
Ischemic heart disease	70 to 74	Female	20 Cigarette-Equivalents	2·47 (2·24–2·71)
Ischemic heart disease	70 to 74	Female	30 Cigarette-Equivalents	3·02 (2·70–3·35)
Ischemic heart disease	75 to 79	Male	0 Cigarette-Equivalents	1·00 (1·00–1·00)
Ischemic heart disease	75 to 79	Male	1 Cigarette-Equivalents	1·04 (1·03–1·05)
Ischemic heart disease	75 to 79	Male	5 Cigarette-Equivalents	1·21 (1·17–1·25)
Ischemic heart disease	75 to 79	Male	10 Cigarette-Equivalents	1·41 (1·33–1·50)
Ischemic heart disease	75 to 79	Male	20 Cigarette-Equivalents	1·46 (1·38–1·53)
Ischemic heart disease	75 to 79	Male	30 Cigarette-Equivalents	1·55 (1·46–1·64)
Ischemic heart disease	75 to 79	Female	0 Cigarette-Equivalents	1·00 (1·00–1·00)
Ischemic heart disease	75 to 79	Female	1 Cigarette-Equivalents	1·06 (1·05–1·07)
Ischemic heart disease	75 to 79	Female	5 Cigarette-Equivalents	1·30 (1·25–1·35)
Ischemic heart disease	75 to 79	Female	10 Cigarette-Equivalents	1·61 (1·50–1·71)
Ischemic heart disease	75 to 79	Female	20 Cigarette-Equivalents	1·81 (1·71–1·93)
Ischemic heart disease	75 to 79	Female	30 Cigarette-Equivalents	2·07 (1·92–2·22)
Ischemic heart disease	80 to 84	Male	0 Cigarette-Equivalents	1·00 (1·00–1·00)
Ischemic heart disease	80 to 84	Male	1 Cigarette-Equivalents	1·04 (1·03–1·05)
Ischemic heart disease	80 to 84	Male	5 Cigarette-Equivalents	1·21 (1·17–1·25)
Ischemic heart disease	80 to 84	Male	10 Cigarette-Equivalents	1·41 (1·33–1·50)

Ischemic heart disease	80 to 84	Male	20 Cigarette-Equivalents	1·46 (1·38–1·53)
Ischemic heart disease	80 to 84	Male	30 Cigarette-Equivalents	1·55 (1·46–1·64)
Ischemic heart disease	80 to 84	Female	0 Cigarette-Equivalents	1·00 (1·00–1·00)
Ischemic heart disease	80 to 84	Female	1 Cigarette-Equivalents	1·06 (1·05–1·07)
Ischemic heart disease	80 to 84	Female	5 Cigarette-Equivalents	1·30 (1·25–1·35)
Ischemic heart disease	80 to 84	Female	10 Cigarette-Equivalents	1·61 (1·50–1·71)
Ischemic heart disease	80 to 84	Female	20 Cigarette-Equivalents	1·81 (1·71–1·93)
Ischemic heart disease	80 to 84	Female	30 Cigarette-Equivalents	2·07 (1·92–2·22)
Ischemic heart disease	85 to 89	Male	0 Cigarette-Equivalents	1·00 (1·00–1·00)
Ischemic heart disease	85 to 89	Male	1 Cigarette-Equivalents	1·02 (1·01–1·02)
Ischemic heart disease	85 to 89	Male	5 Cigarette-Equivalents	1·08 (1·06–1·11)
Ischemic heart disease	85 to 89	Male	10 Cigarette-Equivalents	1·17 (1·12–1·22)
Ischemic heart disease	85 to 89	Male	20 Cigarette-Equivalents	1·19 (1·15–1·23)
Ischemic heart disease	85 to 89	Male	30 Cigarette-Equivalents	1·22 (1·17–1·27)
Ischemic heart disease	85 to 89	Female	0 Cigarette-Equivalents	1·00 (1·00–1·00)
Ischemic heart disease	85 to 89	Female	1 Cigarette-Equivalents	1·02 (1·02–1·03)
Ischemic heart disease	85 to 89	Female	5 Cigarette-Equivalents	1·12 (1·10–1·15)
Ischemic heart disease	85 to 89	Female	10 Cigarette-Equivalents	1·25 (1·20–1·30)
Ischemic heart disease	85 to 89	Female	20 Cigarette-Equivalents	1·32 (1·28–1·37)
Ischemic heart disease	85 to 89	Female	30 Cigarette-Equivalents	1·41 (1·36–1·47)
Ischemic heart disease	90 to 94	Male	0 Cigarette-Equivalents	1·00 (1·00–1·00)
Ischemic heart disease	90 to 94	Male	1 Cigarette-Equivalents	1·02 (1·01–1·02)
Ischemic heart disease	90 to 94	Male	5 Cigarette-Equivalents	1·08 (1·06–1·11)
Ischemic heart disease	90 to 94	Male	10 Cigarette-Equivalents	1·17 (1·12–1·22)
Ischemic heart disease	90 to 94	Male	20 Cigarette-Equivalents	1·19 (1·15–1·23)
Ischemic heart disease	90 to 94	Male	30 Cigarette-Equivalents	1·22 (1·17–1·27)
Ischemic heart disease	90 to 94	Female	0 Cigarette-Equivalents	1·00 (1·00–1·00)

Ischemic heart disease	90 to 94	Female	1 Cigarette-Equivalents	1·02 (1·02–1·03)
Ischemic heart disease	90 to 94	Female	5 Cigarette-Equivalents	1·12 (1·10–1·15)
Ischemic heart disease	90 to 94	Female	10 Cigarette-Equivalents	1·25 (1·20–1·30)
Ischemic heart disease	90 to 94	Female	20 Cigarette-Equivalents	1·32 (1·28–1·37)
Ischemic heart disease	90 to 94	Female	30 Cigarette-Equivalents	1·41 (1·36–1·47)
Ischemic heart disease	95 plus	Male	0 Cigarette-Equivalents	1·00 (1·00–1·00)
Ischemic heart disease	95 plus	Male	1 Cigarette-Equivalents	1·00 (1·00–1·00)
Ischemic heart disease	95 plus	Male	5 Cigarette-Equivalents	1·00 (1·00–1·00)
Ischemic heart disease	95 plus	Male	10 Cigarette-Equivalents	1·00 (1·00–1·01)
Ischemic heart disease	95 plus	Male	20 Cigarette-Equivalents	1·00 (1·00–1·01)
Ischemic heart disease	95 plus	Male	30 Cigarette-Equivalents	1·00 (1·00–1·01)
Ischemic heart disease	95 plus	Female	0 Cigarette-Equivalents	1·00 (1·00–1·00)
Ischemic heart disease	95 plus	Female	1 Cigarette-Equivalents	1·00 (1·00–1·00)
Ischemic heart disease	95 plus	Female	5 Cigarette-Equivalents	1·00 (1·00–1·01)
Ischemic heart disease	95 plus	Female	10 Cigarette-Equivalents	1·00 (1·00–1·01)
Ischemic heart disease	95 plus	Female	20 Cigarette-Equivalents	1·00 (1·00–1·01)
Ischemic heart disease	95 plus	Female	30 Cigarette-Equivalents	1·00 (1·00–1·01)
Stroke	30 to 34	Male	0 Cigarette-Equivalents	1·00 (1·00–1·00)
Stroke	30 to 34	Male	1 Cigarette-Equivalents	1·14 (1·10–1·17)
Stroke	30 to 34	Male	5 Cigarette-Equivalents	1·68 (1·49–1·87)
Stroke	30 to 34	Male	10 Cigarette-Equivalents	2·36 (1·98–2·74)
Stroke	30 to 34	Male	20 Cigarette-Equivalents	2·32 (1·92–2·75)
Stroke	30 to 34	Male	30 Cigarette-Equivalents	3·18 (2·65–3·77)
Stroke	30 to 34	Female	0 Cigarette-Equivalents	1·00 (1·00–1·00)
Stroke	30 to 34	Female	1 Cigarette-Equivalents	1·13 (1·07–1·22)
Stroke	30 to 34	Female	5 Cigarette-Equivalents	1·67 (1·34–2·08)
Stroke	30 to 34	Female	10 Cigarette-Equivalents	2·33 (1·69–3·17)

Stroke	30 to 34	Female	20 Cigarette-Equivalents	3·74 (2·69–4·98)
Stroke	30 to 34	Female	30 Cigarette-Equivalents	5·49 (3·81–7·70)
Stroke	35 to 39	Male	0 Cigarette-Equivalents	1·00 (1·00–1·00)
Stroke	35 to 39	Male	1 Cigarette-Equivalents	1·14 (1·10–1·17)
Stroke	35 to 39	Male	5 Cigarette-Equivalents	1·68 (1·49–1·87)
Stroke	35 to 39	Male	10 Cigarette-Equivalents	2·36 (1·98–2·74)
Stroke	35 to 39	Male	20 Cigarette-Equivalents	2·32 (1·92–2·75)
Stroke	35 to 39	Male	30 Cigarette-Equivalents	3·18 (2·65–3·77)
Stroke	35 to 39	Female	0 Cigarette-Equivalents	1·00 (1·00–1·00)
Stroke	35 to 39	Female	1 Cigarette-Equivalents	1·13 (1·07–1·22)
Stroke	35 to 39	Female	5 Cigarette-Equivalents	1·67 (1·34–2·08)
Stroke	35 to 39	Female	10 Cigarette-Equivalents	2·33 (1·69–3·17)
Stroke	35 to 39	Female	20 Cigarette-Equivalents	3·74 (2·69–4·98)
Stroke	35 to 39	Female	30 Cigarette-Equivalents	5·49 (3·81–7·70)
Stroke	40 to 44	Male	0 Cigarette-Equivalents	1·00 (1·00–1·00)
Stroke	40 to 44	Male	1 Cigarette-Equivalents	1·14 (1·10–1·17)
Stroke	40 to 44	Male	5 Cigarette-Equivalents	1·68 (1·49–1·87)
Stroke	40 to 44	Male	10 Cigarette-Equivalents	2·36 (1·98–2·74)
Stroke	40 to 44	Male	20 Cigarette-Equivalents	2·32 (1·92–2·75)
Stroke	40 to 44	Male	30 Cigarette-Equivalents	3·18 (2·65–3·77)
Stroke	40 to 44	Female	0 Cigarette-Equivalents	1·00 (1·00–1·00)
Stroke	40 to 44	Female	1 Cigarette-Equivalents	1·13 (1·07–1·22)
Stroke	40 to 44	Female	5 Cigarette-Equivalents	1·67 (1·34–2·08)
Stroke	40 to 44	Female	10 Cigarette-Equivalents	2·33 (1·69–3·17)
Stroke	40 to 44	Female	20 Cigarette-Equivalents	3·74 (2·69–4·98)
Stroke	40 to 44	Female	30 Cigarette-Equivalents	5·49 (3·81–7·70)
Stroke	45 to 49	Male	0 Cigarette-Equivalents	1·00 (1·00–1·00)

Stroke	45 to 49	Male	1 Cigarette-Equivalents	1·11 (1·08–1·14)
Stroke	45 to 49	Male	5 Cigarette-Equivalents	1·56 (1·42–1·70)
Stroke	45 to 49	Male	10 Cigarette-Equivalents	2·11 (1·84–2·40)
Stroke	45 to 49	Male	20 Cigarette-Equivalents	1·97 (1·70–2·28)
Stroke	45 to 49	Male	30 Cigarette-Equivalents	2·67 (2·26–3·07)
Stroke	45 to 49	Female	0 Cigarette-Equivalents	1·00 (1·00–1·00)
Stroke	45 to 49	Female	1 Cigarette-Equivalents	1·11 (1·06–1·17)
Stroke	45 to 49	Female	5 Cigarette-Equivalents	1·55 (1·30–1·86)
Stroke	45 to 49	Female	10 Cigarette-Equivalents	2·10 (1·60–2·72)
Stroke	45 to 49	Female	20 Cigarette-Equivalents	3·04 (2·30–3·94)
Stroke	45 to 49	Female	30 Cigarette-Equivalents	4·21 (2·99–5·64)
Stroke	50 to 54	Male	0 Cigarette-Equivalents	1·00 (1·00–1·00)
Stroke	50 to 54	Male	1 Cigarette-Equivalents	1·11 (1·08–1·14)
Stroke	50 to 54	Male	5 Cigarette-Equivalents	1·56 (1·42–1·70)
Stroke	50 to 54	Male	10 Cigarette-Equivalents	2·11 (1·84–2·40)
Stroke	50 to 54	Male	20 Cigarette-Equivalents	1·97 (1·70–2·28)
Stroke	50 to 54	Male	30 Cigarette-Equivalents	2·67 (2·26–3·07)
Stroke	50 to 54	Female	0 Cigarette-Equivalents	1·00 (1·00–1·00)
Stroke	50 to 54	Female	1 Cigarette-Equivalents	1·11 (1·06–1·17)
Stroke	50 to 54	Female	5 Cigarette-Equivalents	1·55 (1·30–1·86)
Stroke	50 to 54	Female	10 Cigarette-Equivalents	2·10 (1·60–2·72)
Stroke	50 to 54	Female	20 Cigarette-Equivalents	3·04 (2·30–3·94)
Stroke	50 to 54	Female	30 Cigarette-Equivalents	4·21 (2·99–5·64)
Stroke	55 to 59	Male	0 Cigarette-Equivalents	1·00 (1·00–1·00)
Stroke	55 to 59	Male	1 Cigarette-Equivalents	1·08 (1·06–1·11)
Stroke	55 to 59	Male	5 Cigarette-Equivalents	1·42 (1·32–1·53)
Stroke	55 to 59	Male	10 Cigarette-Equivalents	1·84 (1·64–2·06)

Stroke	55 to 59	Male	20 Cigarette-Equivalents	1·66 (1·45–1·88)
Stroke	55 to 59	Male	30 Cigarette-Equivalents	2·22 (1·94–2·52)
Stroke	55 to 59	Female	0 Cigarette-Equivalents	1·00 (1·00–1·00)
Stroke	55 to 59	Female	1 Cigarette-Equivalents	1·09 (1·05–1·13)
Stroke	55 to 59	Female	5 Cigarette-Equivalents	1·46 (1·25–1·67)
Stroke	55 to 59	Female	10 Cigarette-Equivalents	1·92 (1·51–2·33)
Stroke	55 to 59	Female	20 Cigarette-Equivalents	2·44 (1·88–3·06)
Stroke	55 to 59	Female	30 Cigarette-Equivalents	3·14 (2·33–4·02)
Stroke	60 to 64	Male	0 Cigarette-Equivalents	1·00 (1·00–1·00)
Stroke	60 to 64	Male	1 Cigarette-Equivalents	1·08 (1·06–1·11)
Stroke	60 to 64	Male	5 Cigarette-Equivalents	1·42 (1·32–1·53)
Stroke	60 to 64	Male	10 Cigarette-Equivalents	1·84 (1·64–2·06)
Stroke	60 to 64	Male	20 Cigarette-Equivalents	1·66 (1·45–1·88)
Stroke	60 to 64	Male	30 Cigarette-Equivalents	2·22 (1·94–2·52)
Stroke	60 to 64	Female	0 Cigarette-Equivalents	1·00 (1·00–1·00)
Stroke	60 to 64	Female	1 Cigarette-Equivalents	1·09 (1·05–1·13)
Stroke	60 to 64	Female	5 Cigarette-Equivalents	1·46 (1·25–1·67)
Stroke	60 to 64	Female	10 Cigarette-Equivalents	1·92 (1·51–2·33)
Stroke	60 to 64	Female	20 Cigarette-Equivalents	2·44 (1·88–3·06)
Stroke	60 to 64	Female	30 Cigarette-Equivalents	3·14 (2·33–4·02)
Stroke	65 to 69	Male	0 Cigarette-Equivalents	1·00 (1·00–1·00)
Stroke	65 to 69	Male	1 Cigarette-Equivalents	1·06 (1·05–1·08)
Stroke	65 to 69	Male	5 Cigarette-Equivalents	1·30 (1·23–1·38)
Stroke	65 to 69	Male	10 Cigarette-Equivalents	1·61 (1·46–1·76)
Stroke	65 to 69	Male	20 Cigarette-Equivalents	1·42 (1·28–1·56)
Stroke	65 to 69	Male	30 Cigarette-Equivalents	1·85 (1·66–2·04)
Stroke	65 to 69	Female	0 Cigarette-Equivalents	1·00 (1·00–1·00)

Stroke	65 to 69	Female	1 Cigarette-Equivalents	1·07 (1·04–1·10)
Stroke	65 to 69	Female	5 Cigarette-Equivalents	1·36 (1·22–1·51)
Stroke	65 to 69	Female	10 Cigarette-Equivalents	1·72 (1·44–2·01)
Stroke	65 to 69	Female	20 Cigarette-Equivalents	1·94 (1·60–2·28)
Stroke	65 to 69	Female	30 Cigarette-Equivalents	2·30 (1·87–2·82)
Stroke	70 to 74	Male	0 Cigarette-Equivalents	1·00 (1·00–1·00)
Stroke	70 to 74	Male	1 Cigarette-Equivalents	1·06 (1·05–1·08)
Stroke	70 to 74	Male	5 Cigarette-Equivalents	1·30 (1·23–1·38)
Stroke	70 to 74	Male	10 Cigarette-Equivalents	1·61 (1·46–1·76)
Stroke	70 to 74	Male	20 Cigarette-Equivalents	1·42 (1·28–1·56)
Stroke	70 to 74	Male	30 Cigarette-Equivalents	1·85 (1·66–2·04)
Stroke	70 to 74	Female	0 Cigarette-Equivalents	1·00 (1·00–1·00)
Stroke	70 to 74	Female	1 Cigarette-Equivalents	1·07 (1·04–1·10)
Stroke	70 to 74	Female	5 Cigarette-Equivalents	1·36 (1·22–1·51)
Stroke	70 to 74	Female	10 Cigarette-Equivalents	1·72 (1·44–2·01)
Stroke	70 to 74	Female	20 Cigarette-Equivalents	1·94 (1·60–2·28)
Stroke	70 to 74	Female	30 Cigarette-Equivalents	2·30 (1·87–2·82)
Stroke	75 to 79	Male	0 Cigarette-Equivalents	1·00 (1·00–1·00)
Stroke	75 to 79	Male	1 Cigarette-Equivalents	1·04 (1·03–1·05)
Stroke	75 to 79	Male	5 Cigarette-Equivalents	1·19 (1·14–1·24)
Stroke	75 to 79	Male	10 Cigarette-Equivalents	1·39 (1·29–1·48)
Stroke	75 to 79	Male	20 Cigarette-Equivalents	1·22 (1·14–1·31)
Stroke	75 to 79	Male	30 Cigarette-Equivalents	1·52 (1·40–1·64)
Stroke	75 to 79	Female	0 Cigarette-Equivalents	1·00 (1·00–1·00)
Stroke	75 to 79	Female	1 Cigarette-Equivalents	1·05 (1·03–1·06)
Stroke	75 to 79	Female	5 Cigarette-Equivalents	1·24 (1·16–1·32)
Stroke	75 to 79	Female	10 Cigarette-Equivalents	1·48 (1·32–1·64)

Stroke	75 to 79	Female	20 Cigarette-Equivalents	1·53 (1·33–1·72)
Stroke	75 to 79	Female	30 Cigarette-Equivalents	1·72 (1·47–2·01)
Stroke	80 to 84	Male	0 Cigarette-Equivalents	1·00 (1·00–1·00)
Stroke	80 to 84	Male	1 Cigarette-Equivalents	1·04 (1·03–1·05)
Stroke	80 to 84	Male	5 Cigarette-Equivalents	1·19 (1·14–1·24)
Stroke	80 to 84	Male	10 Cigarette-Equivalents	1·39 (1·29–1·48)
Stroke	80 to 84	Male	20 Cigarette-Equivalents	1·22 (1·14–1·31)
Stroke	80 to 84	Male	30 Cigarette-Equivalents	1·52 (1·40–1·64)
Stroke	80 to 84	Female	0 Cigarette-Equivalents	1·00 (1·00–1·00)
Stroke	80 to 84	Female	1 Cigarette-Equivalents	1·05 (1·03–1·06)
Stroke	80 to 84	Female	5 Cigarette-Equivalents	1·24 (1·16–1·32)
Stroke	80 to 84	Female	10 Cigarette-Equivalents	1·48 (1·32–1·64)
Stroke	80 to 84	Female	20 Cigarette-Equivalents	1·53 (1·33–1·72)
Stroke	80 to 84	Female	30 Cigarette-Equivalents	1·72 (1·47–2·01)
Stroke	85 to 89	Male	0 Cigarette-Equivalents	1·00 (1·00–1·00)
Stroke	85 to 89	Male	1 Cigarette-Equivalents	1·02 (1·01–1·02)
Stroke	85 to 89	Male	5 Cigarette-Equivalents	1·08 (1·05–1·10)
Stroke	85 to 89	Male	10 Cigarette-Equivalents	1·15 (1·10–1·20)
Stroke	85 to 89	Male	20 Cigarette-Equivalents	1·08 (1·04–1·12)
Stroke	85 to 89	Male	30 Cigarette-Equivalents	1·21 (1·15–1·26)
Stroke	85 to 89	Female	0 Cigarette-Equivalents	1·00 (1·00–1·00)
Stroke	85 to 89	Female	1 Cigarette-Equivalents	1·02 (1·01–1·03)
Stroke	85 to 89	Female	5 Cigarette-Equivalents	1·10 (1·06–1·15)
Stroke	85 to 89	Female	10 Cigarette-Equivalents	1·20 (1·12–1·30)
Stroke	85 to 89	Female	20 Cigarette-Equivalents	1·19 (1·13–1·27)
Stroke	85 to 89	Female	30 Cigarette-Equivalents	1·29 (1·17–1·41)
Stroke	90 to 94	Male	0 Cigarette-Equivalents	1·00 (1·00–1·00)

Stroke	90 to 94	Male	1 Cigarette-Equivalents	1·02 (1·01–1·02)
Stroke	90 to 94	Male	5 Cigarette-Equivalents	1·08 (1·05–1·10)
Stroke	90 to 94	Male	10 Cigarette-Equivalents	1·15 (1·10–1·20)
Stroke	90 to 94	Male	20 Cigarette-Equivalents	1·08 (1·04–1·12)
Stroke	90 to 94	Male	30 Cigarette-Equivalents	1·21 (1·15–1·26)
Stroke	90 to 94	Female	0 Cigarette-Equivalents	1·00 (1·00–1·00)
Stroke	90 to 94	Female	1 Cigarette-Equivalents	1·02 (1·01–1·03)
Stroke	90 to 94	Female	5 Cigarette-Equivalents	1·10 (1·06–1·15)
Stroke	90 to 94	Female	10 Cigarette-Equivalents	1·20 (1·12–1·30)
Stroke	90 to 94	Female	20 Cigarette-Equivalents	1·19 (1·13–1·27)
Stroke	90 to 94	Female	30 Cigarette-Equivalents	1·29 (1·17–1·41)
Stroke	95 plus	Male	0 Cigarette-Equivalents	1·00 (1·00–1·00)
Stroke	95 plus	Male	1 Cigarette-Equivalents	1·00 (1·00–1·00)
Stroke	95 plus	Male	5 Cigarette-Equivalents	1·01 (1·00–1·02)
Stroke	95 plus	Male	10 Cigarette-Equivalents	1·01 (1·00–1·04)
Stroke	95 plus	Male	20 Cigarette-Equivalents	1·00 (1·00–1·02)
Stroke	95 plus	Male	30 Cigarette-Equivalents	1·01 (1·00–1·03)
Stroke	95 plus	Female	0 Cigarette-Equivalents	1·00 (1·00–1·00)
Stroke	95 plus	Female	1 Cigarette-Equivalents	1·00 (1·00–1·00)
Stroke	95 plus	Female	5 Cigarette-Equivalents	1·00 (1·00–1·01)
Stroke	95 plus	Female	10 Cigarette-Equivalents	1·01 (1·00–1·03)
Stroke	95 plus	Female	20 Cigarette-Equivalents	1·00 (1·00–1·01)
Stroke	95 plus	Female	30 Cigarette-Equivalents	1·01 (1·00–1·02)
Atrial fibrillation and flutter	30 to 34	Male	0 Cigarette-Equivalents	1·00 (1·00–1·00)
Atrial fibrillation and flutter	30 to 34	Male	1 Cigarette-Equivalents	1·08 (1·01–1·18)
Atrial fibrillation and flutter	30 to 34	Male	5 Cigarette-Equivalents	1·42 (1·03–1·91)
Atrial fibrillation and flutter	30 to 34	Male	10 Cigarette-Equivalents	1·75 (1·22–2·42)

Atrial fibrillation and flutter	30 to 34	Male	20 Cigarette-Equivalents	2·29 (1·49–3·40)
Atrial fibrillation and flutter	30 to 34	Male	30 Cigarette-Equivalents	2·59 (1·54–4·08)
Atrial fibrillation and flutter	30 to 34	Female	0 Cigarette-Equivalents	1·00 (1·00–1·00)
Atrial fibrillation and flutter	30 to 34	Female	1 Cigarette-Equivalents	1·08 (1·01–1·18)
Atrial fibrillation and flutter	30 to 34	Female	5 Cigarette-Equivalents	1·42 (1·03–1·91)
Atrial fibrillation and flutter	30 to 34	Female	10 Cigarette-Equivalents	1·75 (1·22–2·42)
Atrial fibrillation and flutter	30 to 34	Female	20 Cigarette-Equivalents	2·29 (1·49–3·40)
Atrial fibrillation and flutter	30 to 34	Female	30 Cigarette-Equivalents	2·59 (1·54–4·08)
Atrial fibrillation and flutter	35 to 39	Male	0 Cigarette-Equivalents	1·00 (1·00–1·00)
Atrial fibrillation and flutter	35 to 39	Male	1 Cigarette-Equivalents	1·08 (1·01–1·18)
Atrial fibrillation and flutter	35 to 39	Male	5 Cigarette-Equivalents	1·42 (1·03–1·91)
Atrial fibrillation and flutter	35 to 39	Male	10 Cigarette-Equivalents	1·75 (1·22–2·42)
Atrial fibrillation and flutter	35 to 39	Male	20 Cigarette-Equivalents	2·29 (1·49–3·40)
Atrial fibrillation and flutter	35 to 39	Male	30 Cigarette-Equivalents	2·59 (1·54–4·08)
Atrial fibrillation and flutter	35 to 39	Female	0 Cigarette-Equivalents	1·00 (1·00–1·00)
Atrial fibrillation and flutter	35 to 39	Female	1 Cigarette-Equivalents	1·08 (1·01–1·18)
Atrial fibrillation and flutter	35 to 39	Female	5 Cigarette-Equivalents	1·42 (1·03–1·91)
Atrial fibrillation and flutter	35 to 39	Female	10 Cigarette-Equivalents	1·75 (1·22–2·42)
Atrial fibrillation and flutter	35 to 39	Female	20 Cigarette-Equivalents	2·29 (1·49–3·40)
Atrial fibrillation and flutter	35 to 39	Female	30 Cigarette-Equivalents	2·59 (1·54–4·08)
Atrial fibrillation and flutter	40 to 44	Male	0 Cigarette-Equivalents	1·00 (1·00–1·00)
Atrial fibrillation and flutter	40 to 44	Male	1 Cigarette-Equivalents	1·08 (1·01–1·18)
Atrial fibrillation and flutter	40 to 44	Male	5 Cigarette-Equivalents	1·42 (1·03–1·91)
Atrial fibrillation and flutter	40 to 44	Male	10 Cigarette-Equivalents	1·75 (1·22–2·42)
Atrial fibrillation and flutter	40 to 44	Male	20 Cigarette-Equivalents	2·29 (1·49–3·40)
Atrial fibrillation and flutter	40 to 44	Male	30 Cigarette-Equivalents	2·59 (1·54–4·08)
Atrial fibrillation and flutter	40 to 44	Female	0 Cigarette-Equivalents	1·00 (1·00–1·00)

Atrial fibrillation and flutter	40 to 44	Female	1 Cigarette-Equivalents	1·08 (1·01–1·18)
Atrial fibrillation and flutter	40 to 44	Female	5 Cigarette-Equivalents	1·42 (1·03–1·91)
Atrial fibrillation and flutter	40 to 44	Female	10 Cigarette-Equivalents	1·75 (1·22–2·42)
Atrial fibrillation and flutter	40 to 44	Female	20 Cigarette-Equivalents	2·29 (1·49–3·40)
Atrial fibrillation and flutter	40 to 44	Female	30 Cigarette-Equivalents	2·59 (1·54–4·08)
Atrial fibrillation and flutter	45 to 49	Male	0 Cigarette-Equivalents	1·00 (1·00–1·00)
Atrial fibrillation and flutter	45 to 49	Male	1 Cigarette-Equivalents	1·08 (1·01–1·17)
Atrial fibrillation and flutter	45 to 49	Male	5 Cigarette-Equivalents	1·40 (1·04–1·83)
Atrial fibrillation and flutter	45 to 49	Male	10 Cigarette-Equivalents	1·66 (1·19–2·22)
Atrial fibrillation and flutter	45 to 49	Male	20 Cigarette-Equivalents	2·04 (1·42–2·93)
Atrial fibrillation and flutter	45 to 49	Male	30 Cigarette-Equivalents	2·26 (1·45–3·40)
Atrial fibrillation and flutter	45 to 49	Female	0 Cigarette-Equivalents	1·00 (1·00–1·00)
Atrial fibrillation and flutter	45 to 49	Female	1 Cigarette-Equivalents	1·08 (1·01–1·17)
Atrial fibrillation and flutter	45 to 49	Female	5 Cigarette-Equivalents	1·40 (1·04–1·83)
Atrial fibrillation and flutter	45 to 49	Female	10 Cigarette-Equivalents	1·66 (1·19–2·22)
Atrial fibrillation and flutter	45 to 49	Female	20 Cigarette-Equivalents	2·04 (1·42–2·93)
Atrial fibrillation and flutter	45 to 49	Female	30 Cigarette-Equivalents	2·26 (1·45–3·40)
Atrial fibrillation and flutter	50 to 54	Male	0 Cigarette-Equivalents	1·00 (1·00–1·00)
Atrial fibrillation and flutter	50 to 54	Male	1 Cigarette-Equivalents	1·08 (1·01–1·17)
Atrial fibrillation and flutter	50 to 54	Male	5 Cigarette-Equivalents	1·40 (1·04–1·83)
Atrial fibrillation and flutter	50 to 54	Male	10 Cigarette-Equivalents	1·66 (1·19–2·22)
Atrial fibrillation and flutter	50 to 54	Male	20 Cigarette-Equivalents	2·04 (1·42–2·93)
Atrial fibrillation and flutter	50 to 54	Male	30 Cigarette-Equivalents	2·26 (1·45–3·40)
Atrial fibrillation and flutter	50 to 54	Female	0 Cigarette-Equivalents	1·00 (1·00–1·00)
Atrial fibrillation and flutter	50 to 54	Female	1 Cigarette-Equivalents	1·08 (1·01–1·17)
Atrial fibrillation and flutter	50 to 54	Female	5 Cigarette-Equivalents	1·40 (1·04–1·83)
Atrial fibrillation and flutter	50 to 54	Female	10 Cigarette-Equivalents	1·66 (1·19–2·22)

Atrial fibrillation and flutter	50 to 54	Female	20 Cigarette-Equivalents	2·04 (1·42–2·93)
Atrial fibrillation and flutter	50 to 54	Female	30 Cigarette-Equivalents	2·26 (1·45–3·40)
Atrial fibrillation and flutter	55 to 59	Male	0 Cigarette-Equivalents	1·00 (1·00–1·00)
Atrial fibrillation and flutter	55 to 59	Male	1 Cigarette-Equivalents	1·08 (1·01–1·15)
Atrial fibrillation and flutter	55 to 59	Male	5 Cigarette-Equivalents	1·38 (1·03–1·74)
Atrial fibrillation and flutter	55 to 59	Male	10 Cigarette-Equivalents	1·57 (1·16–2·00)
Atrial fibrillation and flutter	55 to 59	Male	20 Cigarette-Equivalents	1·80 (1·30–2·48)
Atrial fibrillation and flutter	55 to 59	Male	30 Cigarette-Equivalents	1·95 (1·35–2·87)
Atrial fibrillation and flutter	55 to 59	Female	0 Cigarette-Equivalents	1·00 (1·00–1·00)
Atrial fibrillation and flutter	55 to 59	Female	1 Cigarette-Equivalents	1·08 (1·01–1·15)
Atrial fibrillation and flutter	55 to 59	Female	5 Cigarette-Equivalents	1·38 (1·03–1·74)
Atrial fibrillation and flutter	55 to 59	Female	10 Cigarette-Equivalents	1·57 (1·16–2·00)
Atrial fibrillation and flutter	55 to 59	Female	20 Cigarette-Equivalents	1·80 (1·30–2·48)
Atrial fibrillation and flutter	55 to 59	Female	30 Cigarette-Equivalents	1·95 (1·35–2·87)
Atrial fibrillation and flutter	60 to 64	Male	0 Cigarette-Equivalents	1·00 (1·00–1·00)
Atrial fibrillation and flutter	60 to 64	Male	1 Cigarette-Equivalents	1·08 (1·01–1·15)
Atrial fibrillation and flutter	60 to 64	Male	5 Cigarette-Equivalents	1·38 (1·03–1·74)
Atrial fibrillation and flutter	60 to 64	Male	10 Cigarette-Equivalents	1·57 (1·16–2·00)
Atrial fibrillation and flutter	60 to 64	Male	20 Cigarette-Equivalents	1·80 (1·30–2·48)
Atrial fibrillation and flutter	60 to 64	Male	30 Cigarette-Equivalents	1·95 (1·35–2·87)
Atrial fibrillation and flutter	60 to 64	Female	0 Cigarette-Equivalents	1·00 (1·00–1·00)
Atrial fibrillation and flutter	60 to 64	Female	1 Cigarette-Equivalents	1·08 (1·01–1·15)
Atrial fibrillation and flutter	60 to 64	Female	5 Cigarette-Equivalents	1·38 (1·03–1·74)
Atrial fibrillation and flutter	60 to 64	Female	10 Cigarette-Equivalents	1·57 (1·16–2·00)
Atrial fibrillation and flutter	60 to 64	Female	20 Cigarette-Equivalents	1·80 (1·30–2·48)
Atrial fibrillation and flutter	60 to 64	Female	30 Cigarette-Equivalents	1·95 (1·35–2·87)
Atrial fibrillation and flutter	65 to 69	Male	0 Cigarette-Equivalents	1·00 (1·00–1·00)

Atrial fibrillation and flutter	65 to 69	Male	1 Cigarette-Equivalents	1·07 (1·01–1·12)
Atrial fibrillation and flutter	65 to 69	Male	5 Cigarette-Equivalents	1·34 (1·03–1·62)
Atrial fibrillation and flutter	65 to 69	Male	10 Cigarette-Equivalents	1·45 (1·14–1·76)
Atrial fibrillation and flutter	65 to 69	Male	20 Cigarette-Equivalents	1·54 (1·20–2·05)
Atrial fibrillation and flutter	65 to 69	Male	30 Cigarette-Equivalents	1·63 (1·23–2·19)
Atrial fibrillation and flutter	65 to 69	Female	0 Cigarette-Equivalents	1·00 (1·00–1·00)
Atrial fibrillation and flutter	65 to 69	Female	1 Cigarette-Equivalents	1·07 (1·01–1·12)
Atrial fibrillation and flutter	65 to 69	Female	5 Cigarette-Equivalents	1·34 (1·03–1·62)
Atrial fibrillation and flutter	65 to 69	Female	10 Cigarette-Equivalents	1·45 (1·14–1·76)
Atrial fibrillation and flutter	65 to 69	Female	20 Cigarette-Equivalents	1·54 (1·20–2·05)
Atrial fibrillation and flutter	65 to 69	Female	30 Cigarette-Equivalents	1·63 (1·23–2·19)
Atrial fibrillation and flutter	70 to 74	Male	0 Cigarette-Equivalents	1·00 (1·00–1·00)
Atrial fibrillation and flutter	70 to 74	Male	1 Cigarette-Equivalents	1·07 (1·01–1·12)
Atrial fibrillation and flutter	70 to 74	Male	5 Cigarette-Equivalents	1·34 (1·03–1·62)
Atrial fibrillation and flutter	70 to 74	Male	10 Cigarette-Equivalents	1·45 (1·14–1·76)
Atrial fibrillation and flutter	70 to 74	Male	20 Cigarette-Equivalents	1·54 (1·20–2·05)
Atrial fibrillation and flutter	70 to 74	Male	30 Cigarette-Equivalents	1·63 (1·23–2·19)
Atrial fibrillation and flutter	70 to 74	Female	0 Cigarette-Equivalents	1·00 (1·00–1·00)
Atrial fibrillation and flutter	70 to 74	Female	1 Cigarette-Equivalents	1·07 (1·01–1·12)
Atrial fibrillation and flutter	70 to 74	Female	5 Cigarette-Equivalents	1·34 (1·03–1·62)
Atrial fibrillation and flutter	70 to 74	Female	10 Cigarette-Equivalents	1·45 (1·14–1·76)
Atrial fibrillation and flutter	70 to 74	Female	20 Cigarette-Equivalents	1·54 (1·20–2·05)
Atrial fibrillation and flutter	70 to 74	Female	30 Cigarette-Equivalents	1·63 (1·23–2·19)
Atrial fibrillation and flutter	75 to 79	Male	0 Cigarette-Equivalents	1·00 (1·00–1·00)
Atrial fibrillation and flutter	75 to 79	Male	1 Cigarette-Equivalents	1·05 (1·01–1·09)
Atrial fibrillation and flutter	75 to 79	Male	5 Cigarette-Equivalents	1·26 (1·04–1·47)
Atrial fibrillation and flutter	75 to 79	Male	10 Cigarette-Equivalents	1·30 (1·10–1·51)

Atrial fibrillation and flutter	75 to 79	Male	20 Cigarette-Equivalents	1·31 (1·07–1·66)
Atrial fibrillation and flutter	75 to 79	Male	30 Cigarette-Equivalents	1·37 (1·10–1·74)
Atrial fibrillation and flutter	75 to 79	Female	0 Cigarette-Equivalents	1·00 (1·00–1·00)
Atrial fibrillation and flutter	75 to 79	Female	1 Cigarette-Equivalents	1·05 (1·01–1·09)
Atrial fibrillation and flutter	75 to 79	Female	5 Cigarette-Equivalents	1·26 (1·04–1·47)
Atrial fibrillation and flutter	75 to 79	Female	10 Cigarette-Equivalents	1·30 (1·10–1·51)
Atrial fibrillation and flutter	75 to 79	Female	20 Cigarette-Equivalents	1·31 (1·07–1·66)
Atrial fibrillation and flutter	75 to 79	Female	30 Cigarette-Equivalents	1·37 (1·10–1·74)
Atrial fibrillation and flutter	80 to 84	Male	0 Cigarette-Equivalents	1·00 (1·00–1·00)
Atrial fibrillation and flutter	80 to 84	Male	1 Cigarette-Equivalents	1·05 (1·01–1·09)
Atrial fibrillation and flutter	80 to 84	Male	5 Cigarette-Equivalents	1·26 (1·04–1·47)
Atrial fibrillation and flutter	80 to 84	Male	10 Cigarette-Equivalents	1·30 (1·10–1·51)
Atrial fibrillation and flutter	80 to 84	Male	20 Cigarette-Equivalents	1·31 (1·07–1·66)
Atrial fibrillation and flutter	80 to 84	Male	30 Cigarette-Equivalents	1·37 (1·10–1·74)
Atrial fibrillation and flutter	80 to 84	Female	0 Cigarette-Equivalents	1·00 (1·00–1·00)
Atrial fibrillation and flutter	80 to 84	Female	1 Cigarette-Equivalents	1·05 (1·01–1·09)
Atrial fibrillation and flutter	80 to 84	Female	5 Cigarette-Equivalents	1·26 (1·04–1·47)
Atrial fibrillation and flutter	80 to 84	Female	10 Cigarette-Equivalents	1·30 (1·10–1·51)
Atrial fibrillation and flutter	80 to 84	Female	20 Cigarette-Equivalents	1·31 (1·07–1·66)
Atrial fibrillation and flutter	80 to 84	Female	30 Cigarette-Equivalents	1·37 (1·10–1·74)
Atrial fibrillation and flutter	85 to 89	Male	0 Cigarette-Equivalents	1·00 (1·00–1·00)
Atrial fibrillation and flutter	85 to 89	Male	1 Cigarette-Equivalents	1·03 (1·00–1·06)
Atrial fibrillation and flutter	85 to 89	Male	5 Cigarette-Equivalents	1·14 (1·00–1·28)
Atrial fibrillation and flutter	85 to 89	Male	10 Cigarette-Equivalents	1·15 (1·03–1·28)
Atrial fibrillation and flutter	85 to 89	Male	20 Cigarette-Equivalents	1·13 (1·01–1·35)
Atrial fibrillation and flutter	85 to 89	Male	30 Cigarette-Equivalents	1·16 (1·00–1·37)
Atrial fibrillation and flutter	85 to 89	Female	0 Cigarette-Equivalents	1·00 (1·00–1·00)

Atrial fibrillation and flutter	85 to 89	Female	1 Cigarette-Equivalents	1·03 (1·00–1·06)
Atrial fibrillation and flutter	85 to 89	Female	5 Cigarette-Equivalents	1·14 (1·00–1·28)
Atrial fibrillation and flutter	85 to 89	Female	10 Cigarette-Equivalents	1·15 (1·03–1·28)
Atrial fibrillation and flutter	85 to 89	Female	20 Cigarette-Equivalents	1·13 (1·01–1·35)
Atrial fibrillation and flutter	85 to 89	Female	30 Cigarette-Equivalents	1·16 (1·00–1·37)
Atrial fibrillation and flutter	90 to 94	Male	0 Cigarette-Equivalents	1·00 (1·00–1·00)
Atrial fibrillation and flutter	90 to 94	Male	1 Cigarette-Equivalents	1·03 (1·00–1·06)
Atrial fibrillation and flutter	90 to 94	Male	5 Cigarette-Equivalents	1·14 (1·00–1·28)
Atrial fibrillation and flutter	90 to 94	Male	10 Cigarette-Equivalents	1·15 (1·03–1·28)
Atrial fibrillation and flutter	90 to 94	Male	20 Cigarette-Equivalents	1·13 (1·01–1·35)
Atrial fibrillation and flutter	90 to 94	Male	30 Cigarette-Equivalents	1·16 (1·00–1·37)
Atrial fibrillation and flutter	90 to 94	Female	0 Cigarette-Equivalents	1·00 (1·00–1·00)
Atrial fibrillation and flutter	90 to 94	Female	1 Cigarette-Equivalents	1·03 (1·00–1·06)
Atrial fibrillation and flutter	90 to 94	Female	5 Cigarette-Equivalents	1·14 (1·00–1·28)
Atrial fibrillation and flutter	90 to 94	Female	10 Cigarette-Equivalents	1·15 (1·03–1·28)
Atrial fibrillation and flutter	90 to 94	Female	20 Cigarette-Equivalents	1·13 (1·01–1·35)
Atrial fibrillation and flutter	90 to 94	Female	30 Cigarette-Equivalents	1·16 (1·00–1·37)
Atrial fibrillation and flutter	95 plus	Male	0 Cigarette-Equivalents	1·00 (1·00–1·00)
Atrial fibrillation and flutter	95 plus	Male	1 Cigarette-Equivalents	1·00 (1·00–1·01)
Atrial fibrillation and flutter	95 plus	Male	5 Cigarette-Equivalents	1·01 (1·00–1·04)
Atrial fibrillation and flutter	95 plus	Male	10 Cigarette-Equivalents	1·02 (1·00–1·07)
Atrial fibrillation and flutter	95 plus	Male	20 Cigarette-Equivalents	1·03 (1·00–1·13)
Atrial fibrillation and flutter	95 plus	Male	30 Cigarette-Equivalents	1·01 (1·00–1·09)
Atrial fibrillation and flutter	95 plus	Female	0 Cigarette-Equivalents	1·00 (1·00–1·00)
Atrial fibrillation and flutter	95 plus	Female	1 Cigarette-Equivalents	1·00 (1·00–1·01)
Atrial fibrillation and flutter	95 plus	Female	5 Cigarette-Equivalents	1·01 (1·00–1·04)
Atrial fibrillation and flutter	95 plus	Female	10 Cigarette-Equivalents	1·02 (1·00–1·07)

Atrial fibrillation and flutter	95 plus	Female	20 Cigarette-Equivalents	1·03 (1·00–1·13)
Atrial fibrillation and flutter	95 plus	Female	30 Cigarette-Equivalents	1·01 (1·00–1·09)
Aortic aneurysm	30 to 34	Male	0 Cigarette-Equivalents	1·00 (1·00–1·00)
Aortic aneurysm	30 to 34	Male	1 Cigarette-Equivalents	1·34 (1·22–1·46)
Aortic aneurysm	30 to 34	Male	5 Cigarette-Equivalents	2·69 (2·12–3·30)
Aortic aneurysm	30 to 34	Male	10 Cigarette-Equivalents	4·37 (3·24–5·60)
Aortic aneurysm	30 to 34	Male	20 Cigarette-Equivalents	8·04 (6·14–10·1)
Aortic aneurysm	30 to 34	Male	30 Cigarette-Equivalents	11·0 (7·98–14·5)
Aortic aneurysm	30 to 34	Female	0 Cigarette-Equivalents	1·00 (1·00–1·00)
Aortic aneurysm	30 to 34	Female	1 Cigarette-Equivalents	1·34 (1·22–1·46)
Aortic aneurysm	30 to 34	Female	5 Cigarette-Equivalents	2·69 (2·12–3·30)
Aortic aneurysm	30 to 34	Female	10 Cigarette-Equivalents	4·37 (3·24–5·60)
Aortic aneurysm	30 to 34	Female	20 Cigarette-Equivalents	8·04 (6·14–10·1)
Aortic aneurysm	30 to 34	Female	30 Cigarette-Equivalents	11·0 (7·98–14·5)
Aortic aneurysm	35 to 39	Male	0 Cigarette-Equivalents	1·00 (1·00–1·00)
Aortic aneurysm	35 to 39	Male	1 Cigarette-Equivalents	1·34 (1·22–1·46)
Aortic aneurysm	35 to 39	Male	5 Cigarette-Equivalents	2·69 (2·12–3·30)
Aortic aneurysm	35 to 39	Male	10 Cigarette-Equivalents	4·37 (3·24–5·60)
Aortic aneurysm	35 to 39	Male	20 Cigarette-Equivalents	8·04 (6·14–10·1)
Aortic aneurysm	35 to 39	Male	30 Cigarette-Equivalents	11·0 (7·98–14·5)
Aortic aneurysm	35 to 39	Female	0 Cigarette-Equivalents	1·00 (1·00–1·00)
Aortic aneurysm	35 to 39	Female	1 Cigarette-Equivalents	1·34 (1·22–1·46)
Aortic aneurysm	35 to 39	Female	5 Cigarette-Equivalents	2·69 (2·12–3·30)
Aortic aneurysm	35 to 39	Female	10 Cigarette-Equivalents	4·37 (3·24–5·60)
Aortic aneurysm	35 to 39	Female	20 Cigarette-Equivalents	8·04 (6·14–10·1)
Aortic aneurysm	35 to 39	Female	30 Cigarette-Equivalents	11·0 (7·98–14·5)
Aortic aneurysm	40 to 44	Male	0 Cigarette-Equivalents	1·00 (1·00–1·00)

Aortic aneurysm	40 to 44	Male	1 Cigarette-Equivalents	1·34 (1·22–1·46)
Aortic aneurysm	40 to 44	Male	5 Cigarette-Equivalents	2·69 (2·12–3·30)
Aortic aneurysm	40 to 44	Male	10 Cigarette-Equivalents	4·37 (3·24–5·60)
Aortic aneurysm	40 to 44	Male	20 Cigarette-Equivalents	8·04 (6·14–10·1)
Aortic aneurysm	40 to 44	Male	30 Cigarette-Equivalents	11·0 (7·98–14·5)
Aortic aneurysm	40 to 44	Female	0 Cigarette-Equivalents	1·00 (1·00–1·00)
Aortic aneurysm	40 to 44	Female	1 Cigarette-Equivalents	1·34 (1·22–1·46)
Aortic aneurysm	40 to 44	Female	5 Cigarette-Equivalents	2·69 (2·12–3·30)
Aortic aneurysm	40 to 44	Female	10 Cigarette-Equivalents	4·37 (3·24–5·60)
Aortic aneurysm	40 to 44	Female	20 Cigarette-Equivalents	8·04 (6·14–10·1)
Aortic aneurysm	40 to 44	Female	30 Cigarette-Equivalents	11·0 (7·98–14·5)
Aortic aneurysm	45 to 49	Male	0 Cigarette-Equivalents	1·00 (1·00–1·00)
Aortic aneurysm	45 to 49	Male	1 Cigarette-Equivalents	1·27 (1·18–1·36)
Aortic aneurysm	45 to 49	Male	5 Cigarette-Equivalents	2·33 (1·92–2·79)
Aortic aneurysm	45 to 49	Male	10 Cigarette-Equivalents	3·67 (2·84–4·58)
Aortic aneurysm	45 to 49	Male	20 Cigarette-Equivalents	5·88 (4·69–7·23)
Aortic aneurysm	45 to 49	Male	30 Cigarette-Equivalents	7·61 (5·89–9·60)
Aortic aneurysm	45 to 49	Female	0 Cigarette-Equivalents	1·00 (1·00–1·00)
Aortic aneurysm	45 to 49	Female	1 Cigarette-Equivalents	1·27 (1·18–1·36)
Aortic aneurysm	45 to 49	Female	5 Cigarette-Equivalents	2·33 (1·92–2·79)
Aortic aneurysm	45 to 49	Female	10 Cigarette-Equivalents	3·67 (2·84–4·58)
Aortic aneurysm	45 to 49	Female	20 Cigarette-Equivalents	5·88 (4·69–7·23)
Aortic aneurysm	45 to 49	Female	30 Cigarette-Equivalents	7·61 (5·89–9·60)
Aortic aneurysm	50 to 54	Male	0 Cigarette-Equivalents	1·00 (1·00–1·00)
Aortic aneurysm	50 to 54	Male	1 Cigarette-Equivalents	1·27 (1·18–1·36)
Aortic aneurysm	50 to 54	Male	5 Cigarette-Equivalents	2·33 (1·92–2·79)
Aortic aneurysm	50 to 54	Male	10 Cigarette-Equivalents	3·67 (2·84–4·58)

Aortic aneurysm	50 to 54	Male	20 Cigarette-Equivalents	5·88 (4·69–7·23)
Aortic aneurysm	50 to 54	Male	30 Cigarette-Equivalents	7·61 (5·89–9·60)
Aortic aneurysm	50 to 54	Female	0 Cigarette-Equivalents	1·00 (1·00–1·00)
Aortic aneurysm	50 to 54	Female	1 Cigarette-Equivalents	1·27 (1·18–1·36)
Aortic aneurysm	50 to 54	Female	5 Cigarette-Equivalents	2·33 (1·92–2·79)
Aortic aneurysm	50 to 54	Female	10 Cigarette-Equivalents	3·67 (2·84–4·58)
Aortic aneurysm	50 to 54	Female	20 Cigarette-Equivalents	5·88 (4·69–7·23)
Aortic aneurysm	50 to 54	Female	30 Cigarette-Equivalents	7·61 (5·89–9·60)
Aortic aneurysm	55 to 59	Male	0 Cigarette-Equivalents	1·00 (1·00–1·00)
Aortic aneurysm	55 to 59	Male	1 Cigarette-Equivalents	1·20 (1·14–1·26)
Aortic aneurysm	55 to 59	Male	5 Cigarette-Equivalents	2·00 (1·71–2·32)
Aortic aneurysm	55 to 59	Male	10 Cigarette-Equivalents	3·00 (2·43–3·64)
Aortic aneurysm	55 to 59	Male	20 Cigarette-Equivalents	4·21 (3·54–4·98)
Aortic aneurysm	55 to 59	Male	30 Cigarette-Equivalents	5·12 (4·19–6·21)
Aortic aneurysm	55 to 59	Female	0 Cigarette-Equivalents	1·00 (1·00–1·00)
Aortic aneurysm	55 to 59	Female	1 Cigarette-Equivalents	1·20 (1·14–1·26)
Aortic aneurysm	55 to 59	Female	5 Cigarette-Equivalents	2·00 (1·71–2·32)
Aortic aneurysm	55 to 59	Female	10 Cigarette-Equivalents	3·00 (2·43–3·64)
Aortic aneurysm	55 to 59	Female	20 Cigarette-Equivalents	4·21 (3·54–4·98)
Aortic aneurysm	55 to 59	Female	30 Cigarette-Equivalents	5·12 (4·19–6·21)
Aortic aneurysm	60 to 64	Male	0 Cigarette-Equivalents	1·00 (1·00–1·00)
Aortic aneurysm	60 to 64	Male	1 Cigarette-Equivalents	1·20 (1·14–1·26)
Aortic aneurysm	60 to 64	Male	5 Cigarette-Equivalents	2·00 (1·71–2·32)
Aortic aneurysm	60 to 64	Male	10 Cigarette-Equivalents	3·00 (2·43–3·64)
Aortic aneurysm	60 to 64	Male	20 Cigarette-Equivalents	4·21 (3·54–4·98)
Aortic aneurysm	60 to 64	Male	30 Cigarette-Equivalents	5·12 (4·19–6·21)
Aortic aneurysm	60 to 64	Female	0 Cigarette-Equivalents	1·00 (1·00–1·00)

Aortic aneurysm	60 to 64	Female	1 Cigarette-Equivalents	1·20 (1·14–1·26)
Aortic aneurysm	60 to 64	Female	5 Cigarette-Equivalents	2·00 (1·71–2·32)
Aortic aneurysm	60 to 64	Female	10 Cigarette-Equivalents	3·00 (2·43–3·64)
Aortic aneurysm	60 to 64	Female	20 Cigarette-Equivalents	4·21 (3·54–4·98)
Aortic aneurysm	60 to 64	Female	30 Cigarette-Equivalents	5·12 (4·19–6·21)
Aortic aneurysm	65 to 69	Male	0 Cigarette-Equivalents	1·00 (1·00–1·00)
Aortic aneurysm	65 to 69	Male	1 Cigarette-Equivalents	1·14 (1·11–1·17)
Aortic aneurysm	65 to 69	Male	5 Cigarette-Equivalents	1·70 (1·53–1·85)
Aortic aneurysm	65 to 69	Male	10 Cigarette-Equivalents	2·39 (2·05–2·70)
Aortic aneurysm	65 to 69	Male	20 Cigarette-Equivalents	2·97 (2·59–3·38)
Aortic aneurysm	65 to 69	Male	30 Cigarette-Equivalents	3·41 (2·92–3·97)
Aortic aneurysm	65 to 69	Female	0 Cigarette-Equivalents	1·00 (1·00–1·00)
Aortic aneurysm	65 to 69	Female	1 Cigarette-Equivalents	1·14 (1·11–1·17)
Aortic aneurysm	65 to 69	Female	5 Cigarette-Equivalents	1·70 (1·53–1·85)
Aortic aneurysm	65 to 69	Female	10 Cigarette-Equivalents	2·39 (2·05–2·70)
Aortic aneurysm	65 to 69	Female	20 Cigarette-Equivalents	2·97 (2·59–3·38)
Aortic aneurysm	65 to 69	Female	30 Cigarette-Equivalents	3·41 (2·92–3·97)
Aortic aneurysm	70 to 74	Male	0 Cigarette-Equivalents	1·00 (1·00–1·00)
Aortic aneurysm	70 to 74	Male	1 Cigarette-Equivalents	1·14 (1·11–1·17)
Aortic aneurysm	70 to 74	Male	5 Cigarette-Equivalents	1·70 (1·53–1·85)
Aortic aneurysm	70 to 74	Male	10 Cigarette-Equivalents	2·39 (2·05–2·70)
Aortic aneurysm	70 to 74	Male	20 Cigarette-Equivalents	2·97 (2·59–3·38)
Aortic aneurysm	70 to 74	Male	30 Cigarette-Equivalents	3·41 (2·92–3·97)
Aortic aneurysm	70 to 74	Female	0 Cigarette-Equivalents	1·00 (1·00–1·00)
Aortic aneurysm	70 to 74	Female	1 Cigarette-Equivalents	1·14 (1·11–1·17)
Aortic aneurysm	70 to 74	Female	5 Cigarette-Equivalents	1·70 (1·53–1·85)
Aortic aneurysm	70 to 74	Female	10 Cigarette-Equivalents	2·39 (2·05–2·70)

Aortic aneurysm	70 to 74	Female	20 Cigarette-Equivalents	2·97 (2·59–3·38)
Aortic aneurysm	70 to 74	Female	30 Cigarette-Equivalents	3·41 (2·92–3·97)
Aortic aneurysm	75 to 79	Male	0 Cigarette-Equivalents	1·00 (1·00–1·00)
Aortic aneurysm	75 to 79	Male	1 Cigarette-Equivalents	1·08 (1·07–1·10)
Aortic aneurysm	75 to 79	Male	5 Cigarette-Equivalents	1·42 (1·33–1·52)
Aortic aneurysm	75 to 79	Male	10 Cigarette-Equivalents	1·84 (1·66–2·04)
Aortic aneurysm	75 to 79	Male	20 Cigarette-Equivalents	2·06 (1·87–2·26)
Aortic aneurysm	75 to 79	Male	30 Cigarette-Equivalents	2·27 (2·00–2·55)
Aortic aneurysm	75 to 79	Female	0 Cigarette-Equivalents	1·00 (1·00–1·00)
Aortic aneurysm	75 to 79	Female	1 Cigarette-Equivalents	1·08 (1·07–1·10)
Aortic aneurysm	75 to 79	Female	5 Cigarette-Equivalents	1·42 (1·33–1·52)
Aortic aneurysm	75 to 79	Female	10 Cigarette-Equivalents	1·84 (1·66–2·04)
Aortic aneurysm	75 to 79	Female	20 Cigarette-Equivalents	2·06 (1·87–2·26)
Aortic aneurysm	75 to 79	Female	30 Cigarette-Equivalents	2·27 (2·00–2·55)
Aortic aneurysm	80 to 84	Male	0 Cigarette-Equivalents	1·00 (1·00–1·00)
Aortic aneurysm	80 to 84	Male	1 Cigarette-Equivalents	1·08 (1·07–1·10)
Aortic aneurysm	80 to 84	Male	5 Cigarette-Equivalents	1·42 (1·33–1·52)
Aortic aneurysm	80 to 84	Male	10 Cigarette-Equivalents	1·84 (1·66–2·04)
Aortic aneurysm	80 to 84	Male	20 Cigarette-Equivalents	2·06 (1·87–2·26)
Aortic aneurysm	80 to 84	Male	30 Cigarette-Equivalents	2·27 (2·00–2·55)
Aortic aneurysm	80 to 84	Female	0 Cigarette-Equivalents	1·00 (1·00–1·00)
Aortic aneurysm	80 to 84	Female	1 Cigarette-Equivalents	1·08 (1·07–1·10)
Aortic aneurysm	80 to 84	Female	5 Cigarette-Equivalents	1·42 (1·33–1·52)
Aortic aneurysm	80 to 84	Female	10 Cigarette-Equivalents	1·84 (1·66–2·04)
Aortic aneurysm	80 to 84	Female	20 Cigarette-Equivalents	2·06 (1·87–2·26)
Aortic aneurysm	80 to 84	Female	30 Cigarette-Equivalents	2·27 (2·00–2·55)
Aortic aneurysm	85 to 89	Male	0 Cigarette-Equivalents	1·00 (1·00–1·00)

Aortic aneurysm	85 to 89	Male	1 Cigarette-Equivalents	1·04 (1·03–1·05)
Aortic aneurysm	85 to 89	Male	5 Cigarette-Equivalents	1·18 (1·14–1·23)
Aortic aneurysm	85 to 89	Male	10 Cigarette-Equivalents	1·37 (1·27–1·47)
Aortic aneurysm	85 to 89	Male	20 Cigarette-Equivalents	1·41 (1·30–1·51)
Aortic aneurysm	85 to 89	Male	30 Cigarette-Equivalents	1·49 (1·36–1·61)
Aortic aneurysm	85 to 89	Female	0 Cigarette-Equivalents	1·00 (1·00–1·00)
Aortic aneurysm	85 to 89	Female	1 Cigarette-Equivalents	1·04 (1·03–1·05)
Aortic aneurysm	85 to 89	Female	5 Cigarette-Equivalents	1·18 (1·14–1·23)
Aortic aneurysm	85 to 89	Female	10 Cigarette-Equivalents	1·37 (1·27–1·47)
Aortic aneurysm	85 to 89	Female	20 Cigarette-Equivalents	1·41 (1·30–1·51)
Aortic aneurysm	85 to 89	Female	30 Cigarette-Equivalents	1·49 (1·36–1·61)
Aortic aneurysm	90 to 94	Male	0 Cigarette-Equivalents	1·00 (1·00–1·00)
Aortic aneurysm	90 to 94	Male	1 Cigarette-Equivalents	1·04 (1·03–1·05)
Aortic aneurysm	90 to 94	Male	5 Cigarette-Equivalents	1·18 (1·14–1·23)
Aortic aneurysm	90 to 94	Male	10 Cigarette-Equivalents	1·37 (1·27–1·47)
Aortic aneurysm	90 to 94	Male	20 Cigarette-Equivalents	1·41 (1·30–1·51)
Aortic aneurysm	90 to 94	Male	30 Cigarette-Equivalents	1·49 (1·36–1·61)
Aortic aneurysm	90 to 94	Female	0 Cigarette-Equivalents	1·00 (1·00–1·00)
Aortic aneurysm	90 to 94	Female	1 Cigarette-Equivalents	1·04 (1·03–1·05)
Aortic aneurysm	90 to 94	Female	5 Cigarette-Equivalents	1·18 (1·14–1·23)
Aortic aneurysm	90 to 94	Female	10 Cigarette-Equivalents	1·37 (1·27–1·47)
Aortic aneurysm	90 to 94	Female	20 Cigarette-Equivalents	1·41 (1·30–1·51)
Aortic aneurysm	90 to 94	Female	30 Cigarette-Equivalents	1·49 (1·36–1·61)
Aortic aneurysm	95 plus	Male	0 Cigarette-Equivalents	1·00 (1·00–1·00)
Aortic aneurysm	95 plus	Male	1 Cigarette-Equivalents	1·00 (1·00–1·00)
Aortic aneurysm	95 plus	Male	5 Cigarette-Equivalents	1·00 (1·00–1·01)
Aortic aneurysm	95 plus	Male	10 Cigarette-Equivalents	1·00 (1·00–1·02)

Aortic aneurysm	95 plus	Male	20 Cigarette-Equivalents	1·00 (1·00–1·02)
Aortic aneurysm	95 plus	Male	30 Cigarette-Equivalents	1·01 (1·00–1·02)
Aortic aneurysm	95 plus	Female	0 Cigarette-Equivalents	1·00 (1·00–1·00)
Aortic aneurysm	95 plus	Female	1 Cigarette-Equivalents	1·00 (1·00–1·00)
Aortic aneurysm	95 plus	Female	5 Cigarette-Equivalents	1·00 (1·00–1·01)
Aortic aneurysm	95 plus	Female	10 Cigarette-Equivalents	1·00 (1·00–1·02)
Aortic aneurysm	95 plus	Female	20 Cigarette-Equivalents	1·00 (1·00–1·02)
Aortic aneurysm	95 plus	Female	30 Cigarette-Equivalents	1·01 (1·00–1·02)
Lower extremity peripheral arterial disease	30 to 34	Male	0 Cigarette-Equivalents	1·00 (1·00–1·00)
Lower extremity peripheral arterial disease	30 to 34	Male	1 Cigarette-Equivalents	1·22 (1·11–1·39)
Lower extremity peripheral arterial disease	30 to 34	Male	5 Cigarette-Equivalents	2·09 (1·53–2·93)
Lower extremity peripheral arterial disease	30 to 34	Male	10 Cigarette-Equivalents	3·28 (2·18–4·87)
Lower extremity peripheral arterial disease	30 to 34	Male	20 Cigarette-Equivalents	6·77 (3·94–10·8)
Lower extremity peripheral arterial disease	30 to 34	Male	30 Cigarette-Equivalents	9·40 (4·71–16·5)
Lower extremity peripheral arterial disease	30 to 34	Female	0 Cigarette-Equivalents	1·00 (1·00–1·00)
Lower extremity peripheral arterial disease	30 to 34	Female	1 Cigarette-Equivalents	1·22 (1·11–1·39)
Lower extremity peripheral arterial disease	30 to 34	Female	5 Cigarette-Equivalents	2·09 (1·53–2·93)
Lower extremity peripheral arterial disease	30 to 34	Female	10 Cigarette-Equivalents	3·28 (2·18–4·87)
Lower extremity peripheral arterial disease	30 to 34	Female	20 Cigarette-Equivalents	6·77 (3·94–10·8)
Lower extremity peripheral arterial disease	30 to 34	Female	30 Cigarette-Equivalents	9·40 (4·71–16·5)
Lower extremity peripheral arterial disease	35 to 39	Male	0 Cigarette-Equivalents	1·00 (1·00–1·00)
Lower extremity peripheral arterial disease	35 to 39	Male	1 Cigarette-Equivalents	1·22 (1·11–1·39)
Lower extremity peripheral arterial disease	35 to 39	Male	5 Cigarette-Equivalents	2·09 (1·53–2·93)
Lower extremity peripheral arterial disease	35 to 39	Male	10 Cigarette-Equivalents	3·28 (2·18–4·87)
Lower extremity peripheral arterial disease	35 to 39	Male	20 Cigarette-Equivalents	6·77 (3·94–10·8)
Lower extremity peripheral arterial disease	35 to 39	Male	30 Cigarette-Equivalents	9·40 (4·71–16·5)
Lower extremity peripheral arterial disease	35 to 39	Female	0 Cigarette-Equivalents	1·00 (1·00–1·00)

Lower extremity peripheral arterial disease	35 to 39	Female	1 Cigarette-Equivalents	1·22 (1·11–1·39)
Lower extremity peripheral arterial disease	35 to 39	Female	5 Cigarette-Equivalents	2·09 (1·53–2·93)
Lower extremity peripheral arterial disease	35 to 39	Female	10 Cigarette-Equivalents	3·28 (2·18–4·87)
Lower extremity peripheral arterial disease	35 to 39	Female	20 Cigarette-Equivalents	6·77 (3·94–10·8)
Lower extremity peripheral arterial disease	35 to 39	Female	30 Cigarette-Equivalents	9·40 (4·71–16·5)
Lower extremity peripheral arterial disease	40 to 44	Male	0 Cigarette-Equivalents	1·00 (1·00–1·00)
Lower extremity peripheral arterial disease	40 to 44	Male	1 Cigarette-Equivalents	1·22 (1·11–1·39)
Lower extremity peripheral arterial disease	40 to 44	Male	5 Cigarette-Equivalents	2·09 (1·53–2·93)
Lower extremity peripheral arterial disease	40 to 44	Male	10 Cigarette-Equivalents	3·28 (2·18–4·87)
Lower extremity peripheral arterial disease	40 to 44	Male	20 Cigarette-Equivalents	6·77 (3·94–10·8)
Lower extremity peripheral arterial disease	40 to 44	Male	30 Cigarette-Equivalents	9·40 (4·71–16·5)
Lower extremity peripheral arterial disease	40 to 44	Female	0 Cigarette-Equivalents	1·00 (1·00–1·00)
Lower extremity peripheral arterial disease	40 to 44	Female	1 Cigarette-Equivalents	1·22 (1·11–1·39)
Lower extremity peripheral arterial disease	40 to 44	Female	5 Cigarette-Equivalents	2·09 (1·53–2·93)
Lower extremity peripheral arterial disease	40 to 44	Female	10 Cigarette-Equivalents	3·28 (2·18–4·87)
Lower extremity peripheral arterial disease	40 to 44	Female	20 Cigarette-Equivalents	6·77 (3·94–10·8)
Lower extremity peripheral arterial disease	40 to 44	Female	30 Cigarette-Equivalents	9·40 (4·71–16·5)
Lower extremity peripheral arterial disease	45 to 49	Male	0 Cigarette-Equivalents	1·00 (1·00–1·00)
Lower extremity peripheral arterial disease	45 to 49	Male	1 Cigarette-Equivalents	1·19 (1·08–1·35)
Lower extremity peripheral arterial disease	45 to 49	Male	5 Cigarette-Equivalents	1·96 (1·41–2·74)
Lower extremity peripheral arterial disease	45 to 49	Male	10 Cigarette-Equivalents	2·98 (1·89–4·48)
Lower extremity peripheral arterial disease	45 to 49	Male	20 Cigarette-Equivalents	5·74 (3·25–9·15)
Lower extremity peripheral arterial disease	45 to 49	Male	30 Cigarette-Equivalents	7·75 (3·77–13·9)
Lower extremity peripheral arterial disease	45 to 49	Female	0 Cigarette-Equivalents	1·00 (1·00–1·00)
Lower extremity peripheral arterial disease	45 to 49	Female	1 Cigarette-Equivalents	1·19 (1·08–1·35)
Lower extremity peripheral arterial disease	45 to 49	Female	5 Cigarette-Equivalents	1·96 (1·41–2·74)
Lower extremity peripheral arterial disease	45 to 49	Female	10 Cigarette-Equivalents	2·98 (1·89–4·48)

Lower extremity peripheral arterial disease	45 to 49	Female	20 Cigarette-Equivalents	5·74 (3·25–9·15)
Lower extremity peripheral arterial disease	45 to 49	Female	30 Cigarette-Equivalents	7·75 (3·77–13·9)
Lower extremity peripheral arterial disease	50 to 54	Male	0 Cigarette-Equivalents	1·00 (1·00–1·00)
Lower extremity peripheral arterial disease	50 to 54	Male	1 Cigarette-Equivalents	1·19 (1·08–1·35)
Lower extremity peripheral arterial disease	50 to 54	Male	5 Cigarette-Equivalents	1·96 (1·41–2·74)
Lower extremity peripheral arterial disease	50 to 54	Male	10 Cigarette-Equivalents	2·98 (1·89–4·48)
Lower extremity peripheral arterial disease	50 to 54	Male	20 Cigarette-Equivalents	5·74 (3·25–9·15)
Lower extremity peripheral arterial disease	50 to 54	Male	30 Cigarette-Equivalents	7·75 (3·77–13·9)
Lower extremity peripheral arterial disease	50 to 54	Female	0 Cigarette-Equivalents	1·00 (1·00–1·00)
Lower extremity peripheral arterial disease	50 to 54	Female	1 Cigarette-Equivalents	1·19 (1·08–1·35)
Lower extremity peripheral arterial disease	50 to 54	Female	5 Cigarette-Equivalents	1·96 (1·41–2·74)
Lower extremity peripheral arterial disease	50 to 54	Female	10 Cigarette-Equivalents	2·98 (1·89–4·48)
Lower extremity peripheral arterial disease	50 to 54	Female	20 Cigarette-Equivalents	5·74 (3·25–9·15)
Lower extremity peripheral arterial disease	50 to 54	Female	30 Cigarette-Equivalents	7·75 (3·77–13·9)
Lower extremity peripheral arterial disease	55 to 59	Male	0 Cigarette-Equivalents	1·00 (1·00–1·00)
Lower extremity peripheral arterial disease	55 to 59	Male	1 Cigarette-Equivalents	1·22 (1·08–1·40)
Lower extremity peripheral arterial disease	55 to 59	Male	5 Cigarette-Equivalents	2·12 (1·39–3·01)
Lower extremity peripheral arterial disease	55 to 59	Male	10 Cigarette-Equivalents	3·27 (1·82–4·93)
Lower extremity peripheral arterial disease	55 to 59	Male	20 Cigarette-Equivalents	5·90 (2·88–8·81)
Lower extremity peripheral arterial disease	55 to 59	Male	30 Cigarette-Equivalents	7·32 (3·30–11·3)
Lower extremity peripheral arterial disease	55 to 59	Female	0 Cigarette-Equivalents	1·00 (1·00–1·00)
Lower extremity peripheral arterial disease	55 to 59	Female	1 Cigarette-Equivalents	1·22 (1·08–1·40)
Lower extremity peripheral arterial disease	55 to 59	Female	5 Cigarette-Equivalents	2·12 (1·39–3·01)
Lower extremity peripheral arterial disease	55 to 59	Female	10 Cigarette-Equivalents	3·27 (1·82–4·93)
Lower extremity peripheral arterial disease	55 to 59	Female	20 Cigarette-Equivalents	5·90 (2·88–8·81)
Lower extremity peripheral arterial disease	55 to 59	Female	30 Cigarette-Equivalents	7·32 (3·30–11·3)
Lower extremity peripheral arterial disease	60 to 64	Male	0 Cigarette-Equivalents	1·00 (1·00–1·00)

Lower extremity peripheral arterial disease	60 to 64	Male	1 Cigarette-Equivalents	1·22 (1·08–1·40)
Lower extremity peripheral arterial disease	60 to 64	Male	5 Cigarette-Equivalents	2·12 (1·39–3·01)
Lower extremity peripheral arterial disease	60 to 64	Male	10 Cigarette-Equivalents	3·27 (1·82–4·93)
Lower extremity peripheral arterial disease	60 to 64	Male	20 Cigarette-Equivalents	5·90 (2·88–8·81)
Lower extremity peripheral arterial disease	60 to 64	Male	30 Cigarette-Equivalents	7·32 (3·30–11·3)
Lower extremity peripheral arterial disease	60 to 64	Female	0 Cigarette-Equivalents	1·00 (1·00–1·00)
Lower extremity peripheral arterial disease	60 to 64	Female	1 Cigarette-Equivalents	1·22 (1·08–1·40)
Lower extremity peripheral arterial disease	60 to 64	Female	5 Cigarette-Equivalents	2·12 (1·39–3·01)
Lower extremity peripheral arterial disease	60 to 64	Female	10 Cigarette-Equivalents	3·27 (1·82–4·93)
Lower extremity peripheral arterial disease	60 to 64	Female	20 Cigarette-Equivalents	5·90 (2·88–8·81)
Lower extremity peripheral arterial disease	60 to 64	Female	30 Cigarette-Equivalents	7·32 (3·30–11·3)
Lower extremity peripheral arterial disease	65 to 69	Male	0 Cigarette-Equivalents	1·00 (1·00–1·00)
Lower extremity peripheral arterial disease	65 to 69	Male	1 Cigarette-Equivalents	1·21 (1·13–1·30)
Lower extremity peripheral arterial disease	65 to 69	Male	5 Cigarette-Equivalents	2·06 (1·63–2·48)
Lower extremity peripheral arterial disease	65 to 69	Male	10 Cigarette-Equivalents	3·12 (2·34–3·83)
Lower extremity peripheral arterial disease	65 to 69	Male	20 Cigarette-Equivalents	4·91 (3·76–5·91)
Lower extremity peripheral arterial disease	65 to 69	Male	30 Cigarette-Equivalents	5·31 (3·98–6·97)
Lower extremity peripheral arterial disease	65 to 69	Female	0 Cigarette-Equivalents	1·00 (1·00–1·00)
Lower extremity peripheral arterial disease	65 to 69	Female	1 Cigarette-Equivalents	1·21 (1·13–1·30)
Lower extremity peripheral arterial disease	65 to 69	Female	5 Cigarette-Equivalents	2·06 (1·63–2·48)
Lower extremity peripheral arterial disease	65 to 69	Female	10 Cigarette-Equivalents	3·12 (2·34–3·83)
Lower extremity peripheral arterial disease	65 to 69	Female	20 Cigarette-Equivalents	4·91 (3·76–5·91)
Lower extremity peripheral arterial disease	65 to 69	Female	30 Cigarette-Equivalents	5·31 (3·98–6·97)
Lower extremity peripheral arterial disease	70 to 74	Male	0 Cigarette-Equivalents	1·00 (1·00–1·00)
Lower extremity peripheral arterial disease	70 to 74	Male	1 Cigarette-Equivalents	1·21 (1·13–1·30)
Lower extremity peripheral arterial disease	70 to 74	Male	5 Cigarette-Equivalents	2·06 (1·63–2·48)
Lower extremity peripheral arterial disease	70 to 74	Male	10 Cigarette-Equivalents	3·12 (2·34–3·83)

Lower extremity peripheral arterial disease	70 to 74	Male	20 Cigarette-Equivalents	4·91 (3·76–5·91)
Lower extremity peripheral arterial disease	70 to 74	Male	30 Cigarette-Equivalents	5·31 (3·98–6·97)
Lower extremity peripheral arterial disease	70 to 74	Female	0 Cigarette-Equivalents	1·00 (1·00–1·00)
Lower extremity peripheral arterial disease	70 to 74	Female	1 Cigarette-Equivalents	1·21 (1·13–1·30)
Lower extremity peripheral arterial disease	70 to 74	Female	5 Cigarette-Equivalents	2·06 (1·63–2·48)
Lower extremity peripheral arterial disease	70 to 74	Female	10 Cigarette-Equivalents	3·12 (2·34–3·83)
Lower extremity peripheral arterial disease	70 to 74	Female	20 Cigarette-Equivalents	4·91 (3·76–5·91)
Lower extremity peripheral arterial disease	70 to 74	Female	30 Cigarette-Equivalents	5·31 (3·98–6·97)
Lower extremity peripheral arterial disease	75 to 79	Male	0 Cigarette-Equivalents	1·00 (1·00–1·00)
Lower extremity peripheral arterial disease	75 to 79	Male	1 Cigarette-Equivalents	1·13 (1·08–1·18)
Lower extremity peripheral arterial disease	75 to 79	Male	5 Cigarette-Equivalents	1·64 (1·39–1·88)
Lower extremity peripheral arterial disease	75 to 79	Male	10 Cigarette-Equivalents	2·26 (1·81–2·67)
Lower extremity peripheral arterial disease	75 to 79	Male	20 Cigarette-Equivalents	2·99 (2·50–3·45)
Lower extremity peripheral arterial disease	75 to 79	Male	30 Cigarette-Equivalents	2·99 (2·33–3·83)
Lower extremity peripheral arterial disease	75 to 79	Female	0 Cigarette-Equivalents	1·00 (1·00–1·00)
Lower extremity peripheral arterial disease	75 to 79	Female	1 Cigarette-Equivalents	1·13 (1·08–1·18)
Lower extremity peripheral arterial disease	75 to 79	Female	5 Cigarette-Equivalents	1·64 (1·39–1·88)
Lower extremity peripheral arterial disease	75 to 79	Female	10 Cigarette-Equivalents	2·26 (1·81–2·67)
Lower extremity peripheral arterial disease	75 to 79	Female	20 Cigarette-Equivalents	2·99 (2·50–3·45)
Lower extremity peripheral arterial disease	75 to 79	Female	30 Cigarette-Equivalents	2·99 (2·33–3·83)
Lower extremity peripheral arterial disease	80 to 84	Male	0 Cigarette-Equivalents	1·00 (1·00–1·00)
Lower extremity peripheral arterial disease	80 to 84	Male	1 Cigarette-Equivalents	1·13 (1·08–1·18)
Lower extremity peripheral arterial disease	80 to 84	Male	5 Cigarette-Equivalents	1·64 (1·39–1·88)
Lower extremity peripheral arterial disease	80 to 84	Male	10 Cigarette-Equivalents	2·26 (1·81–2·67)
Lower extremity peripheral arterial disease	80 to 84	Male	20 Cigarette-Equivalents	2·99 (2·50–3·45)
Lower extremity peripheral arterial disease	80 to 84	Male	30 Cigarette-Equivalents	2·99 (2·33–3·83)
Lower extremity peripheral arterial disease	80 to 84	Female	0 Cigarette-Equivalents	1·00 (1·00–1·00)

Lower extremity peripheral arterial disease	80 to 84	Female	1 Cigarette-Equivalents	1·13 (1·08–1·18)
Lower extremity peripheral arterial disease	80 to 84	Female	5 Cigarette-Equivalents	1·64 (1·39–1·88)
Lower extremity peripheral arterial disease	80 to 84	Female	10 Cigarette-Equivalents	2·26 (1·81–2·67)
Lower extremity peripheral arterial disease	80 to 84	Female	20 Cigarette-Equivalents	2·99 (2·50–3·45)
Lower extremity peripheral arterial disease	80 to 84	Female	30 Cigarette-Equivalents	2·99 (2·33–3·83)
Lower extremity peripheral arterial disease	85 to 89	Male	0 Cigarette-Equivalents	1·00 (1·00–1·00)
Lower extremity peripheral arterial disease	85 to 89	Male	1 Cigarette-Equivalents	1·06 (1·03–1·09)
Lower extremity peripheral arterial disease	85 to 89	Male	5 Cigarette-Equivalents	1·29 (1·13–1·45)
Lower extremity peripheral arterial disease	85 to 89	Male	10 Cigarette-Equivalents	1·55 (1·27–1·84)
Lower extremity peripheral arterial disease	85 to 89	Male	20 Cigarette-Equivalents	1·73 (1·48–2·01)
Lower extremity peripheral arterial disease	85 to 89	Male	30 Cigarette-Equivalents	1·71 (1·35–2·19)
Lower extremity peripheral arterial disease	85 to 89	Female	0 Cigarette-Equivalents	1·00 (1·00–1·00)
Lower extremity peripheral arterial disease	85 to 89	Female	1 Cigarette-Equivalents	1·06 (1·03–1·09)
Lower extremity peripheral arterial disease	85 to 89	Female	5 Cigarette-Equivalents	1·29 (1·13–1·45)
Lower extremity peripheral arterial disease	85 to 89	Female	10 Cigarette-Equivalents	1·55 (1·27–1·84)
Lower extremity peripheral arterial disease	85 to 89	Female	20 Cigarette-Equivalents	1·73 (1·48–2·01)
Lower extremity peripheral arterial disease	85 to 89	Female	30 Cigarette-Equivalents	1·71 (1·35–2·19)
Lower extremity peripheral arterial disease	90 to 94	Male	0 Cigarette-Equivalents	1·00 (1·00–1·00)
Lower extremity peripheral arterial disease	90 to 94	Male	1 Cigarette-Equivalents	1·06 (1·03–1·09)
Lower extremity peripheral arterial disease	90 to 94	Male	5 Cigarette-Equivalents	1·29 (1·13–1·45)
Lower extremity peripheral arterial disease	90 to 94	Male	10 Cigarette-Equivalents	1·55 (1·27–1·84)
Lower extremity peripheral arterial disease	90 to 94	Male	20 Cigarette-Equivalents	1·73 (1·48–2·01)
Lower extremity peripheral arterial disease	90 to 94	Male	30 Cigarette-Equivalents	1·71 (1·35–2·19)
Lower extremity peripheral arterial disease	90 to 94	Female	0 Cigarette-Equivalents	1·00 (1·00–1·00)
Lower extremity peripheral arterial disease	90 to 94	Female	1 Cigarette-Equivalents	1·06 (1·03–1·09)
Lower extremity peripheral arterial disease	90 to 94	Female	5 Cigarette-Equivalents	1·29 (1·13–1·45)
Lower extremity peripheral arterial disease	90 to 94	Female	10 Cigarette-Equivalents	1·55 (1·27–1·84)

Lower extremity peripheral arterial disease	90 to 94	Female	20 Cigarette-Equivalents	1·73 (1·48–2·01)
Lower extremity peripheral arterial disease	90 to 94	Female	30 Cigarette-Equivalents	1·71 (1·35–2·19)
Lower extremity peripheral arterial disease	95 plus	Male	0 Cigarette-Equivalents	1·00 (1·00–1·00)
Lower extremity peripheral arterial disease	95 plus	Male	1 Cigarette-Equivalents	1·00 (1·00–1·02)
Lower extremity peripheral arterial disease	95 plus	Male	5 Cigarette-Equivalents	1·02 (1·00–1·10)
Lower extremity peripheral arterial disease	95 plus	Male	10 Cigarette-Equivalents	1·04 (1·00–1·18)
Lower extremity peripheral arterial disease	95 plus	Male	20 Cigarette-Equivalents	1·04 (1·00–1·16)
Lower extremity peripheral arterial disease	95 plus	Male	30 Cigarette-Equivalents	1·07 (1·00–1·28)
Lower extremity peripheral arterial disease	95 plus	Female	0 Cigarette-Equivalents	1·00 (1·00–1·00)
Lower extremity peripheral arterial disease	95 plus	Female	1 Cigarette-Equivalents	1·00 (1·00–1·02)
Lower extremity peripheral arterial disease	95 plus	Female	5 Cigarette-Equivalents	1·02 (1·00–1·10)
Lower extremity peripheral arterial disease	95 plus	Female	10 Cigarette-Equivalents	1·04 (1·00–1·18)
Lower extremity peripheral arterial disease	95 plus	Female	20 Cigarette-Equivalents	1·04 (1·00–1·16)
Lower extremity peripheral arterial disease	95 plus	Female	30 Cigarette-Equivalents	1·07 (1·00–1·28)
Chronic obstructive pulmonary disease	All Ages	Both	0 Pack-Years	1·00 (1·00–1·00)
Chronic obstructive pulmonary disease	All Ages	Both	1 Pack-Years	1·26 (1·16–1·36)
Chronic obstructive pulmonary disease	All Ages	Both	5 Pack-Years	2·28 (1·81–2·78)
Chronic obstructive pulmonary disease	All Ages	Both	10 Pack-Years	3·56 (2·63–4·56)
Chronic obstructive pulmonary disease	All Ages	Both	20 Pack-Years	5·79 (4·36–7·36)
Chronic obstructive pulmonary disease	All Ages	Both	40 Pack-Years	7·45 (6·18–9·01)
Chronic obstructive pulmonary disease	All Ages	Both	60 Pack-Years	8·01 (4·24–12·7)
Asthma	All Ages	Both	0 Cigarette-Equivalents	1·00 (1·00–1·00)
Asthma	All Ages	Both	1 Cigarette-Equivalents	1·09 (1·00–1·21)
Asthma	All Ages	Both	5 Cigarette-Equivalents	1·45 (1·13–1·77)
Asthma	All Ages	Both	10 Cigarette-Equivalents	1·73 (1·29–2·14)
Asthma	All Ages	Both	20 Cigarette-Equivalents	1·94 (1·41–2·66)
Asthma	All Ages	Both	30 Cigarette-Equivalents	2·37 (1·45–3·85)

Peptic ulcer disease	All Ages	Both	0 Cigarette-Equivalents	1·00 (1·00–1·00)
Peptic ulcer disease	All Ages	Both	1 Cigarette-Equivalents	1·13 (1·07–1·18)
Peptic ulcer disease	All Ages	Both	5 Cigarette-Equivalents	1·63 (1·35–1·92)
Peptic ulcer disease	All Ages	Both	10 Cigarette-Equivalents	2·00 (1·76–2·26)
Peptic ulcer disease	All Ages	Both	20 Cigarette-Equivalents	2·52 (2·09–2·97)
Peptic ulcer disease	All Ages	Both	30 Cigarette-Equivalents	2·76 (2·21–3·42)
Gallbladder and biliary diseases	All Ages	Both	0 Cigarette-Equivalents	1·00 (1·00–1·00)
Gallbladder and biliary diseases	All Ages	Both	1 Cigarette-Equivalents	1·04 (1·02–1·06)
Gallbladder and biliary diseases	All Ages	Both	5 Cigarette-Equivalents	1·20 (1·11–1·30)
Gallbladder and biliary diseases	All Ages	Both	10 Cigarette-Equivalents	1·35 (1·21–1·50)
Gallbladder and biliary diseases	All Ages	Both	20 Cigarette-Equivalents	1·33 (1·23–1·44)
Gallbladder and biliary diseases	All Ages	Both	30 Cigarette-Equivalents	1·49 (1·34–1·65)
Alzheimer's disease and other dementias	All Ages	Both	0 Cigarette-Equivalents	1·00 (1·00–1·00)
Alzheimer's disease and other dementias	All Ages	Both	1 Cigarette-Equivalents	1·09 (1·03–1·15)
Alzheimer's disease and other dementias	All Ages	Both	5 Cigarette-Equivalents	1·45 (1·16–1·77)
Alzheimer's disease and other dementias	All Ages	Both	10 Cigarette-Equivalents	1·90 (1·33–2·54)
Alzheimer's disease and other dementias	All Ages	Both	20 Cigarette-Equivalents	2·65 (1·77–3·71)
Alzheimer's disease and other dementias	All Ages	Both	30 Cigarette-Equivalents	3·34 (2·07–4·88)
Parkinson's disease	All Ages	Both	0 Cigarette-Equivalents	1·00 (1·00–1·00)
Parkinson's disease	All Ages	Both	1 Cigarette-Equivalents	0·978 (0·953–0·998)
Parkinson's disease	All Ages	Both	5 Cigarette-Equivalents	0·890 (0·763–0·992)
Parkinson's disease	All Ages	Both	10 Cigarette-Equivalents	0·786 (0·626–0·933)
Parkinson's disease	All Ages	Both	20 Cigarette-Equivalents	0·633 (0·480–0·808)
Parkinson's disease	All Ages	Both	30 Cigarette-Equivalents	0·520 (0·346–0·710)
Multiple sclerosis	All Ages	Both	0 Cigarette-Equivalents	1·00 (1·00–1·00)
Multiple sclerosis	All Ages	Both	1 Cigarette-Equivalents	1·07 (1·03–1·11)
Multiple sclerosis	All Ages	Both	5 Cigarette-Equivalents	1·34 (1·14–1·55)

Multiple sclerosis	All Ages	Both	10 Cigarette-Equivalents	1·67 (1·45–1·92)
Multiple sclerosis	All Ages	Both	20 Cigarette-Equivalents	2·01 (1·65–2·39)
Multiple sclerosis	All Ages	Both	30 Cigarette-Equivalents	2·04 (1·58–2·55)
Diabetes mellitus	All Ages	Both	0 Cigarette-Equivalents	1·00 (1·00–1·00)
Diabetes mellitus	All Ages	Both	1 Cigarette-Equivalents	1·07 (1·03–1·10)
Diabetes mellitus	All Ages	Both	5 Cigarette-Equivalents	1·34 (1·16–1·50)
Diabetes mellitus	All Ages	Both	10 Cigarette-Equivalents	1·43 (1·28–1·61)
Diabetes mellitus	All Ages	Both	20 Cigarette-Equivalents	1·64 (1·30–1·98)
Diabetes mellitus	All Ages	Both	30 Cigarette-Equivalents	1·78 (1·42–2·14)
Rheumatoid arthritis	All Ages	Both	0 Cigarette-Equivalents	1·00 (1·00–1·00)
Rheumatoid arthritis	All Ages	Both	1 Cigarette-Equivalents	1·05 (1·00–1·13)
Rheumatoid arthritis	All Ages	Both	5 Cigarette-Equivalents	1·27 (1·00–1·64)
Rheumatoid arthritis	All Ages	Both	10 Cigarette-Equivalents	1·52 (1·07–2·11)
Rheumatoid arthritis	All Ages	Both	20 Cigarette-Equivalents	1·91 (1·18–2·95)
Rheumatoid arthritis	All Ages	Both	30 Cigarette-Equivalents	2·26 (1·15–3·91)
Low back pain	All Ages	Both	0 Cigarette-Equivalents	1·00 (1·00–1·00)
Low back pain	All Ages	Both	1 Cigarette-Equivalents	1·13 (1·04–1·23)
Low back pain	All Ages	Both	5 Cigarette-Equivalents	1·67 (1·39–1·98)
Low back pain	All Ages	Both	10 Cigarette-Equivalents	2·16 (1·75–2·64)
Low back pain	All Ages	Both	20 Cigarette-Equivalents	2·35 (1·61–3·31)
Low back pain	All Ages	Both	30 Cigarette-Equivalents	2·35 (1·61–3·31)
Cataract	All Ages	Both	0 Cigarette-Equivalents	1·00 (1·00–1·00)
Cataract	All Ages	Both	1 Cigarette-Equivalents	1·05 (1·03–1·07)
Cataract	All Ages	Both	5 Cigarette-Equivalents	1·24 (1·16–1·34)
Cataract	All Ages	Both	10 Cigarette-Equivalents	1·44 (1·30–1·60)
Cataract	All Ages	Both	20 Cigarette-Equivalents	1·55 (1·43–1·68)
Cataract	All Ages	Both	30 Cigarette-Equivalents	1·82 (1·49–2·20)

Age-related macular degeneration	All Ages	Both	0 Cigarette-Equivalents	1·00 (1·00–1·00)
Age-related macular degeneration	All Ages	Both	1 Cigarette-Equivalents	1·05 (1·00–1·10)
Age-related macular degeneration	All Ages	Both	5 Cigarette-Equivalents	1·23 (1·00–1·51)
Age-related macular degeneration	All Ages	Both	10 Cigarette-Equivalents	1·45 (1·10–1·88)
Age-related macular degeneration	All Ages	Both	20 Cigarette-Equivalents	1·91 (1·33–2·60)
Age-related macular degeneration	All Ages	Both	30 Cigarette-Equivalents	2·42 (1·62–3·35)
Fracture	All Ages	Both	Prevalence	1·85 (1·52–2·25)

Supplemental Figure S1a. Age-standardized smoking prevalence among males ages 15 and above, 2019.

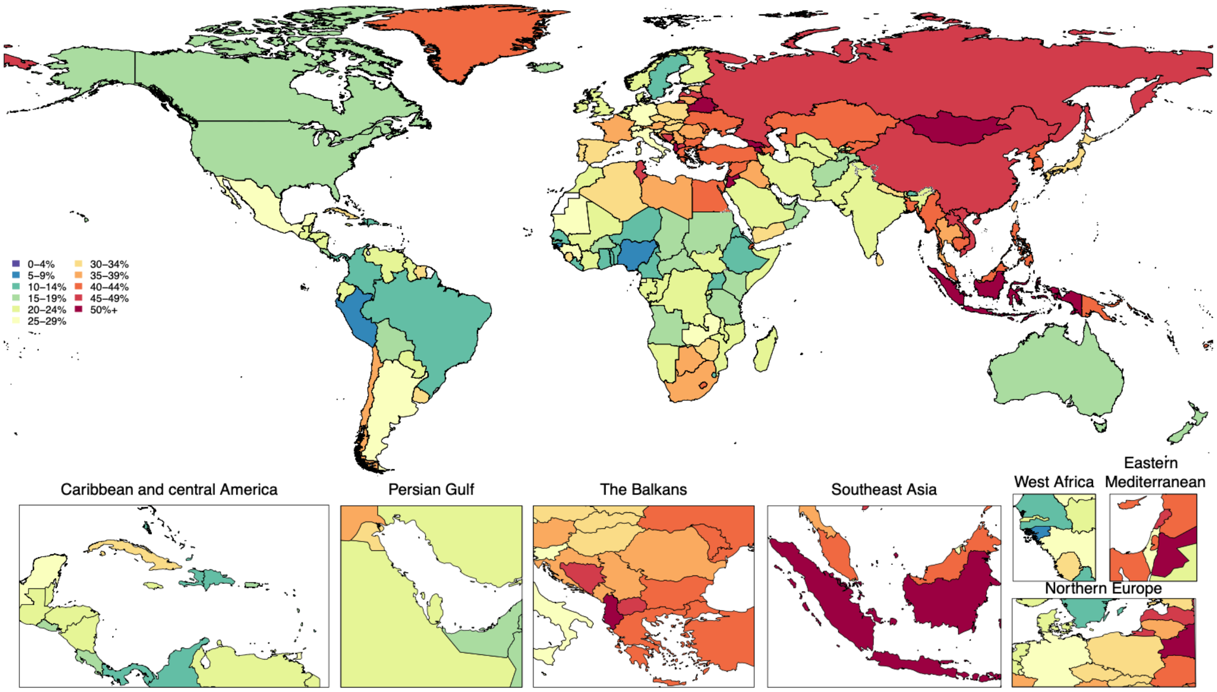

Supplemental Figure S1b. Age-standardized smoking prevalence among females ages 15 and above, 2019.

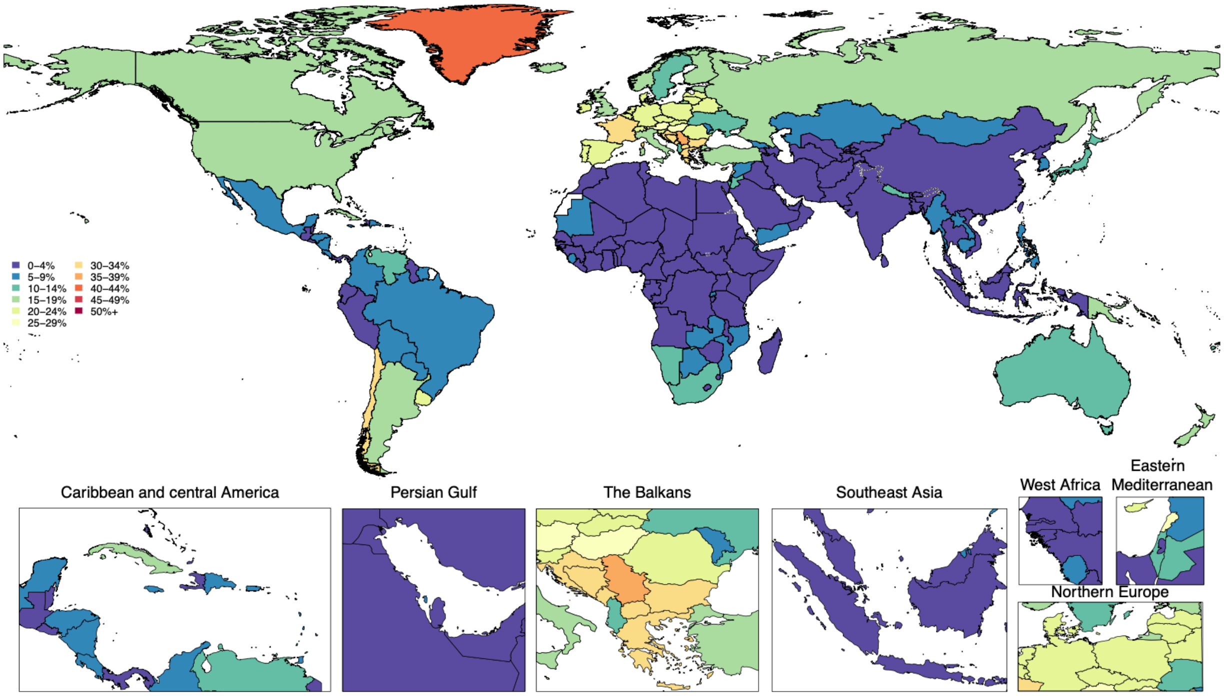

Supplemental Table S2. Number of current smokers (millions), by sex, in 2019, for 204 countries and territories.

Location	Number of Smokers Female (Millions)	Number of Smokers Male (Millions)
**Global**	**194 (189–200)**	**951 (939–962)**
**Central Europe, Eastern Europe, and Central Asia**	**25·6 (24·5–26·7)**	**64·2 (63·3–65·1)**
Central Asia	1·35 (1·22–1·50)	11·5 (11·2–11·8)
Armenia	0·0409 (0·0325–0·0501)	0·647 (0·627–0·667)
Azerbaijan	0·106 (0·0800–0·141)	1·77 (1·70–1·83)
Georgia	0·0989 (0·0843–0·115)	0·712 (0·686–0·736)
Kazakhstan	0·547 (0·460–0·651)	2·91 (2·78–3·03)
Kyrgyzstan	0·110 (0·0893–0·134)	0·956 (0·913–1·00)
Mongolia	0·105 (0·0874–0·126)	0·634 (0·607–0·662)
Tajikistan	0·0419 (0·0316–0·0553)	0·531 (0·488–0·578)
Turkmenistan	0·0558 (0·0428–0·0714)	0·414 (0·379–0·451)
Uzbekistan	0·247 (0·187–0·319)	2·95 (2·74–3·18)
Central Europe	11·5 (11·2–11·9)	16·3 (16·0–16·6)
Albania	0·133 (0·110–0·157)	0·572 (0·552–0·591)
Bosnia and Herzegovina	0·412 (0·362–0·460)	0·617 (0·584–0·649)
Bulgaria	0·791 (0·721–0·862)	1·14 (1·08–1·20)
Croatia	0·545 (0·501–0·589)	0·646 (0·615–0·676)
Czechia	0·940 (0·870–1·02)	1·40 (1·34–1·47)
Hungary	0·989 (0·892–1·09)	1·27 (1·20–1·34)
Montenegro	0·0847 (0·0783–0·0914)	0·0962 (0·0916–0·101)
North Macedonia	0·272 (0·249–0·296)	0·441 (0·422–0·461)
Poland	3·78 (3·50–4·07)	4·94 (4·74–5·17)
Romania	1·68 (1·56–1·82)	2·85 (2·73–2·96)
Serbia	1·28 (1·16–1·39)	1·35 (1·29–1·42)

Slovakia	0·430 (0·386–0·478)	0·727 (0·684–0·771)
Slovenia	0·191 (0·174–0·210)	0·236 (0·223–0·250)
Eastern Europe	12·7 (11·7–13·7)	36·4 (35·5–37·2)
Belarus	0·804 (0·704–0·909)	1·87 (1·78–1·96)
Estonia	0·0972 (0·0883–0·106)	0·160 (0·153–0·167)
Latvia	0·151 (0·137–0·165)	0·335 (0·323–0·347)
Lithuania	0·207 (0·187–0·229)	0·400 (0·382–0·420)
Republic of Moldova	0·104 (0·0856–0·125)	0·602 (0·570–0·633)
Russian Federation	8·96 (8·03–9·91)	25·9 (25·1–26·7)
Ukraine	2·37 (2·08–2·69)	7·09 (6·83–7·34)
**High-income**	**74·3 (72·1–76·7)**	**113 (111–115)**
Australasia	1·65 (1·46–1·86)	1·81 (1·69–1·93)
Australia	1·39 (1·19–1·60)	1·52 (1·40–1·64)
New Zealand	0·263 (0·242–0·286)	0·292 (0·277–0·308)
High-income Asia Pacific	6·34 (5·64–7·13)	27·5 (26·4–28·6)
Brunei Darussalam	0·00866 (0·00674–0·0110)	0·0545 (0·0495–0·0599)
Japan	5·06 (4·37–5·80)	16·9 (16·0–17·8)
Republic of Korea	1·12 (0·907–1·39)	10·0 (9·48–10·6)
Singapore	0·158 (0·125–0·193)	0·533 (0·488–0·582)
High-income North America	22·4 (20·6–24·3)	27·6 (26·3–28·9)
Canada	2·33 (1·99–2·70)	2·59 (2·37–2·80)
Greenland	0·00877 (0·00758–0·0101)	0·0103 (0·00955–0·0110)
United States of America	20·1 (18·3–21·9)	25·0 (23·7–26·4)
Southern Latin America	6·05 (5·51–6·56)	7·79 (7·45–8·14)
Argentina	3·44 (3·03–3·86)	4·85 (4·55–5·15)
Chile	2·31 (2·03–2·59)	2·55 (2·39–2·70)
Uruguay	0·302 (0·256–0·349)	0·391 (0·363–0·420)

Western Europe	37·9 (36·8–39·0)	47·9 (47·1–48·8)
Andorra	0·00791 (0·00636–0·00948)	0·0114 (0·0104–0·0124)
Austria	0·917 (0·829–1·01)	1·27 (1·20–1·34)
Belgium	0·947 (0·874–1·02)	1·10 (1·05–1·15)
Cyprus	0·118 (0·104–0·134)	0·241 (0·230–0·253)
Denmark	0·524 (0·482–0·569)	0·535 (0·506–0·565)
Finland	0·366 (0·332–0·405)	0·517 (0·490–0·546)
France	7·62 (7·06–8·21)	8·65 (8·25–9·04)
Germany	7·45 (6·86–8·02)	10·1 (9·60–10·5)
Greece	1·35 (1·24–1·46)	1·78 (1·71–1·86)
Iceland	0·0200 (0·0173–0·0230)	0·0217 (0·0202–0·0235)
Ireland	0·418 (0·377–0·461)	0·401 (0·372–0·430)
Israel	0·491 (0·423–0·565)	0·855 (0·797–0·916)
Italy	4·54 (4·16–4·92)	6·32 (6·02–6·65)
Luxembourg	0·0498 (0·0454–0·0544)	0·0659 (0·0619–0·0702)
Malta	0·0349 (0·0306–0·0393)	0·0474 (0·0442–0·0506)
Monaco	0·00327 (0·00265–0·00397)	0·00410 (0·00377–0·00445)
Netherlands	1·40 (1·29–1·51)	1·70 (1·61–1·79)
Norway	0·334 (0·290–0·383)	0·429 (0·398–0·464)
Portugal	0·859 (0·780–0·935)	1·30 (1·24–1·36)
San Marino	0·00238 (0·00201–0·00284)	0·00266 (0·00243–0·00289)
Spain	4·22 (3·88–4·59)	5·53 (5·25–5·82)
Sweden	0·581 (0·515–0·656)	0·510 (0·470–0·551)
Switzerland	0·837 (0·748–0·930)	1·05 (0·980–1·11)
United Kingdom	4·82 (4·38–5·28)	5·54 (5·23–5·85)
**Latin America and Caribbean**	**17·4 (16·3–18·4)**	**36·6 (35·7–37·5)**
Andean Latin America	1·13 (0·993–1·28)	3·21 (3·06–3·36)

Bolivia (Plurinational State of)	0·300 (0·242–0·366)	0·731 (0·670–0·793)
Ecuador	0·320 (0·266–0·386)	1·55 (1·47–1·63)
Peru	0·510 (0·408–0·629)	0·924 (0·826–1·04)
Caribbean	1·54 (1·38–1·70)	3·33 (3·20–3·47)
Antigua and Barbuda	0·00206 (0·00158–0·00263)	0·00446 (0·00396–0·00501)
Bahamas	0·00489 (0·00385–0·00617)	0·0167 (0·0152–0·0183)
Barbados	0·00498 (0·00389–0·00628)	0·0157 (0·0142–0·0173)
Belize	0·00637 (0·00484–0·00816)	0·0320 (0·0290–0·0352)
Bermuda	0·00225 (0·00184–0·00274)	0·00465 (0·00425–0·00511)
Cuba	0·767 (0·630–0·915)	1·58 (1·45–1·70)
Dominica	0·00159 (0·00123–0·00202)	0·00406 (0·00362–0·00452)
Dominican Republic	0·347 (0·288–0·408)	0·518 (0·475–0·567)
Grenada	0·00211 (0·00164–0·00268)	0·00749 (0·00673–0·00830)
Guyana	0·0122 (0·00956–0·0152)	0·0707 (0·0659–0·0760)
Haiti	0·118 (0·0907–0·153)	0·395 (0·358–0·431)
Jamaica	0·0745 (0·0622–0·0888)	0·218 (0·200–0·236)
Puerto Rico	0·125 (0·110–0·143)	0·207 (0·194–0·222)
Saint Kitts and Nevis	0·000706 (0·000534–0·000915)	0·00261 (0·00231–0·00292)
Saint Lucia	0·00368 (0·00290–0·00448)	0·0138 (0·0126–0·0152)
Saint Vincent and the Grenadines	0·00177 (0·00136–0·00228)	0·00909 (0·00832–0·00987)
Suriname	0·0195 (0·0156–0·0242)	0·0730 (0·0679–0·0782)
Trinidad and Tobago	0·0431 (0·0343–0·0529)	0·160 (0·148–0·174)
United States Virgin Islands	0·00228 (0·00183–0·00281)	0·00328 (0·00294–0·00365)
Central Latin America	8·41 (7·75–9·16)	20·4 (19·8–21·1)
Colombia	1·86 (1·61–2·14)	2·63 (2·42–2·84)
Costa Rica	0·136 (0·113–0·163)	0·270 (0·249–0·293)
El Salvador	0·0915 (0·0724–0·114)	0·363 (0·330–0·397)

Guatemala	0·283 (0·219–0·358)	1·23 (1·13–1·33)
Honduras	0·179 (0·141–0·225)	0·757 (0·695–0·818)
Mexico	4·37 (3·77–4·99)	12·2 (11·6–12·7)
Nicaragua	0·133 (0·101–0·172)	0·486 (0·437–0·540)
Panama	0·0687 (0·0537–0·0860)	0·188 (0·171–0·208)
Venezuela (Bolivarian Republic of)	1·30 (1·03–1·60)	2·35 (2·11–2·59)
Tropical Latin America	6·30 (5·56–7·04)	9·60 (9·01–10·2)
Brazil	6·10 (5·36–6·83)	9·00 (8·41–9·59)
Paraguay	0·199 (0·163–0·245)	0·598 (0·548–0·650)
**North Africa and Middle East**	**12·1 (11·3–12·9)**	**75·4 (74·3–76·6)**
North Africa and Middle East	12·1 (11·3–12·9)	75·4 (74·3–76·6)
Afghanistan	0·290 (0·222–0·377)	1·97 (1·80–2·15)
Algeria	0·259 (0·199–0·335)	5·08 (4·74–5·40)
Bahrain	0·0218 (0·0174–0·0275)	0·183 (0·163–0·203)
Egypt	0·341 (0·252–0·458)	15·0 (14·6–15·5)
Iran (Islamic Republic of)	1·55 (1·24–1·92)	8·66 (8·05–9·24)
Iraq	0·461 (0·359–0·587)	5·40 (5·04–5·72)
Jordan	0·450 (0·380–0·529)	2·37 (2·29–2·44)
Kuwait	0·0710 (0·0549–0·0898)	0·697 (0·646–0·747)
Lebanon	0·527 (0·462–0·592)	0·849 (0·808–0·891)
Libya	0·0377 (0·0280–0·0500)	1·11 (1·04–1·18)
Morocco	0·144 (0·107–0·192)	3·06 (2·83–3·28)
Oman	0·0205 (0·0154–0·0274)	0·427 (0·385–0·479)
Palestine	0·0574 (0·0439–0·0728)	0·640 (0·601–0·682)
Qatar	0·0148 (0·0112–0·0193)	0·448 (0·412–0·495)
Saudi Arabia	0·245 (0·185–0·315)	4·35 (4·05–4·64)
Sudan	0·250 (0·182–0·335)	2·44 (2·24–2·66)

Syrian Arab Republic	0·356 (0·273–0·466)	2·02 (1·87–2·17)
Tunisia	0·123 (0·0964–0·157)	2·02 (1·93–2·12)
Turkey	5·95 (5·37–6·58)	14·6 (14·0–15·1)
United Arab Emirates	0·0843 (0·0613–0·115)	1·25 (1·12–1·38)
Yemen	0·791 (0·646–0·958)	2·87 (2·64–3·09)
**South Asia**	**19·8 (17·1–23·0)**	**160 (153–166)**
South Asia	19·8 (17·1–23·0)	160 (153–166)
Bangladesh	1·16 (0·914–1·45)	25·0 (23·9–26·2)
Bhutan	0·0130 (0·0102–0·0165)	0·0438 (0·0396–0·0482)
India	14·9 (12·3–18·0)	116 (109–122)
Nepal	1·35 (1·18–1·55)	2·93 (2·77–3·07)
Pakistan	2·39 (1·89–2·95)	15·7 (14·7–16·7)
**Southeast Asia, East Asia, and Oceania**	**36·4 (32·3–41·0)**	**452 (443–460)**
East Asia	24·2 (20·2–28·8)	327 (319–335)
China	23·2 (19·2–27·8)	318 (310–326)
Democratic People's Republic of Korea	0·509 (0·405–0·640)	4·85 (4·60–5·08)
Taiwan (Province of China)	0·483 (0·383–0·596)	4·13 (3·91–4·35)
Oceania	0·746 (0·654–0·845)	1·81 (1·72–1·90)
American Samoa	0·00444 (0·00362–0·00541)	0·00816 (0·00756–0·00878)
Cook Islands	0·00166 (0·00141–0·00195)	0·00236 (0·00221–0·00250)
Fiji	0·0477 (0·0395–0·0571)	0·144 (0·136–0·154)
Guam	0·0119 (0·0106–0·0133)	0·0196 (0·0184–0·0207)
Kiribati	0·0141 (0·0126–0·0155)	0·0239 (0·0230–0·0247)
Marshall Islands	0·00195 (0·00155–0·00243)	0·00739 (0·00682–0·00796)
Micronesia (Federated States of)	0·0133 (0·0113–0·0151)	0·0233 (0·0223–0·0243)
Nauru	0·00146 (0·00130–0·00164)	0·00153 (0·00144–0·00163)
Niue	0·0000949 (0·0000788–0·000112)	0·000168 (0·000156–0·000181)

Northern Mariana Islands	0·00312 (0·00246–0·00393)	0·00749 (0·00692–0·00809)
Palau	0·000780 (0·000667–0·000903)	0·00264 (0·00248–0·00280)
Papua New Guinea	0·585 (0·495–0·685)	1·38 (1·30–1·47)
Samoa	0·00952 (0·00821–0·0110)	0·0274 (0·0258–0·0289)
Solomon Islands	0·0399 (0·0348–0·0451)	0·106 (0·101–0·111)
Tokelau	0·0000772 (0·0000606–0·0000961)	0·000189 (0·000174–0·000203)
Tonga	0·00457 (0·00388–0·00530)	0·0134 (0·0127–0·0142)
Tuvalu	0·000839 (0·000708–0·000986)	0·00200 (0·00188–0·00212)
Vanuatu	0·00585 (0·00451–0·00731)	0·0363 (0·0341–0·0385)
Southeast Asia	11·5 (10·7–12·4)	123 (121–125)
Cambodia	0·295 (0·240–0·356)	2·08 (1·97–2·18)
Indonesia	3·46 (2·86–4·18)	58·0 (56·6–59·2)
Lao People's Democratic Republic	0·162 (0·131–0·195)	1·17 (1·11–1·23)
Malaysia	0·361 (0·287–0·446)	5·08 (4·81–5·35)
Maldives	0·0103 (0·00813–0·0128)	0·120 (0·112–0·128)
Mauritius	0·0273 (0·0222–0·0334)	0·215 (0·205–0·226)
Myanmar	1·77 (1·48–2·06)	7·69 (7·34–8·03)
Philippines	3·03 (2·64–3·46)	16·1 (15·4–16·7)
Seychelles	0·00257 (0·00206–0·00313)	0·0171 (0·0159–0·0182)
Sri Lanka	0·161 (0·125–0·202)	2·43 (2·30–2·56)
Thailand	1·13 (0·934–1·36)	11·6 (11·2–12·0)
Timor-Leste	0·0200 (0·0160–0·0244)	0·263 (0·254–0·272)
Viet Nam	1·07 (0·849–1·33)	18·1 (17·5–18·8)
**Sub-Saharan Africa**	**8·74 (8·23–9·27)**	**50·4 (49·4–51·4)**
Central Sub-Saharan Africa	0·601 (0·502–0·712)	7·29 (6·86–7·77)
Angola	0·208 (0·161–0·267)	1·31 (1·20–1·43)
Central African Republic	0·0243 (0·0178–0·0327)	0·219 (0·193–0·247)

Congo	0·0312 (0·0227–0·0434)	0·341 (0·311–0·375)
Democratic Republic of the Congo	0·302 (0·223–0·397)	5·17 (4·75–5·62)
Equatorial Guinea	0·0116 (0·00783–0·0173)	0·126 (0·112–0·139)
Gabon	0·0234 (0·0176–0·0305)	0·128 (0·118–0·139)
Eastern Sub-Saharan Africa	3·32 (3·07–3·61)	18·4 (17·9–18·9)
Burundi	0·119 (0·0940–0·150)	0·478 (0·441–0·521)
Comoros	0·00738 (0·00555–0·00943)	0·0543 (0·0501–0·0586)
Djibouti	0·0196 (0·0151–0·0251)	0·173 (0·161–0·184)
Eritrea	0·0145 (0·0105–0·0199)	0·255 (0·226–0·288)
Ethiopia	0·370 (0·278–0·492)	2·83 (2·58–3·10)
Kenya	0·371 (0·288–0·483)	2·72 (2·52–2·93)
Madagascar	0·211 (0·160–0·278)	2·05 (1·87–2·23)
Malawi	0·145 (0·113–0·185)	1·02 (0·951–1·09)
Mozambique	0·467 (0·361–0·595)	1·56 (1·41–1·73)
Rwanda	0·255 (0·209–0·305)	0·658 (0·610–0·710)
Somalia	0·134 (0·0996–0·177)	1·02 (0·891–1·14)
South Sudan	0·0629 (0·0470–0·0812)	0·469 (0·419–0·523)
Uganda	0·413 (0·326–0·525)	1·35 (1·24–1·46)
United Republic of Tanzania	0·446 (0·347–0·560)	2·48 (2·30–2·66)
Zambia	0·284 (0·224–0·360)	1·27 (1·20–1·35)
Southern Sub-Saharan Africa	2·48 (2·12–2·88)	9·39 (9·02–9·74)
Botswana	0·0684 (0·0566–0·0802)	0·295 (0·277–0·312)
Eswatini	0·00917 (0·00723–0·0118)	0·0513 (0·0462–0·0570)
Lesotho	0·0116 (0·00886–0·0152)	0·299 (0·283–0·314)
Namibia	0·0787 (0·0647–0·0944)	0·167 (0·155–0·180)
South Africa	2·17 (1·82–2·56)	7·25 (6·90–7·56)
Zimbabwe	0·141 (0·109–0·179)	1·32 (1·25–1·40)

Western Sub-Saharan Africa	2·34 (2·11–2·59)	15·4 (14·8–15·9)
Benin	0·0565 (0·0423–0·0726)	0·318 (0·287–0·349)
Burkina Faso	0·101 (0·0747–0·137)	1·03 (0·940–1·12)
Cabo Verde	0·00588 (0·00460–0·00753)	0·0195 (0·0173–0·0219)
Cameroon	0·128 (0·0938–0·169)	1·19 (1·07–1·31)
Chad	0·115 (0·0860–0·157)	0·599 (0·541–0·659)
Côte d'Ivoire	0·271 (0·202–0·358)	1·89 (1·73–2·07)
Gambia	0·00898 (0·00652–0·0124)	0·151 (0·139–0·163)
Ghana	0·219 (0·163–0·295)	0·921 (0·818–1·03)
Guinea	0·0694 (0·0510–0·0908)	0·920 (0·836–1·01)
Guinea-Bissau	0·00584 (0·00422–0·00775)	0·0437 (0·0378–0·0500)
Liberia	0·0381 (0·0288–0·0495)	0·191 (0·174–0·210)
Mali	0·114 (0·0844–0·149)	1·23 (1·13–1·33)
Mauritania	0·111 (0·0864–0·140)	0·351 (0·320–0·382)
Niger	0·104 (0·0751–0·144)	0·823 (0·735–0·918)
Nigeria	0·664 (0·489–0·892)	4·05 (3·61–4·51)
São Tomé and Príncipe	0·000945 (0·000724–0·00122)	0·00480 (0·00429–0·00539)
Senegal	0·0697 (0·0520–0·0913)	0·638 (0·589–0·694)
Sierra Leone	0·204 (0·162–0·255)	0·716 (0·671–0·767)
Togo	0·0497 (0·0380–0·0640)	0·299 (0·272–0·328)

Supplemental Table S3. Relative percent change in number of smokers and age-standardized smoking prevalence, between 1990 and 2019, for 204 countries and territories.

Location	Percent Change in Number of Smokers, 1990-2019	Percent Change in Age-Standardized Smoking Prevalence, 1990-2019
**Global**	**15·4 (13·8–16·9)**	**-29·6 (-30·5–-28·6)**
**Central Europe, Eastern Europe, and Central Asia**	**-11·1 (-12·9–-8·88)**	**-16·6 (-18·4–-14·6)**
Central Asia	39·6 (34·9–44·1)	-11·0 (-13·9–-8·34)
Armenia	-7·62 (-12·7–-2·59)	-8·48 (-13·4–-3·68)
Azerbaijan	74·9 (62·3–88·6)	3·06 (-3·92–10·7)
Georgia	-26·0 (-30·9–-20·8)	9·15 (1·98–16·8)
Kazakhstan	6·31 (-0·478–13·5)	-12·4 (-17·8–-6·50)
Kyrgyzstan	59·8 (50·2–70·5)	-1·67 (-7·30–4·20)
Mongolia	130 (114–148)	12·3 (5·41–20·1)
Tajikistan	20·7 (6·38–35·8)	-45·1 (-50·9–-39·0)
Turkmenistan	-3·89 (-14·1–7·78)	-43·0 (-48·8–-36·7)
Uzbekistan	191 (154–230)	37·0 (20·6–53·4)
Central Europe	-20·4 (-22·3–-18·5)	-18·3 (-20·3–-16·2)
Albania	34·9 (23·8–46·5)	30·7 (20·4–41·0)
Bosnia and Herzegovina	-2·21 (-10·7–7·52)	25·9 (14·9–38·0)
Bulgaria	-36·0 (-41·0–-30·8)	-18·5 (-25·0–-11·7)
Croatia	-25·9 (-31·0–-20·5)	-14·6 (-20·6–-8·48)
Czechia	-18·4 (-23·7–-12·9)	-20·7 (-26·1–-15·2)
Hungary	-24·3 (-29·4–-19·1)	-20·1 (-25·7–-14·2)
Montenegro	5·45 (-3·91–15·4)	-4·46 (-12·8–4·30)
North Macedonia	21·3 (11·1–32·1)	0·616 (-7·93–9·66)
Poland	-25·5 (-29·1–-21·9)	-31·8 (-34·9–-28·4)

Romania	-24·4<br>(-29·6–-19·6)	-9·82<br>(-15·8–-4·16)
Serbia	-1·55<br>(-9·43–6·52)	5·15<br>(-3·40–13·9)
Slovakia	-12·0<br>(-19·9–-3·33)	-19·9<br>(-27·3–-12·1)
Slovenia	-0·404<br>(-10·3–9·60)	-2·63<br>(-12·1–7·22)
Eastern Europe	-13·5<br>(-16·5–-10·1)	-11·5<br>(-14·7–-7·89)
Belarus	-0·832<br>(-9·16–7·88)	4·98<br>(-4·07–14·4)
Estonia	-32·8<br>(-36·3–-28·6)	-21·9<br>(-26·5–-17·1)
Latvia	-34·5<br>(-38·9–-30·2)	-8·59<br>(-14·5–-2·39)
Lithuania	-27·7<br>(-32·2–-23·0)	-5·28<br>(-11·6–1·21)
Republic of Moldova	-1·02<br>(-9·29–8·09)	7·55<br>(-1·49–17·5)
Russian Federation	-6·56<br>(-11·0–-1·60)	-8·96<br>(-13·4–-4·05)
Ukraine	-32·5<br>(-36·6–-28·3)	-22·9<br>(-27·8–-17·8)
**High-income**	**-17·6**<br>**(-18·9–-16·2)**	**-30·6**<br>**(-31·8–-29·4)**
Australasia	-23·4<br>(-28·6–-17·9)	-46·3<br>(-50·1–-42·4)
Australia	-23·4<br>(-29·6–-16·8)	-47·5<br>(-51·8–-43·1)
New Zealand	-23·7<br>(-27·5–-19·8)	-40·2<br>(-43·1–-37·0)
High-income Asia Pacific	-27·7<br>(-30·5–-24·7)	-33·8<br>(-36·4–-30·8)
Brunei Darussalam	34·2<br>(20·1–50·1)	-34·0<br>(-40·3–-26·2)
Japan	-38·4<br>(-41·6–-35·1)	-37·5<br>(-41·2–-33·7)
Republic of Korea	4·65<br>(-2·84–13·1)	-21·6<br>(-26·9–-15·3)
Singapore	37·1<br>(20·9–54·8)	-30·9<br>(-38·8–-22·0)
High-income North America	-15·1<br>(-19·0–-11·0)	-35·5<br>(-38·5–-32·5)
Canada	-29·7<br>(-35·6–-23·3)	-47·4<br>(-52·0–-42·4)
Greenland	-7·19<br>(-16·3–2·37)	-8·45<br>(-16·9–0·357)
United States of America	-13·1<br>(-17·4–-8·62)	-33·9<br>(-37·1–-30·4)
Southern Latin America	19·6<br>(11·3–28·9)	-18·4<br>(-24·1–-12·1)
Argentina	19·6<br>(7·31–33·9)	-19·7<br>(-27·9–-10·2)

Chile	25·7 (13·5–39·0)	-12·8 (-21·1–-3·29)
Uruguay	-9·24 (-17·4–0·0904)	-21·0 (-28·6–-12·8)
Western Europe	-18·3 (-19·7–-16·8)	-26·5 (-27·8–-25·1)
Andorra	14·6 (2·05–28·3)	-23·6 (-31·8–-14·1)
Austria	1·32 (-4·47–7·77)	-12·0 (-17·3–-6·21)
Belgium	-26·4 (-30·3–-22·4)	-34·6 (-38·3–-30·9)
Cyprus	72·7 (56·4–88·7)	-8·10 (-17·0–0·219)
Denmark	-43·6 (-46·4–-40·8)	-49·3 (-52·2–-46·4)
Finland	-24·9 (-29·4–-20·4)	-29·0 (-33·6–-24·5)
France	-1·82 (-6·13–2·71)	-10·3 (-14·3–-5·95)
Germany	-16·4 (-20·3–-12·2)	-18·9 (-23·0–-14·9)
Greece	-8·80 (-12·9–-4·56)	-11·4 (-15·3–-7·30)
Iceland	-28·2 (-34·6–-20·8)	-49·7 (-54·1–-44·5)
Ireland	-6·02 (-11·9–-0·0343)	-33·6 (-38·1–-29·2)
Israel	23·2 (11·5–36·1)	-35·8 (-42·2–-29·1)
Italy	-27·6 (-31·2–-23·6)	-29·1 (-32·8–-25·3)
Luxembourg	11·0 (3·41–18·6)	-29·8 (-34·6–-24·7)
Malta	-13·5 (-20·6–-5·46)	-27·6 (-34·0–-20·7)
Monaco	-9·45 (-20·6–2·25)	-22·5 (-32·4–-12·0)
Netherlands	-37·0 (-40·1–-33·9)	-44·3 (-47·2–-41·3)
Norway	-41·1 (-46·2–-35·8)	-53·5 (-57·6–-49·1)
Portugal	-1·03 (-6·39–4·34)	-3·46 (-8·97–1·93)
San Marino	-15·1 (-25·5–-3·45)	-38·9 (-46·6–-30·1)
Spain	-19·5 (-23·3–-15·1)	-32·9 (-36·2–-29·3)
Sweden	-33·6 (-39·2–-27·2)	-45·9 (-50·8–-40·2)
Switzerland	1·19 (-5·08–7·64)	-21·2 (-26·3–-15·9)
United Kingdom	-29·4 (-33·2–-25·4)	-38·8 (-42·1–-35·2)

**Latin America and Caribbean**	**-19·8 (-22·5–-16·9)**	**-55·0 (-56·5–-53·4)**
Andean Latin America	63·9 (50·4–77·8)	-16·8 (-23·0–-10·6)
Bolivia (Plurinational State of)	111 (82·3–144)	-4·95 (-17·1–8·12)
Ecuador	95·2 (75·5–115)	-10·4 (-18·7–-1·56)
Peru	20·2 (0·891–42·3)	-31·4 (-41·5–-19·6)
Caribbean	4·13 (-2·09–11·3)	-33·0 (-37·0–-28·6)
Antigua and Barbuda	136 (101–174)	31·5 (12·6–52·0)
Bahamas	60·4 (38·5–84·9)	-3·38 (-15·9–11·1)
Barbados	4·18 (-9·43–20·5)	-12·1 (-24·1–1·88)
Belize	182 (143–222)	0·519 (-11·7–13·3)
Bermuda	6·24 (-7·22–23·0)	-1·43 (-13·8–14·4)
Cuba	-13·4 (-22·4–-2·85)	-28·9 (-36·0–-20·9)
Dominica	3·81 (-11·1–20·1)	-3·09 (-16·6–12·0)
Dominican Republic	74·1 (51·0–98·7)	-13·1 (-24·3–-0·792)
Grenada	74·1 (50·2–101)	9·20 (-6·10–24·8)
Guyana	19·5 (5·09–35·1)	3·04 (-8·07–15·9)
Haiti	21·1 (4·68–38·3)	-47·5 (-54·4–-40·5)
Jamaica	12·0 (0·234–25·0)	-24·9 (-32·4–-17·1)
Puerto Rico	-12·5 (-21·0–-3·15)	-16·8 (-25·2–-7·63)
Saint Kitts and Nevis	97·1 (66·9–132)	2·58 (-12·6–20·6)
Saint Lucia	49·7 (31·1–71·0)	-11·8 (-22·2–0·375)
Saint Vincent and the Grenadines	21·6 (7·16–37·8)	-2·28 (-13·2–9·88)
Suriname	33·7 (19·4–48·9)	-20·6 (-28·4–-11·9)
Trinidad and Tobago	18·9 (5·13–33·4)	-11·2 (-21·4–-0·848)
United States Virgin Islands	-16·0 (-29·1–-1·87)	-24·1 (-36·0–-10·9)
Central Latin America	7·15 (1·85–12·7)	-40·9 (-43·7–-38·2)
Colombia	-11·1 (-22·3–0·794)	-47·1 (-53·4–-40·4)

Costa Rica	-2·53 (-14·5–11·4)	-47·4 (-53·6–-40·5)
El Salvador	79·6 (54·5–107)	30·5 (13·2–49·1)
Guatemala	142 (112–175)	-15·2 (-24·7–-4·86)
Honduras	110 (86·2–136)	-20·2 (-28·4–-11·3)
Mexico	0·600 (-6·45–8·37)	-43·0 (-46·4–-39·0)
Nicaragua	67·0 (45·5–91·6)	-21·7 (-31·2–-11·3)
Panama	18·4 (2·71–36·1)	-38·8 (-46·2–-30·1)
Venezuela (Bolivarian Republic of)	18·0 (3·07–34·4)	-34·7 (-42·5–-26·1)
Tropical Latin America	-51·9 (-55·1–-48·5)	-72·7 (-74·5–-70·7)
Brazil	-53·4 (-56·6–-49·8)	-73·4 (-75·2–-71·4)
Paraguay	28·2 (14·1–43·4)	-40·2 (-46·3–-33·7)
**North Africa and Middle East**	**104 (98·1–111)**	**-8·96 (-11·6–-6·41)**
North Africa and Middle East	104 (98·1–111)	-8·96 (-11·6–-6·41)
Afghanistan	1060 (900–1240)	221 (177–268)
Algeria	102 (79·8–126)	-7·80 (-16·8–1·31)
Bahrain	257 (215–302)	-3·10 (-12·7–7·14)
Egypt	136 (117–155)	15·6 (7·20–24·4)
Iran (Islamic Republic of)	127 (102–155)	2·13 (-8·98–13·8)
Iraq	152 (130–175)	-19·0 (-25·3–-12·4)
Jordan	345 (310–385)	13·7 (5·87–21·9)
Kuwait	125 (103–151)	-21·9 (-28·7–-14·7)
Lebanon	151 (127–177)	31·4 (19·5–44·4)
Libya	131 (109–155)	-5·94 (-14·0–2·82)
Morocco	19·6 (7·75–32·7)	-32·4 (-38·6–-25·4)
Oman	168 (134–206)	-21·4 (-29·6–-12·6)
Palestine	187 (164–210)	-4·40 (-10·9–2·35)
Qatar	635 (535–753)	1·12 (-10·0–13·9)

Saudi Arabia	344<br>(301–393)	37·8<br>(25·6–51·4)
Sudan	101<br>(76·7–129)	-11·9<br>(-21·9–-0·964)
Syrian Arab Republic	20·8<br>(9·49–32·2)	-23·3<br>(-29·9–-16·6)
Tunisia	49·7<br>(39·4–61·0)	-13·5<br>(-18·9–-7·50)
Turkey	45·8<br>(36·5–54·7)	-13·5<br>(-19·0–-8·36)
United Arab Emirates	405<br>(334–482)	-19·6<br>(-29·1–-10·1)
Yemen	183<br>(149–216)	-2·90<br>(-13·1–7·98)
**South Asia**	**23·7**<br>**(17·8–29·7)**	**-39·1**<br>**(-41·9–-36·3)**
South Asia	23·7<br>(17·8–29·7)	-39·1<br>(-41·9–-36·3)
Bangladesh	66·0<br>(55·7–76·4)	-18·0<br>(-22·8–-13·3)
Bhutan	27·7<br>(9·33–49·6)	-11·7<br>(-22·6–0·661)
India	17·3<br>(10·3–24·6)	-41·5<br>(-44·8–-38·0)
Nepal	9·67<br>(0·697–19·7)	-45·1<br>(-49·4–-40·2)
Pakistan	31·2<br>(19·4–44·0)	-41·3<br>(-46·1–-36·2)
**Southeast Asia, East Asia, and Oceania**	**30·0**<br>**(26·5–33·4)**	**-17·5**<br>**(-19·7–-15·4)**
East Asia	22·3<br>(18·0–26·4)	-19·3<br>(-22·2–-16·5)
China	22·2<br>(17·8–26·5)	-19·5<br>(-22·5–-16·6)
Democratic People's Republic of Korea	50·4<br>(38·6–62·5)	0·415<br>(-7·40–8·63)
Taiwan (Province of China)	7·49<br>(-1·00–16·7)	-21·7<br>(-27·9–-15·3)
Oceania	86·9<br>(73·2–102)	-16·1<br>(-21·8–-9·79)
American Samoa	20·8<br>(7·25–36·1)	-5·28<br>(-15·3–5·58)
Cook Islands	-5·03<br>(-15·1–6·36)	-8·58<br>(-18·4–2·20)
Fiji	13·7<br>(2·83–24·7)	-15·7<br>(-23·5–-7·50)
Guam	5·79<br>(-4·32–16·4)	-13·3<br>(-21·1–-4·79)
Kiribati	79·8<br>(64·7–97·1)	3·93<br>(-3·85–13·2)
Marshall Islands	71·4<br>(51·9–93·4)	7·07<br>(-4·34–19·7)
Micronesia (Federated States of)	32·5<br>(21·2–44·2)	6·29<br>(-1·91–14·6)

Nauru	8·10 (-2·79–19·4)	-5·92 (-14·3–2·67)
Niue	-20·0 (-29·5–-9·28)	-5·64 (-17·2–7·29)
Northern Mariana Islands	-16·2 (-25·4–-6·25)	-17·0 (-25·1–-7·77)
Palau	25·4 (12·3–40·2)	-1·41 (-11·6–9·38)
Papua New Guinea	109 (88·6–131)	-19·0 (-26·2–-10·9)
Samoa	28·3 (14·6–41·7)	-11·8 (-19·8–-3·52)
Solomon Islands	124 (103–146)	2·48 (-6·37–11·0)
Tokelau	-4·72 (-15·6–6·82)	-1·33 (-12·0–10·5)
Tonga	-2·61 (-10·2–5·00)	-18·2 (-23·4–-12·3)
Tuvalu	51·9 (36·1–70·2)	10·9 (-0·348–23·8)
Vanuatu	84·0 (65·6–104)	-14·6 (-22·0–-6·17)
Southeast Asia	54·7 (51·1–58·3)	-13·3 (-15·2–-11·4)
Cambodia	91·5 (78·2–105)	-15·7 (-21·0–-10·3)
Indonesia	84·4 (77·0–91·3)	8·86 (4·65–12·7)
Lao People's Democratic Republic	152 (129–172)	14·6 (4·87–23·1)
Malaysia	71·4 (57·5–85·9)	-20·0 (-26·0–-13·5)
Maldives	305 (270–341)	4·94 (-3·03–13·3)
Mauritius	16·2 (7·99–26·1)	-12·2 (-18·4–-5·29)
Myanmar	4·37 (-3·07–12·3)	-37·4 (-41·7–-32·9)
Philippines	45·7 (36·3–56·2)	-30·0 (-34·4–-25·4)
Seychelles	52·7 (37·1–68·5)	-5·72 (-15·1–3·20)
Sri Lanka	-0·651 (-8·12–8·10)	-34·5 (-39·2–-29·0)
Thailand	10·3 (4·28–16·8)	-27·7 (-31·5–-23·6)
Timor-Leste	78·1 (67·6–88·9)	-0·858 (-5·85–4·42)
Viet Nam	62·0 (53·4–70·3)	-17·3 (-21·4–-13·1)
**Sub-Saharan Africa**	**74·6 (69·9–79·1)**	**-25·9 (-27·7–-24·1)**
Central Sub-Saharan Africa	140 (119–162)	-5·17 (-12·7–3·16)

Angola	138 (110–170)	-18·1 (-26·8–-8·44)
Central African Republic	49·9 (28·8–73·0)	-25·7 (-35·8–-15·2)
Congo	233 (190–283)	31·6 (16·3–50·1)
Democratic Republic of the Congo	140 (111–171)	-2·41 (-13·3–9·57)
Equatorial Guinea	344 (279–415)	11·8 (-1·40–26·8)
Gabon	139 (108–175)	14·2 (0·260–29·8)
Eastern Sub-Saharan Africa	82·2 (75·1–90·0)	-22·9 (-25·6–-19·9)
Burundi	44·6 (26·6–63·5)	-32·0 (-39·8–-23·8)
Comoros	71·7 (52·0–93·5)	-13·9 (-22·7–-3·92)
Djibouti	263 (224–303)	14·5 (4·66–25·6)
Eritrea	109 (75·8–146)	-19·3 (-31·2–-6·30)
Ethiopia	98·2 (71·3–128)	-14·0 (-24·5–-1·64)
Kenya	69·1 (50·5–87·3)	-36·1 (-42·2–-29·6)
Madagascar	56·7 (39·4–73·3)	-38·1 (-44·2–-31·9)
Malawi	98·8 (77·5–122)	-4·48 (-14·0–5·88)
Mozambique	108 (81·4–136)	-6·04 (-17·3–6·14)
Rwanda	107 (81·6–133)	-2·34 (-13·6–9·44)
Somalia	142 (109–179)	-16·3 (-26·1–-5·13)
South Sudan	20·1 (4·80–36·9)	-25·0 (-33·6–-16·0)
Uganda	92·8 (69·8–120)	-21·6 (-30·0–-12·5)
United Republic of Tanzania	47·8 (31·6–65·3)	-37·4 (-44·1–-30·5)
Zambia	179 (147–212)	2·20 (-7·50–12·9)
Southern Sub-Saharan Africa	36·1 (28·3–44·8)	-25·6 (-29·7–-21·4)
Botswana	122 (101–146)	-7·14 (-15·3–1·46)
Eswatini	29·9 (13·9–48·0)	-28·6 (-36·9–-19·6)
Lesotho	88·4 (69·9–107)	30·0 (17·3–41·9)
Namibia	53·1 (36·1–70·8)	-23·5 (-31·8–-14·7)

South Africa	28·6 (19·6–37·9)	-29·4 (-34·1–-24·5)
Zimbabwe	72·6 (54·9–90·6)	-3·25 (-12·0–5·32)
Western Sub-Saharan Africa	78·0 (69·3–87·1)	-27·9 (-31·2–-24·4)
Benin	65·6 (44·3–88·7)	-41·3 (-48·1–-34·1)
Burkina Faso	93·4 (68·4–120)	-26·6 (-35·3–-17·2)
Cabo Verde	41·6 (21·8–65·0)	-37·8 (-46·1–-27·8)
Cameroon	113 (85·3–142)	-31·8 (-39·8–-22·8)
Chad	83·4 (60·5–109)	-28·4 (-36·6–-19·5)
Côte d'Ivoire	163 (128–203)	8·93 (-4·08–23·2)
Gambia	68·7 (49·8–88·7)	-35·3 (-41·8–-28·6)
Ghana	134 (99·9–173)	-8·79 (-20·9–4·42)
Guinea	86·2 (65·2–109)	-9·23 (-18·9–1·16)
Guinea-Bissau	147 (106–193)	15·8 (-2·12–34·7)
Liberia	153 (118–190)	-9·31 (-20·9–3·37)
Mali	225 (188–268)	31·9 (18·2–46·8)
Mauritania	66·6 (48·0–87·9)	-20·2 (-28·8–-10·9)
Niger	209 (162–267)	12·0 (-3·79–29·9)
Nigeria	26·2 (9·52–44·1)	-46·3 (-52·9–-39·2)
São Tomé and Príncipe	171 (134–209)	25·8 (8·91–43·9)
Senegal	10·0 (-2·91–24·8)	-50·9 (-56·0–-44·6)
Sierra Leone	101 (77·5–127)	-15·3 (-24·1–-5·37)
Togo	54·1 (34·1–75·8)	-42·1 (-48·9–-34·9)

Supplemental Table S4. Cigarette-equivalents per person aged 15 years and older in 1990 and 2019, for 204 countries and territories.

Location	Cigarettes Per Capita, 1990	Cigarettes Per Capita, 2019
**Global**	**1480 (1440–1530)**	**1110 (1070–1160)**
**Central Europe, Eastern Europe, and Central Asia**	**1950 (1850–2050)**	**1770 (1630–1920)**
Central Asia	1240 (1160–1340)	1150 (1070–1230)
Armenia	2020 (1800–2260)	1910 (1540–2350)
Azerbaijan	1290 (1080–1530)	1640 (1430–1870)
Georgia	1580 (1340–1850)	1770 (1460–2100)
Kazakhstan	1540 (1280–1860)	1420 (1130–1750)
Kyrgyzstan	1880 (1670–2120)	1920 (1570–2300)
Mongolia	912 (816–1020)	1140 (950–1350)
Tajikistan	1570 (1340–1860)	1150 (903–1450)
Turkmenistan	2090 (1870–2320)	1530 (1270–1790)
Uzbekistan	438 (364–530)	518 (440–599)
Central Europe	2530 (2380–2720)	1850 (1710–2000)
Albania	1890 (1620–2220)	1940 (1620–2310)
Bosnia and Herzegovina	1880 (1640–2120)	2330 (2010–2710)
Bulgaria	3800 (3330–4280)	2500 (2140–2920)
Croatia	2720 (2430–3050)	2020 (1690–2420)
Czechia	2250 (1970–2570)	1920 (1580–2340)
Hungary	2900 (2560–3280)	1920 (1590–2280)
Montenegro	2840 (2370–3340)	2910 (2370–3480)
North Macedonia	2770 (2310–3330)	2540 (2200–2910)
Poland	2910 (2470–3470)	1730 (1400–2110)
Romania	1890 (1630–2200)	1490 (1210–1780)
Serbia	2070 (1820–2360)	2130 (1750–2560)

Slovakia	1770<br>(1550–2030)	1400<br>(1190–1640)
Slovenia	2800<br>(2570–3040)	2450<br>(2100–2810)
Eastern Europe	1840<br>(1690–2000)	2000<br>(1740–2280)
Belarus	2150<br>(1780–2560)	2080<br>(1730–2540)
Estonia	1830<br>(1640–2040)	1830<br>(1550–2140)
Latvia	2130<br>(1810–2510)	1570<br>(1260–1960)
Lithuania	1640<br>(1410–1900)	1370<br>(1140–1620)
Republic of Moldova	2090<br>(1670–2590)	2220<br>(1760–2780)
Russian Federation	1790<br>(1600–2010)	2120<br>(1770–2500)
Ukraine	1890<br>(1630–2210)	1640<br>(1320–2030)
**High-income**	**2350**<br>**(2250–2450)**	**1470**<br>**(1390–1550)**
Australasia	1650<br>(1560–1750)	1070<br>(940–1210)
Australia	1670<br>(1570–1790)	1100<br>(947–1270)
New Zealand	1570<br>(1460–1690)	930<br>(769–1110)
High-income Asia Pacific	2150<br>(1930–2390)	1500<br>(1310–1720)
Brunei Darussalam	2150<br>(1780–2590)	1520<br>(1190–1930)
Japan	2150<br>(1860–2440)	1520<br>(1260–1800)
Republic of Korea	2220<br>(1910–2600)	1570<br>(1280–1890)
Singapore	921<br>(769–1100)	548<br>(471–636)
High-income North America	2800<br>(2560–3050)	1440<br>(1260–1620)
Canada	2510<br>(2230–2800)	1450<br>(1230–1700)
Greenland	2400<br>(1810–3120)	1840<br>(1410–2360)
United States of America	2840<br>(2570–3110)	1440<br>(1240–1630)
Southern Latin America	1440<br>(1340–1550)	1180<br>(1050–1340)
Argentina	1590<br>(1460–1750)	1320<br>(1140–1540)
Chile	1040<br>(907–1200)	794<br>(666–944)
Uruguay	1510<br>(1330–1700)	1510<br>(1270–1790)

Western Europe	2260 (2140–2400)	1540 (1430–1660)
Andorra	2150 (1640–2760)	1800 (1380–2290)
Austria	1720 (1630–1820)	2270 (1970–2580)
Belgium	2630 (2390–2850)	1960 (1710–2210)
Cyprus	2420 (2200–2670)	2230 (1840–2730)
Denmark	2590 (2260–2950)	1970 (1640–2360)
Finland	1380 (1280–1510)	1230 (1040–1450)
France	1870 (1580–2170)	1320 (1070–1610)
Germany	2360 (2000–2740)	1650 (1340–2010)
Greece	3470 (2970–4030)	2370 (1880–2960)
Iceland	2690 (2370–3070)	1820 (1500–2200)
Ireland	2160 (1910–2420)	1350 (1110–1620)
Israel	1820 (1570–2090)	1250 (1030–1520)
Italy	2040 (1730–2390)	1520 (1220–1830)
Luxembourg	2140 (1650–2780)	1790 (1370–2280)
Malta	2780 (2470–3100)	2050 (1700–2480)
Monaco	2140 (1650–2720)	1770 (1340–2300)
Netherlands	2440 (2040–2920)	1450 (1150–1760)
Norway	1490 (1380–1610)	1030 (911–1170)
Portugal	1750 (1460–2070)	1250 (993–1530)
San Marino	2160 (1690–2750)	1790 (1380–2320)
Spain	3040 (2570–3590)	1910 (1550–2320)
Sweden	1600 (1420–1790)	1410 (1150–1710)
Switzerland	2770 (2320–3270)	2020 (1620–2490)
United Kingdom	2230 (2000–2510)	1190 (990–1420)
**Latin America and Caribbean**	**1080 (1010–1150)**	**696 (626–783)**
Andean Latin America	337 (309–369)	257 (228–290)

Bolivia (Plurinational State of)	392 (352–438)	268 (231–309)
Ecuador	510 (433–594)	321 (256–400)
Peru	241 (211–279)	221 (180–267)
Caribbean	1220 (1150–1290)	884 (793–985)
Antigua and Barbuda	438 (366–516)	508 (407–619)
Bahamas	535 (438–651)	521 (410–670)
Barbados	681 (619–748)	592 (484–717)
Belize	575 (502–650)	563 (448–700)
Bermuda	754 (609–914)	761 (607–916)
Cuba	2420 (2220–2630)	1860 (1540–2230)
Dominica	493 (437–555)	457 (370–554)
Dominican Republic	766 (648–897)	773 (619–967)
Grenada	443 (378–522)	427 (337–536)
Guyana	599 (550–657)	564 (445–708)
Haiti	281 (253–311)	200 (164–240)
Jamaica	1040 (957–1130)	943 (797–1110)
Puerto Rico	683 (519–869)	648 (496–826)
Saint Kitts and Nevis	383 (338–435)	390 (313–472)
Saint Lucia	770 (681–865)	654 (543–778)
Saint Vincent and the Grenadines	468 (403–545)	522 (406–670)
Suriname	1540 (1410–1670)	1220 (979–1490)
Trinidad and Tobago	1050 (969–1150)	808 (655–980)
United States Virgin Islands	682 (520–868)	648 (498–822)
Central Latin America	803 (737–870)	506 (458–554)
Colombia	870 (764–990)	543 (462–636)
Costa Rica	798 (722–886)	536 (463–615)
El Salvador	464 (411–521)	528 (427–639)

Guatemala	501 (413–606)	452 (360–561)
Honduras	919 (835–1010)	784 (636–961)
Mexico	775 (663–887)	426 (349–508)
Nicaragua	772 (686–868)	616 (501–750)
Panama	607 (551–667)	547 (428–677)
Venezuela (Bolivarian Republic of)	1040 (919–1180)	695 (566–843)
Tropical Latin America	1520 (1360–1690)	994 (823–1210)
Brazil	1530 (1370–1700)	993 (819–1210)
Paraguay	1200 (987–1450)	1050 (820–1330)
**North Africa and Middle East**	**1400 (1320–1470)**	**1130 (1060–1210)**
North Africa and Middle East	1400 (1320–1470)	1130 (1060–1210)
Afghanistan	274 (233–321)	444 (355–546)
Algeria	1260 (1150–1380)	1130 (988–1300)
Bahrain	1240 (981–1600)	1180 (909–1490)
Egypt	1260 (1090–1440)	1290 (1080–1550)
Iran (Islamic Republic of)	816 (720–930)	792 (642–974)
Iraq	1890 (1640–2170)	1450 (1170–1770)
Jordan	1970 (1770–2190)	1450 (1190–1730)
Kuwait	2230 (1910–2580)	1920 (1560–2350)
Lebanon	2040 (1720–2440)	2210 (1730–2740)
Libya	1240 (1140–1350)	1020 (828–1270)
Morocco	944 (839–1060)	631 (536–739)
Oman	1160 (1050–1270)	1140 (899–1430)
Palestine	1230 (957–1560)	1170 (912–1520)
Qatar	1240 (954–1580)	1170 (906–1470)
Saudi Arabia	1230 (956–1560)	1170 (903–1490)
Sudan	171 (142–200)	227 (181–280)

Syrian Arab Republic	1550 (1400–1730)	1230 (992–1530)
Tunisia	1820 (1560–2140)	1510 (1240–1820)
Turkey	2670 (2360–3040)	1770 (1440–2150)
United Arab Emirates	1400 (1250–1540)	1290 (1020–1620)
Yemen	1540 (1310–1790)	1390 (1130–1710)
**South Asia**	**713 (663–767)**	**558 (490–634)**
South Asia	713 (663–767)	558 (490–634)
Bangladesh	559 (517–601)	446 (374–523)
Bhutan	721 (555–916)	630 (482–801)
India	699 (638–768)	559 (473–656)
Nepal	621 (562–690)	478 (396–570)
Pakistan	977 (928–1030)	642 (558–734)
**Southeast Asia, East Asia, and Oceania**	**1830 (1690–1960)**	**1780 (1640–1930)**
East Asia	2090 (1910–2270)	2130 (1930–2350)
China	2100 (1920–2280)	2150 (1950–2370)
Democratic People's Republic of Korea	1430 (1240–1630)	1350 (1060–1680)
Taiwan (Province of China)	2230 (1890–2610)	1630 (1320–2020)
Oceania	982 (828–1170)	950 (784–1140)
American Samoa	984 (751–1260)	963 (734–1230)
Cook Islands	990 (773–1260)	958 (731–1220)
Fiji	790 (718–871)	712 (600–844)
Guam	990 (763–1240)	960 (726–1230)
Kiribati	1070 (968–1200)	1380 (1110–1670)
Marshall Islands	989 (758–1270)	966 (753–1240)
Micronesia (Federated States of)	990 (763–1280)	959 (732–1230)
Nauru	986 (754–1260)	963 (732–1220)
Niue	994 (763–1270)	962 (749–1210)

Northern Mariana Islands	987<br>(761–1260)	960<br>(736–1240)
Palau	1000<br>(770–1280)	965<br>(745–1230)
Papua New Guinea	990<br>(759–1270)	954<br>(746–1210)
Samoa	1880<br>(1640–2130)	1810<br>(1430–2210)
Solomon Islands	1100<br>(1000–1200)	1070<br>(904–1250)
Tokelau	990<br>(766–1270)	963<br>(739–1220)
Tonga	989<br>(757–1260)	959<br>(733–1220)
Tuvalu	989<br>(775–1270)	960<br>(735–1220)
Vanuatu	467<br>(415–525)	442<br>(355–544)
Southeast Asia	1150<br>(1080–1220)	1040<br>(952–1130)
Cambodia	997<br>(888–1120)	1160<br>(925–1460)
Indonesia	1030<br>(889–1170)	1140<br>(955–1340)
Lao People's Democratic Republic	1130<br>(1030–1220)	1170<br>(901–1480)
Malaysia	1290<br>(1150–1430)	932<br>(823–1060)
Maldives	1520<br>(1320–1730)	1200<br>(1000–1420)
Mauritius	1140<br>(1050–1230)	916<br>(768–1080)
Myanmar	1720<br>(1490–1950)	1010<br>(820–1250)
Philippines	1620<br>(1470–1770)	1170<br>(980–1390)
Seychelles	1440<br>(1330–1550)	1340<br>(1080–1650)
Sri Lanka	733<br>(660–812)	403<br>(340–469)
Thailand	1080<br>(990–1200)	782<br>(652–936)
Timor-Leste	1160<br>(890–1500)	1060<br>(810–1350)
Viet Nam	850<br>(704–1010)	942<br>(765–1150)
**Sub-Saharan Africa**	**414**<br>**(399–429)**	**321**<br>**(307–336)**
Central Sub-Saharan Africa	314<br>(295–335)	298<br>(264–335)
Angola	665<br>(613–722)	586<br>(507–676)
Central African Republic	352<br>(300–410)	287<br>(230–347)

Congo	311 (276–351)	328 (273–395)
Democratic Republic of the Congo	217 (194–241)	196 (156–243)
Equatorial Guinea	361 (277–468)	355 (271–455)
Gabon	325 (287–366)	351 (277–441)
Eastern Sub-Saharan Africa	300 (288–313)	260 (244–277)
Burundi	428 (373–491)	292 (230–365)
Comoros	337 (256–429)	316 (246–398)
Djibouti	697 (537–883)	655 (502–835)
Eritrea	337 (258–434)	313 (240–402)
Ethiopia	115 (106–124)	95·1 (81·0–110)
Kenya	365 (316–419)	334 (280–392)
Madagascar	498 (458–543)	280 (230–335)
Malawi	335 (272–409)	326 (254–414)
Mozambique	334 (307–357)	308 (242–389)
Rwanda	460 (372–556)	488 (386–604)
Somalia	335 (260–423)	316 (241–402)
South Sudan	337 (256–431)	317 (242–411)
Uganda	251 (220–289)	249 (196–313)
United Republic of Tanzania	416 (375–457)	346 (291–410)
Zambia	308 (274–344)	296 (233–367)
Southern Sub-Saharan Africa	1260 (1140–1390)	811 (703–930)
Botswana	963 (862–1080)	798 (665–958)
Eswatini	269 (241–301)	230 (183–291)
Lesotho	1120 (1000–1260)	1230 (1000–1470)
Namibia	358 (296–429)	294 (238–358)
South Africa	1430 (1270–1600)	807 (667–954)
Zimbabwe	932 (766–1130)	898 (694–1130)

Western Sub-Saharan Africa	324 (310–341)	298 (279–320)
Benin	351 (301–404)	285 (229–349)
Burkina Faso	274 (230–324)	241 (193–298)
Cabo Verde	339 (314–368)	212 (181–249)
Cameroon	432 (365–506)	358 (295–435)
Chad	342 (263–440)	318 (247–413)
Côte d'Ivoire	750 (669–834)	734 (603–881)
Gambia	627 (557–706)	449 (368–550)
Ghana	209 (192–226)	209 (174–250)
Guinea	595 (528–665)	568 (473–689)
Guinea-Bissau	166 (155–178)	179 (150–213)
Liberia	242 (214–271)	223 (183–270)
Mali	186 (163–212)	252 (209–299)
Mauritania	545 (511–579)	412 (330–507)
Niger	180 (158–205)	229 (184–279)
Nigeria	225 (200–253)	224 (191–261)
São Tomé and Príncipe	143 (122–166)	161 (131–197)
Senegal	685 (636–737)	505 (405–625)
Sierra Leone	406 (380–433)	335 (274–407)
Togo	810 (696–938)	626 (493–782)

Supplemental Table S5. All-cause and all-age smoking attributable deaths by location and sex, 2019. Estimates are reported as total number of attributable deaths, percent of deaths attributable to smoking, and smoking attributable death rate (per 100,000).

Location	Sex	Number	Percent	Rate
**Global**	**Both**	**7690000 (7160000–8200000)**	**13·6 (13·0–14·3)**	**99·4 (92·5–106)**
**Global**	**Female**	**1510000 (1400000–1630000)**	**5·84 (5·47–6·22)**	**39·2 (36·3–42·2)**
**Global**	**Male**	**6180000 (5680000–6680000)**	**20·2 (19·3–21·1)**	**159 (146–172)**
**Central Europe, Eastern Europe, and Central Asia**	**Both**	**775000 (712000–841000)**	**16·3 (15·6–17·1)**	**185 (170–201)**
**Central Europe, Eastern Europe, and Central Asia**	**Female**	**149000 (135000–167000)**	**6·40 (5·99–6·82)**	**68·5 (61·8–76·7)**
**Central Europe, Eastern Europe, and Central Asia**	**Male**	**625000 (569000–683000)**	**26·0 (25·2–26·7)**	**313 (285–342)**
Central Asia	Both	84400 (77000–92500)	13·2 (12·8–13·7)	90·2 (82·3–98·9)
Central Asia	Female	7540 (6590–8580)	2·61 (2·36–2·89)	16·0 (14·0–18·2)
Central Asia	Male	76800 (69800–84500)	22·0 (21·3–22·8)	166 (151–182)
Armenia	Both	4860 (4090–5690)	17·4 (16·6–18·1)	161 (136–189)
Armenia	Female	251 (197–323)	1·85 (1·51–2·26)	16·1 (12·6–20·7)
Armenia	Male	4610 (3880–5390)	32·0 (30·8–33·4)	317 (266–370)
Azerbaijan	Both	12500 (10400–15300)	16·6 (14·8–18·5)	122 (101–149)
Azerbaijan	Female	443 (316–601)	1·29 (0·970–1·67)	8·62 (6·16–11·7)
Azerbaijan	Male	12100 (9960–14800)	29·5 (28·0–31·0)	234 (194–288)
Georgia	Both	7170 (6120–8360)	14·5 (13·8–15·3)	196 (167–228)
Georgia	Female	744 (592–926)	3·08 (2·58–3·73)	39·0 (31·0–48·5)
Georgia	Male	6430 (5490–7470)	25·4 (24·3–26·7)	366 (313–426)
Kazakhstan	Both	20200 (17600–23000)	14·5 (13·7–15·4)	110 (95·6–125)
Kazakhstan	Female	2200 (1790–2660)	3·43 (2·88–4·04)	23·2 (18·9–28·1)
Kazakhstan	Male	18000 (15700–20600)	23·8 (22·6–25·3)	202 (176–231)
Kyrgyzstan	Both	5030 (4380–5690)	14·5 (13·7–15·4)	76·9 (67·0–87·1)
Kyrgyzstan	Female	569 (456–694)	3·69 (3·06–4·39)	17·2 (13·8–21·0)

Kyrgyzstan	Male	4460 (3900–5060)	23·1 (22·0–24·3)	138 (121–157)
Mongolia	Both	3770 (2960–4840)	15·1 (14·1–16·2)	111 (87·4–143)
Mongolia	Female	486 (368–629)	4·95 (4·17–5·77)	28·3 (21·5–36·7)
Mongolia	Male	3280 (2590–4170)	21·8 (20·3–23·3)	196 (155–250)
Tajikistan	Both	4630 (3740–5770)	9·51 (8·62–10·6)	48·8 (39·4–60·8)
Tajikistan	Female	470 (346–637)	2·19 (1·68–2·87)	10·0 (7·38–13·6)
Tajikistan	Male	4160 (3370–5190)	15·3 (13·9–16·7)	86·8 (70·2–108)
Turkmenistan	Both	4040 (3210–5050)	12·0 (11·2–12·8)	79·6 (63·1–99·4)
Turkmenistan	Female	529 (382–697)	3·59 (2·85–4·40)	21·3 (15·3–28·0)
Turkmenistan	Male	3510 (2780–4380)	18·6 (17·4–19·8)	135 (107–169)
Uzbekistan	Both	22200 (18500–26200)	10·9 (10·3–11·5)	65·9 (55·1–77·8)
Uzbekistan	Female	1850 (1380–2440)	2·03 (1·61–2·57)	11·0 (8·16–14·5)
Uzbekistan	Male	20300 (17000–23900)	18·1 (17·0–19·1)	121 (101–142)
Central Europe	Both	248000 (218000–282000)	18·1 (17·4–18·8)	217 (190–247)
Central Europe	Female	74600 (65100–86100)	11·1 (10·5–11·7)	127 (111–147)
Central Europe	Male	174000 (151000–198000)	24·8 (24·0–25·7)	312 (271–356)
Albania	Both	4420 (3410–5690)	19·5 (18·2–21·0)	163 (125–209)
Albania	Female	801 (594–1060)	8·10 (6·70–9·86)	59·0 (43·8–78·2)
Albania	Male	3620 (2750–4690)	28·3 (26·7–30·2)	266 (202–344)
Bosnia and Herzegovina	Both	8700 (6950–10900)	23·2 (22·2–24·4)	264 (211–329)
Bosnia and Herzegovina	Female	2850 (2250–3540)	15·4 (14·0–16·9)	168 (133–209)
Bosnia and Herzegovina	Male	5850 (4660–7280)	30·8 (29·6–32·2)	364 (289–452)
Bulgaria	Both	20600 (16500–25600)	16·6 (15·7–17·6)	298 (238–369)
Bulgaria	Female	5350 (4190–6770)	8·76 (7·86–9·85)	150 (117–190)
Bulgaria	Male	15300 (12200–18700)	24·1 (23·0–25·4)	454 (363–556)
Croatia	Both	9640 (7810–12000)	18·4 (17·5–19·5)	227 (184–282)
Croatia	Female	3070 (2470–3840)	11·6 (10·5–12·7)	140 (113–175)

Croatia	Male	6570 (5260–8110)	25·4 (24·2–26·7)	319 (255–394)
Czechia	Both	20700 (17000–24900)	18·2 (17·3–19·1)	195 (159–234)
Czechia	Female	6840 (5610–8280)	12·2 (11·1–13·2)	127 (104–153)
Czechia	Male	13900 (11400–16800)	24·1 (23·0–25·2)	265 (217–320)
Hungary	Both	24800 (20600–29900)	19·2 (18·4–20·1)	257 (213–309)
Hungary	Female	8730 (7200–10600)	13·1 (12·1–14·0)	172 (142–209)
Hungary	Male	16100 (13300–19400)	25·9 (24·8–27·1)	349 (289–421)
Montenegro	Both	1760 (1490–2070)	25·9 (24·4–27·5)	284 (241–333)
Montenegro	Female	604 (511–710)	19·3 (17·1–21·6)	193 (163–226)
Montenegro	Male	1160 (963–1390)	31·6 (29·9–33·3)	377 (314–452)
North Macedonia	Both	5310 (4230–6580)	22·0 (20·8–23·3)	247 (196–305)
North Macedonia	Female	1650 (1290–2070)	14·6 (13·0–16·4)	155 (122–195)
North Macedonia	Male	3660 (2910–4540)	28·6 (27·1–30·2)	335 (266–415)
Poland	Both	77700 (65000–92400)	19·1 (17·9–20·4)	202 (169–240)
Poland	Female	24200 (19200–29900)	12·2 (11·4–13·3)	122 (96·9–151)
Poland	Male	53500 (42300–66500)	25·5 (24·5–26·7)	288 (228–358)
Romania	Both	39000 (32300–46500)	14·8 (14·1–15·6)	203 (168–242)
Romania	Female	9260 (7450–11300)	7·25 (6·46–8·11)	93·7 (75·5–114)
Romania	Male	29700 (24600–35800)	22·0 (21·0–23·0)	318 (263–382)
Serbia	Both	24400 (19600–29700)	20·7 (19·7–21·8)	278 (224–339)
Serbia	Female	8450 (6870–10400)	14·5 (13·3–15·7)	192 (156–235)
Serbia	Male	15900 (12800–19500)	26·7 (25·4–28·1)	367 (294–450)
Slovakia	Both	8220 (6480–10200)	15·1 (14·2–16·0)	151 (119–187)
Slovakia	Female	1910 (1490–2410)	7·07 (6·32–7·89)	68·7 (53·6–86·5)
Slovakia	Male	6310 (4980–7790)	22·9 (21·6–24·3)	238 (188–293)
Slovenia	Both	2980 (2330–3800)	14·3 (13·4–15·4)	144 (112–183)
Slovenia	Female	892 (680–1150)	8·59 (7·73–9·67)	85·3 (65·0–110)

Slovenia	Male	2090 (1640–2680)	20·0 (18·7–21·3)	203 (160–261)
Eastern Europe	Both	442000 (391000–495000)	16·2 (15·2–17·3)	211 (186–236)
Eastern Europe	Female	67200 (57400–78500)	4·90 (4·49–5·30)	59·8 (51·0–69·8)
Eastern Europe	Male	375000 (325000–427000)	27·6 (26·8–28·4)	384 (333–437)
Belarus	Both	20500 (16400–25500)	16·8 (16·0–17·7)	216 (173–268)
Belarus	Female	2790 (2100–3650)	4·52 (3·84–5·30)	55·0 (41·5–71·9)
Belarus	Male	17700 (14100–22000)	29·5 (28·2–30·8)	400 (319–497)
Estonia	Both	2200 (1730–2760)	13·8 (12·9–15·5)	167 (132–211)
Estonia	Female	644 (505–817)	7·35 (6·61–8·74)	92·8 (72·7–118)
Estonia	Male	1550 (1210–1960)	21·7 (20·4–24·0)	251 (195–318)
Latvia	Both	3690 (3070–4420)	13·4 (12·0–15·0)	193 (160–231)
Latvia	Female	835 (642–1100)	5·47 (4·83–6·26)	80·8 (62·1–106)
Latvia	Male	2850 (2260–3540)	23·4 (22·2–24·8)	324 (257–401)
Lithuania	Both	4930 (4080–5950)	12·8 (12·1–13·6)	177 (146–213)
Lithuania	Female	843 (675–1050)	4·11 (3·60–4·74)	56·0 (44·8–69·6)
Lithuania	Male	4090 (3380–4940)	22·7 (21·6–24·0)	318 (262–383)
Republic of Moldova	Both	5920 (5170–6730)	14·4 (13·5–15·4)	161 (140–183)
Republic of Moldova	Female	685 (538–854)	3·45 (2·78–4·21)	35·5 (27·9–44·3)
Republic of Moldova	Male	5240 (4600–5910)	24·8 (23·4–26·3)	298 (261–336)
Russian Federation	Both	291000 (247000–337000)	16·3 (14·9–17·8)	199 (169–230)
Russian Federation	Female	46800 (38700–56900)	5·16 (4·70–5·62)	59·7 (49·3–72·6)
Russian Federation	Male	245000 (204000–292000)	27·7 (26·8–28·8)	358 (298–427)
Ukraine	Both	113000 (94200–134000)	16·2 (14·6–17·9)	257 (214–305)
Ukraine	Female	14600 (11300–18600)	4·30 (3·74–4·94)	61·5 (47·6–78·5)
Ukraine	Male	98800 (80400–120000)	27·5 (26·3–28·6)	487 (396–590)
**High-income**	**Both**	**1590000 (1530000–1660000)**	**16·0 (15·3–16·7)**	**147 (141–153)**
**High-income**	**Female**	**555000 (525000–586000)**	**11·3 (10·7–11·9)**	**101 (95·4–106)**

**High-income**	**Male**	**1030000 (992000–1080000)**	**20·5 (19·7–21·4)**	**194 (186–203)**
Australasia	Both	23800 (22500–25300)	11·6 (11·0–12·3)	81·9 (77·4–87·1)
Australasia	Female	10200 (9360–11100)	10·3 (9·50–11·2)	69·0 (63·4–75·1)
Australasia	Male	13600 (12900–14400)	12·8 (12·1–13·5)	95·1 (89·8–101)
Australia	Both	19000 (17900–20300)	11·1 (10·5–11·8)	77·3 (72·7–82·6)
Australia	Female	8100 (7340–8930)	9·89 (9·00–10·9)	65·1 (59·0–71·8)
Australia	Male	10900 (10200–11600)	12·3 (11·5–13·0)	89·8 (84·3–96·0)
New Zealand	Both	4790 (4510–5100)	13·9 (13·1–14·7)	107 (100–113)
New Zealand	Female	2080 (1910–2250)	12·4 (11·4–13·3)	89·9 (82·6–97·4)
New Zealand	Male	2720 (2540–2900)	15·3 (14·4–16·3)	124 (116–133)
High-income Asia Pacific	Both	254000 (238000–274000)	14·6 (13·7–15·7)	136 (127–146)
High-income Asia Pacific	Female	45600 (40400–52100)	5·38 (4·77–6·12)	48·0 (42·5–54·9)
High-income Asia Pacific	Male	208000 (196000–223000)	23·3 (21·9–24·9)	226 (212–241)
Brunei Darussalam	Both	267 (231–309)	14·2 (13·0–15·6)	61·1 (52·9–70·6)
Brunei Darussalam	Female	62·2 (50·2–77·0)	7·38 (6·07–9·01)	29·9 (24·1–37·0)
Brunei Darussalam	Male	205 (175–240)	19·7 (18·2–21·3)	89·4 (76·3–105)
Japan	Both	199000 (185000–216000)	14·2 (13·2–15·4)	156 (145–169)
Japan	Female	37000 (32200–42600)	5·37 (4·68–6·18)	56·4 (49·2–65·1)
Japan	Male	162000 (151000–176000)	22·8 (21·3–24·6)	261 (243–282)
Republic of Korea	Both	51600 (47700–55600)	16·2 (15·1–17·3)	96·6 (89·3–104)
Republic of Korea	Female	8070 (6570–9660)	5·45 (4·46–6·52)	30·5 (24·9–36·6)
Republic of Korea	Male	43500 (40500–46900)	25·5 (23·9–27·1)	161 (150–174)
Singapore	Both	2810 (2600–3050)	12·1 (11·2–13·1)	49·6 (45·8–53·8)
Singapore	Female	518 (430–629)	4·80 (4·01–5·81)	18·7 (15·5–22·7)
Singapore	Male	2300 (2120–2480)	18·5 (17·1–19·8)	79·3 (73·3–85·7)
High-income North America	Both	578000 (554000–603000)	17·9 (17·1–18·7)	158 (152–165)
High-income North America	Female	250000 (235000–265000)	16·0 (15·0–17·0)	135 (127–143)

High-income North America	Male	328000 (315000–343000)	19·6 (18·8–20·4)	183 (175–191)
Canada	Both	49600 (46900–52600)	17·2 (16·3–18·2)	136 (128–144)
Canada	Female	20900 (19200–22700)	14·9 (13·7–16·2)	113 (104–122)
Canada	Male	28700 (27000–30500)	19·4 (18·4–20·5)	160 (150–170)
Greenland	Both	127 (105–150)	26·5 (24·9–28·1)	226 (187–268)
Greenland	Female	50·1 (41·3–60·3)	26·7 (24·3–29·0)	188 (155–227)
Greenland	Male	77·2 (62·7–90·9)	26·4 (24·7–28·1)	261 (212–307)
United States of America	Both	528000 (506000–551000)	17·9 (17·2–18·7)	161 (154–168)
United States of America	Female	229000 (215000–243000)	16·1 (15·1–17·1)	137 (129–146)
United States of America	Male	299000 (287000–313000)	19·6 (18·8–20·4)	185 (178–194)
Southern Latin America	Both	67400 (63700–71300)	13·6 (12·9–14·4)	101 (95·4–107)
Southern Latin America	Female	25600 (23500–27900)	10·8 (9·88–11·6)	75·0 (68·8–81·7)
Southern Latin America	Male	41700 (39600–44000)	16·2 (15·4–17·0)	128 (122–135)
Argentina	Both	51300 (48200–54600)	14·7 (13·8–15·6)	114 (107–121)
Argentina	Female	19700 (17900–21700)	11·8 (10·7–12·8)	85·4 (77·6–93·7)
Argentina	Male	31600 (29800–33400)	17·4 (16·5–18·5)	144 (136–152)
Chile	Both	11200 (10400–12100)	9·89 (9·23–10·6)	61·4 (57·2–66·5)
Chile	Female	4450 (4000–5020)	8·32 (7·50–9·27)	48·1 (43·2–54·2)
Chile	Male	6730 (6240–7280)	11·3 (10·5–12·1)	75·3 (69·8–81·5)
Uruguay	Both	4860 (4600–5170)	14·4 (13·6–15·2)	142 (134–151)
Uruguay	Female	1430 (1280–1590)	8·44 (7·61–9·39)	79·8 (71·6–89·0)
Uruguay	Male	3440 (3250–3640)	20·3 (19·3–21·3)	209 (197–221)
Western Europe	Both	667000 (640000–698000)	15·6 (15·0–16·3)	153 (147–160)
Western Europe	Female	224000 (212000–238000)	10·3 (9·79–11·0)	101 (95·6–107)
Western Europe	Male	443000 (425000–463000)	21·0 (20·1–21·9)	207 (198–216)
Andorra	Both	106 (82·3–133)	17·2 (15·6–18·7)	128 (99·1–160)
Andorra	Female	23·9 (16·6–32·6)	8·84 (7·37–10·5)	59·1 (41·0–80·7)

Andorra	Male	82·6 (65·6–101)	23·6 (21·6–25·5)	194 (154–238)
Austria	Both	12300 (11700–13200)	14·9 (14·2–16·0)	138 (131–148)
Austria	Female	4290 (3920–4720)	10·1 (9·27–11·1)	94·8 (86·7–104)
Austria	Male	8030 (7570–8570)	20·0 (19·0–21·3)	183 (172–195)
Belgium	Both	19600 (18600–20900)	17·2 (16·3–18·2)	172 (162–183)
Belgium	Female	6380 (5870–7020)	11·0 (10·2–12·1)	110 (101–121)
Belgium	Male	13200 (12600–14000)	23·5 (22·4–24·8)	236 (224–249)
Cyprus	Both	1530 (1370–1720)	17·6 (16·6–18·7)	117 (105–131)
Cyprus	Female	341 (294–395)	8·26 (7·26–9·39)	51·3 (44·3–59·4)
Cyprus	Male	1190 (1070–1340)	26·1 (24·7–27·6)	184 (165–206)
Denmark	Both	12700 (12000–13500)	23·0 (21·9–24·2)	219 (207–233)
Denmark	Female	5720 (5270–6160)	21·0 (19·5–22·3)	196 (181–211)
Denmark	Male	7010 (6600–7460)	24·9 (23·7–26·3)	243 (228–258)
Finland	Both	6760 (6320–7290)	12·1 (11·4–12·8)	122 (114–132)
Finland	Female	2180 (1970–2420)	7·82 (7·12–8·67)	77·8 (70·2–86·4)
Finland	Male	4580 (4290–4910)	16·2 (15·4–17·2)	168 (157–180)
France	Both	80300 (75200–86700)	13·3 (12·5–14·3)	121 (114–131)
France	Female	23700 (21100–26800)	7·81 (6·98–8·80)	69·4 (62·0–78·6)
France	Male	56700 (53200–60300)	18·9 (17·8–20·0)	177 (166–188)
Germany	Both	144000 (136000–153000)	15·0 (14·2–15·9)	169 (160–180)
Germany	Female	49300 (45300–54100)	10·1 (9·28–11·0)	115 (106–127)
Germany	Male	94400 (89200–100000)	20·0 (19·0–21·2)	224 (211–237)
Greece	Both	26300 (24900–27900)	20·4 (19·5–21·5)	254 (241–269)
Greece	Female	7310 (6670–8010)	11·4 (10·4–12·5)	138 (126–151)
Greece	Male	18900 (18000–20100)	29·3 (28·0–30·9)	377 (358–399)
Iceland	Both	345 (310–381)	16·3 (15·3–17·6)	99·9 (89·8–110)
Iceland	Female	130 (114–148)	13·7 (12·3–15·3)	76·0 (66·6–86·9)

Iceland	Male	215 (192–239)	18·5 (17·2–19·8)	123 (110–137)
Ireland	Both	6180 (5810–6610)	19·1 (18·1–20·2)	126 (118–135)
Ireland	Female	2620 (2400–2840)	16·7 (15·4–18·0)	105 (96·8–114)
Ireland	Male	3570 (3340–3810)	21·3 (20·0–22·6)	147 (138–157)
Israel	Both	6140 (5730–6640)	12·8 (12·1–13·8)	66·0 (61·6–71·4)
Israel	Female	1950 (1750–2190)	8·12 (7·28–9·10)	41·7 (37·3–46·8)
Israel	Male	4200 (3940–4510)	17·5 (16·6–18·7)	90·5 (85·1–97·3)
Italy	Both	90500 (85700–96300)	14·1 (13·4–15·0)	150 (142–160)
Italy	Female	27200 (25000–30100)	8·17 (7·51–9·02)	87·9 (80·6–97·1)
Italy	Male	63300 (59900–67200)	20·5 (19·5–21·7)	216 (204–229)
Luxembourg	Both	676 (594–774)	16·3 (14·9–17·7)	109 (96·1–125)
Luxembourg	Female	239 (200–280)	11·4 (9·92–12·9)	77·6 (65·1–91·0)
Luxembourg	Male	437 (385–498)	21·3 (19·8–23·0)	141 (124–160)
Malta	Both	551 (496–616)	14·6 (13·7–15·6)	125 (113–140)
Malta	Female	142 (122–170)	7·79 (6·87–8·94)	64·8 (55·6–77·3)
Malta	Male	408 (369–453)	20·9 (19·7–22·1)	186 (168–206)
Monaco	Both	97·3 (80·4–115)	18·6 (16·9–20·3)	259 (214–305)
Monaco	Female	32·1 (24·2–40·6)	12·4 (10·5–14·6)	166 (126–211)
Monaco	Male	65·2 (54·9–74·8)	24·5 (22·3–26·6)	356 (300–409)
Netherlands	Both	31500 (29900–33300)	20·1 (19·1–21·1)	184 (174–194)
Netherlands	Female	12800 (11700–13800)	16·1 (14·8–17·4)	148 (136–160)
Netherlands	Male	18700 (17800–19800)	24·1 (22·9–25·5)	220 (209–233)
Norway	Both	4820 (4500–5150)	11·6 (10·9–12·4)	90·1 (84·1–96·2)
Norway	Female	1880 (1650–2130)	8·92 (7·84–10·1)	70·8 (62·2–80·2)
Norway	Male	2940 (2740–3150)	14·5 (13·6–15·4)	109 (102–117)
Portugal	Both	12100 (11400–12900)	10·4 (9·80–11·1)	114 (107–121)
Portugal	Female	1860 (1620–2140)	3·20 (2·79–3·67)	33·3 (29·0–38·3)

Portugal	Male	10300 (9620–11000)	17·7 (16·6–18·8)	203 (190–216)
San Marino	Both	41·5 (27·4–58·1)	13·7 (12·2–15·2)	125 (82·8–175)
San Marino	Female	11·8 (7·58–17·8)	7·99 (6·51–9·56)	68·6 (44·2–104)
San Marino	Male	29·7 (19·7–40·6)	19·1 (17·2–20·9)	187 (124–255)
Spain	Both	66300 (62500–70300)	15·5 (14·7–16·4)	144 (136–153)
Spain	Female	11900 (10600–13400)	5·56 (4·95–6·25)	50·9 (45·2–57·2)
Spain	Male	54400 (51200–57800)	25·4 (24·1–27·0)	241 (227–256)
Sweden	Both	13700 (12800–14800)	14·6 (13·6–15·8)	134 (125–145)
Sweden	Female	6610 (6050–7220)	14·1 (12·9–15·3)	130 (119–142)
Sweden	Male	7080 (6550–7720)	15·1 (14·0–16·5)	138 (128–150)
Switzerland	Both	10200 (9530–11000)	14·6 (13·7–15·7)	116 (109–125)
Switzerland	Female	3730 (3360–4220)	10·4 (9·37–11·6)	84·7 (76·3–95·7)
Switzerland	Male	6490 (6050–6960)	19·2 (18·0–20·5)	148 (138–159)
United Kingdom	Both	120000 (114000–126000)	19·3 (18·4–20·2)	178 (170–187)
United Kingdom	Female	53200 (49800–56300)	17·1 (16·0–18·1)	157 (147–166)
United Kingdom	Male	66600 (63300–70000)	21·4 (20·5–22·5)	200 (190–211)
**Latin America and Caribbean**	**Both**	**324000 (300000–352000)**	**9·07 (8·60–9·59)**	**55·5 (51·3–60·3)**
**Latin America and Caribbean**	**Female**	**106000 (96700–116000)**	**6·62 (6·14–7·11)**	**35·5 (32·5–39·0)**
**Latin America and Caribbean**	**Male**	**219000 (201000–239000)**	**11·0 (10·5–11·6)**	**76·4 (70·1–83·4)**
Andean Latin America	Both	12800 (10600–15300)	4·00 (3·68–4·35)	20·2 (16·6–24·1)
Andean Latin America	Female	2550 (2050–3150)	1·70 (1·47–1·96)	8·02 (6·46–9·92)
Andean Latin America	Male	10300 (8380–12300)	6·01 (5·52–6·55)	32·3 (26·3–38·6)
Bolivia (Plurinational State of)	Both	3530 (2760–4360)	4·64 (4·16–5·12)	29·4 (23·0–36·3)
Bolivia (Plurinational State of)	Female	647 (484–856)	1·73 (1·39–2·12)	10·8 (8·06–14·3)
Bolivia (Plurinational State of)	Male	2880 (2250–3560)	7·47 (6·73–8·22)	47·9 (37·4–59·2)
Ecuador	Both	4800 (3810–6140)	5·19 (4·70–5·73)	27·3 (21·7–34·9)
Ecuador	Female	1010 (775–1300)	2·45 (2·06–2·94)	11·5 (8·78–14·8)

Ecuador	Male	3790 (2980–4860)	7·40 (6·72–8·15)	43·3 (34·1–55·6)
Peru	Both	4490 (3320–6020)	2·94 (2·57–3·33)	13·2 (9·78–17·7)
Peru	Female	890 (632–1240)	1·25 (1·00–1·54)	5·24 (3·73–7·30)
Peru	Male	3600 (2650–4840)	4·42 (3·86–5·06)	21·1 (15·6–28·4)
Caribbean	Both	40000 (34500–46600)	10·6 (9·80–11·4)	84·9 (73·2–98·7)
Caribbean	Female	12300 (10500–14500)	7·03 (6·29–7·79)	51·5 (43·9–60·6)
Caribbean	Male	27700 (23800–32200)	13·6 (12·7–14·6)	119 (102–139)
Antigua and Barbuda	Both	36·8 (31·0–43·9)	6·04 (5·45–6·71)	41·6 (35·0–49·6)
Antigua and Barbuda	Female	11·2 (8·93–14·0)	3·75 (3·09–4·60)	24·7 (19·7–30·9)
Antigua and Barbuda	Male	25·7 (21·5–30·6)	8·20 (7·39–9·04)	59·4 (49·7–70·7)
Bahamas	Both	155 (126–192)	5·69 (5·09–6·36)	41·2 (33·3–51·0)
Bahamas	Female	33·1 (25·1–42·7)	2·78 (2·25–3·41)	17·0 (12·9–22·0)
Bahamas	Male	122 (98·3–152)	7·95 (7·13–8·79)	67·1 (53·9–83·6)
Barbados	Both	157 (131–188)	5·07 (4·59–5·56)	52·7 (43·9–63·2)
Barbados	Female	24·3 (18·8–31·5)	1·55 (1·28–1·87)	15·8 (12·2–20·4)
Barbados	Male	133 (110–159)	8·70 (7·88–9·60)	92·4 (76·7–111)
Belize	Both	138 (118–160)	6·93 (6·33–7·58)	33·7 (28·7–39·1)
Belize	Female	21·3 (17·1–26·5)	2·78 (2·29–3·34)	10·4 (8·28–12·9)
Belize	Male	117 (99·8–136)	9·54 (8·78–10·4)	57·2 (48·9–66·6)
Bermuda	Both	61·3 (51·8–74·0)	10·6 (9·74–11·4)	95·7 (80·9–115)
Bermuda	Female	14·8 (11·8–19·3)	5·97 (5·14–6·96)	44·6 (35·6–58·3)
Bermuda	Male	46·5 (39·4–55·5)	14·0 (12·9–15·1)	150 (127–179)
Cuba	Both	20000 (16300–24100)	18·8 (17·8–20·1)	176 (144–212)
Cuba	Female	6070 (4810–7520)	12·5 (10·9–14·3)	106 (84·3–132)
Cuba	Male	13900 (11400–16900)	24·2 (22·8–25·7)	246 (201–299)
Dominica	Both	41·4 (33·8–50·6)	5·63 (5·14–6·15)	60·3 (49·3–73·6)
Dominica	Female	8·29 (6·47–10·6)	2·47 (2·06–2·95)	24·6 (19·2–31·6)

Dominica	Male	33·2 (26·9–40·7)	8·30 (7·53–9·09)	94·6 (76·7–116)
Dominican Republic	Both	8200 (6480–10500)	11·6 (10·6–12·7)	75·4 (59·6–96·1)
Dominican Republic	Female	3070 (2420–3890)	10·5 (9·08–11·9)	56·7 (44·8–71·9)
Dominican Republic	Male	5140 (4000–6580)	12·4 (11·3–13·6)	93·9 (73·2–120)
Grenada	Both	54·7 (48·5–61·1)	6·69 (6·08–7·31)	53·0 (47·0–59·2)
Grenada	Female	11·6 (9·38–14·0)	3·02 (2·51–3·63)	23·0 (18·6–27·9)
Grenada	Male	43·1 (38·2–48·4)	9·92 (9·04–10·9)	81·6 (72·2–91·6)
Guyana	Both	423 (326–542)	6·32 (5·77–6·96)	54·9 (42·3–70·3)
Guyana	Female	82·5 (61·1–110)	2·85 (2·34–3·52)	21·2 (15·7–28·2)
Guyana	Male	341 (260–436)	8·97 (8·17–9·82)	89·3 (68·1–114)
Haiti	Both	3120 (2350–4200)	3·12 (2·65–3·57)	25·1 (18·9–33·9)
Haiti	Female	891 (650–1250)	1·79 (1·43–2·19)	13·9 (10·2–19·6)
Haiti	Male	2230 (1660–2980)	4·43 (3·68–5·16)	37·0 (27·5–49·5)
Jamaica	Both	1720 (1370–2140)	8·75 (8·10–9·49)	61·2 (48·9–76·2)
Jamaica	Female	398 (308–509)	4·17 (3·53–4·86)	28·1 (21·8–36·0)
Jamaica	Male	1320 (1050–1650)	13·1 (12·1–14·1)	94·8 (75·0–118)
Puerto Rico	Both	2790 (2140–3520)	8·46 (7·56–9·45)	79·2 (60·7–99·9)
Puerto Rico	Female	885 (682–1130)	5·62 (4·82–6·53)	47·9 (36·9–61·4)
Puerto Rico	Male	1900 (1450–2430)	11·1 (9·96–12·3)	114 (86·9–145)
Saint Kitts and Nevis	Both	25·2 (21·0–30·0)	5·05 (4·41–5·66)	42·4 (35·3–50·3)
Saint Kitts and Nevis	Female	5·03 (3·85–6·49)	2·45 (1·95–3·05)	17·0 (13·0–21·9)
Saint Kitts and Nevis	Male	20·2 (16·7–24·0)	6·87 (5·89–7·73)	67·7 (55·8–80·5)
Saint Lucia	Both	106 (89·6–124)	7·50 (6·92–8·08)	60·4 (51·3–71·1)
Saint Lucia	Female	19·7 (15·5–24·7)	3·21 (2·65–3·84)	22·5 (17·6–28·1)
Saint Lucia	Male	85·8 (72·5–101)	10·8 (9·94–11·6)	98·8 (83·5–116)
Saint Vincent and the Grenadines	Both	61·6 (52·2–72·7)	6·00 (5·30–6·73)	54·4 (46·1–64·2)
Saint Vincent and the Grenadines	Female	11·5 (8·93–14·9)	2·58 (2·06–3·21)	20·8 (16·2–26·9)

Saint Vincent and the Grenadines	Male	50·1 (42·4–59·1)	8·63 (7·65–9·64)	86·5 (73·4–102)
Suriname	Both	451 (373–546)	10·3 (9·50–11·1)	78·3 (64·8–94·8)
Suriname	Female	105 (82·7–132)	5·33 (4·48–6·37)	36·1 (28·4–45·5)
Suriname	Male	346 (286–417)	14·4 (13·3–15·5)	121 (100–146)
Trinidad and Tobago	Both	1030 (761–1360)	8·72 (8·04–9·50)	74·1 (54·9–97·8)
Trinidad and Tobago	Female	203 (147–282)	3·91 (3·23–4·72)	29·4 (21·3–40·8)
Trinidad and Tobago	Male	825 (613–1090)	12·5 (11·5–13·6)	118 (88·0–156)
United States Virgin Islands	Both	109 (91·3–129)	8·54 (7·48–9·64)	105 (87·8–124)
United States Virgin Islands	Female	32·8 (26·0–40·3)	6·30 (5·24–7·49)	59·9 (47·6–73·8)
United States Virgin Islands	Male	76·5 (64·5–88·2)	10·1 (8·89–11·3)	155 (131–179)
Central Latin America	Both	97100 (83900–113000)	6·78 (6·37–7·20)	38·8 (33·6–45·3)
Central Latin America	Female	26100 (22100–31000)	4·14 (3·73–4·53)	20·4 (17·3–24·3)
Central Latin America	Male	71000 (60500–83500)	8·85 (8·36–9·40)	58·1 (49·5–68·4)
Colombia	Both	17300 (13400–22100)	7·00 (6·37–7·61)	36·2 (28·0–46·3)
Colombia	Female	6260 (4830–8070)	5·57 (4·77–6·44)	25·6 (19·8–33·0)
Colombia	Male	11000 (8440–14400)	8·21 (7·51–8·93)	47·2 (36·1–61·5)
Costa Rica	Both	2120 (1670–2670)	8·67 (8·12–9·31)	44·9 (35·4–56·5)
Costa Rica	Female	551 (433–701)	5·16 (4·47–5·90)	22·7 (17·8–28·8)
Costa Rica	Male	1570 (1220–1980)	11·4 (10·7–12·2)	68·6 (53·2–86·9)
El Salvador	Both	1700 (1280–2190)	4·24 (3·82–4·72)	27·2 (20·5–35·0)
El Salvador	Female	517 (372–703)	2·90 (2·34–3·49)	15·6 (11·2–21·1)
El Salvador	Male	1190 (892–1530)	5·30 (4·76–5·85)	40·5 (30·4–52·3)
Guatemala	Both	3790 (2870–4920)	3·99 (3·44–4·62)	21·3 (16·2–27·7)
Guatemala	Female	879 (628–1180)	2·10 (1·66–2·63)	9·62 (6·88–12·9)
Guatemala	Male	2910 (2200–3780)	5·49 (4·77–6·31)	33·7 (25·5–43·7)
Honduras	Both	4230 (3620–4960)	8·05 (7·06–9·05)	43·1 (36·9–50·5)
Honduras	Female	1040 (777–1380)	4·13 (3·21–5·25)	20·6 (15·4–27·3)

Honduras	Male	3190 (2760–3670)	11·7 (10·3–13·3)	66·7 (57·7–76·8)
Mexico	Both	48400 (40700–57000)	6·55 (6·00–7·12)	38·7 (32·6–45·6)
Mexico	Female	11400 (9190–14100)	3·52 (3·10–3·97)	17·8 (14·4–22·0)
Mexico	Male	37000 (29900–45000)	8·90 (8·30–9·61)	60·6 (48·9–73·7)
Nicaragua	Both	1650 (1320–1980)	5·63 (5·06–6·20)	25·3 (20·3–30·4)
Nicaragua	Female	264 (206–335)	2·09 (1·69–2·53)	8·02 (6·26–10·2)
Nicaragua	Male	1380 (1110–1660)	8·34 (7·55–9·17)	43·0 (34·4–51·6)
Panama	Both	1180 (902–1520)	5·96 (5·41–6·58)	28·4 (21·7–36·4)
Panama	Female	311 (231–410)	3·63 (2·99–4·41)	15·1 (11·2–19·8)
Panama	Male	871 (663–1130)	7·75 (7·05–8·51)	41·6 (31·6–54·0)
Venezuela (Bolivarian Republic of)	Both	16800 (12700–21700)	8·96 (8·22–9·80)	59·7 (45·3–77·2)
Venezuela (Bolivarian Republic of)	Female	4900 (3670–6500)	6·23 (5·21–7·30)	34·3 (25·7–45·5)
Venezuela (Bolivarian Republic of)	Male	11900 (8900–15400)	10·9 (10·1–11·9)	86·2 (64·6–112)
Tropical Latin America	Both	175000 (164000–185000)	12·1 (11·5–12·8)	78·1 (73·5–82·9)
Tropical Latin America	Female	64700 (59500–70300)	10·1 (9·39–10·9)	56·6 (52·1–61·5)
Tropical Latin America	Male	110000 (103000–117000)	13·6 (13·0–14·4)	101 (94·3–107)
Brazil	Both	170000 (160000–181000)	12·1 (11·5–12·8)	78·5 (74·0–83·5)
Brazil	Female	63400 (58300–68900)	10·1 (9·43–11·0)	57·2 (52·6–62·2)
Brazil	Male	107000 (100000–114000)	13·6 (13·0–14·3)	101 (94·5–108)
Paraguay	Both	4410 (3430–5680)	12·9 (11·7–14·3)	63·7 (49·5–81·9)
Paraguay	Female	1230 (938–1630)	8·45 (7·00–10·1)	36·0 (27·4–47·6)
Paraguay	Male	3180 (2460–4070)	16·2 (15·0–17·7)	90·6 (70·0–116)
**North Africa and Middle East**	**Both**	**374000 (336000–417000)**	**12·1 (11·6–12·7)**	**61·5 (55·1–68·6)**
**North Africa and Middle East**	**Female**	**45300 (40500–51000)**	**3·37 (3·12–3·66)**	**15·5 (13·9–17·4)**
**North Africa and Middle East**	**Male**	**329000 (294000–368000)**	**18·7 (17·9–19·8)**	**104 (92·9–116)**
North Africa and Middle East	Both	374000 (336000–417000)	12·1 (11·6–12·7)	61·5 (55·1–68·6)
North Africa and Middle East	Female	45300 (40500–51000)	3·37 (3·12–3·66)	15·5 (13·9–17·4)

North Africa and Middle East	Male	329000 (294000–368000)	18·7 (17·9–19·8)	104 (92·9–116)
Afghanistan	Both	10300 (7830–13000)	4·09 (3·41–4·71)	26·9 (20·4–33·9)
Afghanistan	Female	1970 (1370–2750)	1·57 (1·20–2·07)	10·5 (7·36–14·8)
Afghanistan	Male	8340 (6320–10400)	6·58 (5·45–7·59)	42·5 (32·2–52·8)
Algeria	Both	21600 (17700–26100)	10·7 (9·95–11·7)	51·5 (42·2–62·3)
Algeria	Female	1930 (1450–2550)	2·04 (1·62–2·57)	9·34 (7·03–12·4)
Algeria	Male	19600 (16100–23800)	18·4 (17·1–20·0)	92·6 (75·9–112)
Bahrain	Both	545 (434–677)	12·8 (11·5–14·2)	37·8 (30·1–46·9)
Bahrain	Female	79·6 (58·2–107)	4·83 (3·75–6·15)	14·6 (10·7–19·7)
Bahrain	Male	466 (367–579)	17·8 (16·1–19·6)	51·9 (40·9–64·5)
Egypt	Both	74000 (57400–96200)	13·2 (12·0–14·7)	74·7 (57·9–97·1)
Egypt	Female	2780 (1920–3880)	1·18 (0·893–1·54)	5·84 (4·03–8·13)
Egypt	Male	71200 (55100–92900)	21·8 (19·9–24·3)	139 (107–181)
Iran (Islamic Republic of)	Both	39900 (37300–43000)	10·2 (9·61–10·9)	47·4 (44·3–51·0)
Iran (Islamic Republic of)	Female	5480 (4530–6490)	3·29 (2·74–3·91)	13·2 (10·9–15·6)
Iran (Islamic Republic of)	Male	34400 (32100–37300)	15·3 (14·5–16·4)	80·5 (75·0–87·1)
Iraq	Both	25200 (19600–30300)	14·0 (12·9–15·0)	59·8 (46·5–71·9)
Iraq	Female	4010 (3070–5130)	5·33 (4·47–6·29)	19·5 (14·9–25·0)
Iraq	Male	21200 (16400–25400)	20·3 (18·9–21·7)	98·2 (76·2–118)
Jordan	Both	4740 (3860–5770)	14·7 (12·9–16·4)	40·7 (33·2–49·6)
Jordan	Female	767 (610–956)	5·56 (4·84–6·33)	14·1 (11·2–17·6)
Jordan	Male	3980 (3140–4970)	21·5 (19·9–23·0)	64·0 (50·5–79·9)
Kuwait	Both	1520 (1250–1850)	15·2 (13·9–16·6)	34·4 (28·2–41·8)
Kuwait	Female	86·1 (65·1–114)	2·92 (2·33–3·66)	4·19 (3·17–5·54)
Kuwait	Male	1440 (1160–1760)	20·3 (19·1–21·8)	60·6 (49·0–74·3)
Lebanon	Both	7810 (6800–8900)	23·1 (21·5–24·9)	151 (131–172)
Lebanon	Female	2730 (2350–3170)	18·2 (16·3–20·3)	104 (89·2–121)

Lebanon	Male	5080 (4420–5790)	26·9 (25·2–28·9)	199 (174–227)
Libya	Both	3660 (2960–4610)	11·6 (10·7–12·7)	54·4 (44·0–68·4)
Libya	Female	111 (79·1–154)	0·813 (0·615–1·07)	3·42 (2·43–4·73)
Libya	Male	3550 (2870–4460)	19·7 (18·4–21·8)	102 (82·5–128)
Morocco	Both	21400 (16300–25300)	9·36 (8·46–10·3)	59·5 (45·5–70·5)
Morocco	Female	1400 (1020–1860)	1·34 (1·07–1·68)	7·81 (5·71–10·4)
Morocco	Male	20000 (15200–23800)	16·1 (14·8–17·8)	111 (84·0–131)
Oman	Both	812 (702–945)	6·55 (5·96–7·27)	17·7 (15·3–20·6)
Oman	Female	90·8 (67·1–119)	2·02 (1·54–2·61)	5·57 (4·12–7·33)
Oman	Male	721 (616–847)	9·15 (8·39–10·0)	24·4 (20·9–28·7)
Palestine	Both	2190 (1870–2550)	13·2 (12·1–14·3)	44·1 (37·7–51·4)
Palestine	Female	210 (159–270)	2·73 (2·13–3·43)	8·62 (6·54–11·1)
Palestine	Male	1980 (1690–2310)	22·1 (20·5–23·9)	78·2 (67·1–91·3)
Qatar	Both	418 (316–543)	9·44 (8·55–10·6)	14·6 (11·0–19·0)
Qatar	Female	15·6 (10·7–22·2)	1·44 (1·06–1·93)	2·15 (1·46–3·06)
Qatar	Male	402 (304–523)	12·0 (10·9–13·4)	18·8 (14·2–24·5)
Saudi Arabia	Both	12300 (9710–15200)	9·55 (8·55–10·8)	34·3 (27·2–42·7)
Saudi Arabia	Female	948 (659–1310)	2·21 (1·67–2·85)	6·37 (4·43–8·77)
Saudi Arabia	Male	11300 (8970–14000)	13·2 (11·9–14·9)	54·3 (43·0–67·4)
Sudan	Both	16100 (12600–20500)	7·98 (6·67–9·33)	39·6 (31·0–50·4)
Sudan	Female	1670 (1160–2390)	1·97 (1·39–2·68)	8·30 (5·74–11·9)
Sudan	Male	14500 (11400–18600)	12·3 (10·3–14·3)	70·0 (54·9–89·7)
Syrian Arab Republic	Both	13200 (9920–17600)	15·6 (14·4–17·0)	91·4 (68·5–121)
Syrian Arab Republic	Female	1860 (1310–2540)	5·15 (4·13–6·36)	25·0 (17·7–34·2)
Syrian Arab Republic	Male	11400 (8570–15300)	23·4 (21·7–25·2)	161 (121–217)
Tunisia	Both	10900 (8220–14100)	16·0 (15·0–17·3)	93·8 (71·0–122)
Tunisia	Female	779 (544–1070)	2·65 (2·08–3·26)	13·4 (9·37–18·4)

Tunisia	Male	10100 (7630–13200)	26·3 (24·8–28·2)	175 (132–229)
Turkey	Both	86200 (69400–105000)	18·9 (18·0–19·9)	106 (85·3–129)
Turkey	Female	13900 (11100–17100)	6·66 (5·99–7·39)	34·5 (27·6–42·5)
Turkey	Male	72300 (58700–88600)	29·3 (27·9–30·6)	176 (143–215)
United Arab Emirates	Both	3730 (2740–4910)	12·8 (11·0–14·7)	40·3 (29·6–53·2)
United Arab Emirates	Female	203 (143–280)	4·01 (3·14–5·07)	8·01 (5·65–11·1)
United Arab Emirates	Male	3520 (2580–4660)	14·6 (12·6–16·9)	52·5 (38·4–69·4)
Yemen	Both	17300 (13700–22500)	9·90 (8·66–11·2)	54·9 (43·6–71·3)
Yemen	Female	4290 (3310–5730)	5·61 (4·70–6·67)	27·6 (21·3–36·8)
Yemen	Male	13000 (10300–17100)	13·2 (11·6–15·2)	81·7 (64·5–107)
**South Asia**	**Both**	**1290000 (1120000–1480000)**	**10·8 (9·78–11·8)**	**71·3 (61·8–81·8)**
**South Asia**	**Female**	**207000 (161000–253000)**	**3·73 (3·00–4·39)**	**23·4 (18·2–28·7)**
**South Asia**	**Male**	**1080000 (921000–1260000)**	**16·9 (15·7–17·8)**	**117 (99·9–137)**
South Asia	Both	1290000 (1120000–1480000)	10·8 (9·78–11·8)	71·3 (61·8–81·8)
South Asia	Female	207000 (161000–253000)	3·73 (3·00–4·39)	23·4 (18·2–28·7)
South Asia	Male	1080000 (921000–1260000)	16·9 (15·7–17·8)	117 (99·9–137)
Bangladesh	Both	106000 (84800–129000)	12·4 (11·5–14·0)	66·4 (53·2–81·1)
Bangladesh	Female	8480 (6290–11300)	2·30 (1·81–2·98)	10·5 (7·80–14·0)
Bangladesh	Male	97200 (77900–119000)	20·2 (18·8–22·6)	124 (99·1–151)
Bhutan	Both	332 (256–419)	7·82 (6·49–9·49)	44·0 (33·9–55·5)
Bhutan	Female	74·0 (53·9–98·8)	3·85 (2·91–4·96)	20·5 (14·9–27·3)
Bhutan	Male	258 (195–333)	11·1 (9·09–13·7)	65·7 (49·7–84·8)
India	Both	1010000 (853000–1200000)	10·8 (9·56–12·0)	72·9 (61·3–86·1)
India	Female	169000 (126000–214000)	3·85 (3·02–4·63)	24·9 (18·6–31·6)
India	Male	845000 (695000–1020000)	16·9 (15·4–18·0)	118 (97·4–143)
Nepal	Both	34200 (27700–39300)	17·7 (16·0–19·2)	112 (91·2–129)
Nepal	Female	12800 (9930–15200)	14·6 (12·1–16·9)	80·7 (62·4–95·8)

Nepal	Male	21400 (17100–24800)	20·2 (18·1–22·0)	147 (118–171)
Pakistan	Both	134000 (107000–167000)	8·94 (7·44–10·6)	59·9 (47·9–74·7)
Pakistan	Female	16500 (12600–21900)	2·39 (1·94–2·95)	15·1 (11·5–20·1)
Pakistan	Male	118000 (89600–152000)	14·5 (12·5–16·3)	103 (78·0–132)
**Southeast Asia, East Asia, and Oceania**	**Both**	**3120000 (2740000–3550000)**	**20·1 (18·5–21·5)**	**145 (127–165)**
**Southeast Asia, East Asia, and Oceania**	**Female**	**413000 (346000–487000)**	**6·23 (5·65–6·91)**	**38·7 (32·5–45·7)**
**Southeast Asia, East Asia, and Oceania**	**Male**	**2710000 (2310000–3150000)**	**30·3 (29·2–31·5)**	**248 (211–288)**
East Asia	Both	2490000 (2100000–2930000)	22·5 (20·5–24·3)	169 (142–199)
East Asia	Female	334000 (271000–408000)	7·22 (6·50–8·07)	46·2 (37·4–56·5)
East Asia	Male	2160000 (1770000–2600000)	33·4 (32·4–34·5)	288 (236–346)
China	Both	2420000 (2030000–2870000)	22·7 (20·7–24·6)	170 (143–202)
China	Female	326000 (263000–400000)	7·35 (6·62–8·25)	46·7 (37·7–57·4)
China	Male	2090000 (1710000–2530000)	33·6 (32·6–34·8)	289 (236–349)
Democratic People's Republic of Korea	Both	43800 (37700–49600)	18·5 (17·0–19·8)	167 (144–189)
Democratic People's Republic of Korea	Female	6940 (5140–9140)	5·75 (4·43–7·26)	52·6 (39·0–69·3)
Democratic People's Republic of Korea	Male	36800 (31900–41300)	31·7 (29·3–33·7)	282 (244–316)
Taiwan (Province of China)	Both	26600 (21300–34200)	14·4 (13·5–15·2)	113 (90·0–145)
Taiwan (Province of China)	Female	1480 (1070–2030)	1·91 (1·54–2·35)	12·4 (9·00–17·1)
Taiwan (Province of China)	Male	25200 (20100–32300)	23·2 (21·9–24·5)	215 (171–276)
Oceania	Both	11000 (8920–13700)	11·4 (10·3–12·5)	83·1 (67·2–103)
Oceania	Female	3080 (2390–3920)	7·40 (6·33–8·58)	47·9 (37·2–60·9)
Oceania	Male	7950 (6430–9810)	14·3 (13·0–15·6)	116 (94·0–143)
American Samoa	Both	55·0 (46·5–63·6)	14·4 (13·2–15·7)	99·0 (83·7–115)
American Samoa	Female	15·1 (12·0–19·0)	8·87 (7·57–10·3)	54·9 (43·4–68·8)
American Samoa	Male	39·8 (34·0–45·2)	18·7 (17·3–20·4)	143 (122–162)
Cook Islands	Both	21·3 (17·9–25·3)	12·9 (11·6–14·2)	119 (99·7–141)
Cook Islands	Female	4·80 (3·55–6·26)	7·11 (5·75–8·60)	52·1 (38·5–68·0)

Cook Islands	Male	16·5 (14·1–19·3)	16·9 (15·2–18·6)	188 (161–220)
Fiji	Both	924 (722–1160)	12·4 (11·5–13·4)	101 (79·3–128)
Fiji	Female	184 (138–241)	5·49 (4·68–6·41)	41·0 (30·7–53·6)
Fiji	Male	740 (576–927)	18·2 (16·8–19·5)	160 (125–201)
Guam	Both	159 (131–191)	14·1 (12·7–15·5)	93·0 (76·5–112)
Guam	Female	46·0 (36·8–56·6)	9·48 (8·12–10·9)	55·6 (44·5–68·4)
Guam	Male	113 (93·2–136)	17·5 (16·0–19·2)	128 (106–154)
Kiribati	Both	252 (199–308)	21·9 (20·2–23·4)	213 (168–260)
Kiribati	Female	91·2 (70·6–113)	17·3 (15·4–19·2)	151 (117–187)
Kiribati	Male	161 (127–198)	25·8 (23·9–27·6)	277 (218–341)
Marshall Islands	Both	52·9 (40·8–66·5)	12·5 (11·0–14·3)	93·0 (71·7–117)
Marshall Islands	Female	10·8 (7·67–14·4)	5·96 (4·68–7·53)	38·9 (27·6–51·8)
Marshall Islands	Male	42·1 (32·6–53·5)	17·4 (15·4–19·6)	145 (112–184)
Micronesia (Federated States of)	Both	167 (118–213)	17·8 (13·4–20·4)	164 (116–208)
Micronesia (Federated States of)	Female	52·2 (38·3–68·8)	12·7 (10·1–15·2)	104 (76·4–137)
Micronesia (Federated States of)	Male	115 (77·1–146)	21·8 (15·4–24·8)	221 (148–282)
Nauru	Both	11·1 (9·02–13·6)	16·7 (14·9–18·5)	105 (85·5–129)
Nauru	Female	4·36 (3·26–5·72)	16·1 (13·8–18·5)	83·5 (62·4–110)
Nauru	Male	6·77 (5·18–8·49)	17·0 (15·2–19·1)	127 (97·2–159)
Niue	Both	2·54 (2·08–2·99)	12·7 (11·4–14·2)	152 (124–179)
Niue	Female	0·694 (0·518–0·916)	7·36 (6·05–8·95)	83·4 (62·3–110)
Niue	Male	1·84 (1·55–2·11)	17·5 (15·8–19·5)	220 (185–251)
Northern Mariana Islands	Both	59·9 (50·9–69·1)	17·1 (15·6–18·7)	141 (120–163)
Northern Mariana Islands	Female	11·5 (8·65–15·0)	8·71 (6·95–10·8)	56·0 (42·1–73·2)
Northern Mariana Islands	Male	48·4 (41·4–54·7)	22·2 (20·4–24·0)	221 (189–249)
Palau	Both	30·2 (23·5–38·3)	14·5 (13·0–16·3)	168 (130–213)
Palau	Female	6·72 (4·78–9·04)	8·16 (6·59–9·99)	82·1 (58·4–110)

Palau	Male	23·5 (18·5–29·7)	18·6 (16·7–20·8)	239 (188–302)
Papua New Guinea	Both	7150 (5460–9310)	10·2 (8·92–11·7)	72·5 (55·4–94·3)
Papua New Guinea	Female	2140 (1550–2860)	7·21 (5·92–8·70)	44·9 (32·6–60·0)
Papua New Guinea	Male	5020 (3810–6500)	12·5 (10·9–14·1)	98·2 (74·6–127)
Samoa	Both	234 (196–283)	17·1 (15·6–18·6)	111 (92·9–134)
Samoa	Female	69·3 (52·8–90·3)	10·6 (9·11–12·3)	67·5 (51·5–88·0)
Samoa	Male	164 (142–194)	23·0 (21·1–24·8)	151 (130–178)
Solomon Islands	Both	1030 (827–1250)	16·5 (15·3–17·8)	157 (126–191)
Solomon Islands	Female	256 (194–321)	9·42 (8·01–10·8)	79·7 (60·5–99·9)
Solomon Islands	Male	770 (619–939)	22·0 (20·3–23·7)	230 (185–281)
Tokelau	Both	1·45 (1·17–1·82)	13·1 (11·4–14·6)	103 (82·8–129)
Tokelau	Female	0·513 (0·371–0·703)	8·36 (6·63–10·3)	74·6 (53·9–102)
Tokelau	Male	0·938 (0·775–1·15)	18·8 (16·6–20·7)	130 (107–159)
Tonga	Both	99·8 (83·1–118)	15·2 (13·9–16·4)	97·5 (81·2–115)
Tonga	Female	17·0 (12·7–21·8)	5·89 (4·79–7·14)	33·1 (24·8–42·4)
Tonga	Male	82·8 (69·6–96·9)	22·5 (20·8–24·3)	163 (137–190)
Tuvalu	Both	16·7 (13·1–21·7)	15·6 (13·9–17·2)	142 (111–184)
Tuvalu	Female	5·22 (3·73–6·91)	10·2 (8·36–12·2)	92·0 (65·8–122)
Tuvalu	Male	11·5 (9·15–14·9)	20·4 (18·4–22·4)	187 (149–243)
Vanuatu	Both	247 (201–314)	11·0 (10·2–12·0)	83·9 (68·1–107)
Vanuatu	Female	22·7 (16·2–30·8)	2·55 (2·02–3·16)	15·6 (11·1–21·2)
Vanuatu	Male	224 (183–285)	16·6 (15·4–18·0)	150 (122–191)
Southeast Asia	Both	623000 (560000–689000)	14·2 (13·3–15·1)	92·5 (83·1–102)
Southeast Asia	Female	75800 (66500–85600)	3·88 (3·52–4·27)	22·5 (19·7–25·4)
Southeast Asia	Male	547000 (486000–610000)	22·5 (21·4–23·4)	163 (144–181)
Cambodia	Both	16500 (13300–18900)	14·8 (13·6–16·0)	99·1 (80·1–114)
Cambodia	Female	2580 (2030–3150)	4·86 (4·08–5·76)	30·5 (24·1–37·2)

Cambodia	Male	13900 (11200–15800)	24·0 (22·1–25·8)	170 (138–194)
Indonesia	Both	246000 (203000–295000)	14·4 (12·7–16·0)	94·9 (78·1–114)
Indonesia	Female	22800 (17400–28200)	2·92 (2·52–3·37)	17·7 (13·6–21·9)
Indonesia	Male	224000 (181000–272000)	24·1 (22·7–25·4)	171 (138–208)
Lao People's Democratic Republic	Both	5850 (4740–6980)	13·2 (11·8–14·4)	81·7 (66·2–97·5)
Lao People's Democratic Republic	Female	731 (543–948)	3·74 (2·97–4·66)	20·5 (15·2–26·6)
Lao People's Democratic Republic	Male	5120 (4150–6120)	20·6 (18·5–22·3)	142 (115–170)
Malaysia	Both	24600 (19700–30400)	14·0 (13·1–14·8)	78·6 (62·9–97·0)
Malaysia	Female	1880 (1450–2430)	2·59 (2·12–3·17)	12·5 (9·57–16·1)
Malaysia	Male	22700 (18100–28200)	22·0 (20·7–23·3)	140 (112–174)
Maldives	Both	205 (173–239)	13·6 (12·7–14·6)	41·1 (34·6–47·9)
Maldives	Female	24·2 (18·2–31·7)	4·09 (3·25–5·17)	12·2 (9·17–16·0)
Maldives	Male	181 (153–212)	19·9 (18·4–21·2)	60·3 (51·1–70·5)
Mauritius	Both	1040 (843–1270)	9·71 (9·12–10·3)	81·5 (66·1–99·6)
Mauritius	Female	96·2 (71·9–125)	1·97 (1·58–2·42)	14·9 (11·1–19·3)
Mauritius	Male	945 (768–1150)	16·2 (15·3–17·2)	150 (122–183)
Myanmar	Both	60000 (51900–70100)	14·3 (12·9–15·6)	110 (95·0–128)
Myanmar	Female	15400 (12800–18400)	8·02 (6·70–9·28)	54·4 (45·2–65·0)
Myanmar	Male	44600 (38700–52400)	19·6 (17·9–21·2)	170 (147–199)
Philippines	Both	95600 (77000–118000)	14·9 (13·3–16·6)	85·2 (68·7–105)
Philippines	Female	17500 (13700–22300)	6·39 (5·68–7·11)	31·7 (24·8–40·3)
Philippines	Male	78100 (60400–101000)	21·3 (20·0–22·6)	137 (106–178)
Seychelles	Both	106 (91·7–121)	13·3 (12·1–14·5)	103 (89·8–118)
Seychelles	Female	10·8 (8·30–14·0)	3·21 (2·53–4·00)	22·3 (17·1–28·9)
Seychelles	Male	94·9 (82·1–109)	20·6 (19·2–22·0)	177 (153–203)
Sri Lanka	Both	11100 (8360–14400)	8·15 (7·48–8·78)	50·7 (38·3–65·8)
Sri Lanka	Female	972 (679–1320)	1·58 (1·26–1·95)	8·61 (6·01–11·7)

Sri Lanka	Male	10100 (7620–13100)	13·6 (12·5–14·6)	95·6 (72·1–124)
Thailand	Both	62900 (47100–81500)	12·6 (11·8–13·5)	89·7 (67·2–116)
Thailand	Female	6980 (5210–9130)	3·21 (2·75–3·77)	19·4 (14·5–25·4)
Thailand	Male	55900 (41700–72700)	19·9 (18·7–21·2)	163 (122–213)
Timor-Leste	Both	928 (714–1120)	12·0 (9·88–13·9)	69·5 (53·5–83·6)
Timor-Leste	Female	83·5 (57·9–117)	2·45 (1·75–3·35)	12·7 (8·78–17·7)
Timor-Leste	Male	844 (643–1010)	19·4 (16·0–22·4)	125 (95·2–150)
Viet Nam	Both	97100 (81500–112000)	15·4 (14·4–16·3)	101 (84·6–117)
Viet Nam	Female	6620 (5080–8460)	2·44 (1·96–3·00)	13·6 (10·5–17·4)
Viet Nam	Male	90500 (76100–105000)	25·1 (23·6–26·6)	189 (159–220)
**Sub-Saharan Africa**	**Both**	**220000 (192000–251000)**	**2·87 (2·56–3·19)**	**20·4 (17·8–23·3)**
**Sub-Saharan Africa**	**Female**	**35600 (30300–41200)**	**1·02 (0·874–1·15)**	**6·50 (5·53–7·54)**
**Sub-Saharan Africa**	**Male**	**184000 (161000–210000)**	**4·44 (3·94–4·95)**	**34·7 (30·3–39·6)**
Central Sub-Saharan Africa	Both	27400 (21800–34200)	3·13 (2·72–3·57)	20·8 (16·5–26·0)
Central Sub-Saharan Africa	Female	2930 (2170–3880)	0·727 (0·580–0·897)	4·43 (3·28–5·86)
Central Sub-Saharan Africa	Male	24400 (19400–30600)	5·20 (4·45–5·92)	37·4 (29·7–46·8)
Angola	Both	7510 (6100–9400)	4·06 (3·66–4·50)	24·9 (20·2–31·2)
Angola	Female	949 (672–1300)	1·12 (0·838–1·43)	6·12 (4·33–8·36)
Angola	Male	6560 (5330–8190)	6·56 (5·89–7·27)	44·9 (36·4–56·0)
Central African Republic	Both	1970 (1480–2580)	2·91 (2·48–3·40)	37·3 (28·0–48·7)
Central African Republic	Female	170 (110–254)	0·566 (0·413–0·758)	6·30 (4·07–9·40)
Central African Republic	Male	1800 (1350–2350)	4·77 (4·08–5·59)	69·5 (52·1–90·7)
Congo	Both	1430 (1130–1790)	4·01 (3·45–4·51)	27·2 (21·4–34·0)
Congo	Female	129 (90·1–182)	0·738 (0·558–0·959)	4·84 (3·39–6·85)
Congo	Male	1300 (1030–1630)	7·11 (6·11–8·03)	50·0 (39·3–62·3)
Democratic Republic of the Congo	Both	15700 (11900–20300)	2·78 (2·28–3·30)	17·9 (13·6–23·2)
Democratic Republic of the Congo	Female	1620 (1130–2260)	0·619 (0·461–0·818)	3·71 (2·57–5·16)

Democratic Republic of the Congo	Male	14100 (10500–18300)	4·65 (3·75–5·58)	32·1 (24·0–41·6)
Equatorial Guinea	Both	192 (137–268)	2·52 (1·91–3·14)	13·5 (9·65–18·8)
Equatorial Guinea	Female	18·4 (11·2–29·3)	0·488 (0·328–0·702)	2·83 (1·72–4·49)
Equatorial Guinea	Male	173 (124–241)	4·52 (3·50–5·56)	22·6 (16·2–31·4)
Gabon	Both	524 (410–643)	4·45 (3·76–5·16)	29·9 (23·4–36·8)
Gabon	Female	42·6 (29·5–60·0)	0·816 (0·604–1·10)	4·71 (3·26–6·64)
Gabon	Male	481 (378–587)	7·33 (6·25–8·44)	56·9 (44·7–69·4)
Eastern Sub-Saharan Africa	Both	82600 (71800–95400)	3·20 (2·86–3·59)	20·1 (17·4–23·2)
Eastern Sub-Saharan Africa	Female	13800 (11500–16400)	1·19 (0·994–1·39)	6·64 (5·53–7·93)
Eastern Sub-Saharan Africa	Male	68900 (59400–79900)	4·83 (4·28–5·44)	33·7 (29·1–39·1)
Burundi	Both	2780 (2080–3630)	3·33 (2·78–3·93)	23·3 (17·4–30·4)
Burundi	Female	345 (235–496)	0·958 (0·723–1·25)	5·75 (3·91–8·27)
Burundi	Male	2430 (1810–3170)	5·12 (4·26–6·02)	41·0 (30·6–53·5)
Comoros	Both	250 (195–317)	4·99 (4·08–5·86)	35·0 (27·3–44·4)
Comoros	Female	32·5 (22·1–45·0)	1·31 (0·934–1·74)	9·08 (6·19–12·6)
Comoros	Male	217 (170–275)	8·62 (7·08–10·1)	61·0 (47·5–77·1)
Djibouti	Both	491 (363–665)	6·48 (5·45–7·55)	40·8 (30·1–55·3)
Djibouti	Female	43·5 (29·6–63·0)	1·36 (1·00–1·79)	7·71 (5·25–11·2)
Djibouti	Male	448 (329–606)	10·2 (8·65–11·8)	70·0 (51·5–94·9)
Eritrea	Both	1470 (1030–1940)	3·38 (2·65–4·10)	21·8 (15·3–28·9)
Eritrea	Female	44·2 (28·6–66·1)	0·224 (0·157–0·321)	1·32 (0·857–1·98)
Eritrea	Male	1420 (991–1870)	6·01 (4·69–7·27)	42·1 (29·4–55·4)
Ethiopia	Both	8120 (6560–9920)	1·45 (1·19–1·77)	7·55 (6·10–9·22)
Ethiopia	Female	486 (342–663)	0·201 (0·146–0·269)	0·912 (0·642–1·24)
Ethiopia	Male	7640 (6050–9450)	2·40 (2·00–2·89)	14·1 (11·1–17·4)
Kenya	Both	10200 (8350–12300)	3·49 (2·95–4·08)	20·4 (16·6–24·4)
Kenya	Female	1230 (936–1620)	0·929 (0·726–1·16)	4·91 (3·72–6·44)

Kenya	Male	9010 (7170–11000)	5·59 (4·76–6·44)	36·0 (28·6–44·0)
Madagascar	Both	5410 (4020–7070)	3·29 (2·77–3·83)	20·3 (15·1–26·5)
Madagascar	Female	569 (387–806)	0·732 (0·546–0·949)	4·27 (2·90–6·04)
Madagascar	Male	4840 (3580–6360)	5·58 (4·70–6·52)	36·3 (26·8–47·6)
Malawi	Both	4750 (3730–5930)	4·07 (3·37–4·77)	25·7 (20·2–32·1)
Malawi	Female	560 (400–772)	1·07 (0·795–1·43)	5·92 (4·23–8·15)
Malawi	Male	4190 (3310–5210)	6·50 (5·36–7·57)	46·7 (36·9–58·1)
Mozambique	Both	8060 (6190–10300)	3·04 (2·48–3·66)	27·3 (21·0–35·0)
Mozambique	Female	1070 (736–1520)	0·901 (0·640–1·20)	6·99 (4·80–9·94)
Mozambique	Male	6990 (5410–8870)	4·78 (3·89–5·76)	49·2 (38·1–62·4)
Rwanda	Both	4960 (4020–6020)	7·24 (6·07–8·41)	39·1 (31·6–47·5)
Rwanda	Female	1920 (1510–2380)	5·76 (4·61–6·98)	29·4 (23·2–36·6)
Rwanda	Male	3040 (2450–3760)	8·64 (7·22–10·2)	49·3 (39·7–61·0)
Somalia	Both	5260 (3770–7060)	2·85 (2·28–3·48)	25·8 (18·5–34·7)
Somalia	Female	643 (402–971)	0·767 (0·515–1·08)	6·45 (4·03–9·75)
Somalia	Male	4610 (3310–6180)	4·59 (3·70–5·62)	44·5 (31·9–59·5)
South Sudan	Both	1920 (1370–2580)	2·64 (2·06–3·28)	20·7 (14·8–27·8)
South Sudan	Female	177 (116–260)	0·560 (0·381–0·785)	3·83 (2·50–5·62)
South Sudan	Male	1740 (1250–2340)	4·23 (3·29–5·28)	37·4 (26·8–50·2)
Uganda	Both	6270 (4990–7720)	2·58 (2·16–3·09)	15·3 (12·1–18·8)
Uganda	Female	1330 (1010–1720)	1·25 (0·955–1·61)	6·38 (4·81–8·23)
Uganda	Male	4940 (3920–6080)	3·64 (3·01–4·35)	24·4 (19·4–30·1)
United Republic of Tanzania	Both	17900 (14700–21600)	5·06 (4·35–5·73)	31·6 (26·0–38·1)
United Republic of Tanzania	Female	4310 (3400–5320)	2·60 (2·10–3·11)	14·8 (11·6–18·2)
United Republic of Tanzania	Male	13600 (11100–16400)	7·21 (6·17–8·23)	49·3 (40·1–59·6)
Zambia	Both	4670 (3600–5810)	3·79 (3·14–4·46)	25·6 (19·7–31·8)
Zambia	Female	1000 (749–1340)	1·88 (1·42–2·37)	10·9 (8·12–14·5)

Zambia	Male	3670 (2820–4560)	5·23 (4·33–6·14)	40·7 (31·3–50·6)
Southern Sub-Saharan Africa	Both	47000 (42700–51300)	6·42 (5·91–6·92)	59·8 (54·3–65·3)
Southern Sub-Saharan Africa	Female	10900 (9640–12300)	3·19 (2·84–3·58)	27·2 (24·0–30·7)
Southern Sub-Saharan Africa	Male	36100 (32700–39500)	9·25 (8·45–10·1)	94·0 (85·3–103)
Botswana	Both	1590 (1200–1990)	7·49 (6·54–8·41)	67·9 (51·4–85·2)
Botswana	Female	335 (238–467)	3·51 (2·78–4·31)	28·2 (20·1–39·3)
Botswana	Male	1250 (954–1550)	10·8 (9·26–12·3)	109 (82·9–135)
Eswatini	Both	362 (279–473)	3·12 (2·68–3·60)	31·7 (24·4–41·4)
Eswatini	Female	84·2 (51·1–125)	1·71 (1·23–2·27)	14·4 (8·75–21·3)
Eswatini	Male	278 (217–352)	4·16 (3·47–4·85)	49·7 (39·0–63·1)
Lesotho	Both	2220 (1740–2750)	6·81 (5·89–7·74)	106 (83·1–131)
Lesotho	Female	267 (171–396)	1·81 (1·31–2·49)	25·3 (16·1–37·4)
Lesotho	Male	1950 (1550–2410)	11·0 (9·43–12·6)	189 (150–233)
Namibia	Both	1150 (933–1420)	6·08 (5·46–6·78)	47·8 (38·8–59·0)
Namibia	Female	415 (308–546)	4·94 (4·11–5·84)	33·5 (24·9–44·0)
Namibia	Male	735 (601–886)	7·00 (6·17–7·93)	63·2 (51·6–76·2)
South Africa	Both	32800 (29900–35800)	6·29 (5·76–6·83)	59·1 (53·8–64·5)
South Africa	Female	8260 (7180–9390)	3·36 (2·94–3·82)	29·1 (25·3–33·1)
South Africa	Male	24600 (22000–27000)	8·90 (8·00–9·75)	90·3 (80·9–99·0)
Zimbabwe	Both	8840 (7010–10700)	6·98 (6·04–8·00)	58·9 (46·7–71·2)
Zimbabwe	Female	1570 (1130–2120)	2·65 (2·08–3·35)	20·2 (14·5–27·2)
Zimbabwe	Male	7260 (5860–8680)	10·8 (9·32–12·4)	101 (81·3–120)
Western Sub-Saharan Africa	Both	62600 (52400–74100)	1·81 (1·54–2·08)	13·7 (11·5–16·2)
Western Sub-Saharan Africa	Female	7910 (6310–9840)	0·496 (0·405–0·593)	3·39 (2·70–4·22)
Western Sub-Saharan Africa	Male	54700 (45800–65000)	2·94 (2·50–3·36)	24·5 (20·5–29·2)
Benin	Both	1940 (1480–2520)	2·08 (1·75–2·41)	15·3 (11·7–19·9)
Benin	Female	255 (174–350)	0·600 (0·442–0·793)	3·96 (2·70–5·43)

Benin	Male	1680 (1280–2160)	3·33 (2·78–3·84)	27·0 (20·6–34·8)
Burkina Faso	Both	2990 (2330–3800)	1·48 (1·18–1·81)	13·2 (10·3–16·7)
Burkina Faso	Female	194 (133–270)	0·209 (0·146–0·283)	1·67 (1·14–2·31)
Burkina Faso	Male	2800 (2180–3540)	2·58 (2·04–3·14)	25·3 (19·8–32·1)
Cabo Verde	Both	157 (136–180)	4·48 (4·04–4·91)	27·8 (24·1–31·9)
Cabo Verde	Female	25·5 (20·1–31·8)	1·67 (1·34–2·05)	9·13 (7·17–11·4)
Cabo Verde	Male	131 (113–149)	6·64 (5·99–7·30)	46·3 (39·7–52·6)
Cameroon	Both	5410 (4150–7150)	2·61 (2·18–3·04)	18·6 (14·3–24·6)
Cameroon	Female	357 (246–507)	0·376 (0·272–0·497)	2·44 (1·68–3·47)
Cameroon	Male	5060 (3860–6630)	4·49 (3·72–5·28)	34·9 (26·6–45·7)
Chad	Both	2970 (2260–3840)	1·90 (1·50–2·35)	18·1 (13·8–23·4)
Chad	Female	351 (243–492)	0·500 (0·347–0·689)	4·25 (2·94–5·96)
Chad	Male	2620 (2010–3370)	3·04 (2·39–3·78)	32·2 (24·7–41·4)
Côte d'Ivoire	Both	6480 (4930–8290)	3·55 (2·87–4·19)	24·8 (18·8–31·7)
Côte d'Ivoire	Female	894 (630–1220)	1·16 (0·831–1·54)	7·06 (4·98–9·66)
Côte d'Ivoire	Male	5590 (4230–7140)	5·29 (4·27–6·26)	41·4 (31·3–52·9)
Gambia	Both	611 (472–756)	4·52 (3·67–5·24)	27·2 (21·0–33·7)
Gambia	Female	33·3 (23·0–46·7)	0·538 (0·400–0·737)	2·92 (2·02–4·09)
Gambia	Male	578 (444–716)	7·90 (6·41–9·12)	52·3 (40·2–64·8)
Ghana	Both	5480 (4370–6770)	2·63 (2·20–3·06)	17·4 (13·8–21·5)
Ghana	Female	816 (615–1080)	0·848 (0·650–1·07)	5·03 (3·79–6·68)
Ghana	Male	4660 (3670–5750)	4·16 (3·44–4·89)	30·4 (24·0–37·6)
Guinea	Both	4250 (3310–5300)	3·72 (3·23–4·20)	33·6 (26·2–41·9)
Guinea	Female	382 (260–536)	0·720 (0·524–0·965)	5·86 (3·99–8·23)
Guinea	Male	3870 (3010–4870)	6·33 (5·49–7·14)	63·1 (49·1–79·4)
Guinea-Bissau	Both	301 (236–377)	2·03 (1·77–2·32)	15·9 (12·4–19·8)
Guinea-Bissau	Female	31·0 (21·6–43·8)	0·465 (0·345–0·612)	3·18 (2·21–4·48)

Guinea-Bissau	Male	270 (213–337)	3·32 (2·83–3·80)	29·2 (23·0–36·4)
Liberia	Both	675 (506–895)	2·27 (1·77–2·78)	14·1 (10·6–18·7)
Liberia	Female	98·5 (67·6–137)	0·705 (0·513–0·923)	4·14 (2·84–5·74)
Liberia	Male	577 (429–764)	3·65 (2·85–4·51)	23·9 (17·8–31·7)
Mali	Both	3600 (2800–4580)	1·79 (1·54–2·06)	16·4 (12·8–20·9)
Mali	Female	421 (298–579)	0·449 (0·331–0·586)	3·81 (2·70–5·25)
Mali	Male	3180 (2470–4080)	2·96 (2·52–3·44)	29·2 (22·7–37·5)
Mauritania	Both	703 (514–898)	3·35 (2·57–3·96)	17·5 (12·8–22·4)
Mauritania	Female	131 (87·6–181)	1·28 (0·912–1·68)	6·43 (4·29–8·86)
Mauritania	Male	572 (419–732)	5·33 (4·08–6·32)	29·0 (21·2–37·1)
Niger	Both	1980 (1470–2570)	0·978 (0·789–1·15)	8·51 (6·32–11·0)
Niger	Female	203 (139–296)	0·214 (0·153–0·290)	1·73 (1·18–2·52)
Niger	Male	1780 (1320–2310)	1·65 (1·33–1·94)	15·4 (11·4–20·0)
Nigeria	Both	17400 (13000–23200)	1·09 (0·822–1·41)	8·08 (6·05–10·8)
Nigeria	Female	2720 (1830–4070)	0·364 (0·253–0·502)	2·44 (1·64–3·65)
Nigeria	Male	14600 (10500–20400)	1·72 (1·31–2·22)	14·2 (10·2–19·8)
São Tomé and Príncipe	Both	43·6 (35·5–51·6)	4·32 (3·82–4·87)	21·2 (17·3–25·1)
São Tomé and Príncipe	Female	6·29 (4·59–8·34)	1·34 (1·02–1·70)	6·15 (4·48–8·15)
São Tomé and Príncipe	Male	37·3 (30·3–44·5)	6·92 (6·07–7·82)	36·2 (29·4–43·2)
Senegal	Both	3110 (2400–3930)	3·46 (2·77–4·08)	20·5 (15·9–26·0)
Senegal	Female	219 (143–314)	0·524 (0·357–0·727)	2·90 (1·89–4·17)
Senegal	Male	2890 (2220–3660)	5·99 (4·79–7·08)	38·1 (29·3–48·2)
Sierra Leone	Both	2220 (1680–2910)	3·17 (2·62–3·69)	26·9 (20·3–35·2)
Sierra Leone	Female	375 (268–520)	1·14 (0·863–1·45)	8·96 (6·41–12·4)
Sierra Leone	Male	1850 (1390–2410)	4·97 (4·08–5·83)	45·1 (34·0–58·8)
Togo	Both	2290 (1790–2930)	4·28 (3·60–5·00)	28·9 (22·5–37·0)
Togo	Female	403 (290–546)	1·63 (1·20–2·11)	9·98 (7·18–13·5)

Togo	Male	1890<br>(1460–2430)	6·55<br>(5·45–7·64)	48·7<br>(37·5–62·6)

Supplemental Table S6. All-cause and all-age smoking attributable disability-adjusted life-years (DALYs) by location and sex, 2019. Estimates are reported as total number of attributable DALYs, percent of DALYs attributable to smoking, and smoking attributable DALY rate (per 100,000).

Location	Sex	Number	Percent	Rate
**Global**	**Both**	**200000000 (185000000–214000000)**	**7·89 (7·19–8·56)**	**2580 (2400–2760)**
**Global**	**Female**	**37900000 (34900000–41000000)**	**3·19 (2·88–3·48)**	**983 (904–1060)**
**Global**	**Male**	**162000000 (149000000–175000000)**	**12·0 (11·1–13·0)**	**4170 (3830–4510)**
**Central Europe, Eastern Europe, and Central Asia**	**Both**	**21700000 (19900000–23600000)**	**13·8 (12·7–14·8)**	**5190 (4770–5660)**
**Central Europe, Eastern Europe, and Central Asia**	**Female**	**4430000 (3980000–4940000)**	**6·14 (5·55–6·68)**	**2030 (1830–2260)**
**Central Europe, Eastern Europe, and Central Asia**	**Male**	**17300000 (15700000–18900000)**	**20·2 (19·0–21·3)**	**8650 (7870–9470)**
Central Asia	Both	2560000 (2340000–2810000)	8·99 (8·27–9·65)	2730 (2500–3000)
Central Asia	Female	240000 (210000–271000)	1·87 (1·63–2·11)	508 (446–575)
Central Asia	Male	2320000 (2110000–2550000)	14·9 (13·9–15·7)	5000 (4550–5500)
Armenia	Both	128000 (109000–147000)	13·9 (12·5–15·2)	4240 (3610–4880)
Armenia	Female	7310 (5780–9180)	1·67 (1·36–2·05)	468 (370–587)
Armenia	Male	121000 (103000–139000)	24·9 (22·7–26·8)	8280 (7050–9560)
Azerbaijan	Both	369000 (307000–444000)	11·7 (10·3–13·2)	3590 (2990–4320)
Azerbaijan	Female	15200 (11200–20000)	1·06 (0·797–1·37)	296 (218–390)
Azerbaijan	Male	354000 (292000–430000)	20·6 (18·8–22·3)	6880 (5680–8360)
Georgia	Both	196000 (169000–226000)	13·5 (12·3–14·7)	5350 (4610–6160)
Georgia	Female	19900 (16500–24100)	3·05 (2·59–3·56)	1040 (864–1260)
Georgia	Male	176000 (152000–203000)	22·2 (20·6–23·8)	10000 (8640–11500)
Kazakhstan	Both	603000 (528000–688000)	10·4 (9·35–11·6)	3280 (2870–3740)
Kazakhstan	Female	71200 (59400–84900)	2·76 (2·32–3·25)	750 (627–895)
Kazakhstan	Male	532000 (462000–609000)	16·7 (15·2–18·2)	5970 (5190–6840)
Kyrgyzstan	Both	151000 (132000–171000)	9·13 (8·09–10·1)	2300 (2020–2620)

Kyrgyzstan	Female	18100 (14900–21700)	2·45 (1·99–2·96)	548 (453–658)
Kyrgyzstan	Male	133000 (117000–150000)	14·6 (13·2–15·8)	4100 (3620–4630)
Mongolia	Both	117000 (92800–150000)	10·0 (8·90–11·1)	3470 (2740–4440)
Mongolia	Female	13900 (10700–18000)	2·91 (2·38–3·43)	810 (625–1050)
Mongolia	Male	104000 (81800–132000)	14·9 (13·5–16·4)	6190 (4900–7920)
Tajikistan	Both	143000 (116000–177000)	5·33 (4·62–6·08)	1510 (1230–1860)
Tajikistan	Female	15000 (11300–19900)	1·24 (0·933–1·62)	320 (240–423)
Tajikistan	Male	128000 (104000–158000)	8·71 (7·59–9·90)	2670 (2170–3300)
Turkmenistan	Both	134000 (107000–165000)	8·44 (7·51–9·32)	2640 (2110–3240)
Turkmenistan	Female	18100 (13400–23700)	2·61 (2·05–3·25)	727 (540–951)
Turkmenistan	Male	116000 (93400–142000)	12·9 (11·6–14·2)	4470 (3600–5480)
Uzbekistan	Both	715000 (604000–838000)	7·11 (6·36–7·84)	2120 (1790–2490)
Uzbekistan	Female	61000 (46700–78900)	1·32 (1·03–1·69)	362 (277–468)
Uzbekistan	Male	655000 (551000–768000)	12·0 (11·0–13·1)	3890 (3280–4570)
Central Europe	Both	6600000 (5840000–7450000)	16·3 (14·9–17·6)	5780 (5110–6520)
Central Europe	Female	2030000 (1790000–2310000)	10·8 (9·78–11·8)	3470 (3050–3950)
Central Europe	Male	4570000 (3990000–5160000)	21·0 (19·4–22·5)	8220 (7170–9270)
Albania	Both	104000 (81300–130000)	13·6 (11·7–15·3)	3830 (2990–4790)
Albania	Female	18400 (14400–23400)	5·35 (4·34–6·44)	1350 (1060–1730)
Albania	Male	85800 (66600–109000)	20·3 (18·0–22·6)	6300 (4890–7970)
Bosnia and Herzegovina	Both	225000 (183000–274000)	19·5 (17·6–21·5)	6810 (5560–8290)
Bosnia and Herzegovina	Female	73600 (60000–88800)	13·4 (11·7–15·0)	4360 (3550–5250)
Bosnia and Herzegovina	Male	151000 (123000–184000)	25·2 (22·8–27·4)	9390 (7630–11400)
Bulgaria	Both	572000 (463000–703000)	17·6 (16·1–18·9)	8250 (6670–10100)
Bulgaria	Female	159000 (128000–194000)	10·7 (9·50–11·8)	4460 (3600–5450)
Bulgaria	Male	413000 (334000–506000)	23·4 (21·7–25·0)	12300 (9930–15000)
Croatia	Both	239000 (197000–290000)	16·2 (14·6–17·8)	5620 (4630–6830)

Croatia	Female	76800 (63500–92700)	10·9 (9·64–12·1)	3510 (2900–4230)
Croatia	Male	162000 (132000–198000)	21·1 (19·1–23·0)	7870 (6430–9610)
Czechia	Both	529000 (444000–628000)	15·3 (13·7–16·8)	4970 (4170–5900)
Czechia	Female	171000 (144000–201000)	10·5 (9·29–11·7)	3160 (2660–3710)
Czechia	Male	358000 (299000–423000)	19·6 (17·7–21·3)	6830 (5700–8080)
Hungary	Both	672000 (566000–798000)	18·1 (16·5–19·8)	6950 (5850–8250)
Hungary	Female	240000 (202000–282000)	13·4 (12·0–14·8)	4740 (3980–5570)
Hungary	Male	432000 (362000–513000)	22·6 (20·8–24·4)	9370 (7860–11100)
Montenegro	Both	46000 (39500–53800)	22·0 (20·0–24·1)	7420 (6360–8680)
Montenegro	Female	16300 (14100–18600)	16·9 (14·8–18·9)	5180 (4500–5940)
Montenegro	Male	29800 (25000–35300)	26·4 (24·0–28·7)	9720 (8140–11500)
North Macedonia	Both	144000 (117000–175000)	19·2 (17·2–21·1)	6680 (5430–8150)
North Macedonia	Female	44600 (36100–54800)	12·9 (11·3–14·5)	4210 (3400–5170)
North Macedonia	Male	99200 (79900–122000)	24·6 (22·3–26·7)	9080 (7310–11200)
Poland	Both	2060000 (1740000–2430000)	16·3 (14·8–17·9)	5370 (4530–6320)
Poland	Female	671000 (551000–812000)	11·7 (10·3–13·0)	3380 (2780–4100)
Poland	Male	1390000 (1120000–1700000)	20·1 (18·3–21·9)	7480 (6010–9130)
Romania	Both	1080000 (902000–1270000)	14·5 (13·2–15·6)	5610 (4690–6620)
Romania	Female	260000 (214000–307000)	7·65 (6·79–8·53)	2640 (2170–3110)
Romania	Male	818000 (684000–976000)	20·2 (18·7–21·6)	8740 (7310–10400)
Serbia	Both	625000 (513000–756000)	19·1 (17·3–20·7)	7140 (5860–8640)
Serbia	Female	219000 (181000–267000)	14·0 (12·5–15·5)	4960 (4100–6040)
Serbia	Male	406000 (331000–493000)	23·6 (21·5–25·6)	9360 (7630–11400)
Slovakia	Both	228000 (184000–279000)	12·9 (11·5–14·3)	4190 (3390–5130)
Slovakia	Female	57500 (46400–70400)	7·02 (6·10–7·91)	2070 (1670–2530)
Slovakia	Male	170000 (137000–208000)	18·1 (16·2–19·9)	6410 (5180–7830)
Slovenia	Both	80300 (64600–99100)	12·8 (11·4–14·3)	3870 (3120–4780)

Slovenia	Female	25100 (20200–30700)	8·58 (7·57–9·63)	2410 (1930–2940)
Slovenia	Male	55100 (44200–68600)	16·6 (14·7–18·3)	5360 (4300–6670)
Eastern Europe	Both	12500000 (11100000–14000000)	14·1 (12·9–15·3)	5970 (5300–6670)
Eastern Europe	Female	2160000 (1870000–2490000)	5·32 (4·77–5·83)	1920 (1660–2220)
Eastern Europe	Male	10400000 (9030000–11800000)	21·5 (20·2–22·7)	10600 (9250–12100)
Belarus	Both	563000 (452000–695000)	15·1 (13·5–16·4)	5930 (4760–7320)
Belarus	Female	90400 (71300–114000)	5·26 (4·49–6·07)	1780 (1410–2250)
Belarus	Male	473000 (379000–583000)	23·4 (21·5–24·9)	10700 (8560–13200)
Estonia	Both	55700 (44800–68300)	12·2 (10·9–13·6)	4240 (3410–5210)
Estonia	Female	16200 (13300–19700)	7·20 (6·35–8·26)	2340 (1910–2840)
Estonia	Male	39400 (31500–48500)	17·1 (15·5–18·9)	6380 (5090–7840)
Latvia	Both	96700 (81000–116000)	12·6 (11·2–13·9)	5050 (4230–6030)
Latvia	Female	22800 (18100–28600)	5·94 (5·20–6·76)	2210 (1750–2770)
Latvia	Male	74000 (59500–91300)	19·2 (17·6–20·8)	8380 (6740–10300)
Lithuania	Both	128000 (107000–153000)	11·7 (10·5–12·8)	4590 (3820–5490)
Lithuania	Female	25000 (20700–30000)	4·71 (4·11–5·35)	1660 (1370–1990)
Lithuania	Male	103000 (86100–124000)	18·3 (16·7–19·7)	8020 (6680–9610)
Republic of Moldova	Both	171000 (149000–194000)	12·5 (11·3–13·7)	4620 (4030–5250)
Republic of Moldova	Female	19900 (15800–24500)	3·14 (2·53–3·84)	1030 (820–1270)
Republic of Moldova	Male	151000 (132000–170000)	20·6 (19·0–22·2)	8560 (7500–9650)
Russian Federation	Both	8330000 (7150000–9670000)	13·9 (12·6–15·4)	5680 (4870–6590)
Russian Federation	Female	1520000 (1280000–1800000)	5·51 (4·90–6·06)	1940 (1630–2300)
Russian Federation	Male	6810000 (5700000–8070000)	21·1 (19·7–22·6)	9970 (8350–11800)
Ukraine	Both	3180000 (2650000–3750000)	14·7 (13·1–16·3)	7220 (6030–8520)
Ukraine	Female	459000 (364000–572000)	4·87 (4·23–5·58)	1930 (1530–2410)
Ukraine	Male	2720000 (2220000–3300000)	22·4 (20·8–23·7)	13400 (10900–16300)
**High-income**	**Both**	**39000000 (36500000–41700000)**	**12·1 (10·9–13·2)**	**3600 (3370–3850)**

**High-income**	**Female**	**14300000 (13100000–15600000)**	**8·96 (7·99–9·88)**	**2590 (2370–2820)**
**High-income**	**Male**	**24800000 (23400000–26300000)**	**15·1 (13·9–16·3)**	**4650 (4380–4930)**
Australasia	Both	629000 (580000–684000)	8·41 (7·53–9·26)	2160 (2000–2350)
Australasia	Female	271000 (245000–299000)	7·40 (6·49–8·29)	1840 (1660–2030)
Australasia	Male	358000 (332000–386000)	9·39 (8·42–10·3)	2500 (2320–2700)
Australia	Both	513000 (471000–560000)	8·18 (7·32–9·04)	2090 (1920–2280)
Australia	Female	219000 (196000–244000)	7·15 (6·28–8·04)	1760 (1580–1960)
Australia	Male	294000 (271000–318000)	9·16 (8·23–10·1)	2420 (2230–2630)
New Zealand	Both	116000 (108000–125000)	9·59 (8·50–10·6)	2590 (2390–2790)
New Zealand	Female	52100 (47300–57100)	8·62 (7·57–9·61)	2260 (2050–2470)
New Zealand	Male	64200 (59600–69100)	10·6 (9·45–11·6)	2930 (2730–3160)
High-income Asia Pacific	Both	5590000 (5170000–6050000)	11·2 (10·0–12·4)	2990 (2760–3230)
High-income Asia Pacific	Female	1060000 (933000–1220000)	4·39 (3·83–5·03)	1120 (983–1290)
High-income Asia Pacific	Male	4530000 (4210000–4870000)	17·6 (16·0–19·1)	4900 (4560–5270)
Brunei Darussalam	Both	8390 (7330–9630)	8·58 (7·54–9·70)	1920 (1680–2200)
Brunei Darussalam	Female	1740 (1400–2170)	3·85 (3·07–4·83)	835 (674–1040)
Brunei Darussalam	Male	6650 (5700–7720)	12·7 (11·4–14·1)	2900 (2490–3370)
Japan	Both	4210000 (3870000–4590000)	11·6 (10·4–12·7)	3300 (3030–3590)
Japan	Female	877000 (763000–1020000)	4·90 (4·26–5·63)	1340 (1160–1550)
Japan	Male	3340000 (3100000–3590000)	18·0 (16·4–19·5)	5360 (4970–5760)
Republic of Korea	Both	1290000 (1180000–1410000)	10·5 (9·26–11·7)	2420 (2210–2640)
Republic of Korea	Female	169000 (143000–200000)	2·94 (2·43–3·49)	641 (543–758)
Republic of Korea	Male	1120000 (1030000–1220000)	17·1 (15·4–18·7)	4160 (3800–4520)
Singapore	Both	79000 (71900–86000)	7·58 (6·56–8·57)	1390 (1270–1520)
Singapore	Female	15100 (12700–18000)	3·00 (2·45–3·58)	545 (459–648)
Singapore	Male	63900 (58300–69700)	11·8 (10·4–13·2)	2210 (2010–2410)
High-income North America	Both	15100000 (14100000–16200000)	12·5 (11·4–13·6)	4150 (3880–4440)

High-income North America	Female	6560000 (6030000–7100000)	11·1 (9·95–12·3)	3540 (3260–3830)
High-income North America	Male	8580000 (8070000–9090000)	13·9 (12·7–15·0)	4780 (4500–5070)
Canada	Both	1190000 (1100000–1270000)	11·9 (10·6–13·1)	3250 (3010–3480)
Canada	Female	507000 (461000–554000)	10·3 (8·98–11·6)	2730 (2480–2990)
Canada	Male	679000 (633000–726000)	13·5 (12·3–14·7)	3780 (3520–4040)
Greenland	Both	3590 (2960–4220)	17·8 (16·2–19·6)	6380 (5260–7520)
Greenland	Female	1410 (1180–1680)	16·7 (14·6–18·9)	5310 (4430–6330)
Greenland	Male	2170 (1780–2570)	18·6 (17·0–20·4)	7340 (6020–8680)
United States of America	Both	14000000 (13000000–14900000)	12·6 (11·5–13·6)	4250 (3970–4550)
United States of America	Female	6050000 (5560000–6560000)	11·2 (10·0–12·4)	3630 (3330–3930)
United States of America	Male	7900000 (7430000–8380000)	13·9 (12·8–15·0)	4900 (4610–5200)
Southern Latin America	Both	1790000 (1680000–1910000)	9·89 (8·93–10·8)	2690 (2520–2860)
Southern Latin America	Female	697000 (633000–763000)	7·98 (7·10–8·85)	2040 (1850–2230)
Southern Latin America	Male	1100000 (1030000–1170000)	11·7 (10·7–12·6)	3370 (3180–3580)
Argentina	Both	1330000 (1240000–1410000)	10·6 (9·52–11·5)	2940 (2760–3130)
Argentina	Female	513000 (464000–560000)	8·53 (7·56–9·55)	2220 (2010–2420)
Argentina	Male	815000 (766000–863000)	12·4 (11·4–13·4)	3700 (3480–3920)
Chile	Both	342000 (313000–375000)	7·58 (6·84–8·27)	1880 (1720–2060)
Chile	Female	145000 (128000–164000)	6·57 (5·79–7·37)	1570 (1380–1770)
Chile	Male	197000 (180000–215000)	8·56 (7·78–9·28)	2200 (2020–2400)
Uruguay	Both	124000 (117000–132000)	11·7 (10·6–12·7)	3620 (3400–3850)
Uruguay	Female	39300 (35600–43200)	7·63 (6·76–8·51)	2200 (1990–2420)
Uruguay	Male	85100 (80300–90100)	15·6 (14·4–16·8)	5170 (4870–5470)
Western Europe	Both	15900000 (14800000–17000000)	12·5 (11·4–13·7)	3640 (3390–3900)
Western Europe	Female	5660000 (5140000–6230000)	8·90 (7·97–9·79)	2550 (2320–2810)
Western Europe	Male	10200000 (9610000–10800000)	16·2 (14·8–17·5)	4760 (4480–5050)
Andorra	Both	2820 (2260–3460)	12·9 (11·1–14·7)	3400 (2720–4160)

Andorra	Female	770 (590–977)	7·63 (6·26–9·15)	1900 (1460–2420)
Andorra	Male	2050 (1640–2490)	17·4 (15·2–19·5)	4820 (3850–5850)
Austria	Both	312000 (288000–337000)	12·3 (11·2–13·5)	3500 (3230–3780)
Austria	Female	114000 (103000–127000)	9·04 (8·06–10·0)	2520 (2270–2800)
Austria	Male	198000 (185000–212000)	15·6 (14·2–17·0)	4500 (4200–4820)
Belgium	Both	460000 (429000–495000)	13·6 (12·3–14·9)	4030 (3760–4330)
Belgium	Female	164000 (148000–181000)	9·69 (8·64–10·8)	2830 (2560–3130)
Belgium	Male	296000 (278000–315000)	17·5 (15·9–19·0)	5270 (4960–5600)
Cyprus	Both	41400 (37100–46400)	12·9 (11·6–14·3)	3150 (2830–3530)
Cyprus	Female	10800 (9230–12500)	6·84 (5·94–7·77)	1620 (1390–1890)
Cyprus	Male	30600 (27700–34200)	18·8 (17·1–20·6)	4720 (4260–5270)
Denmark	Both	276000 (257000–297000)	16·3 (14·7–17·8)	4760 (4430–5120)
Denmark	Female	124000 (113000–135000)	14·7 (13·1–16·4)	4270 (3890–4640)
Denmark	Male	152000 (141000–163000)	17·9 (16·3–19·4)	5260 (4900–5650)
Finland	Both	166000 (152000–180000)	9·93 (8·96–10·9)	2990 (2750–3250)
Finland	Female	56200 (50300–63200)	6·93 (6·18–7·73)	2000 (1790–2260)
Finland	Male	109000 (101000–118000)	12·8 (11·6–13·8)	4010 (3710–4310)
France	Both	1980000 (1810000–2130000)	11·0 (9·85–12·1)	2990 (2740–3220)
France	Female	640000 (566000–722000)	7·07 (6·30–7·89)	1880 (1660–2120)
France	Male	1340000 (1240000–1430000)	14·9 (13·6–16·3)	4170 (3880–4440)
Germany	Both	3550000 (3290000–3830000)	13·0 (11·7–14·2)	4180 (3870–4510)
Germany	Female	1280000 (1160000–1420000)	9·43 (8·40–10·4)	3010 (2710–3330)
Germany	Male	2270000 (2120000–2430000)	16·6 (15·1–17·9)	5370 (5030–5750)
Greece	Both	595000 (557000–637000)	17·6 (16·0–19·2)	5750 (5390–6160)
Greece	Female	181000 (165000–200000)	10·9 (9·70–11·9)	3420 (3100–3760)
Greece	Male	414000 (391000–439000)	24·2 (22·2–26·1)	8220 (7770–8730)
Iceland	Both	8550 (7660–9540)	11·1 (9·76–12·4)	2480 (2220–2770)

Iceland	Female	3340 (2910–3790)	9·00 (7·73–10·3)	1950 (1710–2220)
Iceland	Male	5210 (4690–5780)	13·0 (11·6–14·5)	2990 (2690–3320)
Ireland	Both	145000 (133000–158000)	12·3 (11·0–13·6)	2960 (2720–3230)
Ireland	Female	63300 (57100–70400)	10·7 (9·37–11·9)	2550 (2300–2840)
Ireland	Male	82100 (75600–88400)	14·0 (12·5–15·3)	3380 (3110–3640)
Israel	Both	158000 (144000–173000)	8·66 (7·66–9·58)	1700 (1550–1860)
Israel	Female	51700 (45500–58600)	5·51 (4·77–6·22)	1110 (972–1250)
Israel	Male	107000 (98100–115000)	12·0 (10·8–13·1)	2300 (2120–2490)
Italy	Both	2060000 (1900000–2230000)	11·4 (10·2–12·6)	3420 (3150–3690)
Italy	Female	672000 (598000–751000)	7·30 (6·51–8·12)	2170 (1930–2420)
Italy	Male	1390000 (1290000–1480000)	15·7 (14·2–17·2)	4730 (4410–5060)
Luxembourg	Both	17600 (15500–20000)	11·6 (10·1–13·0)	2850 (2510–3230)
Luxembourg	Female	6520 (5580–7610)	8·55 (7·32–9·75)	2120 (1810–2470)
Luxembourg	Male	11100 (9820–12500)	14·7 (13·0–16·4)	3570 (3160–4030)
Malta	Both	14300 (12800–16100)	11·7 (10·5–12·9)	3270 (2910–3660)
Malta	Female	4200 (3570–4950)	6·94 (6·03–7·94)	1910 (1630–2250)
Malta	Male	10100 (9160–11200)	16·3 (14·9–17·9)	4620 (4180–5130)
Monaco	Both	2190 (1810–2590)	16·0 (14·1–18·1)	5830 (4810–6890)
Monaco	Female	756 (589–941)	11·2 (9·31–13·3)	3920 (3050–4880)
Monaco	Male	1430 (1200–1660)	20·6 (18·5–23·1)	7830 (6580–9090)
Netherlands	Both	715000 (666000–765000)	14·9 (13·4–16·4)	4170 (3880–4460)
Netherlands	Female	303000 (277000–330000)	12·4 (11·0–13·8)	3510 (3210–3820)
Netherlands	Male	412000 (384000–441000)	17·6 (15·9–19·1)	4840 (4520–5180)
Norway	Both	127000 (116000–138000)	9·29 (8·34–10·3)	2370 (2170–2590)
Norway	Female	50700 (44600–57500)	7·40 (6·40–8·38)	1910 (1680–2170)
Norway	Male	75900 (70000–82700)	11·2 (10·0–12·4)	2810 (2590–3060)
Portugal	Both	323000 (296000–351000)	9·71 (8·79–10·6)	3040 (2780–3290)

Portugal	Female	69500 (58700–80500)	4·15 (3·65–4·66)	1240 (1050–1440)
Portugal	Male	254000 (236000–273000)	15·3 (14·0–16·5)	5010 (4660–5390)
San Marino	Both	993 (702–1350)	10·8 (9·02–12·8)	3000 (2120–4090)
San Marino	Female	322 (231–453)	6·97 (5·61–8·63)	1870 (1350–2640)
San Marino	Male	672 (463–910)	14·8 (12·5–17·0)	4220 (2910–5710)
Spain	Both	1640000 (1510000–1760000)	13·0 (11·7–14·3)	3550 (3290–3820)
Spain	Female	407000 (357000–459000)	6·40 (5·70–7·11)	1730 (1520–1950)
Spain	Male	1230000 (1150000–1310000)	19·6 (17·9–21·4)	5450 (5110–5810)
Sweden	Both	304000 (278000–331000)	11·1 (9·97–12·2)	2970 (2720–3240)
Sweden	Female	150000 (135000–165000)	10·9 (9·61–12·1)	2950 (2660–3250)
Sweden	Male	154000 (141000–168000)	11·3 (10·3–12·4)	3000 (2740–3270)
Switzerland	Both	249000 (226000–273000)	11·1 (9·92–12·2)	2830 (2580–3110)
Switzerland	Female	96800 (85600–109000)	8·44 (7·45–9·49)	2200 (1940–2480)
Switzerland	Male	152000 (140000–165000)	13·9 (12·5–15·3)	3470 (3190–3770)
United Kingdom	Both	2720000 (2540000–2930000)	13·8 (12·5–15·2)	4040 (3770–4350)
United Kingdom	Female	1200000 (1100000–1310000)	12·2 (10·9–13·5)	3530 (3230–3860)
United Kingdom	Male	1520000 (1420000–1620000)	15·5 (14·1–16·9)	4570 (4280–4880)
**Latin America and Caribbean**	**Both**	**8560000 (7870000–9330000)**	**5·16 (4·71–5·57)**	**1460 (1350–1600)**
**Latin America and Caribbean**	**Female**	**2860000 (2590000–3150000)**	**3·74 (3·32–4·14)**	**960 (871–1060)**
**Latin America and Caribbean**	**Male**	**5700000 (5240000–6210000)**	**6·38 (5·92–6·82)**	**1990 (1830–2170)**
Andean Latin America	Both	330000 (275000–394000)	2·13 (1·88–2·37)	519 (433–620)
Andean Latin America	Female	70900 (57300–87700)	0·947 (0·800–1·12)	223 (180–276)
Andean Latin America	Male	259000 (214000–307000)	3·23 (2·87–3·55)	815 (672–966)
Bolivia (Plurinational State of)	Both	89100 (70400–110000)	2·44 (2·08–2·81)	741 (586–914)
Bolivia (Plurinational State of)	Female	17700 (13200–23600)	0·975 (0·762–1·21)	294 (220–393)
Bolivia (Plurinational State of)	Male	71400 (56000–88200)	3·87 (3·33–4·43)	1190 (931–1470)
Ecuador	Both	122000 (97400–153000)	2·79 (2·40–3·24)	694 (554–868)

Ecuador	Female	26400 (20800–33500)	1·30 (1·05–1·61)	299 (235–379)
Ecuador	Male	95700 (75800–120000)	4·08 (3·55–4·68)	1090 (865–1380)
Peru	Both	119000 (90900–155000)	1·58 (1·33–1·85)	350 (267–456)
Peru	Female	26800 (19800–36500)	0·734 (0·580–0·914)	158 (117–215)
Peru	Male	92100 (70300–122000)	2·39 (2·02–2·76)	541 (413–714)
Caribbean	Both	1020000 (892000–1180000)	6·16 (5·57–6·75)	2170 (1890–2500)
Caribbean	Female	320000 (276000–372000)	4·07 (3·57–4·56)	1340 (1160–1560)
Caribbean	Male	704000 (613000–807000)	8·03 (7·36–8·72)	3030 (2630–3470)
Antigua and Barbuda	Both	1020 (868–1210)	4·23 (3·71–4·78)	1160 (981–1360)
Antigua and Barbuda	Female	318 (255–403)	2·68 (2·16–3·31)	704 (563–891)
Antigua and Barbuda	Male	705 (595–831)	5·73 (5·09–6·44)	1630 (1380–1920)
Bahamas	Both	4650 (3740–5740)	3·82 (3·31–4·37)	1230 (993–1520)
Bahamas	Female	1000 (758–1300)	1·81 (1·44–2·27)	514 (390–666)
Bahamas	Male	3650 (2940–4480)	5·49 (4·82–6·18)	2000 (1620–2460)
Barbados	Both	3910 (3270–4670)	3·92 (3·49–4·37)	1310 (1100–1570)
Barbados	Female	773 (607–985)	1·53 (1·25–1·84)	501 (393–638)
Barbados	Male	3140 (2610–3760)	6·38 (5·68–7·12)	2180 (1820–2620)
Belize	Both	3970 (3410–4580)	3·71 (3·27–4·16)	969 (833–1120)
Belize	Female	671 (535–836)	1·47 (1·17–1·80)	326 (260–406)
Belize	Male	3300 (2840–3800)	5·39 (4·84–5·94)	1620 (1390–1860)
Bermuda	Both	1460 (1230–1730)	7·94 (7·00–8·98)	2270 (1920–2700)
Bermuda	Female	355 (289–439)	4·30 (3·62–5·12)	1070 (873–1330)
Bermuda	Male	1100 (939–1300)	10·9 (9·78–12·1)	3560 (3040–4210)
Cuba	Both	491000 (409000–583000)	14·1 (12·6–15·7)	4330 (3600–5130)
Cuba	Female	158000 (129000–189000)	9·65 (8·17–11·3)	2760 (2260–3300)
Cuba	Male	334000 (274000–401000)	18·1 (16·4–19·8)	5900 (4850–7100)
Dominica	Both	1060 (877–1290)	4·25 (3·80–4·71)	1550 (1280–1880)

Dominica	Female	208 (163–266)	1·86 (1·53–2·26)	620 (486–791)
Dominica	Male	857 (701–1040)	6·19 (5·58–6·83)	2440 (2000–2970)
Dominican Republic	Both	206000 (162000–262000)	6·23 (5·44–7·04)	1900 (1490–2410)
Dominican Republic	Female	73100 (57900–92800)	5·11 (4·26–6·00)	1350 (1070–1720)
Dominican Republic	Male	133000 (104000–170000)	7·09 (6·30–7·95)	2440 (1900–3110)
Grenada	Both	1570 (1390–1750)	4·84 (4·32–5·42)	1520 (1340–1690)
Grenada	Female	325 (264–399)	2·19 (1·77–2·69)	646 (525–792)
Grenada	Male	1240 (1090–1390)	7·07 (6·33–7·89)	2350 (2070–2640)
Guyana	Both	13500 (10500–17100)	4·35 (3·87–4·90)	1750 (1360–2220)
Guyana	Female	2450 (1830–3270)	1·81 (1·47–2·27)	631 (470–841)
Guyana	Male	11000 (8530–14000)	6·34 (5·72–7·03)	2890 (2240–3670)
Haiti	Both	96300 (73400–128000)	1·63 (1·34–1·97)	777 (592–1030)
Haiti	Female	28700 (21300–39500)	0·976 (0·760–1·24)	450 (334–618)
Haiti	Male	67600 (51000–89500)	2·28 (1·87–2·74)	1120 (848–1490)
Jamaica	Both	45900 (37500–56100)	6·00 (5·29–6·69)	1630 (1330–1990)
Jamaica	Female	10900 (8620–13600)	2·88 (2·40–3·39)	769 (609–963)
Jamaica	Male	35000 (28100–43000)	9·06 (8·06–10·0)	2510 (2020–3080)
Puerto Rico	Both	66900 (53100–82000)	6·16 (5·32–7·10)	1900 (1510–2330)
Puerto Rico	Female	21500 (17000–26400)	4·16 (3·46–4·94)	1160 (921–1430)
Puerto Rico	Male	45400 (35300–56500)	7·99 (7·03–9·11)	2720 (2110–3380)
Saint Kitts and Nevis	Both	746 (609–895)	3·67 (3·07–4·22)	1250 (1020–1500)
Saint Kitts and Nevis	Female	138 (105–180)	1·61 (1·23–2·06)	464 (352–608)
Saint Kitts and Nevis	Male	609 (494–730)	5·17 (4·29–5·94)	2040 (1650–2450)
Saint Lucia	Both	2930 (2500–3440)	5·33 (4·76–5·88)	1680 (1430–1970)
Saint Lucia	Female	559 (449–686)	2·25 (1·84–2·72)	637 (511–782)
Saint Lucia	Male	2370 (2030–2780)	7·88 (7·16–8·59)	2730 (2340–3200)
Saint Vincent and the Grenadines	Both	1720 (1450–2040)	4·45 (3·87–5·04)	1520 (1280–1800)

Saint Vincent and the Grenadines	Female	309 (237–395)	1·80 (1·45–2·25)	558 (429–714)
Saint Vincent and the Grenadines	Male	1410 (1190–1660)	6·56 (5·77–7·35)	2440 (2060–2860)
Suriname	Both	13500 (11200–16200)	6·91 (6·23–7·59)	2340 (1950–2810)
Suriname	Female	3140 (2500–3930)	3·47 (2·81–4·21)	1080 (859–1350)
Suriname	Male	10300 (8570–12300)	9·89 (9·06–10·7)	3630 (3010–4330)
Trinidad and Tobago	Both	29800 (22800–38600)	6·45 (5·64–7·24)	2150 (1650–2780)
Trinidad and Tobago	Female	5970 (4410–8090)	2·87 (2·33–3·52)	864 (638–1170)
Trinidad and Tobago	Male	23800 (18300–31000)	9·37 (8·33–10·4)	3420 (2630–4450)
United States Virgin Islands	Both	2700 (2230–3180)	6·63 (5·69–7·67)	2600 (2140–3060)
United States Virgin Islands	Female	787 (625–969)	4·57 (3·74–5·53)	1440 (1140–1770)
United States Virgin Islands	Male	1920 (1600–2240)	8·13 (7·08–9·25)	3890 (3240–4550)
Central Latin America	Both	2520000 (2160000–2940000)	3·78 (3·39–4·15)	1010 (866–1170)
Central Latin America	Female	706000 (595000–840000)	2·31 (1·99–2·61)	552 (466–657)
Central Latin America	Male	1820000 (1550000–2120000)	5·04 (4·60–5·46)	1490 (1270–1740)
Colombia	Both	465000 (368000–580000)	4·02 (3·52–4·51)	972 (771–1210)
Colombia	Female	173000 (138000–216000)	3·23 (2·72–3·78)	710 (563–883)
Colombia	Male	291000 (229000–369000)	4·69 (4·18–5·20)	1250 (982–1580)
Costa Rica	Both	54000 (43800–66600)	4·96 (4·36–5·54)	1150 (929–1410)
Costa Rica	Female	14400 (11600–17500)	2·83 (2·39–3·28)	592 (475–721)
Costa Rica	Male	39600 (31800–49300)	6·83 (6·11–7·52)	1730 (1390–2160)
El Salvador	Both	46900 (36000–59600)	2·67 (2·32–3·02)	750 (576–953)
El Salvador	Female	14100 (10600–18500)	1·80 (1·41–2·23)	424 (320–557)
El Salvador	Male	32800 (25400–41700)	3·37 (2·97–3·75)	1120 (867–1420)
Guatemala	Both	102000 (77700–132000)	2·00 (1·68–2·36)	574 (437–743)
Guatemala	Female	25000 (18100–33100)	1·09 (0·835–1·37)	274 (198–362)
Guatemala	Male	77000 (58600–99500)	2·75 (2·35–3·21)	891 (678–1150)
Honduras	Both	108000 (92500–128000)	4·23 (3·72–4·79)	1100 (942–1300)

Honduras	Female	27600 (21100–36300)	2·16 (1·70–2·71)	548 (419–720)
Honduras	Male	80300 (68600–94400)	6·31 (5·61–7·13)	1680 (1440–1980)
Mexico	Both	1220000 (1030000–1440000)	3·59 (3·16–4·06)	975 (821–1150)
Mexico	Female	297000 (244000–361000)	1·90 (1·62–2·21)	465 (382–565)
Mexico	Male	921000 (760000–1110000)	5·01 (4·49–5·58)	1510 (1240–1820)
Nicaragua	Both	43900 (35700–52800)	3·02 (2·61–3·42)	675 (548–811)
Nicaragua	Female	9050 (6970–11500)	1·34 (1·06–1·65)	274 (211–350)
Nicaragua	Male	34900 (28400–42000)	4·49 (3·93–5·03)	1090 (884–1310)
Panama	Both	28800 (22600–36100)	2·99 (2·57–3·45)	692 (543–867)
Panama	Female	7100 (5480–9230)	1·60 (1·30–1·95)	344 (265–447)
Panama	Male	21700 (16900–27700)	4·19 (3·69–4·73)	1040 (805–1320)
Venezuela (Bolivarian Republic of)	Both	458000 (352000–586000)	5·50 (4·79–6·22)	1630 (1260–2090)
Venezuela (Bolivarian Republic of)	Female	138000 (106000–181000)	3·76 (3·07–4·54)	966 (742–1270)
Venezuela (Bolivarian Republic of)	Male	320000 (243000–410000)	6·88 (6·10–7·68)	2320 (1760–2980)
Tropical Latin America	Both	4680000 (4370000–5010000)	6·99 (6·30–7·60)	2090 (1960–2240)
Tropical Latin America	Female	1760000 (1610000–1920000)	5·79 (5·06–6·46)	1540 (1410–1680)
Tropical Latin America	Male	2920000 (2720000–3120000)	7·99 (7·37–8·59)	2670 (2490–2850)
Brazil	Both	4570000 (4260000–4890000)	7·00 (6·31–7·61)	2110 (1970–2260)
Brazil	Female	1730000 (1580000–1890000)	5·84 (5·11–6·53)	1560 (1430–1710)
Brazil	Male	2830000 (2640000–3030000)	7·96 (7·34–8·57)	2680 (2500–2870)
Paraguay	Both	111000 (86500–140000)	6·51 (5·61–7·39)	1600 (1250–2020)
Paraguay	Female	28100 (21700–36500)	3·63 (2·93–4·44)	821 (634–1070)
Paraguay	Male	82600 (64800–104000)	8·93 (7·87–10·0)	2350 (1850–2960)
**North Africa and Middle East**	**Both**	**11500000 (10200000–12900000)**	**7·02 (6·39–7·61)**	**1890 (1680–2120)**
**North Africa and Middle East**	**Female**	**1440000 (1290000–1620000)**	**1·90 (1·69–2·12)**	**493 (440–555)**
**North Africa and Middle East**	**Male**	**10000000 (8900000–11300000)**	**11·4 (10·5–12·3)**	**3170 (2820–3570)**
North Africa and Middle East	Both	11500000 (10200000–12900000)	7·02 (6·39–7·61)	1890 (1680–2120)

North Africa and Middle East	Female	1440000 (1290000–1620000)	1·90 (1·69–2·12)	493 (440–555)
North Africa and Middle East	Male	10000000 (8900000–11300000)	11·4 (10·5–12·3)	3170 (2820–3570)
Afghanistan	Both	356000 (275000–450000)	2·08 (1·65–2·49)	931 (719–1180)
Afghanistan	Female	70300 (49300–98100)	0·835 (0·607–1·16)	377 (264–526)
Afghanistan	Male	286000 (218000–360000)	3·29 (2·60–3·93)	1460 (1110–1830)
Algeria	Both	607000 (508000–727000)	6·00 (5·23–6·79)	1450 (1210–1740)
Algeria	Female	52800 (39900–69200)	1·05 (0·826–1·34)	256 (193–335)
Algeria	Male	554000 (464000–664000)	10·9 (9·63–12·2)	2610 (2190–3130)
Bahrain	Both	19900 (16200–24200)	6·96 (5·88–8·11)	1380 (1130–1680)
Bahrain	Female	2460 (1870–3290)	2·19 (1·67–2·91)	452 (344–603)
Bahrain	Male	17400 (14100–21100)	10·1 (8·61–11·6)	1940 (1580–2350)
Egypt	Both	2290000 (1820000–2930000)	8·68 (7·64–9·86)	2310 (1840–2950)
Egypt	Female	78000 (53700–108000)	0·655 (0·477–0·878)	164 (112–226)
Egypt	Male	2210000 (1760000–2830000)	15·3 (13·7–17·2)	4310 (3420–5520)
Iran (Islamic Republic of)	Both	1230000 (1130000–1340000)	6·22 (5·53–6·90)	1460 (1340–1590)
Iran (Islamic Republic of)	Female	177000 (149000–211000)	1·93 (1·57–2·33)	427 (360–508)
Iran (Islamic Republic of)	Male	1050000 (969000–1140000)	9·97 (9·10–10·9)	2460 (2260–2670)
Iraq	Both	750000 (591000–911000)	7·29 (6·21–8·30)	1780 (1400–2160)
Iraq	Female	112000 (85500–144000)	2·38 (1·89–2·94)	546 (416–700)
Iraq	Male	638000 (503000–774000)	11·4 (9·92–12·8)	2960 (2330–3590)
Jordan	Both	161000 (135000–192000)	7·54 (6·37–8·67)	1380 (1160–1650)
Jordan	Female	25600 (21100–30800)	2·55 (2·18–2·96)	473 (388–569)
Jordan	Male	135000 (111000–164000)	11·9 (10·2–13·5)	2180 (1790–2650)
Kuwait	Both	57600 (48200–68600)	7·60 (6·51–8·75)	1300 (1090–1550)
Kuwait	Female	3570 (2730–4610)	1·08 (0·843–1·38)	174 (133–224)
Kuwait	Male	54000 (44900–64600)	12·6 (11·1–14·0)	2280 (1890–2730)
Lebanon	Both	203000 (174000–231000)	15·0 (13·4–16·7)	3920 (3370–4470)

Lebanon	Female	71600 (61700–83500)	10·9 (9·42–12·5)	2720 (2350–3180)
Lebanon	Male	131000 (113000–150000)	19·0 (17·1–20·7)	5150 (4440–5900)
Libya	Both	120000 (97700–148000)	7·01 (6·11–8·02)	1780 (1450–2190)
Libya	Female	3640 (2570–5060)	0·457 (0·338–0·625)	112 (78·9–155)
Libya	Male	116000 (94600–143000)	12·7 (11·3–14·5)	3330 (2720–4120)
Morocco	Both	637000 (493000–768000)	6·30 (5·32–7·16)	1770 (1370–2140)
Morocco	Female	42700 (30900–58000)	0·860 (0·650–1·12)	239 (173–324)
Morocco	Male	594000 (456000–716000)	11·5 (9·95–13·0)	3290 (2520–3960)
Oman	Both	29100 (25200–33800)	3·40 (2·93–3·88)	635 (549–738)
Oman	Female	2570 (1950–3360)	0·828 (0·610–1·08)	158 (120–206)
Oman	Male	26600 (22700–30900)	4·87 (4·25–5·52)	899 (769–1050)
Palestine	Both	66200 (57000–76700)	6·65 (5·65–7·65)	1340 (1150–1550)
Palestine	Female	5730 (4400–7260)	1·21 (0·924–1·54)	236 (181–298)
Palestine	Male	60500 (52000–69900)	11·6 (10·0–13·1)	2400 (2060–2770)
Qatar	Both	20800 (16600–25600)	4·63 (3·97–5·41)	727 (579–895)
Qatar	Female	686 (486–949)	0·570 (0·420–0·779)	94·4 (66·8–131)
Qatar	Male	20100 (16000–24800)	6·13 (5·31–7·08)	942 (750–1160)
Saudi Arabia	Both	492000 (395000–605000)	5·90 (5·08–6·87)	1380 (1100–1690)
Saudi Arabia	Female	34800 (24500–48300)	1·10 (0·794–1·46)	234 (165–324)
Saudi Arabia	Male	457000 (366000–561000)	8·88 (7·73–10·3)	2190 (1760–2690)
Sudan	Both	492000 (379000–630000)	3·88 (3·09–4·84)	1210 (929–1540)
Sudan	Female	53200 (35500–75900)	0·914 (0·620–1·28)	264 (177–377)
Sudan	Male	439000 (339000–564000)	6·40 (5·16–7·85)	2120 (1640–2730)
Syrian Arab Republic	Both	399000 (309000–526000)	10·2 (8·68–11·7)	2760 (2140–3630)
Syrian Arab Republic	Female	57700 (41900–78600)	3·11 (2·37–3·97)	776 (564–1060)
Syrian Arab Republic	Male	342000 (261000–450000)	16·5 (14·4–18·7)	4840 (3700–6370)
Tunisia	Both	296000 (231000–376000)	10·3 (8·84–11·8)	2560 (1990–3250)

Tunisia	Female	21200 (15400–28500)	1·56 (1·19–1·97)	365 (265–491)
Tunisia	Male	275000 (214000–348000)	18·1 (16·1–20·2)	4770 (3710–6050)
Turkey	Both	2520000 (2100000–3020000)	12·9 (11·2–14·4)	3100 (2580–3710)
Turkey	Female	479000 (402000–571000)	5·06 (4·38–5·77)	1190 (999–1420)
Turkey	Male	2040000 (1700000–2460000)	20·2 (17·8–22·2)	4970 (4130–5990)
United Arab Emirates	Both	176000 (136000–228000)	8·21 (6·90–9·53)	1900 (1470–2470)
United Arab Emirates	Female	9260 (6680–12400)	1·87 (1·41–2·42)	366 (264–491)
United Arab Emirates	Male	166000 (128000–217000)	10·1 (8·56–11·7)	2480 (1910–3230)
Yemen	Both	539000 (425000–706000)	4·63 (3·91–5·51)	1710 (1350–2240)
Yemen	Female	135000 (103000–181000)	2·47 (2·00–3·06)	868 (663–1160)
Yemen	Male	404000 (318000–530000)	6·55 (5·56–7·75)	2530 (2000–3330)
**South Asia**	**Both**	**34400000 (30400000–39400000)**	**5·61 (4·93–6·26)**	**1910 (1680–2180)**
**South Asia**	**Female**	**4930000 (4040000–5860000)**	**1·66 (1·38–1·95)**	**558 (457–663)**
**South Asia**	**Male**	**29500000 (25500000–34200000)**	**9·32 (8·34–10·2)**	**3200 (2760–3710)**
South Asia	Both	34400000 (30400000–39400000)	5·61 (4·93–6·26)	1910 (1680–2180)
South Asia	Female	4930000 (4040000–5860000)	1·66 (1·38–1·95)	558 (457–663)
South Asia	Male	29500000 (25500000–34200000)	9·32 (8·34–10·2)	3200 (2760–3710)
Bangladesh	Both	2870000 (2380000–3460000)	6·67 (5·84–7·53)	1800 (1490–2170)
Bangladesh	Female	202000 (156000–259000)	0·966 (0·768–1·21)	251 (194–321)
Bangladesh	Male	2670000 (2200000–3210000)	12·1 (10·7–13·4)	3400 (2800–4090)
Bhutan	Both	8210 (6400–10300)	3·85 (3·14–4·60)	1090 (849–1360)
Bhutan	Female	1920 (1400–2590)	1·89 (1·43–2·43)	531 (388–716)
Bhutan	Male	6300 (4830–7980)	5·61 (4·58–6·77)	1600 (1230–2030)
India	Both	26800000 (22900000–31500000)	5·74 (4·91–6·54)	1930 (1650–2260)
India	Female	3930000 (3070000–4810000)	1·73 (1·39–2·08)	580 (453–710)
India	Male	22900000 (19100000–27600000)	9·52 (8·36–10·6)	3210 (2670–3870)
Nepal	Both	792000 (654000–916000)	8·53 (7·45–9·57)	2600 (2150–3010)

Nepal	Female	290000 (237000–344000)	6·39 (5·31–7·49)	1820 (1490–2160)
Nepal	Male	502000 (408000–584000)	10·6 (9·27–11·9)	3460 (2810–4030)
Pakistan	Both	3940000 (3140000–4910000)	4·19 (3·37–5·07)	1760 (1400–2190)
Pakistan	Female	506000 (392000–663000)	1·13 (0·897–1·42)	463 (359–607)
Pakistan	Male	3440000 (2650000–4420000)	6·96 (5·62–8·38)	2990 (2300–3850)
**Southeast Asia, East Asia, and Oceania**	**Both**	**77600000 (67900000–88300000)**	**12·9 (11·3–14·5)**	**3590 (3140–4090)**
**Southeast Asia, East Asia, and Oceania**	**Female**	**8950000 (7640000–10400000)**	**3·35 (2·88–3·86)**	**839 (716–974)**
**Southeast Asia, East Asia, and Oceania**	**Male**	**68700000 (59300000–79300000)**	**20·6 (18·7–22·4)**	**6280 (5420–7260)**
East Asia	Both	59100000 (50000000–69700000)	14·9 (12·8–16·9)	4020 (3390–4730)
East Asia	Female	6930000 (5710000–8310000)	3·95 (3·34–4·60)	959 (791–1150)
East Asia	Male	52200000 (43100000–62600000)	23·6 (21·3–25·6)	6960 (5750–8350)
China	Both	57300000 (48200000–68000000)	15·0 (12·9–17·0)	4030 (3390–4780)
China	Female	6710000 (5510000–8090000)	3·98 (3·37–4·66)	962 (789–1160)
China	Male	50500000 (41600000–61000000)	23·7 (21·4–25·8)	6970 (5740–8420)
Democratic People's Republic of Korea	Both	1200000 (1020000–1380000)	13·9 (12·5–15·2)	4560 (3880–5260)
Democratic People's Republic of Korea	Female	168000 (128000–217000)	4·04 (3·13–5·15)	1270 (968–1650)
Democratic People's Republic of Korea	Male	1030000 (865000–1180000)	23·0 (21·1–24·7)	7870 (6630–9060)
Taiwan (Province of China)	Both	687000 (545000–859000)	10·7 (9·51–12·0)	2910 (2310–3640)
Taiwan (Province of China)	Female	53500 (40800–69900)	1·86 (1·51–2·28)	449 (343–587)
Taiwan (Province of China)	Male	633000 (502000–790000)	17·9 (16·4–19·5)	5410 (4290–6740)
Oceania	Both	366000 (298000–452000)	6·47 (5·79–7·16)	2750 (2240–3400)
Oceania	Female	101000 (80000–126000)	3·97 (3·41–4·59)	1570 (1240–1960)
Oceania	Male	264000 (214000–325000)	8·53 (7·67–9·39)	3860 (3120–4750)
American Samoa	Both	1780 (1510–2070)	10·8 (9·71–11·9)	3200 (2730–3740)
American Samoa	Female	539 (432–662)	7·08 (5·97–8·22)	1950 (1570–2400)
American Samoa	Male	1240 (1050–1430)	13·9 (12·7–15·3)	4430 (3760–5120)
Cook Islands	Both	638 (533–754)	10·9 (9·50–12·3)	3550 (2970–4190)

Cook Islands	Female	165 (128–209)	6·46 (5·19–7·71)	1790 (1390–2270)
Cook Islands	Male	473 (399–552)	14·4 (12·8–16·0)	5390 (4550–6290)
Fiji	Both	30000 (23800–37300)	9·13 (8·15–10·0)	3300 (2610–4100)
Fiji	Female	6260 (4840–7970)	4·12 (3·47–4·76)	1390 (1080–1770)
Fiji	Male	23800 (18700–29600)	13·4 (12·1–14·7)	5150 (4060–6410)
Guam	Both	4960 (4140–5940)	10·3 (9·01–11·7)	2910 (2420–3480)
Guam	Female	1510 (1230–1830)	7·05 (5·93–8·31)	1820 (1480–2210)
Guam	Male	3450 (2890–4110)	12·8 (11·4–14·3)	3920 (3280–4670)
Kiribati	Both	8560 (6820–10500)	15·0 (13·4–16·6)	7210 (5750–8810)
Kiribati	Female	2950 (2330–3660)	11·4 (9·92–13·0)	4880 (3850–6060)
Kiribati	Male	5610 (4490–6850)	18·0 (16·2–19·9)	9640 (7730–11800)
Marshall Islands	Both	1890 (1460–2380)	8·86 (7·60–10·1)	3330 (2570–4190)
Marshall Islands	Female	383 (276–513)	4·03 (3·09–5·04)	1380 (994–1850)
Marshall Islands	Male	1510 (1170–1900)	12·7 (11·0–14·5)	5180 (4010–6520)
Micronesia (Federated States of)	Both	5590 (3830–7140)	13·3 (9·47–15·6)	5470 (3750–6990)
Micronesia (Federated States of)	Female	1720 (1230–2260)	9·46 (7·05–11·5)	3440 (2460–4500)
Micronesia (Federated States of)	Male	3860 (2540–4950)	16·3 (10·9–19·1)	7430 (4880–9510)
Nauru	Both	430 (350–524)	11·1 (9·82–12·5)	4070 (3320–4970)
Nauru	Female	171 (131–223)	10·3 (8·75–12·1)	3280 (2510–4260)
Nauru	Male	258 (200–322)	11·7 (10·2–13·3)	4850 (3750–6040)
Niue	Both	72·7 (59·3–87·3)	10·6 (9·37–12·0)	4350 (3550–5220)
Niue	Female	20·8 (15·9–26·7)	6·61 (5·44–7·92)	2510 (1920–3210)
Niue	Male	51·9 (42·8–61·1)	14·1 (12·6–15·7)	6180 (5090–7270)
Northern Mariana Islands	Both	1890 (1600–2220)	12·7 (11·2–14·2)	4450 (3760–5220)
Northern Mariana Islands	Female	397 (300–527)	6·49 (5·06–8·09)	1930 (1460–2570)
Northern Mariana Islands	Male	1490 (1270–1720)	17·1 (15·4–18·8)	6810 (5780–7830)
Palau	Both	996 (778–1260)	11·8 (10·3–13·5)	5530 (4320–7000)

Palau	Female	212 (155–279)	6·40 (5·03–7·99)	2590 (1890–3410)
Palau	Male	784 (616–988)	15·3 (13·5–17·3)	7980 (6270–10100)
Papua New Guinea	Both	239000 (182000–308000)	5·52 (4·76–6·37)	2420 (1850–3120)
Papua New Guinea	Female	70000 (52200–91300)	3·58 (2·93–4·31)	1470 (1100–1920)
Papua New Guinea	Male	169000 (131000–217000)	7·12 (6·17–8·15)	3310 (2560–4260)
Samoa	Both	7070 (5850–8630)	11·9 (10·5–13·0)	3350 (2770–4080)
Samoa	Female	2120 (1640–2700)	7·60 (6·38–8·89)	2060 (1600–2630)
Samoa	Male	4960 (4140–5970)	15·6 (14·0–17·1)	4560 (3810–5490)
Solomon Islands	Both	34600 (27800–42200)	11·2 (9·99–12·3)	5270 (4230–6440)
Solomon Islands	Female	8720 (6580–11100)	6·27 (5·21–7·26)	2720 (2050–3440)
Solomon Islands	Male	25800 (20800–31500)	15·2 (13·7–16·7)	7730 (6220–9410)
Tokelau	Both	43·8 (34·8–54·7)	10·2 (8·82–11·6)	3110 (2470–3880)
Tokelau	Female	16·1 (11·7–21·5)	6·99 (5·49–8·69)	2340 (1700–3120)
Tokelau	Male	27·7 (22·4–34·3)	13·9 (12·1–15·6)	3840 (3100–4740)
Tonga	Both	2620 (2190–3120)	9·64 (8·63–10·6)	2560 (2140–3050)
Tonga	Female	480 (375–598)	3·87 (3·20–4·60)	934 (728–1160)
Tonga	Male	2140 (1790–2540)	14·5 (13·2–15·8)	4210 (3510–4980)
Tuvalu	Both	518 (408–665)	11·9 (10·5–13·3)	4390 (3460–5640)
Tuvalu	Female	161 (119–210)	7·98 (6·54–9·53)	2840 (2100–3710)
Tuvalu	Male	357 (282–457)	15·2 (13·5–16·9)	5830 (4610–7470)
Vanuatu	Both	7730 (6180–9870)	7·16 (6·39–8·01)	2620 (2100–3350)
Vanuatu	Female	682 (493–918)	1·48 (1·15–1·83)	469 (339–632)
Vanuatu	Male	7040 (5670–8990)	11·4 (10·2–12·6)	4720 (3790–6020)
Southeast Asia	Both	18100000 (16200000–20100000)	9·14 (8·32–9·94)	2690 (2410–2980)
Southeast Asia	Female	1920000 (1710000–2160000)	2·14 (1·87–2·41)	570 (506–641)
Southeast Asia	Male	16200000 (14400000–18000000)	14·9 (13·8–15·9)	4810 (4280–5350)
Cambodia	Both	447000 (367000–523000)	8·32 (7·25–9·28)	2690 (2210–3150)

Cambodia	Female	63100 (49700–77600)	2·47 (2·04–3·00)	747 (588–918)
Cambodia	Male	384000 (315000–448000)	13·6 (12·0–15·0)	4710 (3860–5500)
Indonesia	Both	7430000 (6180000–8910000)	9·59 (8·31–10·9)	2860 (2380–3430)
Indonesia	Female	617000 (490000–761000)	1·71 (1·42–2·01)	480 (381–592)
Indonesia	Male	6810000 (5580000–8310000)	16·5 (14·8–17·9)	5200 (4260–6350)
Lao People's Democratic Republic	Both	169000 (135000–202000)	7·11 (6·23–7·86)	2360 (1890–2830)
Lao People's Democratic Republic	Female	20700 (15700–26600)	1·92 (1·54–2·35)	583 (441–747)
Lao People's Democratic Republic	Male	148000 (119000–178000)	11·5 (10·1–12·6)	4110 (3300–4950)
Malaysia	Both	701000 (576000–851000)	9·06 (7·96–10·1)	2240 (1840–2720)
Malaysia	Female	47100 (36800–59700)	1·40 (1·11–1·71)	311 (244–395)
Malaysia	Male	654000 (534000–795000)	14·9 (13·4–16·4)	4040 (3300–4910)
Maldives	Both	6180 (5290–7100)	6·82 (5·95–7·68)	1240 (1060–1420)
Maldives	Female	658 (521–819)	1·80 (1·45–2·22)	332 (263–413)
Maldives	Male	5530 (4750–6360)	10·2 (9·02–11·4)	1840 (1580–2120)
Mauritius	Both	30500 (25200–36200)	7·22 (6·48–7·92)	2390 (1980–2840)
Mauritius	Female	2780 (2150–3540)	1·44 (1·17–1·76)	429 (333–547)
Mauritius	Male	27700 (23000–33200)	12·0 (11·0–13·0)	4400 (3650–5270)
Myanmar	Both	1550000 (1330000–1830000)	7·99 (7·20–8·77)	2830 (2430–3340)
Myanmar	Female	356000 (299000–426000)	3·96 (3·32–4·63)	1250 (1050–1500)
Myanmar	Male	1190000 (1020000–1420000)	11·5 (10·4–12·6)	4540 (3870–5400)
Philippines	Both	2940000 (2410000–3610000)	8·99 (7·75–10·3)	2620 (2150–3220)
Philippines	Female	467000 (374000–584000)	3·24 (2·72–3·78)	844 (677–1060)
Philippines	Male	2480000 (1960000–3140000)	13·5 (11·9–15·1)	4350 (3440–5520)
Seychelles	Both	3080 (2700–3530)	9·68 (8·68–10·8)	3020 (2640–3450)
Seychelles	Female	293 (230–369)	2·25 (1·81–2·81)	606 (474–763)
Seychelles	Male	2790 (2420–3200)	14·8 (13·5–16·2)	5190 (4500–5950)
Sri Lanka	Both	313000 (245000–396000)	5·46 (4·77–6·13)	1430 (1120–1810)

Sri Lanka	Female	24600 (18100–32400)	0·933 (0·726–1·16)	218 (160–287)
Sri Lanka	Male	288000 (225000–365000)	9·35 (8·39–10·3)	2730 (2130–3460)
Thailand	Both	1700000 (1320000–2150000)	8·26 (7·18–9·33)	2430 (1890–3070)
Thailand	Female	162000 (126000–204000)	1·78 (1·47–2·11)	450 (352–568)
Thailand	Male	1540000 (1190000–1950000)	13·4 (12·0–14·8)	4500 (3480–5710)
Timor-Leste	Both	25100 (19100–30700)	6·21 (4·78–7·49)	1880 (1430–2300)
Timor-Leste	Female	2170 (1500–3050)	1·17 (0·830–1·64)	328 (228–462)
Timor-Leste	Male	23000 (17300–28000)	10·4 (7·98–12·5)	3400 (2570–4140)
Viet Nam	Both	2770000 (2300000–3270000)	10·7 (9·59–11·8)	2880 (2390–3390)
Viet Nam	Female	156000 (121000–196000)	1·41 (1·13–1·77)	321 (249–404)
Viet Nam	Male	2620000 (2170000–3100000)	17·7 (16·2–19·2)	5480 (4550–6490)
**Sub-Saharan Africa**	**Both**	**6980000 (6100000–8030000)**	**1·37 (1·19–1·54)**	**648 (566–744)**
**Sub-Saharan Africa**	**Female**	**1030000 (881000–1210000)**	**0·430 (0·370–0·492)**	**188 (161–221)**
**Sub-Saharan Africa**	**Male**	**5960000 (5190000–6850000)**	**2·20 (1·91–2·48)**	**1120 (977–1290)**
Central Sub-Saharan Africa	Both	924000 (732000–1150000)	1·60 (1·37–1·85)	702 (556–871)
Central Sub-Saharan Africa	Female	93900 (70300–123000)	0·347 (0·276–0·431)	142 (106–186)
Central Sub-Saharan Africa	Male	830000 (663000–1030000)	2·70 (2·31–3·13)	1270 (1010–1580)
Angola	Both	251000 (202000–310000)	2·00 (1·78–2·24)	834 (670–1030)
Angola	Female	31400 (22500–42700)	0·527 (0·402–0·682)	202 (145–275)
Angola	Male	220000 (176000–271000)	3·32 (2·98–3·71)	1500 (1200–1850)
Central African Republic	Both	71200 (53400–94400)	1·61 (1·36–1·90)	1340 (1010–1780)
Central African Republic	Female	5480 (3590–8080)	0·274 (0·199–0·368)	203 (133–299)
Central African Republic	Male	65700 (49200–86700)	2·72 (2·29–3·22)	2530 (1890–3340)
Congo	Both	46200 (35700–58400)	2·23 (1·91–2·55)	877 (678–1110)
Congo	Female	4150 (2940–5820)	0·407 (0·307–0·531)	156 (111–219)
Congo	Male	42000 (32300–53500)	4·01 (3·44–4·56)	1610 (1240–2050)
Democratic Republic of the Congo	Both	532000 (406000–690000)	1·42 (1·14–1·73)	607 (463–787)

Democratic Republic of the Congo	Female	50900 (36400–70800)	0·291 (0·214–0·381)	116 (83·1–162)
Democratic Republic of the Congo	Male	481000 (366000–623000)	2·41 (1·92–2·95)	1100 (834–1420)
Equatorial Guinea	Both	6160 (4420–8530)	1·22 (0·938–1·53)	434 (312–601)
Equatorial Guinea	Female	566 (353–884)	0·231 (0·153–0·335)	86·7 (54·0–135)
Equatorial Guinea	Male	5590 (4030–7690)	2·17 (1·70–2·66)	729 (526–1000)
Gabon	Both	16800 (13300–20800)	2·70 (2·24–3·22)	962 (759–1190)
Gabon	Female	1320 (924–1850)	0·460 (0·328–0·623)	146 (102–204)
Gabon	Male	15500 (12300–19200)	4·62 (3·86–5·45)	1840 (1450–2270)
Eastern Sub-Saharan Africa	Both	2610000 (2250000–3020000)	1·53 (1·33–1·74)	635 (546–734)
Eastern Sub-Saharan Africa	Female	390000 (318000–467000)	0·492 (0·410–0·580)	188 (153–225)
Eastern Sub-Saharan Africa	Male	2220000 (1900000–2580000)	2·42 (2·10–2·76)	1090 (930–1260)
Burundi	Both	91900 (68600–121000)	1·61 (1·33–1·92)	770 (575–1010)
Burundi	Female	12000 (8180–17200)	0·465 (0·345–0·610)	200 (136–286)
Burundi	Male	79900 (59600–104000)	2·56 (2·12–3·04)	1350 (1010–1760)
Comoros	Both	7010 (5390–8860)	2·70 (2·16–3·29)	981 (755–1240)
Comoros	Female	866 (597–1210)	0·682 (0·483–0·928)	242 (167–339)
Comoros	Male	6140 (4700–7800)	4·65 (3·73–5·62)	1720 (1320–2190)
Djibouti	Both	16300 (11800–22000)	3·48 (2·91–4·13)	1350 (980–1830)
Djibouti	Female	1290 (876–1890)	0·619 (0·452–0·823)	230 (156–336)
Djibouti	Male	15000 (10900–20200)	5·80 (4·87–6·85)	2350 (1710–3170)
Eritrea	Both	56700 (40000–75800)	2·10 (1·66–2·56)	845 (596–1130)
Eritrea	Female	1440 (927–2130)	0·118 (0·0818–0·168)	43·1 (27·8–63·9)
Eritrea	Male	55200 (39000–73900)	3·72 (2·93–4·51)	1640 (1150–2190)
Ethiopia	Both	240000 (197000–290000)	0·631 (0·512–0·773)	223 (183–270)
Ethiopia	Female	16100 (11800–22100)	0·0933 (0·0678–0·126)	30·2 (22·2–41·4)
Ethiopia	Male	223000 (182000–274000)	1·08 (0·870–1·32)	411 (334–504)
Kenya	Both	329000 (273000–398000)	1·86 (1·54–2·21)	655 (543–792)

Kenya	Female	34100 (26300–44600)	0·413 (0·326–0·520)	136 (104–177)
Kenya	Male	295000 (239000–361000)	3·13 (2·61–3·68)	1180 (955–1440)
Madagascar	Both	194000 (148000–251000)	1·81 (1·48–2·19)	728 (554–941)
Madagascar	Female	19600 (13500–27400)	0·380 (0·280–0·508)	147 (101–205)
Madagascar	Male	175000 (132000–226000)	3·12 (2·57–3·76)	1310 (988–1690)
Malawi	Both	144000 (113000–181000)	1·92 (1·53–2·30)	779 (613–979)
Malawi	Female	15900 (11300–21800)	0·459 (0·336–0·616)	168 (119–230)
Malawi	Male	128000 (99900–161000)	3·18 (2·52–3·82)	1420 (1110–1790)
Mozambique	Both	265000 (203000–342000)	1·55 (1·23–1·90)	899 (689–1160)
Mozambique	Female	32900 (22700–46900)	0·413 (0·294–0·558)	215 (148–306)
Mozambique	Male	233000 (179000–298000)	2·53 (2·02–3·12)	1640 (1260–2100)
Rwanda	Both	135000 (109000–165000)	3·16 (2·58–3·75)	1070 (856–1300)
Rwanda	Female	45200 (35700–56500)	2·17 (1·73–2·68)	694 (547–866)
Rwanda	Male	90200 (72000–111000)	4·09 (3·33–4·93)	1460 (1170–1800)
Somalia	Both	183000 (132000–247000)	1·40 (1·12–1·72)	900 (650–1210)
Somalia	Female	19000 (12100–28700)	0·319 (0·218–0·455)	190 (122–288)
Somalia	Male	164000 (118000–220000)	2·31 (1·85–2·82)	1580 (1140–2120)
South Sudan	Both	59400 (43300–80100)	1·13 (0·871–1·44)	640 (467–863)
South Sudan	Female	5230 (3410–7530)	0·217 (0·147–0·299)	113 (73·7–163)
South Sudan	Male	54200 (39500–73100)	1·92 (1·47–2·45)	1160 (847–1570)
Uganda	Both	194000 (154000–240000)	1·14 (0·923–1·38)	472 (374–585)
Uganda	Female	33700 (25100–44400)	0·437 (0·332–0·564)	161 (120–212)
Uganda	Male	160000 (126000–200000)	1·72 (1·39–2·11)	793 (625–990)
United Republic of Tanzania	Both	555000 (453000–678000)	2·39 (1·99–2·80)	978 (799–1200)
United Republic of Tanzania	Female	129000 (100000–162000)	1·15 (0·914–1·40)	442 (344–555)
United Republic of Tanzania	Male	426000 (346000–520000)	3·53 (2·91–4·18)	1540 (1260–1890)
Zambia	Both	140000 (107000–176000)	1·76 (1·40–2·14)	770 (589–967)

Zambia	Female	23100 (17300–30500)	0·641 (0·472–0·825)	251 (188–331)
Zambia	Male	117000 (88100–148000)	2·68 (2·14–3·26)	1300 (978–1640)
Southern Sub-Saharan Africa	Both	1440000 (1300000–1580000)	3·78 (3·42–4·16)	1830 (1650–2010)
Southern Sub-Saharan Africa	Female	299000 (262000–336000)	1·67 (1·48–1·89)	743 (651–834)
Southern Sub-Saharan Africa	Male	1140000 (1030000–1260000)	5·63 (5·08–6·20)	2970 (2680–3280)
Botswana	Both	49300 (36900–63400)	4·38 (3·63–5·10)	2110 (1580–2710)
Botswana	Female	9380 (6610–13000)	1·80 (1·37–2·29)	789 (557–1090)
Botswana	Male	39900 (30000–50600)	6·59 (5·50–7·67)	3470 (2610–4400)
Eswatini	Both	10800 (8100–14200)	1·69 (1·40–2·01)	945 (709–1240)
Eswatini	Female	1770 (1130–2580)	0·638 (0·444–0·856)	303 (194–442)
Eswatini	Male	9020 (6880–11700)	2·50 (2·06–2·96)	1620 (1230–2100)
Lesotho	Both	72700 (56200–90400)	4·41 (3·71–5·12)	3480 (2690–4320)
Lesotho	Female	5650 (3660–8380)	0·759 (0·537–1·06)	533 (346–791)
Lesotho	Male	67100 (52200–83500)	7·40 (6·24–8·64)	6490 (5050–8080)
Namibia	Both	29400 (23500–36600)	3·01 (2·65–3·38)	1220 (977–1520)
Namibia	Female	9230 (6910–12300)	2·08 (1·71–2·51)	745 (557–990)
Namibia	Male	20200 (16100–24900)	3·77 (3·31–4·28)	1740 (1390–2140)
South Africa	Both	1010000 (916000–1100000)	3·80 (3·41–4·20)	1810 (1650–1980)
South Africa	Female	236000 (205000–268000)	1·89 (1·65–2·15)	831 (722–945)
South Africa	Male	773000 (693000–853000)	5·49 (4·85–6·09)	2840 (2550–3130)
Zimbabwe	Both	269000 (213000–327000)	3·74 (3·10–4·39)	1790 (1420–2180)
Zimbabwe	Female	37500 (27200–50400)	1·10 (0·832–1·40)	480 (348–646)
Zimbabwe	Male	231000 (185000–281000)	6·11 (5·08–7·11)	3210 (2560–3890)
Western Sub-Saharan Africa	Both	2010000 (1690000–2380000)	0·826 (0·697–0·956)	440 (371–521)
Western Sub-Saharan Africa	Female	247000 (198000–306000)	0·214 (0·176–0·260)	106 (85·0–131)
Western Sub-Saharan Africa	Male	1760000 (1470000–2080000)	1·38 (1·16–1·60)	790 (661–935)
Benin	Both	58000 (44600–74700)	0·888 (0·742–1·03)	458 (352–590)

Benin	Female	8070<br>(5500–11000)	0·266<br>(0·197–0·342)	125<br>(85·4–171)
Benin	Male	49900<br>(38600–64100)	1·43<br>(1·20–1·64)	802<br>(620–1030)
Burkina Faso	Both	104000<br>(82000–132000)	0·729<br>(0·567–0·910)	460<br>(361–581)
Burkina Faso	Female	6860<br>(4840–9660)	0·101<br>(0·0704–0·139)	58·9<br>(41·5–82·9)
Burkina Faso	Male	97600<br>(76500–123000)	1·29<br>(1·00–1·63)	885<br>(693–1110)
Cabo Verde	Both	4430<br>(3830–5090)	2·99<br>(2·64–3·34)	786<br>(679–904)
Cabo Verde	Female	677<br>(545–836)	1·05<br>(0·848–1·31)	242<br>(195–299)
Cabo Verde	Male	3750<br>(3240–4310)	4·47<br>(3·97–4·97)	1320<br>(1140–1520)
Cameroon	Both	178000<br>(137000–231000)	1·31<br>(1·10–1·56)	612<br>(471–795)
Cameroon	Female	10600<br>(7320–15200)	0·166<br>(0·120–0·222)	72·8<br>(50·1–104)
Cameroon	Male	167000<br>(129000–217000)	2·34<br>(1·95–2·80)	1160<br>(890–1500)
Chad	Both	92700<br>(70700–119000)	0·802<br>(0·620–1·02)	565<br>(431–725)
Chad	Female	10500<br>(7300–14500)	0·195<br>(0·136–0·271)	127<br>(88·3–176)
Chad	Male	82200<br>(63000–105000)	1·34<br>(1·02–1·71)	1010<br>(774–1280)
Côte d'Ivoire	Both	226000<br>(172000–285000)	1·82<br>(1·44–2·20)	862<br>(659–1090)
Côte d'Ivoire	Female	26200<br>(18600–35300)	0·473<br>(0·346–0·621)	207<br>(147–279)
Côte d'Ivoire	Male	199000<br>(152000–254000)	2·90<br>(2·30–3·51)	1480<br>(1120–1880)
Gambia	Both	19100<br>(14900–23700)	2·40<br>(1·89–2·87)	852<br>(662–1060)
Gambia	Female	881<br>(633–1210)	0·232<br>(0·169–0·316)	77·2<br>(55·5–106)
Gambia	Male	18300<br>(14100–22600)	4·39<br>(3·44–5·25)	1650<br>(1280–2040)
Ghana	Both	165000<br>(133000–202000)	1·34<br>(1·11–1·61)	522<br>(423–641)
Ghana	Female	24700<br>(18600–32800)	0·424<br>(0·316–0·549)	152<br>(115–202)
Ghana	Male	140000<br>(112000–172000)	2·17<br>(1·79–2·60)	914<br>(734–1130)
Guinea	Both	128000<br>(102000–159000)	1·72<br>(1·51–1·94)	1010<br>(805–1260)
Guinea	Female	10800<br>(7520–15200)	0·304<br>(0·222–0·404)	166<br>(115–233)
Guinea	Male	117000<br>(92300–146000)	3·01<br>(2·65–3·39)	1920<br>(1510–2390)
Guinea-Bissau	Both	10100<br>(7990–12600)	1·06<br>(0·911–1·22)	533<br>(420–663)

Guinea-Bissau	Female	1020 (710–1410)	0·231 (0·170–0·303)	104 (72·7–144)
Guinea-Bissau	Male	9120 (7180–11300)	1·78 (1·52–2·04)	987 (777–1220)
Liberia	Both	22300 (16900–28800)	1·13 (0·867–1·42)	466 (353–602)
Liberia	Female	3190 (2240–4400)	0·335 (0·241–0·448)	134 (94·1–185)
Liberia	Male	19100 (14400–24800)	1·87 (1·43–2·37)	793 (598–1030)
Mali	Both	109000 (86500–138000)	0·731 (0·637–0·833)	495 (394–630)
Mali	Female	12900 (9130–17300)	0·185 (0·139–0·240)	117 (82·8–157)
Mali	Male	95700 (75400–122000)	1·22 (1·05–1·40)	879 (693–1120)
Mauritania	Both	23700 (17700–30200)	1·91 (1·51–2·23)	589 (440–751)
Mauritania	Female	5110 (3560–6960)	0·832 (0·624–1·06)	250 (174–341)
Mauritania	Male	18500 (13900–23700)	2·97 (2·34–3·50)	940 (706–1200)
Niger	Both	67100 (50700–86000)	0·432 (0·356–0·503)	288 (218–369)
Niger	Female	6900 (4820–9770)	0·0932 (0·0671–0·125)	58·8 (41·1–83·3)
Niger	Male	60200 (45400–77600)	0·742 (0·608–0·863)	520 (392–671)
Nigeria	Both	556000 (422000–724000)	0·480 (0·360–0·634)	259 (197–337)
Nigeria	Female	86500 (59600–126000)	0·156 (0·107–0·218)	77·5 (53·5–113)
Nigeria	Male	469000 (348000–638000)	0·777 (0·570–1·04)	455 (337–618)
São Tomé and Príncipe	Both	1270 (1030–1520)	2·37 (2·07–2·65)	619 (500–741)
São Tomé and Príncipe	Female	178 (132–235)	0·693 (0·531–0·892)	173 (129–229)
São Tomé and Príncipe	Male	1090 (880–1310)	3·89 (3·40–4·39)	1060 (854–1270)
Senegal	Both	99700 (77700–124000)	1·82 (1·45–2·18)	659 (513–821)
Senegal	Female	7130 (4810–9990)	0·274 (0·186–0·378)	94·5 (63·7–132)
Senegal	Male	92600 (72100–116000)	3·23 (2·56–3·88)	1220 (951–1520)
Sierra Leone	Both	72600 (55800–93600)	1·53 (1·27–1·79)	876 (674–1130)
Sierra Leone	Female	13400 (9780–18400)	0·594 (0·462–0·747)	320 (234–440)
Sierra Leone	Male	59200 (45500–76000)	2·38 (1·96–2·78)	1440 (1110–1850)
Togo	Both	72100 (56300–91400)	2·10 (1·72–2·51)	910 (711–1150)

Togo	Female	11400<br>(8280–15400)	0·712<br>(0·518–0·939)	283<br>(205–380)
Togo	Male	60600<br>(46700–77900)	3·33<br>(2·70–3·97)	1560<br>(1210–2010)

Supplemental Table S7. Ratio of smoking attributable years of life lost (YLLs) to years lived with disability (YLDs), by location for 2019.

Location	YLL to YLD Ratio
**Global**	**5·42 (4·17–7·15)**
**Central Europe, Eastern Europe, and Central Asia**	**6·75 (5·04–9·22)**
Central Asia	8·74 (6·48–12·1)
Armenia	7·51 (5·34–10·4)
Azerbaijan	8·99 (6·25–12·8)
Georgia	8·19 (5·91–11·2)
Kazakhstan	7·36 (5·37–10·2)
Kyrgyzstan	7·25 (5·28–10·2)
Mongolia	11·8 (7·98–17·3)
Tajikistan	9·13 (6·19–13·2)
Turkmenistan	9·20 (6·26–13·3)
Uzbekistan	10·5 (7·48–14·9)
Central Europe	4·77 (3·49–6·51)
Albania	4·65 (3·03–6·92)
Bosnia and Herzegovina	4·63 (3·18–6·72)
Bulgaria	6·16 (4·27–8·88)
Croatia	3·98 (2·75–5·74)
Czechia	3·56 (2·53–5·01)
Hungary	5·05 (3·60–7·06)
Montenegro	5·77 (4·07–8·20)
North Macedonia	5·55 (3·79–8·10)
Poland	4·49 (3·23–6·26)
Romania	5·55 (4·04–7·85)

Serbia	5·16 (3·55–7·34)
Slovakia	4·55 (3·09–6·52)
Slovenia	3·22 (2·21–4·64)
Eastern Europe	8·01 (5·86–11·1)
Belarus	7·90 (5·50–11·3)
Estonia	4·64 (3·23–6·85)
Latvia	5·97 (4·27–8·44)
Lithuania	6·15 (4·38–8·68)
Republic of Moldova	7·43 (5·39–10·3)
Russian Federation	7·60 (5·50–10·7)
Ukraine	9·73 (7·00–13·8)
**High-income**	**3·14 (2·44–4·13)**
Australasia	2·68 (2·05–3·55)
Australia	2·58 (1·96–3·43)
New Zealand	3·24 (2·48–4·27)
High-income Asia Pacific	3·11 (2·34–4·18)
Brunei Darussalam	4·39 (3·16–6·07)
Japan	3·12 (2·35–4·18)
Republic of Korea	3·07 (2·28–4·14)
Singapore	2·99 (2·23–4·04)
High-income North America	2·98 (2·37–3·87)
Canada	3·48 (2·67–4·65)
Greenland	5·48 (3·91–7·60)
United States of America	2·94 (2·34–3·81)
Southern Latin America	4·71 (3·54–6·32)
Argentina	5·32 (4·02–7·16)
Chile	3·00 (2·22–4·10)

Uruguay	5·58 (4·19–7·46)
Western Europe	3·21 (2·45–4·26)
Andorra	3·15 (2·17–4·56)
Austria	3·15 (2·38–4·27)
Belgium	3·39 (2·59–4·47)
Cyprus	2·89 (2·14–3·92)
Denmark	3·50 (2·68–4·62)
Finland	3·20 (2·40–4·29)
France	3·44 (2·54–4·65)
Germany	3·15 (2·41–4·21)
Greece	3·85 (2·97–5·05)
Iceland	2·78 (2·07–3·82)
Ireland	2·88 (2·18–3·87)
Israel	2·77 (2·07–3·80)
Italy	3·04 (2·30–4·03)
Luxembourg	2·57 (1·91–3·52)
Malta	3·03 (2·23–4·22)
Monaco	4·84 (3·46–6·58)
Netherlands	3·37 (2·66–4·36)
Norway	2·66 (2·04–3·52)
Portugal	3·18 (2·36–4·32)
San Marino	3·13 (1·89–5·02)
Spain	3·30 (2·50–4·38)
Sweden	2·73 (2·09–3·61)
Switzerland	2·43 (1·83–3·26)
United Kingdom	3·25 (2·50–4·32)
**Latin America and Caribbean**	**5·14 (3·83–7·04)**

Andean Latin America	4·97 (3·44–7·27)
Bolivia (Plurinational State of)	7·19 (4·74–10·6)
Ecuador	5·18 (3·51–7·67)
Peru	3·82 (2·46–5·73)
Caribbean	6·17 (4·49–8·47)
Antigua and Barbuda	4·63 (3·25–6·60)
Bahamas	6·48 (4·44–9·35)
Barbados	5·11 (3·51–7·36)
Belize	5·80 (4·14–8·17)
Bermuda	5·03 (3·59–7·20)
Cuba	5·73 (4·06–8·10)
Dominica	5·63 (3·89–8·06)
Dominican Republic	8·54 (5·83–12·5)
Grenada	6·38 (4·65–8·81)
Guyana	8·74 (5·82–12·7)
Haiti	9·13 (5·75–14·0)
Jamaica	5·27 (3·60–7·60)
Puerto Rico	3·56 (2·42–5·18)
Saint Kitts and Nevis	6·32 (4·34–8·95)
Saint Lucia	5·03 (3·58–7·04)
Saint Vincent and the Grenadines	5·92 (4·23–8·35)
Suriname	5·99 (4·17–8·54)
Trinidad and Tobago	5·31 (3·41–7·88)
United States Virgin Islands	8·14 (5·90–11·4)
Central Latin America	4·62 (3·35–6·57)
Colombia	3·67 (2·45–5·34)
Costa Rica	3·72 (2·51–5·39)

El Salvador	4·78 (3·18–6·96)
Guatemala	5·02 (3·37–7·39)
Honduras	6·95 (4·96–9·77)
Mexico	4·48 (3·23–6·36)
Nicaragua	4·56 (3·13–6·54)
Panama	4·03 (2·71–5·87)
Venezuela (Bolivarian Republic of)	6·23 (4·16–9·18)
Tropical Latin America	5·27 (3·95–7·12)
Brazil	5·26 (3·96–7·13)
Paraguay	5·40 (3·58–7·92)
**North Africa and Middle East**	**4·98 (3·63–6·89)**
North Africa and Middle East	4·98 (3·63–6·89)
Afghanistan	8·92 (5·88–13·2)
Algeria	4·10 (2·93–5·90)
Bahrain	2·57 (1·77–3·74)
Egypt	6·72 (4·50–10·0)
Iran (Islamic Republic of)	3·75 (2·80–5·09)
Iraq	6·41 (4·32–9·44)
Jordan	3·13 (2·21–4·49)
Kuwait	2·77 (1·94–4·02)
Lebanon	4·68 (3·46–6·33)
Libya	4·27 (2·97–6·12)
Morocco	6·26 (4·16–8·98)
Oman	3·35 (2·31–4·89)
Palestine	5·22 (3·74–7·30)
Qatar	1·59 (1·03–2·44)
Saudi Arabia	4·43 (2·97–6·53)

Sudan	6·58 (4·24–9·98)
Syrian Arab Republic	6·55 (4·37–9·58)
Tunisia	4·57 (3·01–6·83)
Turkey	3·94 (2·78–5·60)
United Arab Emirates	3·43 (2·24–5·21)
Yemen	7·51 (5·07–11·0)
**South Asia**	**7·00 (5·43–9·08)**
South Asia	7·00 (5·43–9·08)
Bangladesh	5·76 (4·19–7·94)
Bhutan	5·54 (4·04–7·69)
India	7·01 (5·36–9·27)
Nepal	6·60 (4·94–8·87)
Pakistan	8·35 (6·01–11·8)
**Southeast Asia, East Asia, and Oceania**	**6·47 (4·85–8·69)**
East Asia	6·71 (4·88–9·14)
China	6·71 (4·85–9·16)
Democratic People's Republic of Korea	8·00 (5·94–10·7)
Taiwan (Province of China)	5·09 (3·47–7·31)
Oceania	7·58 (5·29–10·8)
American Samoa	4·85 (3·45–6·75)
Cook Islands	3·93 (2·86–5·38)
Fiji	7·43 (5·00–10·8)
Guam	5·52 (3·90–7·74)
Kiribati	11·1 (7·66–16·1)
Marshall Islands	8·89 (6·00–12·9)
Micronesia (Federated States of)	10·0 (6·47–15·1)
Nauru	9·54 (6·95–13·5)

Niue	5·84 (4·22–8·09)
Northern Mariana Islands	6·66 (4·83–9·23)
Palau	8·05 (5·66–11·7)
Papua New Guinea	6·96 (4·69–10·1)
Samoa	6·87 (4·82–9·85)
Solomon Islands	16·1 (11·2–22·7)
Tokelau	5·29 (3·70–7·59)
Tonga	6·19 (4·32–8·82)
Tuvalu	7·68 (5·27–11·0)
Vanuatu	10·6 (7·16–15·5)
Southeast Asia	5·79 (4·36–7·72)
Cambodia	6·78 (4·91–9·29)
Indonesia	5·94 (4·31–8·37)
Lao People's Democratic Republic	7·12 (5·12–9·92)
Malaysia	5·78 (4·03–8·15)
Maldives	2·86 (2·11–3·90)
Mauritius	4·13 (2·90–5·77)
Myanmar	6·45 (4·95–8·70)
Philippines	5·94 (4·17–8·34)
Seychelles	5·36 (3·97–7·32)
Sri Lanka	3·79 (2·58–5·48)
Thailand	4·21 (2·82–6·11)
Timor-Leste	6·55 (4·49–9·23)
Viet Nam	6·35 (4·56–8·73)
**Sub-Saharan Africa**	**6·89 (5·11–9·37)**
Central Sub-Saharan Africa	8·19 (5·79–11·6)
Angola	7·83 (5·53–11·2)

Central African Republic	15·5<br>(10·6–22·7)
Congo	7·24<br>(5·03–10·3)
Democratic Republic of the Congo	8·00<br>(5·47–11·5)
Equatorial Guinea	5·31<br>(3·28–8·05)
Gabon	7·21<br>(5·03–10·1)
Eastern Sub-Saharan Africa	7·16<br>(5·25–9·87)
Burundi	8·89<br>(6·04–12·7)
Comoros	6·00<br>(4·12–8·54)
Djibouti	6·62<br>(4·31–9·76)
Eritrea	9·67<br>(6·26–14·6)
Ethiopia	6·21<br>(4·31–8·82)
Kenya	6·05<br>(4·24–8·45)
Madagascar	7·10<br>(4·67–10·4)
Malawi	8·03<br>(5·65–11·4)
Mozambique	9·62<br>(6·55–13·9)
Rwanda	6·41<br>(4·70–8·91)
Somalia	11·5<br>(7·56–16·9)
South Sudan	6·18<br>(4·00–9·17)
Uganda	7·12<br>(5·03–10·1)
United Republic of Tanzania	6·19<br>(4·43–8·81)
Zambia	8·35<br>(5·77–12·1)
Southern Sub-Saharan Africa	6·95<br>(5·39–9·10)
Botswana	8·70<br>(5·86–12·7)
Eswatini	10·3<br>(7·26–14·6)
Lesotho	15·6<br>(11·0–21·9)
Namibia	6·97<br>(4·97–9·74)
South Africa	5·99<br>(4·69–7·83)

Zimbabwe	10·8 (7·67–14·7)
Western Sub-Saharan Africa	6·08 (4·43–8·47)
Benin	6·27 (4·26–9·05)
Burkina Faso	7·10 (4·93–10·2)
Cabo Verde	6·45 (4·70–8·90)
Cameroon	6·75 (4·57–9·97)
Chad	7·37 (5·08–10·5)
Côte d'Ivoire	6·47 (4·35–9·42)
Gambia	7·16 (4·96–10·3)
Ghana	7·80 (5·61–11·0)
Guinea	7·74 (5·38–11·0)
Guinea-Bissau	10·4 (7·17–14·6)
Liberia	5·56 (3·73–8·06)
Mali	5·50 (3·85–7·81)
Mauritania	4·06 (2·63–5·97)
Niger	6·14 (3·99–8·85)
Nigeria	4·87 (3·22–7·11)
São Tomé and Príncipe	5·92 (4·41–8·01)
Senegal	5·96 (4·09–8·50)
Sierra Leone	6·53 (4·38–9·56)
Togo	7·64 (5·25–10·9)

Supplemental Figure S2. Ratio of smoking attributable years of life lost (YLLs) to years lived with disability (YLDs), by location and Socio-demographic Index level, in 1990 and 2019. Size of the point corresponds to the smoking-attributable disability-adjusted life-year rate. Colors correspond to the geographic region of the location.

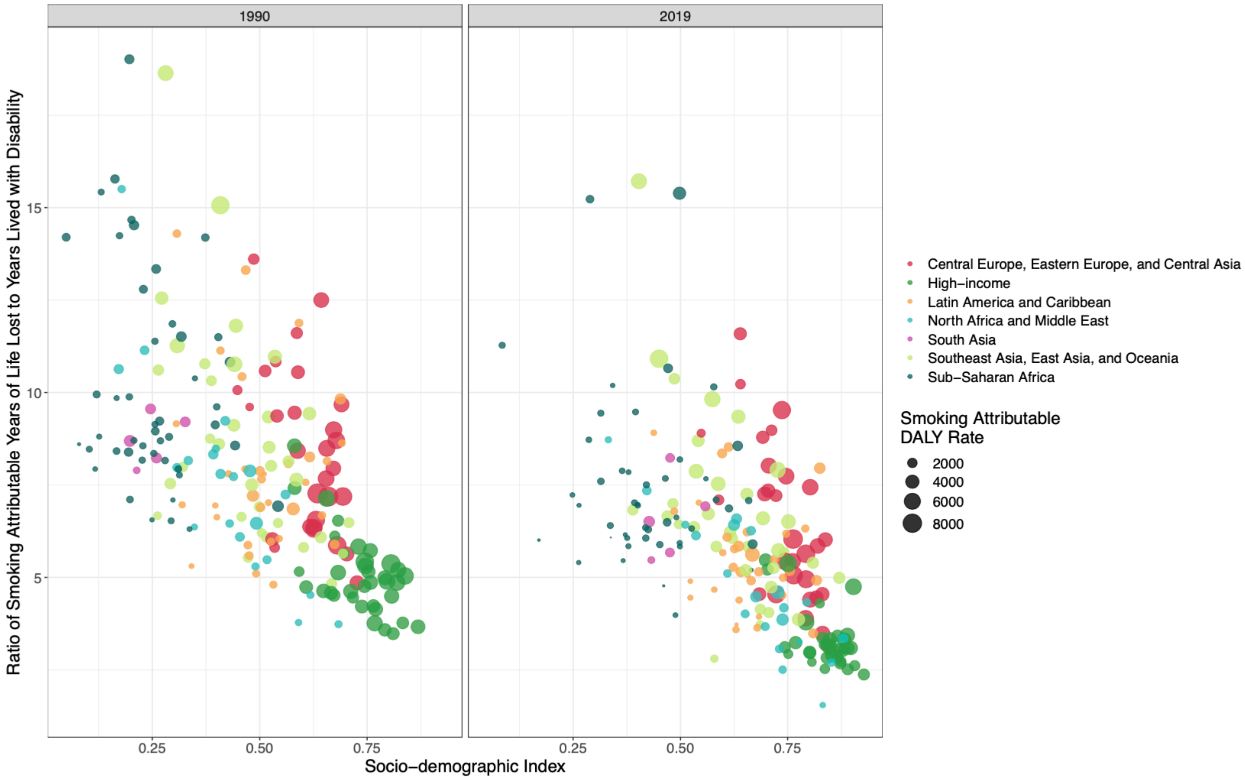

Supplemental Table S8. Number of smoking attributable deaths for all causes and the top four causes of death (chronic obstructive pulmonary disease, ischaemic heart disease, stroke, and lung cancer), by region, in 2019.

Location	All causes	Chronic obstructive pulmonary disease	Ischaemic heart disease	Stroke	Tracheal, bronchus, and lung cancer
**Global**	**7690000 (7160000–8200000)**	**1590000 (1410000–1760000)**	**1680000 (1560000–1810000)**	**931000 (833000–1030000)**	**1310000 (1200000–1430000)**
**Central Europe, Eastern Europe, and Central Asia**	**775000 (712000–841000)**	**57500 (51400–64500)**	**295000 (269000–325000)**	**106000 (95000–117000)**	**126000 (114000–138000)**
Central Asia	84400 (77000–92500)	7610 (6680–8660)	37100 (33300–41200)	13400 (11900–15000)	8870 (7910–9910)
Central Europe	248000 (218000–282000)	22300 (19200–25700)	65400 (56500–74500)	29600 (25500–34400)	60500 (52800–69000)
Eastern Europe	442000 (391000–495000)	27700 (23700–32300)	193000 (170000–217000)	62800 (54900–71900)	56600 (49300–64300)
**High-income**	**1590000 (1530000–1660000)**	**268000 (231000–303000)**	**254000 (234000–272000)**	**84900 (76700–93600)**	**440000 (410000–461000)**
Australasia	23800 (22500–25300)	4590 (3810–5410)	3710 (3340–4000)	1060 (919–1200)	6850 (6220–7370)
High-income Asia Pacific	254000 (238000–274000)	24700 (18900–31000)	23400 (21100–25400)	17100 (15300–19100)	72500 (64100–78000)
High-income North America	578000 (554000–603000)	118000 (97900–133000)	115000 (106000–124000)	26200 (23500–29100)	164000 (153000–172000)
Southern Latin America	67400 (63700–71300)	10800 (9340–12300)	11800 (10900–12700)	5700 (5120–6290)	12700 (11900–13500)
Western Europe	667000 (640000–698000)	110000 (95800–124000)	101000 (92000–108000)	34800 (31200–38800)	184000 (173000–193000)
**Latin America and Caribbean**	**324000 (300000–352000)**	**63700 (55500–72400)**	**73900 (66900–81400)**	**30200 (26800–33800)**	**42500 (38600–46500)**
Andean Latin America	12800 (10600–15300)	2030 (1570–2560)	2470 (1980–2990)	1000 (781–1280)	1630 (1270–2020)
Caribbean	40000 (34500–46600)	4860 (4000–5710)	10700 (9050–12500)	4320 (3610–5110)	6920 (5850–8060)
Central Latin America	97100 (83900–113000)	22800 (18800–27300)	24500 (20700–28900)	6820 (5590–8170)	11400 (9520–13400)
Tropical Latin America	175000 (164000–185000)	34000 (29800–38500)	36300 (33300–39300)	18100 (16100–20200)	22600 (20900–24200)
**North Africa and Middle East**	**374000 (336000–417000)**	**42200 (34900–48800)**	**159000 (138000–186000)**	**37900 (32800–43500)**	**47800 (41900–54500)**
North Africa and Middle East	374000 (336000–417000)	42200 (34900–48800)	159000 (138000–186000)	37900 (32800–43500)	47800 (41900–54500)
**South Asia**	**1290000 (1120000–1480000)**	**457000 (358000–544000)**	**304000 (260000–352000)**	**114000 (97600–132000)**	**58400 (48500–68500)**
South Asia	1290000 (1120000–1480000)	457000 (358000–544000)	304000 (260000–352000)	114000 (97600–132000)	58400 (48500–68500)
**Southeast Asia, East Asia, and Oceania**	**3120000 (2740000–3550000)**	**672000 (573000–781000)**	**558000 (485000–641000)**	**530000 (452000–616000)**	**581000 (483000–686000)**
East Asia	2490000 (2100000–2930000)	565000 (470000–676000)	422000 (354000–502000)	410000 (338000–492000)	504000 (411000–605000)
Oceania	11000 (8920–13700)	2560 (1920–3370)	3390 (2620–4400)	1500 (1100–1970)	766 (567–1100)

Southeast Asia	623000 (560000–689000)	104000 (87600–118000)	132000 (116000–149000)	119000 (104000–135000)	76400 (64900–88400)
**Sub-Saharan Africa**	**220000 (192000–251000)**	**29000 (24100–34100)**	**37400 (31200–44900)**	**27800 (23000–33200)**	**16500 (14300–19100)**
Central Sub-Saharan Africa	27400 (21800–34200)	3560 (2400–4810)	4450 (3340–5830)	3250 (2490–4220)	2270 (1440–4070)
Eastern Sub-Saharan Africa	82600 (71800–95400)	10800 (8670–13200)	12900 (10200–16100)	11800 (9400–14500)	3810 (3080–4830)
Southern Sub-Saharan Africa	47000 (42700–51300)	6670 (5840–7450)	7120 (6310–7940)	4210 (3710–4780)	5630 (5030–6390)
Western Sub-Saharan Africa	62600 (52400–74100)	7930 (6210–9790)	12900 (10400–15600)	8510 (6820–10400)	4830 (3920–5860)

Supplemental Figure S3. Global smoking attributable deaths (number, percent, rate per 100,000) by five-year age group for both sexes combined in 2019.

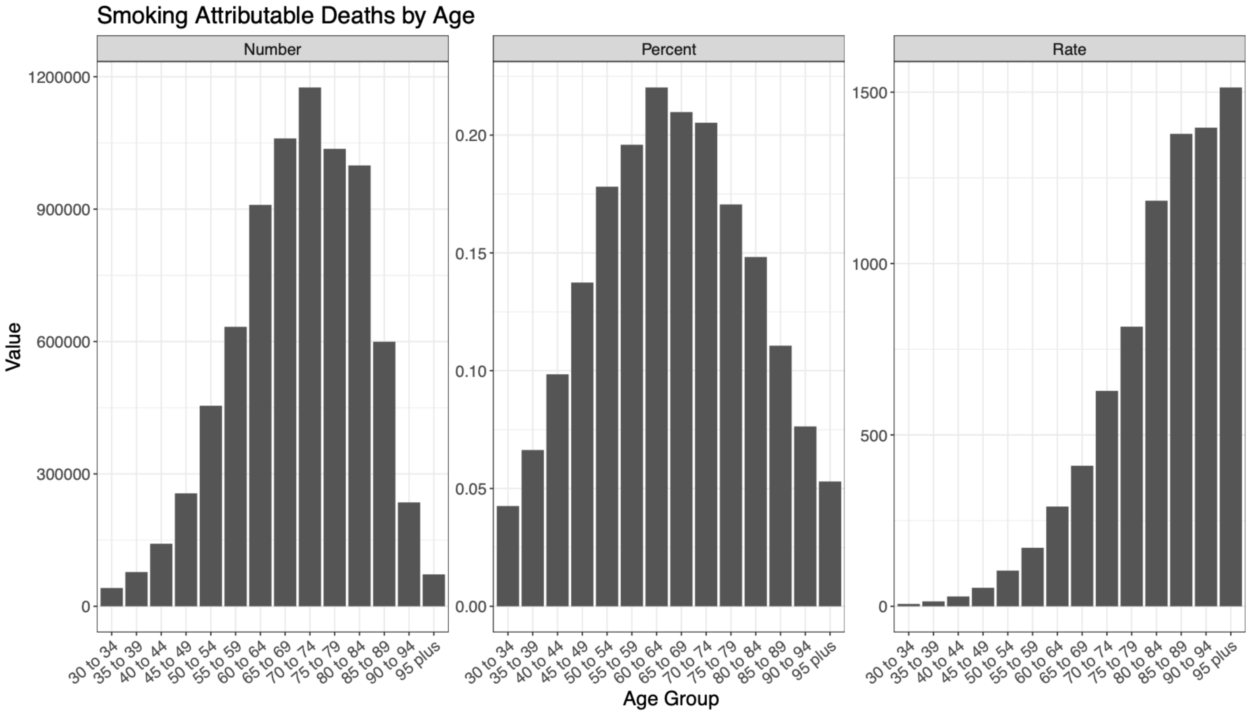

Supplemental Table S9. Percent change in number and share of all-age all-cause deaths attributable to smoking tobacco use, 1990-2019, by location.

Location	Percent Change in Number of Smoking Attributable Deaths, 1990-2019	Percent Change in Share of Deaths Attributable to Smoking, 1990-2019
**Global**	**31·1 (21·2–42·1)**	**8·17 (2·40–15·0)**
**Central Europe, Eastern Europe, and Central Asia**	**1·30 (-7·26–9·91)**	**-7·85 (-11·9–-3·47)**
Central Asia	32·0 (20·4–45·9)	8·65 (3·83–14·1)
Armenia	14·6 (-2·75–34·1)	-2·90 (-8·53–3·44)
Azerbaijan	80·4 (47·7–123)	39·0 (21·7–59·5)
Georgia	-24·3 (-37·3–-10·4)	-11·3 (-18·0–-2·93)
Kazakhstan	-1·29 (-15·4–15·4)	-7·90 (-14·6–0·500)
Kyrgyzstan	3·11 (-10·9–19·1)	3·05 (-3·90–11·0)
Mongolia	92·8 (47·0–155)	60·1 (44·7–78·6)
Tajikistan	29·5 (2·96–61·7)	9·20 (-4·57–24·8)
Turkmenistan	29·0 (2·70–62·4)	5·27 (-4·06–14·7)
Uzbekistan	139 (99·7–187)	53·6 (38·8–71·0)
Central Europe	-13·3 (-23·9–-2·50)	-17·6 (-20·7–-14·6)
Albania	60·8 (21·3–108)	23·8 (13·8–35·6)
Bosnia and Herzegovina	40·2 (12·2–74·2)	7·89 (0·766–15·4)
Bulgaria	-22·2 (-38·6–-2·81)	-29·2 (-34·3–-23·2)
Croatia	-25·9 (-41·0–-6·71)	-25·9 (-30·9–-20·5)
Czechia	-28·9 (-42·1–-13·7)	-19·6 (-25·2–-13·4)
Hungary	-20·7 (-34·4–-4·77)	-11·9 (-16·8–-6·68)
Montenegro	76·9 (48·1–111)	12·0 (2·79–22·2)
North Macedonia	46·0 (16·1–83·6)	0·924 (-7·42–10·8)
Poland	-17·2 (-30·9–-2·60)	-21·6 (-26·8–-16·1)
Romania	-13·7 (-29·5–3·10)	-19·4 (-24·9–-13·8)

Serbia	23·0 (-1·84–50·0)	1·26 (-5·06–8·24)
Slovakia	-24·6 (-40·0–-5·72)	-24·4 (-29·5–-18·9)
Slovenia	-4·32 (-33·1–32·7)	-14·8 (-20·8–-7·98)
Eastern Europe	6·66 (-5·67–19·8)	-3·05 (-9·48–4·07)
Belarus	-0·445 (-20·7–24·2)	-11·1 (-16·3–-5·74)
Estonia	-37·3 (-50·9–-21·5)	-24·7 (-30·7–-14·6)
Latvia	-38·6 (-49·3–-26·0)	-25·3 (-33·8–-16·4)
Lithuania	-24·8 (-38·1–-8·63)	-24·4 (-29·5–-19·0)
Republic of Moldova	-3·77 (-17·8–12·9)	0·188 (-8·97–10·9)
Russian Federation	12·4 (-4·18–30·3)	1·81 (-7·86–12·0)
Ukraine	0·968 (-16·4–19·8)	-10·5 (-20·8–-0·401)
**High-income**	**-5·94 (-8·45–-3·20)**	**-27·3 (-29·2–-25·3)**
Australasia	-23·1 (-27·2–-19·0)	-46·1 (-48·8–-43·3)
Australia	-23·3 (-28·0–-18·3)	-47·2 (-50·2–-44·0)
New Zealand	-22·7 (-27·0–-18·0)	-41·2 (-44·3–-37·9)
High-income Asia Pacific	15·8 (9·72–22·4)	-29·4 (-33·0–-25·4)
Brunei Darussalam	30·7 (10·9–54·7)	-23·8 (-31·1–-15·7)
Japan	14·0 (7·26–21·7)	-33·9 (-37·8–-29·8)
Republic of Korea	23·9 (13·6–37·1)	-7·76 (-14·8–1·37)
Singapore	3·32 (-5·51–12·9)	-37·4 (-42·7–-31·9)
High-income North America	2·01 (-1·41–5·60)	-26·7 (-29·2–-24·2)
Canada	-1·87 (-7·00–3·32)	-35·2 (-38·5–-32·0)
Greenland	21·2 (-1·94–45·2)	10·9 (3·49–20·0)
United States of America	2·39 (-1·30–6·28)	-25·9 (-28·5–-23·2)
Southern Latin America	10·2 (4·72–16·1)	-20·6 (-24·2–-16·8)
Argentina	10·5 (3·68–17·9)	-20·2 (-25·0–-15·1)
Chile	19·2 (9·58–30·1)	-20·0 (-25·9–-13·5)

Uruguay	-8·58<br>(-14·4–-2·28)	-20·0<br>(-24·7–-15·0)
Western Europe	-17·9<br>(-20·5–-15·2)	-27·0<br>(-29·1–-24·7)
Andorra	77·6<br>(25·7–138)	-29·3<br>(-35·6–-22·7)
Austria	-3·27<br>(-9·44–3·07)	-4·14<br>(-10·0–2·12)
Belgium	-18·1<br>(-22·6–-13·1)	-25·9<br>(-29·8–-22·0)
Cyprus	54·6<br>(37·6–74·9)	1·52<br>(-5·50–9·00)
Denmark	-27·1<br>(-31·0–-23·0)	-22·0<br>(-25·5–-18·4)
Finland	-23·9<br>(-30·1–-17·6)	-33·1<br>(-38·0–-27·7)
France	-6·21<br>(-12·2–-0·145)	-20·1<br>(-24·8–-15·1)
Germany	-22·1<br>(-27·5–-16·1)	-26·8<br>(-31·6–-21·3)
Greece	25·0<br>(18·1–33·0)	-11·1<br>(-15·5–-5·95)
Iceland	-11·2<br>(-20·8–-0·889)	-27·4<br>(-33·0–-21·4)
Ireland	-31·6<br>(-35·8–-27·2)	-34·9<br>(-38·2–-31·6)
Israel	7·63<br>(-0·865–17·2)	-34·6<br>(-39·2–-29·3)
Italy	-14·6<br>(-19·1–-9·53)	-29·3<br>(-33·0–-25·2)
Luxembourg	-14·3<br>(-25·0–-1·12)	-20·9<br>(-27·5–-13·5)
Malta	0·690<br>(-10·2–13·3)	-28·0<br>(-33·3–-22·5)
Monaco	11·4<br>(-11·2–40·8)	-7·09<br>(-15·5–2·50)
Netherlands	-14·3<br>(-19·1–-9·47)	-30·7<br>(-34·1–-27·1)
Norway	-40·9<br>(-45·6–-35·7)	-35·5<br>(-40·5–-30·1)
Portugal	-13·6<br>(-19·8–-6·58)	-26·2<br>(-30·9–-20·6)
San Marino	34·0<br>(-11·3–91·5)	-28·7<br>(-36·1–-21·1)
Spain	1·86<br>(-4·32–8·27)	-22·3<br>(-26·7–-17·9)
Sweden	-18·1<br>(-23·7–-11·9)	-18·6<br>(-23·9–-12·5)
Switzerland	-13·8<br>(-19·2–-6·99)	-23·7<br>(-28·4–-18·2)
United Kingdom	-33·9<br>(-36·6–-30·9)	-32·3<br>(-34·8–-29·3)
**Latin America and Caribbean**	**25·6<br>(16·4–35·2)**	**-15·9<br>(-19·7–-12·1)**

Andean Latin America	30·6 (6·95–56·5)	3·09 (-5·25–11·7)
Bolivia (Plurinational State of)	18·5 (-9·88–54·3)	-4·52 (-16·9–8·35)
Ecuador	46·3 (15·6–88·1)	-18·6 (-26·8–-9·36)
Peru	26·3 (-8·50–76·8)	16·3 (-0·224–34·4)
Caribbean	50·3 (30·4–74·0)	6·22 (-1·25–14·1)
Antigua and Barbuda	50·9 (25·2–80·6)	6·45 (-4·45–19·4)
Bahamas	68·0 (32·2–113)	-12·2 (-22·7–0·835)
Barbados	16·4 (-3·66–40·4)	-14·9 (-23·2–-5·38)
Belize	147 (106–193)	12·9 (1·42–25·6)
Bermuda	6·26 (-12·1–30·3)	-10·1 (-19·4–1·05)
Cuba	38·2 (13·9–67·6)	-8·01 (-14·5–-1·53)
Dominica	-10·2 (-26·3–10·3)	-15·7 (-24·2–-6·70)
Dominican Republic	211 (140–304)	71·4 (51·7–94·7)
Grenada	5·58 (-8·58–21·3)	6·27 (-4·92–18·7)
Guyana	-4·14 (-28·5–28·8)	-6·90 (-16·0–3·80)
Haiti	13·6 (-13·8–54·2)	-4·54 (-17·2–11·4)
Jamaica	55·7 (24·7–92·8)	1·50 (-6·32–10·6)
Puerto Rico	12·9 (-11·2–43·7)	-12·5 (-21·8–-2·13)
Saint Kitts and Nevis	6·75 (-13·3–30·5)	3·85 (-10·8–19·5)
Saint Lucia	44·9 (21·5–72·4)	-6·67 (-15·2–2·01)
Saint Vincent and the Grenadines	57·3 (32·0–86·4)	12·0 (-1·99–27·5)
Suriname	79·8 (47·7–118)	10·9 (0·552–23·1)
Trinidad and Tobago	4·88 (-22·4–38·7)	-26·1 (-32·3–-19·2)
United States Virgin Islands	115 (72·6–167)	8·85 (-6·08–25·4)
Central Latin America	39·3 (20·1–60·6)	-15·3 (-20·6–-9·82)
Colombia	13·2 (-13·5–44·7)	-22·2 (-29·7–-14·3)
Costa Rica	59·5 (25·8–102)	-25·6 (-31·3–-19·9)

El Salvador	35·1 (2·27–75·5)	10·1 (-2·27–23·8)
Guatemala	55·9 (14·8–109)	19·7 (1·33–42·5)
Honduras	173 (123–232)	43·5 (23·6–68·4)
Mexico	34·5 (12·6–58·4)	-19·8 (-28·0–-10·9)
Nicaragua	104 (64·8–147)	29·4 (13·7–46·5)
Panama	32·2 (0·103–70·7)	-33·5 (-40·2–-25·9)
Venezuela (Bolivarian Republic of)	64·3 (24·9–115)	-22·0 (-29·2–-14·6)
Tropical Latin America	14·7 (8·01–22·0)	-20·6 (-24·9–-16·0)
Brazil	13·4 (6·82–20·8)	-20·9 (-25·3–-16·3)
Paraguay	102 (55·6–163)	-0·939 (-11·0–10·3)
**North Africa and Middle East**	**72·5 (57·0–90·3)**	**41·3 (33·2–48·8)**
North Africa and Middle East	72·5 (57·0–90·3)	41·3 (33·2–48·8)
Afghanistan	99·2 (47·9–164)	44·2 (16·1–76·4)
Algeria	56·4 (21·0–101)	19·5 (6·88–34·5)
Bahrain	75·5 (35·9–127)	-19·3 (-28·4–-7·95)
Egypt	124 (75·4–186)	85·6 (68·1–104)
Iran (Islamic Republic of)	58·5 (44·1–76·0)	44·6 (30·5–61·4)
Iraq	106 (56·2–163)	36·5 (19·5–55·9)
Jordan	164 (104–236)	22·6 (5·78–41·5)
Kuwait	184 (130–249)	50·9 (36·0–69·1)
Lebanon	109 (72·2–148)	38·7 (24·3–53·4)
Libya	120 (68·6–192)	36·9 (24·3–52·5)
Morocco	58·1 (19·7–92·7)	23·1 (8·42–40·3)
Oman	5·27 (-19·6–41·1)	-16·6 (-28·3–-2·96)
Palestine	85·6 (44·2–146)	36·8 (18·8–58·9)
Qatar	280 (167–433)	13·3 (-2·56–33·6)
Saudi Arabia	187 (112–302)	84·9 (55·1–122)

Sudan	36·9 (5·24–78·7)	72·0 (42·7–107)
Syrian Arab Republic	56·7 (12·7–122)	36·2 (20·4–56·2)
Tunisia	98·0 (47·7–165)	26·0 (14·5–38·5)
Turkey	33·3 (5·62–67·1)	14·5 (4·97–25·2)
United Arab Emirates	614 (398–911)	41·3 (20·0–65·2)
Yemen	102 (53·0–177)	70·3 (39·7–111)
**South Asia**	**52·8 (30·1–78·4)**	**38·5 (23·5–54·9)**
South Asia	52·8 (30·1–78·4)	38·5 (23·5–54·9)
Bangladesh	21·2 (-4·19–51·8)	60·3 (44·3–80·3)
Bhutan	59·2 (19·0–121)	112 (71·9–176)
India	58·9 (30·7–90·8)	39·9 (22·5–59·7)
Nepal	71·8 (33·6–114)	97·4 (73·3–127)
Pakistan	37·8 (7·88–75·9)	9·51 (-11·3–31·6)
**Southeast Asia, East Asia, and Oceania**	**61·6 (36·7–95·9)**	**25·1 (13·1–41·4)**
East Asia	58·7 (27·8–101)	23·5 (9·42–41·4)
China	57·9 (26·2–101)	23·7 (9·29–42·1)
Democratic People's Republic of Korea	116 (72·6–165)	53·9 (37·9–70·1)
Taiwan (Province of China)	62·9 (28·7–110)	-9·12 (-15·4–-2·28)
Oceania	92·5 (56·9–136)	-1·90 (-11·5–8·95)
American Samoa	63·8 (37·0–92·1)	-4·31 (-13·2–5·09)
Cook Islands	18·7 (-3·26–43·0)	-4·52 (-14·5–6·07)
Fiji	25·2 (-7·24–65·8)	-22·7 (-28·8–-16·0)
Guam	92·9 (55·2–137)	-3·88 (-14·2–7·69)
Kiribati	62·5 (24·8–107)	31·6 (21·7–41·5)
Marshall Islands	71·8 (32·1–126)	19·6 (4·33–36·7)
Micronesia (Federated States of)	26·2 (-14·3–70·5)	12·4 (-14·1–28·8)
Nauru	-1·88 (-20·7–22·3)	6·60 (-5·51–20·2)

Niue	-23·4 (-39·5–-5·77)	-1·23 (-13·2–12·3)
Northern Mariana Islands	115 (77·7–156)	13·3 (0·0417–26·8)
Palau	67·0 (23·8–121)	3·65 (-7·92–17·4)
Papua New Guinea	122 (68·4–191)	3·34 (-11·0–20·3)
Samoa	26·7 (1·07–59·4)	-3·00 (-12·3–6·89)
Solomon Islands	90·2 (43·7–147)	8·69 (-1·78–20·2)
Tokelau	-32·4 (-46·5–-13·8)	-4·08 (-16·3–9·43)
Tonga	20·9 (-2·88–49·8)	-6·31 (-14·9–3·79)
Tuvalu	6·29 (-18·3–42·0)	6·32 (-5·28–19·5)
Vanuatu	109 (62·8–175)	3·30 (-7·67–18·2)
Southeast Asia	74·0 (54·5–95·9)	34·0 (25·1–43·9)
Cambodia	82·9 (46·1–120)	89·1 (67·8–111)
Indonesia	118 (74·0–171)	75·7 (51·8–103)
Lao People's Democratic Republic	14·6 (-11·2–47·0)	52·1 (33·2–77·5)
Malaysia	108 (66·2–156)	-2·75 (-9·66–4·25)
Maldives	33·1 (9·95–57·6)	27·5 (13·7–41·7)
Mauritius	1·46 (-17·8–23·5)	-35·0 (-39·6–-30·0)
Myanmar	-14·4 (-30·7–7·64)	5·31 (-6·74–18·6)
Philippines	131 (81·6–189)	33·8 (17·2–52·7)
Seychelles	39·7 (20·5–63·5)	-3·45 (-13·8–8·21)
Sri Lanka	5·92 (-21·0–38·7)	-17·9 (-24·6–-10·7)
Thailand	39·2 (3·39–85·1)	-15·1 (-22·1–-7·57)
Timor-Leste	185 (115–270)	186 (133–252)
Viet Nam	93·6 (54·1–141)	32·6 (21·1–46·9)
**Sub-Saharan Africa**	**34·1 (19·5–50·7)**	**19·8 (8·12–32·5)**
Central Sub-Saharan Africa	40·7 (12·4–75·9)	28·0 (11·4–48·1)
Angola	66·7 (24·4–133)	53·9 (25·6–98·2)

Central African Republic	29·8 (-0·546–69·2)	-9·58 (-22·2–6·56)
Congo	40·5 (7·12–81·5)	19·6 (2·88–37·5)
Democratic Republic of the Congo	33·3 (1·06–74·3)	24·6 (3·23–49·6)
Equatorial Guinea	-11·5 (-38·6–33·7)	-15·9 (-37·7–15·1)
Gabon	35·8 (3·22–71·8)	21·0 (3·42–42·8)
Eastern Sub-Saharan Africa	28·2 (13·1–46·5)	40·3 (25·5–58·9)
Burundi	-22·1 (-41·1–6·65)	-10·7 (-24·9–8·26)
Comoros	27·7 (-5·98–115)	37·4 (8·41–126)
Djibouti	246 (152–385)	89·5 (52·2–134)
Eritrea	50·7 (7·22–139)	78·7 (39·7–170)
Ethiopia	-40·9 (-55·4–-19·0)	-4·22 (-26·2–30·5)
Kenya	119 (75·7–172)	43·6 (19·7–73·5)
Madagascar	-2·89 (-28·4–26·2)	-11·3 (-22·8–1·45)
Malawi	50·5 (19·3–90·5)	106 (71·5–152)
Mozambique	66·2 (27·6–115)	32·4 (8·03–62·5)
Rwanda	11·7 (-10·7–43·2)	81·5 (50·4–123)
Somalia	80·2 (33·0–153)	14·4 (-7·21–44·0)
South Sudan	2·74 (-25·2–41·8)	11·1 (-16·5–47·7)
Uganda	62·4 (27·0–106)	94·8 (56·7–141)
United Republic of Tanzania	55·0 (26·0–88·8)	47·8 (27·5–69·9)
Zambia	62·6 (25·8–108)	50·3 (22·0–81·2)
Southern Sub-Saharan Africa	21·2 (10·1–32·9)	-30·6 (-35·9–-24·7)
Botswana	72·0 (26·1–127)	-15·8 (-27·9–-1·73)
Eswatini	30·9 (-3·60–78·1)	-28·7 (-40·8–-13·9)
Lesotho	65·9 (27·0–112)	-9·32 (-21·3–3·61)
Namibia	14·3 (-9·16–46·7)	-26·2 (-36·4–-13·1)
South Africa	10·2 (0·259–22·4)	-37·9 (-43·6–-31·9)

Zimbabwe	62·2 (27·2–100)	0·657 (-15·5–23·0)
Western Sub-Saharan Africa	52·6 (28·0–81·5)	23·0 (5·19–41·9)
Benin	27·8 (-3·01–66·9)	-8·32 (-23·8–10·3)
Burkina Faso	54·8 (21·6–99·3)	21·1 (-3·13–51·2)
Cabo Verde	35·2 (15·4–57·7)	-3·10 (-13·3–9·12)
Cameroon	100 (51·4–166)	12·6 (-7·37–33·8)
Chad	64·2 (24·2–117)	3·58 (-18·5–32·1)
Côte d'Ivoire	105 (54·7–167)	60·5 (29·6–97·1)
Gambia	90·9 (39·5–163)	37·6 (6·27–77·7)
Ghana	66·6 (27·2–116)	20·5 (0·343–45·9)
Guinea	88·6 (44·4–145)	79·5 (53·7–111)
Guinea-Bissau	-1·77 (-27·2–30·6)	13·7 (-5·12–37·9)
Liberia	4·86 (-21·9–42·7)	35·7 (6·59–73·3)
Mali	125 (75·5–190)	80·1 (53·9–113)
Mauritania	7·62 (-22·1–43·2)	17·0 (-11·0–40·1)
Niger	163 (98·6–260)	111 (72·0–165)
Nigeria	20·2 (-14·7–71·8)	1·04 (-25·7–34·4)
São Tomé and Príncipe	90·8 (51·7–148)	107 (73·9–160)
Senegal	36·3 (2·92–84·0)	30·8 (1·17–69·9)
Sierra Leone	9·49 (-17·2–44·6)	-0·00937 (-14·5–19·2)
Togo	94·7 (48·8–154)	42·5 (16·7–74·0)
